# Population genetic characteristics of Hainan medaka with whole-genome resequencing

**DOI:** 10.3389/fgene.2022.946006

**Published:** 2022-10-12

**Authors:** Zebin Yao, Shuisheng Long, Chun Wang, Chengqin Huang, Hairui Zhang, Liao Jian, Jingru Huang, Yusong Guo, Zhongdian Dong, Zhongduo Wang

**Affiliations:** ^1^ Guangdong South China Sea Key Laboratory of Aquaculture for Aquatic Economic Animals, Fisheries College, Guangdong Ocean University, Zhanjiang, China; ^2^ Guangdong Provincial Engineering Laboratory for Mariculture Organism Breeding, Fisheries College, Guangdong Ocean University, Zhanjiang, China; ^3^ Guangdong Provincial Key Laboratory of Pathogenic Biology and Epidemiology for Aquatic Economic Animals, Fisheries College, Guangdong Ocean University, Zhanjiang, China

**Keywords:** *Oryzias curvinotus*, population genetics, genetics structure, population history, positive selection

## Abstract

The *DMY* gene is deleted in all males of the Sanya population (SY-medaka) of the Hainan medaka, *Oryzias curvinotus*, as recently reported by us. However, due to limited knowledge regarding their population genetic background, it is difficult to explore the possible evolutionary pathway. Herein, we resequenced the whole genome of four populations, including SY-medaka. A total of 56 mitogenomes and 32,826,105 SNPs were identified. We found that the genetic differentiation is highest between SY-medaka and the other populations. The results of the population history of the *O. curvinotus* suggest that the SY-medaka has been in a bottleneck period recently. Further analysis shows that SY-medaka are the most strongly affected by environmental selection. Moreover, we screened some potential genomic regions, and the genes contained in these regions may explain the potential mechanism of the selection process of the SY-medaka. In conclusion, our study can provide new clues for the adaptation process of medaka in the new environment of Sanya.

## Introduction

The *Oryzias* has always been a species of interest for genetics and evolutionary biology, and research on the genetic diversity of *Oryzias* has been reported continuously. In terms of morphology and physiology, when the medaka was first studied, it was found that there were significant differences in the average number of anal fins of *Oryzias latipes* in different regions (Egami, 1954), while *O. latipes* in high latitudes grew faster than those in low latitudes (Yamahira and Takeshi, 2008). Genetic diversity among medaka has also been observed at the cytogenetic level; for example, there are differences in karyotypes among related species (Parenti, 2008). In addition, it was also found that *Oryzias* has two different sex determination systems: XX/XY and ZZ/ZW (Naruse et al., 2011). However, most relevant reports focus on *O. latipes*, the first reported teleost with a male sex-determining gene, *dmy* (Hirayama et al., 2010; Spivakov et al., 2014; Katsumura et al., 2019; Sutra et al., 2019).

**FIGURE 1:**
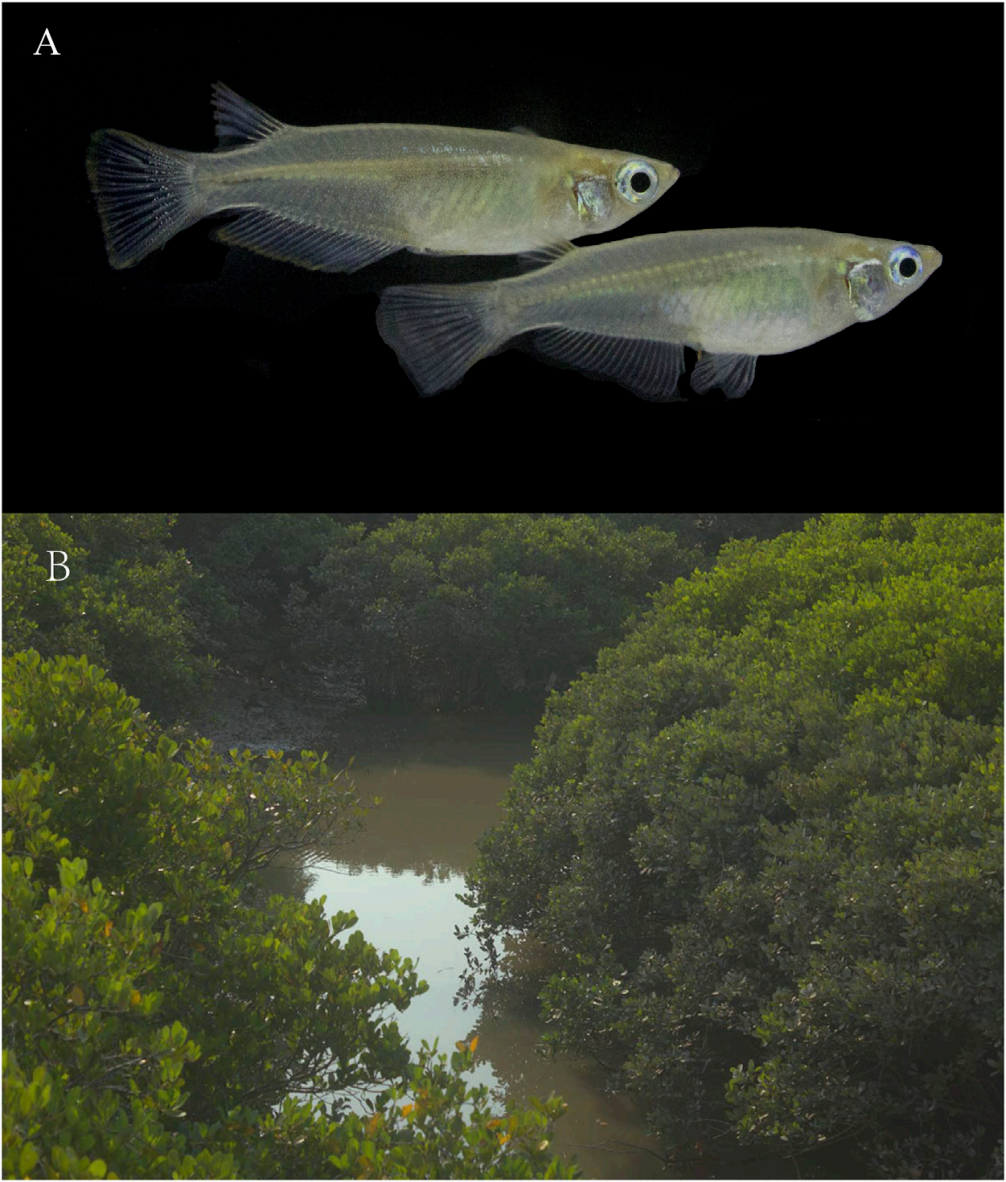
*Oryzias curvinotus*. **(A)** Male (upper) and female (lower) *Oryzias curvinotus* form Sanya. **(B)** Natural habitat of Hainan medaka.

**FIGURE 2:**
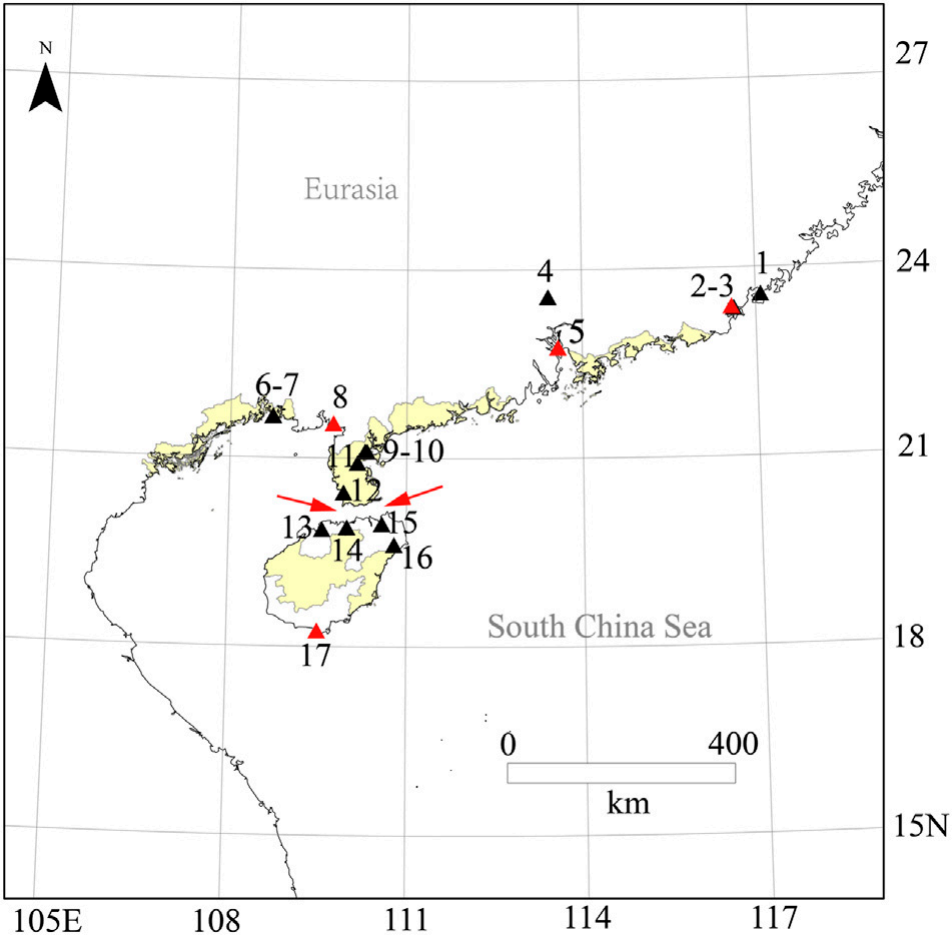
Species distribution of *O. curvinotus*. Species distribution map for *O. curvinotus* and some new investigation sites; 1, Raoping; 2, Dahao; 3, Niutianyang (ST); 4, Conghua; 5, Nansha (JWM); 6, Zhonggui; 7, Yamchow; 8, Gaoqiao (GQ); 9, Huguang; 10, Donghai dao; 11, Fucheng; 12, Leizhou; 13, Lingao County; 14, Chengmai; 15, Dongzhaigang; 16, Wenchang; 17, Sanya (SY); The location IDs are in common with Supplementary Table S2; Four collection sites of *O. curvinotus* for WGS are marked with red triangles. The red arrow refers to the Qiongzhou Strait.

As a closely related species of *O. latipes*, *O. curvinotus* inhabits mangrove forests along the coast of the South China Sea (Figures 1, 2). Moreover, *O. curvinotus* is the second species in genus *Oryzias* containing *dmy* (Matsuda et al., 2003). Especially, we have found that males of the Sanya population (SY-medaka) from Hainan Island generally lack *dmy* gene, which is obviously different from mainland populations such as the Gaoqiao population (GQ-medaka) (Dong et al., 2021). In addition, some phenomena of physiological differences have also been observed, for example, the population differences in the speeds of body color change (Supplementary Figure S2, unpublished) and the inconsistent growth rates (Wang, 2020) (Supplementary Figure S3). Obviously, the SY population of *O. curvinotus* may be different from other populations both in physiological and genetic levels. In particular, geographically, the SY-medaka is separated from the mainland population by the Qiongzhou Strait, which might hinder the gene flows of medaka because of the small body size, the low migrating ability and the bunched eggs by the filaments. Additionally, the climatic conditions also are different, such as water temperature by the latitudinal differences (Supplementary Figure S1). Thus, it is easy to associate external environmental factors with the effects on the various geographic population of the *O. curvinotus*. However, the lack of knowledge regarding population genetic background makes it difficult to reveal the biological mechanism of the above phenomena in *O. curvinotus*. Therefore, there is an urgent need to investigate the available gene pool and population genetics of *O. curvinotus*.

Previous studies of genetic diversity and population structure have often been based on mitochondrial genome (mtDNA) (Hirayama et al., 2010; Mokodongan and Yamahira, 2015). But mtDNA was considered insufficient to fully describe population structure and history because of its single locus (Katsumura et al., 2019). Single nucleotide polymorphisms (SNPs) are considered an effective genetic marker for researching the genetic diversity and adaptive evolution of species. In recent years, as sequencing technology has become more mature and economical, the nuclear genomes of a large number of species have been successfully assembled. Whole genome sequencing (WGS) and SNPs detection have also been widely used. In vertebrates, SNPs occur almost once in every 1,000 nucleotides on average in human DNA, and some are strongly correlated with traits or genetic diseases (Kujovich, 2011; Ji et al., 2012; Ezzeddini et al., 2021). In addition, SNPs in many animals such as fish are associated with environmental adaptation. Recently, many potential genes related to environmental adaptation in fish have been discovered by using SNPs (Jones et al., 2012; Araki et al., 2018; Gaither, Gkafas, De Jong, et al., 2018; Narum et al., 2018; Onainor, 2019; Cádiz et al., 2020), which can usually provide some new clues for species evolution and environmental adaptation research.

Here, we performed a batch of whole-genome re-sequencing of the wild *O. curvinotus* from four geographic groups at different latitudes with the aim of obtaining a high density of SNPs and also analyzed the population structure and evolutionary history of *O. curvinotus* at the nuclear gene level.

## Materials and methods

### Sampling and sequencing

All the 56 wild individuals sequenced in this study were collected from four geographical locations (Figure 2, Supplementary Table S2) along the south coast of China with mangroves, of which Shantou (ST, E116.57° N23.38°), Jiuwangmiao (JWM, E113.58° N22.76°), and Gaoqiao (GQ, E109.74° N21.55°) are located in Guangdong Province, and Sanya (SY, E109.50° N18.25°) is located in Hainan Province.

After collection of each population, all the samples were euthanized and stored in absolute ethanol. Genomic DNA was extracted using the phenol-chloroform method. The fragment size of the sequencing library was 350 bp, and sequencing was conducted on the Illumina HiSeq Xten platform using terminal pairing (PE) 150 bp reads. The sequencing depth of each sample was ×10. The resequencing raw data of all samples in this study have been uploaded to the NCBI Sequence Reading Archive (SRA) with storage number SRR17331594-SRR17331649.

### Read mapping and variant calling

Before mapping the resequenced data to the reference genome, we filtered the adapter sequence and low-quality reads of all original data to obtain high-quality clean data. BWA software (parameters: mem, -T 4, -K 32, -M) was used to compare sequencing results to the reference genome (GQ-medaka, Sequences have been submitted to GenBank, PRJNA821560) of *O. curvinotus*, and GATK4 (MarkDuplicates) software was used to remove PCR duplication (McKenna et al., 2010; Li, 2013).

The standard process of GATK4 software was used to identify variation and extract SNPs. High quality SNP data was filtered using GATK4 (VariantFiltration, default parameters) and VCFtools (parameters: -max-missing 0.5, -maf 0.01, -minDP 3, -minQ 30) (Danecek et al., 2011).

### Annotation

Gene-based SNP annotation was performed according to the annotation of the *O. curvinotus* genome using the package ANNOVAR (Wang et al., 2010). Based on the genome annotation, SNPs were categorized as occurring in exonic regions (overlapping with a coding exon), intronic regions (overlapping with an intron), splice sites (within 2 bp of a splicing junction), upstream and downstream regions (within a 2 kb region upstream or downstream from the transcription start site), or intergenic regions. SNPs in coding exons were further grouped as either synonymous SNPs or nonsynonymous SNPs. Additionally, mutations causing gain or loss of a stop codon were also classified as nonsynonymous SNPs. It should be noted that the statistical results for SNP classification may be redundant due to the overlap of genes.

### Phylogenetic tree

The P-distance matrix was calculated based on the all SNPs data by VCF2Dis (https://github.com/BGI-shenzhen/VCF2Dis) and the NJ-tree was built with 1000 bootstrap by PHYLIP 3.69 (http://evolution.genetics.washington.edu/phylip.html).

### Mitochondrial genome assembly and mtDNA tree

The open source toolkit, GetOrganelle (Jin et al., 2018) were used to assemble the mitochondrial genome based on the reads from the resequenced datasets. Each dataset of 56 samples was completely assembled into a circular sequence. The assembly results were confirmed to be accurate by re-mapping to the assembled mitochondrial sequence. Mitochondrial sequences of other species of the *Oryzias* were obtained from NCBI (Supplementary Table S3), multiple alignment were performed using the muscle method, and the maximum likelihood (ML) tree was built with 1000 bootstrap by MEGA (Kumar et al., 2018).

### Principal component analysis

All SNPs of 56 individuals were used for principal component analysis by GCTA software (Yang et al., 2011). The first three important principal components were retained and graphed. The discrete points reflect the real structure of the population to a certain extent.

### Population structure

To analyze the population structure, the program Admixture was used to estimate ancestry number in a model-based manner from all SNP genotype datasets. To explore the convergence of individuals, we predefined the number of genetic clusters from K = 3 to K = 6.

### Linkage disequilibrium analysis

To estimate and compare the pattern of linkage disequilibrium (LD) of each population, the squared correlation coefficient (r^2^) values between any two SNPs within 100 kb were computed by using the software PopLDdecay (Zhang et al., 2019). For plotting the LD attenuation curve, the SNP data of 56 individuals were divided into four groups according to geographical location, and the average r^2^ of each group was calculated.

### Demographic history reconstruction

PSMC software was used to construct the historical dynamics of the four populations of *O. curvinotus* (Li and Durbin, 2011). PSMC is based on the results of individual resequencing alignment and considers all heterozygous loci of an individual, not just SNPs. When estimating the population historical dynamics of *O. latipes*, it is assumed that the doubling time of each generation is 0.67 years and the mutation rate is 2.5 × 10^−8^ (Spivakov et al., 2014). However, the estimation results of our data based on these parameters are not very reliable. Therefore, in this study, to ensure that all possible results are considered, we used as many parameters as possible for estimation. According to the sexual maturity time of *O. curvinotus* and field investigation, multiple values were used for the mutation rate, ranging from 0.25 × 10^−8^ to 2.5 × 10^−8^, and the generation intervals used were 0.25, 0.33, 0.5, 0.66, or 1 year. Default values were taken for other parameters.

### Polymorphism levels and selection analyses

*F*_ST_ values between populations were calculated using vcftools software. In order to identify potential selected genes, we counted the θπ ratios and *F*_ST_ values in 40 kb windows in 20 kb steps. For comparing groups, the regions with maximum *F*_ST_ values (top 5%, as outliers) and maximum θπ ratio (top 5%, as outliers) were identified as selected regions. We used clusterProfiler to preform KEGG enrichment analysis on the candidate selective gene located in selected regions.

## Results

### Sequencing results and single nucleotide polymorphism identification and annotation

In this research, *O. curvinotus* from the four geographical groups are abbreviated as ST-medaka, JWM-medaka, GQ-medaka, and SY-medaka. Genome mapping of all individuals across the *O. curvinotus*’s 800 Mb genome resulted in an average of 98.21% sequencing coverage (Supplementary Table S1). Through identification and screening, we identified a total of 32,826,105 SNPs. Among them, 875,581 loci were located in exonic regions, 509,253 were synonymous variations, and 355,894 were nonsynonymous variations. Other basic statistical results are shown in Table 1.

**TABLE 1:** Statistics for SNP annotation results.

Category	Number of SNPs
Exonic	875,581
Splice site	1,853
Upstream (2 kb)	1,346,688
Downstream (2 kb)	1,171,335
Upstream/downstream	325,702
Intergenic	13,595,255
Intronic	14,750,035
3′ UTR	574,339
5′ UTR	184,901
3′UTR/5′UTR	414
Category of exon	Number of SNPs
Synonymous	509,253
Nonsynonymous	355,894
Stop-gain	3,796
Stop-loss	683

### Population genetic structure and phylogeny

**FIGURE 3:**
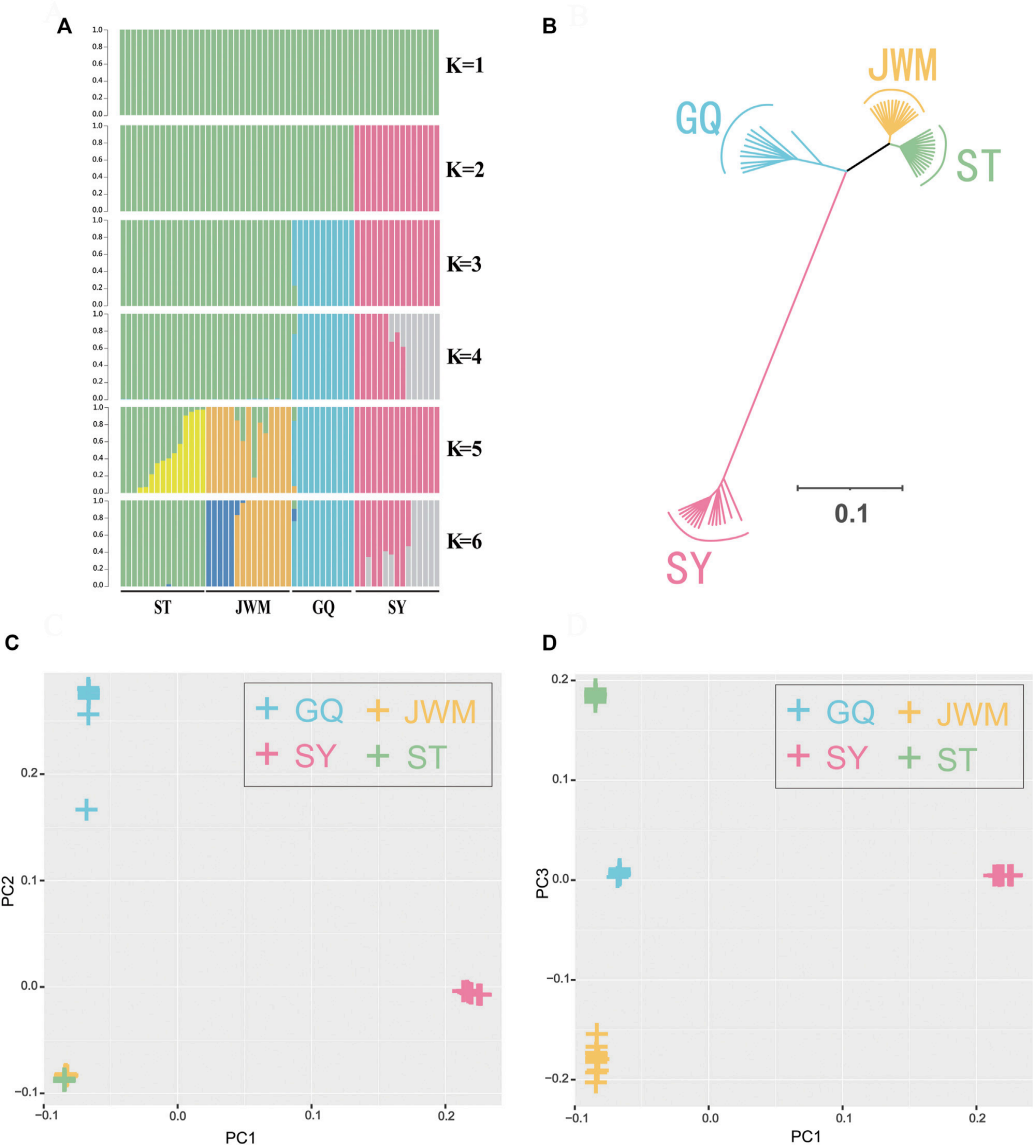
Population genetics. **(A)** Genetic structure of the *O. curvinotus* as inferred by Admixture analysis. The number of populations (K) from 1 to 6 is shown. Each color represents a different hypothetical ancestor. **(B)** Unrooted tree generated by the neighbor-joining method with 1000 bootstrap. **(C,D)** The principal component analysis (PCA) of all individuals.

The population genetic structure (Figure 3A) shows the population structure of the 56 individuals for population numbers (K) from 1 to 6. When K is equal to 2, 3 continental population are grouped, SY-medaka was separated. When K is equal to 3 or 4, ST-medaka and JWM-medaka are grouped. Although JWM-ST and JWM-GQ are almost equal in geographical straight-line distance, JWM-medaka was grouped with GQ-medaka. When K is equal to 4 or 5, JWM-medaka and ST-medaka are forced to be separated. When K is 4 or 6, the ancestral sequences of SY-medaka are separated, while the GQ-medaka can always be clearly separated. Statistical support for the different number of clusters was evaluated based on fivefold cross-validation implemented in Admixture (Supplementary Figure S4). The grouping of subgroups at the CV-error minimum is relatively reasonable. Based on the value at the CV-error minimum, the four populations of *O. curvinotus* are divided into three subgroups.

**FIGURE 4:**
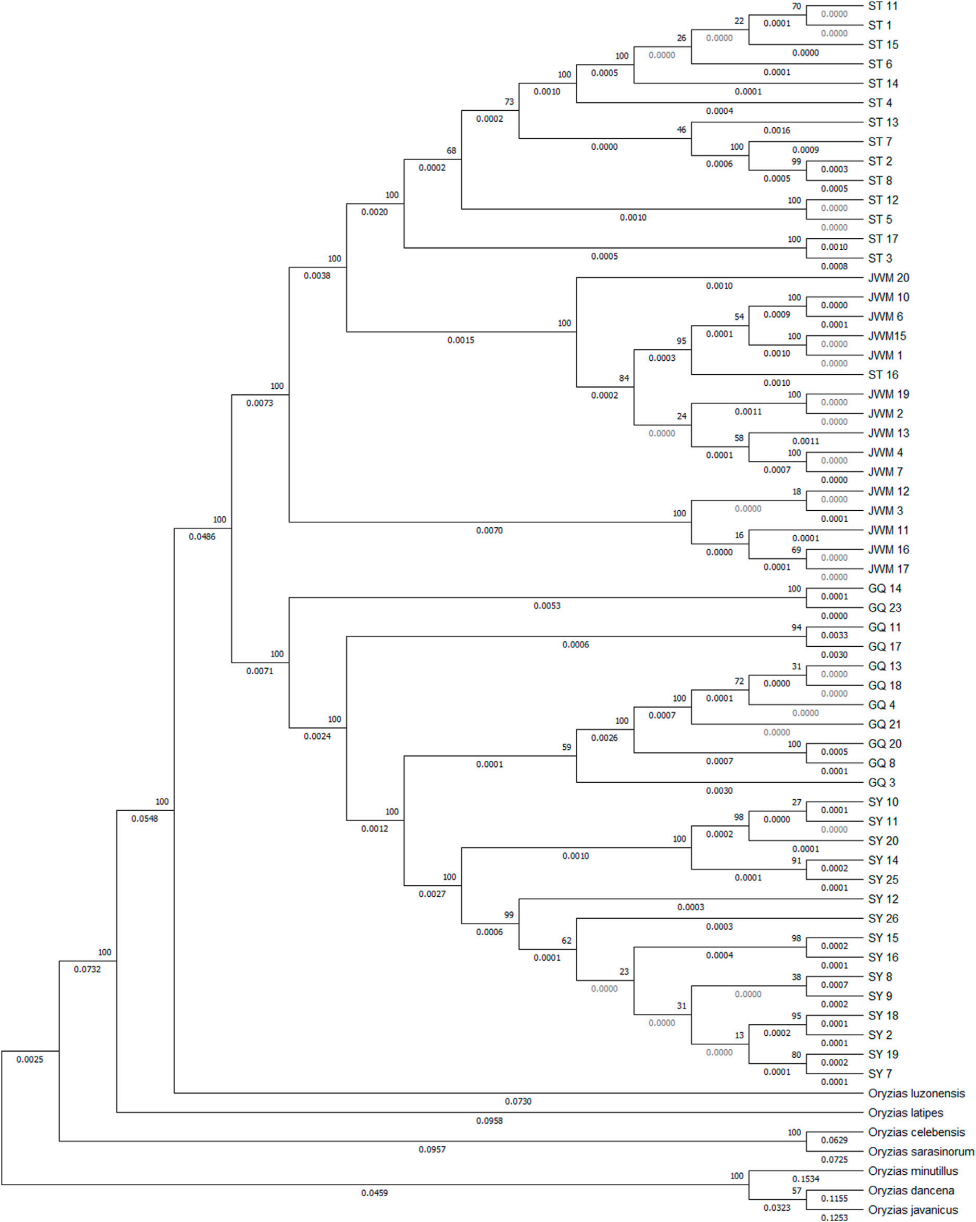
Based on the ML tree of mitochondrial whole sequences, some other fish of the *Oryizas* genus were used as outgroups. The bootstrap value (above branch) and branch lengths (below branch) are shown here.

The VCF2Dis software was used to calculate the genetic distance between all individuals, and the neighbor-joining (NJ) method was used to construct the evolutionary tree. The unrooted tree (Figure 3B, Supplementary Figure S9) demonstrates that the genetic distance between ST-medaka and JWM-medaka is the shortest, and their genetic distance with SY-medaka is the longest, followed by their distance to GQ-medaka. Based on the complete mitochondrial sequence, we constructed the ML tree (Figure 4). Here, other species of *Oryzias* were used as the outgroup. Apart from the clustering of one individual (ST 16) that was different from the NJ tree based on SNPs, all other individuals formed clusters with other local individuals. This demonstrates that the genetic structure analysis based on SNPs is reliable. In addition, the result of ML tree indicate that *O. curvinotus* first differentiated into two branche. One branch includes ST-medaka and JWM-medaka, and the other branch includes GQ-medaka and SY-medaka.

The first three principal components, namely first principal component (PC1), second principal component (PC2), third principal component (PC3) were extracted and plotted. In particular, the PC1 (variance explained = 48.44%) separated the SY-medaka from the other populations, the PC2 (variance explained = 11.93%) separated the GQ-medaka from the other populations (Figure 3C). The PC3 (variance explained = 2.70%) indicated that ST-medaka and JWM-medaka were separated (Figure 3D). The PC3 were extracted and plotted. The variance interpretation sum of the first three principal components was 63.07%.

### Population history of *O. curvinotus*

**FIGURE 5:**
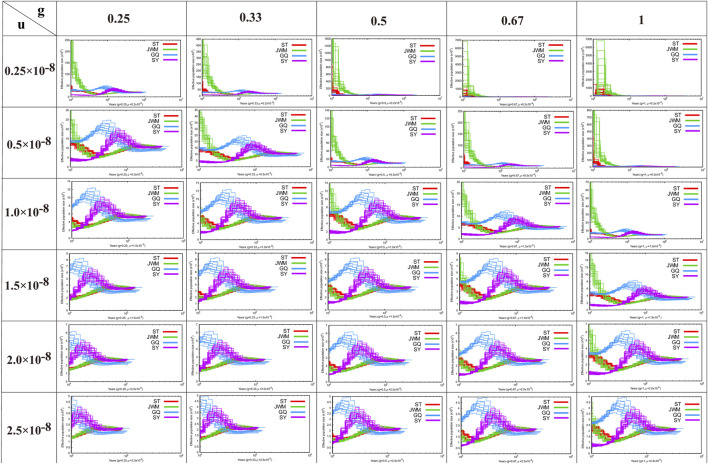
The results of the analysis of the demographic history of the *O. curvintous* are presented. Multiple values ranging from 0.25 × 10^−8^ to 2.5 × 10^−8^ were used for mutation rates (u). The generation intervals (g) were 0.25, 0.33, 0.5, 0.66, and 1 year based on the sexual maturity time of the medaka and field surveys.

In Figure 5, we show the estimation results of historical population dynamics for different possible generation intervals and nucleic acid mutation rates. The results show that the historical dynamic results of most *O. curvinotus* are similar, and some poor results only appear for more extreme parameter values. Taking the estimate of *g* = 0.67, *μ* = 2.5 × 10^−8^ as an example, the effective population sizes of all subgroups of medaka were basically the same until about 100,000 years ago, when the common ancestor of them had not yet diverged. Therefore, the effective population sizes dating back to this period are basically the same. During the period from about 40,000 to 100,000 years ago, the population size changes showed two trends, one is an upward trend containing SY-medaka and GQ-medaka, and the other is a downward trend containing JWM-medaka and ST-medaka, suggesting that two branches of the medaka diverged during this period. While the trends in effective population sizes of WM-medaka and ST-medaka remained consistent and the population sizes were very similar from approximately 10,000 to 40,000 years ago. In contrast, the trends of the other two populations were clearly distinguished, with GQ-medaka showing an upward and then a downward trend, while SY-medaka showed a sharp decline. All four populations of the *O. curvinotus* live along the coast of the South China Sea, but the demographic changes of several of them are very different, which may be due to the different effects of geological events and climatic environmental changes on them.

### Linkage disequilibrium analysis

**FIGURE 6:**
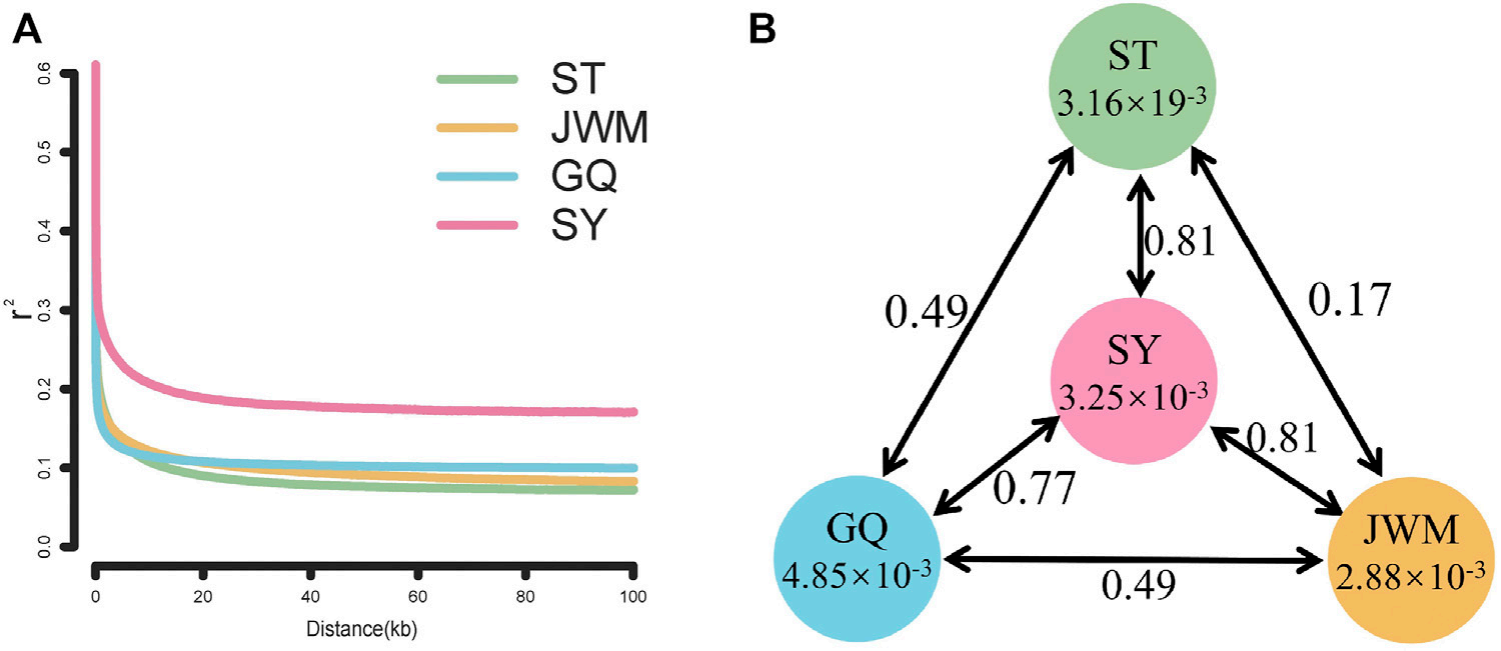
**(A)** Linkage disequilibrium patterns of four populations. **(B)** *F*_ST_ values among populations and θπ in each population.

The LD attenuation diagram (Figure 6A) shows that the LD attenuation speed of each *O. curvinotus* population is very fast and stabilized at an attenuation distance of about 20 kb. In addition, the decay rates of the ST-medaka, JWM-medaka, and GQ-medaka are similar, but significantly lower than that of SY-medaka, indicating that SY-medaka may be under stronger selection pressure. It is worth noting that the LD value of the final convergence of the four groups is correlated with the latitude. At lower latitudes, the stable LD value of each group was higher.

### *θπ* and differentiation index (*F*_*ST*_)

Genome-wide scans were performed using the sliding-window approach, then θπ and *F*_ST_ were calculated between the populations (Supplementary Figures S5, S6). The results showed that SY-medaka had the highest *F*_ST_ value of 0.81 with the other populations, and the smallest *F*_ST_ between the ST-medaka and JWM-medaka (Figure 6B). In addition, GQ-medaka had the highest θπ of 4.85 × 10^−3^, SY-medaka was the second highest at 3.25 × 10^−3^, ST-medaka and JWM-medaka were the least at 3.16 × 19^−3^ and 2.88 × 10^−3^, respectively. The results indicated that SY-medaka produced a large genetic differentiation. Furthermore, that of SY-medaka depicted a highly genetically differentiated population from other populations, suggesting that SY-medaka may have been subjected to strong selection.

### Selected gene

**FIGURE 7:**
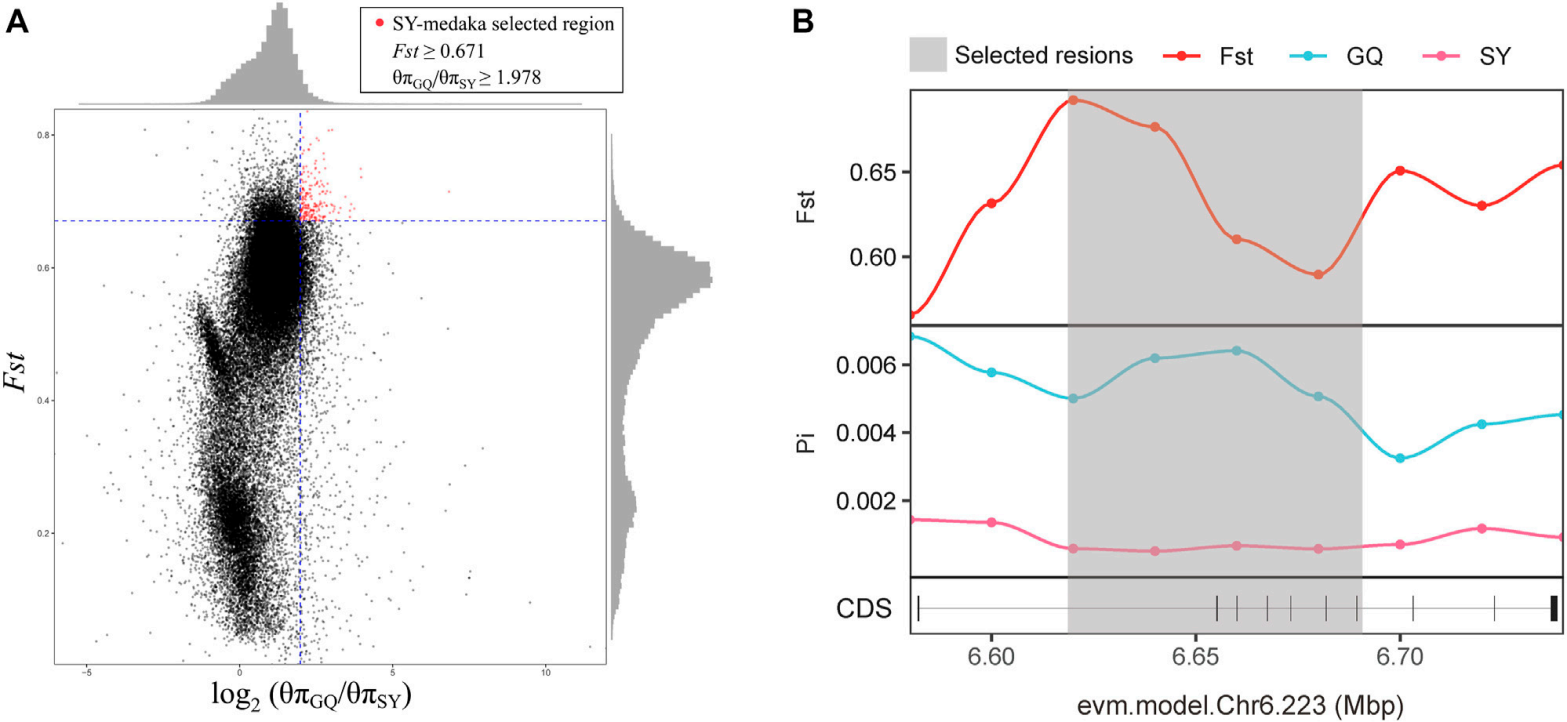
**(A)**The distribution of the θπ ratios (θπGQ-medaka/θπSY-medaka) and *F*_ST_ values (GQ-SY), calculated in 20-kb windows sliding in 10-kb step. Data points on the right of the vertical dashed line (corresponding to the 5% left tail of the empirical θπ ratio distribution), and above the horizontal dashed line (5% right tail of the empirical *F*_ST_ distribution) were identified as selected regions for SY-medaka (red points). **(B)** Examples of genes with strong selective sweep signals in GQ-medaka and SY-medaka. *F*_ST_ and θπ values are plotted using a 10-kb sliding window. Shaded genomic regions were the regions with strong selective signals for SY-medaka.

**FIGURE 8:**
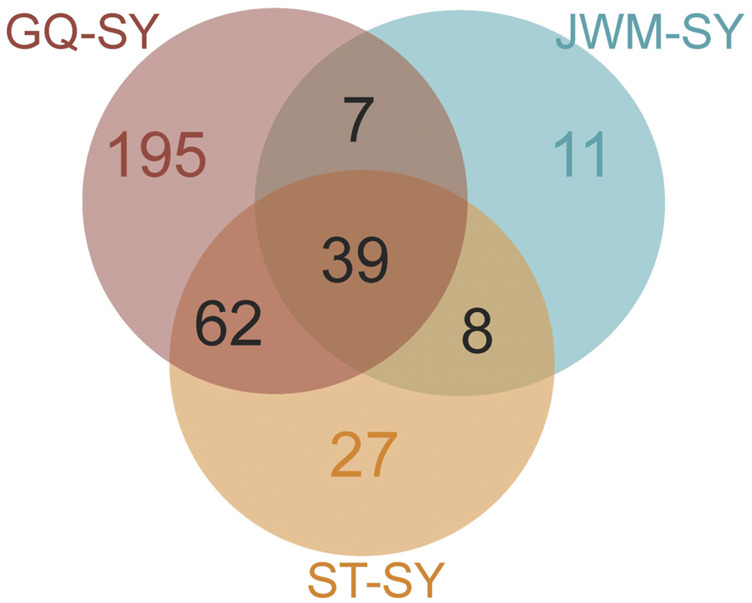
Venn diagram showing the intersection of the number of genes subject to selection in SY-medaka relative to other populations.

In order to identify potential selected genes in SY-medaka, and to detect regions with significant signatures of a selective sweep, we considered the distribution of the θπ ratios and *F*_ST_ values. We selected windows simultaneously with significant high θπ ratios and significant high *F*_ST_ values of the empirical distribution as regions with strong selective sweep signals along the genome (Figure 7, Supplementary Figures S7). Through the intersection of the top 5% windows of *F*_ST_ values and θπ ratios, we screened some strong selection signals of the three subpopulations, in which 136 genes were obtained by comparing ST to SY (ST-SY), 65 genes were obtained for JWM-SY, 303 genes were obtained for GQ-SY, and 39 candidate genes were obtained by taking the intersection of the three groups of genes (Figure 8). 39 selected genes of SY-medaka participate in multiple biological processes, which may indicate the potential processes through which *O. curvinotus* have adapted to the new environment of Sanya. We found that the annotation information of six genes was incomplete, and their specific functions need to be further studied. We performed KEGG enrichment analysis on the other 33 candidate genes to further explore the potential mechanism by which *O. curvinotus* adapted to the new environment of Sanya (Table 2). KEGG enrichment results showed that 12 genes were enriched in KEGG pathways, and only three genes (EVM.Model.Chr6.217, EVM.Model.Chr23.722, and EVM. Model.Chr23.721) were significantly enriched in two pathways (ko04711: circadian rhythm—fly; ko04620: Toll-like receptor signaling pathway).

**TABLE 2:** Functional pathway enrichment for selected genes of SY-medaka.

Gene name	Gene ID in genome	Gene annotation	KEGG path
ATF7IP	EVM.Model.Chr1.310	Activating transcription factor 7-interacting protein 1	—
txnl4a	EVM.Model.Chr11.1183	Thioredoxin-like protein 4A	—
KAF6729704.1	EVM.Model.Chr11.1185^a^	-	—
trit1	EVM.Model.Chr11.1188	tRNA dimethylallyltransferase	—
mycl	EVM.Model.Chr11.1189	Protein L-Myc-1b	—
mfsd2a	EVM.Model.Chr11.1190	Sodium-dependent lysophosphatidylcholine symporter 1-B	—
stk3	EVM.Model.Chr11.497	Serine/threonine-protein kinase 3	—
ridA	EVM.Model.Chr11.498	2-iminobutanoate/2-iminopropanoate deaminase	—
CYCS	EVM.Model.Chr11.499	—	—
rpl30	EVM.Model.Chr11.500	60S ribosomal protein L30	Ribosome, Coronavirus disease
LAPTM4B	EVM.Model.Chr11.501	Lysosomal-associated transmembrane protein 4B	Lysosome
RRM2B	EVM.Model.Chr11.502	Ribonucleoside-diphosphate reductase subunit M2	Purine metabolism
			Pyrimidine metabolism
			Glutathione metabolism
			Drug metabolism, p53 signaling pathway
			DNA Repair and Recombination Proteins
DTNBP1	EVM.Model.Chr11.508	Dysbindin	Membrane trafficking
RVE67048.1	EVM.Model.Chr11.514^a^	—	—
CEP192	EVM.Model.Chr16.896	Centrosomal protein of 192 kDa	—
Ankyrin-3-like isoform X1	EVM.Model.Chr19.33	Ankyrin-3	—
TNPO3	EVM.Model.Chr23.720	Transportin-3	Nucleocytoplasmic transport, Transfer RNA biogenesis
IRF5	EVM.Model.Chr23.721	Interferon regulatory factor 5	Toll-like receptor signaling pathway, Transcription factors
IRF5	EVM.Model.Chr23.722	Interferon regulatory factor 6	Toll-like receptor signaling pathway, Transcription factors
XP_011491718.1	EVM.Model.Chr23.723^a^	—	—
CDHR5-like isoform X1	EVM.Model.Chr23.724	Cadherin-related family member 5	Cell adhesion molecules
D (4) dopamine receptor-like	EVM.Model.Chr23.725	D (4) dopamine receptor	Neuroactive ligand-receptor interaction, Dopaminergic synapse, G-Protein Coupled Receptors
MBC8529904.1	EVM.Model.Chr23.726^a^	—	—
TSPAN12	EVM.Model.Chr23.745	Tetraspanin-12	—
ING3	EVM.Model.Chr23.746	Inhibitor of growth protein 3	Chromosome and associated proteins
CPED1	EVM.Model.Chr23.747	Cadherin-like and PC-esterase domain-containing protein 1	—
BUB1B-like isoform X1	EVM.Model.Chr24.309	Mitotic checkpoint serine/threonine-protein kinase BUB1 beta	—
SPINT1-like	EVM.Model.Chr24.310	Kunitz-type protease inhibitor 1	—
ARHGAP18	Evm.Model.Chr24.798	Rho GTPase-activating protein 18	—
XP_011490382.1	EVM.Model.Chr24.799	—	—
TMEM244	EVM.Model.Chr24.800	Transmembrane protein 244	—
Liprin-beta-2-like isoform X1	EVM.Model.Chr3.434	Liprin-beta-2	—
PPFIBP2	EVM.Model.Chr6.215	Liprin-beta-2	—
KAF6737022.1	EVM.Model.Chr6.216^a^	—	—
ARNTL protein 1	EVM.Model.Chr6.217	Aryl hydrocarbon receptor nuclear translocator-like protein 1	Dopaminergic synapse, Circadian rhythm, Transcription factors
GALNT18-like isoform X1	EVM.Model.Chr6.223	Polypeptide N-acetylgalactosaminyltransferase 18	—
SHANK3 isoform X1	EVM.Model.Chr6.915_EVM.Model.Chr6.916_EVM.Model.Chr6.917	SH3 and multiple ankyrin repeat domains protein 3	Glutamatergic synapse
ATF7IP2	EVM.Model.Chr8.986	Activating transcription factor 7-interacting protein 1	—
EMP2	EVM.Model.Chr8.987	Epithelial membrane protein 2	—

Note: —, indicates no relevant information.

^a^ Indicates that it cannot be annotated, we provide the access number with the highest score by blastx in the corresponding “gene name” column.

## Discussion

### Population genetic structure and genetic differentiation of *O. curvinotus*

We have assessed for the first time the population genetic structure and population history of the *O. curvinotus*, and found that the *O. curvinotus* can be divided into three subgroups, with the ST-medaka and JWM-medaka forming a subgroup located in the east, and the GQ-medaka and SY-medaka each forming a separate subgroup, which we suggest is the result of local subgroups adapting to their respective habitats.

**FIGURE 9:**
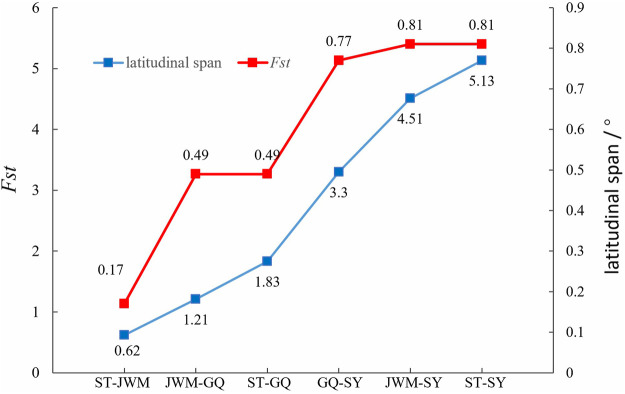
The relation between *F*_ST_ between geographic groups and their latitudinal span.

We found that the genetic divergence of SY-medaka from other geographic groups is large, which may result from different environmental differences, and that such environmental differences are likely to be latitude-related. It has been shown that *O. latipes* in high latitudes grew faster than those in low latitudes (Yamahira and Takeshi, 2008). Similar results were observed in *O. curvinotus*, and other physiological indicators such as heart rate were also found to differ between geographic groups at different latitudes (Wang, 2020) (Supplementary Figures S8, unpublished). We also found that the genetic differentiation of *F*_ST_ between geographic groups showed some correlation with their latitudinal span (Figure 9), suggesting a genetic basis for the physiological differences between geographic groups at different latitudes. Interestingly, the rate of LD decay also shows some correlation with latitude. In the *O. curvinotus*, LD decays particularly rapidly, reaching a steady state at r^2^ at about 20 kb, which is probably due to the very short generation interval (about 3–6 months). The same situation exists in *O. latipes* (Spivakov et al., 2014; Katsumura et al., 2019). This indicates that the fishes of *Oryzias* have some commonalities, which do not change with environmental factors.

In addition, SY-medaka is a special and interesting population. SY-medaka decayed more rapidly amongst the four geographic groups, significantly more than in the other groups, which we attribute to the effects of long-term environmental selection pressures on SY-medaka.

### Population history and selective pressures of *O. curvinotus*

Previous studies have suggested that the latipes group (comprising *O. latipes*, *O. curvinotus* and *O. luzonensis*) arose in 27.3 Mya and that *O. latipes* formed in 9.2 Mya, on the basis of which the species formation and population divergence of the *O. curvinotus* would have occurred within 9.2 million years (Rabosky et al., 2018). Geological evidence suggests that Hainan Island, where SY-medaka is located, was initially closely linked to Eurasia. However, due to the influence of the Indian plate on Eurasia, Hainan Island separated and drifted to its present location. This process occurred at about 65 Mya-24 Mya (Guanghe, 2018), suggesting that Hainan Island was completely detached from the mainland by the time the *O. curvinotus* formed. The separation of Hainan Island indirectly led to the creation of the Qiongzhou Strait, a necessary precondition for the geographic isolation of SY-medaka from other groups, however this geographic isolation was unstable and subject to sea-level elevation.

Some paleoceanographic data demonstrate that the sea level has been changing continuously throughout history, with the last drastic change occurring about 20,000 years ago (Ganopolski et al., 2010; Hertzberg, 2015; Peter et al., 2009). The global sea level continued to decline due to the advent of the Last Glacial Period, the coastline of the Eurasian continent moved southward, and the bottom of the Qiongzhou Strait emerged. The decline of the sea level not only completely separated the groups of *O. curvinotus*, but also turned the original water surface into land. Only the low-lying and river channels are still covered with water, which obviously restricts the living space of *O. curvinotus*. This is consistent with the historical changes that occurred in the populations we analyzed. Since about 100,000 years ago, each group has undergone population decline. During the Last Glacial Period, there was a decline in the population number of the four populations, but the population size of the JWM-medaka and GQ-medaka in Shantou recovered or even exceeded their prior numbers. However, the populations of SY-medaka and GQ-medaka, despite historically experiencing a short population increase, have been decreasing ever since, and both populations seemed to be in a bottleneck period. However, SY-medaka declined the most, to a much greater extent than GQ-medaka, and there was no trend of population recovery in many estimation results. Not only that, but we also found that the population size change of the Southern population of *O. latipes* during the Last Glacial Period was perfectly in line with our speculation that the population of Japanese Southern medaka started to decline at the onset of the Last Glacial Period and began to expand almost simultaneously with the end of the ice age (Spivakov et al., 2014), thus we suggest that the ice age affected the sea level and thus the population size of all *Oryzias* species. Unfortunately, however, for our data, PSMC can only estimate up to the last 10,000 years, and it remains to be further explored whether GQ-medaka and SY-medaka will show expansion after the bottleneck, like the population groups or *O. latipes*.

At the same time, we found that the trends in population change of ST-medaka and JWM-medaka were almost the same, while the change trends of GQ-medaka and SY-medaka were almost the same, although their population levels differed greatly. The ML tree constructed based on mitochondria also reflects that GQ-medaka and SY-medaka are closely related, indicating that they have a common ancestor. However, the NJ tree based on nuclear SNPs revealed that GQ-medaka and SY-medaka are far from each other. Therefore, we believe that SY-medaka was subjected to strong selection that resulted in GQ-medaka and SY-medaka having a common ancestor while being genetically distant.

### Potential environmental factors acting as selection pressures on SY-medaka

In terms of possible environmental factors acting as selective pressures on SY-medaka, we only considered temperature and ultraviolet light. Because *O. curvinotus* is mainly distributed along the coast of southern China, the climatic conditions of the four sampling points are almost the same, except that the light and temperature may have been different. Using climate data collected from the Weather Spark website from 1 January 1980, to 31 December 2016, we compared the climate and weather changes of Shantou, Guangzhou (representing Jiuwangmiao), Gaoqiao, and Sanya within 1 year (“Weather Spark, 2018.). While there were no regional differences in the average daily short-wave solar energy, differences were observed for water temperature. Except for the July–September period, the monthly average water temperature in Sanya was higher than in the other three geographical locations (Supplementary Figures S1), especially from January to February in the cold season, suggesting that the water temperature difference caused by different latitudes may be an important factor by which environmental selection for *O. curvinotus* occurs.

### Selected genes of SY-medaka

39 selected genes of SY-medaka participate in multiple biological processes, which may indicate the potential processes through which *O. curvinotus* have adapted to the new environment of Sanya. Among them, the circadian rhythm pathway may provide possible clues for environmental stress factors. In mammals, heat stress can regulate circadian rhythm through a series of physiological reactions (Hertzberg, 2015), to make the body adapt to this thermal environment. In addition, UV stress research has found that clock genes can regulate the circadian clock to mediate sequential and hierarchical interactions between the heat shock response and tumor inhibition mechanisms, thereby protecting cells from UV stress (Kawamura et al., 2018). Of course, further experiments are needed to verify whether the *O. curvinotus* also responds to environmental changes through these mechanisms. Other genes may also be related to some potential mechanisms. For example, we found that many genes are closely related to cell growth and death. The expression of *stk3*, *ing3*, *trit1,* and *laptm4b* may inhibit cell growth and induce apoptosis (Golovko et al., 2000; Nagashima et al., 2003; Yarham et al., 2014; Blom et al., 2015; Wang et al., 2020), and *cep192* and *bub1b* may play an important role in mitosis (Chan et al., 1999; Zhu et al., 2008; Joukov et al., 2014). Further research is required to better understand their roles and possible mechanisms.

Although many genes lack annotation information and related pathway research, we believe that positive selection of these genes is very important for the adaptation of SY-medaka to the new environment. Furthermore, these genes provide us with an important research basis and research direction. We also note that the genome mapping rate of SY-medaka is about 1% lower than that of other populations, suggesting that more genes are selected against and not found in SY-medaka. It is difficult to identify these genes through the GQ-medaka genome. Therefore, we have started an SY-medaka genome project at the time of writing this paper, which is expected to further explore the environmental adaptation process of SY-medaka.

## Conclusion

In this study, we performed WGS on 56 *O. curvinotus* individuals from four geographic locations, identified 32,826,105 SNPs. The population genetic structure analysis showed that the *O. curvinotus* can be divided into three subgroups. Among them, ST-medaka and JWM-medaka were the most closely related, while the SY-medaka was the most divergent from the other populations. The results of the population history of the *O. curvinotus* suggest that the SY-medaka has been in a bottleneck period recently and a variety of evidence shows that them were subjected to continuous strong selection pressure. By selective screening, we also identified some potential gene regions that were subject to significant positive selection in the SY-medaka. Candidate genes located in these regions may play important roles in biological processes such as circadian rhythm and cell cycle regulation, which may suggest potential adaptive processes that occurred in the new Sanya environment. Our study provides a genetic basis for the study of environmental adaptation of medaka.

## Data Availability

The resequencing raw data of all samples in this study have been uploaded to the NCBI Sequence Reading Archive (SRA) with storage number of PRJNA792300. Genome sequences have been submitted to GenBank (PRJNA821560). SNPs data for all samples have been submitted to Dryad, https://doi.org/10.5061/dryad.r2280gbf7. The complete mitochondrial sequence was submitted in fasta format in the Supplementary Material.
