# Supplementary figures and images for: Population genetic characteristics of Hainan medaka with whole-genome resequencing
Source: Front Genet. 2022 Oct 12;13:946006. doi: 10.3389/fgene.2022.946006 (PMC9597887; doi:10.3389/fgene.2022.946006)

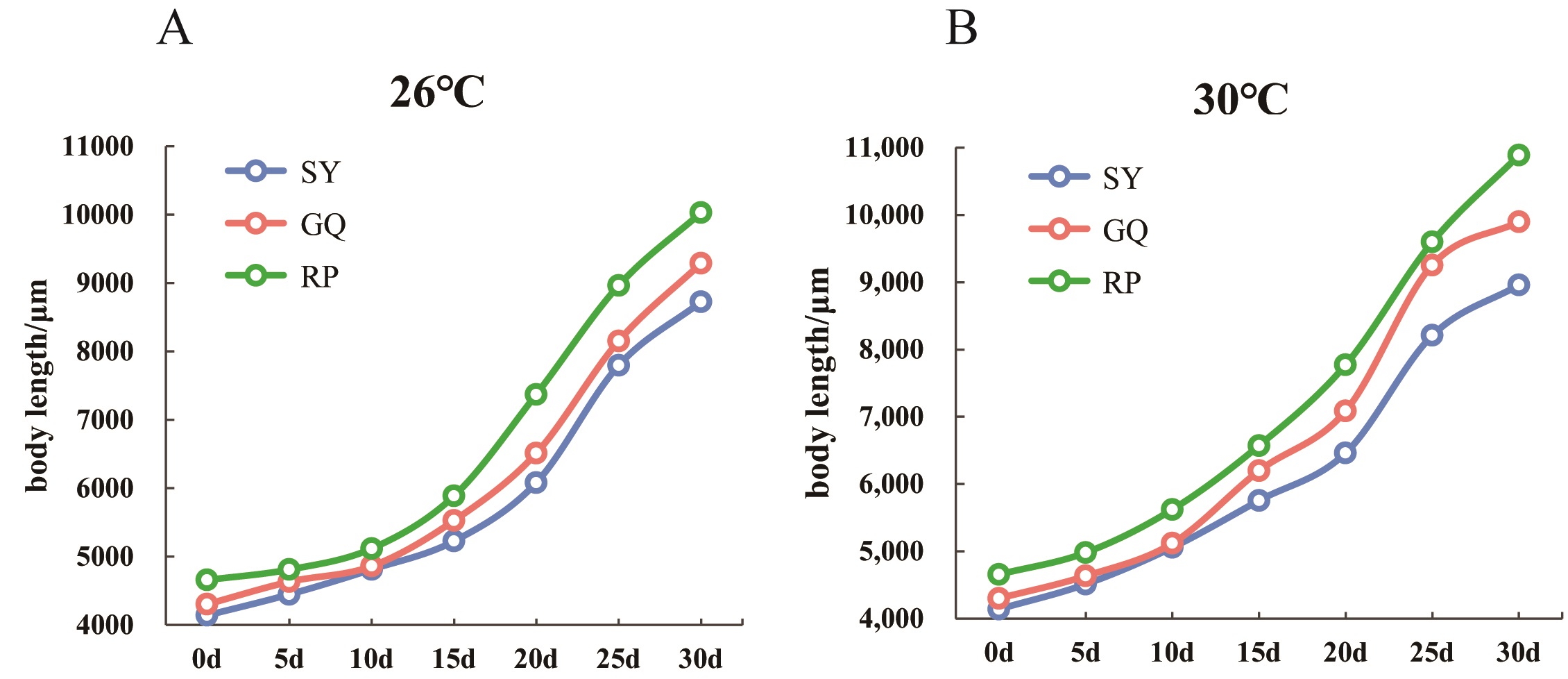

Supplement: Supplementary file 1 [file Image3.JPEG]

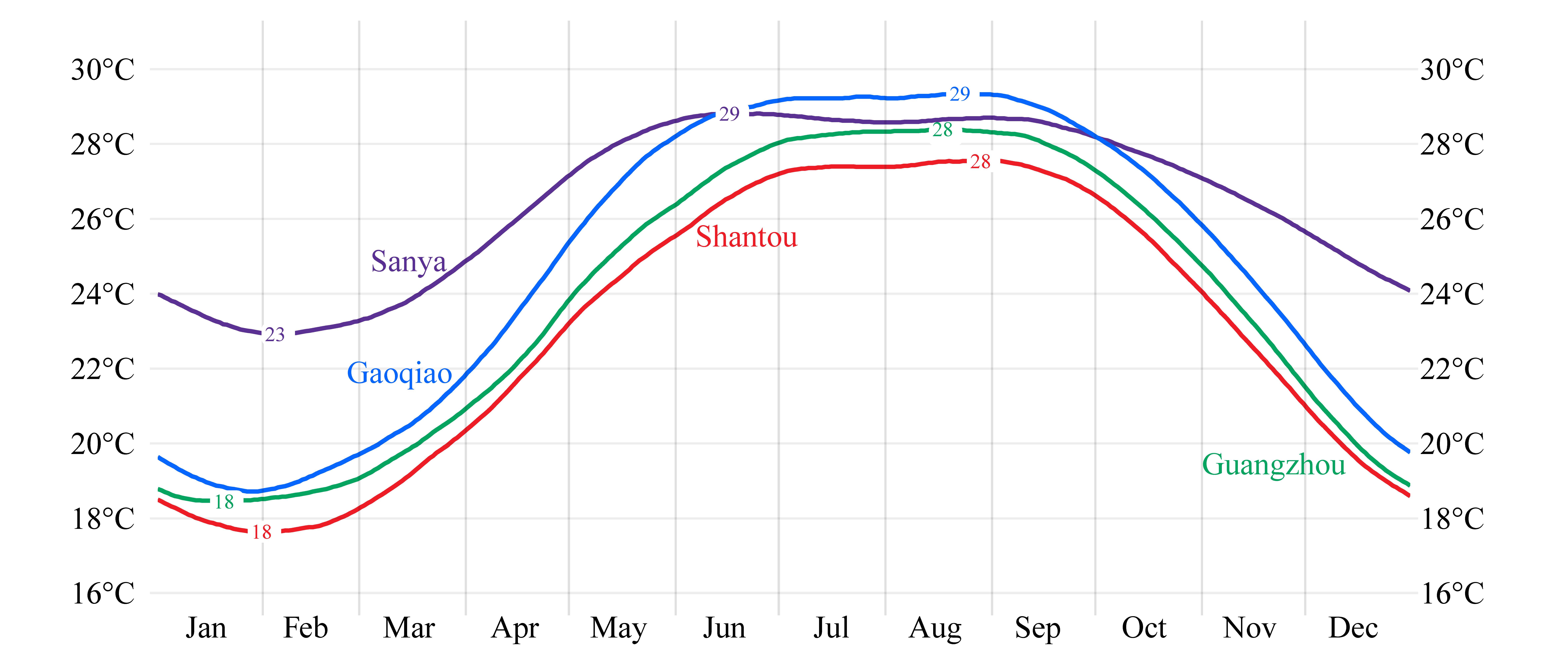

Supplement: Supplementary file 2 [file Image1.JPEG]

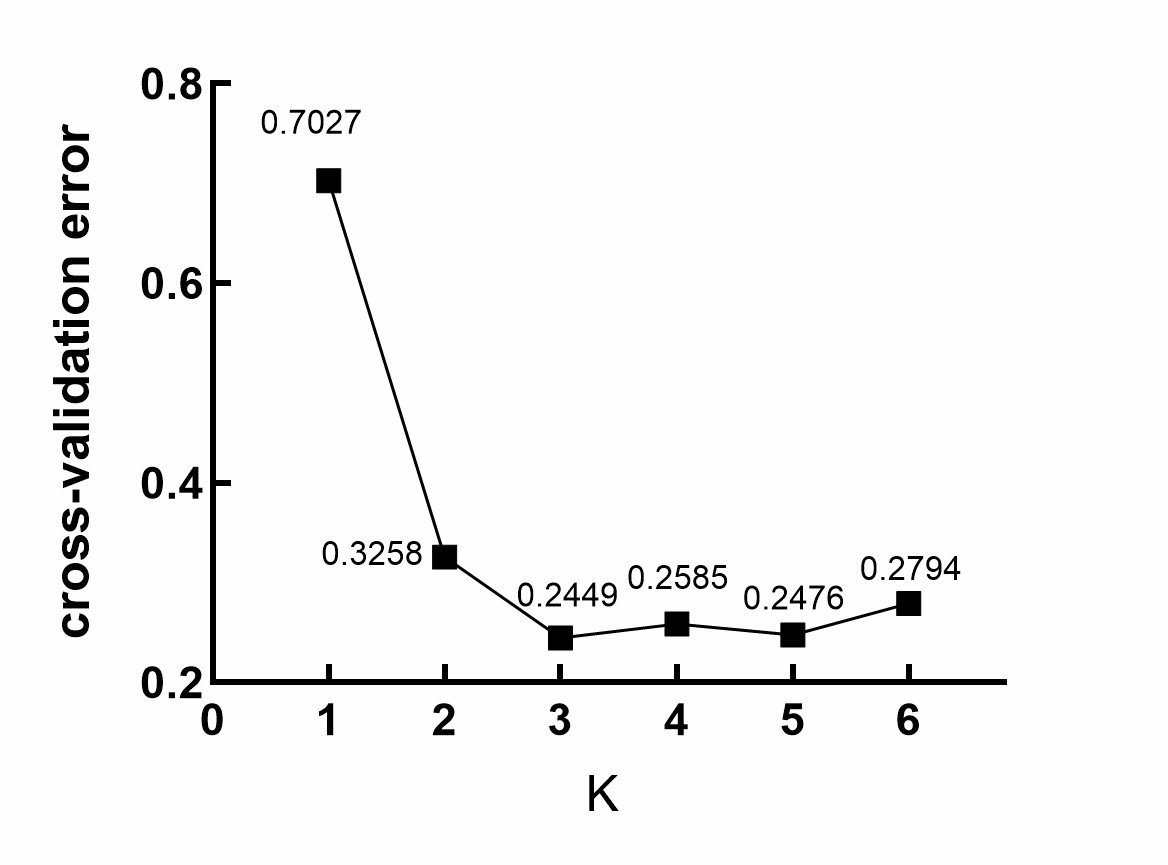

Supplement: Supplementary file 3 [file Image4.JPEG]

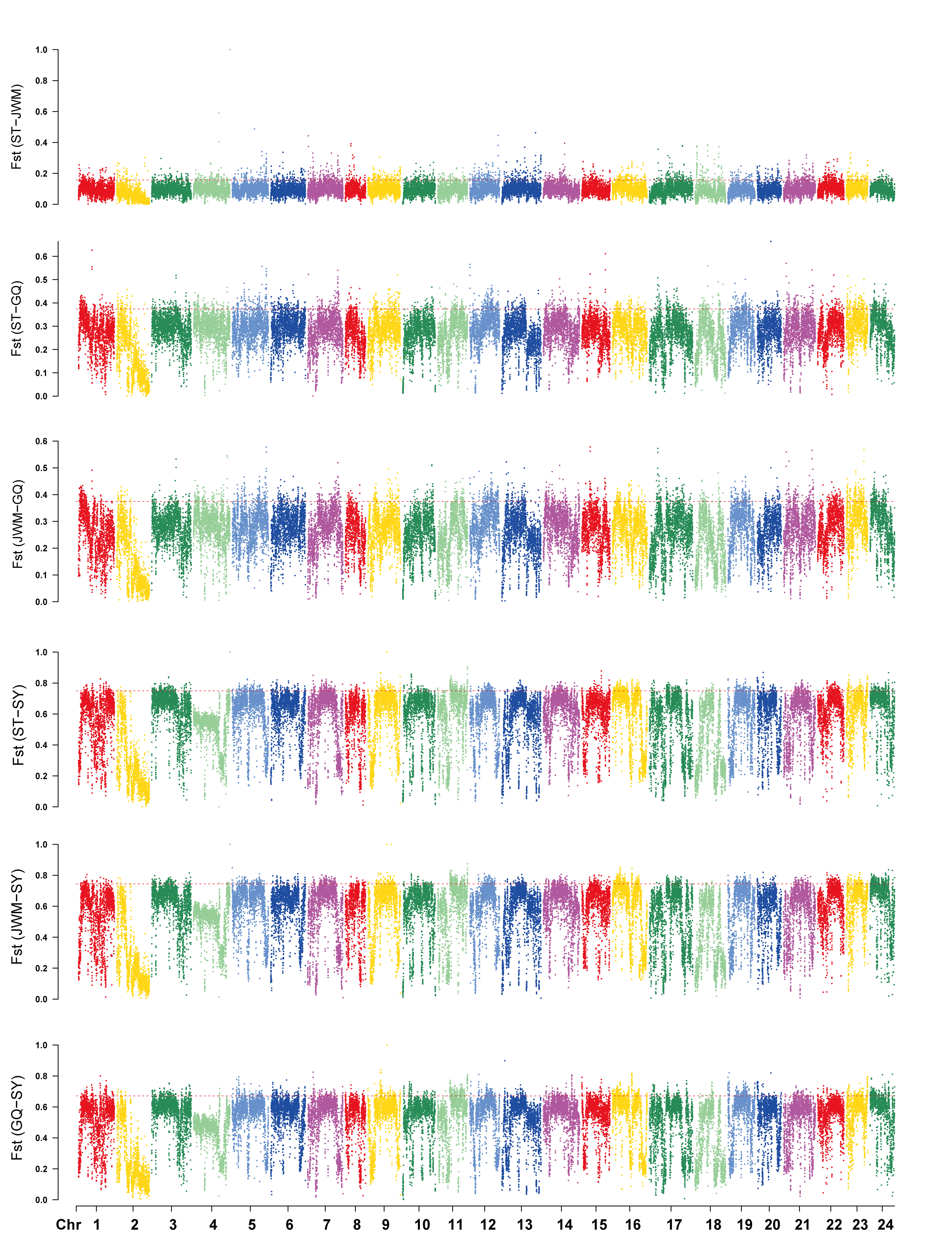

Supplement: Supplementary file 4 [file Image5.PNG]

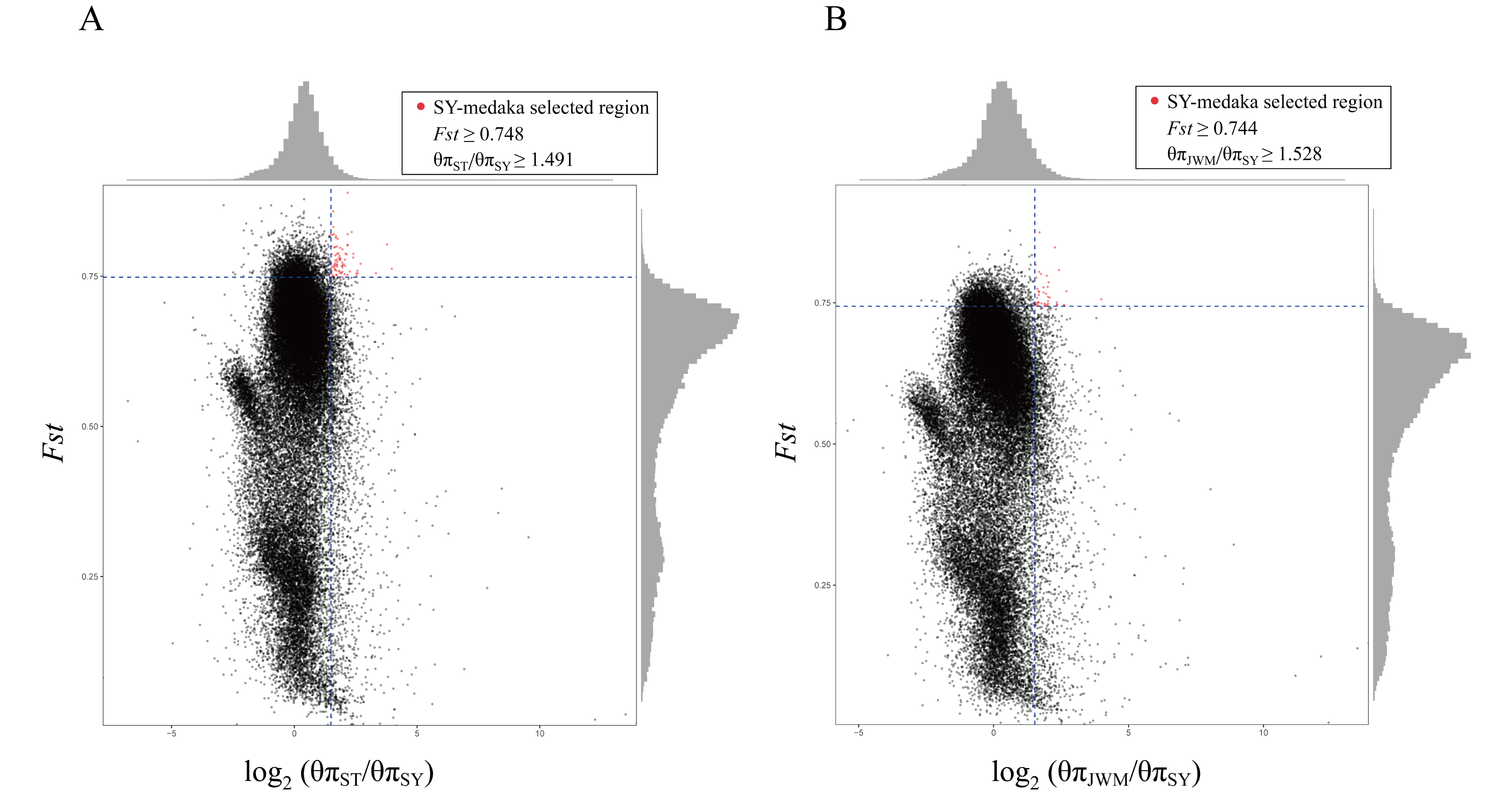

Supplement: Supplementary file 7 [file Image7.PNG]

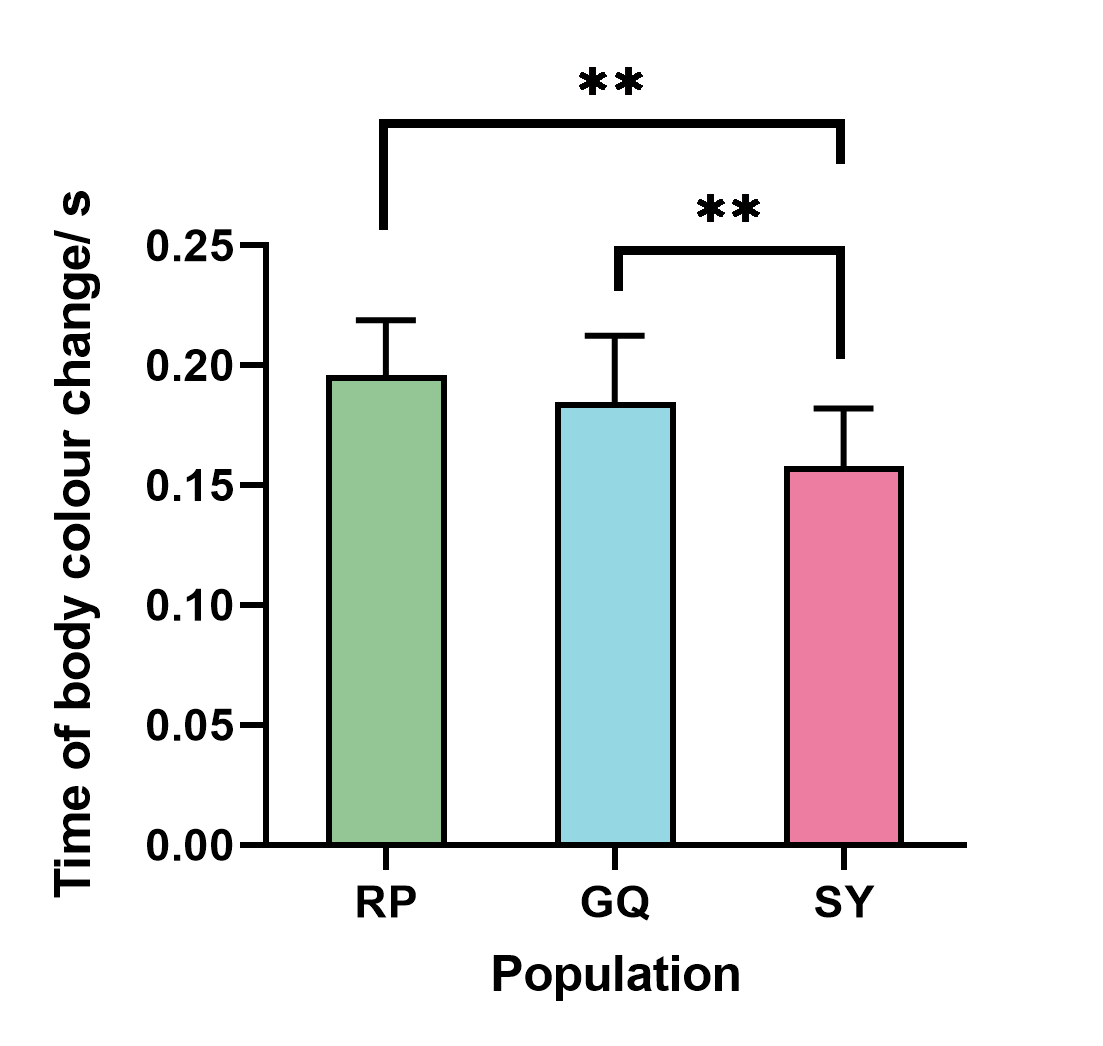

Supplement: Supplementary file 8 [file Image2.PNG]

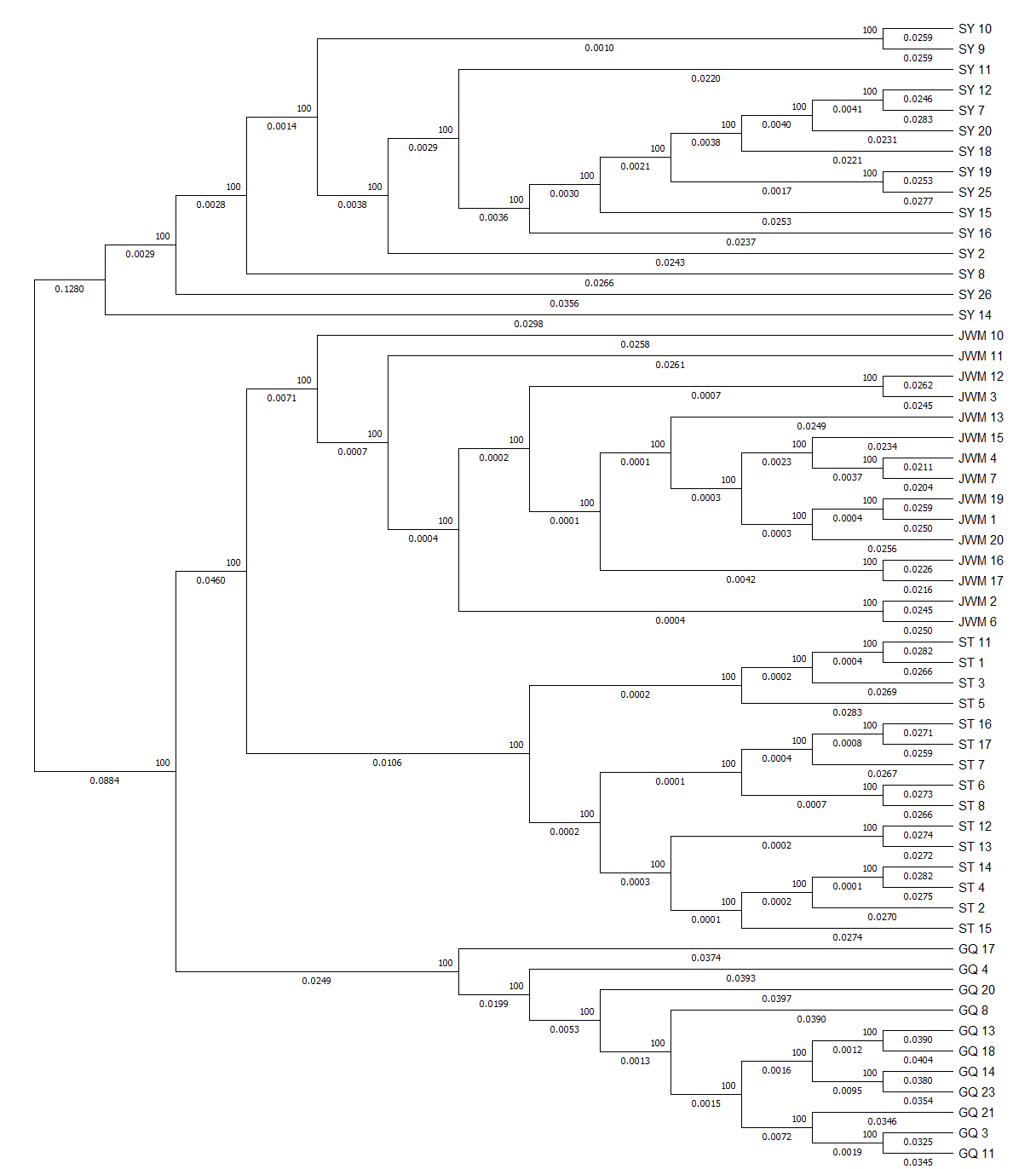

Supplement: Supplementary file 9 [file Image9.PNG]

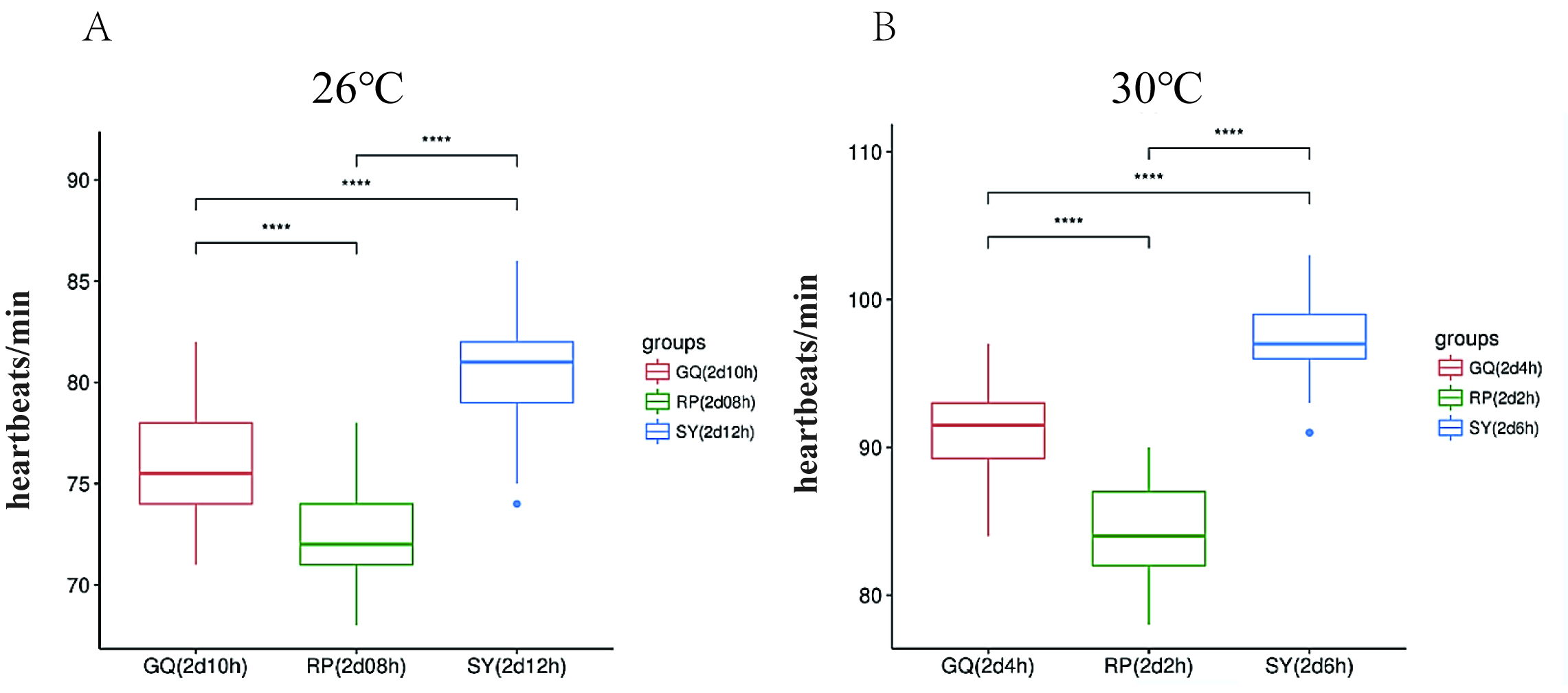

Supplement: Supplementary file 10 [file Image8.JPEG]

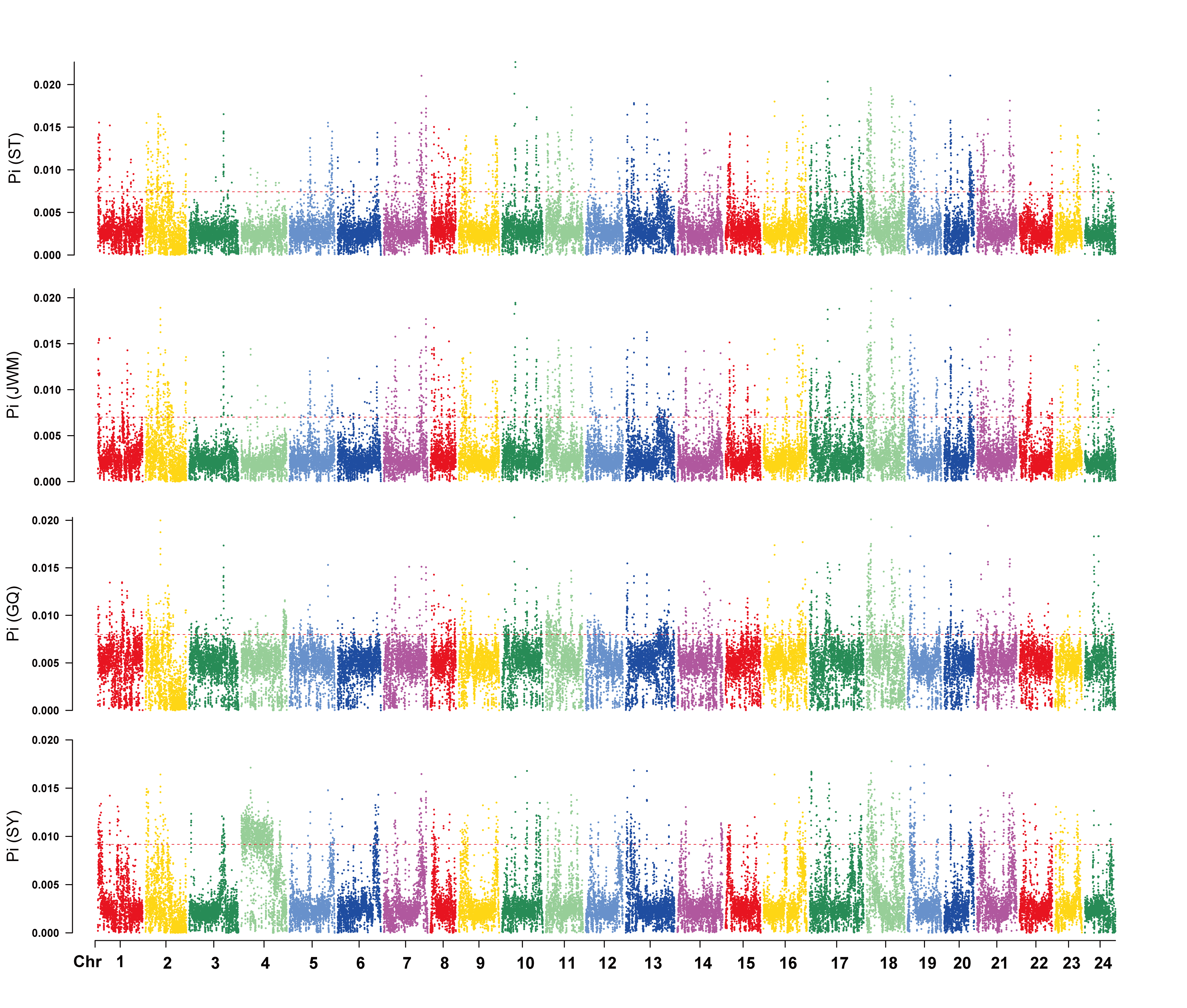

Supplement: Supplementary file 11 [file Image6.PNG]
