# Supplementary material for: Population genetic characteristics of Hainan medaka with whole-genome resequencing
Source: Front Genet. 2022 Oct 12;13:946006. doi: 10.3389/fgene.2022.946006 (PMC9597887; doi:10.3389/fgene.2022.946006)
Supplement: Supplementary file 5 [file Table1.DOC]

Fig .1 *Oryzias curvinotus*. (A) Male (upper) and female (lower) *Oryzias curvinotus* form Sanya. (B) Natural habitat of Hainan medaka.

Fig. 2 Species distribution of *Oryzias curvinotus* . Species distribution map for *Oryzias curvinotus* and some new investigation sites; 1, Raoping; 2, Dahao; 3, Niutianyang(ST); 4, Conghua; 5, Nansha(JWM); 6, Zhonggui; 7, Yamchow; 8, Gaoqiao(GQ); 9, Huguang; 10, Donghai dao; 11, Fucheng; 12, Leizhou; 13, Lingao County; 14, Chengmai; 15, Dongzhaigang ; 16, Wenchang; 17, Sanya(SY); The location IDs are in common with Table 4; Four collection sites of *O. curvinotus* for WGS are marked with red triangles. The red arrow refers to the Qiongzhou Strait.

Fig. 3 Population genetics. (A) Genetic structure of the *O. curvinotus* as inferred by Admixture analysis. The number of populations (K) from 1 to 6 is shown. Each color represents a different hypothetical ancestor. (B) Unrooted tree generated by the neighbor-joining method with 1000 bootstrap. (C-D) The principal component analysis (PCA) of all individuals.

Fig. 4 Based on the ML tree of mitochondrial whole sequences, some other fish of the *Oryizas* genus were used as outgroups. The bootstrap value(above branch) and branch lengths(below branch)are shown here.

Fig. 5 The results of the analysis of the demographic history of the *O.curvintous* are presented. Multiple values ranging from 0.25 × 10^-8^ to 2.5 × 10^-8^ were used for mutation rates(u). The generation intervals(g) were 0.25, 0.33, 0.5, 0.66, and 1 year based on the sexual maturity time of the medaka and field surveys.

Fig. 6 (A) Linkage disequilibrium patterns of 4 populations. (B) *F*_ST_ values among populations and θπ in each population.

Fig. 7 (A)The distribution of the θπ ratios(θπGQ-medaka/θπSY-medaka) and *F*_ST_ values(GQ-SY), calculated in 20-kb windows sliding in 10-kb step. Data points on the right of the vertical dashed line (corresponding to the 5% left tail of the empirical θπ ratio distribution), and above the horizontal dashed line (5% right tail of the empirical *F*_ST_ distribution) were identified as selected regions for SY-medaka(red points). (B) Examples of genes with strong selective sweep signals in GQ-medaka and SY-medaka. *F*_ST_ and θπ values are plotted using a 10-kb sliding window. Shaded genomic regions were the regions with strong selective signals for SY-medaka.

Fig. 8 Venn diagram showing the intersection of the number of genes subject to selection in SY-medaka relative to other populations.

Fig. 9 The relation between *F*_ST_ between geographic groups and their latitudinal span.

Fig. S1 Monthly variation in average water temperature for 4 geographical locations. Data were collected from Weather Spark website from January 1, 1980 to December 31, 2016.

Fig. S2 Response time of body colour to background colour in the O. curvinotus. GQ, GQ-medaka;RP, Raoping population(RP-medaka); SY, SY-medaka.

Fig. S3 Growth rates of three populations of *O. curvinotus* at 26°C and 30°C. GQ, GQ-medaka;RP, Raoping population(RP-medaka); SY, SY-medaka.

Fig. S4 Cross validation errors for each K value. Statistical support for the different number of clusters was evaluated based on fivefold cross-validation implemented in Admixture.

Fig. S5 Genome-wide scans and *F*_ST_ value calculation among populations in 40 kb windows in 20 kb steps.

Fig. S6 Genome-wide scans and θπ value calculation on each populations in 40 kb windows.

Fig. S7 (A)The distribution of the θπ ratios(θπST-medaka/θπSY-medaka) and *F*_ST_ values(ST-SY), calculated in 20-kb windows sliding in 10-kb step. Data points on the right of the vertical dashed line (corresponding to the 5% left tail of the empirical θπ ratio distribution), and above the horizontal dashed line (5% right tail of the empirical FST distribution) were identified as selected regions for SY-medaka(red points). (B)The distribution of the θπ ratios(θπJWM-medaka/θπSY-medaka) and *F*_ST_ values(JWM-SY), calculated in 20-kb windows sliding in 10-kb step. Data points on the right of the vertical dashed line (corresponding to the 5% left tail of the empirical θπ ratio distribution), and above the horizontal dashed line (5% right tail of the empirical *F*_ST_ distribution) were identified as selected regions for SY-medaka(red points).

Fig. S8 Heartbeat rates of different populations of the bow-backed medaka at 26°C and 30°C after 2 days of incubation.

Fig. S9 Unrooted tree generated by the neighbor-joining method with 1000 bootstrap. The bootstrap value(above branch) and branch lengths(below branch) are shown here.
