# Supplementary material for: Population genetic characteristics of Hainan medaka with whole-genome resequencing
Source: Front Genet. 2022 Oct 12;13:946006. doi: 10.3389/fgene.2022.946006 (PMC9597887; doi:10.3389/fgene.2022.946006)
Supplement: Supplementary file 6 [file Table3.DOCX]

>GQ 11

GCTAGTGTAGCTTAACTAAAGCATAACACTGAAGATGTTAAGACAAACCTTAGACTGGTTTCACGAGCACAAAAGTTTGGTCCTGACTTTACTATCAACTTTAGCTAAACTTACACATGCAAGTATCCGCAATCCCGTGAGAATGCCCTACAGTTTCCTTAAAGGAAACAAGGAGCTGGTATCAGGCTCAATTACTCCCGCCCATGACACCTTGCTTAGCCACACCCCCAAGGGAACTCAGCAGTGATAGACATTAAGCAATAAGTGAAAACTTGACTTAATTAAAGCTAAGAGAACCGGTTAAACTCGTGCCAGCCACCGCGGTTATACGAGCGGTTCGAGCTGATAGATTACGGCGTAAAGCGTGGTTAATAAGAATGAAACTAAAGTCGAATGTTTTCAAAGCTGTTATACGCACTCGAAAATTAGAAGCTCAGAAACGAAAGTGACTTTAACCCTATGAACCCACGAAAACTATGAAACAAACTGGGATTAGATACCCCACTATGCATAGCTGTAAACTTTGATGAGCTATTACATTATCATCCGCCTGGGTACTACGAGCATCAGCTTAAAACCCAAAGGACTTGGCGGTGCTTTAGACCCACCTAGAGGAGCCTGTTCTAGAACCGATAACCCCCGTTAAACCTCACCCTCTCTTGTTTTTCCCGCCTATATACCGCCGTCGTCAGCTTACCCTGTGAAGGTCTAATAGTAAGCACAACCAGTTATACTCAAAACGTCAGGTCGAGGTGTAGCATATGAGAGGGGAAGAAATGGGCTACATTCCTTGTTTCAAGGAAAACGGATAACATAATGAAAGGTACGTTAGAAGGAGGATTTAGCAGTAAGCAGCAAATAGAGTGTTCTGCTGAAACTGGCCCTGAAGCGCGCACACACCGCCCGTCACTCTCCCCAACTCCGAGTTAAAAACATATATAAACCTTTGAAGGAACAAAGGGGAGGCAAGTCGTAACATGGTAAGTGTACCGGAAGGTGCACTTGGATAAATCAGAGTATAGCTAAGAAAGAAAAGCATCTCCCTTACACCGAGAAGTCATCCGTGCAAATCGGATTACCCTGACTCTAACAAGCTAGCCCAAAACCTTAACTTAAAAATCAAATATTTCTAGTAATTAATAAACCAAACACATTAAATAAAACATTTTTCCCCCTGAGTATGGGAGACAGAAAAGGATAAAGGAGCTATAGACAAAGTACCGCAAGGGAAAGCTGAAAGAGAAATGAAACAAACCAGTAAAGAAAAACAAAGCAGAGATTAACCCTTGTACCTTTTGCATCATGAATTAGCCAGTTTAATCAAGCAAAGAGCACTGTAGTTTGAAACCCCGAAACTTAGTGAGCTACTTCAAGACAGCCTATGAAATAGGGCAAACCCGTCTCTGTGGCAAAAGAGTGGGAAGATCTTCAAGTAGAGGTGACAGACCTATCGAACTAAGTTATAGCTGGTTGCTCGTGAAATGAATAGAAGTTCAGCCTTTTGCTTTCTAAATTTCGATTTAGCATCACTTAGCCTAAATGACTAGAAAACAAAAGAGTTAGTCAAAGAGGGTACAGCCTGTTTGATAAAAGATACAACTTTACTAGGAGGATAAGAATCATAATTTTAAAGGTTTAATGCCCAGGTGGGCCTAAAAGCAGCCACCCTGATCAATAGCGTTAAAGCTTAAGCATAAAACACACCTACAATTCTGATAAATCAGTTTTAATCCCCTAAGGTTAACGAGCTATTTCATACCTTATGAAAGAAATTATGCTAGTATGAGTAATAAGAAGCTACGAACTTCTCCCTGCACACGTGTAAATCGGAACGGACAAACCACCGAATCTTAACGGCCCCAGTCAAAGAGGGGATGTCGGATAAAAAAAAGAACAAGAAATTCCCGATAAAACCACCGTTAACCCCACACCGGAGTGCTCCCTGGGAAAGACAAAAAGGGACAGAAGGAACTCGGCAAATATGCTCAAGCCTCGCCTGTTTACCAAAAACATCGCCTCTTGTAAAAGTTAAATAAGAGGTACCGCCTGCCCTGTGACTAGTAGTTTAACGGCCGCGGTATTTTGACCGTGCAAAGGTAGCGCAATCACTTGCCTTTTAAATGAAGGCCTGTATGAATGGCACGACGAGGGCTTAACTGTCTCCTCTCCCTAGTCAATGAAATTGATCCCCCCGTGCAGAAGCGGGGATAGTAACATAAGACGAGAAGACCCTGTGGAGCTTTAGACTATAAGCAGACCATGTCAAGAATAACAAACAAGTAAATTAAACAAATTGGCCCCTGCTTCCCTGTCTTTGGTTGGGGCGACCGCGGGATAATAAAAAACTCCCACGAGGATTGAGAACCCTTATCTTATAACCAAGAGCTTCTCCTCTAAGTAACAGAACATCTGACCTTAATGATCCGGCCTGGCCGATCAACGGACCAAGTTACCCCAGGGATAACAGCGCAATCCTCTTTTAGAGTCCATATCGACAAGAGGGTTTACGACCTCGATGTTGGATCAGGACATCCTAATGGTGCAGCCGCTATTAAGGGTTTGTTTGTTCAACAATTAAAGTCCTACGTGATCTGAGTTCAGACCGGAGTAATCCAGGTCAGTTTCTATCTATGACGTACTCTCTTCTAGTACGAAAGGACCGAAGAAAGAAGGCCCATGAAAAATTATGCCTTAGTCTCACCTTATGAAGAAAACTAAATAAGACAAGAGGTTACACCCTTTGGTCATAGAAAATGACATGTTAAGGTGGCAGAGCCCGGATATTGCAAAAGACCTAAGCCCTTTCCACAGAGGTTCAATTCCTCTCCTTAACTATGTTCTCAACAATATTAAGCTTCATTATTAATCCCCTGATTGTTATGATTTTTGTTTTGTTGGCAGTAGCCCTCTTGACCTTAGTAGAGCGTAAAGTGCTAAGCTACATGCAACTTCGTAAAGGCCCAAATGTTGTTGGCCCTTACGGCCTTTTGCAACCCTTCGCTGATGGCTTAAAACTTTTCATGAAAGAGCCCGTCCGGCCCTCCACCTCCTCGCCCGCCTTGTTCTTAATTACCCCTATTATAGCCCTTACCTTAGCCCTAACCCTCTGAGCCCCCCTTCCTATGCCTTTTCCCGTCACCGACCTAAACTTAGGCATTTTATTTATTTTAGCACTATCGAGCCTGGCAGTATATTCTATTCTTGGCTCCGGATGAGCCTCCAACTCTAAATATGCATTAATTGGTGCTCTTCGAGCGGTCGCCCAAACCATCTCTTATGAAGTAAGCTTAGGCCTTATTCTTCTTAACACAATTGTCTTTACGGGGGGTTTTACTCTTCAAACCTTCAGCACCGCACAAGAAGCCACCTGATTACTTCTACCAGCATGACCACTAGCAGCCATGTGATATATCTCCACACTCGCGGAAACTAACCGGGCCCCTTTCGACCTAACTGAAGGAGAGTCCGAACTAGTGTCTGGCTTCAACGTAGAGTATGCCGGCGGACCTTTTGCCCTTTTTTTTCTGGCAGAATACGGTAACATTTTACTTATAAATACCCTCTCAGCAGTTCTATTTCTAGGCTCTTCAACCTACCACAGCTTTCCAGAACTAACCGCGACCTTATTAATGCTTAAAGCCACCCTCCTTTCAGTCGTATTTTTATGAGTGCGAGCATCTTACCCTCGGTTCCGATACGACCAACTAATGCATTTAATTTGAAAAAACTTTTTACCTCTGACCCTAGCGCTAGTTATTTGACACCTTTCTCTTCCGATCACGTTGAGCGGCCTCCCTCCTCAACTTTAACTCAGGAAATGTGCCTGAAAAAGGGTCACTTTGATAGGGTGAATAATGAGGGTTAAAGCCCCTCCATCTCCTTAGAAAGAAGGGGTTTGAACCCTACCTGAAGAGATCAAAACTCTTAGTGCTTCCACTACACCACTTCCTAGTAAAGTCAGCTAATAAAAGCTTTTGGGCCCATACCCCAAATATGTTGGTTAAAATCCTTCCTTTGCTAATGAATCCTTACGTCCTTTCAATTCTACTTATGGGTTTAGGCCTCGGCACTACAGTCACATTCGCTAGCTCACACTGACTCTTAGCATGAATAGGCCTTGAAATAAATACCCTCGCCATTTTGCCATTAATAGCACAACATCACCACCCCCGAGCCGTTGAAGCCACCACCAAGTATTTTTTAATTCAATCGGCAGCCGCAGCAACCATCTTATTTGCCAGCTCAACTAACGCCTGACTTTCGGGCCAGTGGGACATCATAAGTATCAATCACCCCCTTCCAACCGTCATAATTACAATCGCTCTGTCCTTAAAACTAGGCTTGGCCCCTCTTCACGCCTGACTTCCCGAAGTTATTCAAGGACTAGACTTGACCACGGGCTTAATCCTCTCCACATGACAAAAACTCGCACCCTTTGCCCTTCTCGTTCAAATCTTCCCCGACACCCCCCTTCTCATCACTTCTCTAGGGCTTCTTTCAATATTAATTGGGGGGTGAGGAGGTTTAAACCACACACAACTCCGCAAAGTGCTCGCATATTCTTCAATCGCCCACTTAGGCTGAATAATAGTAATTATGCAATTCTCCACCCCCCTTACAATTCTTGCTTTATCAACATACATTGTTATAACATCATCTACTTTCCTAATCTTTAAACTCCTTAAGTCCACAGATATGAACAGCCTGGCAACATCTTGAGCTAAAACCCCCTCCATTACAGCCCTAGCACCTTTAGTGCTATTATCCTTAGGCGGACTCCCTCCCCTCTCGGGCTTTATGCCAAAATGATTAATTATTCAAGAATTAACTAAGCAAGATCTAGCCCTAGTTGCAACCTTGGCCGCCCTCTCTGCGCTACTCAGCCTTTTCTTCTACCTACGCATTTGTTACTCCCTCACATTTACCTCCTCTCCTAATAATCTCATGGGAACGCCCCCCTGACGACTAGTAACAAAGCAAGTATCACTTCCCCTAGCTATAACAACCTCCCTCTCTATTCTTCTACTCCCGGTTACCCCTGCAATCTTATCAGTGGTTCTCCCTTTGTAAAGAGGCTTAGGATAGTATTAAGACCAAGGGCCTTCAAAGCCCTAAGCGGGAGTGAAAGCCCCCCAGCCTCTGTAAGACCTACGGGACACTAACCCACATCTTCTGTATGCAAAACAGACACTTTAATTAAGCTAAAGCCTTCCTAGGTGGGTAGGCCTCGATCCTACAATCTCTTAGTTAACAGCTAAGCGCCTAAACCAGCGGGCATCCATCTACCTTTCCCCCGCCTTGCCGAAAAAAAAAGGCGGGGGAAAGCCCCGGCAGGGTATTAGCCTGCCACTTAAGATTTGCAATCTAATGTGTTAACACCTCGGGGCTGGTAAGAAGAGGACTTTAACCTCTGTCCATGGAGCTACAATCCACCGCTAAACGCTCAGCCACCTTACCTGTGGCAATCACACGTTGATTTTTCTCGACTAATCACAAAGACATCGGCACCCTGTATCTAATCTTTGGTGCCTGGGCGGGAATGGTAGGGACGGCCTTAAGTCTACTCATTCGGGCAGAACTAAGTCAACCAGGCTCCCTATTAGGAGACGACCAGATCTATAACGTAATTGTAACTGCACATGCTTTCGTAATAATTTTCTTTATAGTAATGCCAATCATAATTGGGGGGTTTGGCAACTGATTAATTCCTTTAATGATTGGAGCCCCCGACATGGCCTTCCCACGGATAAATAATATAAGCTTTTGACTCCTGCCCCCTTCTTTCCTTCTATTATTGGCCTCATCTGGTGTAGAAGCTGGTGCCGGAACAGGATGAACCGTATATCCCCCCTTGTCAGGTAATTTGGCACACGCAGGGGCCTCCGTAGATTTAACCATTTTCTCTCTTCACCTGGCCGGAATTTCTTCTATCCTAGGGGCCATTAATTTCATCACAACTATTATTAATATAAAACCTCCAGCCATTTCCCAATATCAAACCCCTTTATTTGTGTGAGCTGTACTAATTACCGCAGTATTACTTCTACTCTCTCTTCCTGTTCTAGCTGCAGGTATCACCATGCTTCTCACAGATCGGAACCTAAATACAACATTTTTCGACCCCGCAGGAGGGGGGGACCCCATTCTTTATCAACATTTATTCTGATTCTTTGGGCATCCTGAAGTTTACATTCTAATTTTGCCCGGCTTCGGAATGATTTCTCACATTGTAGCATATTACTCAGGCAAAAAAGAGCCGTTTGGTTACATGGGAATAGTATGAGCTATAATAGCAATTGGCTTGCTGGGCTTTATCGTATGGGCCCATCATATGTTCACTGTGGGGATGGACGTGGACACTCGAGCTTATTTTACTTCCGCCACTATGATTATCGCAATTCCCACAGGAGTCAAAGTGTTTAGTTGACTAGCTACCTTGCATGGGGGCTCAATCAAATGAGAAACCCCTCTGTTATGAGCTCTGGGCTTTATCTTCTTATTTACTGTCGGAGGTTTAACAGGAATTGTTTTAGCCAACTCATCTCTGGACATTATACTTCATGATACATACTATGTTGTAGCCCACTTCCACTATGTCCTATCTATAGGAGCAGTCTTTGCCATCATGGGAGCATTCGTTCACTGATTCCCCCTATTCTCAGGCTACACCCTTCACAATACGTGAACAAAAATCCACTTCGGAGTTATGTTTGTAGGTGTAAACCTCACCTTTTTCCCTCAGCACTTCTTAGGATTGGCGGGAATACCTCGACGATACTCAGATTACCCTGACGCATACACACTGTGAAATACTATCTCATCCCTGGGGTCATTAATCTCCCTTATTGCTGTAATTATATTCCTATTTATTATCTGGGAGGCATTCGCGGCAAAACGTGAAGTCTTATCAGTTGAACTAACAGCCACAAACGTAGAATGACTGCACGGGTGTCCTCCCCCTTACCATACATTTGAAGAACCTGCATTCGTTCAAATTCAACAGTCCAAATTTTAATCGAGAAAGGAAGGAGTCGAACCCCCATAAACTGGTTTCAAGCCAGCCACATAACCGCTCTGTCACTTTCTTCCCTAAGTTAATAAGATTCTAGTTAAAGGAATAACACTGCCTTGTCAAGGCAAAATTGTGGGTTAAAGCCCCACGTATCTTGCTTATGGCACATCCATCTCAACTAGGATTCCAAGATGCAGCTTCACCCGTTATAGAAGAACTTCTCCACTTTCATGACCATGCATTAATAATTGTTTTCTTAATCAGCACCCTTGTTCTTTACATTATTGTGGCTATGGTAACCACCAAGCTAACAAATAAGTTCATTCTGGACTCCCAAGAAATTGAAATCATCTGAACCTTGCTACCAGCAATTATCCTAATTCTGATCGCCCTCCCCTCCCTTCGCATTCTCTACCTCATGGATGAAATCAATGACCCCCACCTCACAATTAAAGCCATAGGACATCAATGATACTGAAGCTACGAATATACGGATTATGAAGACCTAGGGTTCGACTCATATATGGTCCCTACACAAGACCTCGCCCCTGGTCAATTTCGACTACTTGAGACAGACCATCGCATGGTCATTCCTGTTGAGTCCCCCATCCGGGTTCTTGTCTCCGCCGAGGATGTTTTACACTCATGGGCCGTCCCAAGCCTCGGAGTAAAAATGGACGCCGTCCCCGGCCGCCTAAATCAAACAGCCTTCATTACTTCCCGTCCGGGTGTGTTTTATGGACAATGCTCAGAAATTTGCGGAGCTAATCATAGCTTTATACCCATTGTAGTGGAAGCTGTTCCTCTAGAACACTTCGAGAACTGATCTTACCTAATACTTCAAGATGCCTCACCAGGAAGCTAAAAGGGAATAGCATTAGCCTTTTAAGCTAAAAATTGGTGACTCCCGCCCACCCCTGGTGACATGCCTCAATTGAACCCCGCACCCTGATTTGCTATTATAGTATTTTCATGACTAGTTTTCCTAGCCGTTATCCCACCTAAAGTTCTAGCTCACCATTTTCCCAATGACCCCGCCCCACAGAGCGTAAAAAAATCAAAAACAGAGACCTGATCCTGACCATGACTTTAAGCCTCTTTGATCAATTTATGAGCCCTACACTTCTAGGGGTGCCTCTTATCGGACTCGCCCTAACACTGCCATGAGTCCTTTACTTCCAACCCGGTGCCCGATGACTTAATAACCGCTTGATTACCCTTCAATCTATATTCATGAACTGATTTGTAAAACAAATCTTTCAGCCAATAAGCTTAGGCGGACACAAATGGGCCGCTCTCCTCATATCTTTAATACTATTTTTAATTACCTTAAATATGCTAGGCCTACTGCCTTATACATTTACTCCAACAACGCAGCTGTCACTTAATATAGCCTTTGCAGTCCCACTTTGACTAGCAACTGTCATTATTGGAATACGAAACCAGCCAACACATGCCCTTGGTCACCTCCTCCCCGAAGGAACTCCTACCGCCCTAATCCCGGTTTTAATCGTGATTGAGACAATTAGCCTTTTTATTCGACCATTGGCCCTCGGTGTTCGACTTACCGCAAACTTGACAGCCGGGCACCTTCTAATTCAACTAATTGCAACTGCGGCTTTTGTTCTTTTCCCTATAATGCCCACAGTCGCCGCTCTTACCTCTGTCTTACTATTCTTGCTAACCCTACTAGAAGTCGCCGTGGCCATAATCCAAGCCTATGTATTCGTACTTCTTTTAAGCCTTTACCTACAAGAAAACGTCTAATGGCCCATCAAGCACATGCATATCATATAGTTGACCCAAGCCCTTGACCCCTCACAGGCGCAGTAGCCGCCCTTCTACTTACATCTGGAACAGCAATCTGAATACACTTTAACTCCACAGTTCTCATGTCCCTTGGACTTGTTCTGTTACTACTAACCATATATCAATGATGGCGAGACATTATCCGAGAGGGCACCTTTCAAGGTCATCATACACCCCCTGTTCAAAAAGGCCTTCGGTACGGGATAATTCTATTTATTACCTCAGAGGTCTTCTTTTTCCTAGGTTTCTTCTGAGCATTTTATCACTCAAGCCTAGCCCCGACCCCCGAACTTGGTGGATGTTGACCACCCATGGGTATTACAACACTGGACCCCTTTGAAGTTCCCCTTCTCAATACTGCTGTCCTTCTCGCCTCCGGTGTCACGGTCACTTGAGCTCACCATAGTATTATGGAGGGGCAGCGAAAACAAGCAATTCAGTCCTTGACACTCACAATTCTCCTGGGGTTTTACTTTACATTCCTTCAAGCAATAGAGTACTACGAGGCACCCTTCACCATTGCAGATGGCGTCTATGGCTCTACATTTTTTGTGGCAACAGGGTTTCATGGCCTCCATGTAATTATTGGATCAACATTTCTGGCAGTCTGCCTCTTACGACAAGTCCAGTTTCATTTTACATCAGAACATCACTTTGGATTCGAAGCTGCAGCATGATACTGGCACTTTGTAGACGTAGTCTGACTATTCTTATATATCTCTATCTACTGATGAGGCTCATATCTTTCTAGTATTAAAAAGTACAAGTGACTTCCAATCACTCAGTCTTGGTTAGACTCCAAGGAAAGATAATGAACTTAGTACTAGTCATTATTTGCATCTCATTAGCCCTCGCCGCACTGCTCGCAACTGTTTCATTTTTCCTCCCACAAATAACCCCTGATTATGAGAAACTCTCACCGTATGAGTGCGGCTTTGATCCAGTGGGGTCCGCCCGTTTGCCATTCTCCATTCGCTTTTTTCTAGTCGCAATCCTATTTCTCCTCTTCGACTTAGAAATTGCCTTACTTCTTCCCCTTCCCTGAGGGGACCAACTTCCCTCCCCTCTAACAACTTTCTTTTGAGCTTCTGCTATTCTTATACTACTAACTCTAGGGTTAATCTATGAGTGACTTCAAGGAGGCCTAGAGTGGGCAGAATAGGTACTTAGTTTAATAAAAACATTTGATTTCGGCTCAAAAACTTATGGTTTAAGTCCATATTTACCTGATGACCTTAACTCACTATGCATTCTCGTCAGCCTACTTTGTTAGCTTCATGGGTTTAATTTTTTACCGAAAGCATCTTCTCTCCGCCTTACTTTGCTTAGAAGCGATAATACTTATTCTTTTTATTTCACTATGCCTGTGAGGTCTAGTCTTAGCCTCAAGTGCATTTTCGGCAGGCCCAATGATCTTACTTGCTTTCTCAGCATGTGAAGCAAGTGCAGGCCTAGCACTGCTTGTAGCAATAGCTCGAACCCACGGTACTGACCGTCTAAAAAACCTTAGCCTACTCCAATGTTAATAATTCTTATTCCTACTGTTATGCTTCTACCCACAATCTGACTGAGCCCCACTAAATACCTGTGATCCTCAACACTTGGCCATAGCATAATAATTGCTCTTATAAGCCTCTCCTGACTTAGCCTCCCAGGGGAGGTTGGCTGATCTTCCCTTAACACTTTTATAGCAACAGACCCTCTCTCTACCCCCCTTCTCGTACTCACTTGCTGACTTCTGCCCTTAATAATTCTTGCGAGCCAAAACCATATAGCCCAAGAACCTACCAATCGCCAACGAACCTATATCTCTCTCCTTACTTCCCTTCAAATCTTCTTAATCTTAGCATTTGGGGCAACCGAGATAATCATGTTCTACATTATATTTGAAGCGACCTTAATTCCCACACTCGTAATTATCACACGATGAGGAAACCAAACAGAGCGATTAAACGCAGGTATCTACTTTTTATTTTATACCTTAGCCGGCTCTTTGCCACTACTAGTGGCCCTCCTTCTACTTCAGACCTCGACAGGAACTCTTTCTTTTCTAACCACTCAATTTTTTCCCCCTTTACAACTGCATACAGAAGCAAGTAAATTCTGGTGGGCAGGCTGTTTACTAGCATTCTTAGTAAAAATGCCCCTGTATGGAGCGCACCTTTGACTTCCAAAAGCTCACGTCGAAGCCCCCATCGCCGGGTCAATAGTCCTTGCAGCCGTTCTTTTAAAACTAGGGGGTTACGGTATGATACGAGTCATTATTATCTTAGAGCCCCTCACGAAACAACTCAGCTACCCCTTTATTGTTCTTGCCCTGTGGGGCGTCGTAATAACTGGCTCAATTTGCCTCCGACAAACAGACCTTAAATCACTAATCGCTTACTCCTCAGTAAGCCACATAGGCCTTGTCGCAGCAGGCATCCTAATCCAAACTCCTTGAGGGTTTACAGGGGCATTAATCCTTATAATTGCCCATGGCTTAACTTCCTCCGCCCTATTCTGTCTAGCCAACACTAACTATGAACGAACACATAGCCGAACCATGCTTTTAGCCCGGGGTCTACAAATGGTCCTTCCTCTCTTAGCAACTTGATGGTTTCTATTTACCCTCGCCAACCTAGCACTCCCTCCGCTACCCAACCTCATAGGAGAACTTATGATTATCTCATCCTTGTATAACTGATCAAACTGGTCTCTAATCCTGACCGGGGCGGGAGTACTAATTACCGCTAGTTACTCTCTCCATATATTCCTAACCACTCAACGTGGCCCTATTACTAAACCCGTCTTGGCAATTGAACCAACCCACACACGAGAACATCTCCTCATAATCCTTCACCTTCTTCCCCTCCTCCTTCTAATTTTAAAACCCTGCTTGATCTGGGGCTGAACAGTTTGTAGGCGTAGTTTAAATAAAGCGCTAGATTGTGATTCTAGAAATAAGAGTTAAACCCTCTTCACCCACCGAGAGGGGTCGCCGTGACAGCAAGAACTGCTAATTCTAGCCCCTTTGGTTAAAGTCCGAAGCCCACTCGAACAGGCTTCTAAAGGATAACAGCTCATCCGTTGGTCTTAGGAACCAAAAACTCTTGGTGCAACTCCAAGTAGCAGCTATGCACTTTACAACAATGATTCTCTCCTCAAGCCTAATAACAATTTTCCTTCTTCTTACCCTTCCAGTCCTAGGTACACTAAATCCTAACCCCACGGGGGACCTGTGAGCCACAAAAAACGTTAAAACAGCAGTTAAGATGGCCTTTTTTGTAAGTCTTCTACCTCTTTTTATCTTTCTTAATGAAGGAGTAGAGACTATTATAACAAACTGAAAATGGATAAATACTCTGATATTTGAAATTAATATCAGCTTTAAATTTGACCTCTACTCCGTGGTATTTACCCCTGTAGCCCTCTACGTAACATGATCAATTTTAGAGTTCGCATCTTGGTACATACACAGTGACCCCAATATAAACCGATTCTTTAAGTATCTTCTAATCTTTCTAATCGCTATGGTTGTTCTGGTTACAGCCAACAACATGTTCCAACTATTTATTGGCTGAGAAGGTGTTGGAATTATGTCTTTCTTACTTATTGGCTGGTGGTTCGGGCGGGCTGACGCTAACACTGCGGCCCTCCAGGCCGTAGTTTATAACCGAGTCGGTGATATCGGCCTAATTCTAGCAATAGCATGAATAGTAGTAAACCTAAACTCATGAGAGATACAACAGCTCTTTTCTGTGTCGAAAGGCCATGATATGACCCTTCCCTTATTAGGCCTAGTACTGGCCGCTACCGGAAAGTCCGCCCAGTTTGGACTTCACCCCTGGCTCCCCTCAGCCATAGAGGGTCCAACACCGGTCTCTGCCCTCCTGCACTCTAGCACCATGGTTGTTGCTGGTATTTTCCTTCTTATCCGCCTCAGCCCCTTAATGCAAGAAAGCCCGTTAATTCTCTCAACATGCCTTTGCCTGGGGGCCCTAACTACCGTCTTTACTGCAACATGTGCCCTTACCCAAAATGACATCAAAAAAATTGTTGCATTTTCTACATCAAGTCAATTAGGACTAATAATAGTTACCATCGGACTAGGCCAGCCCCAGCTCGCCTTTCTTCATATCTGCACCCACGCCTTCTTTAAAGCAATACTTTTCTTATGTTCAGGCTCCATCATTCATAGCCTTAATGATGAGCAAGATATCCGAAAAATAGGAGGACTTCACAAGCTCCTTCCACTGACCTCTTCTTGTCTAACCATTGGCAGCCTAGCTCTAACAGGAGTCCCCTTTTTAGCAGGCTTCTTTTCCAAAGACGCCATCATTGAAGCTATAAATACATCCTACCTTAACGCCTGGGCCCTAATTTTAACGCTTCTAGCTACATCATTTACCGCAGTTTACAGTCTCCGAGTCGTATTCTTTGCCTCTATGGGCCACCCGCGTTTTAATCCAATCTCCCCTATTAATGAAAATAACCCTACAGTAATAAACCCTATTAAACGACTCGCTTGGGGAAGCATTTTGGCAGGGTTGCTAATTACGGCCAATATTGTTCCACTTAAAACCCCCGTTTTAACCATGCCTTTCACCTTAAAAATGGCCGCACTGGCTGTAACAATTATAGGACTACTCACAGCCTTAGAACTAGCATCTCTCACGTCCCAACAATTTAAAATCAAACCCTTATCTTCTACTCACCACTTCTCAAATATATTAGGATTTTTCCCGAGTGTGGCCCATCGACTAGTCCCAAAAGCTGGCCTGATTCTTGGGCAACTAGTTGCCAATCAGACAATTGACCAAACCTGACTAGAGAAAACCGGGCCAAAAATAGTAGCCTCCGTTAACCTTCCAATGGCTACTTCAATTAGCAACCTACAGCAGGGTGTAATTAAGACCTACTTCTTATTATTTTTCTTCACCATAATACTGGCAATTCTCATCCTTGTCATCTAACTGCCCGTAAGGTCCCCCGACTTAGCCCTCGAGTTAACTCCAGAACTACAAAAAGCGTCAGTAATAAAACTCATCCCCCAAGCATTAAAACCCCTCCTCCTGAAGAATATATCAGAGCAACCCCACCAAGATCCCCCCGGAATAGCATGAATTCACTAAACTCGTCAGCAGTTATCCATGACCCCTCATACCAACCCTCAGAGAAAAAGACAGAGATAGACGCGACCAGAAACACATATACTGACATAAGAAGCAAAACGGGTCAACTTCCCCACCCCTCAGGATAGGGCTCCGAAGCCAGCGCTGCTGAATACGCAAACACAACTAACATCCCACCTAAATAGATCAAAAACAAAATCAGAGATAAAAATGAACCCCCGTGCCCTACTAAAATGCCACAGCCCATTCCTGCTACTGTGACAAGGCCCAAAGCAGCAAAGTAGGGTGAGGGGTTTGAGGCCACGGCCGCTAGACCTAAAACCAGACCAACTAATAATAAATAAGTTATATAAACCATAATTCTTGCCAGGATTTTAACCAGGGCCTGCGACTTGAAAAACCACCGTTGTACTCAACTACAAGAACCTAATGGCCAATCTTCGAAAAACCCATCCCCTATTAAAAATCGCAAACGATGCCCTGGTTGATCTCCCAGCCCCATCGAACATTTCAGTTTGATGAAACTTCGGGTCTCTTCTAGGACTTTGTTTGGCCGCCCAGATTGTTACGGGCCTTTTCCTTGCAATACATTATACATCAGACATTGCCACAGCATTTTCATCTGTAGCACATATTTGTCGTGACGTCAACTACGGCTGACTAATCCGAAACATGCATGCAAACGGTGCTTCCTTTTTCTTCATTTGCATCTACCTGCACATCGGACGGGGCTTGTATTATGGATCATACTTATATAAAGAGACATGAAATGTGGGTGTTGTCCTTCTCCTCCTAGTGATAATGACTGCTTTCGTAGGCTACGTCCTACCCTGAGGACAAATGTCATTCTGAGGAGCTACCGTCATTACCAACCTTTTATCAGCCATTCCCTACGTTGGAAACGCCCTAGTTCAATGAATCTGAGGCGGATTTTCAGTAGACAACGCCACCCTTACCCGGTTCTTTGCCTTCCATTTCCTCCTTCCCTTTGTGATTGCTGCTGCTACAGTTGTGCATCTTATCTTCCTGCACGAGACAGGATCGAATAATCCAACGGGTTTAAACTCAGACTCTGACAAAGTATCTTTTCACCCCTACTTTTCTTATAAAGATCTTCTAGGCTTTGCTGCCCTACTAGTAGCCCTTATCTCTTTAGCCCTCTTCTCCCCAAATCTACTCGGAGACCCCGATAACTTTACCCCTGCTAATCCTTTAGTGACTCCACCTCACATCAAACCTGAGTGATACTTCTTGTTCGCTTACGCCATTCTACGATCCATCCCAAACAAACTTGGCGGGGTTCTAGCCCTATTAGCCTCTATTCTAGTCCTCTTTCTTGTTCCCATTCTGCATACATCAAAACAACGAAGCCTAACATTCCGGCCCCTGACCCAATTCCTCTTCTGATTGCTAGTCGCCGATGTAATAATTTTAACCTGAATTGGAGGTATGCCTGTAGAACATCCTTACATTATTATTGGACAAATCGCATCCTTCATTTATTTTTTCCTTTTCCTAGTCATAGCGCCTATGGCCGGCCTACTAGAAAACAAAGTCTTAAAATGACAATGCATTAGAAGCTCAGATGAAAGAGCACCGGTCTTGTAAACCAGAGGTCGAAGGTTCAAGCCCTTCCTAGTGCTCAGAGAGAAGGGATTCTAACCCCTGCCCCTGACTCCCAAAGCCAGGATTCTTAGCTAAACTACTCCCTGATTTTCATACACCAGTTTTGCAATCCAGAGCGCATCACTTTTGCCACCAACGTTAAATTAACGTTGCACAAACGTTGCATCAGCGCCCCATGGACACTAAATGACGCGAGGGCGCTAAATAAACACCCCCTACCTCTAGCACCCTTTTAACGATTTCACTTTTTTTTTTTTTTTTGTTTAACGATTACGTTTTTTTTTGCGTTCCCGGACTCTGCCAGATTTCGACCGAAGACTGCCAGAATCCGCTCAAAATCCGCTCAAATACCAATATGTATTATCCCCATAAATGGTTTAAACCATTTTTGCCTAGTACACACTGACCATGCAAGTCAATTATATTTACCCCGCGCTCCAGGCCGCAGTACATACACCTACAGTTGGTGTATTTAGCACAAGTGTGCCTCAGCTAGTTTCAAGTCACCCACATCCTTCCTTTAATTGTTACTTAATGTAGTAAGAGCCCACCATCAGTTGATTCCTTAATGTCAACGGTTCTTGAAGGTGAGGGACAAAAATCGTGGGGGTTTCACTTCTTGAATTATTCCTGGCATTTGGCTCTACATCTCAAGGCCATACATTTCTCGTCTCTCACACTTTCACTGGCCCTGACATTGGTTAATGGTGGAGTACATACTCCTCGTTACCCCCCATGCCGGGCGTTCTTTCTAATGGACAACGGGTTTTCCTTTTTTTTTCCTTTTCACTTGGCATTTCACAGTGCATACAAACCTTGATGACAAGGTTGAACATTTAGAAATCGGCCGCAAAGAATATTGGTGAATTATTTAAAGATATTAACAGATGAATTGCATAAGTGATATCAAGAGCATAAATAACCAAATGAAACTAGGAACGTTTCTATAATATGCCCCCCGGCTTCCGCGCGTCAAACCCCCCTACCCCCCTAAACTAGTAAGAAGTCTATTATTCCTGCAAACCCCCCGGAAACAGGAAACCCCCTACTAGCATTTTAGCCCGCCCAAATTTGTGTGTATTTATATTATTTGTAATATTGCAAAA

>GQ 13

GCTAGTGTAGCTTAACTAAAGCATAACACTGAAGATGTTAAGACAAACCTTAGATTGGTTTCACGAGCACAAAAGTTTGGTCCTGACTTTACTATCAACTTTAGCTAAACTTACACATGCAAGTATCCGCAATCCCGTGAGAATGCCCTACAGTTTCCTTAAAGGAAACAAGGAGCTGGTATCAGGCTCAATTACTCCCGCCCATGACACCTTGCTTAGCCACACCCCCAAGGGAACTCAGCAGTGATAGACATTAAGCAATAAGTGAAAACTTGACTTAATTAAAGCTAAGAGAACCGGTTAAACTCGTGCCAGCCACCGCGGTTATACGAGCGGTTCGAGCTGATAGATTACGGCGTAAAGCGTGGTTAATAAGAATGAAACTAAAGTCGAATGTTTTCAAAGCTGTTATACGCACTCGAAAATTAGAAGGTCAGAAACGAAAGTGACTTTAACCCTATGAACCCACGAAAACTATGAAACAAACTGGGATTAGATACCCCACTATGCATAGCTGTAAACTTTGATGAGCTATTACATTATCATCCGCCTGGGTACTACGAGCATCAGCTTAAAACCCAAAGGACTTGGCGGTGCTTTAGACCCACCTAGAGGAGCCTGTTCTAAAACCGATAACCCCCGTTAAACCTCACCCTCTCTTGTTTTTCCCGCCTATATACCGCCGTCGTCAGCTTACCCTGTGAAGGTCTAATAGTAAGCACAACCAGTTATACTCAAAACGTCAGGTCGAGGTGTAGCATATGAGAGGGGAAGAAATGGGCTACATTCCTTGTTTCAAGGAAAACGGATAACATAATGAAAGGTACGTTAGAAGGAGGATTTAGCAGTAAGCAGCAAATAGAGTGTTCTGCTGAAACTGGCCCTGAAGCGCGCACACACCGCCCGTCACTCTCCCCAACTCCGAGTTAAAAACATATATAAACCTTTGAAGGAACAAAGGGGAGGCAAGTCGTAACATGGTAAGTGTACCGGAAGGTGCACTTGGATAAATCAGAGTATAGCTAAGAAAGAAAAGCATCTCCCTTACACCGAGAAGTCATCCGTGCAAATCGGATTACCCTGACTCTAACAAGCTAGCCCAAAACCTTAACTTAAAAATCAAATATTTCTAGTAATTAATAAACCAAACACATTAAATAAATCATTTTTCCCCCTGAGTATGGGAGACAGAAAAGGATAAAGGAGCTATAGACAAAGTACCGCAAGGGAAAGCTGAAAGAGAAATGAAACAAACCAGTAAAGAAAAACAAAGCAGAGATTAACCCTTGTACCTTTTGCATCATGAATTAGCCAGTTTAATCAAGCAAAGAGCACTGTAGTTTGAGACCCCGAAACTTAGTGAGCTACTTCAAGACAGCCTACGAAATAGGGCAAACCCGTCTCTGTGGCAAAAGAGTGGGAAGATCTTCAAGTAGAGGTGACAGACCTATCGAACTAAGTAATAGCTGGTTGCTCGTGAAATGAATAGAAGTTCAGCCTTTTGCTTTCTAAATTTCGATTTAGCATCACTTAGCCTAAATGATTAGAAAACAAAAGAGTTAGTCAAAGAGGGTACAGCCTGTTTGATAAAAGATACAACTTTACTAGGAGGATAAGAATCATAATTTTAAAGGTTTAATGCCCAGGTGGGCCTAAAAGCAGCCACCCTGATCAATAGCGTTAAAGCTTAAGCATAAAACACACCTACAATTCTGATAAATCAGTTTTAATCCCCTAAAGTTAACGAGCTATTTCATACCTTATGAAAGAAATTATGCTAGTATGAGTAATAAGAAGTTACGAACTTCTCCCTGCACACGTGTAAATCGGAACGGACAAACCACCGAATCTTAACGGCCCCAGTCAAAGAGGGGATGTCGGATAAAAAAAAGAACAAGAAATTCCCGATAAAACCACCGTTGACCCCACACCGGAGTGCTCCCTGGGAAAGACAAAAAGGGACAGAAGGAACTCGGCAAATATGCTCAAGCCTCGCCTGTTTACCAAAAACATCGCCTCTTGTAAAAGTTAAATAAGAGGTACCGCCTGCCCTGTGACTAGTAGTTTAACGGCCGCGGTATTTTGACCGTGCAAAGGTAGCGCAATCACTTGCCTTTTAAATGAAGGCCTGTATGAATGGCACGACGAGGGCTTAACTGTCTCCTCTCCCTAGTCAATGAAATTGATCCCCCCGTGCAGAAGCGGGGATAATAACATAAGACGAGAAGACCCTGTGGAGCTTTAGACTATGAGCAGACCATGTCAAGAATAACAAACAAGTAAATTAAACAAATTGGCCCCTGCTTCCCTGTCTTTGGTTGGGGCGACCGCGGGATAATAAAAAACTCCCACGAGGATTGAGAACCCTTATCTTATAACCAAGAGCTTCTCCTCTAAGTAACAGAACATCTGACCTTAATGATCCGGCCTGGCCGATCAACGGACCGAGTTACCCCAGGGATAACAGCGCAATCCTCTTTTAGAGTCCATATCGACAAGAGGGTTTACGACCTCGATGTTGGATCAGGACATCCTAATGGTGCAGCCGCTATTAAGGGTTTGTTTGTTCAACAATTAAAGTCCTACGTGATCTGAGTTCAGACCGGAGTAATCCAGGTCAGTTTCTATCTATGACGTACTCTCTTCTAGTACGAAAGGACCGAAGAAAGAAGGCCCATGAAAAATTATGCCTTAGTCTCACCTTATGAAGAAAACTAAATAAGACAAGAGGTTACACCCTTTGGTCATAGAAAATGACATGTTAAGGTGGCAGAGCCCGGATATTGCAAAAGACCTAAGCCCTTTCCACAGAGGTTCAATTCCTCTCCTTAACTATGTTCTCAACAATATTAAGCTTCATTATTAATCCCCTGATTGTTATGATTTTTGTTTTGTTGGCAGTAGCCCTCTTGACCTTGGTAGAGCGTAAAGTGCTAAGCTACATGCAACTTCGTAAAGGCCCAAATGTTGTTGGCCCTTACGGCCTTTTGCAACCCTTCGCTGATGGCTTAAAACTTTTCATGAAAGAGCCCGTCCGACCCTCCACCTCCTCGCCCGCCTTGTTCTTAATTACCCCTATTATAGCCCTTACCTTAGCCCTAACCCTCTGAGCCCCCCTTCCTATGCCTTTTCCCGTCACCGACCTAAACTTAGGCATTTTATTTATTTTAGCACTATCGAGCCTGGCAGTATATTCTATTCTTGGCTCCGGATGAGCCTCCAATTCTAAATATGCATTAATTGGTGCTCTTCGAGCGGTCGCCCAAACCATCTCTTATGAAGTAAGCTTGGGCCTTATTCTTCTTAACACAATTGTCTTTACGGGGGGTTTTACTCTTCAAACCTTCAGCACCGCACAAGAAGCCACCTGATTACTTCTACCAGCATGACCACTAGCAGCCATGTGATATATCTCCACACTCGCGGAAACTAACCGGGCCCCTTTCGACCTAACTGAAGGAGAGTCCGAACTAGTGTCTGGCTTCAACGTAGAGTATGCCGGCGGACCTTTTGCCCTTTTTTTTCTGGCAGAATACGGTAACATTTTACTTATAAATACCCTCTCAGCAGTACTATTTCTAGGCTCTTCAACCTACCACAACTTTCCAGAACTAACCGCGACCTTATTAATGCTTAAAGCCACCCTCCTTTCAGTCGTATTTTTATGAGTGCGAGCATCTTACCCTCGGTTCCGATACGACCAACTAATGCATTTAATTTGAAAAAACTTTTTACCTCTGACCCTAGCGCTAGTTATTTGACACCTTTCTCTTCCAATCACGTTGAGCGGCCTCCCTCCTCAACTTTAACTCAGGAAATGTGCCTGAAAAAGGGTCACTTTGATAGGGTGAATAATGAGGGTTAAAGCCCCTCCATCTCCTTAGAAAGAAGGGGTTTGAACCCTACCTGAAGAGATCAAAACTCTTAGTGCTTCCACTACACCACTTCCTAGTAAAGTCAGCTAATAAAAGCTTTTGGGCCCATACCCCAAATATGTTGGTTAAAATCCTTCCTTTGCTAATGAATCCTTACGTCCTTTCAATTCTACTTATGGGCTTAGGCCTCGGCACTACAGTCACATTCGCTAGCTCACACTGACTCTTAGCATGAATAGGCCTTGAAATAAATACCCTCGCCATTTTGCCATTAATAGCACAACATCACCACCCCCGAGCCGTTGAAGCCACCACCAAGTATTTTTTAATTCAATCGGCAGCCGCAGCAACCATCTTATTTGCCAGCTCAACTAACGCCTGACTTTCGGGCCAGTGGGATATCATAAGTATCAATCACCCCCTTCCAACCGTCATAATTACAATCGCTCTGTCCTTAAAACTAGGCTTGGCCCCTCTTCACGCCTGACTTCCCGAAGTTATTCAAGGACTAGACTTGACCACGGGCTTAATCCTCTCCACATGACAAAAACTCGCACCCTTTGCCCTTCTCGTTCAAATCTTCCCCGACACCCCCCTTCTCATCACTTCTCTAGGACTTCTTTCAATATTAATTGGGGGGTGAGGAGGTTTAAACCACACACAACTCCGCAAAGTGCTCGCATATTCTTCGATCGCCCACTTAGGCTGAATAATAGTAATTATGCAATTCTCCACCCCCCTTACAATTCTTGCTTTATCAACATACATTGTTATAACATCATCTACTTTTCTAATCTTTAAACTCCTTAAATCCACAAATATGAACAGCCTGGCAACATCTTGAACTAAAACCCCCTCCATTACAGCCCTAGCACCTTTAGTGCTATTATCCTTAGGCGGACTTCCTCCCCTCTCGGGCTTTATGCCAAAATGATTAATTATTCAAGAATTAACTAAGCAAGATCTAGCCCTAGTTGCAACCTTGGCCGCCCTCTCTGCGCTACTCAGCCTTTTCTTCTACCTACGCATTTGTTACTCCCTCACATTTACCTCCTCTCCTAATAATCTCATGGGAACACCCCCCTGACGACTAGTAACAAAGCAAGTATCACTTCCCCTAGCTATAACAACCTCCCTCTCTATTCTTCTACTCCCGGTTACCCCTGCAATCTTATCAGTGGTTCTCCCTTTGTAAAGAGGCTTAGGATAGTATTAAGACCAAGGGCCTTCAAAGCCCTAAGCGGGAGTGAAAGCCCCCCAGCCTCTGTAAGACCTACGGGACACTAACCCACATCTTCTGTATGCAAAACAGACACTTTAATTAAGCTAAAGCCTTCCTAGGTGGGTAGGCCTCGATCCTACAATCTCTTAGTTAACAGCTAAGCGCCTAAACCAGCGGGCATCCATCTACCTTTCCCCCGCCTTGCCGAAAAAAAAAGGCGGGGGAAAGCCCCGGCAGGGTATTAGCCTGCCACTTAAGATTTGCAATCTAATGTGTTAACACCTCGGGGCTGGTAAGAAGAGGACTTTAACCTCTGTCCATGGGGCTACAATCCACCGCTAAACGCTCAGCCACCTTACCTGTGGCAATCACACGTTGATTTTTCTCAACTAATCACAAAGACATCGGCACCCTGTATCTAATCTTTGGTGCCTGGGCGGGAATGGTAGGGACGGCCTTAAGTCTACTCATTCGGGCAGAACTAAGTCAACCAGGCTCCCTATTAGGAGACGACCAGATCTATAACGTAATTGTAACTGCACATGCTTTCGTAATAATTTTCTTTATAGTAATGCCAATCATAATTGGGGGGTTTGGCAACTGATTAATCCCTTTAATGATTGGAGCCCCCGACATGGCCTTCCCACGGATAAATAATATAAGCTTTTGACTCCTGCCCCCTTCTTTCCTTCTATTATTGGCCTCATCTGGTGTAGAAGCTGGTGCCGGAACAGGATGAACCGTATATCCCCCCTTGTCAGGTAATTTGGCACACGCAGGGGCCTCCGTAGATTTAACCATTTTCTCTCTTCACCTGGCCGGAATTTCTTCTATCCTAGGGGCCATTAATTTCATCACAACTATTATTAATATAAAACCTCCAGCCATTTCCCAATATCAAACCCCTTTATTTGTGTGGGCTGTACTAATTACCGCAGTATTACTTCTACTCTCTCTTCCTGTTCTAGCTGCAGGTATCACCATGCTTCTCACAGATCGGAACCTAAATACAACATTTTTCGACCCCGCAGGAGGGGGGGACCCCATTCTTTATCAACATTTATTCTGATTCTTTGGGCATCCTGAAGTCTACATTCTAATTTTGCCCGGCTTCGGAATGATTTCTCACATTGTAGCATATTACTCAGGCAAAAAAGAGCCGTTTGGTTACATGGGAATAGTATGAGCTATAATAGCAATTGGCTTGCTGGGCTTTATCGTATGAGCCCATCATATGTTCACTGTAGGGATGGACGTGGACACTCGAGCTTATTTTACTTCCGCCACTATAATTATCGCAATTCCCACAGGAGTCAAAGTGTTTAGTTGACTAGCTACCTTGCATGGGGGCTCAATCAAATGAGAAACCCCCCTGTTATGAGCTCTAGGCTTTATCTTCTTATTTACTGTCGGAGGTTTAACAGGAATTGTTTTAGCCAACTCATCTCTGGACATTATACTTCATGATACATACTATGTTGTAGCCCACTTCCACTATGTCCTCTCTATAGGAGCAGTCTTTGCCATCATGGGAGCATTCGTTCACTGATTTCCCCTATTCTCAGGCTACACCCTTCACAATACGTGAACAAAAATCCACTTCGGAGTTATGTTTGTAGGTGTAAACCTCACCTTTTTCCCTCAGCACTTCTTAGGATTGGCGGGAATACCTCGACGATACTCAGATTACCCTGACGCATACACACTGTGAAATACTATCTCATCCCTGGGGTCATTAATCTCCCTTATTGCTGTAATTATATTCCTATTTATTATCTGGGAGGCATTCGCGGCAAAACGTGAAGTCTTATCAGTTGAACTAACAGCCACAAACGTAGAATGACTGCACGGGTGTCCTCCCCCTTACCATACATTTGAAGAACCTGCATTCGTTCAAATTCAACAATCCAAATTTTAATCGAGAAAGGAAGGAGTCGAACCCCCATAAACTGGTTTCAAGCCAGCCACATAACCGCTCTGTCACTTTCTTCCCTAAGTTAATAAGATTCTAGTTAAAGGAATAACACTGCCTTGTCAAGGCAAAATTGTGGGTTAAAGCCCCACGTATCTTGCTTATGGCACATCCATCTCAACTAGGATTCCAAGATGCAGCTTCACCCGTTATAGAAGAACTTCTCCATTTTCATGACCATGCATTAATAATTGTTTTCTTAATCAGCACCCTTGTTCTTTACATTATTGTGGCTATGGTAACCACCAAGCTAACAAATAAGTTCATTCTGGACTCCCAAGAAATTGAAATCATCTGAACCTTGCTACCAGCAATTATCCTAATTCTGATCGCCCTACCCTCCCTTCGCATTCTCTACCTCATGGATGAAATCAATGACCCCCACCTCACAATTAAAGCCATAGGACATCAATGATACTGAAGCTACGAATATACGGATTATGAAGACCTAGGGTTCGACTCGTATATGGTCCCTACACAAGACCTCGCCCCTGGTCAATTTCGACTACTTGAAACAGACCATCGCATGGTCATTCCTGTTGAGTCCCCCATCCGGGTTCTTGTCTCCGCCGAGGATGTTCTACACTCATGGGCCGTCCCAAGCCTTGGAGTAAAAATGGACGCCGTCCCCGGCCGCCTAAATCAAACAGCCTTCATTACTTCCCGTCCGGGTGTGTTTTATGGACAATGCTCAGAAATTTGCGGAGCTAATCATAGCTTTATACCCATTGTAGTGGAAGCTGTTCCTCTAGAACACTTCGAGAACTGATCTTACCTAATACTTCAAGATGCCTCACCAGGAAGCTAAAAGGGGATAGCATTAGCCTTTTAAGCTAAAAATTGGTGACTCCCGCCCACCCCTGGTGACATGCCTCAGTTGAACCCCGCACCCTGATTTGCTATTATAGTATTCTCATGACTAGTTTTCCTAGCCGTTATCCCACCTAAAGTTTTAGCTCACCATTTTCCCAATGACCCCGCCCCACAAAGCGTAAAAAAATCAAAAACAGAGACCTGATCCTGACCATGACTTTAAGCCTCTTTGATCAATTTATGAGCCCTACACTTCTAGGGGTGCCTCTTATCGGACTCGCCCTAACATTGCCATGAGTCCTTTACTTCCAACCCGGTGCCCGATGACTTAATAACCGCTTCATTACCCTTCAATCTATATTCATGAACTGATTTGTAAAACAAATCTTTCAGCCAATAAGCTTAGGCGGACACAAATGGGCCGCTCTCCTCATATCTTTAATACTATTTTTAATTACCTTAAATATGCTAGGCCTACTGCCTTATACATTTACTCCAACAACGCAGCTGTCACTTAATATAGCCTTTGCAGTACCACTTTGACTAGCAACTGTCATTATTGGAATACGAAATCAGCCAACACATGCCCTTGGTCACCTCCTCCCCGAAGGAACTCCTACCGCCCTAATCCCGGTTTTAATCGTGATTGAGACAATTAGCCTTTTTATTCGACCATTGGCCCTCGGTGTTCGACTTACCGCAAACTTGACAGCCGGACACCTTCTAATTCAACTAATTGCAACTGCGGCTTTTGTTCTTTTCCCTATAATGCCCACAGTCGCCGCTCTTACCTCTGTCTTACTATTGTTGCTAACCCTACTAGAAGTCGCCGTGGCCATAATCCAAGCCTATGTATTCGTACTTCTTTTAAGCCTTTACCTACAAGAAAACGTCTAATGGCCCATCAAGCACATGCATATCATATAGTTGACCCAAGCCCTTGACCCCTCACAGGCGCAGTAGCCGCCCTTCTACTTACATCTGGAACAGCAATCTGAATACACTTTAACTCCACGGTTCTCATGTCCCTTGGACTTGTTCTGTTACTACTAACCATATATCAATGATGGCGAGACATTATCCGAGAGGGCACCTTTCAAGGTCATCATACACCCCCTGTTCAAAAAGGCCTTCGGTACGGGATAATTCTGTTTATTACCTCAGAGGTCTTCTTTTTCCTAGGTTTCTTCTGAGCATTTTATCACTCAAGCCTAGCCCCAACCCCCGAACTTGGTGGATGTTGACCACCCATGGGTATTACAACACTGGACCCCTTTGAAGTTCCCCTTCTCAATACTGCTGTCCTTCTCGCCTCCGGTGTCACGGTCACTTGAGCTCACCATAGTATTATGGAGGGGCAGCGAAAACAAGCAATTCAGTCCTTGACACTCACAATTCTCCTGGGGTTTTACTTTACATTCCTTCAAGCAATAGAGTACTACGAGGCACCCTTCACCATTGCAGATGGCGTCTATGGCTCTACATTTTTTGTGGCAACAGGGTTTCATGGCCTCCATGTAATTATTGGGTCAACATTTCTGGCAGTCTGCCTCTTACGACAAGTCCAGTTCCATTTTACATCAGAACATCACTTCGGATTCGAAGCTGCAGCATGATACTGACACTTTGTAGACGTAGTCTGACTATTCTTATATATCTCTATCTACTGATGAGGCTCATATCTTTCTAGTATTAAAAAGTACAAGTGACTTCCAATCACTCAGTCTTGGTTAGACTCCAAGGAAAGATAATGAACTTAGTACTAGTCATTATTTGCATCTCATTAGCCCTCGCCGCACTGCTCGCAACTGTTTCATTTTTCCTCCCACAAATAACCCCTGATTATGAGAAACTCTCACCGTATGAGTGCGGCTTTGATCCAGTGGGATCCGCCCGTTTGCCATTCTCCATTCGCTTTTTTCTAGTCGCAATCCTATTTCTCCTCTTCGACTTAGAAATTGCCTTACTTCTTCCCCTTCCCTGAGGGGACCAACTTCCCTCCCCTCTAACAACTTTCTTTTGAGCTTCTGCTATTCTTATACTACTAACTCTAGGGTTAATCTATGAATGACTTCAAGGGGGCCTAGAGTGGGCAGAATAGGTACTTAGTTTAATAAAAACATTTGATTTCGGCTCAAAAACTTATGGTTTAAGTCCATATTTACCTGATGACCTTAACTCACTATGCATTCTCGTCAGCCTACTTTGTTAGCTTCATGGGTTTAATTTTTTACCGAAAGCATCTTCTCTCCGCCTTACTTTGCTTAGAAGCGATAATACTTATTCTTTTTATTTCACTATGCCTGTGAGGTCTAGTCTTAGCCTCAAGTGCATTTTCGGCAGGCCCAATGATCTTACTTGCTTTCTCAGCATGTGAAGCAAGTGCAGGCCTAGCACTGCTTGTAGCAATAGCTCGAACCCACGGTACTGACCGTTTAAAAAACCTTAGCCTACTCCAATGTTAATAATTCTTATTCCTACTGTTATGCTTCTACCCACAATCTGACTGAGCCCCACTAAATACCTGTGATCCTCAACACTCGGCCATAGCATAATGATTGCTCTTATAAGCCTCTCCTGACTTAGCCTCCCAGGGGAGGTTGGCTGATCTTCCCTTAACACTTTTATAGCAACAGACCCTCTCTCTACCCCCCTTCTCGTACTTACTTGCTGACTTCTGCCCTTAATAATTCTTGCGAGCCAAAACCATATAGCCCAAGAACCTACCAATCGCCAGCGAACCTATATCTCTCTCCTTACTTCCCTTCAAATCTTCTTAATCTTAGCATTTGGGGCAACCGAGATAATCATGTTCTACATTATATTTGAAGCGACCTTAATTCCCACACTCGTAATTATCACACGATGAGGAAACCAAACAGAACGATTAAACGCAGGTATTTACTTTTTATTTTATACCTTAGCCGGCTCTTTACCACTACTAGTGGCCCTCCTTCTACTTCAGACCTCGACAGGAACTCTTTCTTTTCTAACCACTCAATTTTTTCCCCCTTTACAACTGCATACAGAAGCAAGTAAATTCTGGTGGGCAGGCTGTTTACTAGCATTCTTAGTAAAAATGCCCCTATATGGAGCACACCTTTGACTTCCAAAAGCTCACGTCGAAGCCCCCATCGCCGGGTCAATAGTCCTTGCAGCCGTTCTTTTAAAACTAGGGGGTTACGGTATGATACGAGTTATTATTATCTTAGAGCCCCTCACGAAACAACTCAGCTACCCCTTTATTGTTCTTGCCCTGTGGGGCGTCGTAATAACTGGCTCAATTTGCCTCCGACAAACAGACCTTAAATCACTAATCGCTTACTCCTCAGTAAGCCACATAGGCCTTGTCGCAGCAGGCATCCTAATCCAAACTCCTTGGGGGTTTACAGGAGCATTAATCCTTATAATTGCCCATGGCTTAACTTCCTCCGCCCTATTCTGTTTAGCCAACACTAACTATGAGCGAACACATAGCCGAACCATGCTTTTAGCCCGGGGTCTACAAATGGTCCTTCCTCTCTTAGCAACTTGATGGTTTCTATTTACCCTCGCCAACCTAGCACTCCCTCCACTACCCAACCTCATAGGAGAACTTATGATTATCTCATCCTTGTATAACTGGTCAAACTGGTCTCTAATCCTGACCGGGGCGGGAGTACTAATTACCGCTAGTTACTCTCTCCATATATTCCTAACCACTCAACGTGGCCCTATTACTAACCCCGTCTTGGCAATTGAACCAACCCATACACGAGAACATCTCCTCATAATCCTTCACCTTCTTCCCCTCCTCCTTCTAATTTTAAAACCCTGCTTGATCTGGGGCTGAACAGCTTGTAGGCGTAGTTTAAATAAAGCGCTAGATTGTGATTCTAGAAATAAGAGTTAAACCCTCTTCACCCACCGAGAGGGGTCGCCGTGACAGCAAGAACTGCTAATTCTAGCCCCTTTGGTTAAAGTCCGAAGCCCACTCGAACAGGCTTCTAAAGGATAACAGCTCATCCGTTGGTCTTAGGAACCAAAAACTCTTGGTGCAACTCCAAGTAGCAGCTATGCACTTTACAACAATGATTCTCTCCTCAAGCCTAATAACAATTTTCCTTCTTCTTATCCTTCCAGTCCTAGGTACACTAAATCCTAACCCCACAGGGGACCTGTGGGCCACAAAAAACGTTAAAACAGCAGTTAAGATGGCCTTTTTTGTAAGCCTTCTACCTCTTTTTATCTTTCTTAATGAAGGAGTAGAGACTATTATAACAAACTGAAAATGGATAAATACTCTAATATTTGAAATTAATATCAGCTTTAAATTTGACCTCTACTCTGTGGTATTTACCCCTGTAGCCCTCTACGTAACATGATCAATTTTAGAGTTCGCATCTTGGTACATACACAGTGACCCCAATATAAACCGATTCTTTAAGTATCTTCTAATCTTTCTAATCGCTATGGTTGTTCTGGTTACAGCCAACAACATGTTCCAACTATTTATTGGCTGAGAAGGTGTTGGAATTATGTCCTTCTTACTTATTGGCTGGTGGTTCGGGCGGGCTGACGCCAACACTGCGGCCCTCCAAGCCGTAGTTTATAACCGAGTCGGTGATATCGGCCTAATTCTAGCAATAGCATGAATAGTAGTAAACCTAAACTCATGAGAGATACAACAGCTCTTTTCTGTGTCTAAAGGCCATGACATGACCCTTCCCTTATTAGGCCTAGTACTGGCCGCTACCGGAAAGTCCGCCCAGTTTGGGCTTCACCCCTGGCTCCCCTCAGCCATAGAGGGTCCAACACCGGTCTCTGCCCTCCTGCACTCTAGCACCATGGTTGTTGCTGGTATTTTCCTTCTTATCCGCCTCAGCCCCTTAATGCAAGAAAGCCCGTTAATTCTCTCAACATGCCTTTGCCTGGGGGCCCTAACTACCGTCTTTACTGCGACATGTGCCCTTACCCAAAATGACATCAAAAAAATTGTTGCATTTTCTACATCAAGTCAACTAGGACTAATAATAGTTACCATCGGACTAGGCCAGCCCCAGCTCGCCTTTCTTCATATCTGCACCCACGCCTTCTTTAAAGCAATACTTTTCTTATGTTCAGGCTCCATCATTCATAGCCTTAATGATGAACAAGATATCCGAAAAATAGGAGGACTTCACAAGCTCCTTCCACTGACCTCTTCTTGTCTAACCATTGGCAGCCTAGCTCTAACAGGAGTCCCCTTTTTAGCAGGCTTCTTTTCCAAAGACGCCATCATTGAAGCTATAAATACATCCTACCTTAACGCCTGGGCCCTAATTTTAACGCTTCTAGCTACATCATTTACCGCAGTTTACAGTCTCCGAGTCGTATTCTTTGCCTCTATGGGCCACCCGCGTTTTAATCCAATCTCCCCAATTAATGAAAATAACCCTACAGTAATAAACCCTATTAAACGACTCGCTTGAGGAAGCATTTTGGCAGGGTTGCTAATTACGGCCAATATTGTTCCACTTAAAACCCCCGTTTTAACCATGCCTTTCACCTTAAAAATGGCCGCACTGGCTGTAACAATTATAGGACTACTCACAGCCTTAGAACTAGCATCTCTCACGTCCCAACAATTTAAAATCAAACCCTTATCTTCTACTCACCACTTCTCAAATATATTAGGATTTTTCCCGAGTGTAGCCCATCGACTAGTCCCAAAAACTGGCCTGATTCTTGGGCAACTGGTTGCCAATCAGACAATTGACCAAACCTGACTAGAGAAAACCGGGCCAAAAATAGTAGCCTCCGTTAACCTTCCAATGGCTACTTCAATTAGCAACCTACAGCAGGGTGTAATTAAGACCTACTTCTTATTATTTTTCTTCACCATAATACTAGCAATTCTCATCCTTGTCATCTAACTGCCCGTAAGGTCCCCCGACTTAGCCCTCGAGTTAACTCCAGAACTACAAAAAGCGTCAGTAATAAAACTCATCCCCCAAGCATTAAAACTCCTCCTCCTGAAGAATATATCAGAGCAACCCCCCCAAGATCCCCCCGGAATAGCATGAATTCACTAAACTCGTCAGCAGTTATCCATGACCCCTCATACCAACCCTCAGAGAAAAAGACAGAAATAGACGCGACCAGAAACACATATACTGACATAAGAAGCAAAACGGGTCAACTTCCCCACCCCTCAGGATAGGGCTCCGAAGCCAGCGCTGCTGAATACGCAAACACAACTAACATCCCGCCTAAATAGATCAAAAACAAAATCAGAGATAAAAATGAACCCCCGTGCCCTACTAAAATGCCACAGCCCATTCCTGCTACTGTGACAAGGCCCAAAGCAGCAAAGTAGGGTGAGGGGTTTGAGGCCACGGCCGCTAGGCCTAAAACCAGGCCAACTAATAATAAATAAGTTATATAAACCATAATTCTTGCCAGGATTTTAACCAGGGCCTGCGACTTGAAAAACCACCGTTGTACTCAACTACAAGAACCTAATGGCCAATCTTCGAAAAACCCATCCCCTATTAAAAATCGCAAACGATGCCCTGGTTGATCTCCCAGCCCCGTCAAACATTTCAGTTTGATGAAACTTCGGGTCTCTTCTAGGACTTTGTTTGGCCGCCCAGATTATTACGGGCCTTTTCCTTGCAATACATTATACATCAGACATTGCCACAGCATTTTCATCTGTAGCACATATTTGTCGTGACGTCAACTACGGCTGACTAATCCGAAACATGCATGCAAACGGTGCTTCCTTTTTCTTCATTTGCATCTACCTGCACATCGGACGGGGCTTGTATTATGGATCATACTTATATAAAGAGACATGAAATGTGGGTGTTGTCCTTCTCCTCCTAGTGATAATGACTGCTTTCGTAGGCTACGTCCTACCCTGAGGACAAATGTCATTCTGAGGGGCTACCGTCATTACCAACCTTTTATCAGCCATTCCCTACGTTGGAAACGCCCTAGTTCAATGAATCTGAGGCGGATTTTCAGTAGACAACGCCACCCTTACCCGGTTCTTTGCCTTCCATTTCCTCCTTCCCTTTGTGATTGCTGCTGCTACAGTTGTGCATCTTATCTTCCTCCACGAGACAGGATCGAATAATCCAACAGGTTTAAACTCAGACTCTGACAAAGTATCTTTTCACCCCTACTTTTCTTATAAAGATCTTCTAGGCTTTGCTGCCCTACTAGTAGCCCTTATCTCTTTAGCCCTCTTCTCCCCAAATCTACTGGGAGACCCCGATAACTTTACCCCTGCTAATCCTTTAGTGACGCCACCTCACATCAAACCTGAGTGATACTTCTTGTTCGCTTACGCCATTCTACGATCCATCCCAAACAAACTTGGCGGGGTTCTAGCCCTATTAGCCTCTATTCTAGTCCTCTTTCTTGTTCCCATTCTGCACACATCAAAACAGCGAAGCCTAACATTCCGACCCCTAACCCAATTCCTCTTCTGATTGCTAGTCGCCGATGTAATAATTTTAACCTGAATTGGAGGTATGCCTGTAGAACATCCTTACATTATCATTGGACAAATCGCATCCTTCATTTATTTTTCCCTTTTCCTAGTCATAGCGCCTATGGCCGGCCTACTAGAAAACAAAGTCTTAAAATGACAATGCATTAGAAGCTCAGATGAAAGAGCACCGGTCTTGTAAACCAGAGGTCGAAGGTTCAAGCCCTTCCTAGTGCTCAGAGAGAAGGGATTCTAACCCCTGCCCCTGACTCCCAAAGCCAGGATTCTTAACTAAACTACTCCCTGATTTTCATACACCAGTTTTGTAATCCAGAGCGCATCACTTTTGCCACCAACGTTAAATTAACGTTGCACAAACGTTGCATCAGCGCCCCATGGACACTAAATGACGCGAGGGCGCTAAATAAACACCCCCTACCTCTAGCACCCTTTTAACGATTTCACTTTTTTTTTTTTTTTTGTTTAACGATTACGTTTTTTTTTGCGTTCCCGGACTCTGCCAGATTTCGACCGAAGACTGCCAGAATCCGCTCAAAATCCGCTCAAATACCAATATGTATTATCCCCATAAATGGTTTAAACCATTTTTGCCTAGTACACACTGACCATGCAAGTCAATTATATTTACCCCGCGCTCCAGGCCGCAGTACATACACCTACAGTTGGTGTATTTAGCACAAGTGTGCCTCAGCTAGTTTCAAGTCACCCACATCCTTCCTTTAATTGTTACTTAATGTAGTAAGAGCCCACCATCAGTTGATTCCTTAATGTCAACGGTTCTTGAAGGTGAGGGACAAAAATCGTGGGGGTTTCACTTCTTGAATTATTCCTGGCATTTGGCTCTACATCTCAAGGCCATACATTTCTCGTCTCTCACACTTTCACTGGCCCTGACATTGGTTAATGGTGGAGTACATACTCCTCGTTACCCCCCATGCCGGGCGTTCTTTCTAATGGACAACGGGTTTTCCTTTTTTTTTCCTTTTCACTTGGCATTTCACAGTGCATACAAACCTTGATGACAAGGTTGAACATTTAGAAATCGGCCGCAAAGAATATTGGTGAATTATTCAAAGATATTAACAGATGAATTGCATAAGTGATATCAAGAGCATAAATAACCAAATGAAACTAGGAACGTTTCTATAATATGCCCCCCGGCTCCCGCGCGTCAAACCCCCCTACCCCCCTAAACTAGTAAGAAGTCTATTATTCCTGCAAACCCCCCGGAAACAGGAAACCCCCTACTAGCATTTTAGCCCGCCCAAATTTGTGTGTATTTATATTATTTGTAATATTGCAAAA

>GQ 14

GCTAGTGTAGCTTAACTAAAGCATAACACTGAAGATGTTAAGACAAACCTTAGATTGGTTTCACGAGCACAAAAGTTTGGTCCTGACTTTACTATCAACTTTAGCTAAACTTACACATGCAAGTATCCGCAATCCCGTGAGAATGCCCTACAGTTTCCTTAAAGGAAACAAGGAGCTGGTATCAGGCTCGATTACTCCCGCCCATGACACCTTGCTTAGCCACACCCCCAAGGGAACTCAGCAGTGATAGACATTAAGCAATAAGTGAAAACTTGACTTAATTAAAGCTAAGAGAACCGGTTAAACTCGTGCCAGCCACCGCGGTTATACGAGCGGTTCGAGCTGATAGATTACGGCGTAAAGCGTGGTTAATAAGAATGAAACTAAAGTCGAATGTTTTCAAAGCTGTTATACGCACTCGAAAATTAGAAGGTCAGAAACGAAAGTGACTTTAACCCTATGAACCCACGAAAACTATGAAACAAACTGGGATTAGATACCCCACTATGCATAGCTGTAAACTTTGATGAGCTATTACATTATCATCCGCCTGGGTACTACGAGCATCAGCTTAAAACCCAAAGGACTTGGCGGTGCTTTAGACCCACCTAGAGGAGCCTGTTCTAGAACCGATAACCCCCGTTAAACCTCACCCTCTCTTGTTTTTCCCGCCTATATACCGCCGTCGTCAGCTTACCCTGTGAAGGTCTAATAGTAAGCACAACCAGTTATACTCAAAACGTCAGGTCGAGGTGTAGCATATGAGAGGGGAAGAAATGGGCTACATTCCTTGTTTCAAGGAAAACGGATAACATAATGAAAGGTACGTTAGAAGGAGGATTTAGCAGTAAGCAGCAAATAGAGTGTTCTGCTGAAACTGGCCCTGAAGCGCGCACACACCGCCCGTCACTCTCCCCAACTCCGAGTTAAAAACATATATAAACCTTTGAAGGAACAAAGGGGAGGCAAGTCGTAACATGGTAAGTGTACCGGAAGGTGCACTTGGATAAATCAGAGTATAGCTAAGAAAGAAAAGCATCTCCCTTACACCGAGAAGTCATCCGTGCAAATCGGATTACCCTGACTCTAACAAGCTAGCCCAAAACCTTAACTTAAAAATCAAATATTTCTAGTAATTAATAAACCAAACACATTAAATAAATCATTTTTCCCCCTGAGTATGGGAGACAGAAAAGGATAAAGGAGCTATAGACAAAGTACCGCAAGGGAAAGCTGAAAGAGAAATGAAACAAACCAGTAAAGAAAAACAAAGCAGAGATTAACCCTTGTACCTTTTGCATCATGAATTAGCCAGTTTAATCAAGCAAAGAGCACTGTAGTTTGAAACCCCGAAACTTAGTGAGCTACTTCAAGACAGCCTATAAAATAGGGCAAACCCGTCTCTGTGGCAAAAGAGTGGGAAGATCTTCAAGTAGAGGTGACAGACCTATCGAGCTAAGTTATAGCTGGTTGCTCGTGAAATGAATAGAAGTTCAGCCTTTTGCTTTCTAAATTTCGATTTAGCATCACTTAGCCTAAATGATTAGAAAACAAAAGAGTTAGTCAAAGAGGGTACAGCCTGTTTGATAAAAGATACAACTTTACTAGGAGGATAAGAATCATAATTTTAAAGGTTTAATGCCCAGGTGGGCCTAAAAGCAGCCACCCTAATCAATAGCGTTAAAGCTTAAGCATAAAACACGCCTACAATTCTGATAAATCAGTTTTAATCCCCTAAAGTTAACGAGCTATTTCATACCTTATGAAAGAAATTATGCTAGTATGAGTAATAAGAAGTTACGAACTTCTCCCTGCACACGTGTAAATCGGAACGGACAAACCACCGAGTCTTAACGGCCCCAGTCAAAGAGGGGATGTCGGATAAAAAAAAGAACAAGAAATTCCCGATAAAACCACCGTTAACCCCACACCGGAGTGCTCCCTGGGAAAGACAAAAAGGGACAGAAGGAACTCGGCAAATATGCTCAAGCCTCGCCTGTTTACCAAAAACATCGCCTCTTGTAAAAGTTAAATAAGAGGTACCGCCTGCCCTGTGACTAGTAGTTTAACGGCCGCGGTATTTTGACCGTGCAAAGGTAGCGCAATCACTTGCCTTTTAAATGAAGGCCTGTATGAATGGCACGACGAGGGCTTAACTGTCTCCTCTCCCTAGTCAATGAAATTGATCCCCCCGTGCAGAAGCGGGGATAATAACATAAGACGAGAAGACCCTGTGGAGCTTTAGACTATAAGCAGACCATGTCAATAATAACAAACAAGTAAATTAAACAAATTGGCCCCTGCTTCTCTGTCTTTGGTTGGGGCGACCGCGGGATAATAAAAAACTCCCACGAGGATTGAGAACCCTTATCTTATAACCAAGAGCTTCTCCTCTAAGTAACAGAACATCTGACCTTAATGATCCGGCCTGGCCGATCAACGGACCGAGTTACCCCAGGGATAACAGCGCAATCCTCTTTTAGAGTCCATATCGACAAGAGGGTTTACGACCTCGATGTTGGATCAGGACATCCTAATGGTGCAGCCGCTATTAAGGGTTTGTTTGTTCAACAATTAAAGTCCTACGTGATCTGAGTTCAGACCGGAGTAATCCAGGTCAGTTTCTATCTATGACGTACTCTCTTCTAGTACGAAAGGACCGAAGAAAGAAGGCCCATGAAAAATTATGCCTTAGTCTCACCTTATGAAGAAAACTAAATAAGACAAGAGGTTACACCCCTTGGTCATAGAAAATGACATGTTAAGGTGGCAGAGCCCGGATATTGCAAAAGACCTAAGCCCTTTCCACAGAGGTTCAATTCCTCTCCTTAACTATGTTCTCAACAATATTAAGCTTCATTATTAATCCCCTGATTGTTATGATTTTTGTTTTGTTGGCAGTAGCTCTCTTGACCTTGGTAGAGCGTAAAGTGCTAAGCTACATACAACTTCGTAAAGGCCCAAATGTTGTTGGCCCTTACGGCCTTTTGCAACCCTTCGCTGATGGCTTAAAACTTTTCATGAAAGAGCCCGTCCGACCCTCCACCTCCTCGCCCGCCTTGTTCTTAATTACCCCTATTTTAGCCCTTACCTTAGCCCTAACCCTCTGAGCCCCTCTTCCTATGCCTTTTCCCGTCACCGACCTAAACTTAGGCATTTTATTTATTTTAGCACTATCGAGCCTAGCAGTATATTCTATTCTTGGCTCCGGATGAGCCTCCAACTCTAAATATGCATTAATTGGTGCTCTTCGAGCGGTCGCCCAAACCATCTCTTATGAAGTAAGCTTGGGCCTTATTCTTCTTAACACAATTGTCTTTACGGGGGGTTTTACTCTTCAAACCTTCAGCACCGCACAAGAAGCCACCTGATTACTTCTACCAGCATGACCACTAGCAGCCATGTGATATATCTCCACACTCGCGGAAACTAACCGGGCCCCTTTCGACCTAACTGAAGGAGAGTCCGAGCTAGTGTCTGGCTTCAACGTAGAGTATGCCGGCGGACCTTTTGCCCTTTTTTTTCTGGCAGAATACGGTAACATTTTACTTATAAATACCCTCTCAGCAGTACTATTTCTAGGCTCTTCAACCTACCACAGCTTTCCAGAACTAACCGCGACCTTATTAATGCTTAAGGCCACCCTCCTTTCAGTCGTATTTTTATGAGTGCGAGCATCTTACCCTCGGTTCCGATACGACCAGCTAATGCATTTAATTTGAAAAAACTTTTTGCCTCTAACCCTAGCGCTAGTTATTTGACACCTTTCTCTTCCGATCACGTTGAGCGGCCTCCCTCCTCAACTTTAACTCAGGAAATGTGCCTGAAAAAGGGTCACTTTGATAGGGTGAATAATGAGGGTTAAAGCCCCTCCATCTCCTTAGAAAGAAGGGGTTTGAACCCTACCTGAAGAGATCAAAACTCTTAGTGCTTCCACTACACCACTTCCTAGTAAAGTCAGCTAATAAAAGCTTTTGGGCCCATACCCCAAATATGTTGGTTAAAATCCTTCCTTTGCTAATGAATCCTTACGTCCTTTCAATCCTACTTATAGGTTTAGGCCTCGGTACTACAGTCACATTCGCTAGCTCACACTGACTCTTAGCATGAATAGGCCTTGAAATAAATACCCTCGCCATTTTACCATTAATAGCACAACACCACCACCCCCGAGCCGTTGAAGCCACCACCAAATATTTTTTAATTCAATCGGCAGCCGCAGCAACCATCTTATTTGCCAGCTCAACTAACGCCTGACTTTCGGGCCAGTGGGACATCATAAGTATCAATCACCCTCTTCCAACCGTCATAATTATAATTGCTCTGTCCTTAAAACTAGGCTTGGCCCCTCTTCACGCCTGACTTCCCGAAGTTATTCAAGGACTAGACTTGACCACGGGCTTAATCCTCTCCACATGACAAAAACTCGCACCCTTTGCCCTTCTCGTTCAAATCTTCCCCGACACCCCCCTTCTCATCACTTCTCTAGGACTTCTTTCGATATTAATTGGGGGGTGAGGAGGTTTAAACCACACACAACTCCGCAAAGTACTCGCATATTCTTCAATCGCCCACTTAGGCTGAATAATAGTAGTTATGCAATTCTCCACCCCCCTTACAATTCTTGCTTTATCAACATACATTGTTATAACATCATCTACTTTTCTAATCTTTAAACTCCTTAAATCCACAGATATGAACAGCCTGGCAACATCTTGAGCTAAAACCCCCTCCATTACAGCCCTAGCACCTTTAGTGCTATTGTCCTTAGGCGGACTCCCTCCCCTCTCGGGCTTTATGCCAAAATGACTAATTATTCAAGAATTAACTAAGCAAGATCTAGCCCTAGTTGCAACCTTGGCCGCCCTCTCTGCGCTACTCAGCCTTTTCTTCTACCTACGCATTTGTTACTCCCTCACATTTACCTCCTCTCCTAATAATCTCATGGGAACACCCCCCTGACGGCTAGCAACAAAGCAAGTATCACTTCCCCTAGCTATAACAACCTCCCTCTCTATTCTTCTACTCCCGGTTACCCCTGCAATCTTATCAGTGGTTCTCCCTTTGTAAAGAGGCTTAGGATAGTATTAAGACCAAGGGCCTTCAAAGCCCTAAGCGGGGGTGAAAGCCCCCCAGCCTCTGTAAGACCTACGGGACACTAACCCACATCTTCTGTATGCAAAACAGACACTTTAATTAAGCTAAAGCCTTCCTAGGTGGGTAGGCCTCGATCCTACAATCTCTTAGTTAACAGCTAAGCGCCTAAACCAGCGGGCATCCATCTACCTTTCCCCCGCCTTGCCGAAAAAAAAAGGCGGGGGAAAGCCCCGGCAGGGTATTAGCCTGCTACTTAAGATTTGCAATCTAATGTGTTAACACCTCGGGGCTGGTAAGAAGAGGACTTTAACCTCTGTCTATGGGGCTACAATCCACCGCTAAACGCTCAGCCACCTTACCTGTGGCAATCACACGTTGATTTTTCTCAACTAATCACAAAGACATCGGCACCCTGTATCTAATCTTTGGTGCCTGGGCGGGAATGGTAGGGACGGCCTTAAGTCTACTCATTCGGGCAGAACTAAGTCAACCAGGCTCCCTATTAGGAGACGACCAGATCTATAACGTAATTGTAACTGCACATGCTTTCGTAATAATTTTCTTTATAGTAATGCCAATCATAATTGGGGGGTTTGGCAACTGATTAATTCCTTTAATGATTGGAGCCCCCGACATGGCCTTCCCACGGATAAATAACATAAGCTTTTGACTCCTGCCCCCTTCTTTCCTTCTATTATTGGCCTCATCTGGTGTAGAAGCTGGTGCCGGAACAGGATGAACCGTATATCCCCCCTTGTCAGGTAATTTGGCACACGCAGGGGCCTCCGTAGATTTAACCATTTTCTCTCTTCACCTGGCCGGAATTTCTTCTATCCTAGGGGCCATTAATTTCATCACAACTATTATTAATATAAAACCTCCAGCCATTTCCCAATATCAAACCCCTTTATTTGTGTGAGCTGTACTAATCACCGCAGTATTACTTCTACTCTCTCTTCCTGTTCTAGCTGCAGGTATCACGATGCTTCTCACAGATCGGAACCTAAATACAACATTTTTCGACCCCGCAGGAGGGGGGGACCCCATTCTTTATCAACATTTATTCTGATTCTTTGGACATCCTGAAGTCTACATTCTAATTTTGCCCGGCTTCGGAATGATTTCTCACATTGTAGCATATTACTCAGGCAAAAAAGAGCCGTTTGGTTACATGGGAATAGTATGAGCTATAATAGCAATTGGCTTGCTGGGGTTTATCGTATGAGCCCATCATATGTTCACTGTGGGGATGGACGTGGACACTCGAGCTTATTTTACTTCCGCCACTATAATTATCGCAATTCCCACAGGAGTCAAAGTGTTTAGTTGACTAGCTACCTTGCATGGGGGTTCAATCAAATGAGAAACCCCTCTGTTATGAGCTCTGGGCTTTATCTTCTTATTTACTGTCGGAGGTTTAACAGGAATTGTTTTAGCCAACTCATCTCTGGACATTATACTTCATGATACATACTATGTTGTAGCCCACTTCCACTATGTCCTCTCTATAGGAGCAGTCTTTGCCATCATGGGAGCATTCGTTCACTGATTCCCCCTATTCTCAGGCTACACCCTTCACAATACGTGAACAAAAATCCACTTCGGAGTTATGTTTGTAGGTGTAAACCTCACCTTTTTCCCTCAGCACTTCTTAGGGTTGGCGGGAATACCTCGACGATATTCAGATTACCCTGACGCATACACACTGTGAAATACTATCTCATCCCTGGGGTCATTAATCTCCCTTATTGCTGTAATTATATTCCTATTTATTATCTGGGAAGCATTCGCGGCAAAACGTGAAGTCTTATCAGTAGAACTAACAGCCACAAACGTAGAATGACTGCACGGGTGTCCTCCCCCTTACCATACATTTGAAGAACCTGCATTCGTTCAAATTCAACAATCCAAATTTTAATCGAGAAAGGAAGGAGTCGAACCCCCATAAACTGGTTTCAAGCCAGCCACATAACCGCTCTGTCACTTTCTTCCCTAAGTTAATAAGATTCTAGTTAAAAGAATAACACTGCCTTGTCAAGGCAAAATTGTGGGTTAAAGCCCCACGTATCTTGCTTATGGCACATCCATCTCAACTAGGATTCCAAGATGCAGCTTCACCCGTTATAGAAGAACTTCTCCATTTTCATGACCATGCATTAATAATTGTTTTCTTAATCAGCACCCTTGTTCTTTACATTATTGTGGCTATGGTAACCACCAAGCTAACAAATAAGTTCATTCTGGACTCCCAAGAAATTGAAATCATCTGAACCTTACTACCAGCAATTATCCTAATTCTGATCGCCCTGCCCTCCCTTCGCATTCTCTACCTCATGGATGAAATCAATGACCCCCACCTCACAATTAAAGCCATAGGACATCAATGATACTGAAGCTACGAATATACGGATTATGAGGACCTAGGGTTCGACTCATATATGGTCCCTACACAAGACCTCGCCCCTGGTCAATTTCGACTACTTGAGACAGACCATCGCATGGTCATTCCTGTTGAGTCCCCCATCCGGGTTCTTGTCTCCGCCGAGGATGTTTTACACTCATGGGCCGTCCCAAGCCTCGGAGTAAAAATGGACGCCGTCCCCGGCCGCCTAAATCAAACAGCCTTCATTACTTCCCGTCCGGGTGTGTTTTATGGACAATGCTCAGAAATTTGCGGAGCTAATCATAGCTTTATGCCCATTGTAGTGGAAGCTGTTCCTCTAGAACACTTCGAGAACTGATCTTACCTAATACTTCAAGATGCCTCACCAGGAAGCTAAAAGGGAATAGCATTAGCCTTTTAAGCTAAAAATTGGTGACTCCCGCCCACCCCTGGTGACATGCCTCAGTTGAACCCCGCACCCTGATTTGCTATTATAGTATTCTCGTGACTAGTTTTCCTAGCCGTTATCCCACCTAAAGTTCTAGCTCACCATTTTCCCAATGACCCCGCCCCACAAAGCGTAAAAAAATCAAAAACAGAGACCTGATCCTGACCATGACTTTAAGCCTCTTTGATCAATTTATGAGCCCTACACTTCTAGGGGTACCTCTTATCGGACTCGCCCTAACATTGCCATGAGTCCTTTACTTCCAACCCGGTGCCCGATGACTTAATAACCGCTTGATTACCCTTCAGTCTATATTCATGAACTGATTTGTAAAACAAATCTTTCAGCCAATAAGCTTAGGCGGACACAAATGGGCCGCTCTCCTTATATCTTTAATACTATTTTTAATCACCTTAAATATGCTAGGCCTGCTACCTTACACATTTACTCCAACAACGCAGCTGTCACTTAATATAGCCTTTGCAGTACCACTTTGACTAGCAACTGTCATTATTGGAATACGAAATCAGCCAACACATGCCCTTGGTCACCTCCTCCCCGAAGGAACTCCTACCGCCCTAATCCCGGTTTTAATCGTGATTGAGACAATTAGCCTTTTTATTCGACCATTGGCCCTCGGTGTTCGACTTACCGCAAACTTGACAGCCGGGCACCTTCTAATTCAACTAATTGCAACTGCGGCTTTTGTTCTTTTCCCTATAATGCCTACAGTCGCTGCTCTTACCTCTGTCTTACTATTCTTGCTAACCCTACTAGAAGTCGCCGTGGCCATAATCCAAGCCTATGTATTCGTACTTCTTTTAAGCCTTTACCTACAAGAAAACGTCTAATGGCCCATCAAGCACATGCATATCATATAGTTGACCCAAGCCCTTGACCCCTCACAGGCGCAGTAGCCGCCCTTCTACTTACATCTGGAACAGCAATCTGAATACACTTTAACTCCACAGTTCTCATGTCCCTTGGACTTGTCCTGTTACTACTAACCATATATCAATGATGGCGAGACATTATCCGAGAGGGTACCTTTCAAGGTCATCATACACCCCCTGTTCAAAAAGGCCTTCGGTACGGGATAATTCTATTTATTACCTCAGAGGTCTTCTTTTTCCTAGGTTTCTTCTGAGCATTTTATCACTCAAGCCTAGCCCCAACCCCCGAACTTGGTGGATGTTGACCACCCATGGGTATTACAACACTGGACCCCTTTGAAGTTCCCCTTCTCAATACTGCTGTCCTTCTCGCCTCCGGTGTCACAGTCACTTGAGCTCACCATAGTATTATGGAGGGGCAGCGAAAACAAGCAATTCAGTCCTTGACACTCACAATTCTCCTGGGGTTTTACTTTACATTCCTTCAAGCAATAGAGTACTACGAGGCACCCTTCACCATTGCAGATGGCGTCTATGGCTCTACATTTTTTGTGGCAACAGGGTTTCATGGCCTCCATGTAATTATTGGATCAACATTTCTGGCAGTCTGCCTCTTACGACAAGTCCAGTTCCATTTTACATCAGAACATCACTTCGGATTTGAAGCTGCAGCATGATACTGACACTTTGTAGACGTAGTCTGACTATTCTTATATATCTCTATCTACTGATGAGGCTCATATCTTTCTAGTATTAAAAAGTACAAGTGACTTCCAATCACTTAGTCTTGGTTAAACTCCAAGGAAAGATAATGAACTTAGTACTAGTCATTATTTGCATCTCATTAGCCCTCGCCACACTGCTCGCAACTGTTTCATTTTTCCTCCCACAAATAACACCTGATTATGAGAAACTCTCACCGTATGAGTGCGGCTTTGATCCAGTGGGGTCCGCCCGTTTGCCATTCTCCATTCGCTTTTTTCTAGTCGCAATCCTATTTCTCCTCTTCGACTTAGAAATTGCCTTACTTCTTCCCCTTCCCTGAGGAGACCAACTTCCCTCCCCTCTGACAACTTTCTTTTGAGCTTCTGCTATTCTTATACTACTAACTCTAGGGTTAATCTATGAATGACTTCAAGGAGGCCTAGAGTGGGCAGAATAGGTACTTAGTTTAATAAAAACATTTGATTTCGGCTCAAAAACTTATGGTTTAAGTCCATATTTACCTGATGACCTTAACTCACTATGCATTCTCGTCAGCCTACTTTGTTAGCTTCATGGGTTTAATTTTTTACCGAAAGCATCTTCTCTCCGCCTTACTTTGCTTAGAAGCGATAATACTTATTCTTTTTATTTCACTATGCCTGTGAGGCCTAGTCTTAGCCTCAAGTGCATTTTCGGCAGGCCCAATGATCTTACTTGCTTTCTCAGCATGTGAAGCAAGTGCAGGCCTAGCACTGCTTGTAGCAATAGCTCGAACCCACGGTACTGACCGTTTAAAAAACCTTAGCCTACTCCAATGTTAATAATTCTTATTCCTACTGTTATGCTTCTACCCACAATCTGACTGAGCCCCACTAAATACCTGTGATCCTCAACACTTGGCCATAGCATAATAATTGCTCTTATAAGCCTCTCCTGACTTAGCCTCCCAGGGGAGGTTGGCTGATCTTCCCTTAACACTTTTATAGCAACAGACCCTCTCTCTACCCCCCTTCTCGTACTTACTTGCTGACTTCTGCCCTTAATAATTCTTGCGAGCCAAAACCATATAGCCCAAGAACCTACCAATCGCCAGCGAACCTATATCTCTCTCCTTACTTCCCTTCAAATCTTCTTAATCTTAGCATTTGGGGCAACCGAGATAATCATGTTCTACATTATATTTGAAGCGACCTTAATTCCCACACTTGTAATTATCACACGATGAGGAAACCAAACAGAGCGATTAAACGCAGGTATTTACTTTTTATTTTATACCTTAGCCGGCTCTTTACCACTACTAGTGGCCCTCCTTCTACTTCAGACCTCGACAGGAACTCTTTCTTTTCTAACCACTCAATTTTTTCCCCCTTTACAACTGCATACAGAAGCAAGTAAATTCTGGTGGGCAGGCTGTTTACTAGCATTCTTAGTAAAAATGCCCTTATATGGAGCACACCTTTGACTTCCAAAAGCTCACGTCGAAGCCCCCATCGCCGGGTCAATAGTCCTTGCAGCCGTTCTTTTAAAACTAGGGGGTTACGGTATGATACGAGTCATTATTATCTTAGAACCCCTCACGAAACAACTCAGCTACCCCTTTATTATTCTTGCCCTGTGGGGCGTCGTAATAACTGGCTCAATTTGCCTCCGACAAACAGACCTTAAATCACTAATCGCTTACTCCTCAGTAAGCCACATAGGCCTTGTCGCAGCAGGCATCCTAATCCAAACTCCTTGGGGGTTTACAGGAGCATTAATCCTTATAATTGCCCATGGCTTAACTTCCTCCGCCCTATTCTGTTTAGCCAACACTAACTATGAGCGAACACATAGCCGAACCATGCTTTTAGCCCGGGGTCTACAAATGGTCCTTCCTCTCTTAGCAACTTGATGGTTTCTATTTACCCTCGCCAACCTAGCACTCCCTCCGCTACCCAACCTCATAGGAGAACTTATGATTATCTCATCCTTGTATAACTGGTCAAACTGGTCTCTAATCCTGACCGGGGCGGGAGTACTAATTACCGCTAGTTACTCTCTCCATATATTCCTAACCACTCAACGTGGCCCTATTACTAACCCCGTCTTAGCAATTGAACCAACCCACACACGAGAACATCTCCTCATAATCCTTCACCTTCTTCCCCTCCTCCTTCTAATTTTAAAACCCTGCTTGATCTGGGGCTGAACAGTTTGTAGGCGTAGTTTAAATAAAGCGCTAGATTGTGGTTCTAGAAATAAGAGTTAAACCCTCTTCACCCACCGAGAGGGGTCGCCGTGACAGCAAGAACTGCTAATTCTAGCCCCTTTGGTTAAAGTCCGAAGCCCACTCGAACAGGCTTCTAAAGGATAACAGCTCATCCGTTGGTCTTAGGAACCAAAAACTCTTGGTGCAACTCCAAGTAGCAGCTATGCACTTTACAACAATGATCCTCTCCTCAAGCCTAATAACAATTTTCCTTCTTCTTATCCTTCCAATCCTAGGTACACTAAACCCTAGCCCCCCGGGGGACCTATGAGCCACAAAAAACGTTAAAACAGCAGTTAAGATGGCCTTTTTTGTAAGTCTTCTACCTCTTTTTATCTTTCTTAATGAAGGAGTAGAGACTATTATAACAAACTGAAAATGAATAAATACTCTAATATTTGAAATTAATATCAGCTTTAAATTTGACCTCTACTCCGTGGTATTTACCCCTGTAGCCCTCTACGTAACATGATCAATTTTAGAGTTCGCATCTTGGTACATACACAGTGACCCCAATATAAACCGGTTCTTTAAGTATCTTTTGATCTTTCTAATCGCTATGGTTGTTCTGGTTACAGCCAACAACATGTTCCAACTATTTATTGGCTGAGAAGGTGTTGGAATTATGTCTTTCTTACTTATTGGCTGGTGGTTCGGGCGGGCTGACGCCAACACTGCGGCCCTCCAGGCCGTAGTTTATAACCGAGTTGGTGATATCGGCCTAATTCTAGCAATAGCATGAATAGTAGTAAACCTAAACTCATGAGAGATACAACAGCTCTTTTCTGTGTCTAAAGACCATGATATGACCCTTCCCTTATTAGGCCTAGTACTGGCCGCTACCGGAAAGTCCGCCCAGTTTGGACTTCACCCCTGGCTCCCCTCAGCCATAGAGGGTCCAACACCGGTCTCTGCCCTCCTGCACTCTAGCACCATGGTTGTTGCTGGTATTTTCCTTCTTATCCGCCTCAGCCCCTTAATGCAAGAAAGCCCGTTAATTCTCTCAACATGCCTTTGCCTGGGGGCCCTAACTACCGTCTTTACTGCGACATGTGCCCTTACCCAAAATGACATCAAAAAAATTGTTGCATTTTCTACATCAAGTCAATTAGGACTAATAATAGTTACCATCGGACTAGGCCAGCCCCAGCTCGCCTTCCTTCATATCTGCACCCACGCCTTCTTTAAAGCAATACTTTTCTTATGTTCAGGCTCCATCATTCATAGCCTTAATGATGAGCAAGATATCCGAAAAATAGGAGGACTTCACAAGCTCCTTCCACTGACCTCTTCTTGTCTAACCATTGGCAGCCTAGCTCTAACAGGAGTCCCCTTTTTAGCAGGCTTCTTTTCCAAAGACGCCATCATTGAAGCTATAAATACATCCTACCTTAACGCCTGAGCCCTAATTTTAACGCTTCTAGCTACATCATTTACCGCAGTTTACAGTCTCCGAGTCGTATTCTTTGCCTCTATAGGCCACCCGCGTTTTAATCCAATCTCCCCCATTAATGAAAATAACCCTACAGTGATAAACCCTATTAAACGACTCGCTTGGGGAAGCATTTTGGCAGGGTTGCTAATTACGGCCAATATTGTTCCACTTAAAACCCCCGTTTTAACCATGCCTTTCACCTTAAAAATGGCCGCACTGGCTGTTACAATTATAGGACTACTCACAGCCTTAGAACTAGCATCTCTCACGTCCCAACAATTTAAAATCAAACCCTTATCTTCTACTCACCACTTCTCAAATATATTAGGATTTTTCCCCAGTGTAGCCCATCGACTAGTCCCAAAAACTGGCCTGATTCTTGGACAACTAGTTGCCAATCAGACAATTGACCAAACCTGACTAGAGAAGACCGGGCCAAAAATAGTAGCCTCCGTTAACCTTCCAATGGCTACTTCAATTAGCAACCTACAGCAAGGTGTAATTAAGACCTACTTCTTATTATTTTTCTTCACCATAATACTGGCAATTCTCATCCTTGTCATCTAACTGCCCGTAAGGTCCCCCGACTTAGCCCTCGAGTTAACTCCAGAACTACAAAAAGCGTCAGTAATAAAACTCATCCCCCAAGCATTAAAACTCCTCCTCCTGAAGAATATATCAGAGCAACCCCACCAAGATCCCCCCGGAATAGCATGAATTCACTAAACTCGTCAGCAGTTATCCATGACCCCTCATACCAACCCTCAGAGAAAAAGACAGAGATAGACGCGACCAGAAACACATATACTGACATAAGAAGCAAAACCGGTCAACTTCCCCACCCCTCAGGATAAGGCTCCGAAGCCAGCGCTGCTGAATACGCAAACACAACTAACATCCCACCTAAATAGATCAAAAACAAAATCAAAGATAAAAATGAACCCCCGTGCCCTACTAAAATGCCACAGCCCATTCCTGCTACTGTGACAAGGCCCAAGGCAGCAAAGTAGGGTGAGGGGTTCGAGGCCACGGCCGCTAGACCTAAAACCAGACCAACTAGTAATAAATAAGTTATATAAACCATAATTCTTGCCAGGATTTTAACCAGGGCCTGCGACTTGAAAAACCACCGTTGTACTCAACTACAAGAACCTAATGGCCAATCTTCGAAAAACCCATCCCCTATTAAAAATCGCAAACGATGCCCTGGTTGATCTCCCAGCCCCATCGAACATTTCAGTTTGATGAAACTTCGGGTCTCTTCTAGGACTTTGTTTGGCCGCCCAGATTGTTACGGGCCTTTTCCTTGCAATACATTATACATCAGACATTGCCACAGCATTTTCATCTGTAGCACATATTTGTCGTGACGTCAACTACGGCTGACTAATCCGAAACATGCATGCAAACGGTGCTTCCTTTTTCTTCATTTGCATCTACCTGCACATCGGACGGGGCTTGTATTATGGATCATACTTATATAAAGAGACATGAAATGTGGGTGTTATCCTTCTCCTCCTAGTGATAATGACTGCTTTCGTAGGCTACGTCCTACCCTGAGGACAAATGTCATTCTGAGGGGCTACCGTCATTACCAACCTTTTATCAGCCATTCCCTACGTTGGAAACGCCCTAGTTCAATGAATCTGAGGCGGATTTTCAGTAGACAACGCCACCCTTACCCGGTTCTTTGCCTTCCATTTCCTCCTTCCCTTTGTAATTGCTGCTGCTACAGTTGTTCATCTTATCTTCCTGCACGAGACAGGATCGAATAATCCAACGGGTTTAAACTCAGACTCTGACAAAGTATCTTTTCACCCCTACTTTTCTTATAAAGATCTTCTAGGATTTGCTGCCCTACTAGTAGCCCTTATCTCTTTAGCCCTCTTCTCCCCAAATCTACTCGGAGACCCCGATAACTTTACCCCTGCTAATCCTTTAGTGACTCCACCTCACATCAAGCCTGAGTGATACTTCCTGTTCGCTTACGCCATTCTACGATCCATCCCAAACAAACTTGGCGGAGTTCTAGCCCTATTAGCCTCTATTCTAGTCCTCTTTCTTGTTCCCATTCTGCACACATCGAAACAACGAAGCCTAACATTCCGACCCCTGACCCAATTCCTCTTCTGATTGCTAGTCGCCGATGTAATAATTTTAACCTGAATTGGAGGCATGCCTGTAGAACACCCTTACATTATCATTGGACAAATCGCATCCTTCATTTATTTTTCCCTTTTCCTAGTCATAGCGCCTATGGCCGGCCTACTAGAAAACAAAGTCTTAAAATGACAATGCATTAGAAGCTCAGATGAAAGAGCACCGGTCTTGTAAACCAGAGGTCGAAGGTTCAAGCCCTTCCTAGTGCTCAGAGAGAAGGGATTCTAACCCCTGCCCCTGACTCCCAAAGCCAGGATTCTTAGCTAAACTACTCCCTGATTTTCATACACCAGTTTTGCAATCCAGAGCGCATCACTTTTGCCACCAACGTTAAATTAACGTTGCACAAACGTTGCATCAGCGCCCCATGGACACTAAATGACGCGAGGGCGCTAAATAAACACCCCCTACCTCTAGCACCCTTTTAACGATTTCACTTTTTTTTTTTTTTTTGTTTAACGATTACGTTTTTTTTTGCGTTCCCGGACTCTGCCAGATTTCGACCGAAGACTGCCAGAATCCGCTCAAAATCCGCTCAAATACCAATATGTATTATCCCCATAAATGGTTTAAACCATTTTTGCCTAGTACACGCTGACCATGCAAGTCAATTATATTTACCCCGCGCTCCAGGCCGCAGTACATACACCTACAGTTGGTGTATTTAGCACAAGTGTGCCTCAGCTAGTTTCAAGTCACCCACATCCTTCCTTTAATTGTTACTTAATGTAGTAAGAGCCCACCATCAGTTGATTCCTTAATGTCAACGGTTCTTGAAGGTGAGGGACAAAAATCGTGGGGGTTTCACTTCTTGAATTATTCCTGGCATTTGGCTCTACATCTCAAGGCCATACATTTCTCGTCTCTCACACTTTCACTGGCCCTGACATTGGTTAATGGTGGAGTACATACTCCTCGTTACCCCCCATGCCGGGCGTTCTTTCTAATGGACAACGGGTTTTCCTTTTTTTTTCCTTTTCACTTGGCATTTCACAGTGCATACAGACCTTGATGACAAGGTTGAACATTTAGAAATCGGCCGCAAAGAATATTGGTGAGTTATTTAAAGATATTAACAGATGAATTGCATAAGTGATATCAAGAGCATAAATAACCAAATGAAACTAGGAACGTTTCTATAATATGCCCCCCGGCTTCCGCGCGTCAAACCCCCCTACCCCCCTAAACTAGTAAGAAGTCTATTATTCCTGCAAACCCCCCGGAAACAGGAAACCCCCTACTAGCATTTTAGCCCGCCCAAATTTGTGTGTATTTATATTATTTGTAATATTGCAAAA

>GQ 17

GCTAGTGTAGCTTAACTAAAGCATAACACTGAAGATGTTAAGACAAACCTTAGATTGGTTTCACGAGCACAAAAGTTTGGTCCTGACTTTACTATCAACTTTAGCTAAACTTACACATGCAAGTATCCGCAATCCCGTGAGAATGCCCTACAGTTTCCTTAAAGGAAACAAGGAGCTGGTATCAGGCTCAATTACTCCCGCCCATGACACCTTGCTTAGCCACACCCCCAAGGGAACTCAGCAGTGATAAACATTAAGCAATAAGTGAAAACTTGACTTAATTAAAGCTAAGAGAACCGGTTAAACTCGTGCCAGCCACCGCGGTTATACGAGCGGTTCGAGCTGATAGATTACGGCGTAAAGCGTGGTTAATAAGAATGAAACTAAAGTCGAATGTTTTCAAAGCTGTTATACGCACTCGAAAATTAGAAGGTCAGAAACGAAAGTGACTTTAACCCTATGAACCCACGAAAACTATGAAACAAACTGGGATTAGATACCCCACTATGCATAGCTGTAAACTTTGATGAGCTATTACATTATCATCCGCCTGGGTACTACGAGCATCAGCTTAAAACCCAAAGGACTTGGCGGTGCTTTAGACCCACCTAGAGGAGCCTGTTCTAGAACCGATAACCCCCGTTAAACCTCACCCTCTCTTGTTTTTCCCGCCTATATACCGCCGTCGTCAGCTTACCCTGTGAAGGTCTAATAGTAAGCACAACCAGTTATACTCAAAACGTCAGGTCGAGGTGTAGCATATGAGAGGGGAAGAAATGGGCTACATTCCTTGTTTCAAGGAAAACGGATAACATAATGAAAGGTACGTTAGAAGGAGGATTTAGCAGTAAGCAGCAAATAGAGTGTTCTGCTGAAACTGGCCCTGAAGCGCGCACACACCGCCCGTCACTCTCCCCAACTCCGAGTTAAAAACATATATAAACCTTTGAAGGAACAAAGGGGAGGCAAGTCGTAACATGGTAAGTGTACCGGAAGGTGCACTTGGATAAATCAGAGTATAGCTAAGAAAGAAAAGCATCTCCCTTACACCGAGAAGTCATCCGTGCAAATCGGATTACCCTGACTCTAACAAGCTAGCCCAAAACCTTAACTTAAAAATCAAATATTTCTAGTAATTAATAAACCAAACACATCAAATAAATCATTTTTCCCCCTGAGTATGGGAGACAGAAAAGGATAAAGGAGCTATAGACAAAGTACCGCAAGGGAAAGCTGAAAGAGAAATGAAACAAACCAGTAAAGAAAAACAAAGCAGAGATTAACCCTTGTACCTTTTGCATCATGAATTAGCCAGTTTAATCAAGCAAAGAGCACTGTAGTTTGAAACCCCGAAACTTAGTGAGCTACTTCAAGACAGCCTATGAAATAGGGCAAACCCGTCTCTGTGGCAAAAGAGTGGGAAGATCTTCAAGTAGAGGTGACAGACCTATCGAACTAAGTTATAGCTGGTTGCTCGTGAAATGAATAGAAGTTCAGCCTTTTGCTTTCTAAATTTCGATTTAGCATCACTTAGCCTAAATGACTAGAAAACAAAAGAGTTAGTCAAAGAGGGTACAGCCTGTTTGATAAAAGATACAACTTTACTAGGAGGATAAGAATCATAATTTTAAAGGTTTAATGCCCAGGTGGGCCTAAAAGCAGCCACCCTGATCAATAGCGTTAAAGCTTAAGCATAAAACACACCTACAATTCTGATAAATCAGTTTTAATCCCCTAAAGTTAACGAGCTATTTCATACCTTATGAAAGAAATTATGCTAGTATGAGTAATAAGAAGTTACAAACTTCTCCCTGCACACGTGTAAATCGGAACGGACAAACCACCGAATCTTAACGGCCCCAGTCAAAGAGGGGATGTCGGATAAAAAAAAGAACAAGAAATTCCCGATAAAACCACCGTTAACCCCACACCGGAGTGCTCCCTGGGAAAGACAAAAAGGGACAGAAGGAACTCGGCAAATATGCTCAAGCCTCGCCTGTTTACCAAAAACATCGCCTCTTGTAAAAGTTAAATAAGAGGTACCGCCTGCCCTGTGACTAGTAGTTTAACGGCCGCGGTATTTTGACCGTGCAAAGGTAGCGCAATCACTTGCCTTTTAAATGAAGGCCTGTATGAATGGCACGACGAGGGCTTAACTGTCTCCTCTCCCTAGTCAATGAAATTGATCCCCCCGTGCAGAAGCGGGGATAATAACATAAGACGAGAAGACCCTGTGGAGCTTTAGACTATGAGCAGACCATGTCAAGAATAACAAACAAGTAAATTAAACAAATTGGCCCCTGCTTCCCTGTCTTTGGTTGGGGCGACCGCGGGATAATAAAAAACTCCCACGAGGATTGAGAACCCTTATCTTATAACCAAGAGCTTCTCCTCTAAGTGACAGAACATCTGACCTTAATGATCCGGCCTGGCCGATCAACGGACCGAGTTACCCCAGGGATAACAGCGCAATCCTCTTTTAGAGTCCATATCGACAAGAGGGTTTACGACCTCGATGTTGGATCAGGACATCCTAATGGTGCAGCCGCTATTAAGGGTTTGTTTGTTCAACAATTAAAGTCCTACGTGATCTGAGTTCAGACCGGAGTAATCCAGGTCAGTTTCTATCTATGACGTACTCTCTTCTAGTACGAAAGGACCGAAGAAAGAAGGCCCATGAAAAATTATGCCTTAGTCTCACCTTATGAAGAAAACTAAATAAGACAAGAGGTTACACCCTTTGGTCATAGAAAATGACATGTTAAGGTGGCAGAGCCCGGATATTGCAAAAGACCTAAGCCCTTTCCACAGAGGTTCAATTCCTCTCCTTAACTATGTTCTCAACAATATTAAGCTTCATTATTAATCCCCTGATTGTTATGATTTTTGTTTTGTTGGCAGTAGCCCTCTTGACCTTGGTAGAGCGTAAAGTGCTAAGCTACATGCAACTTCGTAAAGGCCCAAATGTTGTTGGCCCTTACGGCCTTTTGCAACCCTTCGCTGATGGCTTAAAACTTTTCATGAAAGAGCCCGTCCGACCCTCCACCTCCTCGCCCGCCTTGTTCTTAATTACCCCTATTATAGCCCTTACCTTAGCCCTAACCCTCTGAGCCCCCCTTCCTATGCCTTTTCCCGTCACCGACCTAAACTTAGGCATTTTATTTATTTTAGCACTATCGAGCCTGGCAGTATATTCTATTCTTGGCTCCGGATGGGCCTCCAACTCCAAATATGCATTAATTGGTGCTCTTCGAGCGGTCGCCCAAACCATCTCTTATGAAGTAAGCTTGGGCCTTATTCTTCTTAACACAATTGTCTTTACGGGGGGTTTTACTCTTCAAACCTTCAGCACCGCACAAGAAGCCACCTGATTACTTCTACCAGCATGACCACTAGCAGCCATGTGATATATCTCCACACTCGCGGAAACTAACCGGGCCCCTTTCGACCTAACTGAAGGAGAGTCCGAACTAGTGTCTGGCTTCAACGTAGAGTATGCCGGCGGACCTTTTGCCCTTTTTTTTCTGGCAGAATACGGTAACATTTTACTTATAAATACCCTCTCAGCAGTACTATTTCTAGGCTCTTCAACCTACCACAGCTTTCCAGAACTAACCGCGACCTTATTAATGCTTAAAGCCACCCTCCTTTCAGTCGTATTTTTATGAGTGCGAGCATCTTACCCTCGGTTCCGATACGACCAACTAATGCATTTAATTTGAAAAAACTTTTTACCTCTGACCCTAGCGCTAGTTATTTGACACCTTTCTCTTCCGATCACGTTGAGCGGCCTCCCTCCTCAACTTTAACTCAGGAAATGTGCCTGAAAAAGGGTCACTTTGATAGGGTGAATAATGAGGGTTAAAGCCCCTCCATCTCCTTAGAAAGAAGGGGTTTGAACCCTACCTGAAGAGATCAAAACTCTTAGTGCTTCCACTACACCACTTCCTAGTAAAGTCAGCTAATAAAAGCTTTTGGGCCCATACCCCAAATATGTTGGTTAAAATCCTTCCTTTGCTAATGAATCCTTACGTCCTTTCAATTCTACTTATGGGTTTAGGCCTCGGCACTACAGTCACATTCGCTAGCTCACACTGACTCTTAGCATGAATAGGCCTTGAAATAAATACCCTCGCCATTTTGCCATTAATAGCACAACATCACCACCCCCGAGCCGTTGAAGCCACCACCAAGTATTTTTTAATTCAATCGGCAGCCGCAGCAACCATCTTATTTGCCAGCTCAACTAACGCCTGACTTTCGGGCCAGTGGGACATCATAAGTATCAATCACCCCCTTCCAACCATCATAATTACAATTGCTCTGTCCTTAAAACTAGGCTTGGCCCCTCTTCACGCCTGACTTCCCGAAGTTATTCAAGGACTAGACTTGACCACGGGCTTAATCCTCTCCACATGACAAAAACTCGCACCCTTTGCCCTTCTCGTTCAAATCTTCCCCGACACCCCCCTTCTCATCACTTCTCTAGGGCTTCTTTCAATATTAATTGGGGGATGAGGAGGTTTAAACCACACACAACTCCGCAAAGTGCTCGCATATTCTTCGATCGCCCACTTAGGCTGAATAATAGTAATTATGCAATTCTCCACCCCCCTTACAATTCTTGCTTTATCAACATACATTGTTATAACATCATCTACTTTTCTAATCTTTAAACTCCTTAAATCCACAGATATGAACAGCCTGGCAACATCTTGAGCTAAAACCCCCTCCATTACAGCCCTAGCACCTTTAGTGCTATTATCCTTAGGCGGACTCCCTCCCCTCTCGGGCTTTATGCCAAAATGATTAATTATTCAAGAATTAACTAAGCAAGATCTAGCCCTAGTTGCAACCTTGGCCGCCCTCTCTGCGCTACTCAGCCTTTTCTTCTACCTACGCATTTGTTACTCCCTCACATTTACCTCCTCTCCTAATAATCTCATGGGGACGCCCCCCTGACGACTAGTAACAAAGCAAGTATCACTTCCCCTAGCTATAACAGCCTCCCTCTCTATTCTTCTACTCCCGGTTACCCCTGCAATCTTATCAGTGGTTCTCCCTTTGTAAAGAGGCTTAGGATAGTATTAAGACCAAGGGCCTTCAAAGCCCTAAGCGGGAGTGAAAGCCCCCCAGCCTCTGTAAGACCTACGGGACACTAACCCACATCTTCTGTATGCAAAACAGACACTTTAATTAAGCTAAAGCCTTCCTAGGTGGGTAGGCCTCGATCCTACAATCTCTTAGTTAACAGCTAAGCGCCTAAACCAGCGGGCATCCATCTACCTTTCCCCCGCCTTGCCGAAAAAAAAAAGGCGGGGGAAAGCCCCGGCAGGGTATTAGCCTGCCACTTAAGATTTGCAATCTAATGTGTTAACACCTCGGGGCTGGTAAGAAGAGGACTTTAACCTCTGTCCATGGGGCTACAATCCACCGCTAAACGCTCAGCCACCTTACCTGTGGCAATCACACGTTGATTTTTCTCAACTAATCACAAAGACATCGGCACCCTGTATCTAATCTTTGGTGCCTGGGCGGGAATGGTAGGGACGGCCTTAAGTCTACTCATTCGGGCAGAACTAAGTCAACCAGGCTCCCTATTAGGAGACGACCAGATCTATAACGTAATTGTAACTGCACATGCTTTCGTAATAATTTTCTTTATAGTAATGCCAATCATAATTGGGGGGTTTGGCAACTGATTAATTCCTTTAATGATTGGAGCCCCCGACATGGCCTTCCCACGGATAAATAATATAAGCTTTTGACTCCTGCCCCCTTCTTTCCTTCTATTATTGGCCTCATCTGGTGTAGAAGCTGGTGCCGGAACAGGATGAACCGTATATCCCCCCTTGTCAGGTAATTTGGCACACGCAGGGGCCTCCGTAGATTTAACCATTTTCTCTCTTCACCTGGCCGGAATTTCTTCTATCCTAGGGGCCATTAATTTCATCACAACTATTATTAATATAAAACCCCCAGCCATTTCCCAATATCAAACCCCTTTATTTGTGTGAGCTGTACTAATTACCGCAGTATTACTTCTACTCTCTCTTCCTGTTCTAGCTGCAGGTATCACCATGCTTCTCACAGATCGGAACCTAAATACAACATTTTTCGACCCCGCAGGAGGGGGGGACCCCATTCTTTATCAACATTTATTCTGATTCTTTGGGCATCCTGAAGTCTACATTCTAATTTTGCCCGGCTTCGGAATGATTTCTCACATTGTAGCATATTACTCAGGCAAAAAAGAGCCGTTTGGTTACATGGGAATAGTATGAGCTATAATAGCAATTGGCTTGCTGGGCTTTATCGTATGAGCCCATCATATGTTCACTGTGGGGATGGACGTGGACACTCGAGCTTATTTTACTTCCGCCACTATGATTATCGCAATTCCTACAGGAGTCAAAGTGTTTAGTTGACTAGCTACCTTGCATGGGGGCTCAATCAAATGAGAAACCCCTCTGTTATGAGCTCTGGGCTTTATCTTCTTATTTACTGTCGGAGGTTTAACAGGAATTGTTTTAGCCAACTCATCTCTGGACATTATACTTCATGATACATACTATGTTGTAGCCCACTTCCACTATGTCCTCTCTATAGGAGCAGTCTTTGCCATCATGGGAGCATTCGTTCACTGATTCCCCCTATTCTCAGGCTACACCCTTCACAGTACGTGAACAAAAATCCACTTCGGAGTTATGTTTGTAGGTGTAAACCTCACCTTTTTCCCTCAGCACTTCTTAGGATTGGCGGGAATACCTCGACGATACTCAGATTACCCTGACGCATACACACTGTGAAATACTATCTCATCCCTGGGGTCATTAATCTCCCTTATTGCTGTAATTATATTCCTATTTATTATCTGGGAAGCATTCGCGGCAAAACGTGAAGTCTTATCAGTTGAACTAACAGCCACAAACGTAGAATGACTGCACGGGTGTCCTCCCCCTTACCATACATTTGAAGAACCTGCATTCGTTCAAATTCAACAATCCAAATTTTAATCGAGAAAGGAAGGAGTCGAACCCCCATAAACTGGTTTCAAGCCAGCCACATAACCGCTCTGTCACTTTCTTCCCTAAGTTAATAAGATTCTAGTTAAAGGAATAACACTGCCTTGTCAAGGCAAAATTGTGGGTTAAAACCCCACGTATCTTGCTTATGGCACATCCATCTCAACTAGGATTCCAAGATGCAGCTTCACCCGTTATAGAAGAACTTCTCCATTTTCATGACCATGCATTAATAATTGTTTTCTTAATCAGCACCCTTGTTCTTTACATTATTGTGGCTATGGTAACCACCAAGCTAACAAATAAGTTCATTCTGGATTCCCAAGAAATTGAAATCATCTGAACCTTGCTACCAGCAATTATCCTAATTCTGATCGCCCTACCCTCCCTTCGCATTCTCTACCTCATGGATGAAATCAATGACCCCCACCTCACAATTAAAGCCATAGGACATCAATGATACTGAAGCTACGAATATACGGATTATGAAGACCTAGGGTTCGACTCATATATGGTCCCTACACAAGACCTCGCCCCTGGTCAATTTCGACTACTTGAGACAGACCATCGCATGGTCATTCCTGTTGAGTCCCCCATCCGGGTTCTTGTCTCCGCCGAGGATGTTTTACACTCATGGGCCGTCCCAAGCCTCGGAGTAAAAATGGACGCCGTCCCCGGCCGCCTAAATCAAACAGCCTTCATTACTTCCCGTCCGGGTGTGTTTTATGGACAATGCTCAGAAATTTGCGGAGCTAATCATAGCTTTATACCCATTGTAGTGGAAGCTGTTCCTCTAGAACACTTCGAGAACTGATCTTACCTAATACTTCAAGATGCCTCACCAGGAAGCTAAAAGGGAATAGCATTAGCCTTTTAAGCTAAAAATTGGTGACTCCCGCCCACCCCTGGTGACATGCCTCAGTTGAACCCCGCACCCTGATTTGCTATTATAGTATTCTCATGACTAGTTTTCCTAGCCGTTATCCCACCTAAAGTTCTAGCCCACCATTTTCCCAATGACCCCGCCCCACAGAGCGTAAAAAAATCAAAAACAGAGACCTGATCCTGACCATGACTTTAAGCCTCTTTGATCAATTTATGAGCCCTACACTTCTAGGGGTGCCTCTTATCGGACTCGCCCTAACATTGCCATGAGTCCTTTACTTCCAACCCGGTGCCCGATGACTTAATAACCGCTTGATTACCCTTCAATCTATATTCATGAACTGATTTGTAAAACAAATCTTTCAGCCAATAAGCTTAGGCGGGCACAAATGGGCCGCTCTCCTCATATCTTTAATACTATTTTTAATTACCTTAAATATGCTAGGCCTACTGCCTTATACATTTACTCCAACAACGCAGCTGTCACTTAATATAGCCTTTGCAGTACCACTTTGACTAGCAACTGTCATTATTGGAATACGAAACCAGCCAACACATGCCCTTGGTCACCTCCTCCCCGAAGGAACTCCTACCGCCCTAATCCCGGTTTTAATCGTGATTGAGACAATTAGCCTTTTTATTCGACCATTGGCCCTCGGTGTTCGACTTACCGCAAACTTGACAGCCGGGCACCTTCTAATTCAACTAATTGCAACTGCGGCTTTTGTTCTTTTCCCTATAATGCCCACAGTCGCCGCTCTTACCTCTGTCTTACTATTCTTGCTGACCCTACTAGAAGTCGCCGTGGCCATAATCCAAGCCTATGTATTCGTACTTCTTTTAAGCCTTTACCTACAAGAAAACGTCTAATGGCCCATCAAGCACATGCATATCATATAGTTGACCCAAGCCCTTGACCCCTCACAGGCGCAGTAGCCGCCCTTCTACTTACATCTGGAACAGCAATCTGAATACACTTTAACTCCACAGTTCTCATGTCCCTTGGACTTGTTCTGTTACTACTAACCATATACCAATGATGACGAGACATTATCCGAGAGGGCACCTTTCAAGGTCATCATACACCCCCTGTTCAAAAAGGCCTTCGGTACGGGATAATTCTATTTATTACCTCAGAGGTCTTCTTTTTCCTAGGTTTCTTCTGAGCATTTTATCACTCAAGCCTAGCCCCAACCCCCGAACTTGGTGGATGTTGACCACCCATGGGTATTACAACACTGGACCCCTTTGAAGTTCCCCTTCTCAATACTGCTGTCCTTCTCGCCTCCGGTGTCACGGTCACTTGAGCTCACCATAGTATTATGGAGGGGCAGCGAAAACAAGCAATTCAGTCCTTGACACTCACAATTCTCCTGGGGTTTTACTTTACATTCCTTCAAGCAATAGAGTACTACGAGGCACCCTTCACCATTGCAGATGGCGTCTATGGCTCTACATTTTTTGTGGCAACAGGGTTTCATGGCCTCCATGTAATTATTGGATCAACATTTCTGGCAGTCTGCCTCTTACGACAAGTCCAGTTCCATTTTACATCAGAACATCACTTCGGATTCGAAGCTGCAGCATGATACTGACACTTTGTAGACGTAGTCTGACTATTCTTATATATCTCTATCTACTGATGAGGCTCATATCTTTCTAGTATTAAAAAGTACAAGTGACTTCCAATCACTCAGTCTTGGTTAGACTCCAAGGAAAGATAATGAACTTAGTACTAGTCATTATTTGCATCTCATTAGCCCTCGCCGCACTGCTCGCAACTGTTTCATTTTTCCTCCCACAAATAACCCCTGATTATGAGAAACTCTCACCGTATGAGTGCGGCTTTGATCCAGTAGGGTCCGCCCGTTTGCCATTCTCCATTCGCTTTTTTCTAGTCGCAATCCTATTTCTCCTCTTCGACTTAGAAATTGCCTTACTTCTTCCCCTTCCCTGAGGGGACCAACTTCCCTCCCCTCTAACAACTTTCTTTTGAGCTTCTGCTATTCTTATACTACTAACTCTAGGGTTAATCTATGAGTGACTTCAAGGAGGCCTAGAGTGGGCAGAATAGGTACTTAGTTTAATAAAAACATTTGATTTCGGCTCAAAAACTTATGGTTTAAGTCCATATTTACCTGATGACCTTAACTCACTATGCATTCTCGTCAGCCTACTTTGTTAGCTTCATGGGTTTAATTTTTTACCGAAAGCATCTTCTCTCCGCCTTACTTTGCTTAGAAGCGATAATACTTATTCTTTTTATTTCACTATGCCTGTGAGGTCTAGTCTTAGCCTCAAGTGCATTTTCGGCAGGCCCAATGATCTTACTTGCTTTCTCAGCATGTGAAGCAAGTGCTGGCCTAGCACTGCTTGTAGCAATAGCTCGAACCCACGGTACTGACCGTCTAAAAAACCTTAGCCTACTCCAATGTTAATAATTCTTATTCCTACTGTTATGCTTCTCCCCACAATCTGACTGAGCCCCACTAAATACCTGTGATCCTCAACACTTGGCCATAGCATAATAATTGCTCTTATAAGCCTCTCCTGACTTAGCCTCCCAGGGGAAGTTGGCTGATCTTCCCTTAACACTTTTATAGCAACAGACCCTCTCTCTACCCCCCTTCTCGTACTTACTTGCTGACTTCTGCCCTTAATAATTCTTGCGAGCCAAAACCATATAGCCCAAGAACCTACCAATCGCCAGCGAACCTATATCTCTCTCCTTACTTCCCTTCAAATCTTCTTAATCTTAGCATTTGGGGCAACCGAGATAATCATGTTCTACATTATATTTGAAGCGACCTTAATTCCCACACTCGTAATTATCACACGATGAGGAAACCAAACAGAGCGATTAAACGCAGGTATTTACTTTTTATTTTATACCTTAGCCGGCTCTTTACCACTACTAGTGGCCCTCCTTCTACTTCAGACCTCGACAGGAACTCTTTCTTTTCTAACCACTCAATTTTTTCCCCCTTTACAACTGCATACAGAAGCAAGTAAATTCTGGTGGGCAGGCTGTTTACTAGCATTCTTAGTAAAAATGCCCCTATATGGAGCACACCTTTGACTTCCAAAAGCTCACGTCGAAGCCCCCATCGCCGGGTCAATAGTCCTTGCAGCCGTTCTTTTAAAACTAGGAGGTTACGGTATAATACGAGTCATTATTATCTTAGAGCCCCTCACGAAACAACTCAGCTACCCCTTTATTGTTCTTGCCCTGTGGGGCGTCGTAATAACTGGCTCAATTTGCCTCCGACAAACAGACCTTAAATCACTAATCGCTTACTCCTCAGTAAGCCACATAGGCCTTGTCGCAGCAGGCATCCTAATCCAAACTCCTTGGGGGTTTACAGGAGCATTAATCCTTATAATTGCCCATGGCTTAACTTCCTCCGCCCTATTCTGTCTAGCCAACACTAACTATGAGCGAACACATAGCCGAACCATGCTTTTAGCCCGGGGTCTACAAATGGTCCTTCCTCTCTTAGCAACTTGATGGTTTCTATTTACCCTCGCCAACCTAGCACTCCCTCCACTACCCAACCTCATAGGAGAACTTATGATTATCTCATCCTTGTATAACTGATCAAACTGGTCTCTAATCCTGACCGGGACGGGAGTACTAATTACCGCTAGTTACTCTCTCCATATATTCCTAACCACTCAACGTGGCCCTATTACTAACCCCATCTTGGCAATTGAACCAACCCACACACGAGAACATCTCCTCATAATCCTTCACCTTCTTCCCCTCCTCCTTCTAATTTTAAAACCCTGCTTGATCTGGGGCTGAACAGTTTGTAGGCGTAGTTTAAATAAAGCGCTAGATTGTGATTCTAGAAATAAGAGTTAAACCCTCTTCACCCACCGAGAGGGGTCGCCGTGACAGCAAGAACTGCTAATTCTAGCCCCTTTGGTTAAAGTCCGAAGCCCACTCGAACAGGCTTCTAAAGGATAACAGCTCATCCGTTGGTCTTAGGAACCAAAAACTCTTGGTGCAACTCCAAGTAGCAGCTATGCACTTTACAACAATGATTCTCTCCTCAAGCCTAATAACAATTTTCCTTCTTCTTATCCTTCCAGTCCTAGGTACACTAAACCCTAACCCCACAGGGGACCTGTGGGCCACAAAAAACGTTAAAACAGCAGTTAAGATGGCCTTTTTTGTAAGTCTTCTACCTCTTTTTATCTTTCTTAATGAAGGAGTAGAGACTATTATAACAAACTGAAAATGGATAAATACTCTAATATTTGAAATTAATATCAGCTTTAAATTTGACCTCTACTCCGTGGTATTTACCCCTGTAGCCCTCTACGTGACATGATCAATTTTAGAGTTCGCATCTTGGTACATACACAGTGACCCCAATATAAACCGATTCTTTAAGTATCTTCTAATCTTTCTAATCGCTATGATTGTTCTGGTTACAGCCAACAACATGTTCCAACTATTTATTGGCTGAGAAGGTGTTGGAATTATGTCTTTCTTACTTATTGGCTGGTGGTTCGGGCGGGCTGACGCCAACACTGCGGCCCTCCAGGCCGTAGTTTATAACCGAGTCGGTGATGTCGGCCTAATTCTAGCAATAGCATGAATAGTAGTAAACCTAAACTCATGAGAGATACAACAGCTCTTTTCTGTGTCTAAAGGTCATGATATGACCCTTCCCTTATTAGGCCTAGTACTGGCCGCTACCGGAAAGTCCGCCCAGTTTGGACTTCACCCCTGGCTCCCCTCAGCCATAGAGGGTCCAACACCGGTCTCTGCCCTCCTGCACTCTAGCACCATGGTTGTTGCTGGTATTTTCCTTCTTATCCGCCTCAGCCCCTTAATGCAAGAAAGCCCGTTAATTCTCTCAACATGCCTTTGCCTGGGGGCCCTAACTACCGTCTTTACTGCAACATGTGCCCTTACCCAAAATGACATCAAAAAAATTGTTGCATTTTCTACATCAAGTCAATTAGGACTAATAATAGTCACCATCGGACTAGGCCAGCCCCAGCTCGCCTTTCTTCATATCTGCACCCACGCCTTCTTTAAAGCAATACTTTTCTTATGTTCAGGCTCCATCATTCATAGCCTTAATGATGAGCAAGATATCCGAAAAATAGGAGGACTTCACAAGCTCCTTCCACTGACCTCTTCTTGTCTAACCATTGGCAGCCTAGCTCTAACAGGAGTCCCCTTTTTAGCAGGCTTCTTTTCCAAAGACGCCATCATTGAAGCTATAAATACATCCTACCTTAACGCCTGGGCCCTAATTTTAACGCTTCTAGCTACATCATTTACCGCAGTTTACAGTCTCCGTGTCGTATTCTTTGCCTCTATGGGCCACCCGCGTTTTAATCCAATCTCCCCTATTAATGAAAATAACCCTACAGTGATAAACCCTATTAAACGACTCGCTTGGGGAAGCATTTTGGCAGGGTTGCTAATTACGGCCAATATTGTTCCACTTAAAACCCCCGTTTTAACCATGCCTTTCACCTTAAAAATGGCCGCACTGGCTGTAACAATCATAGGACTACTCACAGCCTTAGAACTAGCATCTCTCACGTCCCAACAATTTAAAATCAAACCCTTATCTTCTACTCACCACTTCTCAAATATATTAGGATTTTTCCCGAGTGTGGCCCATCGACTAGTCCCAAAAACTGGCCTGATTCTTGGGCAACTAGTTGCCAATCAGACAATTGACCAAACCTGACTAGAGAAAACCGGGCCAAAAATAGTAACCTCCATTAACCTTCCAATGGCTACTTCAATTAGCAACCTACAGCAAGGTGTAATTAAGACCTACTTCTTATTATTTTTCTTCACCATAATACTGGCAATTCTCATCCTTGTCATCTAACTGCCCGTAAGGTCCCCCGACTTAGCCCTCGAGTTAGCTCCAGAACTACAAAAAGCGTCAGTAATAAAACTCATCCCCCAAGCATTAAAACTCCTCCTCCTGAAGAATATATCAGAGCAACCCCACCAAGGTCCCCCCGGAATAGCATGAATTCACTAAACTCGTCAGCAGTTATCCATGACCCCTCATACCAACCCTCAGAGAAAAAGACAGAGATAGACGCGACCAGAAACACATATACTGACATAAGAAGCAAAACGGGTCAACTTCCCCACCCCTCAGGATAGGGCTCCGAAGCCAGCGCTGCTGAATACGCAAACACAACTAACATCCCACCTAAATAGATCAAAAACAAAATCAGAGATAAAAATGAACCCCCGTGCCCTACTAAAATGCCACAGCCCATTCCTGCTACTGTGACAAGGCCCAAAGCAGCAAAGTAGGGTGAGGGGTTTGAGGCCACGGCCGCTAGACCTAAAACCAGACCAACTAATAATAAATAAGTTATATAAACCATAATTCTTGCCAGGATTTTAACCAGGGCCTGCGACTTGAAAAACCACCGTTGTACTCAACTACAAGAACCTAATGGCCAATCTTCGAAAAACCCATCCCCTATTAAAAATCGCAAACGATGCCCTGGTTGATCTCCCAGCCCCATCGAACATTTCAGTTTGATGAAACTTCGGGTCTCTTCTAGGACTTTGTTTGGCCGCCCAGATTGTTACGGGCCTTTTCCTTGCAATACATTATACATCAGACATTGCCACAGCATTTTCATCTGTAGCACATATTTGTCGTGACGTCAACTACGGCTGACTAATCCGAAACATGCATGCAAACGGTGCTTCCTTTTTCTTCATTTGCATCTACCTGCACATCGGACGGGGCTTGTATTATGGATCATACTTATATAAAGAGACATGAAATGTGGGTGTTGTCCTTCTCCTCCTAGTGATAATGACTGCTTTCGTAGGCTACGTCCTACCCTGAGGACAAATGTCATTCTGAGGGGCTACCGTCATTACCAACCTTTTATCAGCCATTCCCTACGTTGGAAACGCCCTAGTTCAATGAATCTGAGGCGGATTTTCAGTAGACAACGCCACCCTTACCCGGTTCTTTGCCTTCCATTTCCTCCTTCCCTTTGTGATTGCTGCTGCTACAGTTGTGCATCTTATCTTCCTACACGAGACAGGATCAAATAATCCAACAGGTTTAAACTCAGACTCTGACAAAGTATCTTTTCACCCCTACTTTTCTTATAAAGATCTTCTAGGCTTTGCTGCCCTACTAGTAGCCCTTATCTCTTTAGCCCTCTTCTCCCCGAATCTACTCGGAGACCCCGATAACTTTACCCCTGCTAATCCTTTAGTGACTCCACCTCACATCAAACCTGAGTGATACTTCTTGTTCGCTTACGCCATTCTACGATCCATCCCAAACAAACTTGGCGGGGTTCTAGCCCTATTAGCCTCTATTCTAGTCCTCTTTCTTGTTCCCATTCTGCACACATCAAAACAACGAAGCCTAACATTCCGACCCCTGACCCAATTCCTCTTCTGATTGCTAGTCGCCGATGTAATAATTTTAACCTGAATTGGAGGTATGCCTGTAGAACATCCTTACATTATCATTGGACAAATCGCATCCTTCATTTATTTTTTTCTTTTCCTAGTCATAGCGCCTATGGCCGGCCTACTAGAAAACAAAGTCTTAAAATGACAATGCATTAGAAGCTCAGATGAAAGAGCACCGGTCTTGTAAACCAGAGGTCGAAGGTTCAAGCCCTTCCTAGTGCTCAGAGAGAAGGGATTCTAACCCCTGCCCCTGACTCCCAAAGCCAGGATTCTTAGCTAAACTACTCCCTGATTTTCATACACCAGTTTTGCAATCCAGAGCGCATCACTTTTGCCACCAACGTTAAATTAACGTTGCACAAACGTTGCATCAGCGCCCCATGGACACTAAATGACGCGAGGGCGCTAAATAAACACCCCCTACCTCTAGCACCCTTTTAACGATTTCACTTTTTTTTTTTTTTTTGTTTAACGATTACGTTTTTTTTTGCGTTCCCGGACTCTGCCAGATTTCGACCGAAGACTGCCAGAATCCGCTCAAAATTCGCTCAAATACCAATATGTATTATCCCCATAAATGGTTTAAACCATTTTTGCCTAGTACACACTGACCATGCAAGTCAATTATATTTACCCCGCGCTCCAGGCCGCAGTACATACACCTACAGTTGGTGTATTTAGCACAAGTGTGCCTCAGCTAGTTTCAAGTCACCCACATCCTTCCTTTAATTGTTACTTAATGTAGTAAGAGCCCACCATCAGTTGATTCCTTAATGTCAACGGTTCTTGAAGGTGAGGGACAAAAATCGTGGGGGTTTCACTTCTTGAATTATTCCTGGCATTTGGCTCTACATCTCAAGGCCATACATTTCTCGTCTCTCACACTTTCACTGGCCCTGACATTGGTTAATGGTGGAGTACATACTCCTCGTTACCCCCCATGCCGGGCGTTCTTTCTAATGGACAACGGGTTTTCCTTTTTTTTTCCTTTTCACTTGGCATTTCACAGTGCATACAAACCTTGATGACAAGGTTGAACATTTAGAAATCGGCCGCAAAGAATATTGGTGAATTATTTAAAGATATTAACAGATGAATTGCATAAGTGATATCAAGAGCATAAATAACCAAATGAAACTAGGAACGTTTCTATAATATGCCCCCCGGCTTCCGCGCGTCAAACCCCCCTACCCCCCTAAACTAGTAAGAAGTCTATTATTCCTGCAAACCCCCCGGAAACAGGAAACCCCCTACTAGCATTTTAGCCCGCCCAAATTTGTGTGTATTTATATTATTTGTAATATTGCAAAA

>GQ 18

GCTAGTGTAGCTTAACTAAAGCATAACACTGAAGATGTTAAGACAAACCTTAGATTGGTTTCACGAGCACAAAAGTTTGGTCCTGACTTTACTATCAACTTTAGCTAAACTTACACATGCAAGTATCCGCAATCCCGTGAGAATGCCCTACAGTTTCCTTAAAGGAAACAAGGAGCTGGTATCAGGCTCAATTACTCCCGCCCATGACACCTTGCTTAGCCACACCCCCAAGGGAACTCAGCAGTGATAGACATTAAGCAATAAGTGAAAACTTGACTTAATTAAAGCTAAGAGAACCGGTTAAACTCGTGCCAGCCACCGCGGTTATACGAGCGGTTCGAGCTGATAGATTACGGCGTAAAGCGTGGTTAATAAGAATGAAACTAAAGTCGAATGTTTTCAAAGCTGTTATACGCACTCGAAAATTAGAAGGTCAGAAACGAAAGTGACTTTAACCCTATGAACCCACGAAAACTATGAAACAAACTGGGATTAGATACCCCACTATGCATAGCTGTAAACTTTGATGAGCTATTACATTATCATCCGCCTGGGTACTACGAGCATCAGCTTAAAACCCAAAGGACTTGGCGGTGCTTTAGACCCACCTAGAGGAGCCTGTTCTAAAACCGATAACCCCCGTTAAACCTCACCCTCTCTTGTTTTTCCCGCCTATATACCGCCGTCGTCAGCTTACCCTGTGAAGGTCTAATAGTAAGCACAACCAGTTATACTCAAAACGTCAGGTCGAGGTGTAGCATATGAGAGGGGAAGAAATGGGCTACATTCCTTGTTTCAAGGAAAACGGATAACATAATGAAAGGTACGTTAGAAGGAGGATTTAGCAGTAAGCAGCAAATAGAGTGTTCTGCTGAAACTGGCCCTGAAGCGCGCACACACCGCCCGTCACTCTCCCCAACTCCGAGTTAAAAACATATATAAACCTTTGAAGGAACAAAGGGGAGGCAAGTCGTAACATGGTAAGTGTACCGGAAGGTGCACTTGGATAAATCAGAGTATAGCTAAGAAAGAAAAGCATCTCCCTTACACCGAGAAGTCATCCGTGCAAATCGGATTACCCTGACTCTAACAAGCTAGCCCAAAACCTTAACTTAAAAATCAAATATTTCTAGTAATTAATAAACCAAACACATTAAATAAATCATTTTTCCCCCTGAGTATGGGAGACAGAAAAGGATAAAGGAGCTATAGACAAAGTACCGCAAGGGAAAGCTGAAAGAGAAATGAAACAAACCAGTAAAGAAAAACAAAGCAGAGATTAACCCTTGTACCTTTTGCATCATGAATTAGCCAGTTTAATCAAGCAAAGAGCACTGTAGTTTGAGACCCCGAAACTTAGTGAGCTACTTCAAGACAGCCTACGAAATAGGGCAAACCCGTCTCTGTGGCAAAAGAGTGGGAAGATCTTCAAGTAGAGGTGACAGACCTATCGAACTAAGTAATAGCTGGTTGCTCGTGAAATGAATAGAAGTTCAGCCTTTTGCTTTCTAAATTTCGATTTAGCATCACTTAGCCTAAATGATTAGAAAACAAAAGAGTTAGTCAAAGAGGGTACAGCCTGTTTGATAAAAGATACAACTTTACTAGGAGGATAAGAATCATAATTTTAAAGGTTTAATGCCCAGGTGGGCCTAAAAGCAGCCACCCTGATCAATAGCGTTAAAGCTTAAGCATAAAACACACCTACAATTCTGATAAATCAGTTTTAATCCCCTAAAGTTAACGAGCTATTTCATACCTTATGAAAGAAATTATGCTAGTATGAGTAATAAGAAGTTACGAACTTCTCCCTGCACACGTGTAAATCGGAACGGACAAACCACCGAATCTTAACGGCCCCAGTCAAAGAGGGGATGTCGGATAAAAAAAAGAACAAGAAATTCCCGATAAAACCACCGTTGACCCCACACCGGAGTGCTCCCTGGGAAAGACAAAAAGGGACAGAAGGAACTCGGCAAATATGCTCAAGCCTCGCCTGTTTACCAAAAACATCGCCTCTTGTAAAAGTTAAATAAGAGGTACCGCCTGCCCTGTGACTAGTAGTTTAACGGCCGCGGTATTTTGACCGTGCAAAGGTAGCGCAATCACTTGCCTTTTAAATGAAGGCCTGTATGAATGGCACGACGAGGGCTTAACTGTCTCCTCTCCCTAGTCAATGAAATTGATCCCCCCGTGCAGAAGCGGGGATAATAACATAAGACGAGAAGACCCTGTGGAGCTTTAGACTATGAGCAGACCATGTCAAGAATAACAAACAAGTAAATTAAACAAATTGGCCCCTGCTTCCCTGTCTTTGGTTGGGGCGACCGCGGGATAATAAAAAACTCCCACGAGGATTGAGAACCCTTATCTTATAACCAAGAGCTTCTCCTCTAAGTAACAGAACATCTGACCTTAATGATCCGGCCTGGCCGATCAACGGACCGAGTTACCCCAGGGATAACAGCGCAATCCTCTTTTAGAGTCCATATCGACAAGAGGGTTTACGACCTCGATGTTGGATCAGGACATCCTAATGGTGCAGCCGCTATTAAGGGTTTGTTTGTTCAACAATTAAAGTCCTACGTGATCTGAGTTCAGACCGGAGTAATCCAGGTCAGTTTCTATCTATGACGTACTCTCTTCTAGTACGAAAGGACCGAAGAAAGAAGGCCCATGAAAAATTATGCCTTAGTCTCACCTTATGAAGAAAACTAAATAAGACAAGAGGTTACACCCTTTGGTCATAGAAAATGACATGTTAAGGTGGCAGAGCCCGGATATTGCAAAAGACCTAAGCCCTTTCCACAGAGGTTCAATTCCTCTCCTTAACTATGTTCTCAACAATATTAAGCTTCATTATTAATCCCCTGATTGTTATGATTTTTGTTTTGTTGGCAGTAGCCCTCTTGACCTTGGTAGAGCGTAAAGTGCTAAGCTACATGCAACTTCGTAAAGGCCCAAATGTTGTTGGCCCTTACGGCCTTTTGCAACCCTTCGCTGATGGCTTAAAACTTTTCATGAAAGAGCCCGTCCGACCCTCCACCTCCTCGCCCGCCTTGTTCTTAATTACCCCTATTATAGCCCTTACCTTAGCCCTAACCCTCTGAGCCCCCCTTCCTATGCCTTTTCCCGTCACCGACCTAAACTTAGGCATTTTATTTATTTTAGCACTATCGAGCCTGGCAGTATATTCTATTCTTGGCTCCGGATGAGCCTCCAATTCTAAATATGCATTAATTGGTGCTCTTCGAGCGGTCGCCCAAACCATCTCTTATGAAGTAAGCTTGGGCCTTATTCTTCTTAACACAATTGTCTTTACGGGGGGTTTTACTCTTCAAACCTTCAGCACCGCACAAGAAGCCACCTGATTACTTCTACCAGCATGACCACTAGCAGCCATGTGATATATCTCCACACTCGCGGAAACTAACCGGGCCCCTTTCGACCTAACTGAAGGAGAGTCCGAACTAGTGTCTGGCTTCAACGTAGAGTATGCCGGCGGACCTTTTGCCCTTTTTTTTCTGGCAGAATACGGTAACATTTTACTTATAAATACCCTCTCAGCAGTACTATTTCTAGGCTCTTCAACCTACCACAACTTTCCAGAACTAACCGCGACCTTATTAATGCTTAAAGCCACCCTCCTTTCAGTCGTATTTTTATGAGTGCGAGCATCTTACCCTCGGTTCCGATACGACCAACTAATGCATTTAATTTGAAAAAACTTTTTACCTCTGACCCTAGCGCTAGTTATTTGACACCTTTCTCTTCCAATCACGTTGAGCGGCCTCCCTCCTCAACTTTAACTCAGGAAATGTGCCTGAAAAAGGGTCACTTTGATAGGGTGAATAATGAGGGTTAAAGCCCCTCCATCTCCTTAGAAAGAAGGGGTTTGAACCCTACCTGAAGAGATCAAAACTCTTAGTGCTTCCACTACACCACTTCCTAGTAAAGTCAGCTAATAAAAGCTTTTGGGCCCATACCCCAAATATGTTGGTTAAAATCCTTCCTTTGCTAATGAATCCTTACGTCCTTTCAATTCTACTTATGGGCTTAGGCCTCGGCACTACAGTCACATTCGCTAGCTCACACTGACTCTTAGCATGAATAGGCCTTGAAATAAATACCCTCGCCATTTTGCCATTAATAGCACAACATCACCACCCCCGAGCCGTTGAAGCCACCACCAAGTATTTTTTAATTCAATCGGCAGCCGCAGCAACCATCTTATTTGCCAGCTCAACTAACGCCTGACTTTCGGGCCAGTGGGATATCATAAGTATCAATCACCCCCTTCCAACCGTCATAATTACAATCGCTCTGTCCTTAAAACTAGGCTTGGCCCCTCTTCACGCCTGACTTCCCGAAGTTATTCAAGGACTAGACTTGACCACGGGCTTAATCCTCTCCACATGACAAAAACTCGCACCCTTTGCCCTTCTCGTTCAAATCTTCCCCGACACCCCCCTTCTCATCACTTCTCTAGGACTTCTTTCAATATTAATTGGGGGGTGAGGAGGTTTAAACCACACACAACTCCGCAAAGTGCTCGCATATTCTTCGATCGCCCACTTAGGCTGAATAATAGTAATTATGCAATTCTCCACCCCCCTTACAATTCTTGCTTTATCAACATACATTGTTATAACATCATCTACTTTTCTAATCTTTAAACTCCTTAAATCCACAAATATGAACAGCCTGGCAACATCTTGAACTAAAACCCCCTCCATTACAGCCCTAGCACCTTTAGTGCTATTATCCTTAGGCGGACTTCCTCCCCTCTCGGGCTTTATGCCAAAATGATTAATTATTCAAGAATTAACTAAGCAAGATCTAGCCCTAGTTGCAACCTTGGCCGCCCTCTCTGCGCTACTCAGCCTTTTCTTCTACCTACGCATTTGTTACTCCCTCACATTTACCTCCTCTCCTAATAATCTCATGGGAACACCCCCCTGACGACTAGTAACAAAGCAAGTATCACTTCCCCTAGCTATAACAACCTCCCTCTCTATTCTTCTACTCCCGGTTACCCCTGCAATCTTATCAGTGGTTCTCCCTTTGTAAAGAGGCTTAGGATAGTATTAAGACCAAGGGCCTTCAAAGCCCTAAGCGGGAGTGAAAGCCCCCCAGCCTCTGTAAGACCTACGGGACACTAACCCACATCTTCTGTATGCAAAACAGACACTTTAATTAAGCTAAAGCCTTCCTAGGTGGGTAGGCCTCGATCCTACAATCTCTTAGTTAACAGCTAAGCGCCTAAACCAGCGGGCATCCATCTACCTTTCCCCCGCCTTGCCGAAAAAAAAAGGCGGGGGAAAGCCCCGGCAGGGTATTAGCCTGCCACTTAAGATTTGCAATCTAATGTGTTAACACCTCGGGGCTGGTAAGAAGAGGACTTTAACCTCTGTCCATGGGGCTACAATCCACCGCTAAACGCTCAGCCACCTTACCTGTGGCAATCACACGTTGATTTTTCTCAACTAATCACAAAGACATCGGCACCCTGTATCTAATCTTTGGTGCCTGGGCGGGAATGGTAGGGACGGCCTTAAGTCTACTCATTCGGGCAGAACTAAGTCAACCAGGCTCCCTATTAGGAGACGACCAGATCTATAACGTAATTGTAACTGCACATGCTTTCGTAATAATTTTCTTTATAGTAATGCCAATCATAATTGGGGGGTTTGGCAACTGATTAATCCCTTTAATGATTGGAGCCCCCGACATGGCCTTCCCACGGATAAATAATATAAGCTTTTGACTCCTGCCCCCTTCTTTCCTTCTATTATTGGCCTCATCTGGTGTAGAAGCTGGTGCCGGAACAGGATGAACCGTATATCCCCCCTTGTCAGGTAATTTGGCACACGCAGGGGCCTCCGTAGATTTAACCATTTTCTCTCTTCACCTGGCCGGAATTTCTTCTATCCTAGGGGCCATTAATTTCATCACAACTATTATTAATATAAAACCTCCAGCCATTTCCCAATATCAAACCCCTTTATTTGTGTGGGCTGTACTAATTACCGCAGTATTACTTCTACTCTCTCTTCCTGTTCTAGCTGCAGGTATCACCATGCTTCTCACAGATCGGAACCTAAATACAACATTTTTCGACCCCGCAGGAGGGGGGGACCCCATTCTTTATCAACATTTATTCTGATTCTTTGGGCATCCTGAAGTCTACATTCTAATTTTGCCCGGCTTCGGAATGATTTCTCACATTGTAGCATATTACTCAGGCAAAAAAGAGCCGTTTGGTTACATGGGAATAGTATGAGCTATAATAGCAATTGGCTTGCTGGGCTTTATCGTATGAGCCCATCATATGTTCACTGTAGGGATGGACGTGGACACTCGAGCTTATTTTACTTCCGCCACTATAATTATCGCAATTCCCACAGGAGTCAAAGTGTTTAGTTGACTAGCTACCTTGCATGGGGGCTCAATCAAATGAGAAACCCCCCTGTTATGAGCTCTAGGCTTTATCTTCTTATTTACTGTCGGAGGTTTAACAGGAATTGTTTTAGCCAACTCATCTCTGGACATTATACTTCATGATACATACTATGTTGTAGCCCACTTCCACTATGTCCTCTCTATAGGAGCAGTCTTTGCCATCATGGGAGCATTCGTTCACTGATTTCCCCTATTCTCAGGCTACACCCTTCACAATACGTGAACAAAAATCCACTTCGGAGTTATGTTTGTAGGTGTAAACCTCACCTTTTTCCCTCAGCACTTCTTAGGATTGGCGGGAATACCTCGACGATACTCAGATTACCCTGACGCATACACACTGTGAAATACTATCTCATCCCTGGGGTCATTAATCTCCCTTATTGCTGTAATTATATTCCTATTTATTATCTGGGAGGCATTCGCGGCAAAACGTGAAGTCTTATCAGTTGAACTAACAGCCACAAACGTAGAATGACTGCACGGGTGTCCTCCCCCTTACCATACATTTGAAGAACCTGCATTCGTTCAAATTCAACAATCCAAATTTTAATCGAGAAAGGAAGGAGTCGAACCCCCATAAACTGGTTTCAAGCCAGCCACATAACCGCTCTGTCACTTTCTTCCCTAAGTTAATAAGATTCTAGTTAAAGGAATAACACTGCCTTGTCAAGGCAAAATTGTGGGTTAAAGCCCCACGTATCTTGCTTATGGCACATCCATCTCAACTAGGATTCCAAGATGCAGCTTCACCCGTTATAGAAGAACTTCTCCATTTTCATGACCATGCATTAATAATTGTTTTCTTAATCAGCACCCTTGTTCTTTACATTATTGTGGCTATGGTAACCACCAAGCTAACAAATAAGTTCATTCTGGACTCCCAAGAAATTGAAATCATCTGAACCTTGCTACCAGCAATTATCCTAATTCTGATCGCCCTACCCTCCCTTCGCATTCTCTACCTCATGGATGAAATCAATGACCCCCACCTCACAATTAAAGCCATAGGACATCAATGATACTGAAGCTACGAATATACGGATTATGAAGACCTAGGGTTCGACTCGTATATGGTCCCTACACAAGACCTCGCCCCTGGTCAATTTCGACTACTTGAAACAGACCATCGCATGGTCATTCCTGTTGAGTCCCCCATCCGGGTTCTTGTCTCCGCCGAGGATGTTCTACACTCATGGGCCGTCCCAAGCCTTGGAGTAAAAATGGACGCCGTCCCCGGCCGCCTAAATCAAACAGCCTTCATTACTTCCCGTCCGGGTGTGTTTTATGGACAATGCTCAGAAATTTGCGGAGCTAATCATAGCTTTATACCCATTGTAGTGGAAGCTGTTCCTCTAGAACACTTCGAGAACTGATCTTACCTAATACTTCAAGATGCCTCACCAGGAAGCTAAAAGGGGATAGCATTAGCCTTTTAAGCTAAAAATTGGTGACTCCCGCCCACCCCTGGTGACATGCCTCAGTTGAACCCCGCACCCTGATTTGCTATTATAGTATTCTCATGACTAGTTTTCCTAGCCGTTATCCCACCTAAAGTTTTAGCTCACCATTTTCCCAATGACCCCGCCCCACAAAGCGTAAAAAAATCAAAAACAGAGACCTGATCCTGACCATGACTTTAAGCCTCTTTGATCAATTTATGAGCCCTACACTTCTAGGGGTGCCTCTTATCGGACTCGCCCTAACATTGCCATGAGTCCTTTACTTCCAACCCGGTGCCCGATGACTTAATAACCGCTTCATTACCCTTCAATCTATATTCATGAACTGATTTGTAAAACAAATCTTTCAGCCAATAAGCTTAGGCGGACACAAATGGGCCGCTCTCCTCATATCTTTAATACTATTTTTAATTACCTTAAATATGCTAGGCCTACTGCCTTATACATTTACTCCAACAACGCAGCTGTCACTTAATATAGCCTTTGCAGTACCACTTTGACTAGCAACTGTCATTATTGGAATACGAAATCAGCCAACACATGCCCTTGGTCACCTCCTCCCCGAAGGAACTCCTACCGCCCTAATCCCGGTTTTAATCGTGATTGAGACAATTAGCCTTTTTATTCGACCATTGGCCCTCGGTGTTCGACTTACCGCAAACTTGACAGCCGGACACCTTCTAATTCAACTAATTGCAACTGCGGCTTTTGTTCTTTTCCCTATAATGCCCACAGTCGCCGCTCTTACCTCTGTCTTACTATTGTTGCTAACCCTACTAGAAGTCGCCGTGGCCATAATCCAAGCCTATGTATTCGTACTTCTTTTAAGCCTTTACCTACAAGAAAACGTCTAATGGCCCATCAAGCACATGCATATCATATAGTTGACCCAAGCCCTTGACCCCTCACAGGCGCAGTAGCCGCCCTTCTACTTACATCTGGAACAGCAATCTGAATACACTTTAACTCCACGGTTCTCATGTCCCTTGGACTTGTTCTGTTACTACTAACCATATATCAATGATGGCGAGACATTATCCGAGAGGGCACCTTTCAAGGTCATCATACACCCCCTGTTCAAAAAGGCCTTCGGTACGGGATAATTCTGTTTATTACCTCAGAGGTCTTCTTTTTCCTAGGTTTCTTCTGAGCATTTTATCACTCAAGCCTAGCCCCAACCCCCGAACTTGGTGGATGTTGACCACCCATGGGTATTACAACACTGGACCCCTTTGAAGTTCCCCTTCTCAATACTGCTGTCCTTCTCGCCTCCGGTGTCACGGTCACTTGAGCTCACCATAGTATTATGGAGGGGCAGCGAAAACAAGCAATTCAGTCCTTGACACTCACAATTCTCCTGGGGTTTTACTTTACATTCCTTCAAGCAATAGAGTACTACGAGGCACCCTTCACCATTGCAGATGGCGTCTATGGCTCTACATTTTTTGTGGCAACAGGGTTTCATGGCCTCCATGTAATTATTGGGTCAACATTTCTGGCAGTCTGCCTCTTACGACAAGTCCAGTTCCATTTTACATCAGAACATCACTTCGGATTCGAAGCTGCAGCATGATACTGACACTTTGTAGACGTAGTCTGACTATTCTTATATATCTCTATCTACTGATGAGGCTCATATCTTTCTAGTATTAAAAAGTACAAGTGACTTCCAATCACTCAGTCTTGGTTAGACTCCAAGGAAAGATAATGAACTTAGTACTAGTCATTATTTGCATCTCATTAGCCCTCGCCGCACTGCTCGCAACTGTTTCATTTTTCCTCCCACAAATAACCCCTGATTATGAGAAACTCTCACCGTATGAGTGCGGCTTTGATCCAGTGGGATCCGCCCGTTTGCCATTCTCCATTCGCTTTTTTCTAGTCGCAATCCTATTTCTCCTCTTCGACTTAGAAATTGCCTTACTTCTTCCCCTTCCCTGAGGGGACCAACTTCCCTCCCCTCTAACAACTTTCTTTTGAGCTTCTGCTATTCTTATACTACTAACTCTAGGGTTAATCTATGAATGACTTCAAGGGGGCCTAGAGTGGGCAGAATAGGTACTTAGTTTAATAAAAACATTTGATTTCGGCTCAAAAACTTATGGTTTAAGTCCATATTTACCTGATGACCTTAACTCACTATGCATTCTCGTCAGCCTACTTTGTTAGCTTCATGGGTTTAATTTTTTACCGAAAGCATCTTCTCTCCGCCTTACTTTGCTTAGAAGCGATAATACTTATTCTTTTTATTTCACTATGCCTGTGAGGTCTAGTCTTAGCCTCAAGTGCATTTTCGGCAGGCCCAATGATCTTACTTGCTTTCTCAGCATGTGAAGCAAGTGCAGGCCTAGCACTGCTTGTAGCAATAGCTCGAACCCACGGTACTGACCGTTTAAAAAACCTTAGCCTACTCCAATGTTAATAATTCTTATTCCTACTGTTATGCTTCTACCCACAATCTGACTGAGCCCCACTAAATACCTGTGATCCTCAACACTCGGCCATAGCATAATGATTGCTCTTATAAGCCTCTCCTGACTTAGCCTCCCAGGGGAGGTTGGCTGATCTTCCCTTAACACTTTTATAGCAACAGACCCTCTCTCTACCCCCCTTCTCGTACTTACTTGCTGACTTCTGCCCTTAATAATTCTTGCGAGCCAAAACCATATAGCCCAAGAACCTACCAATCGCCAGCGAACCTATATCTCTCTCCTTACTTCCCTTCAAATCTTCTTAATCTTAGCATTTGGGGCAACCGAGATAATCATGTTCTACATTATATTTGAAGCGACCTTAATTCCCACACTCGTAATTATCACACGATGAGGAAACCAAACAGAACGATTAAACGCAGGTATTTACTTTTTATTTTATACCTTAGCCGGCTCTTTACCACTACTAGTGGCCCTCCTTCTACTTCAGACCTCGACAGGAACTCTTTCTTTTCTAACCACTCAATTTTTTCCCCCTTTACAACTGCATACAGAAGCAAGTAAATTCTGGTGGGCAGGCTGTTTACTAGCATTCTTAGTAAAAATGCCCCTATATGGAGCACACCTTTGACTTCCAAAAGCTCACGTCGAAGCCCCCATCGCCGGGTCAATAGTCCTTGCAGCCGTTCTTTTAAAACTAGGGGGTTACGGTATGATACGAGTTATTATTATCTTAGAGCCCCTCACGAAACAACTCAGCTACCCCTTTATTGTTCTTGCCCTGTGGGGCGTCGTAATAACTGGCTCAATTTGCCTCCGACAAACAGACCTTAAATCACTAATCGCTTACTCCTCAGTAAGCCACATAGGCCTTGTCGCAGCAGGCATCCTAATCCAAACTCCTTGGGGGTTTACAGGAGCATTAATCCTTATAATTGCCCATGGCTTAACTTCCTCCGCCCTATTCTGTTTAGCCAACACTAACTATGAGCGAACACATAGCCGAACCATGCTTTTAGCCCGGGGTCTACAAATGGTCCTTCCTCTCTTAGCAACTTGATGGTTTCTATTTACCCTCGCCAACCTAGCACTCCCTCCACTACCCAACCTCATAGGAGAACTTATGATTATCTCATCCTTGTATAACTGGTCAAACTGGTCTCTAATCCTGACCGGGGCGGGAGTACTAATTACCGCTAGTTACTCTCTCCATATATTCCTAACCACTCAACGTGGCCCTATTACTAACCCCGTCTTGGCAATTGAACCAACCCATACACGAGAACATCTCCTCATAATCCTTCACCTTCTTCCCCTCCTCCTTCTAATTTTAAAACCCTGCTTGATCTGGGGCTGAACAGCTTGTAGGCGTAGTTTAAATAAAGCGCTAGATTGTGATTCTAGAAATAAGAGTTAAACCCTCTTCACCCACCGAGAGGGGTCGCCGTGACAGCAAGAACTGCTAATTCTAGCCCCTTTGGTTAAAGTCCGAAGCCCACTCGAACAGGCTTCTAAAGGATAACAGCTCATCCGTTGGTCTTAGGAACCAAAAACTCTTGGTGCAACTCCAAGTAGCAGCTATGCACTTTACAACAATGATTCTCTCCTCAAGCCTAATAACAATTTTCCTTCTTCTTATCCTTCCAGTCCTAGGTACACTAAATCCTAACCCCACAGGGGACCTGTGGGCCACAAAAAACGTTAAAACAGCAGTTAAGATGGCCTTTTTTGTAAGCCTTCTACCTCTTTTTATCTTTCTTAATGAAGGAGTAGAGACTATTATAACAAACTGAAAATGGATAAATACTCTAATATTTGAAATTAATATCAGCTTTAAATTTGACCTCTACTCTGTGGTATTTACCCCTGTAGCCCTCTACGTAACATGATCAATTTTAGAGTTCGCATCTTGGTACATACACAGTGACCCCAATATAAACCGATTCTTTAAGTATCTTCTAATCTTTCTAATCGCTATGGTTGTTCTGGTTACAGCCAACAACATGTTCCAACTATTTATTGGCTGAGAAGGTGTTGGAATTATGTCCTTCTTACTTATTGGCTGGTGGTTCGGGCGGGCTGACGCCAACACTGCGGCCCTCCAAGCCGTAGTTTATAACCGAGTCGGTGATATCGGCCTAATTCTAGCAATAGCATGAATAGTAGTAAACCTAAACTCATGAGAGATACAACAGCTCTTTTCTGTGTCTAAAGGCCATGACATGACCCTTCCCTTATTAGGCCTAGTACTGGCCGCTACCGGAAAGTCCGCCCAGTTTGGGCTTCACCCCTGGCTCCCCTCAGCCATAGAGGGTCCAACACCGGTCTCTGCCCTCCTGCACTCTAGCACCATGGTTGTTGCTGGTATTTTCCTTCTTATCCGCCTCAGCCCCTTAATGCAAGAAAGCCCGTTAATTCTCTCAACATGCCTTTGCCTGGGGGCCCTAACTACCGTCTTTACTGCGACATGTGCCCTTACCCAAAATGACATCAAAAAAATTGTTGCATTTTCTACATCAAGTCAACTAGGACTAATAATAGTTACCATCGGACTAGGCCAGCCCCAGCTCGCCTTTCTTCATATCTGCACCCACGCCTTCTTTAAAGCAATACTTTTCTTATGTTCAGGCTCCATCATTCATAGCCTTAATGATGAACAAGATATCCGAAAAATAGGAGGACTTCACAAGCTCCTTCCACTGACCTCTTCTTGTCTAACCATTGGCAGCCTAGCTCTAACAGGAGTCCCCTTTTTAGCAGGCTTCTTTTCCAAAGACGCCATCATTGAAGCTATAAATACATCCTACCTTAACGCCTGGGCCCTAATTTTAACGCTTCTAGCTACATCATTTACCGCAGTTTACAGTCTCCGAGTCGTATTCTTTGCCTCTATGGGCCACCCGCGTTTTAATCCAATCTCCCCAATTAATGAAAATAACCCTACAGTAATAAACCCTATTAAACGACTCGCTTGAGGAAGCATTTTGGCAGGGTTGCTAATTACGGCCAATATTGTTCCACTTAAAACCCCCGTTTTAACCATGCCTTTCACCTTAAAAATGGCCGCACTGGCTGTAACAATTATAGGACTACTCACAGCCTTAGAACTAGCATCTCTCACGTCCCAACAATTTAAAATCAAACCCTTATCTTCTACTCACCACTTCTCAAATATATTAGGATTTTTCCCGAGTGTAGCCCATCGACTAGTCCCAAAAACTGGCCTGATTCTTGGGCAACTGGTTGCCAATCAGACAATTGACCAAACCTGACTAGAGAAAACCGGGCCAAAAATAGTAGCCTCCGTTAACCTTCCAATGGCTACTTCAATTAGCAACCTACAGCAGGGTGTAATTAAGACCTACTTCTTATTATTTTTCTTCACCATAATACTAGCAATTCTCATCCTTGTCATCTAACTGCCCGTAAGGTCCCCCGACTTAGCCCTCGAGTTAACTCCAGAACTACAAAAAGCGTCAGTAATAAAACTCATCCCCCAAGCATTAAAACTCCTCCTCCTGAAGAATATATCAGAGCAACCCCCCCAAGATCCCCCCGGAATAGCATGAATTCACTAAACTCGTCAGCAGTTATCCATGACCCCTCATACCAACCCTCAGAGAAAAAGACAGAAATAGACGCGACCAGAAACACATATACTGACATAAGAAGCAAAACGGGTCAACTTCCCCACCCCTCAGGATAGGGCTCCGAAGCCAGCGCTGCTGAATACGCAAACACAACTAACATCCCGCCTAAATAGATCAAAAACAAAATCAGAGATAAAAATGAACCCCCGTGCCCTACTAAAATGCCACAGCCCATTCCTGCTACTGTGACAAGGCCCAAAGCAGCAAAGTAGGGTGAGGGGTTTGAGGCCACGGCCGCTAGGCCTAAAACCAGGCCAACTAATAATAAATAAGTTATATAAACCATAATTCTTGCCAGGATTTTAACCAGGGCCTGCGACTTGAAAAACCACCGTTGTACTCAACTACAAGAACCTAATGGCCAATCTTCGAAAAACCCATCCCCTATTAAAAATCGCAAACGATGCCCTGGTTGATCTCCCAGCCCCGTCAAACATTTCAGTTTGATGAAACTTCGGGTCTCTTCTAGGACTTTGTTTGGCCGCCCAGATTATTACGGGCCTTTTCCTTGCAATACATTATACATCAGACATTGCCACAGCATTTTCATCTGTAGCACATATTTGTCGTGACGTCAACTACGGCTGACTAATCCGAAACATGCATGCAAACGGTGCTTCCTTTTTCTTCATTTGCATCTACCTGCACATCGGACGGGGCTTGTATTATGGATCATACTTATATAAAGAGACATGAAATGTGGGTGTTGTCCTTCTCCTCCTAGTGATAATGACTGCTTTCGTAGGCTACGTCCTACCCTGAGGACAAATGTCATTCTGAGGGGCTACCGTCATTACCAACCTTTTATCAGCCATTCCCTACGTTGGAAACGCCCTAGTTCAATGAATCTGAGGCGGATTTTCAGTAGACAACGCCACCCTTACCCGGTTCTTTGCCTTCCATTTCCTCCTTCCCTTTGTGATTGCTGCTGCTACAGTTGTGCATCTTATCTTCCTCCACGAGACAGGATCGAATAATCCAACAGGTTTAAACTCAGACTCTGACAAAGTATCTTTTCACCCCTACTTTTCTTATAAAGATCTTCTAGGCTTTGCTGCCCTACTAGTAGCCCTTATCTCTTTAGCCCTCTTCTCCCCAAATCTACTGGGAGACCCCGATAACTTTACCCCTGCTAATCCTTTAGTGACGCCACCTCACATCAAACCTGAGTGATACTTCTTGTTCGCTTACGCCATTCTACGATCCATCCCAAACAAACTTGGCGGGGTTCTAGCCCTATTAGCCTCTATTCTAGTCCTCTTTCTTGTTCCCATTCTGCACACATCAAAACAGCGAAGCCTAACATTCCGACCCCTAACCCAATTCCTCTTCTGATTGCTAGTCGCCGATGTAATAATTTTAACCTGAATTGGAGGTATGCCTGTAGAACATCCTTACATTATCATTGGACAAATCGCATCCTTCATTTATTTTTCCCTTTTCCTAGTCATAGCGCCTATGGCCGGCCTACTAGAAAACAAAGTCTTAAAATGACAATGCATTAGAAGCTCAGATGAAAGAGCACCGGTCTTGTAAACCAGAGGTCGAAGGTTCAAGCCCTTCCTAGTGCTCAGAGAGAAGGGATTCTAACCCCTGCCCCTGACTCCCAAAGCCAGGATTCTTAACTAAACTACTCCCTGATTTTCATACACCAGTTTTGTAATCCAGAGCGCATCACTTTTGCCACCAACGTTAAATTAACGTTGCACAAACGTTGCATCAGCGCCCCATGGACACTAAATGACGCGAGGGCGCTAAATAAACACCCCCTACCTCTAGCACCCTTTTAACGATTTCACTTTTTTTTTTTTTTTTGTTTAACGATTACGTTTTTTTTTGCGTTCCCGGACTCTGCCAGATTTCGACCGAAGACTGCCAGAATCCGCTCAAAATCCGCTCAAATACCAATATGTATTATCCCCATAAATGGTTTAAACCATTTTTGCCTAGTACACACTGACCATGCAAGTCAATTATATTTACCCCGCGCTCCAGGCCGCAGTACATACACCTACAGTTGGTGTATTTAGCACAAGTGTGCCTCAGCTAGTTTCAAGTCACCCACATCCTTCCTTTAATTGTTACTTAATGTAGTAAGAGCCCACCATCAGTTGATTCCTTAATGTCAACGGTTCTTGAAGGTGAGGGACAAAAATCGTGGGGGTTTCACTTCTTGAATTATTCCTGGCATTTGGCTCTACATCTCAAGGCCATACATTTCTCGTCTCTCACACTTTCACTGGCCCTGACATTGGTTAATGGTGGAGTACATACTCCTCGTTACCCCCCATGCCGGGCGTTCTTTCTAATGGACAACGGGTTTTCCTTTTTTTTTCCTTTTCACTTGGCATTTCACAGTGCATACAAACCTTGATGACAAGGTTGAACATTTAGAAATCGGCCGCAAAGAATATTGGTGAATTATTCAAAGATATTAACAGATGAATTGCATAAGTGATATCAAGAGCATAAATAACCAAATGAAACTAGGAACGTTTCTATAATATGCCCCCCGGCTCCCGCGCGTCAAACCCCCCTACCCCCCTAAACTAGTAAGAAGTCTATTATTCCTGCAAACCCCCCGGAAACAGGAAACCCCCTACTAGCATTTTAGCCCGCCCAAATTTGTGTGTATTTATATTATTTGTAATATTGCAAAA

>GQ 20

GCTAGTGTAGCTTAACTAAAGCATAACACTGAAGATGTTAAGACAAACCTTAGATTGGTTTCACGAGCACAAAAGTTTGGTCCTGACTTTACTATCAACTTTAGCTAAACTTACACATGCAAGTATCCGCAATCCCGTGAGAATGCCCTACAGTTTCCTTAAAGGAAACAAGGAGCTGGTATCAGGCTCAATTACTCCCGCCCATGACACCTTGCTTAGCCACACCCCCAAGGGAACTCAGCAGTGATAGACATTAAGCAATAAGTGAAAACTTGACTTAATTAAAGCTAAGAGAACCGGTTAAACTCGTGCCAGCCACCGCGGTTATACGAGCGGTTCAAGCTGATAGATTACGGCGTAAAGCGTGGTTAATAAGAATGAAACTAAAGTCGAATGTTTTCAAAGCTGTTATACGCACTCGAAAATTAGAAGGTCAGAAACGAAAGTGACTTTAACCCTATGAACCCACGAAAACTATGAAACAAACTGGGATTAGATACCCCACTATGCATAGCTGTAAACTTTGATGAGCTATTACATTATCATCCGCCTGGGTACTACGAGCATCAGCTTAAAACCCAAAGGACTTGGCGGTGCTTTAGACCCACCTAGAGGAGCCTGTTCTAGAACCGATAACCCCCGTTAAACCTCACCCTCTCTTGTTTTTCCCGCCTATATACCGCCGTCGTCAGCTTACCCTGTGAAGGTCTAATAGTAAGCACAACCAGTTATACTCAAAACGTCAGGTCGAGGTGTAGCATATGAGAGGGGAAGAAATGGGCTACATTCCTTGTTTCAAGGAAAACGGATAACATAATGAAAGGTACGTTAGAAGGAGGATTTAGCAGTAAGCAGCAAATAGAGTGTTCTGCTGAAACTGGCCCTGAAGCGCGCACACACCGCCCGTCACTCTCCCCAACTCCGAGTTAAAAACATATATAAACCTTTGAAGGAACAAAGGGGAGGCAAGTCGTAACATGGTAAGTGTACCGGAAGGTGCACTTGGATAAATCAGAGTATAGCTAAGAAAGAAAAGCATCTCCCTTACACCGAGAAGTCATCCGTGCAAATCGGATTACCCTGACTCTAACAAGCTAGCCCAAAACCTTAACTTAAAAATCAAATATTTCTAGTAATTAATAAACCAAACACATTAAATAAATCATTTTTCCCCCTGAGTATGGGAGACAGAAAAGGATAAAGGAGCTATAGACAAAGTACCGCAAGGGAAAGCTGAAAGAGAAATGAAACAAACCAGTAAAGAAAAACAAAGCAGAGATTAACCCTTGTACCTTTTGCATCATGAATTAGCCAGTTTAATCAAGCAAAGAGCACTGTAGTTTGAGACCCCGAAACTTAGTGAGCTACTTCAAGACAGCCTACGAAATAGGGCAAACCCGTCTCTGTGGCAAAAGAGTGGGAAGATCTTCAAGTAGAGGTAACAGACCTATCGAACTAAGTTATAGCTGGTTGCTCGTGAAATGAATAGAAGTTCAGCCTTTTGCTTTCTAAATTTCGATTTAGCATCACTTAGCCTAAATGATTAGAAAACAAAAGAGTTAGTCAAAGAGGGTACAGCCTGTTTGATAAAAGATACAACTTTACTAGGAGGATAAGAATCATAATTTTAAAGGTTTAATGCCCAGGTGGGCCTAAAAGCAGCCACCCTGATCAATAGCGTTAAAGCTTAAGCATAAAACACACCTACAATTCTGATAAATCAGTTTTAATCCCCTAAAGTTAACGAGCTATTTCATACCTTATGAAAGAAATTATGCTAGTATGAGTAATAAGAAGTTACGAACTTCTCCCTGCACACGTGTAAATCGGAACGGACAAACCACCGAATCTTAACGGCCCCAGTCAAAGAGGGGATGTCGGATAAAAAAAAGAACAAGAAATTCCCGATAAAACCACCGTTGACCCCACACCGGAGTGCTCCCTGGGAAAGACAAAAAGGGACAGAAGGAACTCGGCAAATATGCTCAAGCCTCGCCTGTTTACCAAAAACATCGCCTCTTGTAAAAGTTAAATAAGAGGTACCGCCTGCCCTGTGACTAGTAGTTTAACGGCCGCGGTATTTTGACCGTGCAAAGGTAGCGCAATCACTTGCCTTTTAAATGAAGGCCTGTATGAATGGCACGACGAGGGCTTAACTGTCTCCTCTCCCTAGTCAATGAAATTGATCCCCCCGTGCAGAAGCGGGGATAATAACATAAGACGAGAAGACCCTGTGGAGCTTTAGACTATGAGCAGACCATGTCAAGAATAACAAACAAGTAAATTAAACAAATTGGCCCCTGCTTCCCTGTCTTTGGTTGGGGCGACCGCGGGATAATAAAAAACTCCCACGAGGATTGAGAACCCTTATCTTATAACCAAGAGCTTCTCCTCTAAGTAACAGAACATCTGACCTTAATGATCCGGCCTGGCCGATCAACGGACCGAGTTACCCCAGGGATAACAGCGCAATCCTCTTTTAGAGTCCATATCGACAAGAGGGTTTACGACCTCGATGTTGGATCAGGACATCCTAATGGTGCAGCCGCTATTAAGGGTTTGTTTGTTCAACAATTAAAGTCCTACGTGATCTGAGTTCAGACCGGAGTAATCCAGGTCAGTTTCTATCTATGACGTACTCTCTTCTAGTACGAAAGGACCGAAGAAAGAAGGCCCATGAAAAATTATGCCTTAGTCTCACCTTATGAAGAAAACTAAATAAGACAAGAGGTTACACCCCTTGGTCATAGAAAATGACATGTTAAGGTGGCAGAGCCCGGATATTGCAAAAGACCTAAGCCCTTTCCACAGAGGTTCAATTCCTCTCCTTAACTATGTTCTCAACAATATTAAGCTTCATTATTAATCCCCTGATTGTTATGATTTTTGTTTTGTTGGCAGTAGCCCTCTTGACCTTGGTAGAGCGTAAAGTGCTAAGCTACATGCAACTTCGTAAAGGCCCAAATGTTGTTGGCCCTTACGGCCTTTTGCAACCCTTCGCTGATGGCTTAAAACTTTTCATGAAAGAGCCCGTCCGACCCTCCACCTCCTCGCCCGCCTTGTTCTTAATTACCCCTATTATAGCCCTTACCTTAGCCCTAACCCTCTGAGCCCCCCTTCCTATGCCTTTTCCCGTCACCGACCTAAACTTAGGCATTTTATTTATTTTAGCACTATCGAGCCTGGCAGTATATTCTATTCTTGGCTCCGGATGAGCCTCCAACTCTAAATATGCATTAATTGGTGCTCTTCGAGCGGTCGCCCAAACCATCTCTTATGAAGTAAGCTTGGGCCTTATTCTTCTTAACACAATTGTCTTTACGGGGGGTTTTACTCTTCAAACCTTCAGCACCGCACAAGAAGCCACCTGATTACTTCTACCAGCATGACCACTAGCAGCCATGTGATATATCTCCACACTCGCGGAAACTAACCGGGCCCCTTTCGACCTAACTGAAGGAGAGTCCGAGCTAGTGTCTGGCTTCAACGTAGAGTATGCCGGCGGACCTTTTGCCCTTTTTTTTCTGGCAGAATACGGTAACATTTTACTTATAAATACCCTCTCAGCAGTACTATTTCTAGGCTCTTCAACCTACCACAACTTTCCAGAACTAACCGCGACCTTATTAATGCTTAAAGCCACCCTCCTTTCAGTCGTATTTTTATGAGTGCGAGCATCTTACCCTCGGTTCCGATACGACCAACTAATGCATTTAATTTGAAAAAACTTTTTACCTCTGACCCTAGCGCTAGTTATTTGACACCTTTCTCTTCCAATCACGCTGAGCGGCCTCCCTCCTCAACTTTAACTCAGGAAATGTGCCTGAAAAAGGGTCACTTTGATAGGGTGAATAATGAGGGTTAAAGCCCCTCCATCTCCTTAGAAAGAAGGGGTTTGAACCCTACCTGAAGAGATCAAAACTCTTAGTGCTTCCACTACACCACTTCCTAGTAAAGTCAGCTAATAAAAGCTTTTGGGCCCATACCCCAAATATGTTGGTTAAAATCCTTCCTTTGCTAATGAATCCTTACGTCCTTTCAATTCTACTTATGGGCTTAGGCCTCGGCACTACAGTCACATTCGCTAGCTCACACTGACTCTTAGCATGAATAGGCCTTGAAATAAATACCCTCGCCATTTTGCCATTAATAGCACAACATCACCACCCCCGAGCCGTTGAAGCCACCACCAAGTATTTTTTAATTCAATCGGCAGCCGCAGCAACCATCTTATTTGCCAGCTCAACTAACGCCTGACTTTCGGGCCAGTGGGATATCATAAGTATCAATCACCCCCTTCCAACCGTCATAATTACAATCGCTCTATCCTTAAAACTAGGCTTGGCCCCTCTTCACGCCTGACTTCCCGAAGTTATTCAAGGACTAGACTTGACCACGGGCTTAATCCTCTCCACATGACAAAAACTCGCACCCTTTGCCCTTCTCGTTCAAATCTTCCCCGACACCCCCCTTCTCATCACTTCTCTAGGACTTCTTTCAATATTAATTGGGGGGTGAGGAGGTTTAAACCACACACAACTCCGCAAAGTGCTCGCATATTCTTCGATCGCCCACTTAGGCTGAATAATAGTAATTATGCAATTCTCCACCCCCCTTACAATTCTTGCTTTATCAACATACATTGTTATAACATCATCTACTTTTCTAATCTTTAAACTCCTTAAATCCACAAATATGAACAGCCTGGCAACATCTTGAGCTAAAACCCCCTCCATTACAGCCCTAGCACCTTTAGTGCTATTATCCTTAGGCGGACTTCCTCCCCTCTCGGGCTTTATGCCAAAATGATTAATTATTCAAGAATTAACTAAGCAAGATCTAGCCCTAGTTGCAACCTTGGCCGCCCTCTCTGCGCTACTCAGCCTTTTCTTCTACCTACGCATTTGTTACTCCCTCACATTTACCTCCTCTCCTAATAATCTCATGGGAACACCCCCCTGACGACTAGTAACAAAGCAAGTATCACTTCCCCTAGCTATAACAACCTCCCTCTCTATTCTTCTACTCCCGGTTACCCCTGCAATCTTATCAGTGGTTCTCCCTTTGTAAAGAGGCTTAGGATAGTATTAAGACCAAGGGCCTTCAAAGCCCTAAGCGGGAGTGAAAGCCCCCCAGCCTCTGTAAGACCTACGGGACACTAACCCACATCTTCTGTATGCAAAACAGACACTTTAATTAAGCTAAAGCCTTCCTAGGTGGGTAGGCCTCGATCCTACAATCTCTTAGTTAACAGCTAAGCGCCTAAACCAGCGGGCATCCATCTACCTTTCCCCCGCCTTGCCGAAAAAAAAAGGCGGGGGAAAGCCCCGGCAGGGTATTAGCCTGCCACTTAAGATTTGCAATCTAATGTGTTAACACCTCGGGGCTGGTAAGAAGAGGACTTTAACCTCTGTCCATGGGGCTACAATCCACCGCTAAACGCTCAGCCACCTTACCTGTGGCAATCACACGTTGATTTTTCTCAACTAATCACAAAGACATCGGCACCCTGTATCTAATCTTTGGTGCCTGGGCGGGAATGGTAGGGACGGCCTTAAGTCTACTCATTCGGGCAGAACTAAGTCAACCAGGCTCCCTATTAGGAGACGACCAGATCTATAACGTAATTGTAACTGCACATGCTTTCGTAATAATTTTCTTTATAGTAATGCCAATCATAATTGGGGGGTTTGGCAACTGATTAATCCCTTTAATGATTGGAGCCCCCGACATGGCCTTCCCACGGATAAATAATATAAGCTTTTGACTCCTGCCCCCTTCTTTCCTTCTATTATTGGCCTCATCTGGTGTAGAAGCTGGTGCCGGAACAGGATGAACCGTATATCCCCCCTTGTCAGGTAATTTGGCACACGCAGGGGCCTCCGTAGATTTAACCATTTTCTCTCTTCACCTGGCCGGAATTTCTTCTATCCTAGGGGCCATTAATTTCATCACAACTATTATTAATATAAAACCTCCAGCCATTTCCCAATATCAAACCCCCTTATTTGTGTGGGCTGTACTAATTACCGCAGTATTACTTCTACTCTCTCTTCCTGTTCTAGCTGCAGGTATCACCATGCTTCTCACAGATCGGAACCTAAATACAACATTTTTCGACCCCGCAGGAGGGGGGGACCCCATTCTTTATCAACATTTATTCTGATTCTTTGGGCATCCTGAAGTCTACATTCTAATTTTGCCCGGCTTCGGAATGATTTCTCACATTGTAGCATATTACTCAGGCAAAAAAGAGCCGTTTGGTTACATGGGAATAGTATGAGCTATAATAGCAATTGGCTTGCTGGGCTTTATCGTATGAGCCCATCATATGTTCACTGTAGGGATGGACGTGGACACTCGAGCTTATTTTACTTCCGCCACTATAATTATCGCAATTCCCACAGGAGTCAAAGTGTTTAGTTGACTAGCTACCTTGCATGGGGGCTCAATCAAATGAGAAACCCCCCTGTTATGAGCTCTAGGCTTTATCTTCTTATTTACTGTCGGAGGTTTAACAGGAATTGTTTTAGCCAACTCATCTCTGGACATTATACTTCATGATACATACTATGTTGTAGCCCACTTCCACTATGTCCTCTCTATAGGAGCAGTCTTTGCCATCATGGGAGCATTCGTTCACTGATTTCCCCTATTCTCAGGCTACACCCTTCACAATACGTGAACAAAAATCCACTTCGGAGTTATGTTTGTAGGTGTAAACCTCACCTTTTTCCCTCAGCACTTCTTAGGATTGGCGGGAATACCTCGACGATACTCAGATTACCCTGACGCATACACACTGTGAAATACTATCTCATCCCTGGGGTCATTAATCTCCCTTATTGCTGTAATTATATTCCTATTTATTATCTGGGAGGCATTCGCGGCAAAACGTGAAGTCTTATCAGTTGAACTAACAGCCACAAACGTAGAATGACTGCACGGGTGTCCTCCCCCTTACCATACATTTGAAGAACCTGCATTCGTTCAAATTCAACAATCCAAAATTTAATCGAGAAAGGAAGGAGTCGAACCCCCATAAACTGGTTTCAAGCCAGCCACATAACCGCTCTGTCACTTTCTTCCCTAAGTTAATAAGATTCTAGTTAAAGGAATAACACTGCCTTGTCAAGGCAAAATTGTGGGTTAAAGCCCCACGTATCTTGCTTATGGCACATCCATCTCAACTAGGATTCCAAGATGCAGCTTCACCCGTTATAGAAGAACTTCTCCATTTTCATGACCATGCATTAATAATTGTTTTCTTAATCAGCACCCTTGTTCTTTACATTATTGTGGCTATGGTAACCACCAAGCTAACAAATAAGTTCATTCTGGACTCCCAAGAAATTGAAATCATCTGAACCTTGCTACCAGCAATTATCCTAATTCTGATCGCCCTACCCTCCCTTCGCATTCTTTACCTCATGGATGAAATCAATGACCCCCACCTCACAATTAAAGCCATAGGACATCAATGATACTGAAGCTACGAATATACGGATTATGAAGACCTAGGGTTCGACTCGTATATGGTCCCTACACAAGACCTCGCCCCTGGTCAATTTCGACTACTTGAAACAGACCATCGCATGGTCATTCCTGTTGAGTCCCCCATCCGGGTTCTTGTCTCCGCCGAGGATGTTTTACACTCATGGGCCGTCCCAAGCCTTGGAGTAAAAATGGACGCCGTCCCCGGCCGCCTAAATCAAACAGCCTTCATTACTTCCCGTCCGGGTGTGTTTTATGGACAGTGCTCAGAAATTTGCGGAGCTAATCATAGCTTTATACCCATTGTAGTGGAAGCTGTTCCTCTAGAACACTTCGAGAACTGATCTTACCTAATACTTCAAGATGCCTCACCAGGAAGCTAAAAGGGGATAGCATTAGCCTTTTAAGCTAAAAATTGGTGACTCCCGCCCACCCCTGGTGACATGCCTCAGTTGAACCCCGCACCCTGATTTGCTATTATAGTATTCTCATGACTAGTTTTCCTAGCCGTTATCCCACCTAAAGTTTTAGCTCACCATTTTCCCAATGACCCCGCCCCACAGAGCGTAAAAAAATCAAAAACAGAGACCTGATCCTGACCATGACTTTAAGCCTCTTTGATCAATTTATGAGCCCTACACTTCTAGGGGTGCCTCTTATCGGACTCGCCCTAACATTGCCATGAGTCCTTTACTTCCAACCCGGTGCCCGATGACTTAATAACCGCTTCATTACCCTTCAATCTATATTCATGAACTGATTTGTAAAACAAATCTTTCAGCCAATAAGCTTAGGCGGACACAAATGGGCCGCTCTCCTCATATCTTTAATACTATTTTTAATTACCTTAAATATGCTAGGCCTACTGCCTTATACATTTACTCCAACAACGCAGCTGTCACTTAATATAGCCTTTGCAGTACCACTTTGACTAGCAACTGTCATTATTGGAATACGAAATCAGCCAACACATGCCCTTGGTCACCTCCTCCCCGAAGGAACTCCTACCGCCCTAATCCCGGTTTTAATCGTGATTGAGACAATTAGCCTTTTTATTCGACCATTGGCCCTCGGTGTTCGACTTACCGCAAACTTGACAGCCGGGCACCTTCTAATTCAACTAATTGCAACTGCGGCTTTTGTTCTTTTCCCTATAATGCCCACAGTCGCCGCTCTTACCTCTGTCTTACTATTCTTGCTAACCCTACTAGAAGTCGCCGTGGCCATAATCCAAGCCTATGTATTCGTACTTCTTTTAAGCCTTTACCTACAAGAAAACGTCTAATGGCCCATCAAGCACATGCATATCATATAGTTGACCCAAGCCCTTGACCCCTCACAGGCGCAGTAGCCGCCCTTCTACTTACATCTGGAACAGCAATCTGAATACACTTTAACTCCACGGTTCTCATGTCCCTTGGACTTGTTCTGTTACTACTAACCATATATCAATGATGGCGAGACATTATCCGAGAGGGCACCTTTCAAGGTCATCATACACCCCCTGTTCAAAAAGGCCTTCGGTACGGGATAATTCTGTTTATTACCTCAGAGGTCTTCTTTTTCCTAGGTTTCTTCTGAGCATTTTATCACTCAAGCCTAGCCCCAACCCCCGAACTTGGTGGATGTTGACCACCCATGGGTATTACAACACTGGACCCCTTTGAAGTTCCCCTTCTCAATACTGCTGTCCTTCTCGCCTCCGGTGTCACGGTCACTTGAGCTCACCATAGTATTATGGAGGGGCAGCGAAAACAAGCAATTCAGTCCTTGACACTCACAATTCTCCTGGGGTTTTACTTTACATTCCTTCAAGCAATAGAGTACTACGAGGCACCCTTCACCATTGCAGATGGCGTCTATGGCTCTACATTTTTTGTGGCAACAGGGTTTCATGGCCTCCATGTAATTATTGGATCAACATTTCTGGCAGTCTGCCTCTTACGACAAGTCCAGTTCCATTTTACATCAGAACATCACTTCGGATTCGAAGCTGCAGCATGATACTGACACTTTGTAGACGTAGTCTGACTATTCTTATATATCTCTATCTACTGATGAGGCTCATATCTTTCTAGTATTAAAAAGTACAAGTGACTTCCAATCACTCAGTCTTGGTTAGACTCCAAGGAAAGATAATGAACTTAGTACTAGTCATTATTTGCATCTCATTAGCCCTCACCGCACTGCTCGCAACTGTTTCATTTTTCCTCCCACAAATAACCCCGGATTATGAGAAACTCTCACCGTATGAGTGCGGCTTTGATCCAGTGGGATCCGCCCGTTTGCCATTCTCCATTCGCTTTTTTCTAGTCGCAATCCTATTTCTCCTCTTCGACTTAGAAATTGCCTTACTTCTTCCCCTTCCCTGAGGGGACCAACTTCCCTCCCCTCTAACAACTTTCTTTTGAGCTTCTGCTATTCTTATACTACTAACTCTAGGGTTAATCTATGAATGACTTCAAGGGGGCCTAGAGTGGGCAGAATAGGTACTTAGTTTAATAAAAACATTTGATTTCGGCTCAAAAACTTATGGTTTAAGTCCATATTTACCTGATGACCTTAACTCACTATGCATTCTCGTCAGCCTACTTTGTTAGCTTCATGGGTTTAATTTTTTACCGAAAGCATCTTCTCTCCGCCTTACTTTGCTTAGAAGCGATAATACTTATTCTTTTTATTTCACTATGCCTGTGAGGTCTAGTCTTAGCCTCAAGTGCATTTTCGGCAGGCCCAATGATCTTACTTGCTTTCTCAGCATGTGAAGCAAGTGCAGGCCTAGCACTGCTTGTAGCAATAGCTCGAACCCACGGTACTGACCGTTTAAAAAACCTTAGCCTACTCCAATGTTAATAATTCTTATTCCTACTGTTATGCTTCTACCCACAATCTGACTGACCCCCACTAAATACCTGTGATCCTCAACACTCGGCCATAGCATAATGATTGCTCTTATAAGCCTCTCCTGACTTAGCCTCCCAGGGGAGGTTGGCTGATCTTCCCTTAACACTTTTATAGCAACAGACCCTCTCTCTACCCCCCTTCTCGTACTTACTTGCTGACTTCTGCCCTTAATAATTCTTGCGAGCCAAAACCATATAGCCCAAGAACCTACCAATCGCCAGCGAACCTATATCTCTCTCCTTACTTCCCTTCAAATCTTCTTAATCTTAGCATTTGGGGCAACCGAGATAATCATGTTCTACATTATATTTGAAGCGACCTTAATTCCCACACTCGTAATTATCACACGATGAGGAAACCAAACAGAGCGATTAAACGCAGGTATTTACTTTTTATTTTATACCTTAGCCGGCTCTTTACCACTACTAGTGGCCCTCCTTCTACTTCAGACCTCGACAGGAACTCTTTCTTTTCTAACCACTCAATTTTTTCCCCCTTTACAACTGCATACAGAAGCAAGTAAATTCTGGTGGGCAGGCTGTTTACTAGCATTCTTAGTAAAAATGCCCCTATATGGAGCACACCTTTGACTTCCAAAAGCTCACGTCGAAGCCCCCATCGCCGGGTCAATAGTCCTTGCAGCCGTTCTTTTAAAACTAGGGGGTTACGGTATGATACGAGTTATTATTATCTTAGAGCCCCTCACGAAACAACTCAGCTACCCCTTTATTGTTCTTGCCCTGTGGGGCGTCGTAATAACTGGCTCAATTTGCCTCCGACAAACAGACCTTAAATCACTAATCGCTTACTCCTCAGTAAGCCACATAGGCCTTGTCGCAGCAGGCATCCTAATCCAAACTCCTTGGGGGTTTACAGGAGCATTAATCCTTATAATTGCCCATGGCTTAACTTCCTCCGCCCTATTCTGTTTAGCCAACACTAACTATGAGCGAACACATAGCCGAACCATGCTTTTAGCCCGGGGTCTACAAATGGTCCTTCCTCTCTTAGCAACTTGATGGTTTCTATTTACCCTCGCCAACCTAGCACTCCCTCCACTACCCAACCTCATAGGAGAACTTATGATTATCTCATCCTTGTATAACTGGTCAAACTGGTCTCTAATCCTGACCGGGGCGGGAGTACTAATTACCGCTAGTTACTCTCTCCATATATTCCTAACCACTCAACGTGGCCCTATCACTAACCCCGTCTTGGCAATTGAACCAACCCATACACGAGAACATCTCCTCATAATCCTTCACCTTCTTCCCCTCCTCCTTCTAATTTTAAAACCCTGCTTGATCTGGGGCTGAACAGCTTGTAGGCGTAGTTTAAATAAAGCGCTAGATTGTGATTCTAGAAATAAGAGTTAAACCCTCTTCACCCACCGAGAGGGGTCGCCGTGACAGCAAGAACTGCTAATTCTAGCCCCTTTGGTTAAAGTCCGAAGCCCACTCGAACAGGCTTCTAAAGGATAACAGCTCATCCGTTGGTCTTAGGAACCAAAAACTCTTGGTGCAACTCCAAGTAGCAGCTATGCACTTTACAACAATGATTCTCTCCTCAAGCCTAATAACAATTTTCCTTCTTCTTATCCTTCCAGTCCTAGGTACACTAAATCCTAACCCCACAGGGGACCTGTGGGCCACAAAAAACGTTAAAACAGCAGTTAAGATGGCCTTTTTTGTAAGCCTTCTACCTCTTTTTATCTTTCTTAATGAAGGAGTAGAGACTATTATAACAAACTGAAAATGGATAAATACTCTAATATTTGAAATTAATATCAGCTTTAAATTTGACCTCTACTCTGTGGTATTTACCCCTGTAGCCCTCTACGTAACATGATCAATTTTAGAGTTCGCATCTTGGTACATACACAGTGACCCCAATATAAACCGATTCTTTAAGTATCTTCTAATCTTTCTAATCGCTATGGTTGTTCTGGTTACAGCCAACAACATGTTCCAACTATTTATTGGCTGAGAAGGTGTTGGAATTATGTCTTTCTTACTTATTGGCTGGTGGTTCGGGCGGGCTGACGCCAACACTGCGGCCCTCCAAGCCGTAGTTTATAACCGAGTCGGTGATATCGGCCTAATTCTAGCAATAGCATGAATAGTAGTAAACCTAAACTCATGAGAGATACAACAGCTCTTTTCTGTGTCTAAAGGCCATGACATGACCCTTCCCTTATTAGGCCTAGTACTGGCCGCTACCGGAAAGTCCGCCCAGTTTGGGCTTCACCCCTGGCTCCCCTCAGCCATAGAAGGTCCAACACCGGTCTCTGCCCTCCTGCACTCTAGCACCATGGTTGTTGCTGGTATTTTCCTTCTTATCCGCCTCAGCCCCTTAATGCAAGAAAGCCCGTTAATTCTCTCAACATGCCTTTGCCTGGGGGCCCTAACTACCGTCTTTACTGCGACATGTGCCCTTACCCAAAATGACATCAAAAAAATTGTTGCATTTTCTACATCAAGTCAATTAGGACTAATAATAGTTACCATCGGACTAGGCCAGCCCCAGCTCGCCTTTCTTCATATCTGCACCCACGCCTTCTTTAAAGCAATACTTTTCTTATGTTCAGGCTCCATCATTCATAGCCTTAATGATGAACAAGATATCCGAAAAATAGGAGGACTTCACAAGCTCCTTCCACTGACCTCTTCTTGTCTAACCATTGGCAGCCTAGCTCTAACAGGAGTCCCCTTTTTAGCAGGCTTCTTTTCCAAAGACGCCATCATTGAAGCTATAAATACATCCTACCTTAACGCCTGGGCCCTAATTTTAACGCTTCTAGCTACATCATTTACCGCAGTTTACAGTCTCCGAGTCGTATTCTTTGCCTCTATGGGCCACCCGCGTTTTAATCCAATCTCCCCAATTAATGAAAATAACCCTACAGTAATAAACCCTATTAAACGACTCGCTTGAGGAAGCATTTTGGCAGGGTTGCTAATTACGGCCAATATTGTTCCACTTAAAACCCCCGTTTTAACCATGCCTTTCACCTTAAAAATGGCCGCACTGGCTGTAACAATTATAGGACTACTCACAGCCTTAGAACTAGCATCTCTCACGTCCCAACAATTTAAAATCAAACCCTTATCTTCTACTCACCACTTCTCAAATATATTAGGATTTTTCCCGAGTGTAGCCCATCGACTAGTCCCAAAAACGGGCCTGATTCTTGGGCAACTGGTTGCCAATCAGACAATTGACCAAACCTGACTAGAGAAAACCGGGCCAAAAATAGTAGCCTCCGTTAACCTTCCAGTGGCTACTTCAATTAGCAACCTACAGCAGGGTGTAATTAAGACCTACTTCTTATTATTTTTCTTCACCATAATACTAGCAATTCTCATCCTTGTCATCTAACTGCCCGTAAGGTCCCCCGACTTAGCCCTCGAGTTAACTCCAGAACTACAAAAAGCGTCAGTAATAAAACTCATCCCCCAAGCATTAAAACTCCTCCTCCTGAAGAATATATCAGAGCAACCCCCCCAAGATCCCCCCGGAATAGCATGAATTCACTAAACTCGTCAGCAGTTATCCATGACCCCTCATACCAACCCTCAGAGAAAAAGACAGAGATAGACGCGACCAGAAACACATATACTGACATAAGAAGCAAAACGGGTCAACTTCCCCACCCCTCAGGATAGGGCTCCGAAGCCAGCGCTGCTGAATACGCAAACACAACTAACATCCCGCCTAAATAGATCAAAAACAAAATCAGAGATAAAAATGAACCCCCGTGCCCTACTAAAATGCCACAGCCCATTCCTGCTACTGTGACAAGGCCCAAAGCAGCAAAGTAGGGTGAGGGGTTTGAGGCCACGGCCGCTAGGCCTAAAACCAGGCCAACTAATAATAAATAAGTTATATAAACCATAATTCTTGCCAGGATTTTAACCAGGGCCTGCGACTTGAAAAACCACCGTTGTACTCAACTACAAGAACCTAATGGCCAATCTTCGAAAAACCCATCCCCTATTAAAAATCGCAAACGATGCCCTGGTTGATCTCCCAGCCCCGTCGAACATTTCAGTTTGATGAAACTTCGGGTCTCTTCTAGGACTTTGTTTGGCCGCCCAGATTATTACGGGCCTTTTCCTTGCAATACATTATACATCAGACATTGCCACAGCATTTTCATCTGTAGCACATATTTGTCGTGACGTCAACTACGGCTGACTAATCCGAAACATGCATGCAAACGGTGCTTCCTTTTTCTTCATTTGCATCTACCTGCACATCGGACGGGGCTTGTATTATGGATCATACTTATATAAAGAGACATGAAATGTGGGTGTTGTCCTTCTCCTCCTAGTGATAATGACTGCTTTCGTAGGCTACGTCCTACCCTGAGGACAAATGTCATTCTGAGGGGCTACCGTCATTACCAACCTTTTATCAGCCATTCCCTACGTTGGAAACGCCCTAGTTCAATGAATCTGAGGCGGGTTTTCAGTAGACAACGCCACCCTTACCCGGTTCTTTGCCTTCCATTTCCTCCTTCCCTTTGTGATTGCTGCTGCTACAGTTGTACATCTTATCTTCCTCCACGAGACAGGATCGAATAATCCAACAGGTTTAAACTCAGACTCTGACAAAGTATCTTTTCACCCCTACTTTTCTTATAAAGATCTTCTAGGCTTTGCTGCCCTACTAGTAGCCCTTATCTCTTTAGCCCTCTTCTCCCCAAATCTACTGGGAGACCCCGATAACTTTACCCCTGCTAATCCTTTAGTGACGCCACCTCACATCAAACCTGAGTGATACTTCTTGTTCGCTTACGCCATTCTACGATCCATCCCAAACAAACTTGGCGGGGTTCTAGCCCTATTAGCCTCTATTCTAGTCCTCTTTCTTGTTCCCATTCTGCACACATCAAAACAGCGAAGCCTAACATTCCGACCCCTAACCCAATTCCTCTTCTGATTGCTAGTCGCCGATGTAATAATTTTAACCTGAATTGGAGGTATGCCTGTAGAACATCCTTACATTATCATTGGACAAATCGCATCCTTCATTTATTTTTCCCTTTTCCTAGTCATAGCGCCTATGGCCGGCCTACTAGAAAACAAAGTCTTAAAATGACAATGCATTAGAAGCTCAGATGAAAGAGCACCGGTCTTGTAAACCAGAGGTCGAAGGTTCAAGCCCTTCCTAGTGCTCAGAGAGAAGGGATTCTAACCCCTGCCCCTGACTCCCAAAGCCAGGATTCTTAACTAAACTACTCCCTGATTTTCATACACCAGTTTTGTAATCCAGAGCGCATCACTTTTGCCACCAACGTTAAATTAACGTTGCACAAACGTTGCATCAGCGCCCCATGGACACTAAATGACGCGAGGGCGCTAAATAAACACCCCCTACCTCTAGCACCCTTTTAACGATTTCACTTTTTTTTTTTTTTTTGTTTAACGATTACGTTTTTTTTTGCGTTCCCGGACTCTGCCAGATTTCGACCGAAGACTGCCAGAATCCGCTCAAAATCCGCTCAAATACCAATATGTATTATCCCCATAAATGGTTTAAACCATTTTTGCCTAGTACACACTGACCATGCAAGTCAATTATATTTACCCCGCGCTCCAGGCCGCAGTACATACACCTACAGTTGGTGTATTTAGCACAAGTGTGCCTCAGCTAGTTTCAAGTCACCCACATCCTTCCTTTAATTGTTACTTAATGTAGTAAGAGCCCACCATCAGTTGATTCCTTAATGTCAACGGTTCTTGAAGGTGAGGGACAAAAATCGTGGGGGTTTCACTTCTTGAATTATTCCTGGCATTTGGCTCTACATCTCAAGGCCATACATTTCTCGTCTCTCACACTTTCACTGGCCCTGACATTGGTTAATGGTGGAGTACATACTCCTCGTTACCCCCCATGCCGGGCGTTCTTTCTAATGGACAACGGGTTTTCCTTTTTTTTTCCTTTTCACTTGGCATTTCACAGTGCATACAAACCTTGATGACAAGGTTGAACATTTAGAAATCGGCCGCAAAGAATATTGGTGAATTATTCAAAGATATTAACAGATGAATTGCATAAGTGATATCAAGAGCATAAATAACCAAATGAAACTAGGAACGTTTCTATAATATGCCCCCCGGCTCCCGCGCGTCAAACCCCCCTACCCCCCTAAACTAGTAAGAAGTCTATTATTCCTGCAAACCCCCCGGAAACAGGAAACCCCCTACTAGCATTTTAGCCCGCCCAAATTTGTGTGTATTTATATTATTTGTAATATTGCAAAA

>GQ 21

GCTAGTGTAGCTTAACTAAAGCATAACACTGAAGATGTTAAGACAAACCTTAGATTGGTTTCACGAGCACAAAAGTTTGGTCCTGACTTTACTATCAACTTTAGCTAAACTTACACATGCAAGTATCCGCAATCCCGTGAGAATGCCCTACAGTTTCCTTAAAGGAAACAAGGAGCTGGTATCAGGCTCAATTACTCCCGCCCATGACACCTTGCTTAGCCACACCCCCAAGGGAACTCAGCAGTGATAGACATTAAGCAATAAGTGAAAACTTGACTTAATTAAAGCTAAGAGAACCGGTTAAACTCGTGCCAGCCACCGCGGTTATACGAGCGGTTCGAGCTGATAGATTACGGCGTAAAGCGTGGTTAATAAGAATGAAACTAAAGTCGAATGTTTTCAAAGCTGTTATACGCACTCGAAAATTAGAAGGTCAGAAACGAAAGTGACTTTAACCCTATGAACCCACGAAAACTATGAAACAAACTGGGATTAGATACCCCACTATGCATAGCTGTAAACTTTGATGAGCTATTACATTATCATCCGCCTGGGTACTACGAGCATCAGCTTAAAACCCAAAGGACTTGGCGGTGCTTTAGACCCACCTAGAGGAGCCTGTTCTAGAACCGATAACCCCCGTTAAACCTCACCCTCTCTTGTTTTTCCCGCCTATATACCGCCGTCGTCAGCTTACCCTGTGAAGGTCTAATAGTAAGCACAACCAGTTATACTCAAAACGTCAGGTCGAGGTGTAGCATATGAGAGGGGAAGAAATGGGCTACATTCCTTGTTTCAAGGAAAACGGATAACATAATGAAAGGTACGTTAGAAGGAGGATTTAGCAGTAAGCAGCAAATAGAGTGTTCTGCTGAAACTGGCCCTGAAGCGCGCACACACCGCCCGTCACTCTCCCCAACTCCGAGTTAAAAACATATATAAACCTTTGAAGGAACAAAGGGGAGGCAAGTCGTAACATGGTAAGTGTACCGGAAGGTGCACTTGGATAAATCAGAGTATAGCTAAGAAAGAAAAGCATCTCCCTTACACCGAGAAGTCATCCGTGCAAATCGGATTACCCTGACTCTAACAAGCTAGCCCAAAACCTTAACTTAAAAATCAAATATTTCTAGTAATTAATAAACCAAACACATTAAATAAATCATTTTTCCCCCTGAGTATGGGAGACAGAAAAGGATAAAGGAGCTATAGACAAAGTACCGCAAGGGAAAGCTGAAAGAGAAATGAAACAAACCAGTAAAGAAAAACAAAGCAGAGATTAACCCTTGTACCTTTTGCATCATGAATTAGCCAGTTTAATCAAGCAAAGAGCACTGTAGTTTGAGACCCCGAAACTTAGTGAGCTACTTCAAGACAGCCTACGAAATAGGGCAAACCCGTCTCTGTGGCAAAAGAGTGGGAAGATCTTCAAGTAGAGGTGACAGACCTATCGAACTAAGTAATAGCTGGTTGCTCGTGAAATGAATAGAAGTTCAGCCTTTTGCTTTCTAAATTTCGATTTAGCATCACTTAGCCTAAATGATTAGAAAACAAAAGAGTTAGTCAAAGAGGGTACAGCCTGTTTGATAAAAGATACAACTTTACTAGGAGGATAAGAATCATAATTTTAAAGGTTTAATGCCCAGGTGGGCCTAAAAGCAGCCACCCTGATCAATAGCGTTAAAGCTTAAGCATAAAACACACCTACAATTCTGATAAATCAGTTTTAATCCCCTAAAGTTAACGAGCTATTTCATACCTTATGAAAGAAATTATGCTAGTATGAGTAATAAGAAGTTACGAACTTCTCCCTGCACACGTGTAAATCGGAACGGACAAACCACCGAATCTTAACGGCCCCAGTCAAAGAGGGGATGTCGGATAAAAAAAAAGAACAAGAAATTCCCGATAAAACCACCGTTGACCCCACACCGGAGTGCTCCCTGGGAAAGACAAAAAGGGACAGAAGGAACTCGGCAAATATGCTCAAGCCTCGCCTGTTTACCAAAAACATCGCCTCTTGTAAAAGTTAAATAAGAGGTACCGCCTGCCCTGTGACTAGTAGTTTAACGGCCGCGGTATTTTGACCGTGCAAAGGTAGCGCAATCACTTGCCTTTTAAATGAAGGCCTGTATGAATGGCACGACGAGGGCTTAACTGTCTCCTCTCCCTAGTCAATGAAATTGATCCCCCCGTGCAGAAGCGGGGATAATAACATAAGACGAGAAGACCCTGTGGAGCTTTAGACTATGAGCAGACCATGTCAAGAATAACAAACAAGTAAATTAAACAAATTGGCCCCTGCTTCCCTGTCTTTGGTTGGGGCGACCGCGGGATAATAAAAAACTCCCACGAGGATTGAGAACCCTTATCTTATAACCAAGAGCTTCTCCTCTAAGTAACAGAACATCTGACCTTAATGATCCGGCCTGGCCGATCAACGGACCGAGTTACCCCAGGGATAACAGCGCAATCCTCTTTTAGAGTCCATATCGACAAGAGGGTTTACGACCTCGATGTTGGATCAGGACATCCTAATGGTGCAGCCGCTATTAAGGGTTTGTTTGTTCAACAATTAAAGTCCTACGTGATCTGAGTTCAGACCGGAGTAATCCAGGTCAGTTTCTATCTATGACGTACTCTCTTCTAGTACGAAAGGACCGAAGAAAGAAGGCCCATGAAAAATTATGCCTTAGTCTCACCTTATGAAGAAAACTAAATAAGACAAGAGGTTACACCCTTTGGTCATAGAAAATGACATGTTAAGGTGGCAGAGCCCGGATATTGCAAAAGACCTAAGCCCTTTCCACAGAGGTTCAATTCCTCTCCTTAACTATGTTCTCAACAATATTAAGCTTCATTATTAATCCCCTGATTGTTATGATTTTTGTTTTGTTGGCAGTAGCCCTCTTGACCTTGGTAGAGCGTAAAGTGCTAAGCTACATGCAACTTCGTAAAGGCCCAAATGTTGTTGGCCCTTACGGCCTTTTGCAACCCTTCGCTGATGGCTTAAAACTTTTCATGAAAGAGCCCGTCCGACCCTCCACCTCCTCGCCCGCCTTGTTCTTAATTACCCCTATTATAGCCCTTACCTTAGCCCTAACCCTCTGAGCCCCCCTTCCTATGCCTTTTCCCGTCACCGACCTAAACTTAGGCATTTTATTTATTTTAGCACTATCGAGCCTGGCAGTATATTCTATTCTTGGCTCCGGATGAGCCTCCAATTCTAAATATGCATTAATTGGTGCTCTTCGAGCGGTCGCCCAAACCATCTCTTATGAAGTAAGCTTGGGCCTTATTCTTCTTAACACAATTGTCTTTACGGGGGGTTTTACTCTTCAAACCTTCAGCACCGCACAAGAAGCCACCTGATTACTTCTACCAGCATGACCACTAGCAGCCATGTGATATATCTCCACACTCGCGGAAACTAACCGGGCCCCTTTCGACCTAACTGAAGGAGAGTCCGAACTAGTGTCTGGCTTCAACGTAGAGTATGCCGGCGGACCTTTTGCCCTTTTTTTTCTGGCAGAATACGGTAACATTTTACTTATAAATACCCTCTCAGCAGTACTATTTCTAGGCTCTTCAACCTACCACAACTTTCCAGAACTAACCGCGACCTTATTAATGCTTAAAGCCACCCTCCTTTCAGTCGTATTTTTATGAGTGCGAGCATCTTACCCTCGGTTCCGATACGACCAACTAATGCATTTAATTTGAAAAAACTTTTTACCTCTGACCCTAGCGCTAGTTATTTGACACCTTTCTCTTCCAATCACGTTGAGCGGCCTCCCTCCTCAACTTTAACTCAGGAAATGTGCCTGAAAAAGGGTCACTTTGATAGGGTGAATAATGAGGGTTAAAGCCCCTCCATCTCCTTAGAAAGAAGGGGTTTGAACCCTACCTGAAGAGATCAAAACTCTTAGTGCTTCCACTACACCACTTCCTAGTAAAGTCAGCTAATAAAAGCTTTTGGGCCCATACCCCAAATATGTTGGTTAAAATCCTTCCTTTGCTAATGAATCCTTACGTCCTTTCAATTCTACTTATGGGCTTAGGCCTCGGCACTACAGTCACATTCGCTAGCTCACACTGACTCTTAGCATGAATAGGCCTTGAAATAAATACCCTCGCCATTTTGCCATTAATAGCACAACATCACCACCCCCGAGCCGTTGAAGCCACCACCAAGTATTTTTTAATTCAATCGGCAGCCGCAGCAACCATCTTATTTGCCAGCTCAACTAACGCCTGACTTTCGGGCCAGTGGGATATCATAAGTATCAATCACCCCCTTCCAACCGTCATAATTACAATCGCTCTGTCCTTAAAACTAGGCTTGGCCCCTCTTCACGCCTGACTTCCCGAAGTTATTCAAGGACTAGACTTGACCACGGGCTTAATCCTCTCCACATGACAAAAACTCGCACCCTTTGCCCTTCTCGTTCAAATCTTCCCCGACACCCCCCTTCTCATCACTTCTCTAGGACTTCTTTCAATATTAATTGGGGGGTGAGGAGGTTTAAACCACACACAACTCCGCAAAGTGCTCGCATATTCTTCGATCGCCCACTTAGGCTGAATAATAGTAATTATGCAATTCTCCACCCCCCTTACAATTCTTGCTTTATCAACATACATTGTTATAACATCATCTACTTTTCTAATCTTTAAACTCCTTAAATCCACAAATATGAACAGCCTGGCAACATCTTGAACTAAAACCCCCTCCATTACAGCCCTAGCACCTTTAGTGCTATTATCCTTAGGCGGACTTCCTCCCCTCTCGGGCTTTATGCCAAAATGATTAATTATTCAAGAATTAACTAAGCAAGATCTAGCCCTAGTTGCAACCTTGGCCGCCCTCTCTGCGCTACTCAGCCTTTTCTTCTACCTACGCATTTGTTACTCCCTCACATTTACCTCCTCTCCTAATAATCTCATGGGAACACCCCCCTGACGACTAGTAACAAAGCAAGTATCACTTCCCCTAGCTATAACAACCTCCCTCTCTATTCTTCTACTCCCGGTTACCCCTGCAATCTTATCAGTGGTTCTCCCTTTGTAAAGAGGCTTAGGATAGTATTAAGACCAAGGGCCTTCAAAGCCCTAAGCGGGAGTGAAAGCCCCCCAGCCTCTGTAAGACCTACGGGACACTAACCCACATCTTCTGTATGCAAAACAGACACTTTAATTAAGCTAAAGCCTTCCTAGGTGGGTAGGCCTCGATCCTACAATCTCTTAGTTAACAGCTAAGCGCCTAAACCAGCGGGCATCCATCTACCTTTCCCCCGCCTTGCCGAAAAAAAAAGGCGGGGGAAAGCCCCGGCAGGGTATTAGCCTGCCACTTAAGATTTGCAATCTAATGTGTTAACACCTCGGGGCTGGTAAGAAGAGGACTTTAACCTCTGTCCATGGGGCTACAATCCACCGCTAAACGCTCAGCCACCTTACCTGTGGCAATCACACGTTGATTTTTCTCAACTAATCACAAAGACATCGGCACCCTGTATCTAATCTTTGGTGCCTGGGCGGGAATGGTAGGGACGGCCTTAAGTCTACTCATTCGGGCAGAACTAAGTCAACCAGGCTCCCTATTAGGAGACGACCAGATCTATAACGTAATTGTAACTGCACATGCTTTCGTAATAATTTTCTTTATAGTAATGCCAATCATAATTGGGGGGTTTGGCAACTGATTAATCCCTTTAATGATTGGAGCCCCCGACATGGCCTTCCCACGGATAAATAATATAAGCTTTTGACTCCTGCCCCCTTCTTTCCTTCTATTATTGGCCTCATCTGGTGTAGAAGCTGGTGCCGGAACAGGATGAACCGTATATCCCCCCTTGTCAGGTAATTTGGCACACGCAGGGGCCTCCGTAGATTTAACCATTTTCTCTCTTCACCTGGCCGGAATTTCTTCTATCCTAGGGGCCATTAATTTCATCACAACTATTATTAATATAAAACCTCCAGCCATTTCCCAATATCAAACCCCTTTATTTGTGTGGGCTGTACTAATTACCGCAGTATTACTTCTACTCTCTCTTCCTGTTCTAGCTGCAGGTATCACCATGCTTCTCACAGATCGGAACCTAAATACAACATTTTTCGACCCCGCAGGAGGGGGGGACCCCATTCTTTATCAACATTTATTCTGATTCTTTGGGCATCCTGAAGTCTACATTCTAATTTTGCCCGGCTTCGGAATGATTTCTCACATTGTAGCATATTACTCAGGCAAAAAAGAGCCGTTTGGTTACATGGGAATAGTATGAGCTATAATAGCAATTGGCTTGCTGGGCTTTATCGTATGAGCCCATCATATGTTCACTGTAGGGATGGACGTGGACACTCGAGCTTATTTTACTTCCGCCACTATAATTATCGCAATTCCCACAGGAGTCAAAGTGTTTAGTTGACTAGCTACCTTGCATGGGGGCTCAATCAAATGAGAAACCCCCCTGTTATGAGCTCTAGGCTTTATCTTCTTATTTACTGTCGGAGGTTTAACAGGAATTGTTTTAGCCAACTCATCTCTGGACATTATACTTCATGATACATACTATGTTGTAGCCCACTTCCACTATGTCCTCTCTATAGGAGCAGTCTTTGCCATCATGGGAGCATTCGTTCACTGATTTCCCCTATTCTCAGGCTACACCCTTCACAATACGTGAACAAAAATCCACTTCGGAGTTATGTTTGTAGGTGTAAACCTCACCTTTTTCCCTCAGCACTTCTTAGGATTGGCGGGAATACCTCGACGATACTCAGATTACCCTGACGCATACACACTGTGAAATACTATCTCATCCCTGGGGTCATTAATCTCCCTTATTGCTGTAATTATATTCCTATTTATTATCTGGGAGGCATTCGCGGCAAAACGTGAAGTCTTATCAGTTGAACTAACAGCCACAAACGTAGAATGACTGCACGGGTGTCCTCCCCCTTACCATACATTTGAAGAACCTGCATTCGTTCAAATTCAACAATCCAAATTTTAATCGAGAAAGGAAGGAGTCGAACCCCCATAAACTGGTTTCAAGCCAGCCACATAACCGCTCTGTCACTTTCTTCCCTAAGTTAATAAGATTCTAGTTAAAGGAATAACACTGCCTTGTCAAGGCAAAATTGTGGGTTAAAGCCCCACGTATCTTGCTTATGGCACATCCATCTCAACTAGGATTCCAAGATGCAGCTTCACCCGTTATAGAAGAACTTCTCCATTTTCATGACCATGCATTAATAATTGTTTTCTTAATCAGCACCCTTGTTCTTTACATTATTGTGGCTATGGTAACCACCAAGCTAACAAATAAGTTCATTCTGGACTCCCAAGAAATTGAAATCATCTGAACCTTGCTACCAGCAATTATCCTAATTCTGATCGCCCTACCCTCCCTTCGCATTCTCTACCTCATGGATGAAATCAATGACCCCCACCTCACAATTAAAGCCATAGGACATCAATGATACTGAAGCTACGAATATACGGATTATGAAGACCTAGGGTTCGACTCGTATATGGTCCCTACACAAGACCTCGCCCCTGGTCAATTTCGACTACTTGAAACAGACCATCGCATGGTCATTCCTGTTGAGTCCCCCATCCGGGTTCTTGTCTCCGCCGAGGATGTTCTACACTCATGGGCCGTCCCAAGCCTTGGAGTAAAAATGGACGCCGTCCCCGGCCGCCTAAATCAAACAGCCTTCATTACTTCCCGTCCGGGTGTGTTTTATGGACAATGCTCAGAAATTTGCGGAGCTAATCATAGCTTTATACCCATTGTAGTGGAAGCTGTTCCTCTAGAACACTTCGAGAACTGATCTTACCTAATACTTCAAGATGCCTCACCAGGAAGCTAAAAGGGGATAGCATTAGCCTTTTAAGCTAAAAATTGGTGACTCCCGCCCACCCCTGGTGACATGCCTCAGTTGAACCCCGCACCCTGATTTGCTATTATAGTATTCTCATGACTAGTTTTCCTAGCCGTTATCCCACCTAAAGTTTTAGCTCACCATTTTCCCAATGACCCCGCCCCACAAAGCGTAAAAAAATCAAAAACAGAGACCTGATCCTGACCATGACTTTAAGCCTCTTTGATCAATTTATGAGCCCTACACTTCTAGGGGTGCCTCTTATCGGACTCGCCCTAACATTGCCATGAGTCCTTTACTTCCAACCCGGTGCCCGATGACTTAATAACCGCTTCATTACCCTTCAATCTATATTCATGAACTGATTTGTAAAACAAATCTTTCAGCCAATAAGCTTAGGCGGACACAAATGGGCCGCTCTCCTCATATCTTTAATACTATTTTTAATTACCTTAAATATGCTAGGCCTACTGCCTTATACATTTACTCCAACAACGCAGCTGTCACTTAATATAGCCTTTGCAGTACCACTTTGACTAGCAACTGTCATTATTGGAATACGAAATCAGCCAACACATGCCCTTGGTCACCTCCTCCCCGAAGGAACTCCTACCGCCCTAATCCCGGTTTTAATCGTGATTGAGACAATTAGCCTTTTTATTCGACCATTGGCCCTCGGTGTTCGACTTACCGCAAACTTGACAGCCGGACACCTTCTAATTCAACTAATTGCAACTGCGGCTTTTGTTCTTTTCCCTATAATGCCCACAGTCGCCGCTCTTACCTCTGTCTTACTATTGTTGCTAACCCTACTAGAAGTCGCCGTGGCCATAATCCAAGCCTATGTATTCGTACTTCTTTTAAGCCTTTACCTACAAGAAAACGTCTAATGGCCCATCAAGCACATGCATATCATATAGTTGACCCAAGCCCTTGACCCCTCACAGGCGCAGTAGCCGCCCTTCTACTTACATCTGGAACAGCAATCTGAATACACTTTAACTCCACGGTTCTCATGTCCCTTGGACTTGTTCTGTTACTACTAACCATATATCAATGATGGCGAGACATTATCCGAGAGGGCACCTTTCAAGGTCATCATACACCCCCTGTTCAAAAAGGCCTTCGGTACGGGATAATTCTGTTTATTACCTCAGAGGTCTTCTTTTTCCTAGGTTTCTTCTGAGCATTTTATCACTCAAGCCTAGCCCCAACCCCCGAACTTGGTGGATGTTGACCACCCATGGGTATTACAACACTGGACCCCTTTGAAGTTCCCCTTCTCAATACTGCTGTCCTTCTCGCCTCCGGTGTCACGGTCACTTGAGCTCACCATAGTATTATGGAGGGGCAGCGAAAACAAGCAATTCAGTCCTTGACACTCACAATTCTCCTGGGGTTTTACTTTACATTCCTTCAAGCAATAGAGTACTACGAGGCACCCTTCACCATTGCAGATGGCGTCTATGGCTCTACATTTTTTGTGGCAACAGGGTTTCATGGCCTCCATGTAATTATTGGGTCAACATTTCTGGCAGTCTGCCTCTTACGACAAGTCCAGTTCCATTTTACATCAGAACATCACTTCGGATTCGAAGCTGCAGCATGATACTGACACTTTGTAGACGTAGTCTGACTATTCTTATATATCTCTATCTACTGATGAGGCTCATATCTTTCTAGTATTAAAAAGTACAAGTGACTTCCAATCACTCAGTCTTGGTTAGACTCCAAGGAAAGATAATGAACTTAGTACTAGTCATTATTTGCATCTCATTAGCCCTCGCCGCACTGCTCGCAACTGTTTCATTTTTCCTCCCACAAATAACCCCTGATTATGAGAAACTCTCACCGTATGAGTGCGGCTTTGATCCAGTGGGATCCGCCCGTTTGCCATTCTCCATTCGCTTTTTTCTAGTCGCAATCCTATTTCTCCTCTTCGACTTAGAAATTGCCTTACTTCTTCCCCTTCCCTGAGGGGACCAACTTCCCTCCCCTCTAACAACTTTCTTTTGAGCTTCTGCTATTCTTATACTACTAACTCTAGGGTTAATCTATGAATGACTTCAAGGGGGCCTAGAGTGGGCAGAATAGGTACTTAGTTTAATAAAAACATTTGATTTCGGCTCAAAAACTTATGGTTTAAGTCCATATTTACCTGATGACCTTAACTCACTATGCATTCTCGTCAGCCTACTTTGTTAGCTTCATGGGTTTAATTTTTTACCGAAAGCATCTTCTCTCCGCCTTACTTTGCTTAGAAGCGATAATACTTATTCTTTTTATTTCACTATGCCTGTGAGGTCTAGTCTTAGCCTCAAGTGCATTTTCGGCAGGCCCAATGATCTTACTTGCTTTCTCAGCATGTGAAGCAAGTGCAGGCCTAGCACTGCTTGTAGCAATAGCTCGAACCCACGGTACTGACCGTTTAAAAAACCTTAGCCTACTCCAATGTTAATAATTCTTATTCCTACTGTTATGCTTCTACCCACAATCTGACTGAGCCCCACTAAATACCTGTGATCCTCAACACTCGGCCATAGCATAATGATTGCTCTTATAAGCCTCTCCTGACTTAGCCTCCCAGGGGAGGTTGGCTGATCTTCCCTTAACACTTTTATAGCAACAGACCCTCTCTCTACCCCCCTTCTCGTACTTACTTGCTGACTTCTGCCCTTAATAATTCTTGCGAGCCAAAACCATATAGCCCAAGAACCTACCAATCGCCAGCGAACCTATATCTCTCTCCTTACTTCCCTTCAAATCTTCTTAATCTTAGCATTTGGGGCAACCGAGATAATCATGTTCTACATTATATTTGAAGCGACCTTAATTCCCACACTCGTAATTATCACACGATGAGGAAACCAAACAGAACGATTAAACGCAGGTATTTACTTTTTATTTTATACCTTAGCCGGCTCTTTACCACTACTAGTGGCCCTCCTTCTACTTCAGACCTCGACAGGAACTCTTTCTTTTCTAACCACTCAATTTTTTCCCCCTTTACAACTGCATACAGAAGCAAGTAAATTCTGGTGGGCAGGCTGTTTACTAGCATTCTTAGTAAAAATGCCCCTATATGGAGCACACCTTTGACTTCCAAAAGCTCACGTCGAAGCCCCCATCGCCGGGTCAATAGTCCTTGCAGCCGTTCTTTTAAAACTAGGGGGTTACGGTATGATACGAGTTATTATTATCTTAGAGCCCCTCACGAAACAACTCAGCTACCCCTTTATTGTTCTTGCCCTGTGGGGCGTCGTAATAACTGGCTCAATTTGCCTCCGACAAACAGACCTTAAATCACTAATCGCTTACTCCTCAGTAAGCCACATAGGCCTTGTCGCAGCAGGCATCCTAATCCAAACTCCTTGGGGGTTTACAGGAGCATTAATCCTTATAATTGCCCATGGCTTAACTTCCTCCGCCCTATTCTGTTTAGCCAACACTAACTATGAGCGAACACATAGCCGAACCATGCTTTTAGCCCGGGGTCTACAAATGGTCCTTCCTCTCTTAGCAACTTGATGGTTTCTATTTACCCTCGCCAACCTAGCACTCCCTCCACTACCCAACCTCATAGGAGAACTTATGATTATCTCATCCTTGTATAACTGGTCAAACTGGTCTCTAATCCTGACCGGGGCGGGAGTACTAATTACCGCTAGTTACTCTCTCCATATATTCCTAACCACTCAACGTGGCCCTATTACTAACCCCGTCTTGGCAATTGAACCAACCCATACACGAGAACATCTCCTCATAATCCTTCACCTTCTTCCCCTCCTCCTTCTAATTTTAAAACCCTGCTTGATCTGGGGCTGAACAGCTTGTAGGCGTAGTTTAAATAAAGCGCTAGATTGTGATTCTAGAAATAAGAGTTAAACCCTCTTCACCCACCGAGAGGGGTCGCCGTGACAGCAAGAACTGCTAATTCTAGCCCCTTTGGTTAAAGTCCGAAGCCCACTCGAACAGGCTTCTAAAGGATAACAGCTCATCCGTTGGTCTTAGGAACCAAAAACTCTTGGTGCAACTCCAAGTAGCAGCTATGCACTTTACAACAATGATTCTCTCCTCAAGCCTAATAACAATTTTCCTTCTTCTTATCCTTCCAGTCCTAGGTACACTAAATCCTAACCCCACAGGGGACCTGTGGGCCACAAAAAACGTTAAAACAGCAGTTAAGATGGCCTTTTTTGTAAGCCTTCTACCTCTTTTTATCTTTCTTAATGAAGGAGTAGAGACTATTATAACAAACTGAAAATGGATAAATACTCTAATATTTGAAATTAATATCAGCTTTAAATTTGACCTCTACTCTGTGGTATTTACCCCTGTAGCCCTCTACGTAACATGATCAATTTTAGAGTTCGCATCTTGGTACATACACAGTGACCCCAATATAAACCGATTCTTTAAGTATCTTCTAATCTTTCTAATCGCTATGGTTGTTCTGGTTACAGCCAACAACATGTTCCAACTATTTATTGGCTGAGAAGGTGTTGGAATTATGTCCTTCTTACTTATTGGCTGGTGGTTCGGGCGGGCTGACGCCAACACTGCGGCCCTCCAAGCCGTAGTTTATAACCGAGTCGGTGATATCGGCCTAATTCTAGCAATAGCATGAATAGTAGTAAACCTAAACTCATGAGAGATACAACAGCTCTTTTCTGTGTCTAAAGGCCATGACATGACCCTTCCCTTATTAGGCCTAGTACTGGCCGCTACCGGAAAGTCCGCCCAGTTTGGGCTTCACCCCTGGCTCCCCTCAGCCATAGAGGGTCCAACACCGGTCTCTGCCCTCCTGCACTCTAGCACCATGGTTGTTGCTGGTATTTTCCTTCTTATCCGCCTCAGCCCCTTAATGCAAGAAAGCCCGTTAATTCTCTCAACATGCCTTTGCCTGGGGGCCCTAACTACCGTCTTTACTGCGACATGTGCCCTTACCCAAAATGACATCAAAAAAATTGTTGCATTTTCTACATCAAGTCAACTAGGACTAATAATAGTTACCATCGGACTAGGCCAGCCCCAGCTCGCCTTTCTTCATATCTGCACCCACGCCTTCTTTAAAGCAATACTTTTCTTATGTTCAGGCTCCATCATTCATAGCCTTAATGATGAACAAGATATCCGAAAAATAGGAGGACTTCACAAGCTCCTTCCACTGACCTCTTCTTGTCTAACCATTGGCAGCCTAGCTCTAACAGGAGTCCCCTTTTTAGCAGGCTTCTTTTCCAAAGACGCCATCATTGAAGCTATAAATACATCCTACCTTAACGCCTGGGCCCTAATTTTAACGCTTCTAGCTACATCATTTACCGCAGTTTACAGTCTCCGAGTCGTATTCTTTGCCTCTATGGGCCACCCGCGTTTTAATCCAATCTCCCCAATTAATGAAAATAACCCTACAGTAATAAACCCTATTAAACGACTCGCTTGAGGAAGCATTTTGGCAGGGTTGCTAATTACGGCCAATATTGTTCCACTTAAAACCCCCGTTTTAACCATGCCTTTCACCTTAAAAATGGCCGCACTGGCTGTAACAATTATAGGACTACTCACAGCCTTAGAACTAGCATCTCTCACGTCCCAACAATTTAAAATCAAACCCTTATCTTCTACTCACCACTTCTCAAATATATTAGGATTTTTCCCGAGTGTAGCCCATCGACTAGTCCCAAAAACTGGCCTGATTCTTGGGCAACTGGTTGCCAATCAGACAATTGACCAAACCTGACTAGAGAAAACCGGGCCAAAAATAGTAGCCTCCGTTAACCTTCCAATGGCTACTTCAATTAGCAACCTACAGCAGGGTGTAATTAAGACCTACTTCTTATTATTTTTCTTCACCATAATACTAGCAATTCTCATCCTTGTCATCTAACTGCCCGTAAGGTCCCCCGACTTAGCCCTCGAGTTAACTCCAGAACTACAAAAAGCGTCAGTAATAAAACTCATCCCCCAAGCATTAAAACTCCTCCTCCTGAAGAATATATCAGAGCAACCCCCCCAAGATCCCCCCGGAATAGCATGAATTCACTAAACTCGTCAGCAGTTATCCATGACCCCTCATACCAACCCTCAGAGAAAAAGACAGAAATAGACGCGACCAGAAACACATATACTGACATAAGAAGCAAAACGGGTCAACTTCCCCACCCCTCAGGATAGGGCTCCGAAGCCAGCGCTGCTGAATACGCAAACACAACTAACATCCCGCCTAAATAGATCAAAAACAAAATCAGAGATAAAAATGAACCCCCGTGCCCTACTAAAATGCCACAGCCCATTCCTGCTACTGTGACAAGGCCCAAAGCAGCAAAGTAGGGTGAGGGGTTTGAGGCCACGGCCGCTAGGCCTAAAACCAGGCCAACTAATAATAAATAAGTTATATAAACCATAATTCTTGCCAGGATTTTAACCAGGGCCTGCGACTTGAAAAACCACCGTTGTACTCAACTACAAGAACCTAATGGCCAATCTTCGAAAAACCCATCCCCTATTAAAAATCGCAAACGATGCCCTGGTTGATCTCCCAGCCCCGTCAAACATTTCAGTTTGATGAAACTTCGGGTCTCTTCTAGGACTTTGTTTGGCCGCCCAGATTATTACGGGCCTTTTCCTTGCAATACATTATACATCAGACATTGCCACAGCATTTTCATCTGTAGCACATATTTGTCGTGACGTCAACTACGGCTGACTAATCCGAAACATGCATGCAAACGGTGCTTCCTTTTTCTTCATTTGCATCTACCTGCACATCGGACGGGGCTTGTATTATGGATCATACTTATATAAAGAGACATGAAATGTGGGTGTTGTCCTTCTCCTCCTAGTGATAATGACTGCTTTCGTAGGCTACGTCCTACCCTGAGGACAAATGTCATTCTGAGGGGCTACCGTCATTACCAACCTTTTATCAGCCATTCCCTACGTTGGAAACGCCCTAGTTCAATGAATCTGAGGCGGATTTTCAGTAGACAACGCCACCCTTACCCGGTTCTTTGCCTTCCATTTCCTCCTTCCCTTTGTGATTGCTGCTGCTACAGTTGTGCATCTTATCTTCCTCCACGAGACAGGATCGAATAATCCAACAGGTTTAAACTCAGACTCTGACAAAGTATCTTTTCACCCCTACTTTTCTTATAAAGATCTTCTAGGCTTTGCTGCCCTACTAGTAGCCCTTATCTCTTTAGCCCTCTTCTCCCCAAATCTACTGGGAGACCCCGATAACTTTACCCCTGCTAATCCTTTAGTGACGCCACCTCACATCAAACCTGAGTGATACTTCTTGTTCGCTTACGCCATTCTACGATCCATCCCAAACAAACTTGGCGGGGTTCTAGCCCTATTAGCCTCTATTCTAGTCCTCTTTCTTGTTCCCATTCTGCACACATCAAAACAGCGAAGCCTAACATTCCGACCCCTAACCCAATTCCTCTTCTGATTGCTAGTCGCCGATGTAATAATTTTAACCTGAATTGGAGGTATGCCTGTAGAACATCCTTACATTATCATTGGACAAATCGCATCCTTCATTTATTTTTCCCTTTTCCTAGTCATAGCGCCTATGGCCGGCCTACTAGAAAACAAAGTCTTAAAATGACAATGCATTAGAAGCTCAGATGAAAGAGCACCGGTCTTGTAAACCAGAGGTCGAAGGTTCAAGCCCTTCCTAGTGCTCAGAGAGAAGGGATTCTAACCCCTGCCCCTGACTCCCAAAGCCAGGATTCTTAACTAAACTACTCCCTGATTTTCATACACCAGTTTTGTAATCCAGAGCGCATCACTTTTGCCACCAACGTTAAATTAACGTTGCACAAACGTTGCATCAGCGCCCCATGGACACTAAATGACGCGAGGGCGCTAAATAAACACCCCCTACCTCTAGCACCCTTTTAACGATTTCACTTTTTTTTTTTTTTTTGTTTAACGATTACGTTTTTTTTTGCGTTCCCGGACTCTGCCAGATTTCGACCGAAGACTGCCAGAATCCGCTCAAAATCCGCTCAAATACCAATATGTATTATCCCCATAAATGGTTTAAACCATTTTTGCCTAGTACACACTGACCATGCAAGTCAATTATATTTACCCCGCGCTCCAGGCCGCAGTACATACACCTACAGTTGGTGTATTTAGCACAAGTGTGCCTCAGCTAGTTTCAAGTCACCCACATCCTTCCTTTAATTGTTACTTAATGTAGTAAGAGCCCACCATCAGTTGATTCCTTAATGTCAACGGTTCTTGAAGGTGAGGGACAAAAATCGTGGGGGTTTCACTTCTTGAATTATTCCTGGCATTTGGCTCTACATCTCAAGGCCATACATTTCTCGTCTCTCACACTTTCACTGGCCCTGACATTGGTTAATGGTGGAGTACATACTCCTCGTTACCCCCCATGCCGGGCGTTCTTTCTAATGGACAACGGGTTTTCCTTTTTTTTTCCTTTTCACTTGGCATTTCACAGTGCATACAAACCTTGATGACAAGGTTGAACATTTAGAAATCGGCCGCAAAGAATATTGGTGAATTATTCAAAGATATTAACAGATGAATTGCATAAGTGATATCAAGAGCATAAATAACCAAATGAAACTAGGAACGTTTCTATAATATGCCCCCCGGCTCCCGCGCGTCAAACCCCCCTACCCCCCTAAACTAGTAAGAAGTCTATTATTCCTGCAAACCCCCCGGAAACAGGAAACCCCCTACTAGCATTTTAGCCCGCCCAAATTTGTGTGTATTTATATTATTTGTAATATTGCAAAA

>GQ 23

GCTAGTGTAGCTTAACTAAAGCATAACACTGAAGATGTTAAGACAAACCTTAGATTGGTTTCACGAGCACAAAAGTTTGGTCCTGACTTTACTATCAACTTTAGCTAAACTTACACATGCAAGTATCCGCAATCCCGTGAGAATGCCCTACAGTTTCCTTAAAGGAAACAAGGAGCTGGTATCAGGCTCGATTACTCCCGCCCATGACACCTTGCTTAGCCACACCCCCAAGGGAACTCAGCAGTGATAGACATTAAGCAATAAGTGAAAACTTGACTTAATTAAAGCTAAGAGAACCGGTTAAACTCGTGCCAGCCACCGCGGTTATACGAGCGGTTCGAGCTGATAGATTACGGCGTAAAGCGTGGTTAATAAGAATGAAACTAAAGTCGAATGTTTTCAAAGCTGTTATACGCACTCGAAAATTAGAAGGTCAGAAACGAAAGTGACTTTAACCCTATGAACCCACGAAAACTATGAAACAAACTGGGATTAGATACCCCACTATGCATAGCTGTAAACTTTGATGAGCTATTACATTATCATCCGCCTGGGTACTACGAGCATCAGCTTAAAACCCAAAGGACTTGGCGGTGCTTTAGACCCACCTAGAGGAGCCTGTTCTAGAACCGATAACCCCCGTTAAACCTCACCCTCTCTTGTTTTTCCCGCCTATATACCGCCGTCGTCAGCTTACCCTGTGAAGGTCTAATAGTAAGCACAACCAGTTATACTCAAAACGTCAGGTCGAGGTGTAGCATATGAGAGGGGAAGAAATGGGCTACATTCCTTGTTTCAAGGAAAACGGATAACATAATGAAAGGTACGTTAGAAGGAGGATTTAGCAGTAAGCAGCAAATAGAGTGTTCTGCTGAAACTGGCCCTGAAGCGCGCACACACCGCCCGTCACTCTCCCCAACTCCGAGTTAAAAACATATATAAACCTTTGAAGGAACAAAGGGGAGGCAAGTCGTAACATGGTAAGTGTACCGGAAGGTGCACTTGGATAAATCAGAGTATAGCTAAGAAAGAAAAGCATCTCCCTTACACCGAGAAGTCATCCGTGCAAATCGGATTACCCTGACTCTAACAAGCTAGCCCAAAACCTTAACTTAAAAATCAAATATTTCTAGTAATTAATAAACCAAACACATTAAATAAATCATTTTTCCCCCTGAGTATGGGAGACAGAAAAGGATAAAGGAGCTATAGACAAAGTACCGCAAGGGAAAGCTGAAAGAGAAATGAAACAAACCAGTAAAGAAAAACAAAGCAGAGATTAACCCTTGTACCTTTTGCATCATGAATTAGCCAGTTTAATCAAGCAAAGAGCACTGTAGTTTGAAACCCCGAAACTTAGTGAGCTACTTCAAGACAGCCTATAAAATAGGGCAAACCCGTCTCTGTGGCAAAAGAGTGGGAAGATCTTCAAGTAGAGGTGACAGACCTATCGAGCTAAGTTATAGCTGGTTGCTCGTGAAATGAATAGAAGTTCAGCCTTTTGCTTTCTAAATTTCGATTTAGCATCACTTAGCCTAAATGATTAGAAAACAAAAGAGTTAGTCAAAGAGGGTACAGCCTGTTTGATAAAAGATACAACTTTACTAGGAGGATAAGAATCATAATTTTAAAGGTTTAATGCCCAGGTGGGCCTAAAAGCAGCCACCCTAATCAATAGCGTTAAAGCTTAAGCATAAAACACGCCTACAATTCTGATAAATCAGTTTTAATCCCCTAAAGTTAACGAGCTATTTCATACCTTATGAAAGAAATTATGCTAGTATGAGTAATAAGAAGTTACGAACTTCTCCCTGCACACGTGTAAATCGGAACGGACAAACCACCGAGTCTTAACGGCCCCAGTCAAAGAGGGGATGTCGGATAAAAAAAAGAACAAGAAATTCCCGATAAAACCACCGTTAACCCCACACCGGAGTGCTCCCTGGGAAAGACAAAAAGGGACAGAAGGAACTCGGCAAATATGCTCAAGCCTCGCCTGTTTACCAAAAACATCGCCTCTTGTAAAAGTTAAATAAGAGGTACCGCCTGCCCTGTGACTAGTAGTTTAACGGCCGCGGTATTTTGACCGTGCAAAGGTAGCGCAATCACTTGCCTTTTAAATGAAGGCCTGTATGAATGGCACGACGAGGGCTTAACTGTCTCCTCTCCCTAGTCAATGAAATTGATCCCCCCGTGCAGAAGCGGGGATAATAACATAAGACGAGAAGACCCTGTGGAGCTTTAGACTATAAGCAGACCATGTCAATAATAACAAACAAGTAAATTAAACAAATTGGCCCCTGCTTCTCTGTCTTTGGTTGGGGCGACCGCGGGATAATAAAAAACTCCCACGAGGATTGAGAACCCTTATCTTATAACCAAGAGCTTCTCCTCTAAGTAACAGAACATCTGACCTTAATGATCCGGCCTGGCCGATCAACGGACCGAGTTACCCCAGGGATAACAGCGCAATCCTCTTTTAGAGTCCATATCGACAAGAGGGTTTACGACCTCGATGTTGGATCAGGACATCCTAATGGTGCAGCCGCTATTAAGGGTTTGTTTGTTCAACAATTAAAGTCCTACGTGATCTGAGTTCAGACCGGAGTAATCCAGGTCAGTTTCTATCTATGACGTACTCTCTTCTAGTACGAAAGGACCGAAGAAAGAAGGCCCATGAAAAATTATGCCTTAGTCTCACCTTATGAAGAAAACTAAATAAGACAAGAGGTTACACCCCTTGGTCATAGAAAATGACATGTTAAGGTGGCAGAGCCCGGATATTGCAAAAGACCTAAGCCCTTTCCACAGAGGTTCAATTCCTCTCCTTAACTATGTTCTCAACAATATTAAGCTTCATTATTAATCCCCTGATTGTTATGATTTTTGTTTTGTTGGCAGTAGCTCTCTTGACCTTGGTAGAGCGTAAAGTGCTAAGCTACATACAACTTCGTAAAGGCCCAAATGTTGTTGGCCCTTACGGCCTTTTGCAACCCTTCGCTGATGGCTTAAAACTTTTCATGAAAGAGCCCGTCCGACCCTCCACCTCCTCGCCCGCCTTGTTCTTAATTACCCCTATTTTAGCCCTTACCTTAGCCCTAACCCTCTGAGCCCCTCTTCCTATGCCTTTTCCCGTCACCGACCTAAACTTAGGCATTTTATTTATTTTAGCACTATCGAGCCTAGCAGTATATTCTATTCTTGGCTCCGGATGAGCCTCCAACTCTAAATATGCATTAATTGGTGCTCTTCGAGCGGTCGCCCAAACCATCTCTTATGAAGTAAGCTTGGGCCTTATTCTTCTTAACACAATTGTCTTTACGGGGGGTTTTACTCTTCAAACCTTCAGCACCGCACAAGAAGCCACCTGATTACTTCTACCAGCATGACCACTAGCAGCCATGTGATATATCTCCACACTCGCGGAAACTAACCGGGCCCCTTTCGACCTAACTGAAGGAGAGTCCGAGCTAGTGTCTGGCTTCAACGTAGAGTATGCCGGCGGACCTTTTGCCCTTTTTTTTCTGGCAGAATACGGTAACATTTTACTTATAAATACCCTCTCAGCAGTACTATTTCTAGGCTCTTCAACCTACCACAGCTTTCCAGAACTAACCGCGACCTTATTAATGCTTAAGGCCACCCTCCTTTCAGTCGTATTTTTATGAGTGCGAGCATCTTACCCTCGGTTCCGATACGACCAGCTAATGCATTTAATTTGAAAAAACTTTTTGCCTCTAACCCTAGCGCTAGTTATTTGACACCTTTCTCTTCCGATCACGTTGAGCGGCCTCCCTCCTCAACTTTAACTCAGGAAATGTGCCTGAAAAAGGGTCACTTTGATAGGGTGAATAATGAGGGTTAAAGCCCCTCCATCTCCTTAGAAAGAAGGGGTTTGAACCCTACCTGAAGAGATCAAAACTCTTAGTGCTTCCACTACACCACTTCCTAGTAAAGTCAGCTAATAAAAGCTTTTGGGCCCATACCCCAAATATGTTGGTTAAAATCCTTCCTTTGCTAATGAATCCTTACGTCCTTTCAATCCTACTTATAGGTTTAGGCCTCGGTACTACAGTCACATTCGCTAGCTCACACTGACTCTTAGCATGAATAGGCCTTGAAATAAATACCCTCGCCATTTTACCATTAATAGCACAACACCACCACCCCCGAGCCGTTGAAGCCACCACCAAATATTTTTTAATTCAATCGGCAGCCGCAGCAACCATCTTATTTGCCAGCTCAACTAACGCCTGACTTTCGGGCCAGTGGGACATCATAAGTATCAATCACCCTCTTCCAACCGTCATAATTATAATTGCTCTGTCCTTAAAACTAGGCTTGGCCCCTCTTCACGCCTGACTTCCCGAAGTTATTCAAGGACTAGACTTGACCACGGGCTTAATCCTCTCCACATGACAAAAACTCGCACCCTTTGCCCTTCTCGTTCAAATCTTCCCCGACACCCCCCTTCTCATCACTTCTCTAGGACTTCTTTCGATATTAATTGGGGGGTGAGGAGGTTTAAACCACACACAACTCCGCAAAGTACTCGCATATTCTTCAATCGCCCACTTAGGCTGAATAATAGTAGTTATGCAATTCTCCACCCCCCTTACAATTCTTGCTTTATCAACATACATTGTTATAACATCATCTACTTTTCTAATCTTTAAACTCCTTAAATCCACAGATATGAACAGCCTGGCAACATCTTGAGCTAAAACCCCCTCCATTACAGCCCTAGCACCTTTAGTGCTATTGTCCTTAGGCGGACTCCCTCCCCTCTCGGGCTTTATGCCAAAATGACTAATTATTCAAGAATTAACTAAGCAAGATCTAGCCCTAGTTGCAACCTTGGCCGCCCTCTCTGCGCTACTCAGCCTTTTCTTCTACCTACGCATTTGTTACTCCCTCACATTTACCTCCTCTCCTAATAATCTCATGGGAACACCCCCCTGACGGCTAGCAACAAAGCAAGTATCACTTCCCCTAGCTATAACAACCTCCCTCTCTATTCTTCTACTCCCGGTTACCCCTGCAATCTTATCAGTGGTTCTCCCTTTGTAAAGAGGCTTAGGATAGTATTAAGACCAAGGGCCTTCAAAGCCCTAAGCGGGGGTGAAAGCCCCCCAGCCTCTGTAAGACCTACGGGACACTAACCCACATCTTCTGTATGCAAAACAGACACTTTAATTAAGCTAAAGCCTTCCTAGGTGGGTAGGCCTCGATCCTACAATCTCTTAGTTAACAGCTAAGCGCCTAAACCAGCGGGCATCCATCTACCTTTCCCCCGCCTTGCCGAAAAAAAAAGGCGGGGGAAAGCCCCGGCAGGGTATTAGCCTGCTACTTAAGATTTGCAATCTAATGTGTTAACACCTCGGGGCTGGTAAGAAGAGGACTTTAACCTCTGTCTATGGGGCTACAATCCACCGCTAAACGCTCAGCCACCTTACCTGTGGCAATCACACGTTGATTTTTCTCAACTAATCACAAAGACATCGGCACCCTGTATCTAATCTTTGGTGCCTGGGCGGGAATGGTAGGGACGGCCTTAAGTCTACTCATTCGGGCAGAACTAAGTCAACCAGGCTCCCTATTAGGAGACGACCAGATCTATAACGTAATTGTAACTGCACATGCTTTCGTAATAATTTTCTTTATAGTAATGCCAATCATAATTGGGGGGTTTGGCAACTGATTAATTCCTTTAATGATTGGAGCCCCCGACATGGCCTTCCCACGGATAAATAACATAAGCTTTTGACTCCTGCCCCCTTCTTTCCTTCTATTATTGGCCTCATCTGGTGTAGAAGCTGGTGCCGGAACAGGATGAACCGTATATCCCCCCTTGTCAGGTAATTTGGCACACGCAGGGGCCTCCGTAGATTTAACCATTTTCTCTCTTCACCTGGCCGGAATTTCTTCTATCCTAGGGGCCATTAATTTCATCACAACTATTATTAATATAAAACCTCCAGCCATTTCCCAATATCAAACCCCTTTATTTGTGTGAGCTGTACTAATCACCGCAGTATTACTTCTACTCTCTCTTCCTGTTCTAGCTGCAGGTATCACGATGCTTCTCACAGATCGGAACCTAAATACAACATTTTTCGACCCCGCAGGAGGGGGGGACCCCATTCTTTATCAACATTTATTCTGATTCTTTGGACATCCTGAAGTCTACATTCTAATTTTGCCCGGCTTCGGAATGATTTCTCACATTGTAGCATATTACTCAGGCAAAAAAGAGCCGTTTGGTTACATGGGAATAGTATGAGCTATAATAGCAATTGGCTTGCTGGGGTTTATCGTATGAGCCCATCATATGTTCACTGTGGGGATGGACGTGGACACTCGAGCTTATTTTACTTCCGCCACTATAATTATCGCAATTCCCACAGGAGTCAAAGTGTTTAGTTGACTAGCTACCTTGCATGGGGGTTCAATCAAATGAGAAACCCCTCTGTTATGAGCTCTGGGCTTTATCTTCTTATTTACTGTCGGAGGTTTAACAGGAATTGTTTTAGCCAACTCATCTCTGGACATTATACTTCATGATACATACTATGTTGTAGCCCACTTCCACTATGTCCTCTCTATAGGAGCAGTCTTTGCCATCATGGGAGCATTCGTTCACTGATTCCCCCTATTCTCAGGCTACACCCTTCACAATACGTGAACAAAAATCCACTTCGGAGTTATGTTTGTAGGTGTAAACCTCACCTTTTTCCCTCAGCACTTCTTAGGGTTGGCGGGAATACCTCGACGATATTCAGATTACCCTGACGCATACACACTGTGAAATACTATCTCATCCCTGGGGTCATTAATCTCCCTTATTGCTGTAATTATATTCCTATTTATTATCTGGGAAGCATTCGCGGCAAAACGTGAAGTCTTATCAGTAGAACTAACAGCCACAAACGTAGAATGACTGCACGGGTGTCCTCCCCCTTACCATACATTTGAAGAACCTGCATTCGTTCAAATTCAACAATCCAAATTTTAATCGAGAAAGGAAGGAGTCGAACCCCCATAAACTGGTTTCAAGCCAGCCACATAACCGCTCTGTCACTTTCTTCCCTAAGTTAATAAGATTCTAGTTAAAAGAATAACACTGCCTTGTCAAGGCAAAATTGTGGGTTAAAGCCCCACGTATCTTGCTTATGGCACATCCATCTCAACTAGGATTCCAAGATGCAGCTTCACCCGTTATAGAAGAACTTCTCCATTTTCATGACCATGCATTAATAATTGTTTTCTTAATCAGCACCCTTGTTCTTTACATTATTGTGGCTATGGTAACCACCAAGCTAACAAATAAGTTCATTCTGGACTCCCAAGAAATTGAAATCATCTGAACCTTACTACCAGCAATTATCCTAATTCTGATCGCCCTGCCCTCCCTTCGCATTCTCTACCTCATGGATGAAATCAATGACCCCCACCTCACAATTAAAGCCATAGGACATCAATGATACTGAAGCTACGAATATACGGATTATGAGGACCTAGGGTTCGACTCATATATGGTCCCTACACAAGACCTCGCCCCTGGTCAATTTCGACTACTTGAGACAGACCATCGCATGGTCATTCCTGTTGAGTCCCCCATCCGGGTTCTTGTCTCCGCCGAGGATGTTTTACACTCATGGGCCGTCCCAAGCCTCGGAGTAAAAATGGACGCCGTCCCCGGCCGCCTAAATCAAACAGCCTTCATTACTTCCCGTCCGGGTGTGTTTTATGGACAATGCTCAGAAATTTGCGGAGCTAATCATAGCTTTATGCCCATTGTAGTGGAAGCTGTTCCTCTAGAACACTTCGAGAACTGATCTTACCTAATACTTCAAGATGCCTCACCAGGAAGCTAAAAGGGAATAGCATTAGCCTTTTAAGCTAAAAATTGGTGACTCCCGCCCACCCCTGGTGACATGCCTCAGTTGAACCCCGCACCCTGATTTGCTATTATAGTATTCTCGTGACTAGTTTTCCTAGCCGTTATCCCACCTAAAGTTCTAGCTCACCATTTTCCCAATGACCCCGCCCCACAAAGCGTAAAAAAATCAAAAACAGAGACCTGATCCTGACCATGACTTTAAGCCTCTTTGATCAATTTATGAGCCCTACACTTCTAGGGGTACCTCTTATCGGACTCGCCCTAACATTGCCATGAGTCCTTTACTTCCAACCCGGTGCCCGATGACTTAATAACCGCTTGATTACCCTTCAGTCTATATTCATGAACTGATTTGTAAAACAAATCTTTCAGCCAATAAGCTTAGGCGGACACAAATGGGCCGCTCTCCTTATATCTTTAATACTATTTTTAATCACCTTAAATATGCTAGGCCTGCTACCTTACACATTTACTCCAACAACGCAGCTGTCACTTAATATAGCCTTTGCAGTACCACTTTGACTAGCAACTGTCATTATTGGAATACGAAATCAGCCAACACATGCCCTTGGTCACCTCCTCCCCGAAGGAACTCCTACCGCCCTAATCCCGGTTTTAATCGTGATTGAGACAATTAGCCTTTTTATTCGACCATTGGCCCTCGGTGTTCGACTTACCGCAAACTTGACAGCCGGGCACCTTCTAATTCAACTAATTGCAACTGCGGCTTTTGTTCTTTTCCCTATAATGCCTACAGTCGCTGCTCTTACCTCTGTCTTACTATTCTTGCTAACCCTACTAGAAGTCGCCGTGGCCATAATCCAAGCCTATGTATTCGTACTTCTTTTAAGCCTTTACCTACAAGAAAACGTCTAATGGCCCATCAAGCACATGCATATCATATAGTTGACCCAAGCCCTTGACCCCTCACAGGCGCAGTAGCCGCCCTTCTACTTACATCTGGAACAGCAATCTGAATACACTTTAACTCCACAGTTCTCATGTCCCTTGGACTTGTCCTGTTACTACTAACCATATATCAATGATGGCGAGACATTATCCGAGAGGGTACCTTTCAAGGTCATCATACACCCCCTGTTCAAAAAGGCCTTCGGTACGGGATAATTCTATTTATTACCTCAGAGGTCTTCTTTTTCCTAGGTTTCTTCTGAGCATTTTATCACTCAAGCCTAGCCCCAACCCCCGAACTTGGTGGATGTTGACCACCCATGGGTATTACAACACTGGACCCCTTTGAAGTTCCCCTTCTCAATACTGCTGTCCTTCTCGCCTCCGGTGTCACAGTCACTTGAGCTCACCATAGTATTATGGAGGGGCAGCGAAAACAAGCAATTCAGTCCTTGACACTCACAATTCTCCTGGGGTTTTACTTTACATTCCTTCAAGCAATAGAGTACTACGAGGCACCCTTCACCATTGCAGATGGCGTCTATGGCTCTACATTTTTTGTGGCAACAGGGTTTCATGGCCTCCATGTAATTATTGGATCAACATTTCTGGCAGTCTGCCTCTTACGACAAGTCCAGTTCCATTTTACATCAGAACATCACTTCGGATTTGAAGCTGCAGCATGATACTGACACTTTGTAGACGTAGTCTGACTATTCTTATATATCTCTATCTACTGATGAGGCTCATATCTTTCTAGTATTAAAAAGTACAAGTGACTTCCAATCACTTAGTCTTGGTTAAACTCCAAGGAAAGATAATGAACTTAGTACTAGTCATTATTTGCATCTCATTAGCCCTCGCCACACTGCTCGCAACTGTTTCATTTTTCCTCCCACAAATAACACCTGATTATGAGAAACTCTCACCGTATGAGTGCGGCTTTGATCCAGTGGGGTCCGCCCGTTTGCCATTCTCCATTCGCTTTTTTCTAGTCGCAATCCTATTTCTCCTCTTCGACTTAGAAATTGCCTTACTTCTTCCCCTTCCCTGAGGAGACCAACTTCCCTCCCCTCTGACAACTTTCTTTTGAGCTTCTGCTATTCTTATACTACTAACTCTAGGGTTAATCTATGAATGACTTCAAGGAGGCCTAGAGTGGGCAGAATAGGTACTTAGTTTAATAAAAACATTTGATTTCGGCTCAAAAACTTATGGTTTAAGTCCATATTTACCTGATGACCTTAACTCACTATGCATTCTCGTCAGCCTACTTTGTTAGCTTCATGGGTTTAATTTTTTACCGAAAGCATCTTCTCTCCGCCTTACTTTGCTTAGAAGCGATAATACTTATTCTTTTTATTTCACTATGCCTGTGAGGCCTAGTCTTAGCCTCAAGTGCATTTTCGGCAGGCCCAATGATCTTACTTGCTTTCTCAGCATGTGAAGCAAGTGCAGGCCTAGCACTGCTTGTAGCAATAGCTCGAACCCACGGTACTGACCGTTTAAAAAACCTTAGCCTACTCCAATGTTAATAATTCTTATTCCTACTGTTATGCTTCTACCCACAATCTGACTGAGCCCCACTAAATACCTGTGATCCTCAACACTTGGCCATAGCATAATAATTGCTCTTATAAGCCTCTCCTGACTTAGCCTCCCAGGGGAGGTTGGCTGATCTTCCCTTAACACTTTTATAGCAACAGACCCTCTCTCTACCCCCCTTCTCGTACTTACTTGCTGACTTCTGCCCTTAATAATTCTTGCGAGCCAAAACCATATAGCCCAAGAACCTACCAATCGCCAGCGAACCTATATCTCTCTCCTTACTTCCCTTCAAATCTTCTTAATCTTAGCATTTGGGGCAACCGAGATAATCATGTTCTACATTATATTTGAAGCGACCTTAATTCCCACACTTGTAATTATCACACGATGAGGAAACCAAACAGAGCGATTAAACGCAGGTATTTACTTTTTATTTTATACCTTAGCCGGCTCTTTACCACTACTAGTGGCCCTCCTTCTACTTCAGACCTCGACAGGAACTCTTTCTTTTCTAACCACTCAATTTTTTCCCCCTTTACAACTGCATACAGAAGCAAGTAAATTCTGGTGGGCAGGCTGTTTACTAGCATTCTTAGTAAAAATGCCCTTATATGGAGCACACCTTTGACTTCCAAAAGCTCACGTCGAAGCCCCCATCGCCGGGTCAATAGTCCTTGCAGCCGTTCTTTTAAAACTAGGGGGTTACGGTATGATACGAGTCATTATTATCTTAGAACCCCTCACGAAACAACTCAGCTACCCCTTTATTATTCTTGCCCTGTGGGGCGTCGTAATAACTGGCTCAATTTGCCTCCGACAAACAGACCTTAAATCACTAATCGCTTACTCCTCAGTAAGCCACATAGGCCTTGTCGCAGCAGGCATCCTAATCCAAACTCCTTGGGGGTTTACAGGAGCATTAATCCTTATAATTGCCCATGGCTTAACTTCCTCCGCCCTATTCTGTTTAGCCAACACTAACTATGAGCGAACACATAGCCGAACCATGCTTTTAGCCCGGGGTCTACAAATGGTCCTTCCTCTCTTAGCAACTTGATGGTTTCTATTTACCCTCGCCAACCTAGCACTCCCTCCGCTACCCAACCTCATAGGAGAACTTATGATTATCTCATCCTTGTATAACTGGTCAAACTGGTCTCTAATCCTGACCGGGGCGGGAGTACTAATTACCGCTAGTTACTCTCTCCATATATTCCTAACCACTCAACGTGGCCCTATTACTAACCCCGTCTTAGCAATTGAACCAACCCACACACGAGAACATCTCCTCATAATCCTTCACCTTCTTCCCCTCCTCCTTCTAATTTTAAAACCCTGCTTGATCTGGGGCTGAACAGTTTGTAGGCGTAGTTTAAATAAAGCGCTAGATTGTGATTCTAGAAATAAGAGTTAAACCCTCTTCACCCACCGAGAGGGGTCGCCGTGACAGCAAGAACTGCTAATTCTAGCCCCTTTGGTTAAAGTCCGAAGCCCACTCGAACAGGCTTCTAAAGGATAACAGCTCATCCGTTGGTCTTAGGAACCAAAAACTCTTGGTGCAACTCCAAGTAGCAGCTATGCACTTTACAACAATGATCCTCTCCTCAAGCCTAATAACAATTTTCCTTCTTCTTATCCTTCCAATCCTAGGTACACTAAACCCTAGCCCCCCGGGGGACCTATGAGCCACAAAAAACGTTAAAACAGCAGTTAAGATGGCCTTTTTTGTAAGTCTTCTACCTCTTTTTATCTTTCTTAATGAAGGAGTAGAGACTATTATAACAAACTGAAAATGAATAAATACTCTAATATTTGAAATTAATATCAGCTTTAAATTTGACCTCTACTCCGTGGTATTTACCCCTGTAGCCCTCTACGTAACATGATCAATTTTAGAGTTCGCATCTTGGTACATACACAGTGACCCCAATATAAACCGGTTCTTTAAGTATCTTTTGATCTTTCTAATCGCTATGGTTGTTCTGGTTACAGCCAACAACATGTTCCAACTATTTATTGGCTGAGAAGGTGTTGGAATTATGTCTTTCTTACTTATTGGCTGGTGGTTCGGGCGGGCTGACGCCAACACTGCGGCCCTCCAGGCCGTAGTTTATAACCGAGTTGGTGATATCGGCCTAATTCTAGCAATAGCATGAATAGTAGTAAACCTAAACTCATGAGAGATACAACAGCTCTTTTCTGTGTCTAAAGACCATGATATGACCCTTCCCTTATTAGGCCTAGTACTGGCCGCTACCGGAAAGTCCGCCCAGTTTGGACTTCACCCCTGGCTCCCCTCAGCCATAGAGGGTCCAACACCGGTCTCTGCCCTCCTGCACTCTAGCACCATGGTTGTTGCTGGTATTTTCCTTCTTATCCGCCTCAGCCCCTTAATGCAAGAAAGCCCGTTAATTCTCTCAACATGCCTTTGCCTGGGGGCCCTAACTACCGTCTTTACTGCGACATGTGCCCTTACCCAAAATGACATCAAAAAAATTGTTGCATTTTCTACATCAAGTCAATTAGGACTAATAATAGTTACCATCGGACTAGGCCAGCCCCAGCTCGCCTTCCTTCATATCTGCACCCACGCCTTCTTTAAAGCAATACTTTTCTTATGTTCAGGCTCCATCATTCATAGCCTTAATGATGAGCAAGATATCCGAAAAATAGGAGGACTTCACAAGCTCCTTCCACTGACCTCTTCTTGTCTAACCATTGGCAGCCTAGCTCTAACAGGAGTCCCCTTTTTAGCAGGCTTCTTTTCCAAAGACGCCATCATTGAAGCTATAAATACATCCTACCTTAACGCCTGAGCCCTAATTTTAACGCTTCTAGCTACATCATTTACCGCAGTTTACAGTCTCCGAGTCGTATTCTTTGCCTCTATAGGCCACCCGCGTTTTAATCCAATCTCCCCCATTAATGAAAATAACCCTACAGTGATAAACCCTATTAAACGACTCGCTTGGGGAAGCATTTTGGCAGGGTTGCTAATTACGGCCAATATTGTTCCACTTAAAACCCCCGTTTTAACCATGCCTTTCACCTTAAAAATGGCCGCACTGGCTGTTACAATTATAGGACTACTCACAGCCTTAGAACTAGCATCTCTCACGTCCCAACAATTTAAAATCAAACCCTTATCTTCTACTCACCACTTCTCAAATATATTAGGATTTTTCCCCAGTGTAGCCCATCGACTAGTCCCAAAAACTGGCCTGATTCTTGGACAACTAGTTGCCAATCAGACAATTGACCAAACCTGACTAGAGAAGACCGGGCCAAAAATAGTAGCCTCCGTTAACCTTCCAATGGCTACTTCAATTAGCAACCTACAGCAAGGTGTAATTAAGACCTACTTCTTATTATTTTTCTTCACCATAATACTGGCAATTCTCATCCTTGTCATCTAACTGCCCGTAAGGTCCCCCGACTTAGCCCTCGAGTTAACTCCAGAACTACAAAAAGCGTCAGTAATAAAACTCATCCCCCAAGCATTAAAACTCCTCCTCCTGAAGAATATATCAGAGCAACCCCACCAAGATCCCCCCGGAATAGCATGAATTCACTAAACTCGTCAGCAGTTATCCATGACCCCTCATACCAACCCTCAGAGAAAAAGACAGAGATAGACGCGACCAGAAACACATATACTGACATAAGAAGCAAAACCGGTCAACTTCCCCACCCCTCAGGATAAGGCTCCGAAGCCAGCGCTGCTGAATACGCAAACACAACTAACATCCCACCTAAATAGATCAAAAACAAAATCAAAGATAAAAATGAACCCCCGTGCCCTACTAAAATGCCACAGCCCATTCCTGCTACTGTGACAAGGCCCAAGGCAGCAAAGTAGGGTGAGGGGTTCGAGGCCACGGCCGCTAGACCTAAAACCAGACCAACTAGTAATAAATAAGTTATATAAACCATAATTCTTGCCAGGATTTTAACCAGGGCCTGCGACTTGAAAAACCACCGTTGTACTCAACTACAAGAACCTAATGGCCAATCTTCGAAAAACCCATCCCCTATTAAAAATCGCAAACGATGCCCTGGTTGATCTCCCAGCCCCATCGAACATTTCAGTTTGATGAAACTTCGGGTCTCTTCTAGGACTTTGTTTGGCCGCCCAGATTGTTACGGGCCTTTTCCTTGCAATACATTATACATCAGACATTGCCACAGCATTTTCATCTGTAGCACATATTTGTCGTGACGTCAACTACGGCTGACTAATCCGAAACATGCATGCAAACGGTGCTTCCTTTTTCTTCATTTGCATCTACCTGCACATCGGACGGGGCTTGTATTATGGATCATACTTATATAAAGAGACATGAAATGTGGGTGTTATCCTTCTCCTCCTAGTGATAATGACTGCTTTCGTAGGCTACGTCCTACCCTGAGGACAAATGTCATTCTGAGGGGCTACCGTCATTACCAACCTTTTATCAGCCATTCCCTACGTTGGAAACGCCCTAGTTCAATGAATCTGAGGCGGATTTTCAGTAGACAACGCCACCCTTACCCGGTTCTTTGCCTTCCATTTCCTCCTTCCCTTTGTAATTGCTGCTGCTACAGTTGTTCATCTTATCTTCCTGCACGAGACAGGATCGAATAATCCAACGGGTTTAAACTCAGACTCTGACAAAGTATCTTTTCACCCCTACTTTTCTTATAAAGATCTTCTAGGATTTGCTGCCCTACTAGTAGCCCTTATCTCTTTAGCCCTCTTCTCCCCAAATCTACTCGGAGACCCCGATAACTTTACCCCTGCTAATCCTTTAGTGACTCCACCTCACATCAAGCCTGAGTGATACTTCCTGTTCGCTTACGCCATTCTACGATCCATCCCAAACAAACTTGGCGGAGTTCTAGCCCTATTAGCCTCTATTCTAGTCCTCTTTCTTGTTCCCATTCTGCACACATCGAAACAACGAAGCCTAACATTCCGACCCCTGACCCAATTCCTCTTCTGATTGCTAGTCGCCGATGTAATAATTTTAACCTGAATTGGAGGCATGCCTGTAGAACACCCTTACATTATCATTGGACAAATCGCATCCTTCATTTATTTTTCCCTTTTCCTAGTCATAGCGCCTATGGCCGGCCTACTAGAAAACAAAGTCTTAAAATGACAATGCATTAGAAGCTCAGATGAAAGAGCACCGGTCTTGTAAACCAGAGGTCGAAGGTTCAAGCCCTTCCTAGTGCTCAGAGAGAAGGGATTCTAACCCCTGCCCCTGACTCCCAAAGCCAGGATTCTTAGCTAAACTACTCCCTGATTTTCATACACCAGTTTTGCAATCCAGAGCGCATCACTTTTGCCACCAACGTTAAATTAACGTTGCACAAACGTTGCATCAGCGCCCCATGGACACTAAATGACGCGAGGGCGCTAAATAAACACCCCCTACCTCTAGCACCCTTTTAACGATTTCACTTTTTTTTTTTTTTTTGTTTAACGATTACGTTTTTTTTTGCGTTCCCGGACTCTGCCAGATTTCGACCGAAGACTGCCAGAATCCGCTCAAAATCCGCTCAAATACCAATATGTATTATCCCCATAAATGGTTTAAACCATTTTTGCCTAGTACACGCTGACCATGCAAGTCAATTATATTTACCCCGCGCTCCAGGCCGCAGTACATACACCTACAGTTGGTGTATTTAGCACAAGTGTGCCTCAGCTAGTTTCAAGTCACCCACATCCTTCCTTTAATTGTTACTTAATGTAGTAAGAGCCCACCATCAGTTGATTCCTTAATGTCAACGGTTCTTGAAGGTGAGGGACAAAAATCGTGGGGGTTTCACTTCTTGAATTATTCCTGGCATTTGGCTCTACATCTCAAGGCCATACATTTCTCGTCTCTCACACTTTCACTGGCCCTGACATTGGTTAATGGTGGAGTACATACTCCTCGTTACCCCCCATGCCGGGCGTTCTTTCTAATGGACAACGGGTTTTCCTTTTTTTTTCCTTTTCACTTGGCATTTCACAGTGCATACAGACCTTGATGACAAGGTTGAACATTTAGAAATCGGCCGCAAAGAATATTGGTGAGTTATTTAAAGATATTAACAGATGAATTGCATAAGTGATATCAAGAGCATAAATAACCAAATGAAACTAGGAACGTTTCTATAATATGCCCCCCGGCTTCCGCGCGTCAAACCCCCCTACCCCCCTAAACTAGTAAGAAGTCTATTATTCCTGCAAACCCCCCGGAAACAGGAAACCCCCTACTAGCATTTTAGCCCGCCCAAATTTGTGTGTATTTATATTATTTGTAATATTGCAAAA

>GQ 3

GCTAGTGTAGCTTAACTAAAGCATAACACTGAAGATGTTAAGACAAACCTTAGATTGGTTTCACGAGCACAAAAGTTTGGTCCTGACTTTACTATCAACTTTAGCTAAACTTACACATGCAAGTATCCGCAATCCCGTGAGAATGCCCTACAGTTTCCTTAAAGGAAACAAGGAGCTGGTATCAGGCTCAATTACTCCCGCCCATGACACCTTGCTTAGCCACACCCCCAAGGGAACTCAGCAGTGATAGACATTAAGCAATAAGTGAAAACTTGACTTAATTAAAGCTAAGAGAACCGGTTAAACTCGTGCCAGCCACCGCGGTTATACGAGCGGTTCGAGCTGATAGATTACGGCGTAAAGCGTGGTTAATAAGAATGAAACTAAAGTCGAATGTTTTCAAAGCTGTTATACGCACTCGAAAATTAGAAGGTCAGAAACGAAAGTGACTTTAACCCTATGAACCCACGAAAACTATGAAACAAACTGGGATTAGATACCCCACTATGCATAGCTGTAAACTTTGATGAGCTGTTACATTATCATCCGCCTGGGTACTACGAGCATCAGCTTAAAACCCAAAGGACTTGGCGGTGCTTTAGACCCACCTAGAGGAGCCTGTTCTAGAACCGATAACCCCCGTTAAACCTCACCCTCTCTTGTTTTTCCCGCCTATATACCGCCGTCGTCAGCTTACCCTGTGAAGGTCTAATAGTAAGCACAACCAGTTATACTCAAAACGTCAGGTCGAGGTGTAGCATATGAGAGGGGAAGAAATGGGCTACATTCCTTGTTTCAAGGAAAACGGATAACATAATGAAAGGTACGTTAGAAGGAGGATTTAGCAGTAAGCAGCAAATAGAGTGTTCTGCTGAAACTGGCCCTGAAGCGCGCACACACCGCCCGTCACTCTCCCCAACTCCGAGTTAAAAACATATATAAACCTTTGAAGGAACAAAGGGGAGGCAAGTCGTAACATGGTAAGTGTACCGGAAGGTGCACTTGGATAAATCAGAGTATAGCTAAGAAAGAAAAGCATCTCCCTTACACCGAGAAGTCATCCGTGCAAATCGGATTACCCTGACTCTAACAAGCTAGCCCAAAACCTTAACTTAAAAATCAAATATTTCTAGTAATTAATAAACCAAACACATTAAATAAATCATTTTTCCCCCTGAGTATGGGAGACAGAAAAGGACAAAGGAGCTATAGACAAAGTACCGCAAGGGAAAGCTGAAAGAGAAATGAAACAAACCAGTAAAGAAAAACAAAGCAGAGATTAACCCTTGTACCTTTTGCATCATGAATTAGCCAGTTTAATCAAGCAAAAAGCACTGTAGTTTGAAACCCCGAAACTTAGTGAGCTACTTCAAGACAGCCTATGAAATAGGGCAAACCCGTCTCTGTGGCAAAAGAGTGGGAAGATCTTCAAGTAGAGGTGACAGACCTATCGAACTAAGTTATAGCTGGTTGCTCGTGAAATGAATAGAAGTTCAGCCTTTTGCTTTCTAAATTTCGATTTAGCATCACTTAGCCTAAATGACTAGAAAACAAAAGAGTTAGTCAAAGAGGGTACAGCCTGTTTGATAAAAGATACAACTTTGCTAGGAGGATAAGAATCATAATTTTAAAGGTTTAATGCCCAGGTGGGCCTAAAAGCAGCCACCCTAATCAATAGCGTTAAAGCTTAAGCATAAAACACACCTACAATTCTGATAAATCAGTTTTAATCCCCTAAAGTTAACGAGCTATTTCATACCTTATGAAAGAAATTATGCTAGTATGAGTAATAAGAAGTTACGAACTTCTCCCTGCACACGTGTAAATCGGAACGGACAAACCACCGAATCTTAACGGCCCCAGTCAAAGAGGGGATGTCGGATAAAAAAAAGAACAAGAAATTCCCGATAAAACCACCGTTAACCCCACACCGGAGTGCTCCCTGGGAAAGACAAAAAGGGACAGAAGGAACTCGGCAAATATGCTCAAGCCTCGCCTGTTTACCAAAAACATCGCCTCTTGTAAAAGTTAAATAAGAGGTACCGCCTGCCCTGTGACTAGTAGTTTAACGGCCGCGGTATTTTGACCGTGCAAAGGTAGCGCAATCACTTGCCTTTTAAATGAAGGCCTGTATGAATGGCACGACGAGGGCTTAACTGTCTCCTCTCCCTAGTCAATGAAATTGATCCCCCCGTGCAGAAGCGGGGATAATAACATAAGACGAGAAGACCCTGTGGAGCTTTAGACTATGAGCAGACCATGTCAAGAATAACAAACAAGTAAATTAAACAAATTGGCCCCTGCTTCCCTGTCTTTGGTTGGGGCGACCGCGGGATAATAAAAAACTCCCACGAGGATTGAGAACCCTTATCTTATAACCAAGAGCTTCTCCTCTAAGTAACAGAACATCTGACCTTAATGATCCGGCCAGGCCGATCAACGGACCGAGTTACCCCAGGGATAACAGCGCAATCCTCTTTTAGAGTCCATATCGACAAGAGGGTTTACGACCTCGATGTTGGATCAGGACATCCTAATGGTGCAGCCGCTATTAAGGGTTTGTTTGTTCAACAATTAAAGTCCTACGTGATCTGAGTTCAGACCGGAGTAATCCAGGTCAGTTTCTATCTATGACGTACTCTCTTCTAGTACGAAAGGACCGAAGAAAGAAGGCCCATGAAAAATTATGCCTTAGTCTCACCTTATGAAGAAAACTAAATAAGACAAGAGGTTACACCCTTTGGTCATAGAAAATGACATGTTAAGGTGGCAGAGCCCGGATATTGCAAAAGACCTAAGCCCTTTCCACAGAGGTTCAATTCCTCTCCTTAACTATGTTCTCAACAATATTAAGCTTCATTATTAATCCCCTGATTGTTATGGTTTTTGTTTTGTTGGCAGTAGCCCTCTTGACCTTGGTAGAGCGTAAAGTGCTAAGCTACATGCAACTTCGTAAAGGCCCAAATGTTGTTGGCCCTTACGGCCTTTTGCAACCCTTCGCTGATGGCTTAAAACTTTTCATGAAAGAGCCCGTCCGACCCTCCACCTCCTCGCCCGCCTTGTTCTTAATTACCCCTATTATAGCCCTTACCTTAGCCCTAACCCTCTGAGCCCCCCTTCCTATGCCTTTTCCCGTCACCGACCTAAACTTAGGCATTTTATTTATTTTAGCACTATCGAGCCTGGCAGTATATTCTATTCTTGGCTCCGGATGAGCCTCCAACTCTAAATATGCATTAATTGGTGCTCTTCGAGCGGTCGCCCAAACCATCTCTTATGAAGTAAGCTTGGGCCTTATTCTTCTTAACACAATTGTCTTTACGGGGGGTTTTACTCTTCAAACCTTCAGCACCGCACAAGAAGCCACCTGATTACTTCTACCAGCATGACCCCTAGCAGCCATGTGATATATCTCCACACTCGCGGAAACTAACCGAGCCCCTTTCGACCTAACTGAAGGAGAGTCCGAACTAGTGTCTGGCTTCAACGTAGAGTATGCCGGCGGACCTTTTGCCCTTTTTTTTCTGGCAGAATACGGTAACATTTTACTTATAAATACCCTCTCAGCAGTACTATTTCTAGGCTCTTCAACCTACCACAGCTTTCCAGAACTAACCGCGACCTTATTAATGCTTAAAGCCACCCTCCTTTCAGTCGTATTTTTATGAGTGCGAGCATCTTACCCTCGGTTCCGATACGACCAACTAATGCATTTAATTTGAAAAAACTTTTTACCTCTGACCCTAGCGCTAGTTATTTGACACCTTTCTCTTCCGATCACGTTGAGCGGCCTCCCTCCTCAACTTTAACTCAGGAAATGTGCCTGAAAAAGGGTCACTTTGATAGGGTGAATAATGAGGGTTAAAGCCCCTCCATCTCCTTAGAAAGAAGGGGTTTGAACCCTACCTGAAGAGATCAAAACTCTTAGTGCTTCCACTACACCACTTCCTAGTAAAGTCAGCTAATAAAAGCTTTTGGGCCCATACCCCAAATATGTTGGTTAAAATCCTTCCTTTGCTAATGAATCCTTACGTCCTTTCAATTCTACTTATGGGTTTAGGCCTCGGCACTACAGTCACATTCGCTAGCTCACACTGACTCTTAGCATGAATAGGCCTTGAAATAAATACCCTCGCCATTTTGCCATTAATAGCACAACATCACCACCCCCGAGCCGTTGAAGCCACCACCAAGTATTTTTTGATTCAATCGGCAGCCGCAGCAACCATCTTATTTGCCAGCTCAACTAACGCCTGACTTTCGGGCCAGTGGGACATCATAAGTATCAATCACCCCCTTCCAACCGTCATAATTACAATCGCTCTGTCCTTAAAACTAGGCTTGGCCCCTCTTCACGCCTGACTTCCCGAAGTTATTCAAGGACTAGACTTGACCACGGGCTTAATCCTCTCCACATGACAAAAACTCGCACCCTTTGCCCTTCTCGTTCAAATCTTCCCCGACACCCCCCTTCTCATCACTTCTTTAGGGCTTCTTTCAATATTAATTGGGGGGTGAGGAGGTTTAAACCACACACAACTCCGCAAAGTGCTCGCATATTCTTCGATCGCCCACTTAGGCTGAATAATAGTAATTATGCAATTCTCCACCCCCCTTACAATTCTTGCTTTATCAACATACATTGTTATAACATCATCTACTTTTCTAATCTTTAAACTCCTTAAATCCACAGATATGAACAGCCTGGCAACATCTTGAGCTAAAACCCCCTCCATTACAGCCCTAGCACCTTTAGTGCTATTATCCTTAGGCGGACTTCCTCCCCTCTCGGGCTTTATGCCAAAATGATTAATTATTCAAGAATTAACTAAGCAAGATCTAGCCCTAGTTGCAACCTTGGCCGCCCTCTCTGCGCTACTCAGCCTTTTCTTCTACCTACGCATTTGTTACTCCCTCACATTTACCTCCTCTCCTAATAATCTCATGGGAACGCCCCCCTGACGACTAGTAACAAAGCAAGTATCACTTCCCCTAGCTATAACAACCTCCCTCTCTATTCTTCTACTCCCGGTCACCCCTGCAATTTTATCAATGGTTCTCCCTTTGTAAAGAGGCTTAGGATAGTATTAAGACCAAGGGCCTTCAAAGCCCTAAGCGGGAGTGAAAGCCCCCCAGCCTCTGTAAGACCTACGGGACACTAACCCACATCTTCTGTATGCAAAACAGACACTTTAATTAAGCTAAAGCCTTCCTAGGTGGGTAGGCCTCGATCCTACAATCTCTTAGTTAACAGCTAAGCGCCTAAACCAGCGGGCATCCATCTACCTTTCCCCCGCCTTGCCGAAAAAAAAAGGCGGGGGAAAGCCCCGGCAGGGTGTTAGCCTGCCACTTAAGATTTGCAATCTAATGTGTTAACACCTCGGGGCTGGTAAGAAGAGGACTTTAACCTCTGTCCATGGGGCTACAATCCACCGCTAAACGCTCAGCCACCTTACCTGTGGCAATCACACGTTGATTTTTCTCAACTAATCACAAAGACATCGGCACCCTGTATCTAATCTTTGGTGCCTGGGCGGGAATGGTAGGGACGGCCTTAAGTCTACTCATTCGAGCAGAACTAAGTCAACCAGGCTCCCTATTAGGAGACGACCAGATCTATAACGTAATTGTAACTGCACATGCTTTCGTAATAATTTTCTTTATAGTAATGCCAATCATAATTGGGGGGTTTGGCAACTGATTAATCCCTTTAATGATTGGAGCCCCCGACATGGCCTTCCCACGGATAAATAATATAAGCTTTTGACTCCTGCCCCCTTCTTTCCTTCTATTATTGGCCTCATCTGGTGTAGAAGCTGGTGCCGGAACAGGATGAACCGTATATCCCCCCTTGTCAGGTAATTTGGCACACGCAGGGGCCTCCGTAGATTTAACCATTTTCTCTCTTCACCTAGCCGGAATTTCTTCTATCCTAGGGGCCATTAATTTCATCACAACTATTATTAATATAAAACCTCCAGCCATTTCCCAATATCAAACCCCTTTATTTGTGTGGGCTGTACTAATTACCGCAGTATTACTTCTACTCTCTCTTCCTGTTCTAGCTGCAGGTATCACCATGCTTCTCACAGATCGGAACCTAAATACAACATTTTTCGACCCCGCAGGAGGGGGGGACCCCATTCTTTATCAACATTTATTCTGATTCTTTGGGCATCCTGAAGTCTACATTCTAATTTTGCCCGGCTTCGGAATGATTTCTCACATTGTAGCATATTACTCAGGCAAAAAAGAGCCGTTTGGTTACATGGGAATAGTATGAGCTATAATAGCAATTGGCTTGCTGGGCTTTATCGTATGAGCCCATCATATGTTCACTGTAGGGATGGACGTGGACACTCGAGCTTATTTTACTTCCGCCACTATAATTATCGCAATTCCCACAGGAGTCAAAGTGTTTAGTTGACTAGCTACCTTGCATGGGGGCTCAATCAAATGAGAAACCCCTCTGTTATGAGCTCTGGGCTTTATCTTCTTATTTACTGTCGGAGGTTTAACAGGAATTGTTTTAGCCAACTCATCTCTGGACATTATACTTCATGATACATACTATGTTGTAGCCCACTTCCACTATGTCCTCTCTATAGGAGCAGTCTTTGCCATCATGGGGGCATTCGTTCACTGATTCCCCCTATTTTCAGGCTACACCCTTCACAATACGTGAACAAAAATCCACTTCGGAGTTATGTTTGTAGGTGTAAACCTCACCTTTTTCCCTCAGCACTTCTTAGGATTGGCGGGAATACCTCGACGATACTCAGATTACCCTGACGCATACACACTGTGAAATACTATCTCATCCCTGGGGTCATTAATCTCCCTTATTGCTGTAATTATGTTCCTATTTATTATCTGGGAGGCATTCGCGGCAAAACGTGAAGTCTTATCAGTTGAACTAACAGCCACAAACGTAGAATGACTGCACGGGTGTCCTCCCCCTTACCATACATTTGAAGAACCTGCATTCGTTCAAATTCAACAATCCAAATTTTAATCGAGAAAGGAAGGAGTCGAACCCCCATAAACTGGTTTCAAGCCAGCCACATAACCGCTCTGTCACTTTCTTCCCTAAGTTAATAAGATTCTAGTTAAAGGAATAACACTGCCTTGTCAAGGCAAAATTGTGGGTTAAAGCCCCACGTATCTTGCTTATGGCACATCCATCTCAACTAGGATTCCAAGATGCAGCTTCACCCGTTATAGAAGAACTTCTCCATTTTCATGACCATGCATTAATAATTGTTTTCTTAATCAGCACCCTTGTTCTTTACATTATTGTGGCTATGGTAACCACCAAGCTAACAAATAAGTTCATTCTGGACTCCCAAGAAATTGAAATCATCTGAACCTTGCTACCAGCAATTATCCTAATTCTGATCGCCCTACCCTCCCTTCGCATTCTCTACCTCATGGATGAAATCAATGACCCCCACCTCACAATTAAAGCCATAGGACATCAATGATACTGAAGCTACGAATATACGGATTATGAAGACCTAGGGTTCGACTCGTATATGGTCCCTACACAAGACCTCGCCCCTGGTCAATTTCGACTACTTGAAACAGACCATCGCATGGTCATTCCTGTTGAGTCCCCCATCCGGGTTCTTGTCTCCGCCGAGGATGTTTTACACTCATGGGCCGTCCCAAGCCTCGGAGTAAAAATGGACGCCGTCCCCGGCCGCCTAAATCAAACAGCCTTCATTACCTCCCGTCCGGGTGTGTTTTATGGACAATGCTCAGAAATTTGCGGAGCTAATCATAGCTTTATACCCATTGTAGTGGAAGCTGTTCCTCTAGAACACTTCGAGAACTGATCTTACCTAATACTTCAAGATGCCTCACCAGGAAGCTAAAAGGGAATAGCATTAGCCTTTTAAGCTAAAAATTGGTGACTCCCGCCCACCCCTGGTGACATGCCTCAGTTGAACCCCGCACCCTGATTTGCTATTATAGTATTCTCATGACTAGTTTTCCTAGCCGTTATCCCACCTAAAGTTTTAGCTCACCATTTTCCCAATGACCCCGCCCCACAGAGCGTAAAAAAATCAAAAACAGAGACCTGATCCTGACCATGACTTTAAGCCTCTTTGATCAATTTATGAGCCCTACACTTCTAGGGGTGCCTCTTATTGGACTCGCCCTAACATTGCCATGAGTCCTTTACTTCCAACCCGGTGCCCGATGACTTAATAACCGCTTCATCACCCTTCAATCTATATTCATGAACTGATTTGTAAAACAAATCTTTCAGCCAATAAGCTTAGGCGGACACAAATGGGCCGCTCTCCTCATATCTTTAATACTATTTTTAATTACCTTAAATATGCTAGGCCTACTGCCTTATACATTTACTCCAACAACGCAGCTGTCACTTAATATAGCCTTTGCAGTACCACTTTGACTAGCAACTGTCATTATTGGAATACGAAATCAGCCAACACATACCCTTGGTCACCTCCTCCCCGAAGGAACTCCTACCGCCCTAATCCCGGTTTTAATCGTGATTGAGACAATTAGCCTTTTTATTCGACCATTGGCCCTCGGTGTTCGACTTACCGCAAACTTGACAGCCGGGCACCTTCTAATTCAACTAATTGCAACTGCGGCTTTTGTTCTTTTCCCTATAATGCCCACAGTCGCCGCTCTTACCTCTGTCTTACTATTCTTGCTAACCCTACTAGAAGTCGCCGTGGCCATAATCCAAGCCTATGTATTCGTACTTCTTTTAAGCCTTTACCTACAAGAAAACGTCTAATGGCCCATCAAGCACATGCATATCATATAGTTGACCCAAGCCCTTGACCCCTCACAGGCGCAGTAGCCGCCCTTCTACTTACATCTGGAACAGCAATCTGAATACACTTTAACTCCACAGTTCTCATGTCCCTTGGACTTGTTCTGCTACTACTAACCATATATCAATGATGGCGAGACATTATCCGAGAGGGCACCTTTCAAGGTCATCATACACCCCCTGTTCAAAAAGGCCTTCGGTACGGGATAATTCTATTTATTACCTCAGAGGTCTTCTTTTTCCTAGGTTTCTTCTGAGCATTTTATCACTCAAGCCTAGCCCCAACCCCCGAACTTGGTGGATGTTGACCACCCATGGGTATTACAACACTGGACCCCTTTGAAGTTCCCCTTCTCAATACTGCTGTCCTTCTCGCCTCCGGTGTCACGGTCACTTGAGCTCACCATAGTATTATGGAGGGGCAGCGAAAACAAGCAATTCAGTCCTTGACACTCACAATTCTCCTGGGGTTTTACTTTACATTCCTTCAAGCAATAGAGTACTACGAGGCACCCTTCACCATTGCAGATGGCGTCTATGGCTCTACATTTTTTGTGGCAACAGGGTTTCATGGCCTCCATGTAATTATTGGATCAACATTTCTGGCAGTCTGCCTCTTACGACAAGTCCAGTTCCATTTTACATCAGAACATCACTTCGGATTCGAAGCTGCAGCATGATACTGACACTTTGTAGACGTAGTCTGACTATTCTTATATATCTCTATCTACTGATGAGGCTCATATCTTTCTAGTATTAAAAAGTACAAGTGACTTCCAATCACTCAGTCTTGGTTAGACTCCAAGGAAAGATAATGAACTTAGTACTAGTCATTATTTGCATCTCATTAGCCCTCGCCGCACTGCTCGCAACTGTTTCATTTTTCCTCCCACAAATAACCCCTGATTATGAGAAACTCTCACCGTATGAGTGCGGCTTTGATCCAGTGGGGTCCGCCCGTTTGCCATTCTCCATTCGCTTTTTTCTAGTCGCAATCCTATTTCTCCTCTTCGACTTAGAAATTGCCTTACTTCTTCCCCTTCCCTGAGGGGACCAACTTCCCTCCCCTCTAACAACTTTCTTTTGAGCTTCTGCTATTCTTATACTACTAACTCTAGGGTTAATCTATGAATGACTTCAAGGGGGCCTAGAGTGGGCAGAATAGGTACTTAGTTTAATAAAAACATTTGATTTCGGCTCAAAAACTTATGGTTTAAGTCCATATTTACCTGATGACCTTAACTCACTATGCATTCTCGTCAGCCTACTTTGTTAGCTTCATGGGTTTAATTTTTTACCGAAAGCATCTTCTCTCCGCCTTACTTTGCTTAGAAGCGATAATACTTATTCTTTTTATTTCACTATGCCTGTGAGGTCTAGTCTTAGCCTCAAGTGTATTTTCGGCAGGCCCAATGATCTTACTTGCTTTCTCAGCATGTGAAGCAAGTGCAGGCCTAGCACTGCTTGTAGCAATAGCTCGAACCCACGGTACTGACCGTTTAAAAAACCTTAGCCTACTCCAATGTTAATAATTCTTATTCCTACTGTTATGCTTCTACCCACAATCTGACTGAGCCCCACTAAATACCTGTGATCCTCAACACTCGGCCATAGCATAATGATTGCTCTTATAAGCCTCTCCTGACTTAGCCTCCCGGGGGAGGTTGGCTGATCTTCCCTTAACACTTTTATAGCAACAGACCCTCTCTCTACCCCCCTTCTCGTACTTACTTGCTGACTTCTGCCCTTAATAATTCTTGCGAGCCAAAACCATATAGCCCAAGAACCTACCAATCGCCAGCGAACCTACATCTCTCTCCTAACTTCCCTTCAAATCTTCTTAATCTTAGCATTTGGGGCAACCGAGATAATCATGTTCTACATTATATTTGAAGCGACCTTAATTCCCACACTCGTAATTATCACACGATGAGGGAACCAAACAGAGCGATTAAACGCAGGTATTTACTTTTTATTTTATACCTTAGCCGGCTCTTTACCACTACTAGTGGCCCTCCTTCTACTTCAGACCTCGACAGGAACTCTTTCTTTTCTAACCACTCAATTTTTTCCCCCTTTACAACTGCATACAGAAGCAAGTAAATTCTGGTGGGCAGGCTGTTTACTAGCATTCTTAGTAAAAATGCCCCTATATGGAGCACACCTTTGACTTCCAAAAGCTCACGTCGAAGCCCCCATCGCCGGGTCAATAGTCCTTGCAGCCGTTCTTTTAAAACTAGGGGGTTACGGTATGATACGAGTCATTATTATCTTAGAGCCCCTCACGAAACAACTCAGCTACCCCTTTATTGTTCTTGCCCTGTGGGGCGTCGTAATAACTGGCTCAATTTGCCTCCGACAAACAGACCTTAAATCACTAATCGCTTACTCCTCAGTAAGCCACATAGGCCTTGTCGCAGCAGGCATCCTAATCCAAACTCCTTGGGGGTTTACAGGAGCATTAATCCTTATAATTGCCCATGGCTTAACTTCCTCCGCCCTATTCTGTTTAGCCAACACTAACTATGAGCGAACACATAGCCGAACCATGCTTTTAGCCCGGGGTCTACAAATGGTCCTTCCTCTCTTAGCAACTTGATGGTTTCTATTTACCCTCGCCAACCTAGCACTCCCTCCGCTACCCAACCTCATAGGGGAACTTATGATTATCTCATCCTTGTATAACTGGTCAAACTGGTCTCTAATCCTGACCGGGGCGGGAGTACTAATTACCGCTAGCTACTCTCTCCATATATTCCTAACCACTCAACGTGGCCCTATTACTAACCCCGTCTTGGCAATTGAACCAACCCACACACGAGAACATCTCCTCATAATCCTTCACCTTCTTCCCCTCCTCCTTCTAATTTTAAAACCCTGCTTGATCTGGGGCTGAACAGTTTGTAGGCGTAGTTTAAATAAAGCGCTAGATTGTGATTCTAGAAATAAGAGTTAAATCCTCTTCACCCACCGAGAGGGGTCGCCGTGACAGCAAGAACTGCTAATTCTAGCCCCTTTGGTTAAAGTCCGAAGCCCACTCGAACAGGCTTCTAAAGGATAACAGCTCATCCGTTGGTCTTAGGAACCAAAAACTCTTGGTGCAACTCCAAGTAGCAGCTATGCACTTTACAACAATGATTCTCTCCTCAAGCCTAATAACAATTTTCCTTCTTCTTATCCTTCCAGTCCTAGGTACACTAAACCCTGACCCCACAGGGGACCTGTGGGCCACAAAAAACGTTAAAACAGCAGTTAAGATGGCCTTTTTTGTAAGCCTTCTACCTCTTTTTATCTTTCTTAATGAAGGAGTAGAGACTATTATAACAAACTGAAAATGGATAAATACTCTAATATTTGAGATTAATATCAGCTTTAAATTTGACCTCTACTCCGTGGTATTTACCCCTGTAGCCCTCTACGTAACATGATCAATTTTAGAGTTCGCATCTTGGTACATACACAGTGACCCCAATATAAACCGATTCTTTAAGTATCTTCTAATCTTTCTAATCGCTATGGTTGTTCTGGTTACAGCCAACAACATGTTCCAACTATTTATTGGCTGAGAAGGTGTTGGAATTATGTCTTTCTTACTTATTGGCTGGTGGTTCGGGCGGGCTGACGCCAACACTGCGGCCCTCCAAGCCGTAGTTTATAACCGAGTCGGTGATATCGGCCTAATTCTAGCAATAGCATGAATAGTAGTAAACCTAAACTCATGAGAGATACAACAGCTCTTTTCTGTGTCTAAAGGCCATGATATGACCCTTCCCTTACTAGGCCTAGTACTGGCCGCTACCGGAAAGTCCGCCCAGTTTGGACTTCACCCCTGGCTCCCCTCAGCCATAGAGGGTCCAACACCGGTCTCTGCCCTCCTGCACTCTAGCACCATGGTTGTTGCTGGTATTTTCCTTCTTATCCGCCTCAGCCCCTTAATGCAAGAAAGCCCGTTAATTCTCTCAACATGCCTTTGCCTGGGGGCCCTAACTACCGTCTTTACTGCGACATGTGCCCTTACCCAAAATGACATCAAAAAAATTGTTGCATTTTCTACATCAAGTCAATTAGGACTAATAATAGTTACCATCGGACTAGGCCAGCCCCAGCTCGCCTTTCTTCATATCTGCACCCACGCCTTCTTTAAAGCAATACTTTTCTTATGTTCAGGCTCCATCATTCATAGCCTTAATGATGAGCAAGATATCCGAAAAATAGGAGGACTTCACAAGCTCCTTCCACTGACCTCTTCTTGTCTAACCATTGGCAGCCTAGCTCTAACAGGAGTCCCCTTTTTAGCAGGCTTCTTTTCCAAAGACGCCATCATTGAAGCTATAAGTACATCCTACCTTAACGCCTGAGCCCTAATTTTAACGCTTCTAGCCACATCATTTACCGCAGTTTACAGTCTCCGAGTCGTATTCTTTGCCTCTATGGGCCACCCGCGTTTTAATCCAATCTCCCCAATTAATGAAAATAACCCTACAGTAATAAACCCTATCAAACGACTCGCTTGGGGAAGCATTTTGGCAGGGTTGCTAATTACGGCCAATATTGTTCCACTTAAAACCCCCGTTTTAACCATGCCTTTCACCTTAAAAATGGCCGCACTGGCTGTAACAATTATAGGACTACTCACAGCCTTAGAACTAGCATCTCTCACGTCCCAACAATTTAAAATCAAACCCTTATCTTCTACTCACCACTTCTCAAATATATTAGGATTTTTCCCGAGTGTAGCCCATCGACTAGTCCCAAAAACTGGCCTGATTCTTGGGCAACTGGTTGCCAATCAGACAATTGACCAAACCTGACTAGAGAAAACCGGGCCAAAAATAGTAGCCTCCGTTAACCTTCCAATGGCTACTTCAATTAGCAACCTACAGCAGGGTGTAATTAAGACCTACTTCTTATTATTTTTCTTCACCATAATACTGGCAATTCTCATCCTTGTCATCTAACTGCCCGTAAGGTCCCCCGACTTAGCCCTCGAGTTAACTCCAGAACTACAAAAAGCGTCAGTAATAAAACTCATCCCCCAAGCATTAAAACTCCTCCTCCTGAAGAATATATCAGAGCAACCCCACCAAGATCCCCCCGGAATAGCATGAATTCACTAAACTCGTCAGCAGTTATCCATGACCCCTCATACCAACCCTCAGAGAAAAAGACAGAGATAGACGAGACCAGAAACACATATACTGACATAAGAAGCAAAACGGGTCAACTTCCCCACCCCTCAGGATAGGGCTCCGAAGCCAGCGCTGCTGAATACGCAAACACAACTAACATCCCACCTAAATAGATCAAAAACAAAATCAGAGATAAAAATGAACCCCCGTGCCCTACTAAAATGCCACAGCCCATTCCTGCTACTGTGACAAGGCCCAAAGCAGCAAAGTAGGGTGAGGGGTTTGAGGCCACGGCCGCTAGACCTAAAACCAGACCAACTAATAATAAATAAGTTATATAAACCATAATTCTTGCCAGGATTTTAACCAGGGCCTGCGACTTGAAAAACCACCGTTGTACTCAACTACAAGAACCTAATGGCCAATCTTCGAAAAACCCATCCCCTATTAAAAATCGCAAACGATGCCCTGGTTGATCTCCCAGCCCCATCAAACATTTCAGTTTGATGAAACTTCGGGTCTCTTCTAGGACTTTGTTTGGCCGCCCAAATTGTTACGGGCCTTTTCCTTGCAATACATTATACATCAGACATTGCCACAGCATTTTCATCTGTAGCACATATTTGTCGTGACGTCAACTACGGCTGACTAATCCGAAACATGCATGCAAACGGTGCTTCCTTTTTCTTCATTTGCATCTACCTGCACATCGGACGGGGCTTGTATTATGGATCCTACTTATATAAAGAGACATGAAATGTGGGTGTTGTCCTTCTCCTCCTAGTGATAATGACTGCTTTCGTAGGCTACGTCCTACCCTGAGGACAAATGTCATTCTGAGGGGCTACCGTCATTACCAACCTTTTATCAGCCATTCCCTACGTTGGAAACGCCCTAGTTCAATGAATCTGAGGCGGATTTTCAGTAGACAACGCCACCCTTACCCGGTTCTTTGCCTTCCATTTCCTCCTTCCCTTTGTGATTGCTGCTGCTACAGTTGTGCATCTTATCTTCCTCCACGAGACAGGATCGAATAATCCAACGGGCTTAAACTCAGACTCTGACAAAGTATCTTTTCACCCCTACTTTTCTTATAAAGATCTTCTAGGCTTTGCTGCCCTACTAGTAGCCCTTATCTCTTTAGCCCTCTTCTCCCCAAACCTACTCGGAGACCCCGATAACTTTACCCCTGCTAATCCTTTAGTGACTCCACCTCACATCAAACCTGAGTGATACTTCTTGTTCGCTTACGCCATTCTACGATCCATCCCAAACAAACTTGGCGGGGTTCTAGCCCTATTAGCCTCTATTCTAGTCCTCTTTCTTGTTCCCATTCTGCACACATCAAAACAACGAAGCCTAACATTCCGACCCCTAACCCAATTCCTCTTCTGATTGCTAGTCGCCGATGTGATAATTTTAACCTGAATTGGAGGTATGCCTGTAGAACATCCTTACATTATCATTGGACAAATCGCATCCTTCATTTATTTTTCCCTTTTCCTAGTCATAGCGCCTATGGCCGGCCTACTAGAAAACAAAGTCTTAAAATGACAATGCATTAGAAGCTCAGATGAAAGAGCACTGGTCTTGTAAACCAGAGGTCGAAGGTTCAAGCCCTTCCTAGTGCTCAGAGAGAAGGGATTCTAACCCCTGCCCCTGACTCCCAAAGCCAGGATTCTTAGTTAAACTACTCCCTGATTTTCATACACCAGTTTTGCAATCCAGAGCGCATCACTTTTGCCACCAACGTTAAATTAACGTTGCACAAACGTTGCATCAGCGCCCCATGGACACTAAATGACGCGAGGGCGCTAAATAAACACCCCCTACCTCTAGCACCCTTTTAACGATTTCACTTTTTTTTTTTTTTTTGTTTAACGATTACGTTTTTTTTGCGTTCCCGGACTCTGCCAGATTTCGACCGAAGACTGCCAGAATCCGCTCAAAATCCGCTCAAATACCAATATGTATTATCCCCATAAATGGTTTAAACCATTTTTGCCTAGTACACACTGACCATGCAAGTCAATTATATTTACCCCGCGCTCCAGGCCGCAGTACATACACCTACAGTTGGTGTATTTAGCACAAGTGTGCCTCAGCTAGTTTCAAGTCACCCACATCCTTCCTTTAATTGTTACTTAATGTAGTAAGAGCCCACCATCAGTTGATTCCTTAATGTCAACGGTTCTTGAAGGTGAGGGACAAAAATCGTGGGGGTTTCACTTCTTGAATTATTCCTGGCATTTGGCTCTACATCTCAAGGCCATACATTTCTCGTCTCTCACACTTTCACTGGCCCTGACATTGGTTAATGGTGGAGTACATACTCCTCGTTACCCCCCATGCCGAGCGTTCTTTCTAATGGACAACGGGTTTTCCTTTTTTTTTCCTTTTCACTTGGCATTTCACAGTGCATACAAACCTTGATGACAAGGTTGAACATTTAGAAATCGGCCGCAAAGAATATTGGTGAATTATTCAAAGATATTAACAGATGAATTGCATAAGTGATATCAAGAGCATAAATAACCAAATGAAACTAGGAACGTTTCTATAATATGCCCCCCGGCTCCCGCGCGTCAAACCCCCCTACCCCCCTAAACTAGTAAGAAGTCTATTATTCCTGCAAACCCCCCGGAAACAGGAAACCCCCTACTAGCATTTTAGCCCGCCCAAATTTGTGTGTATTTATATTATTTGTAATATTGCAAAA

>GQ 4

GCTAGTGTAGCTTAACTAAAGCATAACACTGAAGATGTTAAGACAAACCTTAGATTGGTTTCACGAGCACAAAAGTTTGGTCCTGACTTTACTATCAACTTTAGCTAAACTTACACATGCAAGTATCCGCAATCCCGTGAGAATGCCCTACAGTTTCCTTAAAGGAAACAAGGAGCTGGTATCAGGCTCAATTACTCCCGCCCATGACACCTTGCTTAGCCACACCCCCAAGGGAACTCAGCAGTGATAGACATTAAGCAATAAGTGAAAACTTGACTTAATTAAAGCTAAGAGAACCGGTTAAACTCGTGCCAGCCACCGCGGTTATACGAGCGGTTCGAGCTGATAGATTACGGCGTAAAGCGTGGTTAATAAGAATGAAACTAAAGTCGAATGTTTTCAAAGCTGTTATACGCACTCGAAAATTAGAAGGTCAGAAACGAAAGTGACTTTAACCCTATGAACCCACGAAAACTATGAAACAAACTGGGATTAGATACCCCACTATGCATAGCTGTAAACTTTGATGAGCTATTACATTATCATCCGCCTGGGTACTACGAGCATCAGCTTAAAACCCAAAGGACTTGGCGGTGCTTTAGACCCACCTAGAGGAGCCTGTTCTAAAACCGATAACCCCCGTTAAACCTCACCCTCTCTTGTTTTTCCCGCCTATATACCGCCGTCGTCAGCTTACCCTGTGAAGGTCTAATAGTAAGCACAACCAGTTATACTCAAAACGTCAGGTCGAGGTGTAGCATATGAGAGGGGAAGAAATGGGCTACATTCCTTGTTTCAAGGAAAACGGATAACATAATGAAAGGTACGTTAGAAGGAGGATTTAGCAGTAAGCAGCAAATAGAGTGTTCTGCTGAAACTGGCCCTGAAGCGCGCACACACCGCCCGTCACTCTCCCCAACTCCGAGTTAAAAACATATATAAACCTTTGAAGGAACAAAGGGGAGGCAAGTCGTAACATGGTAAGTGTACCGGAAGGTGCACTTGGATAAATCAGAGTATAGCTAAGAAAGAAAAGCATCTCCCTTACACCGAGAAGTCATCCGTGCAAATCGGATTACCCTGACTCTAACAAGCTAGCCCAAAACCTTAACTTAAAAATCAAATATTTCTAGTAATTAATAAACCAAACACATTAAATAAATCATTTTTCCCCCTGAGTATGGGAGACAGAAAAGGATAAAGGAGCTATAGACAAAGTACCGCAAGGGAAAGCTGAAAGAGAAATGAAACAAACCAGTAAAGAAAAACAAAGCAGAGATTAACCCTTGTACCTTTTGCATCATGAATTAGCCAGTTTAATCAAGCAAAGAGCACTGTAGTTTGAGACCCCGAAACTTAGTGAGCTACTTCAAGACAGCCTACGAAATAGGGCAAACCCGTCTCTGTGGCAAAAGAGTGGGAAGATCTTCAAGTAGAGGTGACAGACCTATCGAACTAAGTAATAGCTGGTTGCTCGTGAAATGAATAGAAGTTCAGCCTTTTGCTTTCTAAATTTCGATTTAGCATCACTTAGCCTAAATGATTAGAAAACAAAAGAGTTAGTCAAAGAGGGTACAGCCTGTTTGATAAAAGATACAACTTTACTAGGAGGATAAGAATCATAATTTTAAAGGTTTAATGCCCAGGTGGGCCTAAAAGCAGCCACCCTGATCAATAGCGTTAAAGCTTAAGCATAAAACACACCTACAATTCTGATAAATCAGTTTTAATCCCCTAAAGTTAACGAGCTATTTCATACCTTATGAAAGAAATTATGCTAGTATGAGTAATAAGAAGTTACGAACTTCTCCCTGCACACGTGTAAATCGGAACGGACAAACCACCGAATCTTAACGGCCCCAGTCAAAGAGGGGATGTCGGATAAAAAAAAAGAACAAGAAATTCCCGATAAAACCACCGTTGACCCCACACCGGAGTGCTCCCTGGGAAAGACAAAAAGGGACAGAAGGAACTCGGCAAATATGCTCAAGCCTCGCCTGTTTACCAAAAACATCGCCTCTTGTAAAAGTTAAATAAGAGGTACCGCCTGCCCTGTGACTAGTAGTTTAACGGCCGCGGTATTTTGACCGTGCAAAGGTAGCGCAATCACTTGCCTTTTAAATGAAGGCCTGTATGAATGGCACGACGAGGGCTTAACTGTCTCCTCTCCCTAGTCAATGAAATTGATCCCCCCGTGCAGAAGCGGGGATAATAACATAAGACGAGAAGACCCTGTGGAGCTTTAGACTATGAGCAGACCATGTCAAGAATAACAAACAAGTAAATTAAACAAATTGGCCCCTGCTTCCCTGTCTTTGGTTGGGGCGACCGCGGGATAATAAAAAACTCCCACGAGGATTGAGAACCCTTATCTTATAACCAAGAGCTTCTCCTCTAAGTAACAGAACATCTGACCTTAATGATCCGGCCTGGCCGATCAACGGACCGAGTTACCCCAGGGATAACAGCGCAATCCTCTTTTAGAGTCCATATCGACAAGAGGGTTTACGACCTCGATGTTGGATCAGGACATCCTAATGGTGCAGCCGCTATTAAGGGTTTGTTTGTTCAACAATTAAAGTCCTACGTGATCTGAGTTCAGACCGGAGTAATCCAGGTCAGTTTCTATCTATGACGTACTCTCTTCTAGTACGAAAGGACCGAAGAAAGAAGGCCCATGAAAAATTATGCCTTAGTCTCACCTTATGAAGAAAACTAAATAAGACAAGAGGTTACACCCTTTGGTCATAGAAAATGACATGTTAAGGTGGCAGAGCCCGGATATTGCAAAAGACCTAAGCCCTTTCCACAGAGGTTCAATTCCTCTCCTTAACTATGTTCTCAACAATATTAAGCTTCATTATTAATCCCCTGATTGTTATGATTTTTGTTTTGTTGGCAGTAGCCCTCTTGACCTTGGTAGAGCGTAAAGTGCTAAGCTACATGCAACTTCGTAAAGGCCCAAATGTTGTTGGCCCTTACGGCCTTTTGCAACCCTTCGCTGATGGCTTAAAACTTTTCATGAAAGAGCCCGTCCGACCCTCCACCTCCTCGCCCGCCTTGTTCTTAATTACCCCTATTATAGCCCTTACCTTAGCCCTAACCCTCTGAGCCCCCCTTCCTATGCCTTTTCCCGTCACCGACCTAAACTTAGGCATTTTATTTATTTTAGCACTATCGAGCCTGGCAGTATATTCTATTCTTGGCTCCGGATGAGCCTCCAATTCTAAATATGCATTAATTGGTGCTCTTCGAGCGGTCGCCCAAACCATCTCTTATGAAGTAAGCTTGGGCCTTATTCTTCTTAACACAATTGTCTTTACGGGGGGTTTTACTCTTCAAACCTTCAGCACCGCACAAGAAGCCACCTGATTACTTCTACCAGCATGACCACTAGCAGCCATGTGATATATCTCCACACTCGCGGAAACTAACCGGGCCCCTTTCGACCTAACTGAAGGAGAGTCCGAACTAGTGTCTGGCTTCAACGTAGAGTATGCCGGCGGACCTTTTGCCCTTTTTTTTCTGGCAGAATACGGTAACATTTTACTTATAAATACCCTCTCAGCAGTACTATTTCTAGGCTCTTCAACCTACCACAACTTTCCAGAACTAACCGCGACCTTATTAATGCTTAAAGCCACCCTCCTTTCAGTCGTATTTTTATGAGTGCGAGCATCTTACCCTCGGTTCCGATACGACCAACTAATGCATTTAATTTGAAAAAACTTTTTACCTCTGACCCTAGCGCTAGTTATTTGACACCTTTCTCTTCCAATCACGTTGAGCGGCCTCCCTCCTCAACTTTAACTCAGGAAATGTGCCTGAAAAAGGGTCACTTTGATAGGGTGAATAATGAGGGTTAAAGCCCCTCCATCTCCTTAGAAAGAAGGGGTTTGAACCCTACCTGAAGAGATCAAAACTCTTAGTGCTTCCACTACACCACTTCCTAGTAAAGTCAGCTAATAAAAGCTTTTGGGCCCATACCCCAAATATGTTGGTTAAAATCCTTCCTTTGCTAATGAATCCTTACGTCCTTTCAATTCTACTTATGGGCTTAGGCCTCGGCACTACAGTCACATTCGCTAGCTCACACTGACTCTTAGCATGAATAGGCCTTGAAATAAATACCCTCGCCATTTTGCCATTAATAGCACAACATCACCACCCCCGAGCCGTTGAAGCCACCACCAAGTATTTTTTAATTCAATCGGCAGCCGCAGCAACCATCTTATTTGCCAGCTCAACTAACGCCTGACTTTCGGGCCAGTGGGATATCATAAGTATCAATCACCCCCTTCCAACCGTCATAATTACAATCGCTCTGTCCTTAAAACTAGGCTTGGCCCCTCTTCACGCCTGACTTCCCGAAGTTATTCAAGGACTAGACTTGACCACGGGCTTAATCCTCTCCACATGACAAAAACTCGCACCCTTTGCCCTTCTCGTTCAAATCTTCCCCGACACCCCCCTTCTCATCACTTCTCTAGGACTTCTTTCAATATTAATTGGGGGGTGAGGAGGTTTAAACCACACACAACTCCGCAAAGTGCTCGCATATTCTTCGATCGCCCACTTAGGCTGAATAATAGTAATTATGCAATTCTCCACCCCCCTTACAATTCTTGCTTTATCAACATACATTGTTATAACATCATCTACTTTTCTAATCTTTAAACTCCTTAAATCCACAAATATGAACAGCCTGGCAACATCTTGAACTAAAACCCCCTCCATTACAGCCCTAGCACCTTTAGTGCTATTATCCTTAGGCGGACTTCCTCCCCTCTCGGGCTTTATGCCAAAATGATTAATTATTCAAGAATTAACTAAGCAAGATCTAGCCCTAGTTGCAACCTTGGCCGCCCTCTCTGCGCTACTCAGCCTTTTCTTCTACCTACGCATTTGTTACTCCCTCACATTTACCTCCTCTCCTAATAATCTCATGGGAACACCCCCCTGACGACTAGTAACAAAGCAAGTATCACTTCCCCTAGCTATAACAACCTCCCTCTCTATTCTTCTACTCCCGGTTACCCCTGCAATCTTATCAGTGGTTCTCCCTTTGTAAAGAGGCTTAGGATAGTATTAAGACCAAGGGCCTTCAAAGCCCTAAGCGGGAGTGAAAGCCCCCCAGCCTCTGTAAGACCTACGGGACACTAACCCACATCTTCTGTATGCAAAACAGACACTTTAATTAAGCTAAAGCCTTCCTAGGTGGGTAGGCCTCGATCCTACAATCTCTTAGTTAACAGCTAAGCGCCTAAACCAGCGGGCATCCATCTACCTTTCCCCCGCCTTGCCGAAAAAAAAAGGCGGGGGAAAGCCCCGGCAGGGTATTAGCCTGCCACTTAAGATTTGCAATCTAATGTGTTAACACCTCGGGGCTGGTAAGAAGAGGACTTTAACCTCTGTCCATGGGGCTACAATCCACCGCTAAACGCTCAGCCACCTTACCTGTGGCAATCACACGTTGATTTTTCTCAACTAATCACAAAGACATCGGCACCCTGTATCTAATCTTTGGTGCCTGGGCGGGAATGGTAGGGACGGCCTTAAGTCTACTCATTCGGGCAGAACTAAGTCAACCAGGCTCCCTATTAGGAGACGACCAGATCTATAACGTAATTGTAACTGCACATGCTTTCGTAATAATTTTCTTTATAGTAATGCCAATCATAATTGGGGGGTTTGGCAACTGATTAATCCCTTTAATGATTGGAGCCCCCGACATGGCCTTCCCACGGATAAATAATATAAGCTTTTGACTCCTGCCCCCTTCTTTCCTTCTATTATTGGCCTCATCTGGTGTAGAAGCTGGTGCCGGAACAGGATGAACCGTATATCCCCCCTTGTCAGGTAATTTGGCACACGCAGGGGCCTCCGTAGATTTAACCATTTTCTCTCTTCACCTGGCCGGAATTTCTTCTATCCTAGGGGCCATTAATTTCATCACAACTATTATTAATATAAAACCTCCAGCCATTTCCCAATATCAAACCCCTTTATTTGTGTGGGCTGTACTAATTACCGCAGTATTACTTCTACTCTCTCTTCCTGTTCTAGCTGCAGGTATCACCATGCTTCTCACAGATCGGAACCTAAATACAACATTTTTCGACCCCGCAGGAGGGGGGGACCCCATTCTTTATCAACATTTATTCTGATTCTTTGGGCATCCTGAAGTCTACATTCTAATTTTGCCCGGCTTCGGAATGATTTCTCACATTGTAGCATATTACTCAGGCAAAAAAGAGCCGTTTGGTTACATGGGAATAGTATGAGCTATAATAGCAATTGGCTTGCTGGGCTTTATCGTATGAGCCCATCATATGTTCACTGTAGGGATGGACGTGGACACTCGAGCTTATTTTACTTCCGCCACTATAATTATCGCAATTCCCACAGGAGTCAAAGTGTTTAGTTGACTAGCTACCTTGCATGGGGGCTCAATCAAATGAGAAACCCCCCTGTTATGAGCTCTAGGCTTTATCTTCTTATTTACTGTCGGAGGTTTAACAGGAATTGTTTTAGCCAACTCATCTCTGGACATTATACTTCATGATACATACTATGTTGTAGCCCACTTCCACTATGTCCTCTCTATAGGAGCAGTCTTTGCCATCATGGGAGCATTCGTTCACTGATTTCCCCTATTCTCAGGCTACACCCTTCACAATACGTGAACAAAAATCCACTTCGGAGTTATGTTTGTAGGTGTAAACCTCACCTTTTTCCCTCAGCACTTCTTAGGATTGGCGGGAATACCTCGACGATACTCAGATTACCCTGACGCATACACACTGTGAAATACTATCTCATCCCTGGGGTCATTAATCTCCCTTATTGCTGTAATTATATTCCTATTTATTATCTGGGAGGCATTCGCGGCAAAACGTGAAGTCTTATCAGTTGAACTAACAGCCACAAACGTAGAATGACTGCACGGGTGTCCTCCCCCTTACCATACATTTGAAGAACCTGCATTCGTTCAAATTCAACAATCCAAATTTTAATCGAGAAAGGAAGGAGTCGAACCCCCATAAACTGGTTTCAAGCCAGCCACATAACCGCTCTGTCACTTTCTTCCCTAAGTTAATAAGATTCTAGTTAAAGGAATAACACTGCCTTGTCAAGGCAAAATTGTGGGTTAAAGCCCCACGTATCTTGCTTATGGCACATCCATCTCAACTAGGATTCCAAGATGCAGCTTCACCCGTTATAGAAGAACTTCTCCATTTTCATGACCATGCATTAATAATTGTTTTCTTAATCAGCACCCTTGTTCTTTACATTATTGTGGCTATGGTAACCACCAAGCTAACAAATAAGTTCATTCTGGACTCCCAAGAAATTGAAATCATCTGAACCTTGCTACCAGCAATTATCCTAATTCTGATCGCCCTACCCTCCCTTCGCATTCTCTACCTCATGGATGAAATCAATGACCCCCACCTCACAATTAAAGCCATAGGACATCAATGATACTGAAGCTACGAATATACGGATTATGAAGACCTAGGGTTCGACTCGTATATGGTCCCTACACAAGACCTCGCCCCTGGTCAATTTCGACTACTTGAAACAGACCATCGCATGGTCATTCCTGTTGAGTCCCCCATCCGGGTTCTTGTCTCCGCCGAGGATGTTCTACACTCATGGGCCGTCCCAAGCCTTGGAGTAAAAATGGACGCCGTCCCCGGCCGCCTAAATCAAACAGCCTTCATTACTTCCCGTCCGGGTGTGTTTTATGGACAATGCTCAGAAATTTGCGGAGCTAATCATAGCTTTATACCCATTGTAGTGGAAGCTGTTCCTCTAGAACACTTCGAGAACTGATCTTACCTAATACTTCAAGATGCCTCACCAGGAAGCTAAAAGGGGATAGCATTAGCCTTTTAAGCTAAAAATTGGTGACTCCCGCCCACCCCTGGTGACATGCCTCAGTTGAACCCCGCACCCTGATTTGCTATTATAGTATTCTCATGACTAGTTTTCCTAGCCGTTATCCCACCTAAAGTTTTAGCTCACCATTTTCCCAATGACCCCGCCCCACAAAGCGTAAAAAAATCAAAAACAGAGACCTGATCCTGACCATGACTTTAAGCCTCTTTGATCAATTTATGAGCCCTACACTTCTAGGGGTGCCTCTTATCGGACTCGCCCTAACATTGCCATGAGTCCTTTACTTCCAACCCGGTGCCCGATGACTTAATAACCGCTTCATTACCCTTCAATCTATATTCATGAACTGATTTGTAAAACAAATCTTTCAGCCAATAAGCTTAGGCGGACACAAATGGGCCGCTCTCCTCATATCTTTAATACTATTTTTAATTACCTTAAATATGCTAGGCCTACTGCCTTATACATTTACTCCAACAACGCAGCTGTCACTTAATATAGCCTTTGCAGTACCACTTTGACTAGCAACTGTCATTATTGGAATACGAAATCAGCCAACACATGCCCTTGGTCACCTCCTCCCCGAAGGAACTCCTACCGCCCTAATCCCGGTTTTAATCGTGATTGAGACAATTAGCCTTTTTATTCGACCATTGGCCCTCGGTGTTCGACTTACCGCAAACTTGACAGCCGGACACCTTCTAATTCAACTAATTGCAACTGCGGCTTTTGTTCTTTTCCCTATAATGCCCACAGTCGCCGCTCTTACCTCTGTCTTACTATTGTTGCTAACCCTACTAGAAGTCGCCGTGGCCATAATCCAAGCCTATGTATTCGTACTTCTTTTAAGCCTTTACCTACAAGAAAACGTCTAATGGCCCATCAAGCACATGCATATCATATAGTTGACCCAAGCCCTTGACCCCTCACAGGCGCAGTAGCCGCCCTTCTACTTACATCTGGAACAGCAATCTGAATACACTTTAACTCCACGGTTCTCATGTCCCTTGGACTTGTTCTGTTACTACTAACCATATATCAATGATGGCGAGACATTATCCGAGAGGGCACCTTTCAAGGTCATCATACACCCCCTGTTCAAAAAGGCCTTCGGTACGGGATAATTCTGTTTATTACCTCAGAGGTCTTCTTTTTCCTAGGTTTCTTCTGAGCATTTTATCACTCAAGCCTAGCCCCAACCCCCGAACTTGGTGGATGTTGACCACCCATGGGTATTACAACACTGGACCCCTTTGAAGTTCCCCTTCTCAATACTGCTGTCCTTCTCGCCTCCGGTGTCACGGTCACTTGAGCTCACCATAGTATTATGGAGGGGCAGCGAAAACAAGCAATTCAGTCCTTGACACTCACAATTCTCCTGGGGTTTTACTTTACATTCCTTCAAGCAATAGAGTACTACGAGGCACCCTTCACCATTGCAGATGGCGTCTATGGCTCTACATTTTTTGTGGCAACAGGGTTTCATGGCCTCCATGTAATTATTGGGTCAACATTTCTGGCAGTCTGCCTCTTACGACAAGTCCAGTTCCATTTTACATCAGAACATCACTTCGGATTCGAAGCTGCAGCATGATACTGACACTTTGTAGACGTAGTCTGACTATTCTTATATATCTCTATCTACTGATGAGGCTCATATCTTTCTAGTATTAAAAAGTACAAGTGACTTCCAATCACTCAGTCTTGGTTAGACTCCAAGGAAAGATAATGAACTTAGTACTAGTCATTATTTGCATCTCATTAGCCCTCGCCGCACTGCTCGCAACTGTTTCATTTTTCCTCCCACAAATAACCCCTGATTATGAGAAACTCTCACCGTATGAGTGCGGCTTTGATCCAGTGGGATCCGCCCGTTTGCCATTCTCCATTCGCTTTTTTCTAGTCGCAATCCTATTTCTCCTCTTCGACTTAGAAATTGCCTTACTTCTTCCCCTTCCCTGAGGGGACCAACTTCCCTCCCCTCTAACAACTTTCTTTTGAGCTTCTGCTATTCTTATACTACTAACTCTAGGGTTAATCTATGAATGACTTCAAGGGGGCCTAGAGTGGGCAGAATAGGTACTTAGTTTAATAAAAACATTTGATTTCGGCTCAAAAACTTATGGTTTAAGTCCATATTTACCTGATGACCTTAACTCACTATGCATTCTCGTCAGCCTACTTTGTTAGCTTCATGGGTTTAATTTTTTACCGAAAGCATCTTCTCTCCGCCTTACTTTGCTTAGAAGCGATAATACTTATTCTTTTTATTTCACTATGCCTGTGAGGTCTAGTCTTAGCCTCAAGTGCATTTTCGGCAGGCCCAATGATCTTACTTGCTTTCTCAGCATGTGAAGCAAGTGCAGGCCTAGCACTGCTTGTAGCAATAGCTCGAACCCACGGTACTGACCGTTTAAAAAACCTTAGCCTACTCCAATGTTAATAATTCTTATTCCTACTGTTATGCTTCTACCCACAATCTGACTGAGCCCCACTAAATACCTGTGATCCTCAACACTCGGCCATAGCATAATGATTGCTCTTATAAGCCTCTCCTGACTTAGCCTCCCAGGGGAGGTTGGCTGATCTTCCCTTAACACTTTTATAGCAACAGACCCTCTCTCTACCCCCCTTCTCGTACTTACTTGCTGACTTCTGCCCTTAATAATTCTTGCGAGCCAAAACCATATAGCCCAAGAACCTACCAATCGCCAGCGAACCTATATCTCTCTCCTTACTTCCCTTCAAATCTTCTTAATCTTAGCATTTGGGGCAACCGAGATAATCATGTTCTACATTATATTTGAAGCGACCTTAATTCCCACACTCGTAATTATCACACGATGAGGAAACCAAACAGAACGATTAAACGCAGGTATTTACTTTTTATTTTATACCTTAGCCGGCTCTTTACCACTACTAGTGGCCCTCCTTCTACTTCAGACCTCGACAGGAACTCTTTCTTTTCTAACCACTCAATTTTTTCCCCCTTTACAACTGCATACAGAAGCAAGTAAATTCTGGTGGGCAGGCTGTTTACTAGCATTCTTAGTAAAAATGCCCCTATATGGAGCACACCTTTGACTTCCAAAAGCTCACGTCGAAGCCCCCATCGCCGGGTCAATAGTCCTTGCAGCCGTTCTTTTAAAACTAGGGGGTTACGGTATGATACGAGTTATTATTATCTTAGAGCCCCTCACGAAACAACTCAGCTACCCCTTTATTGTTCTTGCCCTGTGGGGCGTCGTAATAACTGGCTCAATTTGCCTCCGACAAACAGACCTTAAATCACTAATCGCTTACTCCTCAGTAAGCCACATAGGCCTTGTCGCAGCAGGCATCCTAATCCAAACTCCTTGGGGGTTTACAGGAGCATTAATCCTTATAATTGCCCATGGCTTAACTTCCTCCGCCCTATTCTGTTTAGCCAACACTAACTATGAGCGAACACATAGCCGAACCATGCTTTTAGCCCGGGGTCTACAAATGGTCCTTCCTCTCTTAGCAACTTGATGGTTTCTATTTACCCTCGCCAACCTAGCACTCCCTCCACTACCCAACCTCATAGGAGAACTTATGATTATCTCATCCTTGTATAACTGGTCAAACTGGTCTCTAATCCTGACCGGGGCGGGAGTACTAATTACCGCTAGTTACTCTCTCCATATATTCCTAACCACTCAACGTGGCCCTATTACTAACCCCGTCTTGGCAATTGAACCAACCCATACACGAGAACATCTCCTCATAATCCTTCACCTTCTTCCCCTCCTCCTTCTAATTTTAAAACCCTGCTTGATCTGGGGCTGAACAGCTTGTAGGCGTAGTTTAAATAAAGCGCTAGATTGTGATTCTAGAAATAAGAGTTAAACCCTCTTCACCCACCGAGAGGGGTCGCCGTGACAGCAAGAACTGCTAATTCTAGCCCCTTTGGTTAAAGTCCGAAGCCCACTCGAACAGGCTTCTAAAGGATAACAGCTCATCCGTTGGTCTTAGGAACCAAAAACTCTTGGTGCAACTCCAAGTAGCAGCTATGCACTTTACAACAATGATTCTCTCCTCAAGCCTAATAACAATTTTCCTTCTTCTTATCCTTCCAGTCCTAGGTACACTAAATCCTAACCCCACAGGGGACCTGTGGGCCACAAAAAACGTTAAAACAGCAGTTAAGATGGCCTTTTTTGTAAGCCTTCTACCTCTTTTTATCTTTCTTAATGAAGGAGTAGAGACTATTATAACAAACTGAAAATGGATAAATACTCTAATATTTGAAATTAATATCAGCTTTAAATTTGACCTCTACTCTGTGGTATTTACCCCTGTAGCCCTCTACGTAACATGATCAATTTTAGAGTTCGCATCTTGGTACATACACAGTGACCCCAATATAAACCGATTCTTTAAGTATCTTCTAATCTTTCTAATCGCTATGGTTGTTCTGGTTACAGCCAACAACATGTTCCAACTATTTATTGGCTGAGAAGGTGTTGGAATTATGTCCTTCTTACTTATTGGCTGGTGGTTCGGGCGGGCTGACGCCAACACTGCGGCCCTCCAAGCCGTAGTTTATAACCGAGTCGGTGATATCGGCCTAATTCTAGCAATAGCATGAATAGTAGTAAACCTAAACTCATGAGAGATACAACAGCTCTTTTCTGTGTCTAAAGGCCATGACATGACCCTTCCCTTATTAGGCCTAGTACTGGCCGCTACCGGAAAGTCCGCCCAGTTTGGGCTTCACCCCTGGCTCCCCTCAGCCATAGAGGGTCCAACACCGGTCTCTGCCCTCCTGCACTCTAGCACCATGGTTGTTGCTGGTATTTTCCTTCTTATCCGCCTCAGCCCCTTAATGCAAGAAAGCCCGTTAATTCTCTCAACATGCCTTTGCCTGGGGGCCCTAACTACCGTCTTTACTGCGACATGTGCCCTTACCCAAAATGACATCAAAAAAATTGTTGCATTTTCTACATCAAGTCAACTAGGACTAATAATAGTTACCATCGGACTAGGCCAGCCCCAGCTCGCCTTTCTTCATATCTGCACCCACGCCTTCTTTAAAGCAATACTTTTCTTATGTTCAGGCTCCATCATTCATAGCCTTAATGATGAACAAGATATCCGAAAAATAGGAGGACTTCACAAGCTCCTTCCACTGACCTCTTCTTGTCTAACCATTGGCAGCCTAGCTCTAACAGGAGTCCCCTTTTTAGCAGGCTTCTTTTCCAAAGACGCCATCATTGAAGCTATAAATACATCCTACCTTAACGCCTGGGCCCTAATTTTAACGCTTCTAGCTACATCATTTACCGCAGTTTACAGTCTCCGAGTCGTATTCTTTGCCTCTATGGGCCACCCGCGTTTTAATCCAATCTCCCCAATTAATGAAAATAACCCTACAGTAATAAACCCTATTAAACGACTCGCTTGAGGAAGCATTTTGGCAGGGTTGCTAATTACGGCCAATATTGTTCCACTTAAAACCCCCGTTTTAACCATGCCTTTCACCTTAAAAATGGCCGCACTGGCTGTAACAATTATAGGACTACTCACAGCCTTAGAACTAGCATCTCTCACGTCCCAACAATTTAAAATCAAACCCTTATCTTCTACTCACCACTTCTCAAATATATTAGGATTTTTCCCGAGTGTAGCCCATCGACTAGTCCCAAAAACTGGCCTGATTCTTGGGCAACTGGTTGCCAATCAGACAATTGACCAAACCTGACTAGAGAAAACCGGGCCAAAAATAGTAGCCTCCGTTAACCTTCCAATGGCTACTTCAATTAGCAACCTACAGCAGGGTGTAATTAAGACCTACTTCTTATTATTTTTCTTCACCATAATACTAGCAATTCTCATCCTTGTCATCTAACTGCCCGTAAGGTCCCCCGACTTAGCCCTCGAGTTAACTCCAGAACTACAAAAAGCGTCAGTAATAAAACTCATCCCCCAAGCATTAAAACTCCTCCTCCTGAAGAATATATCAGAGCAACCCCCCCAAGATCCCCCCGGAATAGCATGAATTCACTAAACTCGTCAGCAGTTATCCATGACCCCTCATACCAACCCTCAGAGAAAAAGACAGAAATAGACGCGACCAGAAACACATATACTGACATAAGAAGCAAAACGGGTCAACTTCCCCACCCCTCAGGATAGGGCTCCGAAGCCAGCGCTGCTGAATACGCAAACACAACTAACATCCCGCCTAAATAGATCAAAAACAAAATCAGAGATAAAAATGAACCCCCGTGCCCTACTAAAATGCCACAGCCCATTCCTGCTACTGTGACAAGGCCCAAAGCAGCAAAGTAGGGTGAGGGGTTTGAGGCCACGGCCGCTAGGCCTAAAACCAGGCCAACTAATAATAAATAAGTTATATAAACCATAATTCTTGCCAGGATTTTAACCAGGGCCTGCGACTTGAAAAACCACCGTTGTACTCAACTACAAGAACCTAATGGCCAATCTTCGAAAAACCCATCCCCTATTAAAAATCGCAAACGATGCCCTGGTTGATCTCCCAGCCCCGTCAAACATTTCAGTTTGATGAAACTTCGGGTCTCTTCTAGGACTTTGTTTGGCCGCCCAGATTATTACGGGCCTTTTCCTTGCAATACATTATACATCAGACATTGCCACAGCATTTTCATCTGTAGCACATATTTGTCGTGACGTCAACTACGGCTGACTAATCCGAAACATGCATGCAAACGGTGCTTCCTTTTTCTTCATTTGCATCTACCTGCACATCGGACGGGGCTTGTATTATGGATCATACTTATATAAAGAGACATGAAATGTGGGTGTTGTCCTTCTCCTCCTAGTGATAATGACTGCTTTCGTAGGCTACGTCCTACCCTGAGGACAAATGTCATTCTGAGGGGCTACCGTCATTACCAACCTTTTATCAGCCATTCCCTACGTTGGAAACGCCCTAGTTCAATGAATCTGAGGCGGATTTTCAGTAGACAACGCCACCCTTACCCGGTTCTTTGCCTTCCATTTCCTCCTTCCCTTTGTGATTGCTGCTGCTACAGTTGTGCATCTTATCTTCCTCCACGAGACAGGATCGAATAATCCAACAGGTTTAAACTCAGACTCTGACAAAGTATCTTTTCACCCCTACTTTTCTTATAAAGATCTTCTAGGCTTTGCTGCCCTACTAGTAGCCCTTATCTCTTTAGCCCTCTTCTCCCCAAATCTACTGGGAGACCCCGATAACTTTACCCCTGCTAATCCTTTAGTGACGCCACCTCACATCAAACCTGAGTGATACTTCTTGTTCGCTTACGCCATTCTACGATCCATCCCAAACAAACTTGGCGGGGTTCTAGCCCTATTAGCCTCTATTCTAGTCCTCTTTCTTGTTCCCATTCTGCACACATCAAAACAGCGAAGCCTAACATTCCGACCCCTAACCCAATTCCTCTTCTGATTGCTAGTCGCCGATGTAATAATTTTAACCTGAATTGGAGGTATGCCTGTAGAACATCCTTACATTATCATTGGACAAATCGCATCCTTCATTTATTTTTCCCTTTTCCTAGTCATAGCGCCTATGGCCGGCCTACTAGAAAACAAAGTCTTAAAATGACAATGCATTAGAAGCTCAGATGAAAGAGCACCGGTCTTGTAAACCAGAGGTCGAAGGTTCAAGCCCTTCCTAGTGCTCAGAGAGAAGGGATTCTAACCCCTGCCCCTGACTCCCAAAGCCAGGATTCTTAACTAAACTACTCCCTGATTTTCATACACCAGTTTTGTAATCCAGAGCGCATCACTTTTGCCACCAACGTTAAATTAACGTTGCACAAACGTTGCATCAGCGCCCCATGGACACTAAATGACGCGAGGGCGCTAAATAAACACCCCCTACCTCTAGCACCCTTTTAACGATTTCACTTTTTTTTTTTTTTTTGTTTAACGATTACGTTTTTTTTTGCGTTCCCGGACTCTGCCAGATTTCGACCGAAGACTGCCAGAATCCGCTCAAAATCCGCTCAAATACCAATATGTATTATCCCCATAAATGGTTTAAACCATTTTTGCCTAGTACACACTGACCATGCAAGTCAATTATATTTACCCCGCGCTCCAGGCCGCAGTACATACACCTACAGTTGGTGTATTTAGCACAAGTGTGCCTCAGCTAGTTTCAAGTCACCCACATCCTTCCTTTAATTGTTACTTAATGTAGTAAGAGCCCACCATCAGTTGATTCCTTAATGTCAACGGTTCTTGAAGGTGAGGGACAAAAATCGTGGGGGTTTCACTTCTTGAATTATTCCTGGCATTTGGCTCTACATCTCAAGGCCATACATTTCTCGTCTCTCACACTTTCACTGGCCCTGACATTGGTTAATGGTGGAGTACATACTCCTCGTTACCCCCCATGCCGGGCGTTCTTTCTAATGGACAACGGGTTTTCCTTTTTTTTTCCTTTTCACTTGGCATTTCACAGTGCATACAAACCTTGATGACAAGGTTGAACATTTAGAAATCGGCCGCAAAGAATATTGGTGAATTATTCAAAGATATTAACAGATGAATTGCATAAGTGATATCAAGAGCATAAATAACCAAATGAAACTAGGAACGTTTCTATAATATGCCCCCCGGCTCCCGCGCGTCAAACCCCCCTACCCCCCTAAACTAGTAAGAAGTCTATTATTCCTGCAAACCCCCCGGAAACAGGAAACCCCCTACTAGCATTTTAGCCCGCCCAAATTTGTGTGTATTTATATTATTTGTAATATTGCAAAA

>GQ 8

GCTAGTGTAGCTTAACTAAAGCATAACACTGAAGATGTTAAGACAAACCTTAGATTGGTTTCACGAGCACAAAAGTTTGGTCCTGACTTTACTATCAACTTTAGCTAAACTTACACATGCAAGTATCCGCAATCCCGTGAGAATGCCCTACAGTTTCCTTAAAGGAAACAAGGAGCTGGTATCAGGCTCAATTACTCCCGCCCATGACACCTTGCTTAGCCACACCCCCAAGGGAACTCAGCAGTGATAGACATTAAGCAATAAGTGAAAACTTGACTTAATTAAAGCTAAGAGAACCGGTTAAACTCGTGCCAGCCACCGCGGTTATACGAGCGGTTCGAGCTGATAGATTACGGCGTAAAGCGTGGTTAATAAGAATGAAACTAAAGTCGAATGTTTTCAAAGCTGTTATACGCACTCGAAAATTAGAAGGTCAGAAACGAAAGTGACTTTAACCCTATGAACCCACGAAAACTATGAAACAAACTGGGATTAGATACCCCACTATGCATAGCTGTAAACTTTGATGAGCTATTACATTATCATCCGCCTGGGTACTACGAGCATCAGCTTAAAACCCAAAGGACTTGGCGGTGCTTTAGACCCACCTAGAGGAGCCTGTTCTAGAACCGATAACCCCCGTTAAACCTCACCCTCTCTTGTTTTTCCCGCCTATATACCGCCGTCGTCAGCTTACCCTGTGAAGGTCTAATAGTAAGCACAACCAGTTATACTCAAAACGTCAGGTCGAGGTGTAGCATATGAGAGGGGAAGAAATGGGCTACATTCCTTGTTTCAAGGAAAACGGATAACATAATGAAAGGTACGTTAGAAGGAGGATTTAGCAGTAAGCAGCAAATAGAGTGTTCTGCTGAAACTGGCCCTGAAGCGCGCACACACCGCCCGTCACTCTCCCCAACTCCGAGTTAAAAACATATATAAACCTTTGAAGGAACAAAGGGGAGGCAAGTCGTAACATGGTAAGTGTACCGGAAGGTGCACTTGGATAAATCAGAGTATAGCTAAGAAAGAAAAGCATCTCCCTTACACCGAGAAGTCATCCGTGCAAATCGGATTACCCTGACTCTAACAAGCTAGCCCAAAACCTTAACTTAAAAATCAAATATTTCTAGTAATTAATAAACCAAACACATTAAATAAATCATTTTTCCCCCTGAGTATGGGAGACAGAAAAGGATAAAGGAGCTATAGACAAAGTACCGCAAGGGAAAGCTGAAAGAGAAATGAAACAAACCAGTAAAGAAAAACAAAGCAGAGATTAACCCTTGTACCTTTTGCATCATGAATTAGCCAGTTTAATCAAGCAAAGAGCACTGTAGTTTGAGACCCCGAAACTTAGTGAGCTACTTCAAGACAGCCTACGAAATAGGGCAAACCCGTCTCTGTGGCAAAAGAGTGGGAAGATCTTCAAGTAGAGGTGACAGACCTATCGAACTAAGTTATAGCTGGTTGCTCGTGAAATGAATAGAAGTTCAGCCTTTTGCTTTCTAAATTTCGATTTAGCATCACTTAGCCTAAATGATTAGAAAACAAAAGAGTTAGTCAAAGAGGGTACAGCCTGTTTGATAAAAGATACAACTTTACTAGGAGGATAAGAATCATAATTTTAAAGGTTTAATGCCCAGGTGGGCCTAAAAGCAGCCACCCTGATCAATAGCGTTAAAGCTTAAGCATAAAACACACCTACAATTCTGATAAATCAGTTTTAATCCCCTAAAGTTAACGAGCTATTTCATACCTTATGAAAGAAATTATGCTAGTATGAGTAATAAGAAGTTACGAACTTCTCCCTGCACACGTGTAAATCGGAACGGACAAACCACCGAATCTTAACGGCCCCAGTCAAAGAGGGGATGTCGGATAAAAAAAAGAACAAGAAATTCCCGATAAAACCACCGTTGACCCCACACCGGAGTGCTCCCTGGGAAAGACAAAAAGGGACAGAAGGAACTCGGCAAATATGCTCAAGCCTCGCCTGTTTACCAAAAACATCGCCTCTTGTAAAAGTTAAATAAGAGGTACCGCCTGCCCTGTGACTAGTAGTTTAACGGCCGCGGTATTTTGACCGTGCAAAGGTAGCGCAATCACTTGCCTTTTAAATGAAGGCCTGTATGAATGGCACGACGAGGGCTTAACTGTCTCCTCTCCCTAGTCAATGAAATTGATCCCCCCGTGCAGAAGCGGGGATAATAACATAAGACGAGAAGACCCTGTGGAGCTTTAGACTATGAGCAGACCATGTCAAGAATAACAAACAAGTAAATTAAACAAATTGGCCCCTGCTTCCCTGTCTTTGGTTGGGGCGACCGCGGGATAATAAAAAACTCCCACGAGGATTGAGAACCCTTATCTTATAACCAAGAGCTTCTCCTCTAAGTAACAGAACATCTGACCTTAATGATCCGGCCTGGCCGATCAACGGACCGAGTTACCCCAGGGATAACAGCGCAATCCTCTTTTAGAGTCCATATCGACAAGAGGGTTTACGACCTCGATGTTGGATCAGGACATCCTAATGGTGCAGCCGCTATTAAGGGTTTGTTTGTTCAACAATTAAAGTCCTACGTGATCTGAGTTCAGACCGGAGTAATCCAGGTCAGTTTCTATCTATGACGTACTCTCTTCTAGTACGAAAGGACCGAAGAAAGAAGGCCCATGAAAAATTATGCCTTAGTCTCACCTTATGAAGAAAACTAAATAAGACAAGAGGTTACACCCCTTGGTCATAGAAAATGACATGTTAAGGTGGCAGAGCCCGGATATTGCAAAAGACCTAAGCCCTTTCCACAGAGGTTCAATTCCTCTCCTTAACTATGTTCTCAACAATATTAAGCTTCATTATTAATCCCCTGATTGTTATGATTTTTGTTTTGTTGGCAGTAGCCCTCTTGACCTTGGTAGAGCGTAAAGTGCTAAGCTACATGCAACTTCGTAAAGGCCCAAATGTTGTTGGCCCTTACGGCCTTTTGCAACCCTTCGCTGATGGCTTAAAACTTTTCATGAAAGAGCCCGTCCGACCCTCCACCTCCTCGCCCGCCTTGTTCTTAATTACCCCTATTATAGCCCTTACCTTAGCCCTAACCCTCTGAGCCCCCCTTCCTATGCCTTTTCCCGTCACCGACCTAAACTTAGGCATTTTATTTATTTTAGCACTATCGAGCCTGGCAGTATATTCTATTCTTGGCTCCGGATGAGCCTCCAACTCTAAATATGCATTAATTGGTGCTCTTCGAGCGGTCGCCCAAACCATCTCTTATGAAGTAAGCTTGGGCCTTATTCTTCTTAACACAATTGTCTTTACGGGGGGTTTTACTCTTCAAACCTTCAGCACCGCACAAGAAGCCACCTGATTACTTCTACCAGCATGACCACTAGCAGCCATGTGATATATCTCCACACTCGCGGAAACTAACCGGGCCCCTTTCGACCTAACTGAAGGAGAGTCCGAGCTAGTGTCTGGCTTCAACGTAGAGTATGCCGGCGGACCTTTTGCCCTTTTTTTTCTGGCAGAATACGGTAACATTTTACTTATAAATACCCTCTCAGCAGTACTATTTCTAGGCTCTTCAACCTACCACAACTTTCCAGAACTAACCGCGACCTTATTAATGCTTAAAGCCACCCTCCTTTCAGTCGTATTTTTATGAGTGCGAGCATCTTACCCTCGGTTCCGATACGACCAACTAATGCATTTAATTTGAAAAAACTTTTTACCTCTGACCCTAGCGCTAGTTATTTGACACCTTTCTCTTCCAATCACGTTGAGCGGCCTCCCTCCTCAACTTTAACTCAGGAAATGTGCCTGAAAAAGGGTCACTTTGATAGGGTGAATAATGAGGGTTAAAGCCCCTCCATCTCCTTAGAAAGAAGGGGTTTGAACCCTACCTGAAGAGATCAAAACTCTTAGTGCTTCCACTACACCACTTCCTAGTAAAGTCAGCTAATAAAAGCTTTTGGGCCCATACCCCAAATATGTTGGTTAAAATCCTTCCTTTGCTAATGAATCCTTACGTCCTTTCAATTCTACTTATGGGCTTAGGCCTCGGCACTACAGTCACATTCGCTAGCTCACACTGACTCTTAGCATGAATAGGCCTTGAAATAAATACCCTCGCCATTTTGCCATTAATAGCACAACATCACCACCCCCGAGCCGTTGAAGCCACCACCAAGTATTTTTTAATTCAATCGGCAGCCGCAGCAACCATCTTATTTGCCAGCTCAACTAACGCCTGACTTTCGGGCCAGTGGGATATCATAAGTATCAATCACCCCCTTCCAACCGTCATAATTACAATCGCTCTATCCTTAAAACTAGGCTTGGCCCCTCTTCACGCCTGACTTCCCGAAGTTATTCAAGGACTAGACTTGACCACGGGCTTAATCCTCTCCACATGACAAAAACTCGCACCCTTTGCCCTTCTCGTTCAAATCTTCCCCGACACCCCCCTTCTCATCACTTCTCTAGGACTTCTTTCAATATTAATTGGGGGGTGAGGAGGTTTAAACCACACACAACTCCGCAAAGTGCTCGCATATTCTTCGATCGCCCACTTAGGCTGAATAATAGTAATTATGCAATTCTCCACCCCCCTTACAATTCTTGCTTTATCAACATACATTGTTATAACATCATCTACTTTTCTAATCTTTAAACTCCTTAAATCCACAAATATGAACAGCCTGGCAACATCTTGAGCTAAAACCCCCTCCATTACAGCCCTAGCACCTTTAGTGCTATTATCCTTAGGCGGACTTCCTCCCCTCTCGGGCTTTATGCCAAAATGATTAATTATTCAAGAATTAACTAAGCAAGATCTAGCCCTAGTTGCAACCTTGGCCGCCCTCTCTGCGCTACTCAGCCTTTTCTTCTACCTACGCATTTGTTACTCCCTCACATTTACCTCCTCTCCTAATAATCTCATGGGAACACCCCCCTGACGACTAGTAACAAAGCAAGTATCACTTCCCCTAGCTATAACAACCTCCCTCTCTATTCTTCTACTCCCGGTTACCCCTGCAATCTTATCAGTGGTTCTCCCTTTGTAAAGAGGCTTAGGATAGTATTAAGACCAAGGGCCTTCAAAGCCCTAAGCGGGAGTGAAAGCCCCCCAGCCTCTGTAAGACCTACGGGACACTAACCCACATCTTCTGTATGCAAAACAGACACTTTAATTAAGCTAAAGCCTTCCTAGGTGGGTAGGCCTCGATCCTACAATCTCTTAGTTAACAGCTAAGCGCCTAAACCAGCGGGCATCCATCTACCTTTCCCCCGCCTTGCCGAAAAAAAAAGGCGGGGGAAAGCCCCGGCAGGGTATTAGCCTGCCACTTAAGATTTGCAATCTAATGTGTTAACACCTCGGGGCTGGTAAGAAGAGGACTTTAACCTCTGTCCATGGGGCTACAATCCACCGCTAAACGCTCAGCCACCTTACCTGTGGCAATCACACGTTGATTTTTCTCAACTAATCACAAAGACATCGGCACCCTGTATCTAATCTTTGGTGCCTGGGCGGGAATGGTAGGGACGGCCTTAAGTCTACTCATTCGGGCAGAACTAAGTCAACCAGGCTCCCTATTAGGAGACGACCAGATCTATAACGTAATTGTAACTGCACATGCTTTCGTAATAATTTTCTTTATAGTAATGCCAATCATAATTGGGGGGTTTGGCAACTGATTAATCCCTTTAATGATTGGAGCCCCCGACATGGCCTTCCCACGGATAAATAATATAAGCTTTTGACTCCTGCCCCCTTCTTTCCTTCTATTATTGGCCTCATCTGGTGTAGAAGCTGGTGCCGGAACAGGATGAACCGTATATCCCCCCTTGTCAGGTAATTTGGCACACGCAGGGGCCTCCGTAGATTTAACCATTTTCTCTCTTCACCTGGCCGGAATTTCTTCTATCCTAGGGGCCATTAATTTCATCACAACTATTATTAATATAAAACCTCCAGCCATTTCCCAATATCAAACCCCCTTATTTGTGTGGGCTGTACTAATTACCGCAGTATTACTTCTACTCTCTCTTCCTGTTCTAGCTGCAGGTATCACCATGCTTCTCACAGATCGGAACCTAAATACAACATTTTTCGACCCCGCAGGAGGGGGGGACCCCATTCTTTATCAACATTTATTCTGATTCTTTGGGCATCCTGAAGTCTACATTCTAATTTTGCCCGGCTTCGGAATGATTTCTCACATTGTAGCATATTACTCAGGCAAAAAAGAGCCGTTTGGTTACATGGGAATAGTATGAGCTATAATAGCAATTGGCTTGCTGGGCTTTATCGTATGAGCCCATCATATGTTCACTGTAGGGATGGACGTGGACACTCGAGCTTATTTTACTTCCGCCACTATAATTATCGCAATTCCCACAGGAGTCAAAGTGTTTAGTTGACTAGCTACCTTGCATGGGGGCTCAATCAAATGAGAAACCCCCCTGTTATGAGCTCTAGGCTTTATCTTCTTATTTACTGTCGGAGGTTTAACAGGAATTGTTTTAGCCAACTCATCTCTGGACATTATACTTCATGATACATACTATGTTGTAGCCCACTTCCACTATGTCCTCTCTATAGGAGCAGTCTTTGCCATCATGGGAGCATTCGTTCACTGATTTCCCCTATTCTCAGGCTACACCCTTCACAATACGTGAACAAAAATCCACTTCGGAGTTATGTTTGTAGGTGTAAACCTCACCTTTTTCCCTCAGCACTTCTTAGGATTGGCGGGAATACCTCGACGATACTCAGATTACCCTGACGCATACACACTGTGAAATACTATCTCATCCCTGGGGTCATTAATCTCCCTTATTGCTGTAATTATATTCCTATTTATTATCTGGGAGGCATTCGCGGCAAAACGTGAAGTCTTATCAGTTGAACTAACAGCCACAAACGTAGAATGACTGCACGGGTGTCCTCCCCCTTACCATACATTTGAAGAACCTGCATTCGTTCAAATTCAACAATCCAAATTTTAATCGAGAAAGGAAGGAGTCGAACCCCCATAAACTGGTTTCAAGCCAGCCACATAACCGCTCTGTCACTTTCTTCCCTAAGTTAATAAGATTCTAGTTAAAGGAATAACACTGCCTTGTCAAGGCAAAATTGTGGGTTAAAGCCCCACGTATCTTGCTTATGGCACATCCATCTCAACTAGGATTCCAAGATGCAGCTTCACCCGTTATAGAAGAACTTCTCCATTTTCATGACCATGCATTAATAATTGTTTTCTTAATCAGCACCCTTGTTCTTTACATTATTGTGGCTATGGTAACCACCAAGCTAACAAATAAGTTCATTCTGGACTCCCAAGAAATTGAAATCATCTGAACCTTGCTACCAGCAATTATCCTAATTCTGATCGCCCTACCCTCCCTTCGCATTCTTTACCTCATGGATGAAATCAATGACCCCCACCTCACAATTAAAGCCATAGGACATCAATGATACTGAAGCTACGAATATACGGATTATGAAGACCTAGGGTTCGACTCGTATATGGTCCCTACACAAGACCTCGCCCCTGGTCAATTTCGACTACTTGAAACAGACCATCGCATGGTCATTCCTGTTGAGTCCCCCATCCGGGTTCTTGTCTCCGCCGAGGATGTTTTACACTCATGGGCCGTCCCAAGCCTTGGAGTAAAAATGGACGCCGTCCCCGGCCGCCTAAATCAAACAGCCTTCATTACTTCCCGTCCGGGTGTGTTTTATGGACAATGCTCAGAAATTTGCGGAGCTAATCATAGCTTTATACCCATTGTAGTGGAAGCTGTTCCTCTAGAACACTTCGAGAACTGATCTTACCTAATACTTCAAGATGCCTCACCAGGAAGCTAAAAGGGGATAGCATTAGCCTTTTAAGCTAAAAATTGGTGACTCCCGCCCACCCCTGGTGACATGCCTCAGTTGAACCCCGCACCCTGATTTGCTATTATAGTATTCTCATGACTAGTTTTCCTAGCCGTTATCCCACCTAAAGTTTTAGCTCACCATTTTCCCAATGACCCCGCCCCACAGAGCGTAAAAAAATCAAAAACAGAGACCTGATCCTGACCATGACTTTAAGCCTCTTTGATCAATTTATGAGCCCTACACTTCTAGGGGTGCCTCTTATCGGACTCGCCCTAACATTGCCATGAGTCCTTTACTTCCAACCCGGTGCCCGATGACTTAATAACCGCTTCATTACCCTTCAATCTATATTCATGAACTGATTTGTAAAACAAATCTTTCAGCCAATAAGCTTAGGCGGACACAAATGGGCCGCTCTCCTCATATCTTTAATACTATTTTTAATTACCTTAAATATGCTAGGCCTACTGCCTTATACATTTACTCCAACAACGCAGCTGTCACTTAATATAGCCTTTGCAGTACCACTTTGACTAGCAACTGTCATTATTGGAATACGAAATCAGCCAACACATGCCCTTGGTCACCTCCTCCCCGAAGGAACTCCTACCGCCCTAATCCCGGTTTTAATCGTGATTGAGACAATTAGCCTTTTTATTCGACCATTGGCCCTCGGTGTTCGACTTACCGCAAACTTGACAGCCGGGCACCTTCTAATTCAACTAATTGCAACTGCGGCTTTTGTTCTTTTCCCTATAATGCCCACAGTCGCCGCTCTTACCTCTGTCTTACTATTCTTGCTAACCCTACTAGAAGTCGCCGTGGCCATAATCCAAGCCTATGTATTCGTACTTCTTTTAAGCCTTTACCTACAAGAAAACGTCTAATGGCCCATCAAGCACATGCATATCATATAGTTGACCCAAGCCCTTGACCCCTCACAGGCGCAGTAGCCGCCCTTCTACTTACATCTGGAACAGCAATCTGAATACACTTTAACTCCACGGTTCTCATGTCCCTTGGACTTGTTCTGTTACTACTAACCATATATCAATGATGGCGAGACATTATCCGAGAGGGCACCTTTCAAGGTCATCATACACCCCCTGTTCAAAAAGGCCTTCGGTACGGGATAATTCTGTTTATTACCTCAGAGGTCTTCTTTTTCCTAGGTTTCTTCTGAGCATTTTATCACTCAAGCCTAGCCCCAACCCCCGAACTTGGTGGATGTTGACCACCCATGGGTATTACAACACTGGACCCCTTTGAAGTTCCCCTTCTCAATACTGCTGTCCTTCTCGCCTCCGGTGTCACGGTCACTTGAGCTCACCATAGTATTATGGAGGGGCAGCGAAAACAAGCAATTCAGTCCTTGACACTCACAATTCTCCTGGGGTTTTACTTTACATTCCTTCAAGCAATAGAGTACTACGAGGCACCCTTCACCATTGCAGATGGCGTCTATGGCTCTACATTTTTTGTGGCAACAGGGTTTCATGGCCTCCATGTAATTATTGGGTCAACATTTCTGGCAGTCTGCCTCTTACGACAAGTCCAGTTCCATTTTACATCAGAACATCACTTCGGATTCGAAGCTGCAGCATGATACTGACACTTTGTAGACGTAGTCTGACTATTCTTATATATCTCTATCTACTGATGAGGCTCATATCTTTCTAGTATTAAAAAGTACAAGTGACTTCCAATCACTCAGTCTTGGTTAGACTCCAAGGAAAGATAATGAACTTAGTACTAGTCATTATTTGCATCTCATTAGCCCTCGCCGCACTGCTCGCAACTGTTTCATTTTTCCTCCCACAAATAACCCCGGATTATGAGAAACTCTCACCGTATGAGTGCGGCTTTGATCCAGTGGGATCCGCCCGTTTGCCATTCTCCATTCGCTTTTTTCTAGTCGCAATCCTATTTCTCCTCTTCGACTTAGAAATTGCCTTACTTCTTCCCCTTCCCTGAGGGGACCAACTTCCCTCCCCTCTAACAACTTTCTTTTGAGCTTCTGCTATTCTTATACTACTAACTCTAGGGTTAATCTATGAATGACTTCAAGGGGGCCTAGAGTGGGCAGAATAGGTACTTAGTTTAATAAAAACATTTGATTTCGGCTCAAAAACTTATGGTTTAAGTCCATATTTACCTGATGACCTTAACTCACTATGCATTCTCGTCAGCCTACTTTGTTAGCTTCATGGGTTTAATTTTTTACCGAAAGCATCTTCTCTCCGCCTTACTTTGCTTAGAAGCGATAATACTTATTCTTTTTATTTCACTATGCCTGTGAGGTCTAGTCTTAGCCTCAAGTGCATTTTCGGCAGGCCCAATGATCTTACTTGCTTTCTCAGCATGTGAAGCAAGTGCAGGCCTAGCACTGCTTGTAGCAATAGCTCGAACCCACGGTACTGACCGTTTAAAAAACCTTAGCCTACTCCAATGTTAATAATTCTTATTCCTACTGTTATGCTTCTACCCACAATCTGACTGACCCCCACTAAATACCTGTGATCCTCAACACTCGGCCATAGCATAATGATTGCTCTTATAAGCCTCTCCTGACTTAGCCTCCCAGGGGAGGTTGGCTGATCTTCCCTTAACACTTTTATAGCAACAGACCCTCTCTCTACCCCCCTTCTCGTACTTACTTGCTGACTTCTGCCCTTAATAATTCTTGCGAGCCAAAACCATATAGCCCAAGAACCTACCAATCGCCAGCGAACCTATATCTCTCTCCTTACTTCCCTTCAAATCTTCTTAATCTTAGCATTTGGGGCAACCGAGATAATCATGTTCTACATTATATTTGAAGCGACCTTAATTCCCACACTCGTAATTATCACACGATGAGGAAACCAAACAGAGCGATTAAACGCAGGTATTTACTTTTTATTTTATACCTTAGCCGGCTCTTTACCACTACTAGTGGCCCTCCTTCTACTTCAGACCTCGACAGGAACTCTTTCTTTTCTAACCACTCAATTTTTTCCCCCTTTACAACTGCATACAGAAGCAAGTAAATTCTGGTGGGCAGGCTGTTTACTAGCATTCTTAGTAAAAATGCCCCTATATGGGGCACACCTTTGACTTCCAAAAGCTCACGTCGAAGCCCCCATCGCCGGGTCAATAGTCCTTGCAGCCGTTCTTTTAAAACTAGGGGGTTACGGTATGATACGAGTTACTATTATCTTAGAGCCCCTCACGAAACAACTCAGCTACCCCTTTATTGTTCTTGCCCTGTGGGGCGTCGTAATAACTGGCTCAATTTGCCTCCGACAAACAGACCTTAAATCACTAATCGCTTACTCCTCAGTAAGCCACATAGGCCTTGTCGCAGCAGGCATCCTAATCCAAACTCCTTGGGGGTTTACAGGAGCATTAATCCTTATAATTGCCCATGGCTTAACTTCCTCCGCCCTATTCTGTTTAGCCAACACTAACTATGAGCGAACACATAGCCGAACCATGCTTTTAGCCCGGGGTCTACAAATGGTCCTTCCTCTCTTAGCAACTTGATGGTTTCTATTTACCCTCGCCAACCTAGCACTCCCTCCACTACCCAACCTCATAGGAGAACTTATGATTATCTCATCCTTGTATAACTGGTCAAACTGGTCTCTAATCCTGACCGGGGCGGGAGTACTAATTACCGCTAGTTACTCTCTCCATATATTCCTAACCACTCAACGTGGCCCTATCACTAACCCCGTCTTGGCAATTGAACCAACCCATACACGAGAACATCTCCTCATAATCCTTCACCTTCTTCCCCTCCTCCTTCTAATTTTAAAACCCTGCTTGATCTGGGGCTGAACAGCTTGTAGGCGTAGTTTAAATAAAGCGCTAGATTGTGATTCTAGAAATAAGAGTTAAACCCTCTTCACCCACCGAGAGGGGTCGCCGTGACAGCAAGAACTGCTAATTCTAGCCCCTTTGGTTAAAGTCCGAAGCCCACTCGAACAGGCTTCTAAAGGATAACAGCTCATCCGTTGGTCTTAGGAACCAAAAACTCTTGGTGCAACTCCAAGTAGCAGCTATGCACTTTACAACAATGATTCTCTCCTCAAGCCTAATAACAATTTTCCTTCTTCTTATCCTTCCAGTCCTAGGTACACTAAATCCTAACCCCACAGGGGACCTGTGGGCCACAAAAAACGTTAAAACAGCAGTTAAGATGGCCTTTTTTGTAAGCCTTCTACCTCTTTTTATCTTTCTTAATGAAGGAGTAGAGACTATTATAACAAACTGAAAATGGATAAATACTCTAATATTTGAAATTAATATCAGCTTTAAATTTGACCTCTACTCTGTGGTATTTACCCCTGTAGCCCTCTACGTAACATGATCAATTTTAGAGTTCGCATCTTGGTACATACACAGTGACCCCAATATAAACCGATTCTTTAAGTATCTTCTAATCTTTCTAATCGCTATGGTTGTTCTGGTTACAGCCAACAACATGTTCCAACTATTTATTGGCTGAGAAGGTGTTGGAATTATGTCTTTCTTACTTATTGGCTGGTGGTTCGGGCGGGCTGACGCCAACACTGCGGCCCTCCAAGCCGTAGTTTATAACCGAGTCGGTGATATCGGCCTAATTCTAGCAATAGCATGAATAGTAGTAAACCTAAACTCATGAGAGATACAACAGCTCTTTTCTGTGTCTAAAGGCCATGACATGACCCTTCCCTTATTAGGCCTAGTACTGGCCGCTACCGGAAAGTCCGCCCAGTTTGGGCTTCACCCCTGGCTCCCCTCAGCCATAGAAGGTCCAACACCGGTCTCTGCCCTCCTGCACTCTAGCACCATGGTTGTTGCTGGTATTTTCCTTCTTATCCGCCTCAGCCCCTTAATGCAAGAAAGCCCGTTAATTCTCTCAACATGCCTTTGCCTGGGGGCCCTAACTACCGTCTTTACTGCGACATGTGCCCTTACCCAAAATGACATCAAAAAAATTGTTGCATTTTCTACATCAAGTCAATTAGGACTAATAATAGTTACCATCGGACTAGGCCAGCCCCAGCTCGCCTTTCTTCATATCTGCACCCACGCCTTCTTTAAAGCAATACTTTTCTTATGTTCAGGCTCCATCATTCATAGCCTTAATGATGAACAAGATATCCGAAAAATAGGAGGACTTCACAAGCTCCTTCCACTGACCTCTTCTTGTCTAACCATTGGCAGCCTAGCTCTAACAGGAGTCCCCTTTTTAGCAGGCTTCTTTTCCAAAGACGCCATCATTGAAGCTATAAATACATCCTACCTTAACGCCTGGGCCCTAATTTTAACGCTTCTAGCTACATCATTTACCGCAGTTTACAGTCTCCGAGTCGTATTCTTTGCCTCTATGGGCCACCCGCGTTTTAATCCAATCTCCCCAATTAATGAAAATAACCCTACAGTAATAAACCCTATTAAACGACTCGCTTGAGGAAGCATTTTGGCAGGGTTGCTAATTACGGCCAATATTGTTCCACTTAAAACCCCCGTTTTAACCATGCCTTTCACCTTAAAAATGGCCGCACTGGCTGTAACAATTATAGGACTACTCACAGCCTTAGAACTAGCATCTCTCACGTCCCAACAATTTAAAATCAAACCCTTATCTTCTACTCACCACTTCTCAAATATATTAGGATTTTTCCCGAGTGTAGCCCATCGACTAGTCCCAAAAACTGGCCTGATTCTTGGGCAACTGGTTGCCAATCAGACAATTGACCAAACCTGACTAGAGAAAACCGGGCCAAAAATAGTAGCCTCCGTTAACCTTCCAGTGGCTACTTCAATTAGCAACCTACAGCAGGGTGTAATTAAGACCTACTTCTTATTATTTTTCTTCACCATAATACTAGCAATTCTCATCCTTGTCATCTAACTGCCCGTAAGGTCCCCCGACTTAGCCCTCGAGTTAACTCCAGAACTACAAAAAGCGTCAGTAATAAAACTCATCCCCCAAGCATTAAAACTCCTCCTCCTGAAGAATATATCAGAGCAACCCCCCCAAGATCCCCCCGGAATAGCATGAATTCACTAAACTCGTCAGCAGTTATCCATGACCCCTCATACCAACCCTCAGAGAAAAAGACAGAGATAGACGCGACCAGAAACACATATACTGACATAAGAAGCAAAACGGGTCAACTTCCCCACCCCTCAGGATAGGGCTCCGAAGCCAGCGCTGCTGAATACGCAAACACAACTAACATCCCGCCTAAATAGATCAAAAACAAAATCAGAGATAAAAATGAACCCCCGTGCCCTACTAAAATGCCACAGCCCATTCCTGCTACTGTGACAAGGCCCAAAGCAGCAAAGTAGGGTGAGGGGTTTGAGGCCACGGCCGCTAGGCCTAAAACCAGGCCAACTAATAATAAATAAGTTATATAAACCATAATTCTTGCCAGGATTTTAACCAGGGCCTGCGACTTGAAAAACCACCGTTGTACTCAACTACAAGAACCTAATGGCCAATCTTCGAAAAACCCATCCCCTATTAAAAATCGCAAACGATGCCCTGGTTGATCTCCCAGCCCCGTCAAACATTTCAGTTTGATGAAACTTCGGGTCTCTTCTAGGACTTTGTTTGGCCGCCCAGATTATTACGGGCCTTTTCCTTGCAATACATTATACATCAGACATTGCCACAGCATTTTCATCTGTAGCACATATTTGTCGTGACGTCAACTACGGCTGACTAATCCGAAACATGCATGCAAACGGTGCTTCCTTTTTCTTCATTTGCATCTACCTGCACATCGGACGGGGCTTGTATTATGGATCATACTTATATAAAGAGACATGAAATGTGGGTGTTGTCCTTCTCCTCCTAGTGATAATGACTGCTTTCGTAGGCTACGTCCTACCCTGAGGACAAATGTCATTCTGAGGGGCTACCGTCATTACCAACCTTTTATCAGCCATTCCCTACGTTGGAAACGCCCTAGTTCAATGAATCTGAGGCGGGTTTTCAGTAGACAACGCCACCCTTACCCGGTTCTTTGCCTTCCATTTCCTCCTTCCCTTTGTGATTGCTGCTGCTACAGTTGTACATCTTATCTTCCTCCACGAGACAGGATCGAATAATCCAACAGGTTTAAACTCAGACTCTGACAAAGTATCTTTTCACCCCTACTTTTCTTATAAAGATCTTCTAGGCTTTGCTGCCCTACTAGTAGCCCTTATCTCTTTAGCCCTCTTCTCCCCAAATCTACTGGGAGACCCCGATAACTTTACCCCTGCTAATCCTTTAGTGACGCCACCTCACATCAAACCTGAGTGATACTTCTTGTTCGCTTACGCCATTCTACGATCCATCCCAAACAAACTTGGCGGGGTTCTAGCCCTATTAGCCTCTATTCTAGTCCTCTTTCTTGTTCCCATTCTGCACACATCAAAACAGCGAAGCCTAACATTCCGACCCCTAACCCAATTCCTCTTCTGATTGCTAGTCGCCGATGTAATAATTTTAACCTGAATTGGAGGTATGCCTGTAGAACATCCTTACATTATCATTGGACAAATCGCATCCTTCATTTATTTTTCCCTTTTCCTAGTCATAGCGCCTATGGCCGGCCTACTAGAAAACAAAGTCTTAAAATGACAATGCATTAGAAGCTCAGATGAAAGAGCACCGGTCTTGTAAACCAGAGGTCGAAGGTTCAAGCCCTTCCTAGTGCTCAGAGAGAAGGGATTCTAACCCCTGCCCCTGACTCCCAAAGCCAGGATTCTTAACTAAACTACTCCCTGATTTTCATACACCAGTTTTGTAATCCAGAGCGCATCACTTTTGCCACCAACGTTAAATTAACGTTGCACAAACGTTGCATCAGCGCCCCATGGACACTAAATGACGCGAGGGCGCTAAATAAACACCCCCTACCTCTAGCACCCTTTTAACGATTTCACTTTTTTTTTTTTTTTTGTTTAACGATTACGTTTTTTTTTGCGTTCCCGGACTCTGCCAGATTTCGACCGAAGACTGCCAGAATCCGCTCAAAATCCGCTCAAATACCAATATGTATTATCCCCATAAATGGTTTAAACCATTTTTGCCTAGTACACACTGACCATGCAAGTCAATTATATTTACCCCGCGCTCCAGGCCGCAGTACATACACCTACAGTTGGTGTATTTAGCACAAGTGTGCCTCAGCTAGTTTCAAGTCACCCACATCCTTCCTTTAATTGTTACTTAATGTAGTAAGAGCCCACCATCAGTTGATTCCTTAATGTCAACGGTTCTTGAAGGTGAGGGACAAAAATCGTGGGGGTTTCACTTCTTGAATTATTCCTGGCATTTGGCTCTACATCTCAAGGCCATACATTTCTCGTCTCTCACACTTTCACTGGCCCTGACATTGGTTAATGGTGGAGTACATACTCCTCGTTACCCCCCATGCCGGGCGTTCTTTCTAATGGACAACGGGTTTTCCTTTTTTTTTCCTTTTCACTTGGCATTTCACAGTGCATACAAACCTTGATGACAAGGTTGAACATTTAGAAATCGGCCGCAAAGAATATTGGTGAATTATTCAAAGATATTAACAGATGAATTGCATAAGTGATATCAAGAGCATAAATAACCAAATGAAACTAGGAACGTTTCTATAATATGCCCCCCGGCTCCCGCGCGTCAAACCCCCCTACCCCCCTAAACTAGTAAGAAGTCTATTATTCCTGCAAACCCCCCGGAAACAGGAAACCCCCTACTAGCATTTTAGCCCGCCCAAATTTGTGTGTATTTATATTATTTGTAATATTGCAAAA

>JWM 10

GCTAGTGTAGCTTAACTAAAGCATAACACTGAAGATGTTAAGACAAACCTTAGATTGGTTTCACGAGCACAAAAGTTTGGTCCTGACTTTACTATCAACTTTAGCTAAACTTACACATGCAAGTATCCGCAATCCCGTGAGAATGCCCTACAGTTTCCTTAAAGGAAACAAGGAGCTGGTATCAGGCTCAATTACTCCCGCCCATGACACCTTGCTTAGCCACACCCCCAAGGGAACTCAGCAGTGATAGACATTAAGCAATAAGTGAAAACTTGACTTAATTAAAGCTAAGAGAACCGGTTAAACTCGTGCCAGCCACCGCGGTTATACGAGCGGTTCGAGCTGATAGACTACGGCGTAAAGCGTGGTTAATAAGAATAAAACTAAAGTCGAATGTTTTCAAAGCTGTTATACGCACTCGAAAATTAGAAGACCAGAAACGAAAGTGACTTTAACCCTATGAACCCACGAAAACTATGAAACAAACTGGGATTAGATACCCCACTATGCATAGCTGTAAACTTTGATGAGCCATTACATTATCATCCGCCTGGGTACTACGAGCATCAGCTTAAAACCCAAAGGACTTGGCGGTGCTTTAGACCCACCTAGAGGAGCCTGTTCTAGAACCGATAACCCCCGTTAAACCTCACCCTCTCTTGTTTTTCCCGCCTATATACCGCCGTCGTCAGCTTACCCTGTGAAGGTCTAATAGTAAGCACAACCAGTTATACTCAAAACGTCAGGTCGAGGTGTAGCATATGAGAGGGGAAGAAATGGGCTACATTCCTTGTTTCAAGGAAAACGGATAACATAATGAAAGGTACGTTAGAAGGAGGATTTAGCAGTAAGCAGCAAATAGAGTGTTCTGCTGAAACTGGCCCTGAAGCGCGCACACACCGCCCGTCACTCTCCCCAACTCCGAGTTAAAAACCATATATAAACCTTTGAAGGAACAAAGGGGAGGCAAGTCGTAACATGGTAAGTGTACCGGAAGGTGCACTTGGATAAATCAGAGTATAGCTAAGAAAGAAAAGCATCTCCCTTACACCGAGAAGTCATCCGTGCAAATCGGATTACCCTGACTCTAACAAGCTAGCCCAAAACCTTAACTTAAAAATCAAATATTTCTAGTAATTAATAAACCAAACACATTAAATAAATCATTTTTCCCCCTGAGTATGGGAGACAGAAAAGGATAAAGGAGCTATAGACAAAGTACCGCAAGGGAAAGCTGAAAGAGAAATGAAACAAACCAGTAAAGAAGAACAAAGCAGAGATTAACCCTTGTACCTTTTGCATCATGAATTAGCCAGTTTAATCAAGCAAAGAGCACTGTAGTTTGAACCCCCGAAACTTAGTGAGCTACTTCAAGACAGCCTATGAAATAGGGCAAACCCGTCTCTGTGGCAAAAGAGTGGGAAGATCTTCAAGTAGGGGTGACAGACCTATCGAACTAAGTTATAGCTGGTTGCTCGTGAAATGAATAGAAGTTCAGCCTTTTGCTTTCTAAATTTCGATTTAGCACTACTTAGCCTAAATGACTAGAAAACAAAAGAGTTAGTCAAAGAGGGTACAGCCTGTTTGATAAAAGATACAACTTTACTAGGAGGATAAGAATCATAATTTTAAAGGTTTAATGCCCAGGTGGGCCTAAAAGCAGCCACCCTAATCAATAGCGTTAAAGCTTAAGCATAAAACACACCTACAATTCTGATAAATCAGTTTTAATCCCCTAAAGTTAACGAGCTATTTCATACCTTATGAAAGAAATTATGCTAGTATGAGTAATAAGAAGTTACGAACTTCTCCCTGCACACGTGTAAATCGGAACGGACAAACCACCGAATCTTAACGGCCCCAGTCAAAGAGGGGATGTCGGATAAAAAAAAGAACAAGAAACTCCCGACAAAACCACCGTTAACCCCACACCGGAGTGCTCCCTGGGAAAGACAAAAAGGGACAGAAGGAACTCGGCAAATATGCTCAAGCCTCGCCTGTTTACCAAAAACATCGCCTCTTGTAAAAGTTAAATAAGAGGTACCGCCTGCCCTGTGACTAGTAGTTTAACGGCCGCGGTATTTTGACCGTGCAAAGGTAGCGCAATCACTTGCCTTTTAAATGAAGGCCTGTATGAATGGCACGACGAGGGCTTAACTGTCTCCTCTCCCTAGTCAATGAAATTGATCTCCCCGTGCAGAAGCGGGGATAATAACATAAGACGAGAAGACCCTGTGGAGCTTTAGACCATGAGCAGACCATGTCAAGAATAACAAACAAGTAAATTAAACAGATTGGTCCCTGCTTCTCTGTCTTTGGTTGGGGCGACCGCGGGATAATAAAAAGCTCCCACGAGGATTGAGAACCCTTATCTTATAACCAAGAGCTTCTCCTCTAAGTAACAGAACATCTGACCTTAATGATCCGGCCTGGCCGATCAACGGACCGAGTTACCCCAGGGATAACAGCGCAATCCTCTTTTAGAGTCCATATCGACAAGAGGGTTTACGACCTCGATGTTGGATCAGGACATCCTAATGGTGCAGCCGCTATTAAGGGTTTGTTTGTTCAACAATTAAAGTCCTACGTGATCTGAGTTCAGACCGGAGTAATCCAGGTCAGTTTCTATCTATGACGTACTCTCTTCTAGTACGAAAGGACCGAAGAAAGAAGGCCTATGAAAAGTTATGCCTTAATCTCACCTTATGAAGAAAACTAAATAAGACAAGAGGTTACACCCCTTAGTCATAGAAAATGACATGTTAAGGTGGCAGAGCCCGGATATTGCAAAAGACCTAAGCCCTTTCCACAGAGGTTCAATTCCTCTCCTTAACTATGTTCTCAACAATATTAAGCTTCATTATTAATCCCCTAATTGTTATGGTTTTTGTTTTGCTGGCAGTAGCCCTCTTAACCTTGGTAGAGCGTAAAGTGCTAAGCTACATGCAACTTCGTAAAGGCCCAAATGTTGTTGGCCCTTACGGCCTTTTGCAACCCTTCGCTGATGGCTTGAAACTTTTCATGAAAGAGCCCGTCCGACCCTCCACCTCCTCGCCCGCCTTATTCTTAATTACCCCTATTATAGCCCTTACCTTAGCCCTAACCCTCTGGGCCCCCCTTCCTATGCCTTTTCCCATCACCGACCTAAACTTAGGCATTTTATTTATTTTAGCACTATCGAGCCTGGCAGTATATTCTATTCTTGGCTCCGGATGGGCCTCCAATTCTAAATATGCATTGATTGGTGCTCTTCGAGCAGTCGCCCAAACCATCTCTTATGAAGTGAGCTTGGGCCTTATTCTTCTTAACACAATTGTCTTTACAGGGGGTTTTACTCTTCAAACCTTCAGCACCGCACAAGAAGCCACCTGATTACTTCTACCAGCATGACCACTAGCAGCCATGTGATATATCTCCACACTCGCGGAAACTAACCGGGCCCCTTTCGACTTAACTGAAGGAGAGTCCGAACTAGTGTCTGGCTTCAACGTAGAGTATGCCGGCGGACCTTTTGCCCTTTTTTTTCTGGCAGAATACGGTAACATTTTACTTATAAATACCCTCTCAGCAGTACTATTTCTAGGCTCTTCAACCTACCACAGCTTTCCAGAACTAACCGCGACCTTATTAATGCTTAAAGCCACCCTCCTTTCAGTCGTATTTTTATGAGTGCGAGCATCTTACCCTCGGTTTCGATACGATCAACTAATGCATTTAATTTGAAAAAACTTTTTACCTCTGACCCTAGCACTAGTTATTTGACACCTTTCTCTTCCGATCACGTTGAGCGGCCTTCCCCCTCAACTTTAACTCAGGAAATGTGCCTGAAAAAGGGTCACTTTGATAGGGTGAATAATGAGGGTTAAAGCCCCTCCATCTCCTTAGAAAGAAGGGGTTTGAACCCTACCTGAAGAGATCAAAACTCTTAGTGCTTCCACTACACCACTTCCTAGTAAAGTCAGCTAATAAAAGCTTTTGGGCCCATACCCCAAATATGTTGGTTAAAATCCTTCCTTTGCTAATGAATCCTTACGTCCTTTCAATTCTACTTATAGGTTTAGGCCTCGGCACTACAGTCACATTCGCTAGCTCACACTGACTATTAGCATGAATAGGCCTTGAAATAAATACCCTCGCCATTTTGCCGTTAATAGCACAACATCACCACCCCCGAGCCGTTGAAGCCACCACCAAGTATTTTTTAATTCAATCGGCAGCCGCAGCAACCATCTTATTTGCCAGCTCAACAAACGCCTGACTTTCGGGCCAGTGGGACATCATAAGTATTAATCACCCTCTTCCAACCGTCATAATTACAGTCGCTCTGTCCTTAAAACTAGGCTTGGCCCCTCTTCACGCGTGACTTCCCGAAGTAATTCAAGGCCTGGACTTAACTACGGGCTTAATCCTCTCCACATGACAAAAACTCGCACCCTTTACCCTCCTCGTTCAAATCTTCCCCGACACCCCCCTTCTCATCACTTCTCTAGGACTTCTTTCAATATTAGTTGGGGGATGAGGGGGTTTAAACCACACACAGCTCCGCAAAGTGCTCGCATATTCTTCGATCGCCCACTTAGGCTGAATAATAGTAATTATGCAATTCTCCACCCCCCTTACAATTCTTGCTTTATCAACATACATTGTCATAACATCATCTACTTTTCTAATCTTTAAACTCCTTAAATCCACAGATATGAACAGCCTGGCAACATCTTGAGCTAAAACCCCCTCCATTACAGCCCTAGCACCTTTAGTGCTATTATCCTTAGGCGGACTCCCTCCCCTCTCGGGCTTTATGCCAAAATGACTAATTATTCAGGAGTTAACTAAGCAAGACCTAGCCCTAGTTGCGACCTTAGCCGCCCTCTCTGCGCTACTCAGCCTTTTCTTTTACCTACGCATTTGTTACTCCCTCACATTTACCTCCTCTCCTAATAATCTCATGGGAACACCCCCCTGACGACTAGTAACAAAGCAAGTATCACTTCCCCTGGCTATAACAACCGCCCTCTCTATTCTCCTACTCCCGGTTACCCCTGCAATCTTATCAGTAGTTCTCCCCTTGTAAAGAGGCTTAGGATAGTATTAAGACCAAGGGCCTTCAAAGCCCTAAGCGGGAGTGAAAGCCCCCCAGCCTCTGTAAGACCTACGGGACACTAACCCACATCTTCTGTATGCAAAACAGACACTTTAATTAAGCTAAAGCCTTCCTAGGTGGGTAGGCCTCGATCCTACAATCTCTTAGTTAACAGCTAAGCGCCTAAACCAGCGGGCATCCATCTACCTTTCCCCCGCCTTGCCGAGAAAAAAAGGCGGGGGAAAGCCCCGGCAGGGTATTAGCCTGCTACTTAAGATTTGCAATCTAATGTGTTAACACCTCGGAGCTGGTAAGAAGAGGACTTTAACCTCTGTCTATGGGGCTACAATCCACCGCTAAACGCTCAGCCACCTTACCTGTGGCAATCACACGTTGATTTTTCTCAACTAATCACAAAGACATCGGCACCCTATATCTAATCTTTGGTGCCTGGGCGGGAATAGTAGGGACGGCCTTAAGTCTACTCATTCGGGCAGAATTAAGTCAACCAGGCTCCCTATTAGGAGACGACCAGATCTATAACGTAATTGTAACTGCACATGCTTTCGTAATAATTTTCTTTATAGTAATGCCAATTATAATTGGAGGGTTCGGCAACTGATTAATTCCTTTAATGATCGGAGCTCCCGACATGGCCTTCCCCCGGATAAATAATATAAGCTTTTGACTCCTGCCCCCTTCTTTCCTTCTATTATTGGCCTCATCTGGTGTAGAAGCTGGTGCCGGGACAGGATGAACCGTATATCCCCCCTTGTCCGGTAATTTGGCACACGCAGGGGCCTCCGTAGATTTAACCATTTTCTCTCTTCACCTGGCCGGAATCTCTTCTATTCTAGGGGCCATTAATTTCATTACAACTATTATTAATATAAAACCTCCAGCCATCTCCCAATATCAAACCCCTTTATTTGTCTGAGCTGTTCTAATTACCGCAGTATTACTCCTACTCTCTCTTCCTGTTCTAGCTGCAGGTATCACTATGCTTCTCACAGATCGAAACCTAAATACAACATTTTTCGACCCCGCAGGAGGGGGGGACCCCATTCTTTATCAACACTTATTCTGATTCTTTGGGCATCCTGAAGTCTACATTCTGATTTTGCCCGGCTTCGGAATGATTTCTCACATTGTAGCATATTACTCAGGCAAAAAAGAGCCGTTTGGCTACATGGGAATAGTATGAGCTATAATAGCAATTGGCTTACTGGGGTTTATCGTATGAGCCCATCATATGTTCACTGTAGGGATGGACGTGGACACTCGAGCTTATTTTACATCCGCCACTATAATTATCGCAATTCCTACAGGAGTCAAAGTGTTTAGTTGACTAGCTACCTTGCATGGGGGCTCAATCAAATGAGAGACCCCTCTGTTATGAGCTCTAGGCTTTATTTTCTTATTTACTGTCGGAGGTTTAACAGGAATTGTTTTAGCCAACTCATCTCTGGACATTATACTTCATGACACATACTATGTTGTAGCCCACTTCCACTATGTCCTCTCTATAGGAGCAGTCTTTGCCATCATGGGGGCATTCGTTCACTGATTCCCCCTATTCTCAGGCTACACCCTTCACAATACGTGAACAAAAATCCACTTCGGAGTTATGTTTGTAGGTGTAAACCTCACCTTTTTCCCTCAGCACTTCTTAGGATTGGCGGGAATACCTCGACGATATTCAGATTACCCTGACGCATACACACTGTGAAATACTATCTCATCCCTGGGGTCACTAATCTCCCTTATTGCTGTAATTATATTCCTATTTATTATCTGGGAAGCATTCGCGGCAAAACGTGAAGTCTTATCAGTTGAACTAACAGCCACAAACGTAGAATGACTGCACGGGTGTCCTCCCCCTTACCATACATTTGAAGAACCTGCATTCGTTCAAATTCAGCAATCCAAATTTTAATCGAGAAAGGAAGGAGTCGAACCCCCATAAACTGGTTTCAAGCCAGCCACATAACCGCTCTGTCACTTTCTTCTCTAAGTTAATAAGATTCTAGTTAAAAGAATAACGCTGCCTTGTCAAGGCAAAATTGTGGGTTAAAGCCCCACGTATCTTGCTTATGGCACATCCATCTCAACTAGGATTCCAAGATGCAGCTTCACCCGTTATAGAAGAACTTCTCCATTTTCATGACCATGCATTAATAATTGTTTTCTTAATCAGCACCCTTGTTCTTTACATTATTGTGGCTATGGTAACCACCAAGCTAACAAATAAGTTCATTCTGGACTCCCAAGAAATTGAAATCATCTGAACCTTACTACCAGCAATTATCCTAATTCTGATCGCCCTACCCTCCCTTCGCATTCTCTACCTCATGGATGAAATCAATGACCCCCACCTCACAATTAAAGCCATGGGACATCAATGATACTGAAGCTACGAATATACGGATTATGAAGACCTGGGGTTCGACTCATATATGGTCCCTACACAAGATCTCGCCCCTGGTCAATTTCGACTACTTGAGACAGACCATCGCATGGTTATTCCTGTTGAGTCCCCCATCCGAGTTCTTGTCTCCGCCGAGGATGTCTTACATTCATGAGCCGTCCCGAGCCTCGGAGTAAAAATGGACGCCGTCCCCGGCCGCCTAAATCAAACAGCCTTCATTACTTCCCGACCAGGTGTGTTTTATGGACAATGCTCAGAGATTTGCGGAGCTAATCATAGCTTTATACCCATTGTAGTGGAAGCTGTTCCTCTAGAACACTTCGAGAACTGGTCTTACCTAATACTTCAAGATGCCTCACCAGGAAGCTAAAAGGGAATAGCATTAGCCTTTTAAGCTAAAAATTGGTGACTCCCGCCCACCCCTGGTGACATGCCTCAGTTGAACCCCGCACCCTGATTTGCTATTATAGTATTCTCGTGACTAGTTTTCCTAGCCGTTATTCCACCTAAAGTTCTAGCTCACCATTTTCCCAATGACCCCGCCCCACAGAGCGTAAAAAAATCAAAAACAGAGACTTGACCCTGACCATGACTTTAAGCCTCTTTGATCAATTTATGAGCCCTACACTTCTAGGGGTGCCTCTTATCGGACTCGCCCTAACATTGCCATGAGTCCTTTACTTCCGACCCGGTGCCCGATGACTTAATAACCGCTTGATTACCCTTCAATCTATATTCATAAACTGGTTTGTAAAACAAATCTTTCAACCAATAAGCTTAGGCGGACACAAATGGGCCGCTCTCCTCATATCTTTAATACTATTTTTAATTACCTTAAATATGCTGGGCCTGCTGCCTTACACATTTACTCCAACAACGCAGCTGTCACTTAATATAGCCTTTGCAGTTCCACTTTGACTAGCAACTGTCATTATTGGAATACGAAACCAGCCAACACATGCCCTTGGTCACCTTCTCCCTGAAGGAACTCCTACCGCCCTAATCCCGGTTTTAATCGTGATTGAAACAATTAGCCTTTTTATTCGACCCTTGGCCCTCGGTGTTCGACTTACCGCAAACTTGACAGCCGGACACCTTCTAATTCAACTAATTGCAACTGCGGCTTTTGTTCTTTTCCCTATAATACCTACAGTAGCTGCTCTTACCTCTGTCTTACTATTCTTGCTAACCCTGCTAGAAGTCGCCGTGGCCATAATCCAAGCCTATGTATTTGTACTTCTTTTAAGCCTTTATCTACAAGAAAACGTCTAATGGCCCATCAAGCACATGCATATCATATAGTTGACCCAAGCCCTTGACCCCTCACAGGCGCAGTAGCCGCCCTTCTACTTACGTCTGGAACAGCAATCTGAATACACTTTAACTCCACAGTTCTCATGTCCCTTGGACTTGTCCTGCTACTACTAACCATATATCAATGATGGCGAGACATTATCCGAGAGGGTACCTTTCAAGGTCATCATACACCCCCTGTTCAAAAGGGCCTTCGGTACGGGATAATTCTATTTATTACCTCAGAGGTCTTCTTTTTCCTAGGTTTCTTCTGAGCATTTTATCACTCAAGCCTAGCCCCAACCCCCGAACTTGGTGGGTGTTGACCACCTATGGGTATTACAACACTGGACCCCTTTGAAGTCCCCCTTCTCAATACTGCTGTCCTTCTCGCCTCCGGTGTCACGGTCACTTGAGCTCACCATAGTATTATGGAGGGGCAGCGAAAACAAGCAATTCAATCCTTAACACTCACAATTCTACTGGGGTTCTACTTTACATTCCTTCAAGCAATAGAGTACTACGAGGCACCCTTCACCATTGCAGATGGCGTCTATGGCTCTACATTTTTTGTGGCAACGGGGTTTCATGGCCTCCATGTAATTATTGGGTCAACATTTCTGGCAGTCTGCCTCTTACGACAAGTCCAATTCCACTTTACATCAGAACATCACTTCGGATTTGAAGCTGCAGCATGATACTGACACTTTGTAGACGTAGTCTGACTATTCTTATATATCTCTATCTACTGATGAGGCTCATATCTTTCTAGTATTAAAAAGTACAAGTGACTTCCAATCACTCAGTCTTGGTTAGACTCCAAGGAAAGATAATGAACTTAGTACTAGTCATTATTTGCATCTCATTAGCCCTCGCCGCACTGCTCGCAACTGTTTCATTTTTTCTCCCACAAATAACCCCTGATTATGAGAAACTCTCACCGTATGAGTGCGGCTTTGATCCAGTGGGATCCGCCCGTTTGCCATTCTCCATTCGCTTTTTTCTAGTCGCAATCCTATTTCTCCTCTTCGACTTAGAAATTGCCTTACTTCTCCCCCTTCCCTGAGGAGACCAACTCCCCTCCCCTCTGACAACTTTCTTCTGAGCTTCTGCTATCCTTATACTACTAACTCTAGGGTTAATCTATGAATGACTTCAAGGGGGCCTAGAGTGGGCAGAATAGGTACTTAGTTTAATAAAAACATTTGATTTCGGCTCAAAAACTTATGGTTTAAGTCCATATTTACCTGATGACCTTAACTCACTATGCATTCTCGTCAGCCTACTTTGTCAGCTTCATGGGTCTAATTTTTTACCGAAAGCATCTTCTCTCCGCCTTACTTTGCTTAGAAGCCATAATACTTATTCTTTTTATTTCACTATGCCTGTGAGGCCTAGTCTTAGCCTCAAGTGCATTTTCGGCAGGCCCAATGATCTTACTTGCTTTCTCAGCATGTGAAGCAAGTGCAGGCCTAGCACTGCTTGTAGCAATAGCTCGAACCCACGGGACTGACCGTTTAAAAAACCTTAGCCTACTCCAATGTTAATAATTCTTATTCCTACTGTTATGCTTCTACCCACAATCTGACTAAGCCCCGCTAAATGCCTGTGGTCCTCAGCACTTGGCCATAGCATAATAATTGCTCTTATAAGCCTCTCCTGACTTAGCCTCCCGGGGGAGGTTGGCTGATCTTCCCTTAACACTTTTATAGCAACAGACCCTCTCTCTACCCCCCTTCTCGTACTTACTTGCTGACTTCTACCCTTAATAATTCTTGCGAGCCAAAACCATATAGCCCAAGAACCTACCAATCGCCAGCGAACCTATATCTCTCTCCTTACTTCCCTTCAAATCTTCTTAATCTTAGCATTTGGAGCAACCGAGATAATTATGTTCTATATTATATTTGAAGCGACCTTAATTCCCACACTCGTAATTATCACACGATGAGGGAACCAAACAGAGCGATTAAACGCAGGTATTTACTTTTTATTTTATACCTTAGCCGGCTCTTTACCACTACTAGTGGCCCTCCTTCTACTTCAGACCTCGACAGGAACTCTTTCTTTTCTAACCACTCAATTTTTTCCCCCCTTACAACTGCATACAGAAGCAAGTAAATTCTGGTGGGCGGGCTGTTTACTAGCATTCTTAGTAAAAATGCCGCTATATGGGGCACACCTTTGACTTCCAAAAGCTCACGTCGAAGCCCCCATCGCCGGGTCAATAGTCCTTGCAGCCGTTCTTTTAAAACTAGGGGGTTACGGTATGATACGAGTCATTATTATCTTAGAACCCCTAACGAAACAACTCAGCTACCCCTTTATTGTTCTTGCCCTGTGGGGCGTTGTAATAACTGGCTCAATCTGCCTCCGACAAACAGACCTTAAATCACTAATCGCTTACTCCTCAGTAAGCCACATGGGCCTTGTCGCAGCAGGCATCCTGATCCAAACTCCTTGGGGGTTTACAGGAGCATTAATCCTTATAATTGCCCATGGCTTAACCTCCTCCGCCCTATTCTGTTTAGCCAACACTAACTATGAGCGAACACATAGCCGAACCATACTTTTAGCCCGGGGTCTACAAATGGTCCTTCCTCTTTTAGCAACTTGGTGGTTTCTACTTACCCTCGCCAACCTAGCACTCCCTCCGCTACCCAACCTCATAGGAGAGCTTATGATTATCTCATCCTTGTATAACTGGTCAAACTGGTCTCTAATCCTGACCGGAGCGGGAGTACTAATTACCGCTAGCTACTCTCTTCATATATTCCTAACCACTCAACGCGGCCCTATTACTAACCCCGTCTTGGCAATTGAACCAACCCACACACGAGAACATCTCCTCATAATTCTTCACCTTCTTCCTCTCCTCCTTCTAATTTTAAAACCCTGCTTGATCTGGGGCTGAACAGTTTGTAGGCGTAGTTTAAATAAAGCGCTAGATTGTGATTCTAGAAATAAGAGTTAAACCCTCTTCACCCACCGAGAGGGGTCGCCGTGACAGCAAGAACTGCTAATTCTAGCCCCTTTGGTTAAAATCCGAAGCCCACTCGAACAGGCTTCTAAAGGATAACAGCTCATCCGTTGGTCTTAGGAACCAAAAACTCTTGGTGCAACTCCAAGTAGCAGCTATGCACTTTACAACAATGATTCTCTCCTCAAGCCTAATAACAATTTTCCTTCTTCTCATCCTTCCAGTCCTAGGTACACTAAACCCTAACCCCACGGGGGGCCTGTGAGCCACAAAAAACGTTAAAACGGCAGTTAAGATAGCCTTTTTTGTAAGTCTTTTGCCTCTTTTTATCTTTCTTAATGAAGGGGTAGAGACTGTTATAACAAACTGAAAATGAATAAATACTCTAATGTTTGAAATTAATATCAGCTTTAAATTTGACCTCTACTCCGTAGTATTTACCCCTGTGGCCCTCTACGTAACATGATCAATTTTAGAGTTCGCATCTTGGTATATACACAGTGATCCCAACATAAACCGATTCTTTAAATATCTTCTAATCTTTCTTATTGCTATGGTCGTTCTGGTCACAGCCAACAACATGTTCCAACTATTTATTGGCTGAGAAGGTGTTGGAATTATATCTTTCTTACTTATTGGCTGGTGATTCGGGCGGGCTGACGCCAACACTGCGGCCCTCCAGGCCGTAGTTTATAACCGAGTTGGTGATATCGGCCTTATTCTAGCAATAGCATGAATAGTAGCAAACCTAAACTCATGAGAAATACAACAGCTCTTTTCTATGTCTAAAGGCCATGATATAACCCTTCCTTTATTAGGCCTAGTACTGGCCGCTACCGGAAAGTCCGCCCAATTTGGACTTCACCCCTGGCTCCCCTCAGCCATAGAGGGTCCAACACCGGTCTCTGCCCTCCTGCACTCTAGCACCATGGTGGTTGCTGGTATTTTTCTTCTTATTCGCCTCAGCCCCTTAATGCAAGAAAGCCCATTAATTCTCTCAACATGCCTTTGCCTGGGGGCCCTAACTACCGTCTTTACTGCAACATGTGCCCTTACCCAAAATGACATTAAAAAAATCGTTGCATTTTCTACATCAAGTCAATTAGGACTAATGATAGTTACCATCGGACTAGGCCAGCCCCAGCTCGCCTTTCTTCATATCTGCACCCACGCCTTCTTTAAAGCAATACTTTTCTTATGTTCCGGCTCCATCATTCATAGCCTTAATGATGAGCAAGATATCCGAAAAATAGGCGGACTTCACGAGGTACTTCCACTGACCTCTTCTTGTCTAACCATTGGCAGCCTAGCTCTAACAGGAGTCCCCTTTTTAGCAGGCTTCTTTTCCAAAGACGCCATCATTGAAGCTATAAATACATCCTACCTTAACGCCTGAGCCCTGATTTTAACGCTTCTAGCTACATCATTCACCGCAGTTTACAGCCTCCGAGTCGTATTCTTTGCCTCTATGGGCCACCCGCGTTTTAATCCAGTCTCCCCAATTAATGAAAATAACCCTACAGTGATAAACCCCATTAAACGACTCGCTTGAGGAAGCATTTTGGCAGGGTTGCTAATTACGACCAATATTGTTCCACTTAAAACCCCCGTTTTAACCATGCCTTTCACCTTAAAAATGGCCGCACTGACTGTAACAATTATAGGACTACTCACAGCCTTAGAACTAGCGTCTCTCACGTCCCAACAATTTAAAATCAAACCCTTATCTTCTACTCACCACTTCTCAAATATATTAGGATTTTTCCCCAGTGTAGTCCATCGACTAGTCCCAAAAACTGGCCTGATTCTAGGACAACTAGTTGCCAATCAGACAGTTGACCAAACCTGACTAGAGAAAACAGGACCAAAAATAGTAACCTCCGTTAACCTTCCAATAGCTACTTCAATTAGCAGCCTACAGCAGGGTGTAATTAAGACCTACTTCTTATTATTTTTCTTCACCATAATACTGGCAATTCTCATCTTTGTCGTCTAACTGCCCGTAAAGTCCCCCGACTTAGCCCCCGAGTTAACTCTAGAACTACAAAAAGCGTCAGTAATAAAACTCATCCCCCAAGCATTAAAACTCCTCCTCCTGAAGAATATATCAGAGCAACCCCACCGAAATCCCCCCGAAAGAGCATGAATTCACTAAACTCGTCAGCAGTTACCCATGACCCCTCATACCAGCCCTCAGAGAAAAAAACAGAGATAGACGCGACCAGGAACACATATACTGACATAAGAAGCAAAACGGGTCAACTTCCCCACCCCTCAGGATAAGGCTCCGAAGCCAGCGCTGCTGAGTACGCAAACACAACTAACATCCCACCTAAATAGATCAAAAACAAAATCAGAGATAAAAATGAACCCCCATGCCCTACTAAAATGCCACAGCCCATTCCTGCTACTGTAACAAGACCTAAAGCAGCAAAGTAAGGTGACGGGTTCGAGGCCACGGCCGCTAGACCTAAGACCAAACCAACTAATAATAAATAAGTCATATAAACCATAATTCTTGCCAGGATTTTAACCAGGGCCTGCGACTTGAAAAACCACCGTTGTACTCAACTACAAGAACCTAATGGCCAATCTTCGAAAAACCCATCCCCTATTAAAAATCGCAAACGATGCCCTCGTTGATCTCCCAGCCCCGTCGAACATTTCAGTTTGATGAAACTTCGGGTCTCTTCTAGGACTTTGTTTGGCCGCCCAAATCGTTACGGGCCTTTTCCTTGCAATACATTATACATCAGACATTGCCACAGCATTTTCATCTGTAGCACATATTTGTCGTGATGTCAACTACGGCTGACTAATCCGGAACATGCATGCAAACGGTGCTTCCTTTTTCTTCATTTGCATCTACCTGCACATCGGACGGGGCTTGTATTATGGATCATACTTATATAAAGAGACATGAAATGTAGGTGTTGTCCTTCTCCTCCTAGTGATAATGACTGCTTTCGTAGGCTACGTCCTACCCTGAGGACAAATGTCATTCTGAGGGGCCACCGTCATTACCAACCTTTTATCAGCCATTCCCTACATTGGAAACGCCCTAGTTCAATGGATCTGAGGCGGATTTTCAGTAGACAACGCCACCCTTACCCGGTTCTTTGCCTTCCATTTCCTCCTCCCCTTTGTAATTGCTGCTGCTACAGTTGTACATCTTATTTTCCTGCACGAGACAGGGTCGAATAACCCAACGGGTTTAAACTCAGACTCTGACAAAGTGTCTTTTCACCCCTACTTTTCTTATAAAGATCTCCTAGGCTTTGCTGCCCTGCTAGTAGCCCTTATCTCTTTAGCCCTCTTCTCCCCTAATCTACTTGGAGACCCTGATAACTTTACCCCTGCTAATCCTTTAGTAACTCCACCTCACATCAAACCTGAGTGATACTTCCTGTTCGCTTACGCCATTCTACGATCCATCCCAAACAAGCTTGGTGGGGTCCTAGCCCTATTAGCCTCTATTCTAGTTCTCTTTCTTGTCCCCATCCTGCACACATCAAAACAACGAAGCCTAACATTCCGACCCCTAACCCAATTCCTCTTCTGATTACTAGTCGCCGATGTAATAATTTTAACCTGAATCGGAGGTATGCCTGTAGAACACCCTTACATTATCATTGGGCAAGTCGCATCCTTCATTTATTTTTCCCTTTTTCTAGTTATGGCGCCTATGGCCGGCCTACTAGAAAACAAAGTCTTAAAATGACAATGCATTAGAAGCTCAGATGAGAGAGCACCGGTCTTGTAAGCCAGAGGTCGAAGGTTCAAGTCCTTCCTAGTGCTCAGAGAGAAGGGATTCTAACCCCTGCCCCTGGCTCCCAAAGCCAGGATTCTTAGCTAAACTACTCGCTGATTTTCATATACCAGTTTTGCAATCCAGAGCGCATCACTTTTGCCACCAGCGTTAAATTAACGTTGCACAAACGTTGCATCAGCACCCCATGGACACTAAATGACGCGAGGGCGTTAAATAGACACCCTCTACCTCTAGCACCCTTTTAACGATTTCACTTTTTTTTTTTTTTTTGTTTAACGATTACGTTTTTTTTTGCGTTCCCGGACTCTGCCAGATTTCGACCGAAGACTGCCAGAATCCGCTCAAAATCCGCTCAAATACCAATATGTATTATCCCCATAAATGGTTTAAACCATTTTTGCCTAGTACACACTGACCATGCAAGTCAATTATATTTACCCCGCGCTCCAGGCCGCAGTACATACACCTACAGTTGGTGTATTTAGCACAAGTGTGCCTCAGCTAGTTTCAAGTCACCCACATCCTTCCTTTAATTGTTACTTAATGTAGTAAGAGCCCACCATCAGTTGATTCCTTAATGTCAACGGTTCTTGAAGGTGAGGGACAAAAATCGTGGGGGTTTCACCTCTTGAATTATTCCTGGCATTTGGCTCTACATCTCAAGGCCATACATTTCTCGTCTCTCACACTTTCACTGGCCCTGACATTGGTTAATGGTGGAGTACATACTCCTCGTTACCCCCCATGCCGGGCGTTCTTTCTAATGGACAACGGGTTTTCCTTTTTTTTTCCTTTTCACTTGGCATTTCACAGTGCATACAAACCTTGTTGACAAGGTTGAACATTTAGAAATCGGCCGCAAAGAATATTGGTGAGTTATTTAAAGATATTAACAGATGAATTGCATAACTGATATCAAGAGCATAAATAACCAAATGAAACTAGGAACGTTTCTATAATATGACCCCCCGGCTTCCGCGCGTCAAACCCCCCTACCCCCCTAAACTAGTAAGAAGGCTATTATTCCTGCAAACCCCCCGGAAACAGGAAACCCCCTACTAGCATTTTAGCCCGCCCAAATTTGTGTGTATTTACATTATTTGTAATATTGCAAAA

>JWM 11

GCTAGTGTAGCTTAACTAAAGCATAACACTGAAGATGTTAAGACAAACCTTAGATTGGTTTCACGAGCACAAAAGTTTGGTCCTGACTTTACTATCAACTTTAGCTAAACTTACACATGCAAGTATCCGCAATCCCGTGAGAATGCCCTACAGTTTCCTTAAAGGAAACAAGGAGCTGGTATCAGGCTCAATTACTCCCGCCCATGACACCTTGCTTAGCCACACCCCCAAGGGAACTCAGCAGTGATAGACATTAAGCAATAAGTGAAAACTTGACTTAATTAAAGCTAAGAGAACCGGTCAAACTCGTGCCAGCCACCGCGGTTATACGAGCGGTTCGAGCTGATAGACTACGGCGTAAAGCGTGGTTAATAAGAATAAAACTAAAGTCGAATGTTTTCAAAGCTGTTATACGCACTCGAAAATTAGAAGACCAGAAACGAAAGTGACTTTAACCCTATGAACCCACGAAAACTATGAAACAAACTGGGATTAGATACCCCACTATGCATAGCTGTAAACTTTGATGAACTATTACATTATCATCCGCCTGGGTACTACGAGCATTAGCTTAAAACCCAAAGGACTTGGCGGTGCTTTAGACCCACCTAGAGGAGCCTGTTCTAGAACCGATAACCCCCGTTAAACCTCACCCTCTCTTGTTTTTCCCGCCTATATACCGCCGTCGTCAGCTTACCCTGTGAAGGTCTAATAGTAAGCACAACCAGTTATACTCAAAACGTCAGGTCGAGGTGTAGCATATGAGAGGGGAAGAAATGGGCTACATTCCTTGTTTCAAGGAAAACGGATAACATAATGAAAGGTACGTTAGAAGGAGGATTTAGCAGTAAGCAGCAAATAGAGTGTTCTGCTGAAACTGGCCCTGAAGCGCGCACACACCGCCCGTCACTCTCCCCAACTCCGAGTTAAAAACCATATGTAAACCTTTGAAGGAACAAAGGGGAGGCAAGTCGTAACATGGTAAGTGTACCGGAAGGTGCACTTGGATAAATCAGAGTATAGCTAAGAAAGAAAAGCATCTCCCTTACACCGAGAAGTCATCCGTGCAAATCGGATTACCCTGACTCTAACAAGCTAGCCCAAAACCTTAACTTAAAAATCAAATATTTCTAATAATTAATAAACTAACCACATTAAATAAATCATTTTTCCCCCTGAGTATGGGAGACAGAAAAGGATAGAGGAGCTATAGACAAAGTACCGCAAGGGAAAGCTGAAAGAGAAATGAAACAAACCAGTAAAGAAGAACAAAGCAGAGATTAACCCTTGTACCTTTTGCATCATGAATTAGCCAGTTTAATCAAGCAAAGAGCACTGTAGTTTGAACCCCCGAAACTTAGTGAGCTACTTCAAGACAGCCTATGAAATAGGGCAAACCCGTCTCTGTGGCAAAAGAGTGGGAAGATCTTCAAGTAGAGGTGACAGACCTATCGAACTAAGTTATAGCTGGTTGCTCGTGAAATGAATAGAAGTTCAGCCTTTTGCTTTCTAAATTTCGATTTAGCACTACTTAGCCTAAATGACTAGAAAACAAAAGAGTTAGTCAAAGAGGGTACAGCCTGTTTGATAAAAGATACAACTTTACTAGGAGGATAAGAATCATAATTTTAAAGGTTTAATGCCCAGGTGGGCCTAAAAGCAGCCACCCTAATCAATAGCGTTAAAGCTTAAGCATAAAACGCACCTACAATTCTGATAAATCAGTTTAAATCCCCTAAGGTTAACGAGCTATTTCATACCTTATGAAAGAAATTATGCTAGTATGAGTAATAAGAAGTTACGAACTTCTCCCTGCACACGTGTAAATCGGAACGGACAAACCACCGAATCTTAACGGCCCCAGTCAAAGAGGGGATGTCGGATAAAAAAAAGAACAAGAAGCTCCCGACAAAACCACCGTTAACCCCACACCGGAGTGCTCCCTGGGAAAGACAAAAAGGGACAGAAGGAACTCGGCAAGTATGCTCAAGCCTCGCCTGTTTACCAAAAACATCGCCTCTTGTAAAAGTTAAATAAGAGGTACCGCCTGCCCTGTGACTAGTAGTTTAACGGCCGCGGTATTTTGACCGTGCAAAGGTAGCGCAATCACTTGCCTTTTAAATGAAGGCCTGTATGAATGGCACGACGAGGGCTTAACTGTCTCCTCTCCCTAGTCAATGAAATTGATCCCCCCGTGCAGAAGCGGGGATAATAACATAAGACGAGAAGACCCTGTGGAGCTTTAGACTATGAGCAGACCATGTCAAGAATAACAAACAAGTAAATTAAACAAATTGGTCCCTGCTTCTCTGTCTTTGGTTGGGGCGACCGCGGGATAATAAAAAACTCCCACGAGGATTGAGAACCCTTATCTTATAACCAAGAGCTTCTCCTCTAAGTAACAGAACATCTGACCTTAATGATCCGGCCTGGCCGATCAACGGACCGAGTTACCCCAGGGATAACAGCGCAATCCTCTTTTAGAGTCCATATCGACAAGAGGGTTTACGACCTCGATGTTGGATCAGGACATCCTAATGGTGCAGCCGCTATTAAGGGTTTGTTTGTTCAACAATTAAAGTCCTACGTGATCTGAGTTCAGACCGGAGTAATCCAGGTCAGTTTCTATCTATGACGTACTCTCTTCTAGTACGAAAGGACCGAAGAAAGAAGGCCTATGAAAAGTTATGCCTTAGTCTCACCTTATGAAGAAAACTAAATAAGACAAGAGGTTACACCCCTTAGTCATAGAAAATGACATGTTAAGGTGGCAGAGCCCGGATATTGCAAAAGACCTAAGCCCTTTCCACAGAGGTTCAATTCCTCTCCTTAACTATGTTCTCAACAATATTAAGCTTCATTATTAATCCCCTAATTGTTATGGTTTTTGTTTTGCTGGCAGTAGCCCTCTTGACCTTGGTAGAACGTAAAGTGCTAAGCTACATGCAACTTCGTAAAGGCCCAAATGTTGTTGGCCCTTACGGCCTTTTACAACCCTTCGCTGATGGCTTAAAACTTTTCATGAAAGAGCCCGTCCGACCCTCCACCTCCTCGCCCGCCTTATTCTTAATTACCCCTATTATAGCCCTTACCTTAGCCCTAACCCTCTGGGCCCCCCTTCCTATGCCTTTTCCCATCACCGACCTAAACTTAGGCATTTTATTTATTTTAGCACTATCGAGCCTGGCAGTATATTCTATTCTTGGCTCCGGATGGGCCTCCAATTCTAAATATGCACTAATTGGTGCTCTTCGAGCGGTCGCCCAAACCATCTCTTATGAGGTGAGCTTGGGCCTTATTCTTCTTAACACAATTGTCTTTACGGGGGGTTTTACTCTTCAAACCTTCAGCACCGCACAAGAAGCCACCTGATTACTTCTACCAGCTTGGCCACTAGCAGCCATGTGATATATTTCCACACTCGCGGAAACTAACCGGGCCCCTTTCGACCTAACTGAAGGAGAGTCCGAACTAGTGTCTGGCTTCAACGTAGAGTATGCCGGCGGACCTTTTGCCCTTTTTTTTCTGGCAGAATACGGTAACATTTTACTTATAAATACCCTCTCAGCAGTACTATTTCTAGGCTCTTCAACCTACCACAGCTTTCCAGAACTAACCGCGACCTTATTAATGCTTAAAGCCACCCTCCTTTCAGTCGTATTTTTATGAGTGCGAGCATCTTACCCTCGGTTTCGATACGATCAACTAATACATTTAATTTGAAAAAACTTTTTACCTCTGACCCTAGCACTAGTTATTTGACACCTTTCTCTTCCGATCACGTTGAGCGGCCTCCCCCCTCAACTTTAACTGGGGAAATGTGCCTGAAAAAGGGTCACTTTGATAGGGTGAATAATGAGGGTTAAAGCCCCTCCATCTCCTTAGAAAGAAGGGGTTTGAACCCTACCTGAAGAGATCAAAACTCTTAGTGCTTCCACTACACCACTTCCTAGTAAAGTCAGCTAATAAAAGCTTTTGGGCCCATACCCCAAATATGTTGGTTAAAATCCTTCCTTTGCTAATGAATCCTTACGTCCTTTCAATTCTACTTATAGGTTTAGGCCTCGGCACTACAGTCACATTCGCTAGCTCACACTGACTATTAGCATGAATAGGCCTTGAAATAAATACCCTCGCCATTTTGCCGTTAATAGCACAACATCACCACCCCCGAGCCGTTGAAGCCACCACCAAGTATTTTTTAATTCAATCGGCAGCCGCAGCAACCATCTTATTTGCCAGCTCAACTAACGCCTGACTTTCGGGCCAGTGGGACATCATAAGTATTAATCACCCTCTTCCAACCGTCATAATTACAGTCGCTCTGTCCTTAAAACTAGGCTTGGCCCCTCTTCACGCGTGACTTCCCGAAGTAATTCAAGGCCTAGACTTAACCACGGGCTTAATCCTCTCCACATGACAAAAACTCGCACCCTTTGCCCTCCTCGTTCAAATCTTCCCCGACACTCCCCTTCTCATCACTTCTCTAGGACTTCTTTCAATATTAGTTGGGGGGTGAGGGGGTTTAAACCACACACAACTCCGCAAAGTGCTCGCATATTCTTCGATCGCCCACTTAGGCTGAATGATAGTAATTATGCAATTCTCCACCCCCCTTACAATTCTTGCTTTATCAACATACATTGTTATAACATCATCTACTTTTCTAATCTTTAAACTCCTTAAATCCACAGATATGAACAGCCTGGCAACATCTTGAGCTAAAACCCCCTCCATTACAGCCCTGGCACCTTTAGTGCTATTATCCTTGGGCGGACTCCCTCCCCTCTCGGGCTTTATGCCAAAATGACTAATTATTCAAGAGTTAACTAAACAAGATCTAGCCCTAGTTGCGACCTTAGCCGCCCTCTCTGCGCTACTCAGCCTTTTCTTTTACTTACGCATTTGTTACTCCCTCACATTTACCTCCTCTCCTAATAATCTCATGGGAACACCCCCCTGACGACTAATAACAAAGCAAGTATCACTTCCCCTGGCTATAACAACCTCCCTCTCTATTCTTCTACTCCCGGTTACCCCTGCAATCTTATCAGTGGTTCTCCCTTTGTAAAGAGGCTTAGGATAGTATTAAGACCAAGGGCCTTCAAAGCCCTAAGCGGGAGTGAAAGCCCCCCCAGCCTCTGTAAGACCTACGGGACACTAACCCACATCTTCTGTATGCAAAACAGACACTTTAATTAAGCTAAAGCCTTCCTAGGTGGGTAGGCCTCGATCCTACAATCTCTTAGTTAACAGCTAAGCGCCTAAACCAGCGGGCATCCATCTACCTTTCCCCCGCCTTGCCGAAAAAAAAAGGCGGGGGAAAGCCCCGGCAGGGTATTAGCCTGCTACTTAAGATTTGCAATCTAATGTGTTAACACCTCGGAGCTGGTAAGAAGAGGACTTTAACCTCTGTCTATGGGGCTACAATCCACCGCTAAACGCTCAGCCACCTTACCTGTGGCAATCACACGTTGATTTTTCTCAACTAATCACAAAGACATCGGCACCCTATATCTAATCTTTGGTGCCTGGGCGGGAATAGTAGGGACGGCCTTAAGTCTACTCATTCGGGCAGAATTAAGTCAACCAGGCTCCCTATTAGGAGACGACCAGATCTATAACGTAATTGTAACTGCACATGCTTTCGTAATAATTTTCTTTATAGTAATGCCAATCATAATTGGAGGATTTGGTAACTGATTAATTCCTTTAATGATCGGAGCTCCCGACATGGCCTTCCCCCGGATAAATAATATAAGCTTTTGACTCCTGCCCCCTTCTTTCCTTCTATTATTGGCCTCATCTGGTGTAGAAGCTGGTGCCGGGACAGGATGAACCGTATATCCCCCCTTGTCCGGTAATTTGGCACACGCAGGGGCCTCCGTAGATTTAACCATTTTCTCTCTGCACCTGGCCGGAATTTCTTCTATTCTAGGGGCCATTAATTTCATTACAACTATTATTAATATAAAACCTCCAGCCATTTCCCAATATCAAACCCCTTTATTTGTCTGAGCTGTTCTAATTACCGCAGTATTACTCCTACTCTCTCTTCCTGTTCTAGCTGCGGGTATCACTATGCTTCTCACAGATCGAAACCTAAATACAACATTTTTCGACCCCGCAGGAGGGGGGGACCCCATTCTTTATCAACATTTATTCTGATTCTTTGGGCATCCTGAAGTCTACATTCTGATTTTGCCCGGCTTCGGAATGATTTCTCACATTGTAGCATATTACTCAGGCAAAAAAGAGCCGTTTGGCTACATGGGAATAGTATGAGCTATAATAGCAATTGGCCTACTGGGGTTTATCGTATGAGCCCATCATATGTTCACTGTAGGAATGGACGTGGACACTCGAGCTTATTTTACATCCGCCACTATAATTATCGCAATTCCTACAGGAGTCAAAGTGTTTAGTTGACTAGCTACCTTGCATGGGGGCTCAATCAAATGAGAGACCCCCCTGTTATGAGCTCTAGGCTTTATTTTCTTATTTACTGTTGGAGGTTTAACAGGAATTGTTTTAGCCAACTCATCTCTGGATATTATACTTCATGACACATACTACGTTGTAGCCCACTTCCACTATGTCCTCTCTATAGGAGCAGTCTTTGCCATCATGGGGGCATTCGTTCACTGATTCCCCCTATTCTCAGGCTACACCCTTCACAATACGTGAACAAAAATCCACTTCGGAGTTATGTTTGTAGGTGTAAACCTCACCTTTTTCCCTCAGCACTTCTTAGGATTGGCGGGAATACCTCGACGATATTCAGATTACCCTGACGCATACACACTGTGAAATACTATCTCATCTCTGGGGTCACTAATCTCCCTTATTGCTGTAATTATATTCCTATTTATTATCTGGGAAGCATTCGCGGCAAAACGTGAAGTCTTATCAGTTGAACTAACAGCCACAAACGTAGAATGACTGCACGGGTGTCCTCCCCCTTACCATACATTTGAAGAACCCGCATTCGTTCAAATTCAGCAATCCAAATTTTAATCGAGAAAGGAAGGAGTCGAACCCCCATAAACTGGTTTCAAGCCAGCCACATAACCGCTCTGTCACTTTCTTCCCTAAGTTAATAAGATTCTAGTTAAAAGAATAACGCTGCCTTGTCAAGGCAAAATTGTGGGTTAAAGCCCCACGTATCTTGCTTATGGCACATCCATCTCAACTAGGATTCCAAGATGCAGCTTCACCCGTTATAGAAGAACTTCTCCATTTTCATGACCATGCATTAATAATTGTTTTCTTAATCAGCACCCTTGTTCTTTACATTATTGTGGCTATGGTAACCACCAAGCTAACAAATAAGTTCATTCTGGACTCCCAAGAAATTGAAATCATCTGAACCTTACTACCAGCAATTATCCTAATTCTGATCGCCCTGCCCTCCCTTCGCATTCTCTACCTCATGGATGAAATCAATGACCCCCACCTCACAATTAAAGCCATAGGACATCAATGATACTGAAGCTACGAATATACGGATTATGAAGACCTGGGGTTCGACTCATATATGGTCCCTACACAAGATCTCGCCCCTGGTCAATTTCGACTACTTGAGACAGACCACCGCATGGTTATTCCTGTTGAGTCTCCCATCCGAGTTCTTGTCTCCGCCGAGGATGTCTTACACTCATGAGCCGTCCCGAGCCTCGGAGTAAAAATGGACGCCGTCCCCGGCCGCCTAAATCAAACAGCCTTCATTACTTCCCGCCCAGGTGTGTTTTATGGACAATGCTCAGAAATTTGCGGAGCTAATCATAGCTTTATACCCATTGTAGTGGAGGCTGTTCCTCTAGAACACTTCGAGAACTGGTCTTACCTAATACTTCAAGATGCCTCACCAGGAAGCTAAAAGGGAATAGCATTAGCCTTTTAAGCTAAAAATTGGTGACTCCCGCCCACCCCTGGTGACATGCCTCAGTTGAACCCCGCACCCTGATTTGCTATTATAGTATTCTCGTGACTAGTTTTCCTAGCCGTTATTCCACCTAAAGTTCTAGCTCACCATTTTCCCAATGACCCCGCCCCACAAAGCGTAAAAAAATCAAAAACAGAGACTTGACCCTGACCATGACTTTAAGCCTCTTTGATCAATTTATGAGCCCTACACTTCTAGGGGTGCCTCTTATCGGACTCGCCCTAACATTACCATGAGTCCTTTACTTCCGACCCGGTGCCCGATGACTTAATAACCGCTTGATTACCCTTCAATCTATATTCATGAACTGGTTTGTAAAACAAATCTTTCAGCCAATAAGCTTAGGCGGACACAAATGGGCTGCTCTCCTCATATCTTTAATACTATTTTTAATCACCTTAAATATGCTAGGCCTGCTGCCTTACACATTTACTCCAACAACGCAGCTGTCACTTAATATAGCCTTTGCAGTTCCACTTTGACTAGCAACTGTCATTATTGGAATACGAAACCAGCCAACACATGCCCTTGGTCACCTTCTCCCCGAAGGGACTCCTACCGCCCTAATCCCGGTTTTAATCGTGATTGAAACAATTAGCCTTTTTATTCGACCCTTGGCCCTCGGTGTTCGACTTACCGCAAACTTGACAGCCGGACACCTTCTAATTCAACTAATTGCAACTGCGGCTTTTGTTCTTTTCCCTATAATACCTACAGTAGCTACTCTTACCTCTGTCTTACTATTCTTGCTAACCCTGCTAGAAGTCGCCGTGGCCATAATCCAAGCCTATGTATTTGTACTTCTTTTAAGCCTTTATCTACAAGAAAACGTCTAATGGCCCATCAAGCACATGCATATCATATAGTTGACCCAAGCCCTTGACCCCTCACAGGCGCAGTAGCCGCCCTTCTACTTACGTCTGGAACAGCAATCTGAATACACTTTAACTCCACAGTTCTCATGTCCCTTGGACTTGTCCTGCTACTATTAACCATATATCAATGATGGCGGGACATTATCCGAGAGGGAACCTTTCAAGGTCATCATACACCCCCTGTTCAAAAGGGCCTTCGGTACGGGATAATTCTATTTATTACCTCAGAGGTCTTCTTTTTCCTAGGTTTCTTCTGAGCATTTTATCACTCAAGCCTAGCCCCAACCCCCGAACTTGGCGGGTGTTGACCACCTATGGGTATTACAACACTGGACCCCTTTGAAGTCCCCCTTCTCAATACTGCTGTCCTTCTCGCCTCCGGTGTCACGGTCACTTGAGCTCACCATAGTATTATGGAGGGGCAGCGAAAACAAGCAATTCAGTCCTTAACACTCACAATTCTCCTGGGGTTCTACTTTACGTTCCTTCAAGCAATAGAGTACTACGAGGCACCCTTCACCATTGCAGATGGCGTCTATGGCTCTACATTTTTTGTGGCAACGGGGTTTCATGGCCTCCATGTAATTATTGGATCAACATTCCTGGCAGTCTGCCTCTTACGACAAGTCCAATTCCACTTTACATCAGAACATCACTTCGGATTTGAAGCTGCAGCATGATACTGACACTTTGTAGACGTAGTCTGACTATTCTTATATATCTCTATCTACTGATGAGGCTCATATCTTTCTAGTATTAAAAAGTACAAGTGACTTCCAATCACTCAGTCTTGGTTAGACTCCAAGGAAAGATAATGAACTTAGTACTAGTCATTATTTGCATCTCATTAGCCCTCGCCGCACTGCTCGCAACTGTTTCATTTTTCCTCCCACAAATAACCCCTGATTATGAGAAACTCTCACCGTATGAGTGCGGCTTTGATCCAGTGGGGTCCGCCCGTTTGCCATTCTCCATTCGCTTTTTTCTAGTCGCAATCCTATTTCTCCTCTTCGACTTAGAAATTGCCTTACTCCTTCCCCTTCCCTGAGGGGACCAACTCCCCTCCCCTCTGACAACTTTCTTCTGAGCTTCTGCTATCCTTATGCTACTAACTCTAGGGTTAATCTATGAATGACTTCAAGGGGGCCTAGAATGGGCAGAATAGGTACTTAGTTTAATAAAAACATTTGATTTCGGCTCAAAAACTTATGGTTTAAGTCCATATTTACCTGATGACCTTAACTCACTATGCATTCTCGTCAGCCTACTTTGTCAGCTTCATGGGTCTAATTTTTTACCGAAAGCATCTTCTCTCCGTCTTACTTTGCTTAGAAGCCATAATACTTATTCTTTTTATTTCACTATGCCTGTGAGGCCTAGTCTTAGCCTCAAGTGCATTTTCGGCAGGCCCAATGATCTTACTTGCTTTCTCAGCATGTGAAGCAAGTGCAGGCCTAGCACTGCTTGTAGCAATAGCTCGAACCCACGGGACTGACCGTTTAAAAAACCTAAGCCTACTCCAATGTTAATAATTCTTATTCCTACTGTTATGCTTCTACCCACAATCTGACTAAGCCCCGCTAAATACCTGTGGTCCTCAACACTTGGCCATAGCATAATAATTGCTCTTATAAGCCTCTCCTGACTTAGCCTCCCAGGGGAGGTTGGCTGATCTTCCCTTAACACTTTTATAGCAACAGACCCTCTCTCTACCCCCCTTCTCGTACTTACTTGCTGACTTCTACCCTTAATAATTCTTGCGAGCCAAAACCATATAGCCCAAGAACCTACCAATCGCCAGCGAACCTATATCTCTTTACTTACTTCCCTTCAAATCTTCTTAATCTTAGCATTTGGAGCAACCGAGATAATTATGTTCTACATTATATTTGAAGCGACCTTAATTCCCACACTCGTAATTATCACACGATGAGGAAACCAAACAGAGCGATTAAACGCAGGTATTTACTTTTTATTTTATACCTTAGCTGGCTCTTTACCACTACTAGTGGCACTTCTTCTACTTCAGACCTCGACAGGAACTCTTTCTTTTCTAACCACTCAATTTTTTTCCCCCTTACAACTGCATACAGAAGCAAGTAAGTTCTGGTGGGCGGGCTGTTTACTAGCATTCTTAGTAAAAATACCGCTATATGGGGCACACCTTTGACTTCCAAAAGCTCACGTCGAAGCCCCCATCGCCGGGTCAATAGTCCTTGCAGCCGTTCTTTTAAAACTAGGGGGTTACGGTATGATACGAGTCATTATTATCTTAGACCCCTTAACGAAACAACTCAGCTACCCCTTTATTGTTCTTGCCCTGTGGGGCGTTGTAATAACTGGCTCAATCTGCCTCCGACAAACAGACCTTAAATCACTAATCGCTTACTCCTCAGTAAGCCACATAGGCCTTGTTGCAGCAGGCATCCTGATCCAAACTCCTTGGGGGTTTACAGGAGCATTAATCCTTATAATTGCCCATGGCTTAACTTCCTCCGCCCTATTCTGTTTAGCCAACACTAACTATGAGCGAACACATAGCCGAACCATGCTTTTAGCCCGGGGCCTACAAATGGTCCTTCCTCTCTTAGCAACTTGATGGTTTCTATTTACCCTCGCCAACCTAGCACTCCCCCCGCTACCCAACCTCATAGGAGAACTTATGATTATCTCATCCCTGTATAACTGGTCAAACTGGTCTTTAGTCCTGACCGGGGCGGGAGTACTAATTACCGCTAGCTACTCTCTTCATATATTCCTAACCACTCAACGCGGCCCTATTACTAACCCCGTCTTGGCAATTGAACCAACCCACACACGAGAACATCTCCTCATAATTCTTCACCTTCTTCCCCTCCTCCTTCTAATTTTAAAACCCTGCTTGATCTGGGGCTGAACAGTTTGTAGGCGTAGTTTAAATAAAGCGCTAGATTGTGATTCTAGAAATAAGAGTTAAACCCTCTTCACCCACCGAGAGGGGTCGCCGTGACAGCAAGAACTGCTAATTCTAGCCCCTTTGGTTAAAATCCGAAGCCCACTCGAACAGGCTTCTAAAGGATAATAGCTCATCCGTTGGTCTTAGGAACCAAAAACTCTTGGTGCAACTCCAAGTAGCAGCTATGCACTTTACAACAATGATTCTCTCCTCAAGCCTAATAACAATTTTTCTTCTTCTCATCCTTCCAGTCCTAGGTACACTAAACCCTAACCCCACGGGGGGCCTGTGAGCCACAAAAAACGTTAAAACGGCAGTTAAGATAGCCTTTTTTGTAAGTCTTTTGCCTCTTTTTATCTTTCTTAATGAAGGGGTAGAGACTGTTATAACAAACTGAAAATGAATAAATACTCTAATGTTTGAAATTAATATCAGCTTTAAATTTGACCTCTACTCCGTAGTGTTTACCCCTGTGGCCCTCTACGTAACATGATCAATTTTAGAGTTCGCATCTTGGTATATACACAGTGACCCCAACATAAACCGGTTCTTTAAATATCTTCTAATCTTTCTCATTGCTATGGTCGTTCTGGTCACAGCCAACAACATGTTCCAACTATTTATTGGCTGAGAAGGTGTTGGAATTATGTCTTTCTTACTTATTGGCTGGTGATTCGGGCGGGCTGACGCCAACACTGCAGCCCTCCAGGCCGTAGTTTATAACCGAGTTGGTGATATCGGCCTGATTCTAGCAATAGCATGAATAGTAGTAAACCTAAACTCATGAGAAATACAACAGCTCTTTTCTATGTCTAAAGGCCATGATATAACCCTTCCTTTATTAGGCCTAGTACTGGCCGCTACCGGAAAGTCCGCCCAGTTTGGACTTCACCCCTGGCTCCCGTCAGCCATAGAGGGTCCAACACCGGTCTCTGCCCTCCTGCACTCTAGCACCATGGTTGTTGCTGGTATTTTTCTTCTTATTCGCCTCAGCCCCTTAATGCAAGAAAGCCCGTTAATTCTCTCAACATGCCTTTGCCTGGGGGCCCTAACTACCGTCTTTACTGCAACATGTGCCCTTACCCAAAATGACATTAAAAAAATTGTTGCATTTTCTACATCAAGTCAATTAGGACTAATAATAGTTACCATCGGACTAGGCCAGCCCCAGCTCGCCTTTCTTCATATCTGCACCCACGCCTTCTTTAAAGCAATACTTTTCTTATGTTCCGGCTCCATTATTCATAGCCTTAATGATGAGCAAGATATCCGAAAAATAGGCGGGCTTCACAAGGTGCTTCCACTGACCTCTTCTTGTCTAACCATTGGCAGCCTAGCTCTAACAGGAGTCCCCTTTTTAGCAGGCTTCTTTTCCAAAGACGCCATCATTGAAGCTATAAATACATCCTACCTTAACGCCTGAGCCCTAATTTTAACGCTTCTAGCTACATCATTTACCGCAGTTTACAGTCTCCGAGTCGTATTCTTTGCCTCTATGGGCCACCCGCGTTTTAATCCAATCTCCCCAATTAATGAAAATAACCCTACAGTGATAAACCCCCTTAAACGACTCGCTTGGGGAAGCATTTTGGCAGGGTTGCTAATTACGACCAATATTGTTCCACTTAAAACCCCCGTTTTAACCATGCCTTTCACCTTAAAAATGGCCGCACTGACTGTAACAATCATAGGACTACTCACAGCCTTAGAACTAGCGTCTCTCACGTCCCAACAATTTAAAATCAAACCCTTATCTTCTACTCACCACTTCTCAAATATATTAGGATTTTTCCCCAGTGTAGTCCATCGACTAGTCCCAAAAACTGGCCTGATTCTTGGACAACTAGTTGCCAATCAGACAGTTGACCAAACCTGACTAGAGAAAACCGGGCCAAAAATAGTAACCTCCGTTAACCTTCCAATAGCTACTTCAATTAGCAGCCTGCAGCAGGGTGTAATTAAAACCTACTTCTTATTATTTTTCTTCACCATAATACTGGCAATTCTCATCCTTGTCGTCTAACTGCCCGTAAGGTCCCCCGACTTAGCCCCCGAGTTAACTCTAGAACTACAAAAAGCGTCAGTAATAAAACCCATCCCCCAAGCATTAAAACCCCTCCTCCTGAAGAATATATCAAAGCAACCCCACCAAAATCCCCCCGAAAGAGCATGAATTCACTAAATTCGTCAGCAGTTATCCATGAACCCTCATACCAGCCCTCGGAGAAAAAAACAGAGATAGACGCGACCAGAAACACATATACTGACATAAGAAGCAAAACGGGTCAACTTCCCCACCCCTCAGGATAAGGCTCCGAAGCCAGCGCTGCTGAGTACGCAAACACAACTAACATCCCACCTAAATAAATCAAAAACAAAATCAGAGATAAAAATGAACCCCCATGCCCTACTAAAATGCCACAGCCCATTCCTGCTACTGTAACAAGCCCCAAAGCAGCAAAGTAAGGTGACGGGTTCGAGGCCACGGCCGCTAGACCTAAAACCAAACCAACTAATAATAAATAAGTCATATAAACCATAATTCTTGCCAGGATTTTAACCAGGGCCTGCGACTTGAAAAACCACCGTTGTACTCAACTACAAGAACCTAATGGCCAATCTTCGAAAAACCCATCCCCTATTAAAAATCGCAAACGATGCCCTCGTTGATCTCCCAGCCCCATCGAACATTTCAGTTTGATGAAACTTCGGATCTCTTCTAGGACTTTGTTTGGCCGCCCAGATCGTTACGGGCCTTTTCCTTGCAATACATTATACATCAGACATTGCCACAGCATTTTCATCTGTCGCACATATTTGTCGTGATGTTAACTACGGCTGACTAATCCGAAACATGCATGCAAACGGTGCTTCCTTTTTCTTCATTTGCATCTACCTGCACATCGGACGGGGCTTGTATTATGGATCATACTTATATAAAGAGACATGAAATGTAGGTGTTGTCCTTCTCCTCCTTGTGATAATGACTGCTTTCGTAGGCTACGTCCTACCCTGAGGACAAATATCATTCTGAGGGGCTACCGTCATTACCAACCTTTTATCAGCCATCCCCTACATTGGAAACGCCCTAGTTCAATGGATCTGAGGCGGATTTTCAGTAGACAACGCCACCCTTACCCGGTTCTTTGCCTTCCATTTCCTCCTCCCCTTTGTAATTGCTGCTGCTACAGTTGTACATCTTATTTTCCTGCACGAGACAGGGTCGAATAACCCAACGGGTTTAAACTCAGACTCTGACAAAGTGTCTTTTCACCCCTACTTTTCTTATAAAGATCTTCTAGGCTTTGCTGCCCTGCTAGTAGCCCTTATCTCTTTAGCCCTCTTCTCCCCTAATCTACTTGGAGACCCTGACAACTTTACCCCTGCTAATCCTTTAGTAACTCCACCTCACATCAAACCTGAGTGATACTTCCTGTTCGCTTACGCCATTCTACGATCCATCCCAAACAAGCTTGGCGGGGTTCTAGCCCTATTAGCCTCTATTCTAGTTCTCTTTCTTGTCCCTATCCTGCACACATCAAAACAACGAAGCCTAACATTCCGACCCCTAACCCAATTCCTCTTCTGACTGCTAGTCGCCGATGTAATAATTTTAACCTGAATCGGAGGTATGCCTGTAGAACACCCTTACATTATCATTGGACAAGTCGCATCCTTCATTTATTTCTCCCTTTTTCTAGTCATGGCGCCTATGGCCGGCCTACTAGAAAACAAAGTCTTAAAATGACAATGCATTAGAAGCTCAGATGAAAGAGCACCGGTCTTGTAAGCCAGAGGTCGAAGGTTCAAGCCCTTCCTAATGCTCAGAGAGAAGGGATTCTAACCCCTGCCCCTGGCTCCCAAAGCCAGGATTCTTAGCTAAACTACTCGCTGATTTTCATACACCAGTTTTGCAATCCAGAGCGCATCACTTTTGCTACCAACGTTAAATTAACGTTGCACAAACGTTGCATCAGCACCCCATGGACACTAAATGACGCGAGGACGTTGAATAGACACCCCCTACCTCTAGCACCCTTTTAACGATTTCACTTTTTTTTTTTTTTTTGTTTAACGATTACGTTTTTTTTTGCGTTCCCGGACTCTGCCAGATTTCGACCGAAAACTGCCAGAATCCGCTCAAAATCCGCTCAAATACCAATATGTATTATCCCCATAAATGGTTTAAACCATTTTTGCCTAGTACACACTGACCATGCAAGTCAATTATATTTACCCCGCGCTCCAGGCCGCAGTACATACACCTACAGTTGGTGTATTTAGCACAAGTGTGCCTCAGCTAGTTTCAAGTCACCCACATCCTTCCTTTAATTGTTACTTAATGTAGTAAGAGCCCACCATCAGTTGATTCCTTAATGTCAACGGTTCTTGAAGGTGAGGGACAAAAATCGTGGGGGTTTCACCTCTTGAATTATTCCTGGCATTTGGCTCTACATCTCAAGGCCATACATTTCTCGTCTCTCACACTTTCACTGGCCCTGACATTGGTTAATGGTGGAGTACATACTCCTCGTTACCCCCCATGCCGGGCGTTCTTTCTAATGGACAACGGGTTTTCCTTTTTTTTTCCTTTTCACTTGGCATTTCACAGTGCATACAGACCTTGTTGACAAGGTTGAACATTTAGAAACCGGCCGCAAAGAATATTGGTGAATTATTTAAAGATATTAACAGATGAATTGCATAACTGATATCAAGAGCATAAATAACCAAATGAAACTAGGAACGTTTCTATAATATGACCCCCCGGCTTCCGCGCGTCAAACCCCCCTACCCCCCTAAACTAGTAAGAAGGCTATTATTCCTGCAAACCCCCCGGAAACAGGAAACCCCCTACTAGCATTTTAGCCCGCCCAAATTTGTGTGTATTTACATTATTTGTAATATTGCAAAA

>JWM 12

GCTAGTGTAGCTTAACTAAAGCATAACACTGAAGATGTTAAGACAAACCTTAGATTGGTTTCACGAGCACAAAAGTTTGGTCCTGACTTTACTATCAACTTTAGCTAAACTTACACATGCAAGTATCCGCAATCCCGTGAGAATGCCCTACAGTTTCCTTAAAGGAAACAAGGAGCTGGTATCAGGCTCAATTACTCCCGCCCATGACACCTTGCTTAGCCACACCCCCAAGGGAACTCAGCAGTGATAGACATTAAGCAATAAGTGAAAACTTGACTTAATTAAAGCTAAGAGAACCGGTCAAACTCGTGCCAGCCACCGCGGTTATACGAGCGGTTCGAGCTGATAGACTACGGCGTAAAGCGTGGTTAATAAGAATAAAACTAAAGTCGAATGTTTTCAAAGCTGTTATACGCACTCGAAAATTAGAAGACCAGAAACGAAAGTGACTTTAACCCTATGAACCCACGAAAACTATGAAACAAACTGGGATTAGATACCCCACTATGCATAGCTGTAAACTTTGATGAACTATTACATTATCATCCGCCTGGGTACTACGAGCATTAGCTTAAAACCCAAAGGACTTGGCGGTGCTTTAGACCCACCTAGAGGAGCCTGTTCTAGAACCGATAACCCCCGTTAAACCTCACCCTCTCTTGTTTTTCCCGCCTATATACCGCCGTCGTCAGCTTACCCTGTGAAGGTCTAATAGTAAGCACAACCAGTTATACTCAAAACGTCAGGTCGAGGTGTAGCATATGAGAGGGGAAGAAATGGGCTACATTCCTTGTTTCAAGGAAAACGGATAACATAATGAAAGGTACGTTAGAAGGAGGATTTAGCAGTAAGCAGCAAATAGAGTGTTCTGCTGAAACTGGCCCTGAAGCGCGCACACACCGCCCGTCACTCTCCCCAACTCCGAGTTAAAAACCATATGTAAACCTTTGAAGGAACAAAGGGGAGGCAAGTCGTAACATGGTAAGTGTACCGGAAGGTGCACTTGGATAAATCAGAGTATAGCTAAGAAAGAAAAGCATCTCCCTTACACCGAGAAGTCATCCGTGCAAATCGGATTACCCTGACTCTAACAAGCTAGCCCAAAACCTTAACTTAAAAATCAAATATTTCTAATAATTAATAAACTAACCACATTAAATAAATCATTTTTCCCCCTGAGTATGGGAGACAGAAAAGGATAGAGGAGCTATAGACAAAGTACCGCAAGGGAAAGCTGAAAGAGAAATGAAACAAACCAGTAAAGAAGAACAAAGCAGAGATTAACCCTTGTACCTTTTGCATCATGAATTAGCCAGTTTAATCAAGCAAAGAGCACTGTAGTTTGAACCCCCGAAACTTAGTGAGCTACTTCAAGACAGCCTATGAAATAGGGCAAACCCGTCTCTGTGGCAAAAGAGTGGGAAGATCTTCAAGTAGAGGTGACAGACCTATCGAACTAAGTTATAGCTGGTTGCTCGTGAAATGAATAGAAGTTCAGCCTTTTGCTTTCTAAATTTCGATTTAGCACTACTTAGCCTAAATGACTAGAAAACAAAAGAGTTAGTCAAAGAGGGTACAGCCTGTTTGATAAAAGATACAACTTTACTAGGAGGATAAGAATCATAATTTTAAAGGTTTAATGCCCAGGTGGGCCTAAAAGCAGCCACCCTAATCAATAGCGTTAAAGCTTAAGCATAAAACGCACCTACAATTCTGATAAATCAGTTTAAATCCCCTAAGGTTAACGAGCTATTTCATACCTTATGAAAGAAATTATGCTAGTATGAGTAATAAGAAGTTACGAACTTCTCCCTGCACACGTGTAAATCGGAACGGACAAACCACCGAATCTTAACGGCCCCAGTCAAAGAGGGGATGTCGGATAAAAAAAAGAACAAGAAGCTCCCGACAAAACCACCGTTAACCCCACACCGGAGTGCTCCCTGGGAAAGACAAAAAGGGACAGAAGGAACTCGGCAAGTATGCTCAAGCCTCGCCTGTTTACCAAAAACATCGCCTCTTGTAAAAGTTAAATAAGAGGTACCGCCTGCCCTGTGACTAGTAGTTTAACGGCCGCGGTATTTTGACCGTGCAAAGGTAGCGCAATCACTTGCCTTTTAAATGAAGGCCTGTATGAATGGCACGACGAGGGCTTAACTGTCTCCTCTCCCTAGTCAATGAAATTGATCCCCCCGTGCAGAAGCGGGGATAATAACATAAGACGAGAAGACCCTGTGGAGCTTTAGACTATGAGCAGACCATGTCAAGAATAACAAACAAGTAAATTAAACAAATTGGTCCCTGCTTCTCTGTCTTTGGTTGGGGCGACCGCGGGATAATAAAAAACTCCCACGAGGATTGAGAACCCTTATCTTATAACCAAGAGCTTCTCCTCTAAGTAACAGAACATCTGACCTTAATGATCCGGCCTGGCCGATCAACGGACCGAGTTACCCCAGGGATAACAGCGCAATCCTCTTTTAGAGTCCATATCGACAAGAGGGTTTACGACCTCGATGTTGGATCAGGACATCCTAATGGTGCAGCCGCTATTAAGGGTTTGTTTGTTCAACAATTAAAGTCCTACGTGATCTGAGTTCAGACCGGAGTAATCCAGGTCAGTTTCTATCTATGACGTACTCTCTTCTAGTACGAAAGGACCGAAGAAAGAAGGCCTATGAAAAGTTATGCCTTAGTCTCACCTTATGAAGAAAACTAAATAAGACAAGAGGTTACACCCCTTAGTCATAGAAAATGACATGTTAAGGTGGCAGAGCCCGGATATTGCAAAAGACCTAAGCCCTTTCCACAGAGGTTCAATTCCTCTCCTTAACTATGTTCTCAACAATATTAAGCTTCATTATTAATCCCCTAATTGTTATGGTTTTTGTTTTGCTGGCAGTAGCCCTCTTGACCTTGGTAGAACGTAAAGTGCTAAGCTACATGCAACTTCGTAAAGGCCCAAATGTTGTTGGCCCTTACGGCCTTTTACAACCCTTCGCTGATGGCTTAAAACTTTTCATGAAAGAGCCCGTCCGACCCTCCACCTCCTCGCCCGCCTTATTCTTAATTACCCCTATTATAGCCCTTACCTTAGCCCTAACCCTCTGGGCCCCCCTTCCTATGCCTTTTCCCATCACCGACCTAAACTTAGGCATTTTATTTATTTTAGCACTATCGAGCCTGGCAGTATATTCTATTCTTGGCTCCGGATGGGCCTCCAATTCTAAATATGCACTAATTGGTGCTCTTCGAGCGGTCGCCCAAACCATCTCTTATGAGGTGAGCTTGGGCCTTATTCTTCTTAACACAATTGTCTTTACGGGGGGTTTTACTCTTCAAACCTTCAGCACCGCACAAGAAGCCACCTGATTACTTCTACCAGCTTGGCCACTAGCAGCCATGTGATATATTTCCACACTCGCGGAAACTAACCGGGCCCCTTTCGACCTAACTGAAGGAGAGTCCGAACTAGTGTCTGGCTTCAACGTAGAGTATGCCGGCGGACCTTTTGCCCTTTTTTTTCTGGCAGAATACGGTAACATTTTACTTATAAATACCCTCTCAGCAGTACTATTTCTAGGCTCTTCAACCTACCACAGCTTTCCAGAACTAACCGCGACCTTATTAATGCTTAAAGCCACCCTCCTTTCAGTCGTATTTTTATGAGTGCGAGCATCTTACCCTCGGTTTCGATACGATCAACTAATACATTTAATTTGAAAAAACTTTTTACCTCTGACCCTAGCACTAGTTATTTGACACCTTTCTCTTCCGATCACGTTGAGCGGCCTCCCCCCTCAACTTTAACTGGGGAAATGTGCCTGAAAAAGGGTCACTTTGATAGGGTGAATAATGAGGGTTAAAGCCCCTCCATCTCCTTAGAAAGAAGGGGTTTGAACCCTACCTGAAGAGATCAAAACTCTTAGTGCTTCCACTACACCACTTCCTAGTAAAGTCAGCTAATAAAAGCTTTTGGGCCCATACCCCAAATATGTTGGTTAAAATCCTTCCTTTGCTAATGAATCCTTACGTCCTTTCAATTCTACTTATAGGTTTAGGCCTCGGCACTACAGTCACATTCGCTAGCTCACACTGACTATTAGCATGAATAGGCCTTGAAATAAATACCCTCGCCATTTTGCCGTTAATAGCACAACATCACCACCCCCGAGCCGTTGAAGCCACCACCAAGTATTTTTTAATTCAATCGGCAGCCGCAGCAACCATCTTATTTGCCAGCTCAACTAACGCCTGACTTTCGGGCCAGTGGGACATCATAAGTATTAATCACCCTCTTCCAACCGTCATAATTACAGTCGCTCTGTCCTTAAAACTAGGCTTGGCCCCTCTTCACGCGTGACTTCCCGAAGTAATTCAAGGCCTAGACTTAACCACGGGCTTAATCCTCTCCACATGACAAAAACTCGCACCCTTTGCCCTCCTCGTTCAAATCTTCCCCGACACTCCCCTTCTCATCACTTCTCTAGGACTTCTTTCAATATTAGTTGGGGGGTGAGGGGGTTTAAACCACACACAACTCCGCAAAGTGCTCGCATATTCTTCGATCGCCCACTTAGGCTGAATGATAGTAATTATGCAATTCTCCACCCCCCTTACAATTCTTGCTTTATCAACATACATTGTTATAACATCATCTACTTTTCTAATCTTTAAACTCCTTAAATCCACAGATATGAACAGCCTGGCAACATCTTGAGCTAAAACCCCCTCCATTACAGCCCTGGCACCTTTAGTGCTATTATCCTTGGGCGGACTCCCTCCCCTCTCGGGCTTTATGCCAAAATGACTAATTATTCAAGAGTTAACTAAACAAGATCTAGCCCTAGTTGCGACCTTAGCCGCCCTCTCTGCGCTACTCAGCCTTTTCTTTTACTTACGCATTTGTTACTCCCTCACATTTACCTCCTCTCCTAATAATCTCATGGGAACACCCCCCTGACGACTAATAACAAAGCAAGTATCACTTCCCCTGGCTATAACAACCTCCCTCTCTATTCTTCTACTCCCGGTTACCCCTGCAATCTTATCAGTGGTTCTCCCTTTGTAAAGAGGCTTAGGATAGTATTAAGACCAAGGGCCTTCAAAGCCCTAAGCGGGAGTGAAAGCCCCCCAGCCTCTGTAAGACCTACGGGACACTAACCCACATCTTCTGTATGCAAAACAGACACTTTAATTAAGCTAAAGCCTTCCTAGGTGGGTAGGCCTCGATCCTACAATCTCTTAGTTAACAGCTAAGCGCCTAAACCAGCGGGCATCCATCTACCTTTCCCCCGCCTTGCCGAAAAAAAAAGGCGGGGGAAAGCCCCGGCAGGGTATTAGCCTGCTACTTAAGATTTGCAATCTAATGTGTTAACACCTCGGAGCTGGTAAGAAGAGGACTTTAACCTCTGTCTATGGGGCTACAATCCACCGCTAAACGCTCAGCCACCTTACCTGTGGCAATCACACGTTGATTTTTCTCAACTAATCACAAAGACATCGGCACCCTATATCTAATCTTTGGTGCCTGGGCGGGAATAGTAGGGACGGCCTTAAGTCTACTCATTCGGGCAGAATTAAGTCAACCAGGCTCCCTATTAGGAGACGACCAGATCTATAACGTAATTGTAACTGCACATGCTTTCGTAATAATTTTCTTTATAGTAATGCCAATCATAATTGGAGGATTTGGTAACTGATTAATTCCTTTAATGATCGGAGCTCCCGACATGGCCTTCCCCCGGATAAATAATATAAGCTTTTGACTCCTGCCCCCTTCTTTCCTTCTATTATTGGCCTCATCTGGTGTAGAAGCTGGTGCCGGGACAGGATGAACCGTATATCCCCCCTTGTCCGGTAATTTGGCACACGCAGGGGCCTCCGTAGATTTAACCATTTTCTCTCTGCACCTGGCCGGAATTTCTTCTATTCTAGGGGCCATTAATTTCATTACAACTATTATTAATATAAAACCTCCAGCCATTTCCCAATATCAAACCCCTTTATTTGTCTGAGCTGTTCTAATTACCGCAGTATTACTCCTACTCTCTCTTCCTGTTCTAGCTGCGGGTATCACTATGCTTCTCACAGATCGAAACCTAAATACAACATTTTTCGACCCCGCAGGAGGGGGGGACCCCATTCTTTATCAACATTTATTCTGATTCTTTGGGCATCCTGAAGTCTACATTCTGATTTTGCCCGGCTTCGGAATGATTTCTCACATTGTAGCATATTACTCAGGCAAAAAAGAGCCGTTTGGCTACATGGGAATAGTATGAGCTATAATAGCAATTGGCCTACTGGGGTTTATCGTATGAGCCCATCATATGTTCACTGTAGGAATGGACGTGGACACTCGAGCTTATTTTACATCCGCCACTATAATTATCGCAATTCCTACAGGAGTCAAAGTGTTTAGTTGACTAGCTACCTTGCATGGGGGCTCAATCAAATGAGAGACCCCCCTGTTATGAGCTCTAGGCTTTATTTTCTTATTTACTGTTGGAGGTTTAACAGGAATTGTTTTAGCCAACTCATCTCTGGATATTATACTTCATGACACATACTACGTTGTAGCCCACTTCCACTATGTCCTCTCTATAGGAGCAGTCTTTGCCATCATGGGGGCATTCGTTCACTGATTCCCCCTATTCTCAGGCTACACCCTTCACAATACGTGAACAAAAATCCACTTCGGAGTTATGTTTGTAGGTGTAAACCTCACCTTTTTCCCTCAGCACTTCTTAGGATTGGCGGGAATACCTCGACGATATTCAGATTACCCTGACGCATACACACTGTGAAATACTATCTCATCTCTGGGGTCACTAATCTCCCTTATTGCTGTAATTATATTCCTATTTATTATCTGGGAAGCATTCGCGGCAAAACGTGAAGTCTTATCAGTTGAACTAACAGCCACAAACGTAGAATGACTGCACGGGTGTCCTCCCCCTTACCATACATTTGAAGAACCCGCATTCGTTCAAATTCAGCAATCCAAATTTTAATCGAGAAAGGAAGGAGTCGAACCCCCATAAACTGGTTTCAAGCCAGCCACATAACCGCTCTGTCACTTTCTTCCCTAAGTTAATAAGATTCTAGTTAAAAGAATAACGCTGCCTTGTCAAGGCAAAATTGTGGGTTAAAGCCCCACGTATCTTGCTTATGGCACATCCATCTCAACTAGGATTCCAAGATGCAGCTTCACCCGTTATAGAAGAACTTCTCCATTTTCATGACCATGCATTAATAATTGTTTTCTTAATCAGCACCCTTGTTCTTTACATTATTGTGGCTATGGTAACCACCAAGCTAACAAATAAGTTCATTCTGGACTCCCAAGAAATTGAAATCATCTGAACCTTACTACCAGCAATTATCCTAATTCTGATCGCCCTGCCCTCCCTTCGCATTCTCTACCTCATGGATGAAATCAATGACCCCCACCTCACAATTAAAGCCATAGGACATCAATGATACTGAAGCTACGAATATACGGATTATGAAGACCTGGGGTTCGACTCATATATGGTCCCTACACAAGATCTCGCCCCTGGTCAATTTCGACTACTTGAGACAGACCACCGCATGGTTATTCCTGTTGAGTCTCCCATCCGAGTTCTTGTCTCCGCCGAGGATGTCTTACACTCATGAGCCGTCCCGAGCCTCGGAGTAAAAATGGACGCCGTCCCCGGCCGCCTAAATCAAACAGCCTTCATTACTTCCCGCCCAGGTGTGTTTTATGGACAATGCTCAGAAATTTGCGGAGCTAATCATAGCTTTATACCCATTGTAGTGGAGGCTGTTCCTCTAGAACACTTCGAGAACTGGTCTTACCTAATACTTCAAGATGCCTCACCAGGAAGCTAAAAGGGAATAGCATTAGCCTTTTAAGCTAAAAATTGGTGACTCCCGCCCACCCCTGGTGACATGCCTCAGTTGAACCCCGCACCCTGATTTGCTATTATAGTATTCTCGTGACTAGTTTTCCTAGCCGTTATTCCACCTAAAGTTCTAGCTCACCATTTTCCCAATGACCCCGCCCCACAAAGCGTAAAAAAATCAAAAACAGAGACTTGACCCTGACCATGACTTTAAGCCTCTTTGATCAATTTATGAGCCCTACACTTCTAGGGGTGCCTCTTATCGGACTCGCCCTAACATTACCATGAGTCCTTTACTTCCGACCCGGTGCCCGATGACTTAATAACCGCTTGATTACCCTTCAATCTATATTCATGAACTGGTTTGTAAAACAAATCTTTCAGCCAATAAGCTTAGGCGGACACAAATGGGCTGCTCTCCTCATATCTTTAATACTATTTTTAATCACCTTAAATATGCTAGGCCTGCTGCCTTACACATTTACTCCAACAACGCAGCTGTCACTTAATATAGCCTTTGCAGTTCCACTTTGACTAGCAACTGTCATTATTGGAATACGAAACCAGCCAACACATGCCCTTGGTCACCTTCTCCCCGAAGGGACTCCTACCGCCCTAATCCCGGTTTTAATCGTGATTGAAACAATTAGCCTTTTTATTCGACCCTTGGCCCTCGGTGTTCGACTTACCGCAAACTTGACAGCCGGACACCTTCTAATTCAACTAATTGCAACTGCGGCTTTTGTTCTTTTCCCTATAATACCTACAGTAGCTACTCTTACCTCTGTCTTACTATTCTTGCTAACCCTGCTAGAAGTCGCCGTGGCCATAATCCAAGCCTATGTATTTGTACTTCTTTTAAGCCTTTATCTACAAGAAAACGTCTAATGGCCCATCAAGCACATGCATATCATATAGTTGACCCAAGCCCTTGACCCCTCACAGGCGCAGTAGCCGCCCTTCTACTTACGTCTGGAACAGCAATCTGAATACACTTTAACTCCACAGTTCTCATGTCCCTTGGACTTGTCCTGCTACTATTAACCATATATCAATGATGGCGGGACATTATCCGAGAGGGAACCTTTCAAGGTCATCATACACCCCCTGTTCAAAAGGGCCTTCGGTACGGGATAATTCTATTTATTACCTCAGAGGTCTTCTTTTTCCTAGGTTTCTTCTGAGCATTTTATCACTCAAGCCTAGCCCCAACCCCCGAACTTGGCGGGTGTTGACCACCTATGGGTATTACAACACTGGACCCCTTTGAAGTCCCCCTTCTCAATACTGCTGTCCTTCTCGCCTCCGGTGTCACGGTCACTTGAGCTCACCATAGTATTATGGAGGGGCAGCGAAAACAAGCAATTCAGTCCTTAACACTCACAATTCTCCTGGGGTTCTACTTTACGTTCCTTCAAGCAATAGAGTACTACGAGGCACCCTTCACCATTGCAGATGGCGTCTATGGCTCTACATTTTTTGTGGCAACGGGGTTTCATGGCCTCCATGTAATTATTGGATCAACATTCCTGGCAGTCTGCCTCTTACGACAAGTCCAATTCCACTTTACATCAGAACATCACTTCGGATTTGAAGCTGCAGCATGATACTGACACTTTGTAGACGTAGTCTGACTATTCTTATATATCTCTATCTACTGATGAGGCTCATATCTTTCTAGTATTAAAAAGTACAAGTGACTTCCAATCACTCAGTCTTGGTTAGACTCCAAGGAAAGATAATGAACTTAGTACTAGTCATTATTTGCATCTCATTAGCCCTCGCCGCACTGCTCGCAACTGTTTCATTTTTCCTCCCACAAATAACCCCTGATTATGAGAAACTCTCACCGTATGAGTGCGGCTTTGATCCAGTGGGGTCCGCCCGTTTGCCATTCTCCATTCGCTTTTTTCTAGTCGCAATCCTATTTCTCCTCTTCGACTTAGAAATTGCCTTACTCCTTCCCCTTCCCTGAGGGGACCAACTCCCCTCCCCTCTGACAACTTTCTTCTGAGCTTCTGCTATCCTTATGCTACTAACTCTAGGGTTAATCTATGAATGACTTCAAGGGGGCCTAGAATGGGCAGAATAGGTACTTAGTTTAATAAAAACATTTGATTTCGGCTCAAAAACTTATGGTTTAAGTCCATATTTACCTGATGACCTTAACTCACTATGCATTCTCGTCAGCCTACTTTGTCAGCTTCATGGGTCTAATTTTTTACCGAAAGCATCTTCTCTCCGCCTTACTTTGCTTAGAAGCCATAATACTTATTCTTTTTATTTCACTATGCCTGTGAGGCCTAGTCTTAGCCTCAAGTGCATTTTCGGCAGGCCCAATGATCTTACTTGCTTTCTCAGCATGTGAAGCAAGTGCAGGCCTAGCACTGCTTGTAGCAATAGCTCGAACCCACGGGACTGACCGTTTAAAAAACCTAAGCCTACTCCAATGTTAATAATTCTTATTCCTACTGTTATGCTTCTACCCACAATCTGACTAAGCCCCGCTAAATACCTGTGGTCCTCAACACTTGGCCATAGCATAATAATTGCTCTTATAAGCCTCTCCTGACTTAGCCTCCCAGGGGAGGTTGGCTGATCTTCCCTTAACACTTTTATAGCAACAGACCCTCTCTCTACCCCCCTTCTCGTACTTACTTGCTGACTTCTACCCTTAATAATTCTTGCGAGCCAAAACCATATAGCCCAAGAACCTACCAATCGCCAGCGAACCTATATCTCTTTACTTACTTCCCTTCAAATCTTCTTAATCTTAGCATTTGGAGCAACCGAGATAATTATGTTCTACATTATATTTGAAGCGACCTTAATTCCCACACTCGTAATTATCACACGATGAGGAAACCAAACAGAGCGATTAAACGCAGGTATTTACTTTTTATTTTATACCTTAGCTGGCTCTTTACCACTACTAGTGGCACTTCTTCTACTTCAGACCTCGACAGGAACTCTTTCTTTTCTAACCACTCAATTTTTTTCCCCCTTACAACTGCATACAGAAGCAAGTAAGTTCTGGTGGGCGGGCTGTTTACTAGCATTCTTAGTAAAAATACCGCTATATGGGGCACACCTTTGACTTCCAAAAGCTCACGTCGAAGCCCCCATCGCCGGGTCAATAGTCCTTGCAGCCGTTCTTTTAAAACTAGGGGGTTACGGTATGATACGAGTCATTATTATCTTAGACCCCTTAACGAAACAACTCAGCTACCCCTTTATTGTTCTTGCCCTGTGGGGCGTTGTAATAACTGGCTCAATCTGCCTCCGACAAACAGACCTTAAATCACTAATCGCTTACTCCTCAGTAAGCCACATAGGCCTTGTTGCAGCAGGCATCCTGATCCAAACTCCTTGGGGGTTTACAGGAGCATTAATCCTTATAATTGCCCATGGCTTAACTTCCTCCGCCCTATTCTGTTTAGCCAACACTAACTATGAGCGAACACATAGCCGAACCATGCTTTTAGCCCGGGGCCTACAAATGGTCCTTCCTCTCTTAGCAACTTGATGGTTTCTATTTACCCTCGCCAACCTAGCACTCCCCCCGCTACCCAACCTCATAGGAGAACTTATGATTATCTCATCCCTGTATAACTGGTCAAACTGGTCTTTAGTCCTGACCGGGGCGGGAGTACTAATTACCGCTAGCTACTCTCTTCATATATTCCTAACCACTCAACGCGGCCCTATTACTAACCCCGTCTTGGCAATTGAACCAACCCACACACGAGAACATCTCCTCATAATTCTTCACCTTCTTCCCCTCCTCCTTCTAATTTTAAAACCCTGCTTGATCTGGGGCTGAACAGTTTGTAGGCGTAGTTTAAATAAAGCGCTAGATTGTGATTCTAGAAATAAGAGTTAAACCCTCTTCACCCACCGAGAGGGGTCGCCGTGACAGCAAGAACTGCTAATTCTAGCCCCTTTGGTTAAAATCCGAAGCCCACTCGAACAGGCTTCTAAAGGATAATAGCTCATCCGTTGGTCTTAGGAACCAAAAACTCTTGGTGCAACTCCAAGTAGCAGCTATGCACTTTACAACAATGATTCTCTCCTCAAGCCTAATAACAATTTTTCTTCTTCTCATCCTTCCAGTCCTAGGTACACTAAACCCTAACCCCACGGGGGGCCTGTGAGCCACAAAAAACGTTAAAACGGCAGTTAAGATAGCCTTTTTTGTAAGTCTTTTGCCTCTTTTTATCTTTCTTAATGAAGGGGTAGAGACTGTTATAACAAACTGAAAATGAATAAATACTCTAATGTTTGAAATTAATATCAGCTTTAAATTTGACCTCTACTCCGTAGTGTTTACCCCTGTGGCCCTCTACGTAACATGATCAATTTTAGAGTTCGCATCTTGGTATATACACAGTGACCCCAACATAAACCGGTTCTTTAAATATCTTCTAATCTTTCTCATTGCTATGGTCGTTCTGGTCACAGCCAACAACATGTTCCAACTATTTATTGGCTGAGAAGGTGTTGGAATTATGTCTTTCTTACTTATTGGCTGGTGATTCGGGCGGGCTGACGCCAACACTGCAGCCCTCCAGGCCGTAGTTTATAACCGAGTTGGTGATATCGGCCTGATTCTAGCAATAGCATGAATAGTAGTAAACCTAAACTCATGAGAAATACAACAGCTCTTTTCTATGTCTAAAGGCCATGATATAACCCTTCCTTTATTAGGCCTAGTACTGGCCGCTACCGGAAAGTCCGCCCAGTTTGGACTTCACCCCTGGCTCCCGTCAGCCATAGAGGGTCCAACACCGGTCTCTGCCCTCCTGCACTCTAGCACCATGGTTGTTGCTGGTATTTTTCTTCTTATTCGCCTCAGCCCCTTAATGCAAGAAAGCCCGTTAATTCTCTCAACATGCCTTTGCCTGGGGGCCCTAACTACCGTCTTTACTGCAACATGTGCCCTTACCCAAAATGACATTAAAAAAATTGTTGCATTTTCTACATCAAGTCAATTAGGACTAATAATAGTTACCATCGGACTAGGCCAGCCCCAGCTCGCCTTTCTTCATATCTGCACCCACGCCTTCTTTAAAGCAATACTTTTCTTATGTTCCGGCTCCATTATTCATAGCCTTAATGATGAGCAAGATATCCGAAAAATAGGCGGGCTTCACAAGGTGCTTCCACTGACCTCTTCTTGTCTAACCATTGGCAGCCTAGCTCTAACAGGAGTCCCCTTTTTAGCAGGCTTCTTTTCCAAAGACGCCATCATTGAAGCTATAAATACATCCTACCTTAACGCCTGAGCCCTAATTTTAACGCTTCTAGCTACATCATTTACCGCAGTTTACAGTCTCCGAGTCGTATTCTTTGCCTCTATGGGCCACCCGCGTTTTAATCCAATCTCCCCAATTAATGAAAATAACCCTACAGTGATAAACCCCCTTAAACGACTCGCTTGGGGAAGCATTTTGGCAGGGTTGCTAATTACGACCAATATTGTTCCACTTAAAACCCCCGTTTTAACCATGCCTTTCACCTTAAAAATGGCCGCACTGACTGTAACAATCATAGGACTACTCACAGCCTTAGAACTAGCGTCTCTCACGTCCCAACAATTTAAAATCAAACCCTTATCTTCTACTCACCACTTCTCAAATATATTAGGATTTTTCCCCAGTGTAGTCCATCGACTAGTCCCAAAAACTGGCCTGATTCTTGGACAACTAGTTGCCAATCAGACAGTTGACCAAACCTGACTAGAGAAAACCGGGCCAAAAATAGTAACCTCCGTTAACCTTCCAATAGCTACTTCAATTAGCAGCCTGCAGCAGGGTGTAATTAAAACCTACTTCTTATTATTTTTCTTCACCATAATACTGGCAATTCTCATCCTTGTCGTCTAACTGCCCGTAAGGTCCCCCGACTTAGCCCCCGAGTTAACTCTAGAACTACAAAAAGCGTCAGTAATAAAACCCATCCCCCAAGCATTAAAACCCCTCCTCCTGAAGAATATATCAAAGCAACCCCACCAAAATCCCCCCGAAAGAGCATGAATTCACTAAATTCGTCAGCAGTTATCCATGAACCCTCATACCAGCCCTCGGAGAAAAAAACAGAGATAGACGCGACCAGAAACACATATACTGACATAAGAAGCAAAACGGGTCAACTTCCCCACCCCTCAGGATAAGGCTCCGAAGCCAGCGCTGCTGAGTACGCAAACACAACTAACATCCCACCTAAATAAATCAAAAACAAAATCAGAGATAAAAATGAACCCCCATGCCCTACTAAAATGCCACAGCCCATTCCTGCTACTGTAACAAGCCCCAAAGCAGCAAAGTAAGGTGACGGGTTCGAGGCCACGGCCGCTAGACCTAAAACCAAACCAACTAATAATAAATAAGTCATATAAACCATAATTCTTGCCAGGATTTTAACCAGGGCCTGCGACTTGAAAAACCACCGTTGTACTCAACTACAAGAACCTAATGGCCAATCTTCGAAAAACCCATCCCCTATTAAAAATCGCAAACGATGCCCTCGTTGATCTCCCAGCCCCATCGAACATTTCAGTTTGATGAAACTTCGGATCTCTTCTAGGACTTTGTTTGGCCGCCCAGATCGTTACGGGCCTTTTCCTTGCAATACATTATACATCAGACATTGCCACAGCATTTTCATCTGTCGCACATATTTGTCGTGATGTTAACTACGGCTGACTAATCCGAAACATGCATGCAAACGGTGCTTCCTTTTTCTTCATTTGCATCTACCTGCACATCGGACGGGGCTTGTATTATGGATCATACTTATATAAAGAGACATGAAATGTAGGTGTTGTCCTTCTCCTCCTTGTGATAATGACTGCTTTCGTAGGCTACGTCCTACCCTGAGGACAAATATCATTCTGAGGGGCTACCGTCATTACCAACCTTTTATCAGCCATCCCCTACATTGGAAACGCCCTAGTTCAATGGATCTGAGGCGGATTTTCAGTAGACAACGCCACCCTTACCCGGTTCTTTGCCTTCCATTTCCTCCTCCCCTTTGTAATTGCTGCTGCTACAGTTGTACATCTTATTTTCCTGCACGAGACAGGGTCGAATAACCCAACGGGTTTAAACTCAGACTCTGACAAAGTGTCTTTTCACCCCTACTTTTCTTATAAAGATCTTCTAGGCTTTGCTGCCCTGCTAGTAGCCCTTATCTCTTTAGCCCTCTTCTCCCCTAATCTACTTGGAGACCCTGACAACTTTACCCCTGCTAATCCTTTAGTAACTCCACCTCACATCAAACCTGAGTGATACTTCCTGTTCGCTTACGCCATTCTACGATCCATCCCAAACAAGCTTGGCGGGGTTCTAGCCCTATTAGCCTCTATTCTAGTTCTCTTTCTTGTCCCTATCCTGCACACATCAAAACAACGAAGCCTAACATTCCGACCCCTAACCCAATTCCTCTTCTGACTGCTAGTCGCCGATGTAATAATTTTAACCTGAATCGGAGGTATGCCTGTAGAACACCCTTACATTATCATTGGACAAGTCGCATCCTTCATTTATTTCTCCCTTTTTCTAGTCATGGCGCCTATGGCCGGCCTACTAGAAAACAAAGTCTTAAAATGACAATGCATTAGAAGCTCAGATGAAAGAGCACCGGTCTTGTAAGCCAGAGGTCGAAGGTTCAAGCCCTTCCTAATGCTCAGAGAGAAGGGATTCTAACCCCTGCCCCTGGCTCCCAAAGCCAGGATTCTTAGCTAAACTACTCGCTGATTTTCATACACCAGTTTTGCAATCCAGAGCGCATCACTTTTGCTACCAACGTTAAATTAACGTTGCACAAACGTTGCATCAGCACCCCATGGACACTAAATGACGCGAGGACGTTGAATAGACACCCCCTACCTCTAGCACCCTTTTAACGATTTCACTTTTTTTTTTTTTTTTGTTTAACGATTACGTTTTTTTTTGCGTTCCCGGACTCTGCCAGATTTCGACCGAAAACTGCCAGAATCCGCTCAAAATCCGCTCAAATACCAATATGTATTATCCCCATAAATGGTTTAAACCATTTTTGCCTAGTACACACTGACCATGCAAGTCAATTATATTTACCCCGCGCTCCAGGCCGCAGTACATACACCTACAGTTGGTGTATTTAGCACAAGTGTGCCTCAGCTAGTTTCAAGTCACCCACATCCTTCCTTTAATTGTTACTTAATGTAGTAAGAGCCCACCATCAGTTGATTCCTTAATGTCAACGGTTCTTGAAGGTGAGGGACAAAAATCGTGGGGGTTTCACCTCTTGAATTATTCCTGGCATTTGGCTCTACATCTCAAGGCCATACATTTCTCGTCTCTCACACTTTCACTGGCCCTGACATTGGTTAATGGTGGAGTACATACTCCTCGTTACCCCCCATGCCGGGCGTTCTTTCTAATGGACAACGGGTTTTCCTTTTTTTTTCCTTTTCACTTGGCATTTCACAGTGCATACAGACCTTGTTGACAAGGTTGAACATTTAGAAACCGGCCGCAAAGAATATTGGTGAATTATTTAAAGATATTAACAGATGAATTGCATAACTGATATCAAGAGCATAAATAACCAAATGAAACTAGGAACGTTTCTATAATATGACCCCCCGGCTTCCGCGCGTCAAACCCCCCTACCCCCCTAAACTAGTAAGAAGGCTATTATTCCTGCAAACCCCCCGGAAACAGGAAACCCCCTACTAGCATTTTAGCCCGCCCAAATTTGTGTGTATTTACATTATTTGTAATATTGCAAAA

>JWM 13

GCTAGTGTAGCTTAACTAAAGCATAACACTGAAGATGTTAAGACAAACCTTAGATTGGTTTCACGAGCACAAAAGTTTGGTCCTGACTTTACTATCAACTTTAGCTAAACTTACACATGCAAGTATCCGCAATCCCGTGAGAATGCCCTACAGTTTCCTTAAAGGAAACAAGGAGCTGGTATCAGGCTCAATTACTCCCGCCCATGACACCTTGCTTAGCCACACCCCCAAGGGAACTCAGCAGTGATAGACATTAAGCAATAAGTGAAAACTTGACTTAATTAAAGCTAAGAGAACCGGTTAAACTCGTGCCAGCCACCGCGGTTATACGAGCGGTTCGAGCTGATAGACTACGGCGTAAAGCGTGGTTAATAAGAATAAAACTAAAGTCGAATGTTTTCAAAGCTGTTATACGCACTCGAAAATTAGAAGACCAGAAACGAAAGTGACTTTAACCCTATGAACCCACGAAAACTATGAAACAAACTGGGATTAGATACCCCACTATGCATAGCTGTAAACTTTGATGAGCCATTACATTATCATCCGCCTGGGTACTACGAGCATCAGCTTAAAACCCAAAGGACTTGGCGGTGCTTTAGACCCACCTAGAGGAGCCTGTTCTAGAACCGATAACCCCCGTTAAACCTCACCCTCTCTTGTTTTTCCCGCCTATATACCGCCGTCGTCAGCTTACCCTGTGAAGGTCTAATAGTAAGCACAACCAGTTATACTCAAAACGTCAGGTCGAGGTGTAGCATATGAGAGGGGAAGAAATGGGCTACATTCCTTGTTTCAAGGAAAACGGATAACATAATGAAAGGTACGTTAGAAGGAGGATTTAGCAGTAAGCAGCAAATAGAGTGTTCTGCTGAAACTGGCCCTGAAGCGCGCACACACCGCCCGTCACTCTCCCCAACTCCGAGTTAAAAACCATATATAAACCTTTGAAGGAACAAAGGGGAGGCAAGTCGTAACATGGTAAGTGTACCGGAAGGTGCACTTGGATAAATCAGAGTATAGCTAAGAAAGAAAAGCATCTCCCTTACACCGAGAAGTCATCCGTGCAAATCGGATTACCCTGACTCTAACAAGCTAGCCCAAAACCTTAACTTAAAAATCAAATATTTCTAGTAATTAATAAACCAAACACATTAAATAAATCATTTTTCCCCCTGAGTATGGGAGACAGAAAAGGATAAAGGAGCTATAGACAAAGTACCGCAAGGGAAAGCTGAAAGAGAAATGAAACAAACCAGTAAAGAAGAACAAAGCAGAGATTAACCCTTGTACCTTTTGCATCATGAATTAGCCAGTTTAATCAAGCAAAGAGCACTGTAGTTTGAACCCCCGAAACTTAGTGAGCTACTTCAAGACAGCCTATGAAATAGGGCAAACCCGTCTCTGTGGCAAAAGAGTGGGAAGATCTTCAAGTAGAGGTGACAGACCTATCGAACTAAGTTATAGCTGGTTGCTCGTGAAATGAATAGAAGTTCAGCCTTTTGCTTTCTAAATTTCGATTTAGCACTACTTAGCCTAAATGACTAGAAAACAAAAGAGTTAGTCAAAGAGGGTACAGCCTGTTTGATAAAAGATACAACTTTACTAGGAGGATAAGAATCATAATTTTAAAGGTTTAATGCCCAGGTGGGCCTAAAAGCAGCCACCCTAATCAATAGCGTTAAAGCTTAAGCATAAAACACACCTACAATTCTGATAAATCAGTTTTAATCCCCTAAAGTTAACGAGCTATTTCATACCTTATGAAAGAAATTATGCTAGTATGAGTAATAAGAAGTTACGAACTTCTCCCTGCACACGTGTAAATCGGAACGGACAAACCACCGAATCTTAACGGCCCCAGTCAAAGAGGGGATGTCGGATAAAAAAAAGAACAAGAAACTCCCGACAAAACCACCGTTAACCCCACACCGGAGTGCTCCCTGGGAAAGACAAAAAGGGACAGAAGGAACTCGGCAAATATGCTCAAGCCTCGCCTGTTTACCAAAAACATCGCCTCTTGTAAAAGTTAAATAAGAGGTACCGCCTGCCCTGTGACTAGTAGTTTAACGGCCGCGGTATTTTGACCGTGCAAAGGTAGCGCAATCACTTGCCTTTTAAATGAAGGCCTGTATGAATGGCACGACGAGGGCTTAACTGTCTCCTCTCCCTAGTCAATGAAATTGATCTCCCCGTGCAGAAGCGGGGATAATAACATAAGACGAGAAGACCCTGTGGAGCTTTAGACTATGAGCAGACCATGTCAAGAATAACAAACAAGTAAATTAAACAGATTGGTCCCTGCTTCTCTGTCTTTGGTTGGGGCGACCGCGGGATAATAAAAAGCTCCCACGAGGATTGAGAACCCTTATCTTATAACCAAGAGCTTCTCCTCTAAGTAACAGAACATCTGACCTTAATGATCCGGCCTGGCCGATCAACGGACCGAGTTACCCCAGGGATAACAGCGCAATCCTCTTTTAGAGTCCATATCGACAAGAGGGTTTACGACCTCGATGTTGGATCAGGACATCCTAATGGTGCAGCCGCTATTAAGGGTTTGTTTGTTCAACAATTAAAGTCCTACGTGATCTGAGTTCAGACCGGAGTAATCCAGGTCAGTTTCTATCTATGACGTACTCTCTTCTAGTACGAAAGGACCGAAGAAAGAAGGCCTATGAAAAGTTATGCCTTAGTCTCACCTTATGAAGAAAACTAAATAAGACAAGAGGTTACACCCCTTAGTCATAGAAAATGACATGTTAAGGTGGCAGAGCCCGGATATTGCAAAAGACCTAAGCCCTTTCCACAGAGGTTCAATTCCTCTCCTTAACTATGTTCTCAACAATATTAAGCTTCATTATTAATCCCCTAATTGTTATGGTTTTTGTTTTGCTGGCAGTAGCCCTCTTAACCTTGGTAGAGCGTAAAGTGCTAAGCTACATGCAACTTCGTAAAGGCCCAAATGTTGTTGGCCCTTACGGCCTTTTGCAACCCTTCGCTGATGGCTTGAAACTTTTCATGAAAGAGCCCGTCCGACCCTCCACCTCCTCGCCCGCCTTATTCTTAATTACCCCTATTATAGCCCTTACCTTAGCCCTAACCCTCTGGGCCCCCCTTCCTATGCCTTTTCCCATCACCGACCTAAACTTAGGCATTTTATTTATTTTAGCACTATCGAGCCTGGCAGTATATTCTATTCTTGGCTCCGGATGGGCCTCCAATTCTAAATATGCATTGATTGGTGCTCTTCGAGCAGTCGCCCAAACCATCTCTTATGAAGTGAGCTTGGGCCTTATTCTTCTTAACACAATTGTCTTTACAGGGGGTTTTACTCTTCAAACCTTCAGCACCGCACAAGAAGCCACCTGATTACTTCTACCAGCATGACCACTAGCAGCCATGTGATATATCTCCACACTCGCGGAAACTAACCGGGCCCCTTTCGACTTAACTGAAGGAGAGTCCGAACTAGTGTCTGGCTTCAACGTAGAGTATGCCGGCGGACCTTTTGCCCTTTTTTTTCTGGCAGAATACGGTAACATTTTACTTATAAATACCCTCTCAGCAGTACTATTTCTAGGCTCTTCAACCTACCACAGCTTTCCAGAACTAACCGCGACCTTATTAATGCTTAAAGCCACCCTCCTTTCAGTCGTATTTTTATGAGTGCGAGCATCTTACCCTCGGTTTCGATACGATCAACTAATGCATTTAATTTGAAAAAACTTTTTACCTCTGACCCTAGCACTAGTTATTTGACACCTTTCTCTTCCGATCACGTTGAGCGGCCTTCCCCCTCAACTTTAACTCAGGAAATGTGCCTGAAAAAGGGTCACTTTGATAGGGTGAATAATGAGGGTTAAAGCCCCTCCATCTCCTTAGAAAGAAGGGGTTTGAACCCTACCTGAAGAGATCAAAACTCTTAGTGCTTCCACTACACCACTTCCTAGTAAAGTCAGCTAATAAAAGCTTTTGGGCCCATACCCCAAATATGTTGGTTAAAATCCTTCCTTTGCTAATGAATCCTTACGTCCTTTCAATTCTACTTATAGGTTTAGGCCTCGGCACTACAGTCACATTCGCTAGCTCACACTGACTATTAGCATGAATAGGCCTTGAAATAAATACCCTCGCCATTTTGCCGTTAATAGCACAACATCACCACCCCCGAGCCGTTGAAGCCACCACCAAGTATTTTTTAATTCAATCGACAGCCGCAGCAACCATCTTATTTGCCAGCTCAACAAACGCCTGACTTTCGGGCCAGTGGGACATCATAAGTATTAATCACCCTCTTCCAACCGTCATAATTACAGTCGCTCTGTCCTTAAAACTAGGCTTGGCCCCTCTTCACGCGTGACTTCCCGAAGTAATTCAAGGCCTGGACTTAACTACGGGCTTAATCCTCTCCACATGACAAAAACTCGCACCCTTTGCCCTCCTCGTTCAAATCTTCCCCGACACCCCCCTTCTCATCACTTCTCTAGGACTTCTTTCAATATTAGTTGGGGGATGAGGGGGTTTAAACCACACACAGCTCCGCAAAGTGCTCGCATATTCTTCGATCGCCCACTTAGGCTGAATAATAGTAATTATGCAATTCTCCACCCCCCTTACAATTCTTGCTTTATCAACATACATTGTCATAACATCATCTACTTTTCTAATCTTTAAACTCCTTAAATCCACAGATATGAACAGCCTGGCAACATCTTGAGCTAAAACCCCCTCCATTACAGCCCTAGCACCTTTAGTGCTATTATCCTTAGGCGGACTCCCTCCCCTCTCGGGCTTTATGCCAAAATGACTAATTATTCAGGAGTTAACTAAGCAAGACCTAGCCCTAGTTGCGACCTTAGCCGCCCTCTCTGCGCTACTCAGCCTTTTCTTTTACCTACGCATTTGTTACTCCCTCACATTTACCTCCTCTCCTAATAATCTCATGGGAACACCCCCCTGACGACTAGTAACAAAGCAAGTATCACTTCCCCTGGCTATAACAACCGCCCTCTCTATTCTCCTACTCCCGGTTACCCCTGCAATCTTATCAGTAGTTCTCCCCTTGTAAAGAGGCTTAGGATAGTATTAAGACCAAGGGCCTTCAAAGCCCTAAGCGGGAGTGAAAGCCCCCCAGCCTCTGTAAGACCTACGGGACACTAACCCACATCTTCTGTATGCAAAACAGACACTTTAATTAAGCTAAAGCCTTCCTAGGTGGGTAGGCCTCGATCCTACAATCTCTTAGTTAACAGCTAAGCGCCTAAACCAGCGGGCATCCATCTACCTTTCCCCCGCCTTGCCGAAAAAAAAAGGCGGGGGAAAGCCCCGGCAGGGTATAAGCCTGCTACTTAAGATTTGCAATCTAATGTGTTAACACCTCGGAGCTGGTAAGAAGAGGACTTTAACCTCTGTCTATGGGGCTACAATCCACCGCTAAACGCTCAGCCACCTTACCTGTGGCAATCACACGTTGATTTTTCTCAACTAATCACAAAGACATCGGCACCCTATATCTAATTTTTGGTGCCTGGGCGGGAATAGTAGGGACGGCCTTAAGTCTACTCATTCGGGCAGAATTAAGTCAACCAGGCTCCCTATTAGGAGACGACCAGATCTATAACGTAATTGTAACTGCACATGCTTTCGTAATAATTTTCTTTATAGTAATGCCAATTATAATTGGAGGGTTCGGCAACTGATTAATTCCTTTAATGATCGGAGCTCCCGACATGGCCTTCCCCCGGATAAATAATATAAGCTTTTGACTCCTGCCCCCTTCTTTCCTTCTATTATTGGCCTCATCTGGTGTAGAAGCTGGTGCCGGGACAGGATGAACCGTATATCCCCCCTTGTCCGGTAATTTGGCACACGCAGGGGCCTCCGTAGATTTAACCATTTTCTCTCTTCACCTGGCCGGAATCTCTTCTATTCTAGGGGCCATTAATTTCATTACAACTATTATTAATATAAAACCTCCAGCCATTTCCCAATATCAAACCCCTTTATTTGTCTGAGCTGTTCTAATTACCGCAGTATTACTCCTACTCTCTCTTCCTGTTCTAGCTGCAGGTATCACTATGCTTCTCACAGATCGAAACCTAAATACAACATTTTTCGACCCCGCAGGAGGGGGGGACCCCATTCTTTATCAACATTTATTCTGATTCTTTGGGCATCCTGAAGTCTACATTCTGATTTTGCCCGGCTTCGGAATGATTTCTCACATTGTAGCATATTACTCAGGCAAAAAAGAGCCGTTTGGCTACATGGGAATAGTATGAGCTATAATAGCAATTGGCTTACTGGGGTTTATCGTATGAGCCCATCATATGTTCACTGTAGGGATGGACGTGGACACTCGAGCTTATTTTACATCCGCCACTATAATTATCGCAATTCCTACAGGAGTCAAAGTGTTTAGTTGACTAGCTACCTTGCATGGGGGCTCAATCAAATGAGAGACCCCTCTGTTATGAGCTCTAGGCTTTATTTTCTTATTTACTGTCGGAGGTTTAACAGGAATTGTTTTAGCCAACTCATCTCTGGACATTATACTTCATGACACATACTATGTTGTAGCCCACTTCCACTATGTCCTCTCTATAGGAGCAGTCTTTGCCATCATGGGGGCATTCGTTCACTGATTTCCCCTATTCTCAGGCTACACCCTTCACAATACGTGAACAAAAATCCACTTCGGAGTTATGTTTGTAGGTGTAAACCTCACCTTTTTCCCTCAGCACTTCTTAGGATTGGCGGGAATACCTCGACGATATTCAGATTACCCTGACGCATACACACTGTGAAATACTATCTCATCCCTGGGGTCACTAATCTCCCTTATTGCTGTAATTATATTCCTATTTATTATCTGGGAAGCATTCGCGGCAAAACGTGAAGTCTTATCAGTTGAACTAACAGCCACAAACGTAGAATGACTGCACGGGTGTCCTCCCCCTTACCATACATTTGAAGAACCTGCATTCGTTCAAATTCAGCAATCCAAATTTTAATCGAGAAAGGAAGGAGTCGAACCCCCATAAACTGGTTTCAAGCCAACCACATAACCGCTCTGTCACTTTCTTCTCTAAGTTAATAAGATTCTAGTTAAAAGAATAACGCTGCCTTGTCAAGGCAAAATTGTGGGTTAAAGCCCCACGTATCTTGCTTATGGCACATCCATCTCAACTAGGATTCCAAGATGCAGCTTCACCCGTTATAGAAGAACTTCTCCATTTTCATGACCATGCATTAATAATTGTTTTCTTAATCAGCACCCTTGTTCTTTACATTATTGTGGCTATGGTAACCACCGAGCTAACAAATAAGTTCATTCTGGACTCCCAAGAAATTGAAATCATCTGAACCTTACTACCAGCAATTATCCTAATTCTGATCGCCCTACCCTCCCTTCGCATTCTCTACCTCATGGATGAAATCAATGACCCCCACCTCACAATTAAAGCCATGGGACATCAATGATACTGAAGCTACGAATATACGGATTATGAAGACCTGGGGTTCGACTCATATATGGTCCCTACACAAGATCTCGCCCCTGGTCAATTTCGACTACTTGAGACAGACCATCGCATGGTTATTCCTGTTGAGTCCCCCATCCGAGTTCTTGTCTCCGCCGAGGATGTCTTACATTCATGAGCCGTCCCGAGCCTCGGAGTAAAAATGGACGCCGTCCCCGGCCGCCTAAATCAAACAGCCTTCATTACTTCCCGACCAGGTGTGTTTTATGGACAATGCTCAGAGATTTGCGGAGCTAATCATAGCTTTATACCCATTGTAGTGGAAGCTGTTCCTCTAGAACACTTCGAGAACTGGTCTTACCTAATACTTCAAGATGCCTCACCAGGAAGCTAAAAGGGAATAGCATTAGCCTTTTAAGCTAAAAATTGGTGACTCCCGCCCACCCCTGGTGACATGCCTCAGTTGAACCCCGCACCCTGATTTGCTATTATAGTATTCTCGTGACTAGTTTTCCTAGCCGTTATTCCACCTAAAGTTCTAGCTCACCATTTTCCCAATGACCCCGCCCCACAGAGCGTAAAAAAATCAAAAACAGAGACTTGACCCTGACCATGACTTTAAGCCTCTTTGATCAATTTATGAGCCCTACACTTCTAGGGGTGCCTCTTATCGGACTCGCCCTAACATTGCCATGAGTCCTTTACTTCCGACCCGGTGCCCGATGGCTTAATAACCGCTTGATTACCCTTCAATCTATATTCATAAACTGGTTTGTAAAACAAATCTTTCAACCAATAAGCTTAGGCGGACACAAATGGGCCGCTCTCCTCATATCTTTAATACTATTTTTAATTACCTTAAATATGCTGGGCCTGCTGCCTTACACATTTACTCCAACAACGCAGCTGTCACTTAATATAGCCTTTGCAGTTCCACTTTGACTAGCAACTGTCATTATTGGAATACGAAACCAGCCAACACATGCCCTTGGTCACCTTCTCCCTGAAGGAACTCCTACCGCCCTAATCCCGGTTTTAATCGTGATTGAAACAATTAGCCTTTTTATTCGACCCTTGGCCCTCGGTGTTCGACTTACCGCAAACTTGACAGCCGGACACCTTCTAATTCAACTAATTGCAACTGCGGCTTTTATTCTTTTCCCTATAATACCTACAGTAGCTGCTCTTACCTCTGTCTTACTATTCTTGCTAACCCTGCTAGAAGTCGCCGTGGCCATAATCCAAGCCTATGTATTTGTACTTCTTTTAAGCCTTTATCTACAAGAAAACGTCTAATGGCCCATCAAGCACATGCATATCATATAGTTGACCCAAGCCCTTGACCCCTCACAGGCGCAGTAGCCGCCCTTCTACTTACGTCTGGAACAGCAATCTGAATACACTTTAACTCCACAGTTCTCATGTCCCTTGGACTTGTCCTGCTACTACTAACCATATATCAATGATGGCGAGACATTATCCGAGAGGGTACCTTTCAAGGTCATCATACACCCCCTGTTCAAAAGGGCCTTCGGTACGGGATAATTCTATTTATTACCTCAGAGGTCTTCTTTTTCCTAGGTTTCTTCTGAGCATTTTATCACTCAAGCCTAGCCCCAACCCCCGAACTTGGTGGGTGTTGACCACCTATGGGTATTACAACACTGGACCCCTTTGAAGTCCCCCTTCTCAATACTGCTGTCCTTCTCGCCTCCGGTGTCACGGTCACTTGAGCTCACCATAGTATTATGGAGGGGCAGCGAAAACAAGCAATTCAATCCTTAACACTCACAATTCTACTGGGGTTCTACTTTACATTCCTTCAAGCAATAGAGTACTACGAGGCACCCTTCACCATTGCAGATGGCGTCTATGGCTCTACATTTTTTGTGGCAACGGGGTTTCATGGCCTCCATGTAATTATTGGGTCAACATTTCTGGCAGTCTGCCTCTTACGACAAGTCCAATTCCACTTTACATCAGAACATCACTTCGGATTTGAAGCTGCAGCATGATACTGACACTTTGTAGACGTAGTCTGACTATTCTTATATATCTCTATCTACTGATGAGGCTCATATCTTTCTAGTATTAAAAAGTACAAGTGACTTCCAATCACTCAGTCTTGGTTAGACTCCAAGGAAAGATAATGAACTTAGTACTAGTCATTATTTGCATCTCATTAGCCCTCGCCGCACTGCTCGCAACTGTTTCATTTTTTCTCCCACAAATAACCCCTGATTATGAGAAACTCTCACCGTATGAGTGCGGCTTTGATCCAGTGGGATCCGCCCGTTTGCCATTCTCCATTCGCTTTTTTCTAGTCGCAATCCTATTTCTCCTCTTCGACTTAGAAATTGCCTTACTTCTCCCCCTTCCCTGAGGAGACCAACTCCCCTCCCCTCTGACAACTTTCTTCTGAGCTTCTGCTATCCTTATACTACTAACTCTAGGGTTAATCTATGAATGACTTCAAGGGGGCCTAGAGTGGGCAGAATAGGTACTTAGTTTAATTAAAAACATTTGATTTCGGCTCAAAAACTTATGGTTTAAGTCCATATTTACCTGATGACCTTAACTCACTATGCATTCTCGTCAGCCTACTTTGTCAGCTTCATGGGTCTAATTTTTTACCGAAAGCATCTTCTCTCCGCCTTACTTTGCTTAGAAGCCATAATACTTATTCTTTTTATTTCACTATGCCTGTGAGGCCTAGTCTTAGCCTCAAGTGCATTTTCGGCAGGCCCAATGATCTTACTTGCTTTCTCAGCATGTGAAGCAAGTGCAGGCCTAGCACTGCTTGTAGCAATAGCTCGAACCCACGGGACTGACCGTTTAAAAAACCTTAGCCTACTCCAATGTTAATAATTCTTATTCCTACTGTTATGCTTCTACCCACAATCTGACTAAGCCCCGCTAAATACCTGTGGTCCTCAGCACTTGGCCATAGCATAATAATTGCTCTTGTAAGCCTCTCCTGACTTAGCCTCCCGGGGGAGGTTGGCTGATCTTCCCTTAACACTTTTATAGCAACAGACCCTCTCTCTACCCCCCTTCTCGTACTTACTTGCTGACTTCTACCCTTAATAATTCTTGCGAGCCAAAACCATATAGCCCAAGAACCTACCAATCGCCAGCGAACCTATATCTCTCTCCTTACTTCCCTTCAAATCTTCTTAATCTTAGCATTTGGAGCAACCGAGATAATTATGTTCTATATTATATTTGAAGCGACCTTAATTCCCACACTCGTAATTATTACACGATGAGGGAACCAAACAGAGCGATTAAACGCAGGTATTTACTTTTTATTTTATACCTTAGCCGGCTCTTTACCACTACTAGTGGCCCTCCTTCTACTTCAGACCTCGACAGGAACTCTTTCTTTTCTAACCACTCAATTTTTTCCCCCCTTACAACTGCATACAGAAGCAAGTAAATTCTGGTGGGCGGGCTGTTTACTAGCATTCTTAGTAAAAATGCCGCTATATGGGGCACACCTTTGACTTCCAAAAGCTCACGTCGAAGCCCCCATCGCCGGGTCAATAGTCCTTGCAGCCGTTCTTTTAAAACTAGGGGGTTACGGTATGATACGAGTCATTATTATCTTAGAACCCCTAACGAAACAACTCAGCTACCCCTTTATTGTTCTTGCCCTGTGGGGCGTTGTAATAACTGGCTCAATCTGCCTCCGACAAACAGACCTTAAATCACTAATCGCTTACTCCTCAGTAAGCCACATGGGCCTTGTCGCAGCAGGCATCCTGATCCAAACTCCTTGGGGGTTTACAGGAGCATTAATCCTTATAATTGCCCATGGCTTAACCTCCTCCGCCCTATTCTGTTTAGCCAACACTAACTATGAGCGAACACATAGCCGAACCATACTTTTAGCCCGGGGTCTACAAATGGTCCTTCCTCTTTTAGCAACTTGGTGGTTTCTACTTACCCTCGCCAACCTAGCACTCCCTCCGCTACCCAACCTCATAGGAGAGCTTATGATTATCTCATCCTTGTATAACTGGTCAAACTGGTCTCTAATCCTGACCGGAGCGGGACTACTAATTACCGCTAGCTACTCTCTTCATATATTCCTAACCACTCAACGCGGCCCTATTACTAACCCCGTCTTGGCAATTGAACCAACCCACACACGAGAACATCTCCTCATAATTCTTCACCTTCTTCCTCTCCTCCTTCTAATTTTAAAACCCTGCTTGATCTGGGGCTGAACAGTTTGTAGGCGTAGTTTAAATAAAGCGCTAGATTGTGATTCTAGAAATAAGAGTTAAACCCTCTTCACCCACCGAGAGGGGTCGCCGTGACAGCAAGAACTGCTAATTCTAGCCCCTTTGGTTAAAATCCAAAGCCCACTCGAACAGGCTTCTAAAGGATAACAGCTCATCCGTTGGTCTTAGGAACCAAAAACTCTTGGTGCAACTCCAAGTAGCAGCTATGCACTTTACAACAATGATTCTCTCCTCAAGCCTAATAACAATTTTCCTTCTTCTCATCCTCCCAGTCCTAGGTACACTAAACCCTAACCCCACGGGGGGCCTGTGAGCCACAAAAAACGTTAAAACGGCAGTTAAGATGGCCTTTTTTGTAAGTCTTTTGCCTCTTTTTATCTTTCTTAATGAAGGGGTAGAGACTGTTATAACAAACTGAAAATGAATAAATACTCTAATGTTTGAAATTAATATCAGCTTTAAATTTGACCTCTACTCCGTAGTATTTACCCCCGTGGCCCTCTACGTAACATGATCAATTTTAGAGTTCGCATCTTGGTATATACACAGTGATCCCAACATAAACCGATTCTTTAAATATCTTCTAATCTTTCTTATTGCTATGGTCGTTCTGGTCACAGCCAACAACATGTTCCAACTATTTATTGGCTGAGAAGGTGTTGGAATTATATCTTTCTTACTTATTGGCTGGTGATTCGGGCGGGCTGACGCCAACACTGCGGCCCTCCAGGCCGTAGTTTATAACCGAGTTGGTGATATCGGCCTTATTCTAGCAATAGCATGAATAGTAGCAAACCTAAACTCATGAGAAATACAACAGCTCTTTTCTATGTCTAAAGGCCATGATATAACCCTTCCTTTATTAGGCCTACTACTGGCCGCTACCGGAAAGTCCGCCCAATTTGGACTTCACCCCTGGCTCCCCTCAGCCATAGAGGGTCCAACACCGGTCTCTGCCCTCCTGCACTCTAGCACCATGGTGGTTGCTGGTATTTTTCTTCTTATTCGCCTCAGCCCCTTAATGCAAGAAAGCCCATTAATTCTCTCAACATGCCTTTGCCTGGGGGCCCTAACTACCGTCTTTACTGCAACATGTGCCCTTACCCAAAATGACATTAAAAAAATCGTTGCATTTTCTACATCAAGTCAATTAGGACTAATAATAGTTACCATCGGACTAGGCCAGCCCCAGCTCGCCTTTCTTCATATCTGCACCCACGCCTTCTTTAAAGCAATACTTTTCTTATGTTCCGGCTCCATCATTCATAGCCTTAATGATGAGCAAGATATCCGAAAAATAGGCGGACTTCACAAGGTGCTTCCACTGACCTCTTCTTGTCTAACCATTGGCAGCCTAGCTCTAACAGGAGTCCCCTTTTTAGCAGGCTTCTTTTCCAAAGACGCCATCATTGAAGCTATAAATACATCCTACCTTAACGCCTGAGCCCTAATTTTAACGCTTCTAGCTACATCATTCACCGCAGTTTACAGTCTCCGAGTCGTATTCTTTGCCTCTATGGGCCACCCGCGTTTTAATCCAGTCTCCCCAATTAATGAAAATAACCCTACAGTGATAAACCCCATTAAACGACTCGCTTGGGGAAGCATTTTGGCAGGGTTGCTAATTACGACCAATATTGTTCCACTTAAAACCCCCGTTTTAACCATGCCTTTCACCTTAAAAATGGCCGCACTGGCTGTAACAATTATAGGACTACTCACAGCCTTAGAACTAGCGTCTCTCACGTCCCAACAATTTAAAATCAAACCCTTATCTTCTACTCACCACTTCTCAAATATATTAGGATTTTTCCCCAGTGTAGTCCATCGACTAGTCCCAAAAACTGGCCTGATTCTAGGACAACTAGTTGCCAATCAGACAGTTGACCAAACCTGACTAGAGAAAACAGGACCAAAAATAGTAACCTCCGTTAACCTTCCAATAGCTACTTCAATTAGCAGCCTACAGCAGGGTGTAATTAAGACCTACTTCTTATTATTTTTCTTCACCATAATACTGGCAATTCTCATCTTTGTCGTCTAACTGCCCGTAAGGTCCCCCGACTTAGCCCCCGAGTTAACTCTAGAACTACAAAAAGCGTCAGTAATAAAACTCATCCCCCAAGCATTAAAACTCCTCCTCCTGAAGAATATATCAGAGCAACCCCACCGAAATCCCCCCGAAAGAGCATGAATTCACTAAACTCGTCAGCAGTTACCCATGACCCCTCATACCAGCCCTCAGAGAAAAAAACAGAGATAGACGCGACCAGGAACACATATACTGACATAAGAAGCAAAACGGGTCAACTTCCCCACCCCTCAGGATAAGGCTCCGAAGCCAGCGCTGCTGAGTACGCAAACACAACTAACATCCCACCTAAATAGATCAAAAACAAAATCAGAGATAAAAATGAACCCCCATGCCCTACTAAAATGCCACAGCCCATTCCTGCTACTGTAACAAGACCTAAAGCAGCAAAGTAGGGTGACGGGTTCGAGGCCACGGCCGCTAGACCTAAGACCAAACCAACTAATAATAAATAAGTCATATAAACCATAATTCTTGCCAGGATTTTAACCAGGGCCTGCGACTTGAAAAACCACCGTTGTACTCAACTACAAGAACCTAATGGCCAATCTTCGAAAAACCCATCCCCTATTAAAAATCGCAAACGATGCCCTCGTTGATCTCCCAGCCCCGTCGAACATTTCAGTTTGATGAAACTTCGGGTCTCTTCTAGGACTTTGTTTGGCCGCCCAAATCGTTACGGGCCTTTTCCTTGCAATACATTATACATCAGACATTGCCACAGCATTTTCATCTGTAGCACATATTTGTCGTGATGTCAACTACGGCTGACTAATCCGGAACATGCATGCAAACGGTGCTTCCTTTTTCTTCATTTGCATCTACCTGCACATCGGACGGGGCTTGTATTATGGATCATACTTATATAAAGAGACATGAAATGTAGGTGTTGTCCTTCTCCTCCTAGTGATAATGACTGCTTTCGTAGGCTACGTCCTACCCTGAGGACAAATGTCATTCTGAGGGGCCACCGTCATTACCAACCTTTTATCAGCCATTCCCTACATTGGAAACGCCCTAGTTCAATGGATCTGAGGCGGATTTTCAGTAGACAACGCCACCCTTACCCGGTTCTTTGCCTTCCATTTCCTCCTCCCCTTTGTAATTGCTGCTGCTACAGTTGTACATCTTATTTTCCTGCACGAGACAGGGTCGAATAACCCAACGGGTTTAAACTCAGACTCTGACAAAGTGTCTTTTCACCCCTACTTTTCTTATAAAGATCTTCTAGGCTTTGCTGCCCTGCTAGTAGCCCTTATCTCTTTAGCCCTCTTCTCCCCTAATCTACTTGGAGACCCTGATAACTTTACCCCTGCTAATCCTTTAGTAACTCCACCTCACATCAAACCTGAGTGATACTTCCTGTTCGCTTACGCCATTCTACGATCCATCCCAAACAAGCTTGGTGGGGTTCTAGCCCTATTAGCCTCTATTCTAGTTCTCTTTCTTGTCCCTATCCTGCACACATCAAAACAACGAAGCCTAACATTCCGACCCCTAACCCAATTCCTCTTCTGATTGCTAGTCGCCGATGTAATAATTTTAACCTGAATCGGAGGTATGCCTGTAGAACACCCTTACATTATCATTGGGCAAGTCGCATCCTTCATTTATTTTTCCCTTTTTCTAGTTATGGCGCCTATGGCCGGCCTACTAGAAAACAAAGTCTTAAAATGACAATGCATTAGAAGCTCAGATGAGAGAGCACCGGTCTTGTAAGCCAGAGGTCGAAGGTTCAAGTCCTTCCTAGTGCTCAGAGAGAAGGGATTCTAACCCCTGCCCCTGGCTCCCAAAGCCAGGATTCTTAGCTAAACTACTCGCTGATTTTCATATACCAGTTTTGCAATCCAGAGCGCATCACTTTTGCCACCAGCGTTAAATTAACGTTGCACAAACGTTGCATCAGCACCCCATGGACACTAAATGACGCGAGGGCGTTAAATAGACACCCTCTACCTCTAGCACCCTTTTAACGATTTCACTTTTTTTTTTTTTTTTGTTTAACGATTACGTTTTTTTTTGCGTTCCCGGACTCTGCCAGATTTCGACCGAAGACTGCCAGAATCCGCTCAAAATCCGCTCAAATACCAATATGTATTATCCCCATAAATGGTTTAAACCATTTTTGCCTAGTACACACTGACCATGCAAGTCAATTATATTTACCCCGCGCTCCAGGCCGCAGTACATACACCTACAGTTGGTGTATTTAGCACAAGTGTGCCTCAGCTAGTTTCAAGTCACCCACATCCTTCCTTTAATTGTTACTTAATGTAGTAAGAGCCCACCATCAGTTGATTCCTTAATGTCAACGGTTCTTGAAGGTGAGGGACAAAAATCGTGGGGGTTTCACCTCTTGAATTATTCCTGGCATTTGGCTCTACATCTCAAGGCCATACATTTCTCGTCTCTCACACTTTCACTGGCCCTGACATTGGTTAATGGTGGAGTACATACTCCTCGTTACCCCCCATGCCGGGCGTTCTTTCTAATGGACAACGGGTTTTCCTTTTTTTTTCCTTTTCACTTGGCATTTCACAGTGCATACAGACCTTGTTGACAAGGTTGAACATTTAGAAATCGGCCGCAAAGAATATTGGTGAATTATTTAAAGATATTAACAGATGAATTGCATAACTGATATCAAGAGCATAAATAACCAAATGAAACTAGGAACGTTTCTATAATATGACCCCCCGGCTTCCGCGCGTCAAACCCCCCTACCCCCCTAAACTAGTAAGAAGGCTATTATTCCTGCAAACCCCCCGGAAACAGGAAACCCCCTACTAGCATTTTAGCCCGCCCAAATTTGTGTGTATTTACATTATTTGTAATATTGCAAAA

>JWM 15

GCTAGTGTAGCTTAACTAAAGCATAACACTGAAGATGTTAAGACAAACCTTAGATTGGTTTCACGAGCACAAAAGTTTGGTCCTGACTTTACTATCAACTTTAGCTAAACTTACACATGCAAGTATCCGCAATCCCGTGAGAATGCCCTACAGTTTCCTTAAAGGAAACAAGGAGCTGGTATCAGGCTCAATTACTCCCGCCCATGACACCTTGCTTAGCCACACCCCCAAGGGAACTCAGCAGTGATAGACATTAAGCAATAAGTGAAAACTTGACTTAATTAAAGCTAAGAGAACCGGTTAAACTCGTGCCAGCCACCGCGGTTATACGAGCGGTTCGAGCTGATAGACTACGGCGTAAAGCGTGGTTAATAAGAATAAAACTAAAGTCGAATGTTTTCAAAGCTGTTATACGCACTCGAAAATTAGAAGACCAGAAACGAAAGTGACTTTAACCCTATGAACCCACGAAAACTATGAAACAAACTGGGATTAGATACCCCACTATGCATAGCTGTAAACTTTGATGAGCCATTACATTATCATCCGCCTGGGTACTACGAGCATCAGCTTAAAACCCAAAGGACTTGGCGGTGCTTTAGACCCACCTAGAGGAGCCTGTTCTAGAACCGATAACCCCCGTTAAACCTCACCCTCTCTTGTTTTTCCCGCCTATATACCGCCGTCGTCAGCTTACCCTGTGAAGGTCTAATAGTAAGCACAACCAGTTATACTCAAAACGTCAGGTCGAGGTGTAGCATATGAGAGGGGAAGAAATGGGCTACATTCCTTGTTTCAAGGAAAACGGATAACATAATGAAAGGTACGTTAGAAGGAGGATTTAGCAGTAAGCAGCAAATAGAGTGTTCTGCTGAAACTGGCCCTGAAGCGCGCACACACCGCCCGTCACTCTCCCCAACTCCGAGTTAAAAACCATATATAAACCTTTGAAGGAACAAAGGGGAGGCAAGTCGTAACATGGTAAGTGTACCGGAAGGTGCACTTGGATAAATCAGAGTATAGCTAAGAAAGAAAAGCATCTCCCTTACACCGAGAAGTCATCCGTGCAAATCGGATTACCCTGACTCTAACAAGCTAGCCCAAAACCTTAACTTAAAAATCAAATATTTCTAGTAATTAATAAACCAAACACATTAAATAAATCATTTTTCCCCCTGAGTATGGGAGACAGAAAAGGATAAAGGAGCTATAGACAAAGTACCGCAAGGGAAAGCTGAAAGAGAAATGAAATAAACCAGTAAAGAAGAACAAAGCAGAGATTAACCCTTGTACCTTTTGCATCATGAATTAGCCAGTTTAATCAAGCAAAGAGCACTGTAGTTTGAACCCCCGAAACTTAGTGAGCTACTTCAAGACAGCCTATGAAATAGGGCAAACCCGTCTCTGTGGCAAAAGAGTGGGAAGATCTTCAAGTAGAGGTGACAGACCTATCGAACTAAGTTATAGCTGGTTGCTCGTGAAATGAATAGAAGTTCAGCCTTTTGCTTTCTAAATTTCGATTTAGCACTACTTAGCCTAAATGACTAGAAAACAAAAGAGTTAGTCAAAGAGGGTACAGCCTGTTTGATAAAAGATACAACTTTACTAGGAGGATAAGAATCATAATTTTAAAGGTTTAATGCCCAGGTGGGCCTAAAAGCAGCCACCCTAATCAATAGCGTTAAAGCTTAAGCATAAAACACACCTACAATTCTGATAAATCAGTTTTAATCCCCTAAAGTTAACGAGCTATTTCATACCTTATGAAAGAAATTATGCTAGTATGAGTAATAAGAAGTTACGAACTTCTCCCTGCACACGTGTAAATCGGAACGGACAAACCACCGAATCTTAACGGCCCCAGTCAAAGAGGGGATGTCGGATAAAAAAAAGAACAAGAAACTCCCGACAAAACCACCGTTAACCCCACACCGGAGTGCTCCCTGGGAAAGACAAAAAGGGACAGAAGGAACTCGGCAAATATGCTCAAGCCTCGCCTGTTTACCAAAAACATCGCCTCTTGTAAAAGTTAAATAAGAGGTACCGCCTGCCCTGTGACTAGTAGTTTAACGGCCGCGGTATTTTGACCGTGCAAAGGTAGCGCAATCACTTGCCTTTTAAATGAAGGCCTGTATGAATGGCACGACGAGGGCTTAACTGTCTCCTCTCCCTAGTCAATGAAATTGATCTCCCCGTGCAGAAGCGGGGATAATAACATAAGACGAGAAGACCCTGTGGAGCTTTAGACTATGAGCAGACCATGTCAAGAATAACAAACAAGTAAATTAAACAGATTGGTCCCTGCTTCTCTGTCTTTGGTTGGGGCGACCGCGGGATAATAAAAAGCTCCCACGAGGATTGAGAACCCTTATCTTATAACCAAGAGCTTCTCCTCTAAGTAACAGAACATCTGACCTTAATGATCCGGCCTGGCCGATCAACGGACCGAGTTACCCCAGGGATAACAGCGCAATCCTCTTTTAGAGTCCATATCGACAAGAGGGTTTACGACCTCGATGTTGGATCAGGACATCCTAATGGTGCAGCCGCTATTAAGGGTTTGTTTGTTCAACAATTAAAGTCCTACGTGATCTGAGTTCAGACCGGAGTAATCCAGGTCAGTTTCTATCTATGACGTACTCTCTTCTAGTACGAAAGGACCGAAGAAAGAAGGCCTATGAAAAGTTATGCCTTAATCTCACCTTATGAAGAAAACTAAATAAGACAAGAGGTTACACCCCTTAGTCATAGAAAATGACATGTTAAGGTGGCAGAGCCCGGATATTGCAAAAGACCTAAGCCCTTTCCACAGAGGTTCAATTCCTCTCCTTAACTATGTTCTCAACAATATTAAGCTTCATTATTAATCCCCTAATTGTTATGGTTTTTGTTTTGCTGGCAGTAGCCCTCTTAACCTTGGTAGAGCGTAAAGTGCTAAGCTACATGCAACTTCGTAAAGGCCCAAATGTTGTTGGCCCTTACGGCCTTTTGCAACCCTTCGCTGATGGCTTGAAACTTTTCATGAAAGAGCCCGTCCGACCCTCCACCTCCTCGCCCGCCTTATTCTTAATTACCCCTATTATAGCCCTTACCTTAGCCCTAACCCTCTGGGCCCCCCTTCCTATGCCTTTTCCCATCACCGACCTAAACTTAGGCATTTTATTTATTTTAGCACTATCGAGCCTGGCAGTATATTCTATTCTTGGCTCCGGATGGGCCTCCAATTCTAAATATGCATTGATTGGTGCTCTTCGAGCAGTCGCCCAAACCATCTCTTATGAAGTGAGCTTGGGCCTTATTCTTCTTAACACAATTGTCTTTACAGGGGGTTTTACTCTTCAAACCTTCAGCACCGCACAAGAAGCCACCTGATTACTTCTACCAGCATGACCACTAGCAGCCATGTGATATATCTCCACGCTCGCGGAAACTAACCGGGCCCCTTTCGACTTAACTGAAGGAGAGTCCGAACTAGTGTCTGGCTTCAACGTAGAATATGCCGGCGGACCTTTTGCCCTTTTTTTTCTGGCAGAATACGGTAACATTTTACTTATAAATACCCTCTCAGCAGTACTATTTCTAGGCTCTTCAACCTACCACAGCTTTCCAGAACTAACCGCGACCTTATTAATGCTTAAAGCCACCCTCCTTTCAGTCGTATTTTTATGAGTGCGAGCATCTTACCCTCGGTTTCGATACGATCAACTAATGCATTTAATTTGAAAAAACTTTTTACCTCTGACCCTAGCACTAGTTATTTGACACCTTTCTCTTCCGATCACGTTGAGCGGCCTTCCCCCTCAACTTTAACTCAGGAAATGTGCCTGAAAAAGGGTCACTTTGATAGGGTGAATAATGAGGGTTAAAGCCCCTCCATCTCCTTAGAAAGAAGGGGTTTGAACCCTACCTGAAGAGATCAAAACTCTTAGTGCTTCCACTACACCACTTCCTAGTAAAGTCAGCTAATAAAAGCTTTTGGGCCCATACCCCAAATATGTTGGTTAAAATCCTTCCTTTGCTAATGAATCCTTACGTCCTTTCAATTCTACTTATAGGTTTAGGCCTCGGCACTACAGTCACATTCGCTAGCTCACACTGACTATTAGCATGAATAGGCCTTGAAATAAACACCCTCGCCATTTTGCCGTTAATAGCACAACATCACCACCCCCGAGCCGTTGAAGCCACCACCAAGTATTTTTTAATTCAATCGGCAGCCGCAGCAACCATCTTATTTGCCAGCTCAACAAACGCCTGACTTTCGGGCCAGTGGGACATCATAAGTATTAATCACCCTCTTCCAACCGTCATAATTACAGTCGCTCTGTCCTTAAAACTAGGCTTGGCCCCTCTTCACGCGTGACTTCCCGAAGTAATTCAAGGCCTGGACTTAACTACGGGCTTAATCCTCTCCACATGACAAAAACTCGCACCCTTTGCCCTCCTCGTTCAAATCTTCCCCGACACCCCCCTTCTCATCACTTCTCTAGGACTTCTTTCAATATTAGTTGGGGGATGAGGGGGTTTAAACCACACACAGCTCCGCAAAGTGCTCGCATATTCTTCGATCGCCCACTTAGGCTGAATAATAGTAATTATGCAATTCTCCACCCCCCTTACAATTCTTGCTTTATCAACATACATTGTCATAACATCATCTACTTTTCTAATCTTTAAACTCCTTAAATCCACAGATATGAACAGCCTGGCAACATCTTGAGCTAAAACCCCCTCCATTACAGCCCTAGCACCTTTAGTGCTATTATCCTTAGGCGGACTCCCTCCCCTCTCGGGCTTTATGCCAAAATGACTAATTATTCAGGAGTTAACTAAGCAAGACCTAGCCCTAGTTGCGACCTTAGCCGCCCTCTCTGCGCTACTCAGCCTTTTCTTTTACCTACGCATTTGTTACTCCCTCACATTTACCTCCTCTCCTAATAATCTCATGGGAACACCCCCCTGACGACTAGTAACAAAGCAAGTATCACTTCCCCTGGCTATAACAACCGCCCTCTCTATTCTCCTACTCCCGGTTACCCCTGCAATCTTATCAGTAGTTCTCCCCTTGTAAAGAGGCTTAGGATAGTATTAAGACCAAGGGCCTTCAAAGCCCTAAGCGGGAGTGAAAGCCCCCCAGCCTCTGTAAGACCTACGGGACACTAACCCACATCTTCTGTATGCAAAACAGACACTTTAATTAAGCTAAAGCCTTCCTAGGTGGGTAGGCCTCGATCCTACAATCTCTTAGTTAACAGCTAAGCGCCTAAACCAGCGGGCATCCATCTACCTTTCCCCCGCCTTGCCGAGAAAAAAAGGCGGGGGAAAGCCCCGGCAGGGTATTAGCCTGCTACTTAAGATTTGCAATCTAATGTGTTAACACCTCGGAGCTGGTAAGAAGAGGACTTTAACCTCTGTCTATGGGGCTACAATCCACCGCTAAACGCTCAGCCACCTTACCTGTGGCAATCACACGTTGATTTTTCTCAACTAATCACAAAGACATCGGCACCCTATATCTAATCTTTGGTGCCTGGGCGGGAATAGTAGGGACGGCCTTAAGTCTACTCATTCGGGCAGAATTAAGTCAACCAGGCTCCCTATTAGGAGACGACCAGATCTATAACGTAATTGTAACTGCACATGCTTTCGTAATAATTTTCTTTATAGTAATGCCAATTATAATTGGAGGGTTCGGCAACTGATTAATTCCTTTAATGATCGGAGCTCCCGACATGGCCTTCCCCCGGATAAATAATATAAGCTTTTGACTCCTGCCCCCTTCTTTCCTTCTATTATTGGCCTCATCTGGTGTAGAAGCTGGTGCCGGGACAGGATGAACCGTATATCCCCCCTTGTCCGGTAATTTGGCACACGCAGGGGCCTCCGTAGATTTAACCATTTTCTCTCTTCACCTGGCCGGAATCTCTTCTATTCTAGGGGCCATTAATTTCATTACAACTATTATTAATATAAAACCTCCAGCCATCTCCCAATATCAAACCCCTTTATTTGTCTGAGCTGTTCTAATTACCGCAGTATTACTCCTACTCTCTCTTCCTGTTCTAGCTGCAGGTATCACTATGCTTCTCACAGATCGAAACCTAAATACAACATTTTTCGACCCCGCAGGAGGGGGGGACCCCATTCTTTATCAACATTTATTCTGATTCTTTGGGCATCCTGAAGTCTACATTCTGATTTTGCCCGGCTTCGGAATGATTTCTCACATTGTAGCATATTACTCAGGCAAAAAAGAGCCGTTTGGCTACATGGGAATAGTATGAGCTATAATAGCAATTGGCTTACTGGGGTTTATCGTATGAGCCCATCATATGTTCACTGTAGGGATGGACGTGGACACTCGAGCTTATTTTACATCCGCCACTATAATTATCGCAATTCCTACAGGAGTTAAAGTGTTTAGTTGACTAGCTACCTTGCATGGGGGCTCAATCAAATGAGAGACCCCTCTGTTATGAGCTCTAGGCTTTATTTTCTTATTTACTGTCGGAGGTTTAACAGGAATTGTTTTAGCCAACTCATCTCTGGACATTATACTTCATGACACATACTATGTTGTAGCCCACTTCCACTATGTCCTCTCTATAGGAGCAGTCTTTGCCATCATGGGGGCATTCGTTCACTGATTCCCCCTATTCTCAGGCTACACCCTTCACAATACGTGAACAAAAATCCACTTCGGAGTTATGTTTGTAGGTGTAAACCTCACCTTTTTCCCTCAGCACTTCTTAGGATTGGCGGGAATACCTCGACGATATTCAGATTACCCTGACGCATACACACTGTGAAATACTATCTCATCCCTGGGGTCACTAATCTCCCTTATTGCTGTAATTATATTCCTATTTATTATCTGGGAAGCATTCGCGGCAAAACGTGAAGTCTTATCAGTTGAACTAACAGCCACAAACGTAGAATGACTGCACGGGTGTCCTCCCCCTTACCATACATTTGAAGAACCTGCATTCGTTCAAATTCAGCAATCCAAATTTTAATCGAGAAAGGAAGGAGTCGAACCCCCATAAACTGGTTTCAAGCCAGCCACATAACCGCTCTGTCACTTTCTTCTCTAAGTTAATAAGATTCTAGTTAAAAGAATAACGCTGCCTTGTCAAGGCAAAATTGTGGGTTAAAGCCCCACGTATCTTGCTTATGGCACATCCATCTCAACTAGGATTCCAAGATGCAGCTTCACCCGTTATAGAAGAACTTCTCCATTTTCATGACCATGCATTAATAATTGTTTTCTTAATCAGCACCCTTGTTCTTTACATTATTGTGGCTATGGTAACCACCAAGCTAACAAATAAGTTCATTCTGGACTCCCAAGAAATTGAAATCATCTGAACCTTACTACCAGCAATTATCCTAATTCTGATCGCCCTACCCTCCCTTCGCATTCTCTACCTCATGGATGAAATCAATGACCCCCACCTCACAATTAAAGCCATGGGACATCAATGATACTGAAGCTACGAATATACGGATTATGAAGACCTGGGGTTCGACTCATATATGGTCCCTACACAAGATCTCGCCCCTGGTCAATTTCGACTACTTGAGACAGACCATCGCATGGTTATTCCTGTTGAGTCCCCCATCCGAGTTCTTGTCTCCGCCGAGGATGTCTTACATTCATGAGCCGTCCCGAGCCTCGGAGTAAAAATGGACGCCGTCCCCGGCCGCCTAAATCAAACAGCCTTCATTACTTCCCGACCAGGTGTGTTTTATGGACAATGCTCAGAGATTTGCGGAGCTAATCATAGCTTTATACCCATTGTAGTGGAAGCTGTTCCTCTAGAACACTTCGAGAACTGGTCTTACCTAATACTTCAAGATGCCTCACCAGGAAGCTAAAAGGGAATAGCATTAGCCTTTTAAGCTAAAAATTGGTGACTCCCGCCCACCCCTGGTGACATGCCTCAGTTGAACCCCGCACCCTGATTTGCTATTATAGTATTCTCGTGACTAGTTTTCCTAGCCGTTATTCCACCTAAAGTTCTAGCTCACCATTTTCCCAATGACCCCGCCCCACAGAGCGTAAAAAAATCAAAAACAGAGACTTGACCCTGACCATGACTTTAAGCCTCTTTGATCAATTTATGAGCCCTACACTTCTAGGGGTGCCTCTTATCGGACTCGCCCTAACATTGCCATGAGTCCTTTACTTCCGACCCGGTGCCCGATGACTTAATAACCGCTTGATTACCCTTCAATCTATATTCATAAACTGGTTTGTAAAACAAATCTTTCAACCAATAAGCTTAGGCGGACACAAATGGGCCGCTCTCCTCATATCTTTAATACTATTTTTAATTACCTTAAATATGCTAGGCCTGCTGCCTTACACATTTACTCCAACAACGCAGCTGTCACTTAATATAGCCTTTGCAGTTCCACTTTGACTAGCAACTGTCATTATTGGAATACGAAACCAGCCAACACATGCCCTTGGTCACCTTCTCCCTGAAGGAACTCCTACCGCCCTAATCCCGGTTTTAATCGTGATTGAAACAATTAGCCTTTTTATTCGACCCTTGGCCCTCGGTGTTCGACTTACCGCAAACTTGACAGCCGGACACCTTCTAATTCAACTAATTGCAACTGCGGCCTTTGTTCTTTTCCCTATAATACCTACAGTAGCTGCTCTTACCTCTGTCTTACTATTCTTGCTAACCCTGCTAGAAGTCGCCGTGGCCATAATCCAAGCCTATGTATTTGTACTTCTTTTAAGCCTTTATCTACAAGAAAACGTCTAATGGCCCATCAAGCACATGCATATCATATAGTTGACCCAAGCCCTTGACCCCTCACAGGCGCAGTAGCCGCCCTTCTACTTACGTCTGGAACAGCAATCTGAATACACTTTAACTCCACAGTTCTCATGTCCCTTGGACTTGTCCTGCTACTACTAACCATATATCAATGATGGCGAGACATTATCCGAGAGGGTACCTTTCAAGGTCATCATACACCCCCTGTTCAAAAGGGCCTTCGGTACGGGATAATTCTATTTATTACCTCAGAGGTCTTCTTTTTCCTAGGTTTCTTCTGAGCATTTTATCACTCAAGCCTAGCCCCAACCCCCGAACTTGGTGGGTGTTGACCACCTATGGGTATTACAACACTGGACCCCTTTGAAGTCCCCCTTCTCAATACTGCTGTCCTTCTCGCCTCCGGTGTCACGGTCACTTGAGCTCACCATAGTATTATGGAGGGGCAGCGAAAACAAGCAATTCAATCCTTAACACTCACAATTCTACTGGGGTTCTACTTTACATTCCTTCAAGCAATAGAGTACTACGAGGCACCCTTCACCATTGCAGATGGCGTCTATGGCTCTACATTTTTTGTGGCAACGGGGTTTCATGGCCTCCATGTAATTATTGGGTCAACATTTCTGGCAGTCTGCCTCTTACGACAAGTCCAATTCCACTTTACATCGGAACATCACTTCGGATTTGAAGCTGCAGCATGATACTGACACTTTGTAGACGTAGTCTGACTATTCTTATATATCTCTATCTACTGATGAGGCTCATATCTTTCTAGTATTAAAAAGTACAAGTGACTTCCAATCACTCAGTCTTGGTTAGACTCCAAGGAAAGATAATGAACTTAGTACTAGTCATTATTTGCATCTCATTAGCCCTCGCCGCACTGCTCGCAACTGTTTCATTTTTTCTCCCACAAATAACCCCTGATTATGAGAAACTCTCACCGTATGAGTGCGGCTTTGATCCAGTGGGATCCGCCCGTTTGCCATTCTCCATTCGCTTTTTTCTAGTCGCAATCCTATTTCTCCTCTTCGACTTAGAAATTGCCTTACTTCTCCCCCTTCCCTGAGGAGACCAACTCCCCTCCCCTCTGACAACTTTCTTCTGAGCTTCTGCTATCCTTATACTACTAACTCTAGGGTTAATCTATGAATGACTTCAAGGGGGCCTAGAGTGGGCAGAATAGGTACTTAGTTTAATAAAAACATTTGATTTCGGCTCAAAAACTTATGGTTTAAGTCCATATTTACCTGATGACCTTAACTCACTATGCATTCTCGTCAGCCTACTTTGTCAGCTTCATGGGTCTAATTTTTTACCGAAAGCATCTTCTCTCCGCCTTACTTTGCTTAGAAGCCATAATACTTATTCTTTTTATTTCACTATGCCTGTGAGGCCTAGTCTTAGCCTCAAGTGCATTTTCGGCAGGCCCAATGATCTTACTTGCTTTCTCAGCATGTGAAGCAAGTGCAGGCCTAGCACTGCTTGTAGCAATAGCTCGAACCCACGGGACTGACCGTTTAAAAAACCTTAGCCTACTCCAATGTTAATAATTCTTATTCCTACTGTTATGCTTCTACCCACAATCTGACTAAGCCCCGCTAAATACCTGTGGTCCTCAGCACTTGGCCATAGCATAATAATTGCTCTTATAAGCCTCTCCTGATTTAGCCTCCCGGGGGAGGTTGGCTGATCTTCCCTTAACACTTTTATAGCAACAGACCCTCTCTCTACCCCCCTTCTCGTACTTACTTGCTGACTTCTACCCTTAATAATTCTTGCGAGCCAAAACCATATAGCCCAAGAACCTACCAATCGCCAGCGAACCTATATCTCTCTCCTTACTTCCCTTCAAATCTTCTTAATCTTAGCATTTGGAGCAACCGAGATAATTATGTTCTATATTATATTTGAAGCGACCTTAATTCCCACACTCGTAATTATCACACGATGAGGGAACCAAACAGAGCGATTAAACGCAGGTATTTACTTTTTATTTTATACCTTAGCCGGCTCTTTACCACTACTAGTGGCCCTCCTTCTACTTCAGACCTCGACAGGAACTCTTTCTTTTCTAACCACTCAATTTTTTCCCCCCTTACAACTGCATACAGAAGCAAGTAAATTCTGGTGGGCGGGCTGTTTACTAGCATTCTTAGTAAAAATGCCGCTATATGGGGCACACCTTTGACTTCCAAAAGCTCACGTCGAAGCCCCCATCGCCGGGTCAATAGTCCTTGCAGCCGTTCTTTTAAAACTAGGGGGTTACGGTATGATACGAGTCATTATTATCTTAGAACCCCTAACGAAACAACTCAGCTACCCCTTTATTGTTCTTGCCCTGTGGGGCGTTGTAATAACTGGCTCAATCTGCCTCCGACAAACAGACCTTAAATCACTAATCGCTTACTCCTCAGTAAGCCACATGGGCCTTGTCGCAGCAGGCATCCTGATCCAAACTCCTTGGGGGTTTACAGGAGCATTAATCCTTATAATTGCCCATGGCTTAACCTCCTCCGCCCTATTCTGTTTAGCCAACACTAACTATGAGCGAACACATAGCCGAACCATACTTTTAGCCCGGGGTCTACAAATGGTCCTTCCTCTTTTAGCAACTTGGTGGTTTCTACTTACCCTCGCCAACCTAGCACTCCCTCCGCTACCCAACCTCATAGGAGAGCTTATGATTATCTCATCCTTGTATAACTGGTCAAACTGGTCTCTAATCCTGACCGGAGCGGGAGTACTAATTACCGCTAGCTACTCTCTTCATATATTCCTAACCACTCAACGCGGCCCTATTACTAACCCCGTCTTGGCAATTGAACCAACCCACACACGAGAACATCTCCTCATAATTCTTCACCTTCTTCCTCTCCTCCTTCTAATTTTAAAACCCTGCTTGATCTGGGGCTGAACAGTTTGTAGGCGTAGTTTAAATAAAGCGCTAGATTGTGATTCTAGAAATAAGAGTTAAACCCTCTTCACCCACCGAGAGGGGTCGCCGTGACAGCAAGAACTGCTAATTCTAGCCCCTTTGGTTAAAATCCGAAGCCCACTCGAACAGGCTTCTAAAGGATAACAGCTCATCCGTTGGTCTTAGGAACCAAAAACTCTTGGTGCAACTCCAAGTAGCAGCTATGCACTTTACAACAATGATTCTCTCCTCAAGCCTAATAACAATTTTCCTTATTCTCATCCTTCCAGTCCTAGGTACACTAAACCCTAACCCCACGGGGGGCCTGTGAGCCACAAAAAACGTTAAAACGGCAGTTAAGATAGCCTTTTTTGTAAGTCTTTTGCCTCTTTTTATCTTTCTTAATGAAGGGGTAGAGACTGTTATAACAAACTGAAAATGAATAAATACTCTAATGTTTGAAATTAATATCAGCTTTAAATTTGACCTCTACTCCGTAGTATTTACCCCTGTGGCCCTCTACGTAACATGATCAATTTTAGAGTTCGCATCTTGGTATATACACAGTGATCCCAACATAAACCGATTCTTTAAATATCTTCTAATCTTTCTTATTGCTATGGTCGTTCTGGTCACAGCCAACAACATGTTCCAACTATTTATTGGCTGAGAAGGTGTTGGAATTATATCTTTCTTACTTATTGGCTGGTGATTCGGGCGGGCTGACGCCAACACTGCGGCCCTCCAGGCCGTAGTTTATAACCGAGTTGGTGATATCGGCCTTATTCTAGCAATAGCATGAATAGTAGCAAACCTAAACTCATGAGAAATACAACAGCTCTTTTCTATGTCTAAAGGCCATGATATAACCCTTCCTTTATTAGGCCTAGTACTGGCCGCTACCGGAAAGTCCGCCCAATTTGGACTTCACCCCTGGCTCCCCTCAGCCATAGAGGGTCCAACACCGGTCTCTGCCCTCCTGCATTCTAGCACCATGGTAGTTGCTGGTATTTTTCTTCTTATTCGCCTCAGCCCCTTAATGCAAGAAAGCCCATTAATTCTCTCAACATGCCTTTGCCTGGGGGCCCTAACTACCGTCTTTACTGCAACATGTGCCCTTACCCAAAATGACATTAAAAAAATCGTTGCATTTTCTACATCAAGTCAATTAGGACTAATGATAGTTACCATCGGACTAGGCCAGCCCCAGCTCGCCTTTCTTCATATCTGCACCCACGCCTTCTTTAAAGCAATACTTTTCTTATGTTCCGGCTCCATCATTCATAGCCTTAATGATGAGCAAGATATCCGAAAAATAGGCGGACTTCACAAGGTACTTCCACTGACCTCTTCTTGTCTAACCATTGGCAGCCTAGCTCTAACAGGAGTCCCCTTTTTAGCAGGCTTCTTTTCCAAAGACGCCATCATTGAAGCTATAAATACATCCTACCTTAACGCCTGAGCCCTAATTTTAACGCTTCTAGCTACATCATTCACCGCAGTTTACAGTCTCCGAGTCGTATTCTTTGCCTCTATGGGCCACCCGCGTTTTAATCCAGTCTCCCCAATTAATGAAAATAACCCTACAGTGATAAACCCCATTAAACGACTCGCTTGGGGAAGCATTTTGGCAGGGTTGCTAATTACGACCAATATTGTTCCACTTAAAACCCCCGTTTTAACCATGCCTTTCACCTTAAAAATGGCCGCACTGACTGTAACAATTATAGGACTACTCACAGCCTTAGAACTAGCGTCTCTCACGTCCCAACAATTTAAAATCAAACCCTTATCTTCTACTCACCACTTCTCAAATATATTAGGATTTTTCCCCAGTGTAGTCCATCGACTAGTCCCAAAAACTGGCCTGATTCTAGGACAACTAGTTGCCAATCAGACAGTTGACCAAACCTGACTAGAGAAAACAGGACCAAAAATAGTAACCTCCGTTAACCTTCCAATAGCTACTTCAATTAGCAGCCTACAGCAGGGTGTAATTAAGACCTACTTCTTATTATTTTTCTTCACCATAATACTGGCAATTCTCATCTTTGTCGTCTAACTGCCCGTAAGGTCCCCCGACTTAGCCCCCGAGTTAACTCTAGAACTACAAAAAGCGTCAGTAATAAAACTCATCCCCCAAGCATTAAAACTCCTCCTCCTGAAGAATATATCAGAGCAACCCCACCGAAATCCCCCCGAAAGAGCATGAATTCACTAAACTCGTCAGCAGTTACCCATGACCCCTCATACCAGCCCTCAGAGAAAAAAACAGAGATAGACGCGACCAGGAACACATACACTGACATAAGAAGCAAAACGGGTCAACTTCCCCACCCCTCAGGATAAGGCTCCGAAGCCAGCGCTGCTGAGTACGCAAACACAACTAACATCCCACCTAAATAGATCAAAAACAAAATCAGAGATAAAAATGAACCCCCATGCCCTACTAAAATGCCACAGCCCATTCCTGCTACTGTAACAAGACCTAAAGCAGCAAAGTAGGGTGACGGGTTCGAGGCCACGGCCGCTAGACCTAAGACCAAACCAACTAATAATAAATAAGTCATATAAACCATAATTCTTGCCAGGATTTTAACCAGGGCCTGCGACTTGAAAAACCACCGTTGTACTCAACTACAAGAACCTAATGGCCAATCTTCGAAAAACCCATCCCCTATTAAAAATCGCAAACGATGCCCTCGTTGATCTCCCAGCCCCGTCGAACATTTCAGTTTGATGAAACTTCGGGTCTCTTCTAGGACTTTGTTTGGCCGCCCAAATCGTTACGGGCCTTTTCCTTGCAATACATTATACATCAGACATTGCCACAGCATTTTCATCTGTAGCACATATTTGTCGTGATGTCAACTACGGCTGACTAATCCGGAACATGCATGCAAACGGTGCTTCCTTTTTCTTCATTTGCATCTACCTGCACATCGGACGGGGCTTGTATTATGGATCATACTTATATAAAGAGACATGAAATGTAGGTGTTGTCCTTCTCCTCCTAGTGATAATGACTGCTTTCGTAGGCTACGTCCTACCCTGAGGACAAATGTCATTCTGAGGGGCCACCGTCATTACCAACCTTTTATCAGCCATTCCTTACATTGGAAACGCCCTAGTTCAATGGATCTGAGGCGGATTTTCAGTAGACAACGCCACCCTTACCCGGTTCTTTGCCTTCCATTTCCTCCTCCCCTTTGTAATTGCTGCTGCTACAATTGTACATCTTATTTTCCTGCACGAGACAGGGTCGAATAACCCAACGGGTTTAAACTCAGACTCTGACAAAGTGTCTTTTCACCCCTACTTTTCTTATAAAGATCTCCTAGGCTTTGCTGCCCTGCTAGTAGCCCTTATCTCTTTAGCCCTCTTCTCCCCTAATCTACTTGGAGACCCTGATAACTTTACCCCTGCTAATCCTTTAGTAACTCCACCTCACATCAAACCTGAGTGATACTTCCTGTTCGCTTACGCCATTCTACGATCCATCCCAAACAAGCTTGGTGGGGTTCTAGCCCTATTAGCCTCTATTCTAGTTCTCTTTCTTGTCCCTATCCTGCACACATCAAAACAACGAAGCCTAACATTCCGACCCCTAACCCAGTTCCTCTTCTGATTGCTAGTCGCCGATGTAATAATTTTAACCTGAATCGGAGGTATGCCTGTAGAACACCCTTACATTATCATTGGGCAAGTCGCATCCTTCATTTATTTTTCCCTTTTTCTAGTTATGGCACCTATGGCCGGCCTACTAGAAAACAAAGTCTTAAAATGACAATGCATTAGAAGCTCAGATGAGAGAGCACCGGTCTTGTAAGCCAGAGGTCGAAGGTTCAAGTCCTTCCTAGTGCTCAGAGAGAAGGGATTCTAACCCCTGCCCCTGGCTCCCAAAGCCAGGATTCTTAGCTAAACTACTCGCTGATTTTCATATACCAGTTTTGCAATCCAGAGCGCATCACTTTTGCCACCAGCGTTAAATTAACGTTGCACAAACGTTGCATCAGCACCCCATGGACACTAAATGACGCGAGGGCGTTAAATAGACACCCTCTACCTCTAGCACCCTTTTAACGATTTCACTTTTTTTTTTTTTTTTGTTTAACGATTACGTTTTTTTTTGCGTTCCCGGACTCTGCCAGATTTCGACCGAAGACTGCCAGAATCCGCTCAAAATCCGCTCAAATACCAATATGTATTATCCCCATAAGTGGTTTAAACCATTTTTGCCTAGTACACACTGACCATGCAAGTCAATTATATTTACCCCGCGCTCCAGGCCGCAGTACATACACCTACAGTTGGTGTATTTAGCACAAGTGTGCCTCAGCTAGTTTCAAGTCACCCACATCCTTCCTTTAATTGTTACTTAATGTAGTAAGAGCCCACCATCAGTTGATTCCTTAATGTCAACGGTTCTTGAAGGTGAGGGACAAAAATCGTGGGGGTTTCACCTCTTGAATTATTCCTGGCATTTGGCTCTACATCTCAAGGCCATACATTTCTCGTCTCTCACACTTTCACTGGCCCTGACATTGGTTAATGGTGGAGTACATACTCCTCGTTACCCCCCATGCCGGGCGTTCTTTCTAATGGACAACGGGTTTTCCTTTTTTTTTCCTTTTCACTTGGCATTTCACAGTGCATACAAACCTTGTTGACAAGGTTGAACATTTAGAAATCGGCCGCAAAGAATATTGGTGAATTATTTAAAGATATTAACAGATGAATTGCATAACTGATATCAAGAGCATAAATAACCAAATGAAACTAGGAACGTTTCTATAATATGACCCCCCGGCTTCCGCGCGTCAAACCCCCCTACCCCCCTAAACTAGTAAGAAGGCTATTATTCCTGCAAACCCCCCGGAAACAGGAAACCCCCTACTAGCATTTTAGCCCGCCCAAATTTGTGTGTATTTACATTATTTGTAATATTGCAAAA

>JWM 16

GCTAGTGTAGCTTAACTAAAGCATAACACTGAAGATGTTAAGACAAACCTTAGATTGGTTTCACGAGCACAAAAGTTTGGTCCTGACTTTACTATCAACTTTAGCTAAACTTACACATGCAAGTATCCGCAATCCCGTGAGAATGCCCTACAGTTTCCTTAAAGGAAACAAGGAGCTGGTATCAGGCTCAATTACTCCCGCCCATGACACCTTGCTTAGCCACACCCCCAAGGGAACTCAGCAGTGATAGACATTAAGCAATAAGTGAAAACTTGACTTAATTAAAGCTAAGAGAACCGGTCAAACTCGTGCCAGCCACCGCGGTTATACGAGCGGTTCGAGCTGATAGACTACGGCGTAAAGCGTGGTTAATAAGAATAAAACTAAAGTCGAATGTTTTCAAAGCTGTTATACGCACTCGAAAATTAGAAGACCAGAAACGAAAGTGACTTTAACCCTATGAACCCACGAAAACTATGAAACAAACTGGGATTAGATACCCCACTATGCATAGCTGTAAACTTTGATGAACTATTACATTATCATCCGCCTGGGTACTACGAGCATTAGCTTAAAACCCAAAGGACTTGGCGGTGCTTTAGACCCACCTAGAGGAGCCTGTTCTAGAACCGATAACCCCCGTTAAACCTCACCCTCTCTTGTTTTTCCCGCCTATATACCGCCGTCGTCAGCTTACCCTGTGAAGGTCTAATAGTAAGCACAACCAGTTATACTCAAAACGTCAGGTCGAGGTGTAGCATATGAGAGGGGAAGAAATGGGCTACATTCCTTGTTTCAAGGAAAACGGATAACATAATGAAAGGTACGTTAGAAGGAGGATTTAGCAGTAAGCAGCAAATAGAGTGTTCTGCTGAAACTGGCCCTGAAGCGCGCACACACCGCCCGTCACTCTCCCCAACTCCGAGTTAAAAACCATATGTAAACCTTTGAAGGAACAAAGGGGAGGCAAGTCGTAACATGGTAAGTGTACCGGAAGGTGCACTTGGATAAATCAGAGTATAGCTAAGAAAGAAAAGCATCTCCCTTACACCGAGAAGTCATCCGTGCAAATCGGATTACCCTGACTCTAACAAGCTAGCCCAAAACCTTAACTTAAAAATCAAATATTTCTAATAATTAATAAACTAACCACATTAAATAAATCATTTTTCCCCCTGAGTATGGGAGACAGAAAAGGATAGAGGAGCTATAGACAAAGTACCGCAAGGGAAAGCTGAAAGAGAAATGAAACAAACCAGTAAAGAAGAACAAAGCAGAGATTAACCCTTGTACCTTTTGCATCATGAATTAGCCAGTTTAATCAAGCAAAGAGCACTGTAGTTTGAACCCCCGAAACTTAGTGAGCTACTTCAAGACAGCCTATGAAATAGGGCAAACCCGTCTCTGTGGCAAAAGAGTGGGAAGATCTTCAAGTAGAGGTGACAGACCTATCGAACTAAGTTATAGCTGGTTGCTCGTGAAATGAATAGAAGTTCAGCCTTTTGCTTTCTAAATTTCGATTTAGCACTACTTAGCCTAAATGACTAGAAAACAAAAGAGTTAGTCAAAGAGGGTACAGCCTGTTTGATAAAAGATACAACTTTACTAGGAGGATAAGAATCATAATTTTAAAGGTTTAATGCCCAGGTGGGCCTAAAAGCAGCCACCCTAATCAATAGCGTTAAAGCTTAAGCATAAAACGCACCTACAATTCTGATAAATCAGTTTAAATCCCCTAAGGTTAACGAGCTATTTCATACCTTATGAAAGAAATTATGCTAGTATGAGTAATAAGAAGTTACGAACTTCTCCCTGCACACGTGTAAATCGGAACGGACAAACCACCGAATCTTAACGGCCCCAGTCAAAGAGGGGATGTCGGATAAAAAAAAGAACAAGAAGCTCCCGACAAAACCACCGTTAACCCCACACCGGAGTGCTCCCTGGGAAAGACAAAAAGGGACAGAAGGAACTCGGCAAGTATGCTCAAGCCTCGCCTGTTTACCAAAAACATCGCCTCTTGTAAAAGTTAAATAAGAGGTACCGCCTGCCCTGTGACTAGTAGTTTAACGGCCGCGGTATTTTGACCGTGCAAAGGTAGCGCAATCACTTGCCTTTTAAATGAAGGCCTGTATGAATGGCACGACGAGGGCTTAACTGTCTCCTCTCCCTAGTCAATGAAATTGATCCCCCCGTGCAGAAGCGGGGATAATAACATAAGACGAGAAGACCCTGTGGAGCTTTAGACTATGAGCAGACCATGTCAAGAATAACAAACAAGTAAATTAAACAAATTGGTCCCTGCTTCTCTGTCTTTGGTTGGGGCGACCGCGGGATAATAAAAAACTCCCACGAGGATTGAGAACCCTTATCTTATAACCAAGAGCTTCTCCTCTAAGTAACAGAACATCTGACCTTAATGATCCGGCCTGGCCGATCAACGGACCGAGTTACCCCAGGGATAACAGCGCAATCCTCTTTTAGAGTCCATATCGACAAGAGGGTTTACGACCTCGATGTTGGATCAGGACATCCTAATGGTGCAGCCGCTATTAAGGGTTTGTTTGTTCAACAATTAAAGTCCTACGTGATCTGAGTTCAGACCGGAGTAATCCAGGTCAGTTTCTATCTATGACGTACTCTCTTCTAGTACGAAAGGACCGAAGAAAGAAGGCCTATGAAAAGTTATGCCTTAGTCTCACCTTATGAAGAAAACTAAATAAGACAAGAGGTTACACCCCTTAGTCATAGAAAATGACATGTTAAGGTGGCAGAGCCCGGATATTGCAAAAGACCTAAGCCCTTTCCACAGAGGTTCAATTCCTCTCCTTAACTATGTTCTCAACAATATTAAGCTTCATTATTAATCCCCTAATTGTTATGGTTTTTGTTTTGCTGGCAGTAGCCCTCTTGACCTTGGTAGAACGTAAAGTGCTAAGCTACATGCAACTTCGTAAAGGCCCAAATGTTGTTGGCCCTTACGGCCTTTTACAACCCTTCGCTGATGGCTTAAAACTTTTCATGAAAGAGCCCGTCCGACCCTCCACCTCCTCGCCCGCCTTATTCTTAATTACCCCTATTATAGCCCTTACCTTAGCCCTAACCCTCTGGGCCCCCCTTCCTATGCCTTTTCCCATCACCGACCTAAACTTAGGCATTTTATTTATTTTAGCACTATCGAGCCTGGCAGTATATTCTATTCTTGGCTCCGGATGGGCCTCCAATTCTAAATATGCACTAATTGGTGCTCTTCGAGCGGTCGCCCAAACCATCTCTTATGAGGTGAGCTTGGGCCTTATTCTTCTTAACACAATTGTCTTTACGGGGGGTTTTACTCTTCAAACCTTCAGCACCGCACAAGAAGCCACCTGATTACTTCTACCAGCTTGGCCACTAGCAGCCATGTGATATATTTCCACACTCGCGGAAACTAACCGGGCCCCTTTCGACCTAACTGAAGGAGAGTCCGAACTAGTGTCTGGCTTCAACGTAGAGTATGCCGGCGGACCTTTTGCCCTTTTTTTTCTGGCAGAATACGGTAACATTTTACTTATAAATACCCTCTCAGCAGTACTATTTCTAGGCTCTTCAACCTACCACAGCTTTCCAGAACTAACCGCGACCTTATTAATGCTTAAAGCCACCCTCCTTTCAGTCGTATTTTTATGAGTGCGAGCATCTTACCCTCGGTTTCGATACGATCAACTAATACATTTAATTTGAAAAAACTTTTTACCTCTGACCCTAGCACTAGTTATTTGACACCTTTCTCTTCCGATCACGTTGAGCGGCCTCCCCCCTCAACTTTAACTGGGGAAATGTGCCTGAAAAAGGGTCACTTTGATAGGGTGAATAATGAGGGTTAAAGCCCCTCCATCTCCTTAGAAAGAAGGGGTTTGAACCCTACCTGAAGAGATCAAAACTCTTAGTGCTTCCACTACACCACTTCCTAGTAAAGTCAGCTAATAAAAGCTTTTGGGCCCATACCCCAAATATGTTGGTTAAAATCCTTCCTTTGCTAATGAATCCTTACGTCCTTTCAATTCTACTTATAGGTTTAGGCCTCGGCACTACAGTCACATTCGCTAGCTCACACTGACTATTAGCATGAATAGGCCTTGAAATAAATACCCTCGCCATTTTGCCGTTAATAGCACAACATCACCACCCCCGAGCCGTTGAAGCCACCACCAAGTATTTTTTAATTCAATCGGCAGCCGCAGCAACCATCTTATTTGCCAGCTCAACTAACGCCTGACTTTCGGGCCAGTGGGACATCATAAGTATTAATCACCCTCTTCCAACCGTCATAATTACAGTCGCTCTGTCCTTAAAACTAGGCTTGGCCCCTCTTCACGCGTGACTTCCCGAAGTAATTCAAGGCCTAGACTTAACCACGGGCTTAATCCTCTCCACATGACAAAAACTCGCACCCTTTGCCCTCCTCGTTCAAATCTTCCCCGACACTCCCCTTCTCATCACTTCTCTAGGACTTCTTTCAATATTAGTTGGGGGGTGAGGGGGTTTAAACCACACACAACTCCGCAAAGTGCTCGCATATTCTTCGATCGCCCACTTAGGCTGAATGATAGTAATTATGCAATTCTCCACCCCCCTTACAATTCTTGCTTTATCAACATACATTGTTATAACATCATCTACTTTTCTAATCTTTAAACTCCTTAAATCCACAGATATGAACAGCCTGGCAACATCTTGAGCTAAAACCCCCTCCATTACAGCCCTGGCACCTTTAGTGCTATTATCCTTGGGCGGACTCCCTCCCCTCTCGGGCTTTATGCCAAAATGACTAATTATTCAAGAGTTAACTAAACAAGATCTAGCCCTAGTTGCGACCTTAGCCGCCCTCTCTGCGCTACTCAGCCTTTTCTTTTACTTACGCATTTGTTACTCCCTCACATTTACCTCCTCTCCTAATAATCTCATGGGAACACCCCCCTGACGACTAATAACAAAGCAAGTATCACTTCCCCTGGCTATAACAACCTCCCTCTCTATTCTTCTACTCCCGGTTACCCCTGCAATCTTATCAGTGGTTCTCCCTTTGTAAAGAGGCTTAGGATAGTATTAAGACCAAGGGCCTTCAAAGCCCTAAACGGGAGTGAAAGCCCCCCAGCCTCTGTAAGACCTACGGGACACTAACCCACATCTTCTGTATGCAAAACAGACACTTTAATTAAGCTAAAGCCTTCCTAGGTGGGTAGGCCTCGATCCTACAATCTCTTAGTTAACAGCTAAGCGCCTAAACCAGCGGGCATCCATCTACCTTTCCCCCGCCTTGCCGAAAAAAAAAGGCGGGGGAAAGCCCCGGCAGGGTATTAGCCTGCTACTTAAGATTTGCAATCTAATGTGTTAACACCTCGGAGCTGGTAAGAAGAGGACTTTAACCTCTGTCTATGGGGCTACAATCCACCGCTAAACGCTCAGCCACCTTACCTGTGGCAATCACACGTTGATTTTTCTCAACTAATCACAAAGACATCGGCACCCTATATCTAATCTTTGGTGCCTGGGCGGGAATAGTAGGGACGGCCTTAAGTCTACTCATTCGGGCAGAATTAAGTCAACCAGGCTCCCTATTAGGAGACGACCAGATCTATAACGTAATTGTAACTGCACATGCTTTCGTAATAATTTTCTTTATAGTAATGCCAATCATAATTGGAGGATTTGGTAACTGATTAATTCCTTTAATGATCGGAGCTCCCGACATGGCCTTCCCCCGGATAAATAATATAAGCTTTTGACTCCTGCCCCCTTCTTTCCTTCTATTATTGGCCTCATCTGGTGTAGAAGCTGGTGCCGGGACAGGATGAACCGTATATCCCCCCTTGTCCGGTAATTTGGCACACGCAGGGGCCTCCGTAGATTTAACCATTTTCTCTCTGCACCTGGCCGGAATTTCTTCTATTCTAGGGGCCATTAATTTCATTACAACTATTATTAATATAAAACCTCCAGCCATTTCCCAATATCAAACCCCTTTATTTGTCTGAGCTGTTCTAATTACCGCAGTATTACTCCTACTCTCTCTTCCTGTTCTAGCTGCGGGTATCACTATGCTTCTCACAGATCGAAACCTAAATACAACATTTTTCGACCCCGCAGGAGGGGGGGACCCCATTCTTTATCAACATTTATTCTGATTCTTTGGGCATCCTGAAGTCTACATTCTGATTTTGCCCGGCTTCGGAATGATTTCTCACATTGTAGCATATTACTCAGGCAAAAAAGAGCCGTTTGGCTACATGGGAATAGTATGAGCTATAATAGCAATTGGCCTACTGGGGTTTATCGTATGAGCCCATCATATGTTCACTGTAGGAATGGACGTGGACACTCGAGCTTATTTTACATCCGCCACTATAATTATCGCAATTCCTACAGGAGTCAAAGTGTTTAGTTGACTAGCTACCTTGCATGGGGGCTCAATCAAATGAGAGACCCCCCTGTTATGAGCTCTAGGCTTTATTTTCTTATTTACTGTTGGAGGTTTAACAGGAATTGTTTTAGCCAACTCATCTCTGGATATTATACTTCATGACACATACTACGTTGTAGCCCACTTCCACTATGTCCTCTCTATAGGAGCAGTCTTTGCCATCATGGGGGCATTCGTTCACTGATTCCCCCTATTCTCAGGCTACACCCTTCACAATACGTGAACAAAAATCCACTTCGGAGTTATGTTTGTAGGTGTAAACCTCACCTTTTTCCCTCAGCACTTCTTAGGATTGGCGGGAATACCTCGACGATATTCAGATTACCCTGACGCATACACACTGTGAAATACTATCTCATCTCTGGGGTCACTAATCTCCCTTATTGCTGTAATTATATTCCTATTTATTATCTGGGAAGCATTCGCGGCAAAACGTGAAGTCTTATCAGTTGAACTAACAGCCACAAACGTAGAATGACTGCACGGGTGTCCTCCCCCTTACCATACATTTGAAGAACCCGCATTCGTTCAAATTCAGCAATCCAAATTTTAATCGAGAAAGGAAGGAGTCGAACCCCCATAAACTGGTTTCAAGCCAGCCACATAACCGCTCTGTCACTTTCTTCCCTAAGTTAATAAGATTCTAGTTAAAAGAATAACGCTGCCTTGTCAAGGCAAAATTGTGGGTTAAAGCCCCACGTATCTTGCTTATGGCACATCCATCTCAACTAGGATTCCAAGATGCAGCTTCACCCGTTATAGAAGAACTTCTCCATTTTCATGACCATGCATTAATAATTGTTTTCTTAATCAGCACCCTTGTTCTTTACATTATTGTGGCTATGGTAACCACCAAGCTAACAAATAAGTTCATTCTGGACTCCCAAGAAATTGAAATCATCTGAACCTTACTACCAGCAATTATCCTAATTCTGATCGCCCTGCCCTCCCTTCGCATTCTCTACCTCATGGATGAAATCAATGACCCCCACCTCACAATTAAAGCCATAGGACATCAATGATACTGAAGCTACGAATATACGGATTATGAAGACCTGGGGTTCGACTCATATATGGTCCCTACACAAGATCTCGCCCCTGGTCAATTTCGACTACTTGAGACAGACCACCGCATGGTTATTCCTGTTGAGTCTCCCATCCGAGTTCTTGTCTCCGCCGAGGATGTCTTACACTCATGAGCCGTCCCGAGCCTCGGAGTAAAAATGGACGCCGTCCCCGGCCGCCTAAATCAAACAGCCTTCATTACTTCCCGCCCAGGTGTGTTTTATGGACAATGCTCAGAAATTTGCGGAGCTAATCATAGCTTTATACCCATTGTAGTGGAGGCTGTTCCTCTAGAACACTTCGAGAACTGGTCTTACCTAATACTTCAAGATGCCTCACCAGGAAGCTAAAAGGGAATAGCATTAGCCTTTTAAGCTAAAAATTGGTGACTCCCGCCCACCCCTGGTGACATGCCTCAGTTGAACCCCGCACCCTGATTTGCTATTATAGTATTCTCGTGACTAGTTTTCCTAGCCGTTATTCCACCTAAAGTTCTAGCTCACCATTTTCCCAATGACCCCGCCCCACAAAGCGTAAAAAAATCAAAAACAGAGACTTGACCCTGACCATGACTTTAAGCCTCTTTGATCAATTTATGAGCCCTACACTTCTAGGGGTGCCTCTTATCGGACTCGCCCTAACATTACCATGAGTCCTTTACTTCCGACCCGGTGCCCGATGACTTAATAACCGCTTGATTACCCTTCAATCTATATTCATGAACTGGTTTGTAAAACAAATCTTTCAGCCAATAAGCTTAGGCGGACACAAATGGGCTGCTCTCCTCATATCTTTAATACTATTTTTAATCACCTTAAATATGCTAGGCCTGCTGCCTTACACATTTACTCCAACAACGCAGCTGTCACTTAATATAGCCTTTGCAGTTCCACTTTGACTAGCAACTGTCATTATTGGAATACGAAACCAGCCAACACATGCCCTTGGTCACCTTCTCCCCGAAGGGACTCCTACCGCCCTAATCCCGGTTTTAATCGTGATTGAAACAATTAGCCTTTTTATTCGACCCTTGGCCCTCGGTGTTCGACTTACCGCAAACTTGACAGCCGGACACCTTCTAATTCAACTAATTGCAACTGCGGCTTTTGTTCTTTTCCCTATAATACCTACAGTAGCTACTCTTACCTCTGTCTTACTATTCTTGCTAACCCTGCTAGAAGTCGCCGTGGCCATAATCCAAGCCTATGTATTTGTACTTCTTTTAAGCCTTTATCTACAAGAAAACGTCTAATGGCCCATCAAGCACATGCATATCATATAGTTGACCCAAGCCCTTGACCCCTCACAGGCGCAGTAGCCGCCCTTCTACTTACGTCTGGAACAGCAATCTGAATACACTTTAACTCCACAGTTCTCATGTCCCTTGGACTTGTCCTGCTACTATTAACCATATATCAATGATGGCGGGACATTATCCGAGAGGGAACCTTTCAAGGTCATCATACACCCCCTGTTCAAAAGGGCCTTCGGTACGGGATAATTCTATTTATTACCTCAGAGGTCTTCTTTTTCCTAGGTTTCTTCTGAGCATTTTATCACTCAAGCCTAGCCCCAACCCCCGAACTTGGCGGGTGTTGACCACCTATGGGTATTACAACACTGGACCCCTTTGAAGTCCCCCTTCTCAATACTGCTGTCCTTCTCGCCTCCGGTGTCACGGTCACTTGAGCTCACCATAGTATTATGGAGGGGCAGCGAAAACAAGCAATTCAGTCCTTAACACTCACAATTCTCCTGGGGTTCTACTTTACGTTCCTTCAAGCAATAGAGTACTACGAGGCACCCTTCACCATTGCAGATGGCGTCTATGGCTCTACATTTTTTGTGGCAACGGGGTTTCATGGCCTCCATGTAATTATTGGATCAACATTCCTGGCAGTCTGCCTCTTACGACAAGTCCAATTCCACTTTACATCAGAACATCACTTCGGATTTGAAGCTGCAGCATGATACTGACACTTTGTAGACGTAGTCTGACTATTCTTATATATCTCTATCTACTGATGAGGCTCATATCTTTCTAGTATTAAAAAGTACAAGTGACTTCCAATCACTCAGTCTTGGTTAGACTCCAAGGAAAGATAATGAACTTAGTACTAGTCATTATTTGCATCTCATTAGCCCTCGCCGCACTGCTCGCAACTGTTTCATTTTTCCTCCCACAAATAACCCCTGATTATGAGAAACTCTCACCGTATGAGTGCGGCTTTGATCCAGTGGGGTCCGCCCGTTTGCCATTCTCCATTCGCTTTTTTCTAGTCGCAATCCTATTTCTCCTCTTCGACTTAGAAATTGCCTTACTCCTTCCCCTTCCCTGAGGGGACCAACTCCCCTCCCCTCTGACAACTTTCTTCTGAGCTTCTGCTATCCTTATGCTACTAACTCTAGGGTTAATCTATGAATGACTTCAAGGGGGCCTAGAATGGGCAGAATAGGTACTTAGTTTAATAAAAACATTTGATTTCGGCTCAAAAACTTATGGTTTAAGTCCATATTTACCTGATGACCTTAACTCACTATGCATTCTCGTCAGCCTACTTTGTCAGCTTCATGGGTCTAATTTTTTACCGAAAGCATCTTCTCTCCGCCTTACTTTGCTTAGAAGCCATAATACTTATTCTTTTTATTTCACTATGCCTGTGAGGCCTAGTCTTAGCCTCAAGTGCATTTTCGGCAGGCCCAATGATCTTACTTGCTTTCTCAGCATGTGAAGCAAGTGCAGGCCTAGCACTGCTTGTAGCAATAGCTCGAACCCACGGGACTGACCGTTTAAAAAACCTAAGCCTACTCCAATGTTAATAATTCTTATTCCTACTGTTATGCTTCTACCCACAATCTGACTAAGCCCCGCTAAATACCTGTGGTCCTCAACACTTGGCCATAGCATAATAATTGCTCTTATAAGCCTCTCCTGACTTAGCCTCCCAGGGGAGGTTGGCTGATCTTCCCTTAACACTTTTATAGCAACAGACCCTCTCTCTACCCCCCTTCTCGTACTTACTTGCTGACTTCTACCCTTAATAATTCTTGCGAGCCAAAACCATATAGCCCAAGAACCTACCAATCGCCAGCGAACCTATATCTCTTTACTTACTTCCCTTCAAATCTTCTTAATCTTAGCATTTGGAGCAACCGAGATAATTATGTTCTACATTATATTTGAAGCGACCTTAATTCCCACACTCGTAATTATCACACGATGAGGAAACCAAACAGAGCGATTAAACGCAGGTATTTACTTTTTATTTTATACCTTAGCTGGCTCTTTACCACTACTAGTGGCACTTCTTCTACTTCAGACCTCGACAGGAACTCTTTCTTTTCTAACCACTCAATTTTTTTCCCCCTTACAACTGCATACAGAAGCAAGTAAGTTCTGGTGGGCGGGCTGTTTACTAGCATTCTTAGTAAAAATACCGCTATATGGGGCACACCTTTGACTTCCAAAAGCTCACGTCGAAGCCCCCATCGCCGGGTCAATAGTCCTTGCAGCCGTTCTTTTAAAACTAGGGGGTTACGGTATGATACGAGTCATTATTATCTTAGACCCCTTAACGAAACAACTCAGCTACCCCTTTATTGTTCTTGCCCTGTGGGGCGTTGTAATAACTGGCTCAATCTGCCTCCGACAAACAGACCTTAAATCACTAATCGCTTACTCCTCAGTAAGCCACATAGGCCTTGTTGCAGCAGGCATCCTGATCCAAACTCCTTGGGGGTTTACAGGAGCATTAATCCTTATAATTGCCCATGGCTTAACTTCCTCCGCCCTATTCTGTTTAGCCAACACTAACTATGAGCGAACACATAGCCGAACCATGCTTTTAGCCCGGGGCCTACAAATGGTCCTTCCTCTCTTAGCAACTTGATGGTTTCTATTTACCCTCGCCAACCTAGCACTCCCCCCGCTACCCAACCTCATAGGAGAACTTATGATTATCTCATCCCTGTATAACTGGTCAAACTGGTCTTTAGTCCTGACCGGGGCGGGAGTACTAATTACCGCTAGCTACTCTCTTCATATATTCCTAACCACTCAACGCGGCCCTATTACTAACCCCGTCTTGGCAATTGAACCAACCCACACACGAGAACATCTCCTCATAATTCTTCACCTTCTTCCCCTCCTCCTTCTAATTTTAAAACCCTGCTTGATCTGGGGCTGAACAGTTTGTAGGCGTAGTTTAAATAAAGCGCTAGATTGTGATTCTAGAAATAAGAGTTAAACCCTCTTCACCCACCGAGAGGGGTCGCCGTGACAGCAAGAACTGCTAATTCTAGCCCCTTTGGTTAAAATCCGAAGCCCACTCGAACAGGCTTCTAAAGGATAATAGCTCATCCGTTGGTCTTAGGAACCAAAAACTCTTGGTGCAACTCCAAGTAGCAGCTATGCACTTTACAACAATGATTCTCTCCTCAAGCCTAATAACAATTTTTCTTCTTCTCATCCTTCCAGTCCTAGGTACACTAAACCCTAACCCCACGGGGGGCCTGTGAGCCACAAAAAACGTTAAAACGGCAGTTAAGATAGCCTTTTTTGTAAGTCTTTTGCCTCTTTTTATCTTTCTTAATGAAGGGGTAGAGACTGTTATAACAAACTGAAAATGAATAAATACTCTAATGTTTGAAATTAATATCAGCTTTAAATTTGACCTCTACTCCGTAGTGTTTACCCCTGTGGCCCTCTACGTAACATGATCAATTTTAGAGTTCGCATCTTGGTATATACACAGTGACCCCAACATAAACCGGTTCTTTAAATATCTTCTAATCTTTCTCATTGCTATGGTCGTTCTGGTCACAGCCAACAACATGTTCCAACTATTTATTGGCTGAGAAGGTGTTGGAATTATGTCTTTCTTACTTATTGGCTGGTGATTCGGGCGGGCTGACGCCAACACTGCAGCCCTCCAGGCCGTAGTTTATAACCGAGTTGGTGATATCGGCCTGATTCTAGCAATAGCATGAATAGTAGTAAACCTAAACTCATGAGAAATACAACAGCTCTTTTCTATGTCTAAAGGCCATGATATAACCCTTCCTTTATTAGGCCTAGTACTGGCCGCTACCGGAAAGTCCGCCCAGTTTGGACTTCACCCCTGGCTCCCGTCAGCCATAGAGGGTCCAACACCGGTCTCTGCCCTCCTGCACTCTAGCACCATGGTTGTTGCTGGTATTTTTCTTCTTATTCGCCTCAGCCCCTTAATGCAAGAAAGCCCGTTAATTCTCTCAACATGCCTTTGCCTGGGGGCCCTAACTACCGTCTTTACTGCAACATGTGCCCTTACCCAAAATGACATTAAAAAAATTGTTGCATTTTCTACATCAAGTCAATTAGGACTAATAATAGTTACCATCGGACTAGGCCAGCCCCAGCTCGCCTTTCTTCATATCTGCACCCACGCCTTCTTTAAAGCAATACTTTTCTTATGTTCCGGCTCCATTATTCATAGCCTTAATGATGAGCAAGATATCCGAAAAATAGGCGGGCTTCACAAGGTGCTTCCACTGACCTCTTCTTGTCTAACCATTGGCAGCCTAGCTCTAACAGGAGTCCCCTTTTTAGCAGGCTTCTTTTCCAAAGACGCCATCATTGAAGCTATAAATACATCCTACCTTAACGCCTGAGCCCTAATTTTAACGCTTCTAGCTACATCATTTACCGCAGTTTACAGTCTCCGAGTCGTATTCTTTGCCTCTATGGGCCACCCGCGTTTTAATCCAATCTCCCCAATTAATGAAAATAACCCTACAGTGATAAACCCCCTTAAACGACTCGCTTGGGGAAGCATTTTGGCAGGGTTGCTAATTACGACCAATATTGTTCCACTTAAAACCCCCGTTTTAACCATGCCTTTCACCTTAAAAATGGCCGCACTGACTGTAACAATCATAGGACTACTCACAGCCTTAGAACTAGCGTCTCTCACGTCCCAACAATTTAAAATCAAACCCTTATCTTCTACTCACCACTTCTCAAATATATTAGGATTTTTCCCCAGTGTAGTCCATCGACTAGTCCCAAAAACTGGCCTGATTCTTGGACAACTAGTTGCCAATCAGACAGTTGACCAAACCTGACTAGAGAAAACCGGGCCAAAAATAGTAACCTCCGTTAACCTTCCAATAGCTACTTCAATTAGCAGCCTGCAGCAGGGTGTAATTAAAACCTACTTCTTATTATTTTTCTTCACCATAATACTGGCAATTCTCATCCTTGTCGTCTAACTGCCCGTAAGGTCCCCCGACTTAGCCCCCGAGTTAACTCTAGAACTACAAAAAGCGTCAGTAATAAAACCCATCCCCCAAGCATTAAAACCCCTCCTCCTGAAGAATATATCAAAGCAACCCCACCAAAATCCCCCCGAAAGAGCATGAATTCACTAAATTCGTCAGCAGTTATCCATGAACCCTCATACCAGCCCTCGGAGAAAAAAACAGAGATAGACGCGACCAGAAACACATATACTGACATAAGAAGCAAAACGGGTCAACTTCCCCACCCCTCAGGATAAGGCTCCGAAGCCAGCGCTGCTGAGTACGCAAACACAACTAACATCCCACCTAAATAAATCAAAAACAAAATCAGAGATAAAAATGAACCCCCATGCCCTACTAAAATGCCACAGCCCATTCCTGCTACTGTAACAAGCCCCAAAGCAGCAAAGTAAGGTGACGGGTTCGAGGCCACGGCCGCTAGACCTAAAACCAAACCAACTAATAATAAATAAGTCATATAAACCATAATTCTTGCCAGGATTTTAACCAGGGCCTGCGACTTGAAAAACCACCGTTGTACTCAACTACAAGAACCTAATGGCCAATCTTCGAAAAACCCATCCCCTATTAAAAATCGCAAACGATGCCCTCGTTGATCTCCCAGCCCCATCGAACATTTCAGTTTGATGAAACTTCGGATCTCTTCTAGGACTTTGTTTGGCCGCCCAGATCGTTACGGGCCTTTTCCTTGCAATACATTATACATCAGACATTGCCACAGCATTTTCATCTGTCGCACATATTTGTCGTGATGTTAACTACGGCTGACTAATCCGAAACATGCATGCAAACGGTGCTTCCTTTTTCTTCATTTGCATCTACCTGCACATCGGACGGGGCTTGTATTATGGATCATACTTATATAAAGAGACATGAAATGTAGGTGTTGTCCTTCTCCTCCTTGTGATAATGACTGCTTTCGTAGGCTACGTCCTACCCTGAGGACAAATATCATTCTGAGGGGCTACCGTCATTACCAACCTTTTATCAGCCATCCCCTACATTGGAAACGCCCTAGTTCAATGGATCTGAGGCGGATTTTCAGTAGACAACGCCACCCTTACCCGGTTCTTTGCCTTCCATTTCCTCCTCCCCTTTGTAATTGCTGCTGCTACAGTTGTACATCTTATTTTCCTGCACGAGACAGGGTCGAATAACCCAACGGGTTTAAACTCAGACTCTGACAAAGTGTCTTTTCACCCCTACTTTTCTTATAAAGATCTTCTAGGCTTTGCTGCCCTGCTAGTAGCCCTTATCTCTTTAGCCCTCTTCTCCCCTAATCTACTTGGAGACCCTGACAACTTTACCCCTGCTAATCCTTTAGTAACTCCACCTCACATCAAACCTGAGTGATACTTCCTGTTCGCTTACGCCATTCTACGATCCATCCCAAACAAGCTTGGCGGGGTTCTAGCCCTATTAGCCTCTATTCTAGTTCTCTTTCTTGTCCCTATCCTGCACACATCAAAACAACGAAGCCTAACATTCCGACCCCTAACCCAATTCCTCTTCTGACTGCTAGTCGCCGATGTAATAATTTTAACCTGAATCGGAGGTATGCCTGTAGAACACCCTTACATTATCATTGGACAAGTCGCATCCTTCATTTATTTCTCCCTTTTTCTAGTCATGGCGCCTATGGCCGGCCTACTAGAAAACAAAGTCTTAAAATGACAATGCATTAGAAGCTCAGATGAAAGAGCACCGGTCTTGTAAGCCAGAGGTCGAAGGTTCAAGCCCTTCCTAATGCTCAGAGAGAAGGGATTCTAACCCCTGCCCCTGGCTCCCAAAGCCAGGATTCTTAGCTAAACTACTCGCTGATTTTCATACACCAGTTTTGCAATCCAGAGCGCATCACTTTTGCTACCAACGTTAAATTAACGTTGCACAAACGTTGCATCAGCACCCCATGGACACTAAATGACGCGAGGACGTTGAATAGACACCCCCTACCTCTAGCACCCTTTTAACGATTTCACTTTTTTTTTTTTTTTTGTTTAACGATTACGTTTTTTTTTGCGTTCCCGGACTCTGCCAGATTTCGACCGAAAACTGCCAGAATCCGCTCAAAATCCGCTCAAATACCAATATGTATTATCCCCATAAATGGTTTAAACCATTTTTGCCTAGTACACACTGACCATGCAAGTCAATTATATTTACCCCGCGCTCCAGGCCGCAGTACATACACCTACAGTTGGTGTATTTAGCACAAGTGTGCCTCAGCTAGTTTCAAGTCACCCACATCCTTCCTTTAATTGTTACTTAATGTAGTAAGAGCCCACCATCAGTTGATTCCTTAATGTCAACGGTTCTTGAAGGTGAGGGACAAAAATCGTGGGGGTTTCACCTCTTGAATTATTCCTGGCATTTGGCTCTACATCTCAAGGCCATACATTTCTCGTCTCTCACACTTTCACTGGCCCTGACATTGGTTAATGGTGGAGTACATACTCCTCGTTACCCCCCATGCCGGGCGTTCTTTCTAATGGACAACGGGTTTTCCTTTTTTTTTCCTTTTCACTTGGCATTTCACAGTGCATACAGACCTTGTTGACAAGGTTGAACATTTAGAAACCGGCCGCAAAGAATATTGGTGAATTATTTAAAGATATTAACAGATGAATTGCATAACTGATATCAAGAGCATAAATAACCAAATGAAACTAGGAACGTTTCTATAATATGACCCCCCGGCTTCCGCGCGTCAAACCCCCCTACCCCCCTAAACTAGTAAGAAGGCTATTATTCCTGCAAACCCCCCGGAAACAGGAAACCCCCTACTAGCATTTTAGCCCGCCCAAATTTGTGTGTATTTACATTATTTGTAATATTGCAAAA

>JWM 17

GCTAGTGTAGCTTAACTAAAGCATAACACTGAAGATGTTAAGACAAACCTTAGATTGGTTTCACGAGCACAAAAGTTTGGTCCTGACTTTACTATCAACTTTAGCTAAACTTACACATGCAAGTATCCGCAATCCCGTGAGAATGCCCTACAGTTTCCTTAAAGGAAACAAGGAGCTGGTATCAGGCTCAATTACTCCCGCCCATGACACCTTGCTTAGCCACACCCCCAAGGGAACTCAGCAGTGATAGACATTAAGCAATAAGTGAAAACTTGACTTAATTAAAGCTAAGAGAACCGGTCAAACTCGTGCCAGCCACCGCGGTTATACGAGCGGTTCGAGCTGATAGACTACGGCGTAAAGCGTGGTTAATAAGAATAAAACTAAAGTCGAATGTTTTCAAAGCTGTTATACGCACTCGAAAATTAGAAGACCAGAAACGAAAGTGACTTTAACCCTATGAACCCACGAAAACTATGAAACAAACTGGGATTAGATACCCCACTATGCATAGCTGTAAACTTTGATGAACTATTACATTATCATCCGCCTGGGTACTACGAGCATTAGCTTAAAACCCAAAGGACTTGGCGGTGCTTTAGACCCACCTAGAGGAGCCTGTTCTAGAACCGATAACCCCCGTTAAACCTCACCCTCTCTTGTTTTTCCCGCCTATATACCGCCGTCGTCAGCTTACCCTGTGAAGGTCTAATAGTAAGCACAACCAGTTATACTCAAAACGTCAGGTCGAGGTGTAGCATATGAGAGGGGAAGAAATGGGCTACATTCCTTGTTTCAAGGAAAACGGATAACATAATGAAAGGTACGTTAGAAGGAGGATTTAGCAGTAAGCAGCAAATAGAGTGTTCTGCTGAAACTGGCCCTGAAGCGCGCACACACCGCCCGTCACTCTCCCCAACTCCGAGTTAAAAACCATATGTAAACCTTTGAAGGAACAAAGGGGAGGCAAGTCGTAACATGGTAAGTGTACCGGAAGGTGCACTTGGATAAATCAGAGTATAGCTAAGAAAGAAAAGCATCTCCCTTACACCGAGAAGTCATCCGTGCAAATCGGATTACCCTGACTCTAACAAGCTAGCCCAAAACCTTAACTTAAAAATCAAATATTTCTAATAATTAATAAACTAACCACATTAAATAAATCATTTTTCCCCCTGAGTATGGGAGACAGAAAAGGATAGAGGAGCTATAGACAAAGTACCGCAAGGGAAAGCTGAAAGAGAAATGAAACAAACCAGTAAAGAAGAACAAAGCAGAGATTAACCCTTGTACCTTTTGCATCATGAATTAGCCAGTTTAATCAAGCAAAGAGCACTGTAGTTTGAACCCCCGAAACTTAGTGAGCTACTTCAAGACAGCCTATGAAATAGGGCAAACCCGTCTCTGTGGCAAAAGAGTGGGAAGATCTTCAAGTAGAGGTGACAGACCTATCGAACTAAGTTATAGCTGGTTGCTCGTGAAATGAATAGAAGTTCAGCCTTTTGCTTTCTAAATTTCGATTTAGCACTACTTAGCCTAAATGACTAGAAAACAAAAGAGTTAGTCAAAGAGGGTACAGCCTGTTTGATAAAAGATACAACTTTACTAGGAGGATAAGAATCATAATTTTAAAGGTTTAATGCCCAGGTGGGCCTAAAAGCAGCCACCCTAATCAATAGCGTTAAAGCTTAAGCATAAAACGCACCTACAATTCTGATAAATCAGTTTAAATCCCCTAAGGTTAACGAGCTATTTCATACCTTATGAAAGAAATTATGCTAGTATGAGTAATAAGAAGTTACGAACTTCTCCCTGCACACGTGTAAATCGGAACGGACAAACCACCGAATCTTAACGGCCCCAGTCAAAGAGGGGATGTCGGATAAAAAAAAGAACAAGAAGCTCCCGACAAAACCACCGTTAACCCCACACCGGAGTGCTCCCTGGGAAAGACAAAAAGGGACAGAAGGAACTCGGCAAGTATGCTCAAGCCTCGCCTGTTTACCAAAAACATCGCCTCTTGTAAAAGTTAAATAAGAGGTACCGCCTGCCCTGTGACTAGTAGTTTAACGGCCGCGGTATTTTGACCGTGCAAAGGTAGCGCAATCACTTGCCTTTTAAATGAAGGCCTGTATGAATGGCACGACGAGGGCTTAACTGTCTCCTCTCCCTAGTCAATGAAATTGATCCCCCCGTGCAGAAGCGGGGATAATAACATAAGACGAGAAGACCCTGTGGAGCTTTAGACTATGAGCAGACCATGTCAAGAATAACAAACAAGTAAATTAAACAAATTGGTCCCTGCTTCTCTGTCTTTGGTTGGGGCGACCGCGGGATAATAAAAAACTCCCACGAGGATTGAGAACCCTTATCTTATAACCAAGAGCTTCTCCTCTAAGTAACAGAACATCTGACCTTAATGATCCGGCCTGGCCGATCAACGGACCGAGTTACCCCAGGGATAACAGCGCAATCCTCTTTTAGAGTCCATATCGACAAGAGGGTTTACGACCTCGATGTTGGATCAGGACATCCTAATGGTGCAGCCGCTATTAAGGGTTTGTTTGTTCAACAATTAAAGTCCTACGTGATCTGAGTTCAGACCGGAGTAATCCAGGTCAGTTTCTATCTATGACGTACTCTCTTCTAGTACGAAAGGACCGAAGAAAGAAGGCCTATGAAAAGTTATGCCTTAGTCTCACCTTATGAAGAAAACTAAATAAGACAAGAGGTTACACCCCTTAGTCATAGAAAATGACATGTTAAGGTGGCAGAGCCCGGATATTGCAAAAGACCTAAGCCCTTTCCACAGAGGTTCAATTCCTCTCCTTAACTATGTTCTCAACAATATTAAGCTTCATTATTAATCCCCTAATTGTTATGGTTTTTGTTTTGCTGGCAGTAGCCCTCTTGACCTTGGTAGAACGTAAAGTGCTAAGCTACATGCAACTTCGTAAAGGCCCAAATGTTGTTGGCCCTTACGGCCTTTTACAACCCTTCGCTGATGGCTTAAAACTTTTCATGAAAGAGCCCGTCCGACCCTCCACCTCCTCGCCCGCCTTATTCTTAATTACCCCTATTATAGCCCTTACCTTAGCCCTAACCCTCTGGGCCCCCCTTCCTATGCCTTTTCCCATCACCGACCTAAACTTAGGCATTTTATTTATTTTAGCACTATCGAGCCTGGCAGTATATTCTATTCTTGGCTCCGGATGGGCCTCCAATTCTAAATATGCACTAATTGGTGCTCTTCGAGCGGTCGCCCAAACCATCTCTTATGAGGTGAGCTTGGGCCTTATTCTTCTTAACACAATTGTCTTTACGGGGGGTTTTACTCTTCAAACCTTCAGCACCGCACAAGAAGCCACCTGATTACTTCTACCAGCTTGGCCACTAGCAGCCATGTGATATATTTCCACACTCGCGGAAACTAACCGGGCCCCTTTCGACCTAACTGAAGGAGAGTCCGAACTAGTGTCTGGCTTCAACGTAGAGTATGCCGGCGGACCTTTTGCCCTTTTTTTTCTGGCAGAATACGGTAACATTTTACTTATAAATACCCTCTCAGCAGTACTATTTCTAGGCTCTTCAACCTACCACAGCTTTCCAGAACTAACCGCGACCTTATTAATGCTTAAAGCCACCCTCCTTTCAGTCGTATTTTTATGAGTGCGAGCATCTTACCCTCGGTTTCGATACGATCAACTAATACATTTAATTTGAAAAAACTTTTTACCTCTGACCCTAGCACTAGTTATTTGACACCTTTCTCTTCCGATCACGTTGAGCGGCCTCCCCCCTCAACTTTAACTGGGGAAATGTGCCTGAAAAAGGGTCACTTTGATAGGGTGAATAATGAGGGTTAAAGCCCCTCCATCTCCTTAGAAAGAAGGGGTTTGAACCCTACCTGAAGAGATCAAAACTCTTAGTGCTTCCACTACACCACTTCCTAGTAAAGTCAGCTAATAAAAGCTTTTGGGCCCATACCCCAAATATGTTGGTTAAAATCCTTCCTTTGCTAATGAATCCTTACGTCCTTTCAATTCTACTTATAGGTTTAGGCCTCGGCACTACAGTCACATTCGCTAGCTCACACTGACTATTAGCATGAATAGGCCTTGAAATAAATACCCTCGCCATTTTGCCGTTAATAGCACAACATCACCACCCCCGAGCCGTTGAAGCCACCACCAAGTATTTTTTAATTCAATCGGCAGCCGCAGCAACCATCTTATTTGCCAGCTCAACTAACGCCTGACTTTCGGGCCAGTGGGACATCATAAGTATTAATCACCCTCTTCCAACCGTCATAATTACAGTCGCTCTGTCCTTAAAACTAGGCTTGGCCCCTCTTCACGCGTGACTTCCCGAAGTAATTCAAGGCCTAGACTTAACCACGGGCTTAATCCTCTCCACATGACAAAAACTCGCACCCTTTGCCCTCCTCGTTCAAATCTTCCCCGACACTCCCCTTCTCATCACTTCTCTAGGACTTCTTTCAATATTAGTTGGGGGGTGAGGGGGTTTAAACCACACACAACTCCGCAAAGTGCTCGCATATTCTTCGATCGCCCACTTAGGCTGAATGATAGTAATTATGCAATTCTCCACCCCCCTTACAATTCTTGCTTTATCAACATACATTGTTATAACATCATCTACTTTTCTAATCTTTAAACTCCTTAAATCCACAGATATGAACAGCCTGGCAACATCTTGAGCTAAAACCCCCTCCATTACAGCCCTGGCACCTTTAGTGCTATTATCCTTGGGCGGACTCCCTCCCCTCTCGGGCTTTATGCCAAAATGACTAATTATTCAAGAGTTAACTAAACAAGATCTAGCCCTAGTTGCGACCTTAGCCGCCCTCTCTGCGCTACTCAGCCTTTTCTTTTACTTACGCATTTGTTACTCCCTCACATTTACCTCCTCTCCTAATAATCTCATGGGAACACCCCCCTGACGACTAATAACAAAGCAAGTATCACTTCCCCTGGCTATAACAACCTCCCTCTCTATTCTTCTACTCCCGGTTACCCCTGCAATCTTATCAGTGGTTCTCCCTTTGTAAAGAGGCTTAGGATAGTATTAAGACCAAGGGCCTTCAAAGCCCTAAACGGGAGTGAAAGCCCCCCAGCCTCTGTAAGACCTACGGGACACTAACCCACATCTTCTGTATGCAAAACAGACACTTTAATTAAGCTAAAGCCTTCCTAGGTGGGTAGGCCTCGATCCTACAATCTCTTAGTTAACAGCTAAGCGCCTAAACCAGCGGGCATCCATCTACCTTTCCCCCGCCTTGCCGAAAAAAAAAGGCGGGGGAAAGCCCCGGCAGGGTATTAGCCTGCTACTTAAGATTTGCAATCTAATGTGTTAACACCTCGGAGCTGGTAAGAAGAGGACTTTAACCTCTGTCTATGGGGCTACAATCCACCGCTAAACGCTCAGCCACCTTACCTGTGGCAATCACACGTTGATTTTTCTCAACTAATCACAAAGACATCGGCACCCTATATCTAATCTTTGGTGCCTGGGCGGGAATAGTAGGGACGGCCTTAAGTCTACTCATTCGGGCAGAATTAAGTCAACCAGGCTCCCTATTAGGAGACGACCAGATCTATAACGTAATTGTAACTGCACATGCTTTCGTAATAATTTTCTTTATAGTAATGCCAATCATAATTGGAGGATTTGGTAACTGATTAATTCCTTTAATGATCGGAGCTCCCGACATGGCCTTCCCCCGGATAAATAATATAAGCTTTTGACTCCTGCCCCCTTCTTTCCTTCTATTATTGGCCTCATCTGGTGTAGAAGCTGGTGCCGGGACAGGATGAACCGTATATCCCCCCTTGTCCGGTAATTTGGCACACGCAGGGGCCTCCGTAGATTTAACCATTTTCTCTCTGCACCTGGCCGGAATTTCTTCTATTCTAGGGGCCATTAATTTCATTACAACTATTATTAATATAAAACCTCCAGCCATTTCCCAATATCAAACCCCTTTATTTGTCTGAGCTGTTCTAATTACCGCAGTATTACTCCTACTCTCTCTTCCTGTTCTAGCTGCGGGTATCACTATGCTTCTCACAGATCGAAACCTAAATACAACATTTTTCGACCCCGCAGGAGGGGGGGACCCCATTCTTTATCAACATTTATTCTGATTCTTTGGGCATCCTGAAGTCTACATTCTGATTTTGCCCGGCTTCGGAATGATTTCTCACATTGTAGCATATTACTCAGGCAAAAAAGAGCCGTTTGGCTACATGGGAATAGTATGAGCTATAATAGCAATTGGCCTACTGGGGTTTATCGTATGAGCCCATCATATGTTCACTGTAGGAATGGACGTGGACACTCGAGCTTATTTTACATCCGCCACTATAATTATCGCAATTCCTACAGGAGTCAAAGTGTTTAGTTGACTAGCTACCTTGCATGGGGGCTCAATCAAATGAGAGACCCCCCTGTTATGAGCTCTAGGCTTTATTTTCTTATTTACTGTTGGAGGTTTAACAGGAATTGTTTTAGCCAACTCATCTCTGGATATTATACTTCATGACACATACTACGTTGTAGCCCACTTCCACTATGTCCTCTCTATAGGAGCAGTCTTTGCCATCATGGGGGCATTCGTTCACTGATTCCCCCTATTCTCAGGCTACACCCTTCACAATACGTGAACAAAAATCCACTTCGGAGTTATGTTTGTAGGTGTAAACCTCACCTTTTTCCCTCAGCACTTCTTAGGATTGGCGGGAATACCTCGACGATATTCAGATTACCCTGACGCATACACACTGTGAAATACTATCTCATCTCTGGGGTCACTAATCTCCCTTATTGCTGTAATTATATTCCTATTTATTATCTGGGAAGCATTCGCGGCAAAACGTGAAGTCTTATCAGTTGAACTAACAGCCACAAACGTAGAATGACTGCACGGGTGTCCTCCCCCTTACCATACATTTGAAGAACCCGCATTCGTTCAAATTCAGCAATCCAAATTTTAATCGAGAAAGGAAGGAGTCGAACCCCCATAAACTGGTTTCAAGCCAGCCACATAACCGCTCTGTCACTTTCTTCCCTAAGTTAATAAGATTCTAGTTAAAAGAATAACGCTGCCTTGTCAAGGCAAAATTGTGGGTTAAAGCCCCACGTATCTTGCTTATGGCACATCCATCTCAACTAGGATTCCAAGATGCAGCTTCACCCGTTATAGAAGAACTTCTCCATTTTCATGACCATGCATTAATAATTGTTTTCTTAATCAGCACCCTTGTTCTTTACATTATTGTGGCTATGGTAACCACCAAGCTAACAAATAAGTTCATTCTGGACTCCCAAGAAATTGAAATCATCTGAACCTTACTACCAGCAATTATCCTAATTCTGATCGCCCTGCCCTCCCTTCGCATTCTCTACCTCATGGATGAAATCAATGACCCCCACCTCACAATTAAAGCCATAGGACATCAATGATACTGAAGCTACGAATATACGGATTATGAAGACCTGGGGTTCGACTCATATATGGTCCCTACACAAGATCTCGCCCCTGGTCAATTTCGACTACTTGAGACAGACCACCGCATGGTTATTCCTGTTGAGTCTCCCATCCGAGTTCTTGTCTCCGCCGAGGATGTCTTACACTCATGAGCCGTCCCGAGCCTCGGAGTAAAAATGGACGCCGTCCCCGGCCGCCTAAATCAAACAGCCTTCATTACTTCCCGCCCAGGTGTGTTTTATGGACAATGCTCAGAAATTTGCGGAGCTAATCATAGCTTTATACCCATTGTAGTGGAGGCTGTTCCTCTAGAACACTTCGAGAACTGGTCTTACCTAATACTTCAAGATGCCTCACCAGGAAGCTAAAAGGGAATAGCATTAGCCTTTTAAGCTAAAAATTGGTGACTCCCGCCCACCCCTGGTGACATGCCTCAGTTGAACCCCGCACCCTGATTTGCTATTATAGTATTCTCGTGACTAGTTTTCCTAGCCGTTATTCCACCTAAAGTTCTAGCTCACCATTTTCCCAATGACCCCGCCCCACAAAGCGTAAAAAAATCAAAAACAGAGACTTGACCCTGACCATGACTTTAAGCCTCTTTGATCAATTTATGAGCCCTACACTTCTAGGGGTGCCTCTTATCGGACTCGCCCTAACATTACCATGAGTCCTTTACTTCCGACCCGGTGCCCGATGACTTAATAACCGCTTGATTACCCTTCAATCTATATTCATGAACTGGTTTGTAAAACAAATCTTTCAGCCAATAAGCTTAGGCGGACACAAATGGGCTGCTCTCCTCATATCTTTAATACTATTTTTAATCACCTTAAATATGCTAGGCCTGCTGCCTTACACATTTACTCCAACAACGCAGCTGTCACTTAATATAGCCTTTGCAGTTCCACTTTGACTAGCAACTGTCATTATTGGAATACGAAACCAGCCAACACATGCCCTTGGTCACCTTCTCCCCGAAGGGACTCCTACCGCCCTAATCCCGGTTTTAATCGTGATTGAAACAATTAGCCTTTTTATTCGACCCTTGGCCCTCGGTGTTCGACTTACCGCAAACTTGACAGCCGGACACCTTCTAATTCAACTAATTGCAACTGCGGCTTTTGTTCTTTTCCCTATAATACCTACAGTAGCTACTCTTACCTCTGTCTTACTATTCTTGCTAACCCTGCTAGAAGTCGCCGTGGCCATAATCCAAGCCTATGTATTTGTACTTCTTTTAAGCCTTTATCTACAAGAAAACGTCTAATGGCCCATCAAGCACATGCATATCATATAGTTGACCCAAGCCCTTGACCCCTCACAGGCGCAGTAGCCGCCCTTCTACTTACGTCTGGAACAGCAATCTGAATACACTTTAACTCCACAGTTCTCATGTCCCTTGGACTTGTCCTGCTACTATTAACCATATATCAATGATGGCGGGACATTATCCGAGAGGGAACCTTTCAAGGTCATCATACACCCCCTGTTCAAAAGGGCCTTCGGTACGGGATAATTCTATTTATTACCTCAGAGGTCTTCTTTTTCCTAGGTTTCTTCTGAGCATTTTATCACTCAAGCCTAGCCCCAACCCCCGAACTTGGCGGGTGTTGACCACCTATGGGTATTACAACACTGGACCCCTTTGAAGTCCCCCTTCTCAATACTGCTGTCCTTCTCGCCTCCGGTGTCACGGTCACTTGAGCTCACCATAGTATTATGGAGGGGCAGCGAAAACAAGCAATTCAGTCCTTAACACTCACAATTCTCCTGGGGTTCTACTTTACGTTCCTTCAAGCAATAGAGTACTACGAGGCACCCTTCACCATTGCAGATGGCGTCTATGGCTCTACATTTTTTGTGGCAACGGGGTTTCATGGCCTCCATGTAATTATTGGATCAACATTCCTGGCAGTCTGCCTCTTACGACAAGTCCAATTCCACTTTACATCAGAACATCACTTCGGATTTGAAGCTGCAGCATGATACTGACACTTTGTAGACGTAGTCTGACTATTCTTATATATCTCTATCTACTGATGAGGCTCATATCTTTCTAGTATTAAAAAGTACAAGTGACTTCCAATCACTCAGTCTTGGTTAGACTCCAAGGAAAGATAATGAACTTAGTACTAGTCATTATTTGCATCTCATTAGCCCTCGCCGCACTGCTCGCAACTGTTTCATTTTTCCTCCCACAAATAACCCCTGATTATGAGAAACTCTCACCGTATGAGTGCGGCTTTGATCCAGTGGGGTCCGCCCGTTTGCCATTCTCCATTCGCTTTTTTCTAGTCGCAATCCTATTTCTCCTCTTCGACTTAGAAATTGCCTTACTCCTTCCCCTTCCCTGAGGGGACCAACTCCCCTCCCCTCTGACAACTTTCTTCTGAGCTTCTGCTATCCTTATGCTACTAACTCTAGGGTTAATCTATGAATGACTTCAAGGGGGCCTAGAATGGGCAGAATAGGTACTTAGTTTAATAAAAACATTTGATTTCGGCTCAAAAACTTATGGTTTAAGTCCATATTTACCTGATGACCTTAACTCACTATGCATTCTCGTCAGCCTACTTTGTCAGCTTCATGGGTCTAATTTTTTACCGAAAGCATCTTCTCTCCGCCTTACTTTGCTTAGAAGCCATAATACTTATTCTTTTTATTTCACTATGCCTGTGAGGCCTAGTCTTAGCCTCAAGTGCATTTTCGGCAGGCCCAATGATCTTACTTGCTTTCTCAGCATGTGAAGCAAGTGCAGGCCTAGCACTGCTTGTAGCAATAGCTCGAACCCACGGGACTGACCGTTTAAAAAACCTAAGCCTACTCCAATGTTAATAATTCTTATTCCTACTGTTATGCTTCTACCCACAATCTGACTAAGCCCCGCTAAATACCTGTGGTCCTCAACACTTGGCCATAGCATAATAATTGCTCTTATAAGCCTCTCCTGACTTAGCCTCCCAGGGGAGGTTGGCTGATCTTCCCTTAACACTTTTATAGCAACAGACCCTCTCTCTACCCCCCTTCTCGTACTTACTTGCTGACTTCTACCCTTAATAATTCTTGCGAGCCAAAACCATATAGCCCAAGAACCTACCAATCGCCAGCGAACCTATATCTCTTTACTTACTTCCCTTCAAATCTTCTTAATCTTAGCATTTGGAGCAACCGAGATAATTATGTTCTACATTATATTTGAAGCGACCTTAATTCCCACACTCGTAATTATCACACGATGAGGAAACCAAACAGAGCGATTAAACGCAGGTATTTACTTTTTATTTTATACCTTAGCTGGCTCTTTACCACTACTAGTGGCACTTCTTCTACTTCAGACCTCGACAGGAACTCTTTCTTTTCTAACCACTCAATTTTTTTCCCCCTTACAACTGCATACAGAAGCAAGTAAGTTCTGGTGGGCGGGCTGTTTACTAGCATTCTTAGTAAAAATACCGCTATATGGGGCACACCTTTGACTTCCAAAAGCTCACGTCGAAGCCCCCATCGCCGGGTCAATAGTCCTTGCAGCCGTTCTTTTAAAACTAGGGGGTTACGGTATGATACGAGTCATTATTATCTTAGACCCCTTAACGAAACAACTCAGCTACCCCTTTATTGTTCTTGCCCTGTGGGGCGTTGTAATAACTGGCTCAATCTGCCTCCGACAAACAGACCTTAAATCACTAATCGCTTACTCCTCAGTAAGCCACATAGGCCTTGTTGCAGCAGGCATCCTGATCCAAACTCCTTGGGGGTTTACAGGAGCATTAATCCTTATAATTGCCCATGGCTTAACTTCCTCCGCCCTATTCTGTTTAGCCAACACTAACTATGAGCGAACACATAGCCGAACCATGCTTTTAGCCCGGGGCCTACAAATGGTCCTTCCTCTCTTAGCAACTTGATGGTTTCTATTTACCCTCGCCAACCTAGCACTCCCCCCGCTACCCAACCTCATAGGAGAACTTATGATTATCTCATCCCTGTATAACTGGTCAAACTGGTCTTTAGTCCTGACCGGGGCGGGAGTACTAATTACCGCTAGCTACTCTCTTCATATATTCCTAACCACTCAACGCGGCCCTATTACTAACCCCGTCTTGGCAATTGAACCAACCCACACACGAGAACATCTCCTCATAATTCTTCACCTTCTTCCCCTCCTCCTTCTAATTTTAAAACCCTGCTTGATCTGGGGCTGAACAGTTTGTAGGCGTAGTTTAAATAAAGCGCTAGATTGTGATTCTAGAAATAAGAGTTAAACCCTCTTCACCCACCGAGAGGGGTCGCCGTGACAGCAAGAACTGCTAATTCTAGCCCCTTTGGTTAAAATCCGAAGCCCACTCGAACAGGCTTCTAAAGGATAATAGCTCATCCGTTGGTCTTAGGAACCAAAAACTCTTGGTGCAACTCCAAGTAGCAGCTATGCACTTTACAACAATGATTCTCTCCTCAAGCCTAATAACAATTTTTCTTCTTCTCATCCTTCCAGTCCTAGGTACACTAAACCCTAACCCCACGGGGGGCCTGTGAGCCACAAAAAACGTTAAAACGGCAGTTAAGATAGCCTTTTTTGTAAGTCTTTTGCCTCTTTTTATCTTTCTTAATGAAGGGGTAGAGACTGTTATAACAAACTGAAAATGAATAAATACTCTAATGTTTGAAATTAATATCAGCTTTAAATTTGACCTCTACTCCGTAGTGTTTACCCCTGTGGCCCTCTACGTAACATGATCAATTTTAGAGTTCGCATCTTGGTATATACACAGTGACCCCAACATAAACCGGTTCTTTAAATATCTTCTAATCTTTCTCATTGCTATGGTCGTTCTGGTCACAGCCAACAACATGTTCCAACTATTTATTGGCTGAGAAGGTGTTGGAATTATGTCTTTCTTACTTATTGGCTGGTGATTCGGGCGGGCTGACGCCAACACTGCAGCCCTCCAGGCCGTAGTTTATAACCGAGTTGGTGATATCGGCCTGATTCTAGCAATAGCATGAATAGTAGTAAACCTAAACTCATGAGAAATACAACAGCTCTTTTCTATGTCTAAAGGCCATGATATAACCCTTCCTTTATTAGGCCTAGTACTGGCCGCTACCGGAAAGTCCGCCCAGTTTGGACTTCACCCCTGGCTCCCGTCAGCCATAGAGGGTCCAACACCGGTCTCTGCCCTCCTGCACTCTAGCACCATGGTTGTTGCTGGTATTTTTCTTCTTATTCGCCTCAGCCCCTTAATGCAAGAAAGCCCGTTAATTCTCTCAACATGCCTTTGCCTGGGGGCCCTAACTACCGTCTTTACTGCAACATGTGCCCTTACCCAAAATGACATTAAAAAAATTGTTGCATTTTCTACATCAAGTCAATTAGGACTAATAATAGTTACCATCGGACTAGGCCAGCCCCAGCTCGCCTTTCTTCATATCTGCACCCACGCCTTCTTTAAAGCAATACTTTTCTTATGTTCCGGCTCCATTATTCATAGCCTTAATGATGAGCAAGATATCCGAAAAATAGGCGGGCTTCACAAGGTGCTTCCACTGACCTCTTCTTGTCTAACCATTGGCAGCCTAGCTCTAACAGGAGTCCCCTTTTTAGCAGGCTTCTTTTCCAAAGACGCCATCATTGAAGCTATAAATACATCCTACCTTAACGCCTGAGCCCTAATTTTAACGCTTCTAGCTACATCATTTACCGCAGTTTACAGTCTCCGAGTCGTATTCTTTGCCTCTATGGGCCACCCGCGTTTTAATCCAATCTCCCCAATTAATGAAAATAACCCTACAGTGATAAACCCCCTTAAACGACTCGCTTGGGGAAGCATTTTGGCAGGGTTGCTAATTACGACCAATATTGTTCCACTTAAAACCCCCGTTTTAACCATGCCTTTCACCTTAAAAATGGCCGCACTGACTGTAACAATCATAGGACTACTCACAGCCTTAGAACTAGCGTCTCTCACGTCCCAACAATTTAAAATCAAACCCTTATCTTCTACTCACCACTTCTCAAATATATTAGGATTTTTCCCCAGTGTAGTCCATCGACTAGTCCCAAAAACTGGCCTGATTCTTGGACAACTAGTTGCCAATCAGACAGTTGACCAAACCTGACTAGAGAAAACCGGGCCAAAAATAGTAACCTCCGTTAACCTTCCAATAGCTACTTCAATTAGCAGCCTGCAGCAGGGTGTAATTAAAACCTACTTCTTATTATTTTTCTTCACCATAATACTGGCAATTCTCATCCTTGTCGTCTAACTGCCCGTAAGGTCCCCCGACTTAGCCCCCGAGTTAACTCTAGAACTACAAAAAGCGTCAGTAATAAAACCCATCCCCCAAGCATTAAAACCCCTCCTCCTGAAGAATATATCAAAGCAACCCCACCAAAATCCCCCCGAAAGAGCATGAATTCACTAAATTCGTCAGCAGTTATCCATGAACCCTCATACCAGCCCTCGGAGAAAAAAACAGAGATAGACGCGACCAGAAACACATATACTGACATAAGAAGCAAAACGGGTCAACTTCCCCACCCCTCAGGATAAGGCTCCGAAGCCAGCGCTGCTGAGTACGCAAACACAACTAACATCCCACCTAAATAAATCAAAAACAAAATCAGAGATAAAAATGAACCCCCATGCCCTACTAAAATGCCACAGCCCATTCCTGCTACTGTAACAAGCCCCAAAGCAGCAAAGTAAGGTGACGGGTTCGAGGCCACGGCCGCTAGACCTAAAACCAAACCAACTAATAATAAATAAGTCATATAAACCATAATTCTTGCCAGGATTTTAACCAGGGCCTGCGACTTGAAAAACCACCGTTGTACTCAACTACAAGAACCTAATGGCCAATCTTCGAAAAACCCATCCCCTATTAAAAATCGCAAACGATGCCCTCGTTGATCTCCCAGCCCCATCGAACATTTCAGTTTGATGAAACTTCGGATCTCTTCTAGGACTTTGTTTGGCCGCCCAGATCGTTACGGGCCTTTTCCTTGCAATACATTATACATCAGACATTGCCACAGCATTTTCATCTGTCGCACATATTTGTCGTGATGTTAACTACGGCTGACTAATCCGAAACATGCATGCAAACGGTGCTTCCTTTTTCTTCATTTGCATCTACCTGCACATCGGACGGGGCTTGTATTATGGATCATACTTATATAAAGAGACATGAAATGTAGGTGTTGTCCTTCTCCTCCTTGTGATAATGACTGCTTTCGTAGGCTACGTCCTACCCTGAGGACAAATATCATTCTGAGGGGCTACCGTCATTACCAACCTTTTATCAGCCATCCCCTACATTGGAAACGCCCTAGTTCAATGGATCTGAGGCGGATTTTCAGTAGACAACGCCACCCTTACCCGGTTCTTTGCCTTCCATTTCCTCCTCCCCTTTGTAATTGCTGCTGCTACAGTTGTACATCTTATTTTCCTGCACGAGACAGGGTCGAATAACCCAACGGGTTTAAACTCAGACTCTGACAAAGTGTCTTTTCACCCCTACTTTTCTTATAAAGATCTTCTAGGCTTTGCTGCCCTGCTAGTAGCCCTTATCTCTTTAGCCCTCTTCTCCCCTAATCTACTTGGAGACCCTGACAACTTTACCCCTGCTAATCCTTTAGTAACTCCACCTCACATCAAACCTGAGTGATACTTCCTGTTCGCTTACGCCATTCTACGATCCATCCCAAACAAGCTTGGCGGGGTTCTAGCCCTATTAGCCTCTATTCTAGTTCTCTTTCTTGTCCCTATCCTGCACACATCAAAACAACGAAGCCTAACATTCCGACCCCTAACCCAATTCCTCTTCTGACTGCTAGTCGCCGATGTAATAATTTTAACCTGAATCGGAGGTATGCCTGTAGAACACCCTTACATTATCATTGGACAAGTCGCATCCTTCATTTATTTCTCCCTTTTTCTAGTCATGGCGCCTATGGCCGGCCTACTAGAAAACAAAGTCTTAAAATGACAATGCATTAGAAGCTCAGATGAAAGAGCACCGGTCTTGTAAGCCAGAGGTCGAAGGTTCAAGCCCTTCCTAATGCTCAGAGAGAAGGGATTCTAACCCCTGCCCCTGGCTCCCAAAGCCAGGATTCTTAGCTAAACTACTCGCTGATTTTCATACACCAGTTTTGCAATCCAGAGCGCATCACTTTTGCTACCAACGTTAAATTAACGTTGCACAAACGTTGCATCAGCACCCCATGGACACTAAATGACGCGAGGACGTTGAATAGACACCCCCTACCTCTAGCACCCTTTTAACGATTTCACTTTTTTTTTTTTTTTTGTTTAACGATTACGTTTTTTTTTGCGTTCCCGGACTCTGCCAGATTTCGACCGAAAACTGCCAGAATCCGCTCAAAATCCGCTCAAATACCAATATGTATTATCCCCATAAATGGTTTAAACCATTTTTGCCTAGTACACACTGACCATGCAAGTCAATTATATTTACCCCGCGCTCCAGGCCGCAGTACATACACCTACAGTTGGTGTATTTAGCACAAGTGTGCCTCAGCTAGTTTCAAGTCACCCACATCCTTCCTTTAATTGTTACTTAATGTAGTAAGAGCCCACCATCAGTTGATTCCTTAATGTCAACGGTTCTTGAAGGTGAGGGACAAAAATCGTGGGGGTTTCACCTCTTGAATTATTCCTGGCATTTGGCTCTACATCTCAAGGCCATACATTTCTCGTCTCTCACACTTTCACTGGCCCTGACATTGGTTAATGGTGGAGTACATACTCCTCGTTACCCCCCATGCCGGGCGTTCTTTCTAATGGACAACGGGTTTTCCTTTTTTTTTCCTTTTCACTTGGCATTTCACAGTGCATACAGACCTTGTTGACAAGGTTGAACATTTAGAAACCGGCCGCAAAGAATATTGGTGAATTATTTAAAGATATTAACAGATGAATTGCATAACTGATATCAAGAGCATAAATAACCAAATGAAACTAGGAACGTTTCTATAATATGACCCCCCGGCTTCCGCGCGTCAAACCCCCCTACCCCCCTAAACTAGTAAGAAGGCTATTATTCCTGCAAACCCCCCGGAAACAGGAAACCCCCTACTAGCATTTTAGCCCGCCCAAATTTGTGTGTATTTACATTATTTGTAATATTGCAAAA

>JWM 19

GCTAGTGTAGCTTAACTAAAGCATAACACTGAAGATGTTAAGACAAACCTTAGATTGGTTTCACGAGCACAAAAGTTTGGTCCTGACTTTACTATCAACTTTAGCTAAACTTACACATGCAAGTATCCGCAATCCCGTGAGAATGCCCTACAGTTTCCTTAAAGGAAACAAGGAGCTGGTATCAGGCTCAATTACTCCCGCCCATGACACCTTGCTTAGCCACACCCCCAAGGGAACTCAGCAGTGATAGACATTAAGCAATAAGTGAAAACTTGACTTAATTAAAGCTAAGAGAACCGGTTAAACTCGTGCCAGCCACCGCGGTTATACGAGCGGTTCGAGCTGATAGACTACGGCGTAAAGCGTGGTTAATAAGAATAAAACTAAAGTCGAATGTTTTCAAAGCTGTTATACGCACTCGAAAATTAGAAGACCAGAAACGAAAGTGACTTTAACCCTATGAACCCACGAAAACTATGAAACAAACTGGGATTAGATACCCCACTATGCATAGCTGTAAACTTTGATGAGCCATTACATTATCATCCGCCTGGGTACTACGAGCATCAGCTTAAAACCCAAAGGACTTGGCGGTGCTTTAGACCCACCTAGAGGAGCCTGTTCTAGAACCGATAACCCCCGTTAAACCTCACCCTCTCTTGTTTTTCCCGCCTATATACCGCCGTCGTCAGCTTACCCTGTGAAGGTCTAATAGTAAGCACAACCAGTTATACTCAAAACGTCAGGTCGAGGTGTAGCATATGAGAGGGGAAGAAATGGGCTACATTCCTTGTTTCAAGGAAAACGGATAACATAATGAAAGGTACGTTAGAAGGAGGATTTAGCAGTAAGCAGCAAATAGAGTGTTCTGCTGAAACTGGCCCTGAAGCGCGCACACACCGCCCGTCACTCTCCCCAACTCCGAGTTAAAAACCATATATAAACCTTTGAAGGAACAAAGGGGAGGCAAGTCGTAACATGGTAAGTGTACCGGAAGGTGCACTTGGATAAATCAGAGTATAGCTAAGAAAGAAAAGCATCTCCCTTACACCGAGAAGTCATCCGTGCAAATCGGATTACCCTGACTCTAACAAGCTAGCCCAAAACCTTAACTTAAAAATCAAATATTTCTAGTAATTAATAAACCAAACACATTAAATAAATCATTTTTCCCCCTGAGTATGGGAGACAGAAAAGGATAAAGGAGCTATAGACAAAGTACCGCAAGGGAAAGCTGAAAGAGAAATGAAACAAACCAGTAAAGAAGAACAAAGCAGAGATTAACCCTTGTACCTTTTGCATCATGAATTAGCCAGTTTAATCAAGCAAAGAGCACTGTAGTTTGAACCCCCGAAACTTAGTGAGCTACTTCAAGACAGCCTATGAAATAGGGCAAACCCGTCTCTGTGGCAAAAGAGTGGGAAGATCTTCAAGTAGAGGTGACAGACCTATCGAACTAAGTTATAGCTGGTTGCTCGTGAAATGAATAGAAGTTCAGCCTTTTGCTTTCTAAATTTCGATTTAGCACTACTTAGCCTAAATGACTAGAAAACAAAAGAGTTAGTCAAAGAGGGTACAGCCTGTTTGATAAAAGATACAACTTTACTAGGAGGATAAGAATCATAATTTTAAAGGTTTAATGCCCAGGTGGGCCTAAAAGCAGCCACCCTAATCAATAGCGTTAAAGCTTAAGCATAAAACACACCTACAATTCTGATAAATCAGTTTTAATCCCCTAAAGTTAACGAGCTATTTCATACCTTATGAAAGAAATTATGCTAGTATGAGTAATAAGAAGTTACGAACTTCTCCCTGCACACGTGTAAATCGGAACGGACAAACCACCGAATCTTAACGGCCCCAGTCAAAGAGGGGATGTCGGATAAAAAAAAGAACAAGAAACTCCCGACAAAACCACCGTTAACCCCACACCGGAGTGCTCCCTGGGAAAGACAAAAAGGGACAGAAGGAACTCGGCAAATATGCTCAAGCCTCGCCTGTTTACCAAAAACATCGCCTCTTGTAAAAGTTAAATAAGAGGTACCGCCTGCCCTGTGACTAGTAGTTTAACGGCCGCGGTATTTTGACCGTGCAAAGGTAGCGCAATCACTTGCCTTTTAAATGAAGGCCTGTATGAATGGCACGACGAGGGCTTAACTGTCTCCTCTCCCTAGTCAATGAAATTGATCTCCCCGTGCAGAAGCGGGGATAATAACATAAGACGAGAAGACCCTGTGGAGCTTTAGACTATGAGCAGACCATGTCAAGAATAACAAACAAGTAAATTAAACAGATTGGTCCCTGCTTCTCTGTCTTTGGTTGGGGCGACCGCGGGATAATAAAAAGCTCCCACGAGGATTGAGAACCCTTATCTTATAACCAAGAGCTTCTCCTCTAAGTAACAGAACATCTGACCTTAATGATCCGGCCTGGCCGATCAACGGACCGAGTTACCCCAGGGATAACAGCGCAATCCTCTTTTAGAGTCCATATCGACAAGAGGGTTTACGACCTCGATGTTGGATCAGGACATCCTAATGGTGCAGCCGCTATTAAGGGTTTGTTTGTTCAACAATTAAAGTCCTACGTGATCTGAGTTCAGACCGGAGTAATCCAGGTCAGTTTCTATCTATGACGTACTCTCTTCTAGTACGAAAGGACCGAAGAAAGAAGGCCTATGAAAAGTTATGCCTTAGTCTCACCTTATGAAGAAAACTAAATAAGACAAGAGGTTACACCCCTTAGTCATAGAAAATGACATGTTAAGGTGGCAGAGCCCGGATATTGCAAAAGACCTAAGCCCTTTCCACAGAGGTTCAATTCCTCTCCTTAACTATGTTCTCAACAATATTAAGCTTCATTATTAATCCCCTAATTGTTATGGTTTTTGTTTTGCTGGCAGTAGCCCTCTTAACCTTGGTAGAGCGTAAAGTGCTAAGCTACATGCAACTTCGTAAAGGCCCAAATGTTGTTGGCCCTTACGGCCTTTTGCAACCCTTCGCTGATGGCTTGAAACTTTTCATGAAAGAGCCCGTCCGACCCTCCACCTCCTCGCCCGCCTTATTCTTAATTACCCCTATTATAGCCCTTACCTTAGCCCTAACCCTCTGAGCCCCCCTTCCTATGCCTTTTCCCATCACCGACCTAAACTTAGGCATTTTATTTATTTTAGCACTATCGAGCCTGGCAGTATATTCTATTCTTGGCTCCGGATGGGCCTCCAATTCTAAATATGCATTGATTGGTGCTCTTCGAGCAGTCGCCCAAACCATCTCTTATGAAGTGAGCTTGGGCCTTATTCTTCTTAACACAATTGTCTTTACAGGGGGTTTTACTCTTCAAACCTTCAGCACCGCACAAGAAGCCACCTGATTACTTCTACCAGCATGACCACTAGCAGCCATGTGATATATCTCCACACTCGCGGAAACTAACCGGGCCCCTTTCGACTTAACTGAAGGAGAGTCCGAACTAGTGTCTGGCTTCAACGTAGAGTATGCCGGCGGACCTTTTGCCCTTTTTTTTCTGGCAGAATACGGTAACATTTTACTTATAAATACCCTCTCAGCAGTACTATTTCTAGGCTCTTCAACCTACCACAGCTTTCCAGAACTAACCGCGACCTTATTAATGCTTAAAGCCACCCTCCTTTCAGTCGTATTTTTATGAGTGCGAGCATCTTACCCTCGGTTTCGATACGATCAACTAATGCATTTAATTTGAAAAAACTTTTTACCTCTGACCCTAGCACTAGTTATTTGACACCTTTCTCTTCCGATCACGTTGAGCGGCCTTCCCCCTCAACTTTAACTCAGGAAATGTGCCTGAAAAAGGATCACTTTGATAGGGTGAATAATGAGGGTTAAAGCCCCTCCATCTCCTTAGAAAGAAGGGGTTTGAACCCTACCTGAAGAGATCAAAACTCTTAGTGCTTCCACTACACCACTTCCTAGTAAAGTCAGCTAATAAAAGCTTTTGGGCCCATACCCCAAATATGTTGGTTAAAATCCTTCCTTTGCTAATGAATCCTTACGTCCTTTCAATTCTACTTATAGGATTAGGCCTCGGCACTACAGTCACATTCGCTAGCTCACACTGACTATTAGCATGAATAGGCCTTGAAATAAATACCCTCGCCATTTTGCCGTTAATAGCACAACATCACCACCCCCGAGCCGTTGAAGCCACCACCAAGTATTTTTTAATTCAATCGGCAGCCGCAGCAACCATCTTATTTGCCAGCTCAACAAACGCCTGACTTTCGGGCCAGTGGGACATCATAAGTATTAATCACCCTCTTCCAACCGTCATAATTACAGTCGCTCTGTCCTTAAAACTAGGCTTGGCCCCTCTTCACGCGTGACTTCCCGAAGTAATTCAAGGCCTGGACTTAACTACGGGCTTAATCCTCTCCACATGACAAAAACTCGCACCCTTTGCCCTCCTCGTTCAAATCTTCCCCGACACCCCCCTTCTCATCACTTCTCTAGGACTTCTTTCAATATTAGTTGGGGGATGAGGGGGTTTAAACCACACACAGCTCCGCAAAGTGCTCGCATATTCTTCGATCGCCCACTTAGGCTGAATAATAGTAATTATGCAATTCTCCACCCCCCTTACAATTCTTGCTTTATCAACATACATTGTCATAACATCATCTACTTTTCTAATCTTTAAACTCCTTAAATCCACAGATATGAACAGCCTGGCAACATCTTGAGCTAAAACCCCCTCCATTACAGCCCTAGCACCTTTAGTGCTATTATCCTTAGGCGGACTCCCTCCCCTCTCGGGCTTTATGCCAAAATGACTAATTATTCAGGAGTTAACTAAGCAAGACCTAGCCCTAGTTGCGACCTTAGCCGCCCTCTCTGCGCTACTCAGCCTTTTCTTTTACCTACGCATTTGTTACTCCCTCACATTTACCTCCTCTCCTAATAATCTCATGGGAACACCCCCCTGACGACTAGTAACAAAGCAAGTATCACTTCCCCTGGCTATGACAACCGCCCTCTCTATTCTCCTACTCCCGGTTACCCCTGCAATCTTATCAGTAGTTCTCCCCTTGTAAAGAGGCTTAGGATAGTATTAAGACCAAGGGCCTTCAAAGCCCTAAGCGGGAGTGAAAGCCCCCCAGCCTCTGTAAGACCTACGGGACACTAACCCACATCTTCTGTATGCAAAACAGACACTTTAATTAAGCTAAAGCCTTCCTAGGTGGGTAGGCCTCGATCCTACAATCTCTTAGTTAACAGCTAAGCGCCTAAACCAGCGGGCATCCATCTACCTTTCCCCCGCCTTGCCGAAAAAAAAAGGCGGGGGAAAGCCCCGGCAGGGTATTAGCCTGCTACTTAAGATTTGCAATCTAATGTGTTAACACCTCGGAGCTGGTAAGAAGAGGACTTTAACCTCTGTCTATGGGGCTACAATCCACCGCTAAACGCTCAGCCACCTTACCTGTGGCAATCACACGTTGATTTTTCTCAACTAATCACAAAGACATCGGCACCCTATATCTAATCTTTGGTGCCTGGGCGGGAATAGTAGGGACGGCCTTAAGTCTACTCATTCGGGCAGAATTAAGTCAACCAGGCTCCCTATTAGGGGACGACCAGATCTATAACGTAATTGTAACTGCACATGCTTTCGTAATAATTTTCTTTATAGTAATGCCAATTATAATTGGAGGGTTCGGCAACTGATTAATTCCTTTAATGATCGGAGCTCCCGACATGGCCTTCCCCCGGATAAATAATATAAGCTTTTGACTCCTGCCCCCTTCTTTCCTTCTATTATTGGCCTCATCTGGTGTAGAAGCTGGTGCCGGGACAGGATGAACCGTATATCCCCCCTTGTCCGGTAATTTGGCACACGCAGGGGCCTCCGTAGATTTAACCATTTTCTCTCTTCACCTGGCCGGAATCTCTTCTATTCTAGGGGCCATTAATTTCATTACAACTATTATTAATATAAAACCTCCAGCCATTTCCCAATATCAAACCCCTTTATTTGTCTGAGCTGTTCTAATTACCGCAGTATTACTCCTACTCTCTCTTCCTGTTCTAGCTGCAGGTATCACTATGCTTCTCACAGATCGAAACCTAAATACAACATTTTTCGACCCCGCAGGAGGGGGGGACCCCATTCTTTATCAACATTTATTCTGATTCTTTGGGCATCCTGAAGTCTACATTCTGATTTTGCCCGGCTTCGGAATGATTTCTCACATTGTAGCATATTACTCAGGCAAAAAAGAGCCGTTTGGCTACATGGGAATAGTATGAGCTATAATAGCAATTGGCTTACTGGGGTTTATCGTATGAGCCCATCATATGTTCACTGTAGGGATGGACGTGGACACTCGAGCTTATTTTACATCCGCCACTATAATTATCGCAATTCCTACAGGAGTCAAAGTGTTTAGTTGACTAGCTACCTTGCATGGGGGCTCAATCAAATGAGAGACCCCTCTGTTATGAGCTCTAGGCTTTATTTTCTTATTTACTGTCGGAGGTTTAACAGGAATTGTTTTAGCCAACTCATCTCTGGACATTATACTTCATGACACATACTATGTTGTAGCCCACTTCCACTATGTCCTCTCTATAGGAGCAGTCTTTGCCATCATGGGGGCATTCGTTCACTGATTCCCCCTATTCTCAGGCTACACCCTTCACAATACGTGAACAAAAATCCACTTCGGAGTTATGTTTGTAGGTGTAAACCTCACCTTTTTCCCTCAGCACTTCTTAGGATTGGCGGGAATACCTCGACGATATTCAGATTACCCTGACGCATACACACTGTGAAATACTATCTCATCCCTGGGGTCACTAATCTCCCTTATTGCTGTAATTATATTCCTATTTATTATCTGGGAAGCATTCGCGGCAAAACGTGAAGTCTTATCAGTTGAACTAACAGCCACAAACGTAGAATGACTGCACGGGTGTCCTCCCCCTTACCATACATTTGAAGAACCTGCATTCGTTCAAATTCAGCAATCCAAATTTTAATCGAGAAAGGAAGGAGTCGAACCCCCATAAACTGGTTTCAAGCCAGCCACATAACCGCTCTGTCACTTTCTTCTCTAAGTTAATAAGATTCTAGTTAAAAGAATAACGCTGCCTTGTCAAGGCAAAATTGTGGGTTAAAGCCCCACGTATCTTGCTTATGGCACATCCATCTCAACTAGGATTCCAAGATGCAGCTTCACCCGTTATAGAAGAACTTCTCCATTTTCATGACCATGCATTAATAATTGTTTTCTTAATCAGCACCCTTGTTCTTTACATTATTGTGGCTATGGTAACCACCAAGCTAACAAATAAGTTCATTCTGGACTCCCAAGAAATTGAAATCATCTGAACCTTACTACCAGCAATTATCCTAATTCTGATCGCCCTACCCTCCCTTCGCATTCTCTACCTCATGGATGAAATCAATGACCCCCACCTCACAATTAAAGCCATGGGACATCAATGATACTGAAGCTACGAATATACGGATTATGAAGACCTGGGGTTCGACTCATATATGGTCCCTACACAAGATCTCGCCCCTGGTCAATTTCGACTACTTGAGACAGACCATCGCATGGTTATTCCTGTTGAGTCCCCCATCCGAGTTCTTGTCTCCGCCGAGGATGTCTTACATTCATGAGCCGTCCCGAGCCTCGGAGTAAAAATGGACGCCGTCCCCGGCCGCCTAAATCAAACAGCCTTCATTACTTCCCGACCAGGTGTGTTTTATGGACAATGCTCAGAGATTTGCGGAGCTAATCATAGCTTTATACCCATTGTAGTGGAAGCTGTTCCTCTAGAACACTTCGAGAACTGGTCTTACCTAATACTTCAAGATGCCTCACCAGGAAGCTAAAAGGGAATAGCATTAGCCTTTTAAGCTAAAAATTGGTGACTCCCGCCCACCCCTGGTGACATGCCTCAGTTGAACCCCGCACCCTGATTTGCTATTATAGTATTCTCGTGGCTAGTTTTCCTAGCCGTTATTCCACCTAAAGTTCTAGCTCACCATTTTCCCAATGACCCCGCCCCACAGAGCGTAAAAAAATCAAAAACAGAGACTTGACCCTGACCATGACTTTAAGCCTCTTTGATCAATTTATGAGCCCTACACTTCTAGGGGTGCCTCTTATCGGACTCGCCCTAACATTGCCATGAGTCCTTTACTTCCGACCCGGTGCCCGATGACTTAATAACCGCTTGATTACCCTTCAATCTATATTCATAAACTGGTTTGTAAAACAAATCTTTCAACCAATAAGCTTAGGCGGACACAAATGGGCCGCTCTCCTCATATCTTTAATACTATTTTTAATTACCTTAAATATGCTGGGCCTGCTGCCTTACACATTTACTCCAACAACGCAGCTGTCACTTAATATAGCCTTTGCAGTTCCACTTTGACTAGCAACTGTCATTATTGGAATACGAAACCAGCCAACACATGCCCTTGGTCACCTTCTCCCTGAAGGAACTCCTACCGCCCTAATCCCGGTTTTAATCGTGATTGAAACAATTAGCCTTTTTATTCGACCCTTGGCCCTCGGTGTTCGACTTACCGCAAACTTGACAGCCGGACACCTTCTAATTCAACTAATTGCAACTGCGGCTTTTGTTCTTTTCCCTATAATACCAACAGTAGCTGCTCTTACCTCTGTCTTACTATTCTTGCTAACCCTGCTAGAAGTCGCCGTGGCCATAATCCAAGCCTATGTATTTGTACTTCTTTTAAGCCTTTATCTACAAGAAAACGTCTAATGGCCCATCAAGCACATGCATATCATATAGTTGACCCAAGCCCTTGACCCCTCACAGGCGCAGTAGCCGCCCTTCTACTTACGTCTGGAACAGCAATCTGAATACACTTTAACTCCACAGTTCTCATGTCCCTTGGACTTGTCCTGCTACTACTAACCATATATCAATGATGGCGAGACATTATCCGAGAGGGTACCTTTCAAGGTCATCATACACCCCCTGTTCAAAAGGGCCTTCGGTACGGGATAATTCTATTTATTACCTCAGAGGTCTTCTTTTTCCTAGGTTTCTTCTGAGCATTTTATCACTCAAGCCTAGCCCCAACCCCCGAACTTGGTGGGTGTTGACCACCTATGGGTATTACAACACTGGACCCCTTTGAAGTCCCCCTTCTCAATACTGCTGTCCTTCTCGCCTCCGGTGTCACGGTCACTTGAGCTCACCATAGTATTATGGAGGGGCAGCGAAAACAAGCAATTCAATCCTTAACACTCACAATTCTACTGGGGTTCTACTTTACATTCCTTCAAGCAATAGAGTACTACGAGGCACCCTTCACCATTGCAGATGGCGTCTATGGCTCTACATTTTTTGTGGCAACGGGGTTTCATGGCCTCCATGTAATTATTGGGTCAACATTTCTGGCAGTCTGCCTCTTACGACAAGTCCAATTCCACTTTACATCAGAACATCACTTCGGATTTGAAGCTGCAGCATGATACTGACACTTTGTAGACGTAGTCTGACTATTCTTATATATCTCTATCTACTGATGAGGCTCATATCTTTCTAGTATTAAAAAGTACAAGTGACTTCCAATCACTCAGTCTTGGTTAGACTCCAAGGAAAGATAATGAACTTAGTACTAGTCATTATTTGCATCTCATTAGCCCTCGCCGCACTGCTCGCAACTGTTTCATTTTTTCTCCCACAAATAACCCCTGATTATGAGAAACTCTCACCATATGAGTGCGGCTTTGACCCAGTGGGATCCGCCCGTTTGCCATTCTCCATTCGCTTTTTTCTAGTCGCAATCCTATTTCTCCTCTTCGACTTAGAAATTGCCTTACTTCTCCCCCTTCCCTGAGGAGACCAACTCCCCTCCCCTCTGACAACTTTCTTCTGAGCTTCTGCTATCCTTATACTACTAACTCTAGGGTTAATCTATGAATGACTTCAAGGGGGCCTAGAGTGGGCAGAATAGGTACTTAGTTTAATAAAAACATTTGATTTCGGCTCAAAAACTTATGGTTTAAGTCCATATTTACCTGATGACCTTAACTCACTATGCATTCTCGTCAGCCTACTTTGTCAGCTTCATGGGTCTAATTTTTTACCGAAAGCATCTTCTCTCCGCCTTACTTTGCTTAGAAGCCATAATACTTATTCTTTTTATTTCACTATGCCTGTGAGGCCTAGTCTTAGCCTCAAGTGCATTTTCGGCAGGCCCAATGATCTTACTTGCTTTCTCAGCATGTGAAGCAAGTGCAGGCCTAGCACTGCTTGTAGCAATAGCTCGAACCCACGGGACTGACCGTTTAAAAAACCTTAGCCTACTCCAATGTTAATAATTCTTATTCCTACTGTTATGCTTCTACCCACAATCTGACTAAGCCCCGCTAAATACCTGTGGTCCTCAGCACTTGGCCATAGCATAATAATTGCTCTTATAAGCCTCTCCTGACTTAGCCTCCCGGGGGAGGTTGGCTGATCTTCCCTTAACACTTTTATAGCAACAGACCCTCTCTCTACCCCCCTTCTCGTACTTACTTGCTGACTTCTACCCTTAATAATTCTTGCAAGCCAAAACCATATAGCCCAAGAACCTACCAATCGCCAGCGAACCTATATCTCTCTCCTTACTTCCCTTCAAATCTTCTTAATCTTAGCATTTGGAGCAACCGAGATAATTATGTTCTATATTATATTTGAAGCGACCTTAATTCCCACACTCGTAATTATCACACGATGAGGGAACCAAACAGAGCGATTAAACGCAGGTATTTACTTTTTATTTTATACCTTAGCCGGCTCTTTACCACTACTAGTGGCCCTCCTTCTACTTCAGACCTCGACAGGAACTCTTTCTTTTCTAACCACTCAATTTTTTCCCCCCTTACAACTGCATACAGAAGCAAGTAAGTTCTGGTGGGCGGGCTGTTTACTAGCATTCTTAGTAAAAATGCCGCTATATGGGGCACACCTTTGACTTCCAAAAGCTCACGTCGAAGCCCCCATCGCCGGGTCAATAGTCCTTGCAGCCGTTCTTTTAAAACTAGGGGGTTACGGTATGATACGAGTCATTATTATCTTAGAACCCCTAACGAAACAACTCAGCTACCCCTTTATTGTTCTTGCCCTGTGGGGCGTTGTAATAACTGGCTCAATCTGCCTCCGACAAACAGACCTTAAATCACTAATCGCTTACTCCTCAGTAAGCCACATGGGCCTTGTCGCAGCAGGCATCCTGATCCAAACTCCTTGGGGGTTTACAGGAGCATTAATCCTTATAATTGCCCATGGCTTAACCTCCTCCGCCCTATTCTGTTTAGCCAACACTAACTATGAGCGAACACATAGCCGAACCATACTTTTAGCCCGGGGTTTACAAATGGTCCTTCCTCTTTTAGCAACTTGGTGGTTTCTACTTACCCTCGCCAACCTAGCACTCCCTCCGCTACCCAACCTCATAGGGGAGCTTATGATTATCTCATCCTTGTATAACTGGTCAAACTGGTCTCTAATCCTGACCGGAGCGGGAGTACTAATTACCGCTAGCTACTCTCTTCATATATTCCTAACCACTCAACGCGGCCCTATTACTAACCCCGTCTTGGCAATTGAACCAACCCACACACGAGAACATCTCCTCATAATTCTTCACCTTCTTCCTCTCCTCCTTCTAATTTTAAAACCCTGCTTGATCTGGGGCTGAACAGTTTGTAGGCGTAGTTTAAATAAAGCGCTAGATTGTGATTCTAGAAATAAGAGTTAAACCCTCTTCACCCACCGAGAGGGGTCGCCGTGACAGCAAGAACTGCTAATTCTAGCCCCTTTGGTTAAAATCCGAAGCCCACTCGAACAGGCTTCTAAAGGATAACAGCTCATCCGTTGGTCTTAGGAACCAAAAACTCTTGGTGCAACTCCAAGTAGCAGCTATGCACTTTACAACAATGATTCTCTCCTCAAGCCTAATAACAATTTTCCTTCTTCTCATCCTTCCAGTCCTAGGAACACTAAACCCTAACCCCACGGGGGGCCTGTGAGCCACAAAAAACGTTAAAACGGCAGTTAAGATAGCCTTTTTTGTAAGTCTTTTGCCTCTTTTTATCTTTCTTAATGAAGGGGTAGAGACTGTTATAACAAACTGAAAATGAATAAATACTCTAATGTTTGAAATTAATATCAGCTTTAAATTTGACCTCTACTCCGTAGTATTTACCCCTGTGGCCCTCTACGTAACATGATCAATTTTAGAGTTCGCATCTTGGTATATACACAGTGATCCCAACATAAACCGATTCTTTAAATATCTTCTAATCTTTCTTATTGCTATGGTCGTTCTGGTCACAGCCAACAACATGTTCCAACTATTTATTGGCTGAGAAGGTGTTGGAATTATATCTTTCTTACTTATTGGCTGGTGATTCGGGCGGGCTGACGCCAACACTGCGGCCCTCCAGGCCGTAGTTTATAACCGAGTTGGTGATATCGGCCTTATTCTAGCAATAGCATGAATAGTAGCAAACCTAAACTCATGAGAAATACAACAGCTCTTTTCTATGTCTAAAGGCCATGATATAACCCTTCCTTTATTAGGCCTAGTACTGGCCGCTACCGGAAAGTCCGCCCAATTTGGACTTCACCCCTGGCTCCCCTCAGCCATAGAGGGTCCAACACCGGTCTCTGCCCTCCTGCACTCTAGCACCATGGTGGTTGCTGGTATTTTTCTTCTTATTCGCCTCAGCCCCTTAATGCAAGAAAGCCCATTAATTCTCTCAACATGCCTTTGCCTGGGGGCCCTAACTACCGTCTTTACTGCAACATGTGCCCTTACCCAAAATGACATTAAAAAAATCGTTGCATTTTCTACATCAAGTCAATTAGGACTAATAATAGTTACCATCGGACTAGGCCAGCCCCAGCTCGCCTTTCTTCATATCTGCACCCACGCCTTCTTTAAAGCAATACTTTTCTTATGTTCCGGCTCCATCATTCATAGCCTTAACGATGAGCAAGATATCCGAAAAATAGGCGGACTTCACAAGGTGCTTCCACTGACCTCTTCTTGTCTAACCATTGGCAGCCTAGCTCTAACAGGAGTCCCCTTTTTAGCAGGCTTCTTTTCCAAAGACGCCATCATTGAAGCTATAAATACATCCTACCTTAACGCCTGAGCCCTAATTTTAACGCTTCTAGCTACATCATTCACCGCAGTTTACAGTCTCCGAGTCGTATTCTTTGCCTCTATGGGCCACCCGCGTTTTAATCCAGTCTCCCCAATTAATGAAAATAACCCTACAGTGATAAACCCCATTAAACGACTCGCTTGGGGAAGCATTTTGGCAGGGTTGCTAATTACGACCAATATTGTTCCACTTAAAACCCCCGTTTTAACCATGCCTTTCACCTTAAAAATGGCCGCACTGACTGTAACAATTATAGGACTACTCACAGCCTTAGAACTAGCGTCTCTCACGTCCCAACAATTTAAAATCAAACCCTTATCTTCTACTCACCACTTCTCAAATATATTAGGATTTTTCCCCAGTGTAGTCCATCGACTAGTCCCAAAAACTGGCCTGATTCTAGGACAACTAGTTGCCAATCAGACAGTTGACCAAACCTGACTAGAGAAAACAGGACCAAAAATAGTAACCTCCGTTAACCTTCCAATAGCTACTTCAATTAGCAGCCTACAGCAGGGTGTAATTAAGACCTACTTCTTATTATTTTTCTTCACCATAATACTGGCAATTCTCATCTTTGTCGTCTAACTGCCCGTAAGGTCCCCCGACTTAGCCCCCGAGTTAACTCTAGAACTACAAAAAGCGTCAGTAATAAAACTCATCCCCCAAGCATTAAAACTCCTCCTCCTGAAGAATATATCAGAGCAACCCCACCGAAATCCCCCCGAAAGAGCATGAATTCACTAAACTCGTCAGCAGTTACCCATGACCCCTCATACCAGCCCTCAGAGAAAAAAACAGAGATAGACGCGACCAGGAACACATATACTGACATAAGAAGCAAAACGGGTCAACTTCCCCACCCCTCAGGATAAGGCTCCGAAGCCAGCGCTGCTGAGTACGCAAACACAACTAACATCCCACCTAAATAGATCAAAAACAAAATCAGAGATAAAAATGAACCCCCATGCCCTACTAAAATGCCACAGCCCATTCCTGCTACTGTAACAAGACCTAAAGCAGCAAAGTAGGGTGACGGGTTCGAGGCCACGGCCGCTAGACCTAAGACCAAACCAACTAATAATAAATAAGTCATATAAACCATAATTCTTGCCAGGATTTTAACCAGGGCCTGCGACTTGAAAAACCACCGTTGTACTCAACTACAAGAACCTAATGGCCAATCTTCGAAAAACCCATCCCCTATTAAAAATCGCAAACGATGCCCTCGTTGATCTCCCAGCCCCGTCGAACATTTCAGTTTGATGAAACTTCGGGTCTCTTCTAGGACTTTGTTTAGCCGCCCAAATCGTTACGGGCCTTTTCCTTGCAATACATTATACATCAGACATTGCCACAGCATTTTCATCTGTAGCACATATTTGTCGTGATGTCAACTACGGCTGACTAATCCGGAACATGCATGCAAACGGTGCTTCCTTTTTCTTCATTTGCATCTACCTGCACATCGGACGGGGCTTGTATTATGGATCATACTTATATAAAGAGACATGAAATGTAGGTGTTGTCCTTCTCCTCCTAGTGATAATGACTGCTTTCGTAGGCTACGTTCTACCCTGAGGACAAATGTCATTCTGAGGGGCCACCGTCATTACCAACCTTTTATCAGCCATTCCCTACATTGGAAACGCCCTAGTTCAATGGATCTGAGGCGGATTTTCAGTAGACAACGCCACCCTTACCCGGTTTTTTGCCTTCCATTTCCTCCTCCCCTTTGTAATTGCTGCTGCTACAGTTGTACATCTTATTTTCCTGCACGAGACAGGGTCGAATAACCCAACGGGTTTAAACTCAGACTCTGACAAAGTGTCTTTTCACCCCTACTTTTCTTATAAAGATCTTCTAGGCTTTGCTGCCCTGCTAGTAGCCCTTATCTCTTTAGCCCTCTTCTCCCCTAATCTACTTGGAGACCCTGATAACTTTACCCCTGCTAATCCTTTAGTAACTCCACCTCACATCAAACCTGAGTGATACTTCCTGTTCGCTTACGCCATTCTACGATCCATCCCAAACAAGCTTGGTGGGGTTCTAGCCCTATTAGCCTCTATTCTAGTTCTCTTTCTTGTCCCTATCCTGCACACATCAAAACAACGAAGCCTAACATTCCGACCCCTAACCCAATTCCTCTTCTGATTGCTAGTCGCCGATGTAATAATTTTAACCTGAATCGGAGGTATGCCTGTAGAACACCCTTACATTATCATTGGGCAAGTCGCATCCTTCATTTATTTTTCCCTTTTTCTAGTTATGGCGCCTATGGCCGGCCTACTAGAAAACAAAGTCTTAAAATGACAATGCATTAGAAGCTCAGATGAGAGAGCACCGGTCTTGTAAGCCAGAGGTCGAAGGTTCAAGTCCTTCCTAGTGCTCAGAGAGAAGGGATTCTAACCCCTGCCCCTGGCTCCCAAAGCCAGGATTCTTAGCTAAACTACTCGCTGATTTTCATATACCAGTTTTGCAATCCAGAGCGCATCACTTTTGCCACCAGCGTTAAATTAACGTTGCACAAACGTTGCATCAGCACCCCATGGACACTAAATGACGCGAGGGCGTTAAATAGACACCCTCTACCTCTAGCACCCTTTTAACGATTTCACTTTTTTTTTTTTTTTTGTTTAACGATTACGTTTTTTTTTGCGTTCCCGGACTCTGCCAGATTTCGACCGAAGACTGCCAGAATCCGCTCAAAATCCGCTCAAATACCAATATGTATTATCCCCATAAGTGGTTTAAACCATTTTTGCCTAGTACACACTGACCATGCAAGTCAATTATATTTACCCCGCGCTCCAGGCCGCAGTACATACACCTACAGTTGGTGTATTTAGCACAAGTGTGCCTCAGCTAGTTTCAAGTCACCCACATCCTTCCTTTAATTGTTACTTAATGTAGTAAGAGCCCACCATCAGTTGATTCCTTAATGTCAACGGTTCTTGAAGGTGAGGGACAAAAATCGTGGGGGTTTCACCTCTTGAATTATTCCTGGCATTTGGCTCTACATCTCAAGGCCATACATTTCTCGTCTCTCACACTTTCACTGGCCCTGACATTGGTTAATGGTGGAGTACATACTCCTCGTTACCCCCCATGCCGGGCGTTCTTTCTAATGGACAACGGGTTTTCCTTTTTTTTTCCTTTTCACTTGGCATTTCACAGTGCATACAAACCTTGTTGACAAGGTTGAACATTTAGAAATCGGCCGCAAAGAATATTGGTGAATTATTTAAAGATATTAACAGATGAATTGCATAACTGATATCAAGAGCATAAATAACCAAATGAAACTAGGAACGTTTCTATAATATGACCCCCCGGCTTCCGCGCGTCAAACCCCCCTACCCCCCTAAACTAGTAAGAAGGCTATTATTCCTGCAAACCCCCCGGAAACAGGAAACCCCCTACTAGCATTTTAGCCCGCCCAAATTTGTGTGTATTTACATTATTTGTAATATTGCAAAA

>JWM 1

GCTAGTGTAGCTTAACTAAAGCATAACACTGAAGATGTTAAGACAAACCTTAGATTGGTTTCACGAGCACAAAAGTTTGGTCCTGACTTTACTATCAACTTTAGCTAAACTTACACATGCAAGTATCCGCAATCCCGTGAGAATGCCCTACAGTTTCCTTAAAGGAAACAAGGAGCTGGTATCAGGCTCAATTACTCCCGCCCATGACACCTTGCTTAGCCACACCCCCAAGGGAACTCAGCAGTGATAGACATTAAGCAATAAGTGAAAACTTGACTTAATTAAAGCTAAGAGAACCGGTTAAACTCGTGCCAGCCACCGCGGTTATACGAGCGGTTCGAGCTGATAGACTACGGCGTAAAGCGTGGTTAATAAGAATAAAACTAAAGTCGAATGTTTTCAAAGCTGTTATACGCACTCGAAAATTAGAAGACCAGAAACGAAAGTGACTTTAACCCTATGAACCCACGAAAACTATGAAACAAACTGGGATTAGATACCCCACTATGCATAGCTGTAAACTTTGATGAGCCATTACATTATCATCCGCCTGGGTACTACGAGCATCAGCTTAAAACCCAAAGGACTTGGCGGTGCTTTAGACCCACCTAGAGGAGCCTGTTCTAGAACCGATAACCCCCGTTAAACCTCACCCTCTCTTGTTTTTCCCGCCTATATACCGCCGTCGTCAGCTTACCCTGTGAAGGTCTAATAGTAAGCACAACCAGTTATACTCAAAACGTCAGGTCGAGGTGTAGCATATGAGAGGGGAAGAAATGGGCTACATTCCTTGTTTCAAGGAAAACGGATAACATAATGAAAGGTACGTTAGAAGGAGGATTTAGCAGTAAGCAGCAAATAGAGTGTTCTGCTGAAACTGGCCCTGAAGCGCGCACACACCGCCCGTCACTCTCCCCAACTCCGAGTTAAAAACCATATATAAACCTTTGAAGGAACAAAGGGGAGGCAAGTCGTAACATGGTAAGTGTACCGGAAGGTGCACTTGGATAAATCAGAGTATAGCTAAGAAAGAAAAGCATCTCCCTTACACCGAGAAGTCATCCGTGCAAATCGGATTACCCTGACTCTAACAAGCTAGCCCAAAACCTTAACTTAAAAATCAAATATTTCTAGTAATTAATAAACCAAACACATTAAATAAATCATTTTTCCCCCTGAGTATGGGAGACAGAAAAGGATAAAGGAGCTATAGACAAAGTACCGCAAGGGAAAGCTGAAAGAGAAATGAAATAAACCAGTAAAGAAGAACAAAGCAGAGATTAACCCTTGTACCTTTTGCATCATGAATTAGCCAGTTTAATCAAGCAAAGAGCACTGTAGTTTGAACCCCCGAAACTTAGTGAGCTACTTCAAGACAGCCTATGAAATAGGGCAAACCCGTCTCTGTGGCAAAAGAGTGGGAAGATCTTCAAGTAGAGGTGACAGACCTATCGAACTAAGTTATAGCTGGTTGCTCGTGAAATGAATAGAAGTTCAGCCTTTTGCTTTCTAAATTTCGATTTAGCACTACTTAGCCTAAATGACTAGAAAACAAAAGAGTTAGTCAAAGAGGGTACAGCCTGTTTGATAAAAGATACAACTTTACTAGGAGGATAAGAATCATAATTTTAAAGGTTTAATGCCCAGGTGGGCCTAAAAGCAGCCACCCTAATCAATAGCGTTAAAGCTTAAGCATAAAACACACCTACAATTCTGATAAATCAGTTTTAATCCCCTAAAGTTAACGAGCTATTTCATACCTTATGAAAGAAATTATGCTAGTATGAGTAATAAGAAGTTACGAACTTCTCCCTGCACACGTGTAAATCGGAACGGACAAACCACCGAATCTTAACGGCCCCAGTCAAAGAGGGGATGTCGGATAAAAAAAAGAACAAGAAACTCCCGACAAAACCACCGTTAACCCCACACCGGAGTGCTCCCTGGGAAAGACAAAAAGGGACAGAAGGAACTCGGCAAATATGCTCAAGCCTCGCCTGTTTACCAAAAACATCGCCTCTTGTAAAAGTTAAATAAGAGGTACCGCCTGCCCTGTGACTAGTAGTTTAACGGCCGCGGTATTTTGACCGTGCAAAGGTAGCGCAATCACTTGCCTTTTAAATGAAGGCCTGTATGAATGGCACGACGAGGGCTTAACTGTCTCCTCTCCCTAGTCAATGAAATTGATCTCCCCGTGCAGAAGCGGGGATAATAACATAAGACGAGAAGACCCTGTGGAGCTTTAGACTATGAGCAGACCATGTCAAGAATAACAAACAAGTAAATTAAACAGATTGGTCCCTGCTTCTCTGTCTTTGGTTGGGGCGACCGCGGGATAATAAAAAGCTCCCACGAGGATTGAGAACCCTTATCTTATAACCAAGAGCTTCTCCTCTAAGTAACAGAACATCTGACCTTAATGATCCGGCCTGGCCGATCAACGGACCGAGTTACCCCAGGGATAACAGCGCAATCCTCTTTTAGAGTCCATATCGACAAGAGGGTTTACGACCTCGATGTTGGATCAGGACATCCTAATGGTGCAGCCGCTATTAAGGGTTTGTTTGTTCAACAATTAAAGTCCTACGTGATCTGAGTTCAGACCGGAGTAATCCAGGTCAGTTTCTATCTATGACGTACTCTCTTCTAGTACGAAAGGACCGAAGAAAGAAGGCCTATGAAAAGTTATGCCTTAATCTCACCTTATGAAGAAAACTAAATAAGACAAGAGGTTACACCCCTTAGTCATAGAAAATGACATGTTAAGGTGGCAGAGCCCGGATATTGCAAAAGACCTAAGCCCTTTCCACAGAGGTTCAATTCCTCTCCTTAACTATGTTCTCAACAATATTAAGCTTCATTATTAATCCCCTAATTGTTATGGTTTTTGTTTTGCTGGCAGTAGCCCTCTTAACCTTGGTAGAGCGTAAAGTGCTAAGCTACATGCAACTTCGTAAAGGCCCAAATGTTGTTGGCCCTTACGGCCTTTTGCAACCCTTCGCTGATGGCTTGAAACTTTTCATGAAAGAGCCCGTCCGACCCTCCACCTCCTCGCCCGCCTTATTCTTAATTACCCCTATTATAGCCCTTACCTTAGCCCTAACCCTCTGGGCCCCCCTTCCTATGCCTTTTCCCATCACCGACCTAAACTTAGGCATTTTATTTATTTTAGCACTATCGAGCCTGGCAGTATATTCTATTCTTGGCTCCGGATGGGCCTCCAATTCTAAATATGCATTGATTGGTGCTCTTCGAGCAGTCGCCCAAACCATCTCTTATGAAGTGAGCTTGGGCCTTATTCTTCTTAACACAATTGTCTTTACAGGGGGTTTTACTCTTCAAACCTTCAGCACCGCACAAGAAGCCACCTGATTACTTCTACCAGCATGACCACTAGCAGCCATGTGATATATCTCCACGCTCGCGGAAACTAACCGGGCCCCTTTCGACTTAACTGAAGGAGAGTCCGAACTAGTGTCTGGCTTCAACGTAGAATATGCCGGCGGACCTTTTGCCCTTTTTTTTCTGGCAGAATACGGTAACATTTTACTTATAAATACCCTCTCAGCAGTACTATTTCTAGGCTCTTCAACCTACCACAGCTTTCCAGAACTAACCGCGACCTTATTAATGCTTAAAGCCACCCTCCTTTCAGTCGTATTTTTATGAGTGCGAGCATCTTACCCTCGGTTTCGATACGATCAACTAATGCATTTAATTTGAAAAAACTTTTTACCTCTGACCCTAGCACTAGTTATTTGACACCTTTCTCTTCCGATCACGTTGAGCGGCCTTCCCCCTCAACTTTAACTCAGGAAATGTGCCTGAAAAAGGGTCACTTTGATAGGGTGAATAATGAGGGTTAAAGCCCCTCCATCTCCTTAGAAAGAAGGGGTTTGAACCCTACCTGAAGAGATCAAAACTCTTAGTGCTTCCACTACACCACTTCCTAGTAAAGTCAGCTAATAAAAGCTTTTGGGCCCATACCCCAAATATGTTGGTTAAAATCCTTCCTTTGCTAATGAATCCTTACGTCCTTTCAATTCTACTTATAGGTTTAGGCCTCGGCACTACAGTCACATTCGCTAGCTCACACTGACTATTAGCATGAATAGGCCTTGAAATAAACACCCTCGCCATTTTGCCGTTAATAGCACAACATCACCACCCCCGAGCCGTTGAAGCCACCACCAAGTATTTTTTAATTCAATCGGCAGCCGCAGCAACCATCTTATTTGCCAGCTCAACAAACGCCTGACTTTCGGGCCAGTGGGACATCATAAGTATTAATCACCCTCTTCCAACCGTCATAATTACAGTCGCTCTGTCCTTAAAACTAGGCTTGGCCCCTCTTCACGCGTGACTTCCCGAAGTAATTCAAGGCCTGGACTTAACTACGGGCTTAATCCTCTCCACATGACAAAAACTCGCACCCTTTGCCCTCCTCGTTCAAATCTTCCCCGACACCCCCCTTCTCATCACTTCTCTAGGACTTCTTTCAATATTAGTTGGGGGATGAGGGGGTTTAAACCACACACAGCTCCGCAAAGTGCTCGCATATTCTTCGATCGCCCACTTAGGCTGAATAATAGTAATTATGCAATTCTCCACCCCCCTTACAATTCTTGCTTTATCAACATACATTGTCATAACATCATCTACTTTTCTAATCTTTAAACTCCTTAAATCCACAGATATGAACAGCCTGGCAACATCTTGAGCTAAAACCCCCTCCATTACAGCCCTAGCACCTTTAGTGCTATTATCCTTAGGCGGACTCCCTCCCCTCTCGGGCTTTATGCCAAAATGACTAATTATTCAGGAGTTAACTAAGCAAGACCTAGCCCTAGTTGCGACCTTAGCCGCCCTCTCTGCGCTACTCAGCCTTTTCTTTTACCTACGCATTTGTTACTCCCTCACATTTACCTCCTCTCCTAATAATCTCATGGGAACACCCCCCTGACGACTAGTAACAAAGCAAGTATCACTTCCCCTGGCTATAACAACCGCCCTCTCTATTCTCCTACTCCCGGTTACCCCTGCAATCTTATCAGTAGTTCTCCCCTTGTAAAGAGGCTTAGGATAGTATTAAGACCAAGGGCCTTCAAAGCCCTAAGCGGGAGTGAAAGCCCCCCAGCCTCTGTAAGACCTACGGGACACTAACCCACATCTTCTGTATGCAAAACAGACACTTTAATTAAGCTAAAGCCTTCCTAGGTGGGTAGGCCTCGATCCTACAATCTCTTAGTTAACAGCTAAGCGCCTAAACCAGCGGGCATCCATCTACCTTTCCCCCGCCTTGCCGAGAAAAAAAGGCGGGGGAAAGCCCCGGCAGGGTATTAGCCTGCTACTTAAGATTTGCAATCTAATGTGTTAACACCTCGGAGCTGGTAAGAAGAGGACTTTAACCTCTGTCTATGGGGCTACAATCCACCGCTAAACGCTCAGCCACCTTACCTGTGGCAATCACACGTTGATTTTTCTCAACTAATCACAAAGACATCGGCACCCTATATCTAATCTTTGGTGCCTGGGCGGGAATAGTAGGGACGGCCTTAAGTCTACTCATTCGGGCAGAATTAAGTCAACCAGGCTCCCTATTAGGAGACGACCAGATCTATAACGTAATTGTAACTGCACATGCTTTCGTAATAATTTTCTTTATAGTAATGCCAATTATAATTGGAGGGTTCGGCAACTGATTAATTCCTTTAATGATCGGAGCTCCCGACATGGCCTTCCCCCGGATAAATAATATAAGCTTTTGACTCCTGCCCCCTTCTTTCCTTCTATTATTGGCCTCATCTGGTGTAGAAGCTGGTGCCGGGACAGGATGAACCGTATATCCCCCCTTGTCCGGTAATTTGGCACACGCAGGGGCCTCCGTAGATTTAACCATTTTCTCTCTTCACCTGGCCGGAATCTCTTCTATTCTAGGGGCCATTAATTTCATTACAACTATTATTAATATAAAACCTCCAGCCATCTCCCAATATCAAACCCCTTTATTTGTCTGAGCTGTTCTAATTACCGCAGTATTACTCCTACTCTCTCTTCCTGTTCTAGCTGCAGGTATCACTATGCTTCTCACAGATCGAAACCTAAATACAACATTTTTCGACCCCGCAGGAGGGGGGGACCCCATTCTTTATCAACATTTATTCTGATTCTTTGGGCATCCTGAAGTCTACATTCTGATTTTGCCCGGCTTCGGAATGATTTCTCACATTGTAGCATATTACTCAGGCAAAAAAGAGCCGTTTGGCTACATGGGAATAGTATGAGCTATAATAGCAATTGGCTTACTGGGGTTTATCGTATGAGCCCATCATATGTTCACTGTAGGGATGGACGTGGACACTCGAGCTTATTTTACATCCGCCACTATAATTATCGCAATTCCTACAGGAGTTAAAGTGTTTAGTTGACTAGCTACCTTGCATGGGGGCTCAATCAAATGAGAGACCCCTCTGTTATGAGCTCTAGGCTTTATTTTCTTATTTACTGTCGGAGGTTTAACAGGAATTGTTTTAGCCAACTCATCTCTGGACATTATACTTCATGACACATACTATGTTGTAGCCCACTTCCACTATGTCCTCTCTATAGGAGCAGTCTTTGCCATCATGGGGGCATTCGTTCACTGATTCCCCCTATTCTCAGGCTACACCCTTCACAATACGTGAACAAAAATCCACTTCGGAGTTATGTTTGTAGGTGTAAACCTCACCTTTTTCCCTCAGCACTTCTTAGGATTGGCGGGAATACCTCGACGATATTCAGATTACCCTGACGCATACACACTGTGAAATACTATCTCATCCCTGGGGTCACTAATCTCCCTTATTGCTGTAATTATATTCCTATTTATTATCTGGGAAGCATTCGCGGCAAAACGTGAAGTCTTATCAGTTGAACTAACAGCCACAAACGTAGAATGACTGCACGGGTGTCCTCCCCCTTACCATACATTTGAAGAACCTGCATTCGTTCAAATTCAGCAATCCAAATTTTAATCGAGAAAGGAAGGAGTCGAACCCCCATAAACTGGTTTCAAGCCAGCCACATAACCGCTCTGTCACTTTCTTCTCTAAGTTAATAAGATTCTAGTTAAAAGAATAACGCTGCCTTGTCAAGGCAAAATTGTGGGTTAAAGCCCCACGTATCTTGCTTATGGCACATCCATCTCAACTAGGATTCCAAGATGCAGCTTCACCCGTTATAGAAGAACTTCTCCATTTTCATGACCATGCATTAATAATTGTTTTCTTAATCAGCACCCTTGTTCTTTACATTATTGTGGCTATGGTAACCACCAAGCTAACAAATAAGTTCATTCTGGACTCCCAAGAAATTGAAATCATCTGAACCTTACTACCAGCAATTATCCTAATTCTGATCGCCCTACCCTCCCTTCGCATTCTCTACCTCATGGATGAAATCAATGACCCCCACCTCACAATTAAAGCCATGGGACATCAATGATACTGAAGCTACGAATATACGGATTATGAAGACCTGGGGTTCGACTCATATATGGTCCCTACACAAGATCTCGCCCCTGGTCAATTTCGACTACTTGAGACAGACCATCGCATGGTTATTCCTGTTGAGTCCCCCATCCGAGTTCTTGTCTCCGCCGAGGATGTCTTACATTCATGAGCCGTCCCGAGCCTCGGAGTAAAAATGGACGCCGTCCCCGGCCGCCTAAATCAAACAGCCTTCATTACTTCCCGACCAGGTGTGTTTTATGGACAATGCTCAGAGATTTGCGGAGCTAATCATAGCTTTATACCCATTGTAGTGGAAGCTGTTCCTCTAGAACACTTCGAGAACTGGTCTTACCTAATACTTCAAGATGCCTCACCAGGAAGCTAAAAGGGAATAGCATTAGCCTTTTAAGCTAAAAATTGGTGACTCCCGCCCACCCCTGGTGACATGCCTCAGTTGAACCCCGCACCCTGATTTGCTATTATAGTATTCTCGTGACTAGTTTTCCTAGCCGTTATTCCACCTAAAGTTCTAGCTCACCATTTTCCCAATGACCCCGCCCCACAGAGCGTAAAAAAATCAAAAACAGAGACTTGACCCTGACCATGACTTTAAGCCTCTTTGATCAATTTATGAGCCCTACACTTCTAGGGGTGCCTCTTATCGGACTCGCCCTAACATTGCCATGAGTCCTTTACTTCCGACCCGGTGCCCGATGACTTAATAACCGCTTGATTACCCTTCAATCTATATTCATAAACTGGTTTGTAAAACAAATCTTTCAACCAATAAGCTTAGGCGGACACAAATGGGCCGCTCTCCTCATATCTTTAATACTATTTTTAATTACCTTAAATATGCTAGGCCTGCTGCCTTACACATTTACTCCAACAACGCAGCTGTCACTTAATATAGCCTTTGCAGTTCCACTTTGACTAGCAACTGTCATTATTGGAATACGAAACCAGCCAACACATGCCCTTGGTCACCTTCTCCCTGAAGGAACTCCTACCGCCCTAATCCCGGTTTTAATCGTGATTGAAACAATTAGCCTTTTTATTCGACCCTTGGCCCTCGGTGTTCGACTTACCGCAAACTTGACAGCCGGACACCTTCTAATTCAACTAATTGCAACTGCGGCCTTTGTTCTTTTCCCTATAATACCTACAGTAGCTGCTCTTACCTCTGTCTTACTATTCTTGCTAACCCTGCTAGAAGTCGCCGTGGCCATAATCCAAGCCTATGTATTTGTACTTCTTTTAAGCCTTTATCTACAAGAAAACGTCTAATGGCCCATCAAGCACATGCATATCATATAGTTGACCCAAGCCCTTGACCCCTCACAGGCGCAGTAGCCGCCCTTCTACTTACGTCTGGAACAGCAATCTGAATACACTTTAACTCCACAGTTCTCATGTCCCTTGGACTTGTCCTGCTACTACTAACCATATATCAATGATGGCGAGACATTATCCGAGAGGGTACCTTTCAAGGTCATCATACACCCCCTGTTCAAAAGGGCCTTCGGTACGGGATAATTCTATTTATTACCTCAGAGGTCTTCTTTTTCCTAGGTTTCTTCTGAGCATTTTATCACTCAAGCCTAGCCCCAACCCCCGAACTTGGTGGGTGTTGACCACCTATGGGTATTACAACACTGGACCCCTTTGAAGTCCCCCTTCTCAATACTGCTGTCCTTCTCGCCTCCGGTGTCACGGTCACTTGAGCTCACCATAGTATTATGGAGGGGCAGCGAAAACAAGCAATTCAATCCTTAACACTCACAATTCTACTGGGGTTCTACTTTACATTCCTTCAAGCAATAGAGTACTACGAGGCACCCTTCACCATTGCAGATGGCGTCTATGGCTCTACATTTTTTGTGGCAACGGGGTTTCATGGCCTCCATGTAATTATTGGGTCAACATTTCTGGCAGTCTGCCTCTTACGACAAGTCCAATTCCACTTTACATCGGAACATCACTTCGGATTTGAAGCTGCAGCATGATACTGACACTTTGTAGACGTAGTCTGACTATTCTTATATATCTCTATCTACTGATGAGGCTCATATCTTTCTAGTATTAAAAAGTACAAGTGACTTCCAATCACTCAGTCTTGGTTAGACTCCAAGGAAAGATAATGAACTTAGTACTAGTCATTATTTGCATCTCATTAGCCCTCGCCGCACTGCTCGCAACTGTTTCATTTTTTCTCCCACAAATAACCCCTGATTATGAGAAACTCTCACCGTATGAGTGCGGCTTTGATCCAGTGGGATCCGCCCGTTTGCCATTCTCCATTCGCTTTTTTCTAGTCGCAATCCTATTTCTCCTCTTCGACTTAGAAATTGCCTTACTTCTCCCCCTTCCCTGAGGAGACCAACTCCCCTCCCCTCTGACAACTTTCTTCTGAGCTTCTGCTATCCTTATACTACTAACTCTAGGGTTAATCTATGAATGACTTCAAGGGGGCCTAGAGTGGGCAGAATAGGTACTTAGTTTAATAAAAACATTTGATTTCGGCTCAAAAACTTATGGTTTAAGTCCATATTTACCTGATGACCTTAACTCACTATGCATTCTCGTCAGCCTACTTTGTCAGCTTCATGGGTCTAATTTTTTACCGAAAGCATCTTCTCTCCGCCTTACTTTGCTTAGAAGCCATAATACTTATTCTTTTTATTTCACTATGCCTGTGAGGCCTAGTCTTAGCCTCAAGTGCATTTTCGGCAGGCCCAATGATCTTACTTGCTTTCTCAGCATGTGAAGCAAGTGCAGGCCTAGCACTGCTTGTAGCAATAGCTCGAACCCACGGGACTGACCGTTTAAAAAACCTTAGCCTACTCCAATGTTAATAATTCTTATTCCTACTGTTATGCTTCTACCCACAATCTGACTAAGCCCCGCTAAATACCTGTGGTCCTCAGCACTTGGCCATAGCATAATAATTGCTCTTATAAGCCTCTCCTGATTTAGCCTCCCGGGGGAGGTTGGCTGATCTTCCCTTAACACTTTTATAGCAACAGACCCTCTCTCTACCCCCCTTCTCGTACTTACTTGCTGACTTCTACCCTTAATAATTCTTGCGAGCCAAAACCATATAGCCCAAGAACCTACCAATCGCCAGCGAACCTATATCTCTCTCCTTACTTCCCTTCAAATCTTCTTAATCTTAGCATTTGGAGCAACCGAGATAATTATGTTCTATATTATATTTGAAGCGACCTTAATTCCCACACTCGTAATTATCACACGATGAGGGAACCAAACAGAGCGATTAAACGCAGGTATTTACTTTTTATTTTATACCTTAGCCGGCTCTTTACCACTACTAGTGGCCCTCCTTCTACTTCAGACCTCGACAGGAACTCTTTCTTTTCTAACCACTCAATTTTTTCCCCCCTTACAACTGCATACAGAAGCAAGTAAATTCTGGTGGGCGGGCTGTTTACTAGCATTCTTAGTAAAAATGCCGCTATATGGGGCACACCTTTGACTTCCAAAAGCTCACGTCGAAGCCCCCATCGCCGGGTCAATAGTCCTTGCAGCCGTTCTTTTAAAACTAGGGGGTTACGGTATGATACGAGTCATTATTATCTTAGAACCCCTAACGAAACAACTCAGCTACCCCTTTATTGTTCTTGCCCTGTGGGGCGTTGTAATAACTGGCTCAATCTGCCTCCGACAAACAGACCTTAAATCACTAATCGCTTACTCCTCAGTAAGCCACATGGGCCTTGTCGCAGCAGGCATCCTGATCCAAACTCCTTGGGGGTTTACAGGAGCATTAATCCTTATAATTGCCCATGGCTTAACCTCCTCCGCCCTATTCTGTTTAGCCAACACTAACTATGAGCGAACACATAGCCGAACCATACTTTTAGCCCGGGGTCTACAAATGGTCCTTCCTCTTTTAGCAACTTGGTGGTTTCTACTTACCCTCGCCAACCTAGCACTCCCTCCGCTACCCAACCTCATAGGAGAGCTTATGATTATCTCATCCTTGTATAACTGGTCAAACTGGTCTCTAATCCTGACCGGAGCGGGAGTACTAATTACCGCTAGCTACTCTCTTCATATATTCCTAACCACTCAACGCGGCCCTATTACTAACCCCGTCTTGGCAATTGAACCAACCCACACACGAGAACATCTCCTCATAATTCTTCACCTTCTTCCTCTCCTCCTTCTAATTTTAAAACCCTGCTTGATCTGGGGCTGAACAGTTTGTAGGCGTAGTTTAAATAAAGCGCTAGATTGTGATTCTAGAAATAAGAGTTAAACCCTCTTCACCCACCGAGAGGGGTCGCCGTGACAGCAAGAACTGCTAATTCTAGCCCCTTTGGTTAAAATCCGAAGCCCACTCGAACAGGCTTCTAAAGGATAACAGCTCATCCGTTGGTCTTAGGAACCAAAAACTCTTGGTGCAACTCCAAGTAGCAGCTATGCACTTTACAACAATGATTCTCTCCTCAAGCCTAATAACAATTTTCCTTATTCTCATCCTTCCAGTCCTAGGTACACTAAACCCTAACCCCACGGGGGGCCTGTGAGCCACAAAAAACGTTAAAACGGCAGTTAAGATAGCCTTTTTTGTAAGTCTTTTGCCTCTTTTTATCTTTCTTAATGAAGGGGTAGAGACTGTTATAACAAACTGAAAATGAATAAATACTCTAATGTTTGAAATTAATATCAGCTTTAAATTTGACCTCTACTCCGTAGTATTTACCCCTGTGGCCCTCTACGTAACATGATCAATTTTAGAGTTCGCATCTTGGTATATACACAGTGATCCCAACATAAACCGATTCTTTAAATATCTTCTAATCTTTCTTATTGCTATGGTCGTTCTGGTCACAGCCAACAACATGTTCCAACTATTTATTGGCTGAGAAGGTGTTGGAATTATATCTTTCTTACTTATTGGCTGGTGATTCGGGCGGGCTGACGCCAACACTGCGGCCCTCCAGGCCGTAGTTTATAACCGAGTTGGTGATATCGGCCTTATTCTAGCAATAGCATGAATAGTAGCAAACCTAAACTCATGAGAAATACAACAGCTCTTTTCTATGTCTAAAGGCCATGATATAACCCTTCCTTTATTAGGCCTAGTACTGGCCGCTACCGGAAAGTCCGCCCAATTTGGACTTCACCCCTGGCTCCCCTCAGCCATAGAGGGTCCAACACCGGTCTCTGCCCTCCTGCATTCTAGCACCATGGTAGTTGCTGGTATTTTTCTTCTTATTCGCCTCAGCCCCTTAATGCAAGAAAGCCCATTAATTCTCTCAACATGCCTTTGCCTGGGGGCCCTAACTACCGTCTTTACTGCAACATGTGCCCTTACCCAAAATGACATTAAAAAAATCGTTGCATTTTCTACATCAAGTCAATTAGGACTAATGATAGTTACCATCGGACTAGGCCAGCCCCAGCTCGCCTTTCTTCATATCTGCACCCACGCCTTCTTTAAAGCAATACTTTTCTTATGTTCCGGCTCCATCATTCATAGCCTTAATGATGAGCAAGATATCCGAAAAATAGGCGGACTTCACAAGGTACTTCCACTGACCTCTTCTTGTCTAACCATTGGCAGCCTAGCTCTAACAGGAGTCCCCTTTTTAGCAGGCTTCTTTTCCAAAGACGCCATCATTGAAGCTATAAATACATCCTACCTTAACGCCTGAGCCCTAATTTTAACGCTTCTAGCTACATCATTCACCGCAGTTTACAGTCTCCGAGTCGTATTCTTTGCCTCTATGGGCCACCCGCGTTTTAATCCAGTCTCCCCAATTAATGAAAATAACCCTACAGTGATAAACCCCATTAAACGACTCGCTTGGGGAAGCATTTTGGCAGGGTTGCTAATTACGACCAATATTGTTCCACTTAAAACCCCCGTTTTAACCATGCCTTTCACCTTAAAAATGGCCGCACTGACTGTAACAATTATAGGACTACTCACAGCCTTAGAACTAGCGTCTCTCACGTCCCAACAATTTAAAATCAAACCCTTATCTTCTACTCACCACTTCTCAAATATATTAGGATTTTTCCCCAGTGTAGTCCATCGACTAGTCCCAAAAACTGGCCTGATTCTAGGACAACTAGTTGCCAATCAGACAGTTGACCAAACCTGACTAGAGAAAACAGGACCAAAAATAGTAACCTCCGTTAACCTTCCAATAGCTACTTCAATTAGCAGCCTACAGCAGGGTGTAATTAAGACCTACTTCTTATTATTTTTCTTCACCATAATACTGGCAATTCTCATCTTTGTCGTCTAACTGCCCGTAAGGTCCCCCGACTTAGCCCCCGAGTTAACTCTAGAACTACAAAAAGCGTCAGTAATAAAACTCATCCCCCAAGCATTAAAACTCCTCCTCCTGAAGAATATATCAGAGCAACCCCACCGAAATCCCCCCGAAAGAGCATGAATTCACTAAACTCGTCAGCAGTTACCCATGACCCCTCATACCAGCCCTCAGAGAAAAAAACAGAGATAGACGCGACCAGGAACACATACACTGACATAAGAAGCAAAACGGGTCAACTTCCCCACCCCTCAGGATAAGGCTCCGAAGCCAGCGCTGCTGAGTACGCAAACACAACTAACATCCCACCTAAATAGATCAAAAACAAAATCAGAGATAAAAATGAACCCCCATGCCCTACTAAAATGCCACAGCCCATTCCTGCTACTGTAACAAGACCTAAAGCAGCAAAGTAGGGTGACGGGTTCGAGGCCACGGCCGCTAGACCTAAGACCAAACCAACTAATAATAAATAAGTCATATAAACCATAATTCTTGCCAGGATTTTAACCAGGGCCTGCGACTTGAAAAACCACCGTTGTACTCAACTACAAGAACCTAATGGCCAATCTTCGAAAAACCCATCCCCTATTAAAAATCGCAAACGATGCCCTCGTTGATCTCCCAGCCCCGTCGAACATTTCAGTTTGATGAAACTTCGGGTCTCTTCTAGGACTTTGTTTGGCCGCCCAAATCGTTACGGGCCTTTTCCTTGCAATACATTATACATCAGACATTGCCACAGCATTTTCATCTGTAGCACATATTTGTCGTGATGTCAACTACGGCTGACTAATCCGGAACATGCATGCAAACGGTGCTTCCTTTTTCTTCATTTGCATCTACCTGCACATCGGACGGGGCTTGTATTATGGATCATACTTATATAAAGAGACATGAAATGTAGGTGTTGTCCTTCTCCTCCTAGTGATAATGACTGCTTTCGTAGGCTACGTCCTACCCTGAGGACAAATGTCATTCTGAGGGGCCACCGTCATTACCAACCTTTTATCAGCCATTCCTTACATTGGAAACGCCCTAGTTCAATGGATCTGAGGCGGATTTTCAGTAGACAACGCCACCCTTACCCGGTTCTTTGCCTTCCATTTCCTCCTCCCCTTTGTAATTGCTGCTGCTACAATTGTACATCTTATTTTCCTGCACGAGACAGGGTCGAATAACCCAACGGGTTTAAACTCAGACTCTGACAAAGTGTCTTTTCACCCCTACTTTTCTTATAAAGATCTCCTAGGCTTTGCTGCCCTGCTAGTAGCCCTTATCTCTTTAGCCCTCTTCTCCCCTAATCTACTTGGAGACCCTGATAACTTTACCCCTGCTAATCCTTTAGTAACTCCACCTCACATCAAACCTGAGTGATACTTCCTGTTCGCTTACGCCATTCTACGATCCATCCCAAACAAGCTTGGTGGGGTTCTAGCCCTATTAGCCTCTATTCTAGTTCTCTTTCTTGTCCCTATCCTGCACACATCAAAACAACGAAGCCTAACATTCCGACCCCTAACCCAGTTCCTCTTCTGATTGCTAGTCGCCGATGTAATAATTTTAACCTGAATCGGAGGTATGCCTGTAGAACACCCTTACATTATCATTGGGCAAGTCGCATCCTTCATTTATTTTTCCCTTTTTCTAGTTATGGCACCTATGGCCGGCCTACTAGAAAACAAAGTCTTAAAATGACAATGCATTAGAAGCTCAGATGAGAGAGCACCGGTCTTGTAAGCCAGAGGTCGAAGGTTCAAGTCCTTCCTAGTGCTCAGAGAGAAGGGATTCTAACCCCTGCCCCTGGCTCCCAAAGCCAGGATTCTTAGCTAAACTACTCGCTGATTTTCATATACCAGTTTTGCAATCCAGAGCGCATCACTTTTGCCACCAGCGTTAAATTAACGTTGCACAAACGTTGCATCAGCACCCCATGGACACTAAATGACGCGAGGGCGTTAAATAGACACCCTCTACCTCTAGCACCCTTTTAACGATTTCACTTTTTTTTTTTTTTTTGTTTAACGATTACGTTTTTTTTTGCGTTCCCGGACTCTGCCAGATTTCGACCGAAGACTGCCAGAATCCGCTCAAAATCCGCTCAAATACCAATATGTATTATCCCCATAAGTGGTTTAAACCATTTTTGCCTAGTACACACTGACCATGCAAGTCAATTATATTTACCCCGCGCTCCAGGCCGCAGTACATACACCTACAGTTGGTGTATTTAGCACAAGTGTGCCTCAGCTAGTTTCAAGTCACCCACATCCTTCCTTTAATTGTTACTTAATGTAGTAAGAGCCCACCATCAGTTGATTCCTTAATGTCAACGGTTCTTGAAGGTGAGGGACAAAAATCGTGGGGGTTTCACCTCTTGAATTATTCCTGGCATTTGGCTCTACATCTCAAGGCCATACATTTCTCGTCTCTCACACTTTCACTGGCCCTGACATTGGTTAATGGTGGAGTACATACTCCTCGTTACCCCCCATGCCGGGCGTTCTTTCTAATGGACAACGGGTTTTCCTTTTTTTTTCCTTTTCACTTGGCATTTCACAGTGCATACAAACCTTGTTGACAAGGTTGAACATTTAGAAATCGGCCGCAAAGAATATTGGTGAATTATTTAAAGATATTAACAGATGAATTGCATAACTGATATCAAGAGCATAAATAACCAAATGAAACTAGGAACGTTTCTATAATATGACCCCCCGGCTTCCGCGCGTCAAACCCCCCTACCCCCCTAAACTAGTAAGAAGGCTATTATTCCTGCAAACCCCCCGGAAACAGGAAACCCCCTACTAGCATTTTAGCCCGCCCAAATTTGTGTGTATTTACATTATTTGTAATATTGCAAAA

>JWM 20

GCTAGTGTAGCTTAACTAAAGCATAACACTGAAGATGTTAAGACAAACCTTAGATTGGTTTCACGAGCACAAAAGTTTGGTCCTGACTTTACTATCAACTTTAGCTAAACTTACACATGCAAGTATCCGCAATCCCGTGAGAATGCCCTACAGTTTCCTTAAAGGAAACAAGGAGCTGGTATCAGGCTCAATTACTCCCGCCCATGACACCTTGCTTAGCCACACCCCCAAGGGAACTCAGCAGTGATAGACATTAAGCAATAAGTGAAAACTTGACTTAATTAAAGCTAAGAGAACCGGTTAAACTCGTGCCAGCCACCGCGGTTATACGAGCGGTTCGAGCTGATAGACTACGGCGTAAAGCGTGGTTAATAAGAATAAAACTAAAGTCGAATGTTTTCAAAGCTGTTATACGCACTCGAAAATTAGAAGACCAGAAACGAAAGTGACTTTAACCCTATGAACCCACGAAAACTATGAAACAAACTGGGATTAGATACCCCACTATGCATAGCTGTAAACTTTGATGAGCCATTACATTATCATCCGCCTGGGTACTACGAGCATCAGCTTAAAACCCAAAGGACTTGGCGGTGCTTTAGACCCACCTAGAGGAGCCTGTTCTAGAACCGATAACCCCCGTTAAACCTCACCCTCTCTTGTTTTTCCCGCCTATATACCGCCGTCGTCAGCTTACCCTGTGAAGGTCTAATAGTAAGCACAACCAGTTATACTCAAAACGTCAGGTCGAGGTGTAGCATATGAGAGGGGAAGAAATGGGCTACATTCCTTGTTTCAAGGAAAACGGATAACATAATGAAAGGTACGTTAGAAGGAGGATTTAGCAGTAAGCAGCAAATAGAGTGTTCTGCTGAAACTGGCCCTGAAGCGCGCACACACCGCCCGTCACTCTCCCCAACTCCGAGTTAAAAACCATATATAAACCTTTGAAGGAACAAAGGGGAGGCAAGTCGTAACATGGTAAGTGTACCGGAAGGTGCACTTGGATAAATCAGAGTATAGCTAAGAAAGAAAAGCATCTCCCTTACACCGAGAAGTCATCCGTGCAAATCGGATTACCCTGACTCTAACAAGCTAGCCCAAAACCTTAACTTAAAAATCAAATATTTCTAGTAATTAATAAACCAAACACATTAAATAAATCATTTTTCCCCCTGAGTATGGGAGACAGAAAAGGATAAAGGAGCTATAGACAAAGTACCGCAAGGGAAAGCTGAAAGAGAAATGAAACAAACCAGTAAAGAAGAACAAAGCAGAGATTAACCCTTGTACCTTTTGCATCATGAATTAGCCAGTTTAATCAAGCAAAGAGCACTGTAGTTTGAACCCCCGAAACTTAGTGAGCTACTTCAAGACAGCCTATGAAATAGGGCAAACCCGTCTCTGTGGCAAAAGAGTGGGAAGATCTTCAAGTAGAGGTGACAGACCTATCGAACTAAGTTATAGCTGGTTGCTCGTGAAATGAATAGAAGTTCAGCCTTTTGCTTTCTAAATTTCGATTTAGCACTACTTAGCCTAAATGACTAGAAAACAAAAGAGTTAGTCAAAGAGGGTACAGCCTGTTTGATAAAAGATACAACTTTACTAGGAGGATAAGAATCATAATTTTAAAGGTTTAATGCCCAGGTGGGCCTAAAAGCAGCCACCCTAATCAATAGCGTTAAAGCTTAAGCATAAAACACACCTACAATTCTGATAAATCAGTTTTAATCCCCTAAAGTTAACGAGCTATTTCATACCTTATGAAAGAAATTATGCTAGTATGAGTAATAAGAAGTTACGAACTTCTCCCTGCACACGTGTAAATCGGAACGGACAAACCACCGAATCTTAACGGCCCCAGTCAAAGAGGGGATGTCGGATAAAAAAAAGAACAAGAAACTCCCGACAAAACCACCGTTAACCCCACACCGGAGTGCTCCCTGGGAAAGACAAAAAGGGACAGAAGGAACTCGGCAAATATGCTCAAGCCTCGCCTGTTTACCAAAAACATCGCCTCTTGTAAAAGTTAAATAAGAGGTACCGCCTGCCCTGTGACTAGTAGTTTAACGGCCGCGGTATTTTGACCGTGCAAAGGTAGCGCAATCACTTGCCTTTTAAATGAAGGCCTGTATGAATGGCACGACGAGGGCTTAACTGTCTCCTCTCCCTAGTCAATGAAATTGATCTCCCCGTGCAGAAGCGGGGATAATAACATAAGACGAGAAGACCCTGTGGAGCTTTAGACTATGAGCAGACCATGTCAAGAATAACAAACAAGTAAATTAAACAGATTGGTCCCTGCTTCTCTGTCTTTGGTTGGGGCGACCGCGGGATAATAAAAAGCTCCCACGAGGATTGAGAACCCTTATCTTATAACCAAGAGCTTCTCCTCTAAGTAACAGAACATCTGACCTTAATGATCCGGCCTGGCCGATCAACGGACCGAGTTACCCCAGGGATAACAGCGCAATCCTCTTTTAGAGTCCATATCGACAAGAGGGTTTACGACCTCGATGTTGGATCAGGACATCCTAATGGTGCAGCCGCTATTAAGGGTTTGTTTGTTCAACAATTAAAGTCCTACGTGATCTGAGTTCAGACCGGAGTAATCCAGGTCAGTTTCTATCTATGACGTACTCTCTTCTAGTACGAAAGGACCGAAGAAAGAAGGCCTATGAAAAGTTATGCCTTAGTCTCACCTTATGAAGAAAACTAAATAAGACAAGAGGTTACACCCCTTAGTCATAGAAAATGACATGTTAAGGTGGCAGAGCCCGGATATTGCAAAAGACCTAAGCCCTTTCCACAGAGGTTCAATTCCTCTCCTTAACTATGTTCTCAACAATATTAAGCTTCATTATTAATCCCCTAATTGTTATGGTTTTTGTTTTGCTGGCAGTAGCCCTCTTAACCTTGGTAGAGCGTAAAGTGCTAAGCTACATGCAACTTCGTAAAGGCCCAAATGTTGTTGGCCCTTACGGCCTTTTGCAACCCTTCGCTGATGGCTTGAAACTTTTCATGAAAGAGCCCGTCCGACCCTCCACCTCCTCGCCCGCCTTATTCTTAATTACCCCTATTATAGCCCTTACCTTAGCCCTAACCCTCTGGGCCCCCCTTCCTATGCCTTTTCCCATCACCGACCTAAACTTAGGCATTTTATTTATTTTAGCACTATCGAGCCTGGCAGTATATTCTATTCTTGGCTCCGGATGGGCCTCCAATTCTAAATATGCATTGATTGGTGCTCTTCGAGCAGTCGCCCAAACCATCTCTTATGAAGTGAGCTTGGGCCTTATTCTTCTTAACACAATTGTCTTTACAGGGGGTTTTACTCTTCAAACCTTCAGCACCGCACAAGAAGCCACCTGATTACTTCTACCAGCATGACCACTAGCAGCCATGTGATATATCTCCACACTCGCGGAAACTAACCGGGCCCCTTTCGACTTAACTGAAGGAGAGTCCGAACTAGTGTCTGGCTTCAACGTAGAGTATGCCGGCGGACCTTTTGCCCTTTTTTTTCTGGCAGAATACGGTAACATTTTACTTATAAATACCCTCTCAGCAGTACTATTTCTAGGCTCTTCAACCTACCACAGCTTTCCAGAACTAACCGCGACCTTATTAATGCTTAAAGCCACCCTCCTTTCAGTCGTATTTTTATGAGTGCGAGCATCTTACCCTCGGTTTCGATACGATCAACTAATGCATTTAATTTGAAAAAACTTTTTACCTCTGACCCTAGCACTAGTTATTTGACACCTTTCTCTTCCGATCACGTTGAGCGGCCTTCCCCCTCAACTTTAACTCAGGAAATGTGCCTGAAAAAGGGTCACTTTGATAGGGTGAATAATGAGGGTTAAAGCCCCTCCATCTCCTTAGAAAGAAGGGGTTTGAACCCTACCTGAAGAGATCAAAACTCTTAGTGCTTCCACTACACCACTTCCTAGTAAAGTCAGCTAATAAAAGCTTTTGGGCCCATACCCCAAATATGTTGGTTAAAATCCTTCCTTTGCTAATGAATCCTTACGTCCTTTCAATTCTACTTATAGGTCTAGGCCTCGGCACTACGGTCACATTCGCTAGCTCACACTGACTATTAGCATGAATAGGCCTTGAAATAAATACCCTCGCCATTTTGCCGTTAATAGCACAACATCACCACCCCCGAGCCGTTGAAGCCACCACCAAGTATTTTTTAATTCAATCGGCAGCCGCAGCAACCATCTTATTTGCCAGCTCAACAAACGCCTGACTTTCGGGCCAGTGGGACATCATAAGTATTAATCACCCTCTTCCAACCGTCATAATTACAGTCGCTCTGTCCTTAAAACTAGGCTTGGCCCCTCTTCACGCGTGACTTCCCGAAGTAATTCAAGGCCTGGACTTAACTACGGGCTTAATCCTCTCCACATGACAAAAACTCGCACCCTTTGCCCTCCTCGTTCAAATCTTCCCCGACACCCCCCTTCTCATCACTTCTCTAGGACTTCTTTCAATATTAGTTGGGGGATGAGGGGGTTTAAACCACACACAGCTCCGCAAAGTGCTCGCATATTCTTCGATCGCCCACTTAGGCTGAATAATAGTAATTATGCAATTCTCCACCCCCCTTACAATTCTTGCTTTATCAACATACATTGTCATAACATCATCTACTTTTCTAATCTTTAAACTCCTTAAATCCACAGATATGAACAGCCTGGCAACATCTTGAGCTAAAACCCCCTCCATTACAGCCCTAGCACCTTTAGTGCTATTATCCTTAGGCGGACTCCCTCCCCTCTCGGGCTTTATGCCAAAATGACTAATTATTCAGGAGTTAACTAAGCAAGACCTAGCCCTAGTTGCGACCTTAGCCGCCCTCTCTGCGCTACTCAGCCTTTTCTTTTACCTACGCATTTGTTACTCCCTCACATTTACCTCCTCTCCTAATAATCTCATGGGAACACCCCCCTGACGACTAGTAACAAAGCAAGTATCACTTCCCCTGGCTATAACAACCGCCCTCTCTATTCTCCTACTCCCGGTTACCCCTGCAATCTTATCAGTAGTTCTCCCCTTGTAAAGAGGCTTAGGATAGTATTAAGACCAAGGGCCTTCAAAGCCCTAAGCGGGAGTGAAAGCCCCCCAGCCTCTGTAAGACCTACGGGACACTAACCCACATCTTCTGTATGCAAAACAGACACTTTAATTAAGCTAAAGCCTTCCTAGGTGGGTAGGCCTCGATCCTACAATCTCTTAGTTAACAGCTAAGCGCCTAAACCAGCGGGCATCCATCTACCTTTCCCCCGCCTTGCCGAAAAAAAAAGGCGGGGGAAAGCCCCGGCAGGGTATTAGCCTGCTACTTAAGATTTGCAATCTGATGTGTTAACACCTCGGAGCTGGTAAGAAGAGGACTTTAACCTCTGTCTATGGGGCTACAATCCACCGCTAAACGCTCAGCCACCTTACCTGTGGCAATCACACGTTGATTTTTCTCAACTAATCACAAAGACATCGGCACCCTATATCTAATCTTTGGTGCCTGGGCGGGAATAGTAGGGACGGCCTTAAGTCTACTCATTCGGGCAGAATTAAGTCAACCAGGCTCCCTATTAGGAGACGACCAGATCTATAACGTAATTGTAACTGCACATGCTTTCGTAATAATTTTCTTTATAGTAATGCCAATTATAATTGGAGGGTTCGGCAACTGATTAATTCCTTTAATGATTGGAGCTCCCGACATGGCCTTCCCCCGGATAAATAATATAAGCTTTTGACTCCTGCCCCCTTCTTTCCTTCTATTATTGGCCTCATCTGGTGTAGAAGCTGGTGCCGGGACAGGATGAACCGTATATCCCCCCTTGTCCGGTAATTTGGCACACGCAGGGGCCTCCGTAGATTTAACCATTTTCTCTCTTCACCTGGCCGGAATCTCTTCTATTCTAGGGGCCATTAATTTCATTACAACTATTATTAATATAAAACCTCCAGCCATTTCCCAATATCAAACCCCTTTATTTGTCTGAGCTGTTCTAATTACCGCAGTATTACTCCTACTCTCTCTTCCTGTTCTAGCTGCAGGTATCACTATGCTTCTCACAGATCGAAACCTAAATACAACATTTTTCGACCCCGCAGGAGGGGGGGACCCCATTCTTTATCAACATTTATTCTGATTCTTTGGGCATCCTGAAGTCTACATTCTGATTTTGCCCGGCTTCGGAATGATTTCTCACATTGTAGCATATTACTCAGGCAAAAAAGAGCCGTTTGGCTACATGGGAATAGTATGAGCTATAATAGCAATTGGCTTACTGGGGTTTATCGTATGAGCCCATCATATGTTCACTGTAGGGATGGACGTGGACACTCGAGCTTATTTTACATCCGCCACTATAATTATCGCAATTCCTACAGGAGTCAAAGTGTTTAGTTGACTAGCTACCTTGCATGGGGGCTCAATCAAATGAGAGACCCCTCTGTTATGAGCTCTAGGCTTTATTTTCTTATTTACTGTCGGAGGTTTAACAGGAATTGTTTTAGCCAACTCATCTCTGGACATTATACTTCATGACACATACTATGTTGTAGCCCACTTCCACTATGTCCTCTCTATAGGAGCAGTCTTTGCCATCATGGGGGCATTCGTTCACTGATTCCCCCTATTCTCAGGCTACACCCTTCACAATACGTGAACAAAAATCCACTTCGGAGTTATGTTTGTAGGTGTAAACCTCACCTTTTTCCCTCAGCACTTCTTAGGATTGGCGGGAATACCTCGACGATATTCAGATTACCCTGACGCATACACACTGTGAAATACTATCTCATCCCTGGGGTCACTAATCTCCCTTATTGCTGTAATTATATTCCTATTTATTATCTGGGAAGCATTCGCGGCAAAACGTGAAGTATTATCAGTTGAACTAACAGCCACAAACGTAGAATGACTGCACGGGTGTCCTCCCCCTTACCATACATTTGAAGAACCTGCATTCGTTCAAATTCAGCAATCCAAATTTTAATCGAGAAAGGAAGGAGTCGAACCCCCATAAACTGGTTTCAAGCCAGCCACATAACCGCTCTGTCACTTTCTTCTCTAAGTTAATAAGATTCTAGTTAAAAGAATAACGCTGCCTTGTCAAGGCAAAATTGTGGGTTAAAGCCCCACGTATCTTGCTTATGGCACATCCATCTCAACTAGGATTCCAAGATGCAGCTTCACCCGTTATAGAAGAACTTCTCCATTTTCATGACCATGCATTAATAATTGTTTTCTTAATCAGCACCCTTGTTCTTTACATTATTGTGGCTATGGTAACCACCAAGCTAACAAATAAGTTCATTCTGGACTCCCAAGAAATTGAAATCATCTGAACCTTACTACCAGCAATTATCCTAATTCTGATCGCCCTACCCTCCCTTCGCATTCTCTACCTCATGGATGAAATCAATGACCCCCACCTCACAATTAAAGCCATGGGACATCAATGATACTGAAGCTACGAATATACGGATTATGAAGACCTGGGGTTCGACTCATATATGGTCCCTACACAAGATCTCGCCCCTGGTCAATTTCGACTACTTGAGACAGACCATCGCATGGTTATTCCTGTTGAGTCCCCCATCCGAGTTCTTGTCTCCGCCGAGGATGTCTTACATTCATGAGCCGTCCCGAGCCTCGGAGTAAAAATGGACGCCGTCCCCGGCCGCCTAAATCAAACAGCCTTCATTACTTCCCGACCAGGTGTGTTTTATGGACAATGCTCAGAGATTTGCGGAGCTAATCATAGCTTTATACCCATTGTAGTGGAAGCTGTTCCTCTAGAACACTTCGAGAACTGGTCTTACCTAATACTTCAAGATGCCTCACCAGGAAGCTAAAAGGGAATAGCATTAGCCTTTTAAGCTAAAAATTGGTGACTCCCGCCCACCCCTGGTGACATGCCTCAGTTGAACCCCGCACCCTGATTTGCTATTATAGTATTCTCGTGACTAGTTTTCCTAGCCGTTATTCCACCTAAAGTTCTAGCTCACCATTTTCCCAATGACCCCGCCCCACAGAGCGTAAAAAAATCAAAAACAGAGACTTGACCCTGACCATGACTTTAAGCCTCTTTGATCAATTTATGAGCCCTACACTTCTAGGGGTGCCTCTTATCGGACTCGCCCTAACATTGCCATGAGTCCTTTACTTCCGACCCGGTGCCCGATGACTTAATAACCGCTTGATTACCCTTCAATCTATATTCATAAACTGGTTTGTAAAACAAATCTTTCAACCAATAAGCTTAGGCGGACACAAATGGGCCGCTCTCCTCATATCTTTAATACTATTTTTAATTACCTTAAATATGCTGGGCCTGCTGCCTTACACATTTACTCCAACAACGCAGCTGTCACTTAATATAGCCTTTGCAGTTCCACTTTGACTAGCAACTGTCATTATTGGAATACGAAACCAGCCAACACATGCCCTTGGTCACCTTCTCCCTGAAGGAACTCCTACCGCCCTAATCCCGGTTTTAATCGTGATTGAAACAATTAGCCTTTTTATTCGACCCTTGGCCCTCGGTGTTCGACTTACCGCAAACTTGACAGCCGGACACCTTCTAATTCAACTAATTGCAACTGCGGCTTTTGTTCTTTTCCCTATAATACCTACAGTAGCTGCTCTTACCTCTGTCTTACTATTCTTGCTAACCCTGCTAGAAGTCGCCGTGGCCATAATCCAAGCCTATGTATTTGTACTTCTTTTAAGCCTTTATCTACAAGAAAACGTCTAATGGCCCATCAAGCACATGCATATCATATAGTTGACCCAAGCCCTTGACCCCTCACAGGCGCAGTAGCCGCCCTTCTACTTACGTCTGGAACAGCAATCTGAATACACTTTAACTCCACAGTTCTCATGTCCCTTGGACTTGTCCTGCTACTACTAACCATATATCAATGATGGCGAGACATTATCCGAGAGGGTACCTTTCAAGGTCATCATACACCCCCTGTTCAAAAGGGCCTTCGGTACGGGATAATTCTATTTATTACCTCAGAGGTCTTCTTTTTCCTAGGTTTCTTCTGAGCATTTTATCACTCAAGCCTAGCCCCAACCCCCGAACTTGGTGGGTGTTGACCACCTATGGGTATTACAACACTGGACCCCTTTGAAGTCCCCCTTCTCAATACTGCTGTCCTTCTCGCCTCCGGTGTCACGGTCACTTGAGCTCACCATAGTATTATGGAGGGGCAGCGAAAACAAGCAATTCAATCCTTAACACTCACAATTCTACTGGGGTTCTACTTTACATTCCTTCAAGCAATAGAGTACTACGAGGCACCCTTCACCATTGCAGATGGCGTCTATGGCTCTACATTTTTTGTGGCAACGGGGTTTCATGGCCTCCATGTAATTATTGGGTCAACATTTCTGGCAGTCTGCCTCTTACGACAAGTCCAATTCCACTTTACATCAGAACATCACTTCGGATTTGAAGCTGCAGCATGATACTGACACTTTGTAGACGTAGTCTGACTATTCTTATATATCTCTATCTACTGATGAGGCTCATATCTTTCTAGTATTAAAAAGTACAAGTGACTTCCAATCACTCAGTCTTGGTTAGACTCCAAGGAAAGATAATGAACTTAGTACTAGTCATTATTTGCATCTCATTAGCCCTCGCCGCACTGCTCGCAACTGTTTCATTTTTTCTCCCACAAATAACCCCTGATTATGAGAAACTCTCACCGTATGAGTGCGGCTTTGATCCAGTGGGATCCGCCCGTTTGCCATTCTCCATTCGCTTTTTTCTAGTCGCAATCCTATTTCTCCTCTTCGACTTAGAAATTGCCTTACTTCTTCCCCTTCCCTGAGGAGACCAACTCCCCTCCCCTCTGACAACTTTCTTCTGAGCTTCTGCTATCCTTATACTACTAACTCTAGGGTTAATCTATGAATGACTTCAAGGGGGCCTAGAGTGGGCAGAATAGGTACTTAGTTTAATAAAAACATTTGATTTCGGCTCAAAAACTTATGGTTTAAGTCCATATTTACCTGATGACCTTAACTCACTATGCATTCTCGTCAGCCTACTTTGTCAGCTTCATGGGTCTAATTTTTTACCGAAAGCATCTTCTCTCCGCCTTACTTTGCTTAGAAGCCATAATACTTATTCTTTTTATTTCACTATGCCTGTGAGGCCTAGTCTTAGCCTCAAGTGCATTTTCGGCAGGCCCAATGATCTTACTTGCTTTCTCAGCATGTGAAGCAAGTGCAGGCCTAGCACTGCTTGTAGCAATAGCTCGAACCCACGGGACTGACCGTTTAAAAAACCTTAGCCTACTCCAATGTTAATAATTCTTATTCCTACTGTTATGCTTCTACCCACAATCTGACTAAGCCCCGCTAAATACCTGTGGTCCTCAGCACTTGGCCATAGCATAATAATTGCTCTTATAAGCCTCTCCTGACTTAGCCTCCCGGGGGAGGTTGGCTGATCTTTCCTTAACACTTTTATAGCAACAGACCCTCTCTCTACCCCCCTTCTCGTACTTACTTGCTGACTTCTACCCTTAATAATTCTTGCGAGCCAAAACCATATAGCCCAGGAACCTACCAATCGCCAGCGAACCTATATCTCTCTCCTTACTTCCCTTCAAATCTTCTTAATCTTAGCATTTGGAGCAACCGAGATAATTATGTTCTATATTATATTTGAAGCGACCTTAATTCCCACACTCGTAATTATCACACGATGAGGGAACCAAACAGAGCGATTAAACGCAGGTATTTACTTTTTATTTTATACCTTAGCCGGCTCTTTACCACTACTAGTGGCCCTCCTTCTACTTCAGACCTCGACAGGAACTCTTTCTTTTCTAACCACTCAATTTTTTCCCCCCTTACAACTGCATACAGAAGCAAGTAAATTCTGGTGGGCGGGCTGTTTACTAGCATTCTTAGTAAAAATGCCGCTATATGGGGCACACCTTTGACTTCCAAAAGCTCACGTCGAAGCCCCCATCGCCGGATCAATAGTCCTTGCAGCCGTTCTTTTAAAACTAGGGGGTTACGGTATGATACGAGTCATTATTATCTTAGAACCCCTAACGAAACAACTCAGCTACCCCTTTATTGTTCTTGCCCTGTGGGGCGTTGTAATAACTGGCTCAATCTGCCTCCGACAAACAGACCTTAAATCACTAATCGCTTACTCCTCAGTAAGCCACATGGGCCTTGTCGCAGCAGGCATCCTGATCCAAACTCCTTGGGGGTTTACAGGAGCATTAATCCTTATAATTGCCCATGGCTTAACCTCCTCCGCCCTATTCTGTTTAGCCAACACTAACTATGAGCGAACACATAGCCGAACCATACTTTTAGCCCGGGGTCTACAAATGGTCCTTCCTCTTTTAGCAACTTGGTGGTTTCTACTTACCCTCGCCAACCTAGCACTCCCTCCGCTACCCAACCTCATAGGAGAGCTTATGATTATCTCATCCTTGTATAACTGGTCAAACTGGTCTCTAATCCTGACCGGAGCGGGAGTACTAATTACCGCTAGCTACTCTCTTCATATATTCCTAACCACTCAACGCGGCCCTATTACTAACCCCGTCTTGGCAATTGAACCAACCCACACACGAGAACATCTCCTCATAATTCTTCACCTTCTTCCTCTCCTCCTTCTAATTTTAAAACCCTGCTTGATCTGGGGCTGAACAGTTTGTAGGCGTAGTTTAAATAAAGCGCTAGATTGTGATTCTAGAAATAAGAGTTAAACCCTCTTCACCCACCGAGAGGGGTCGCCGTGACAGCAAGAACTGCTAATTCTAGCCCCTTTGGTTAAAATCCGAAGCCCACTCGAACAGGCTTCTAAAGGATAACAGCTCATCCGTTGGTCTTAGGAACCAAAAACTCTTGGTGCAACTCCAAGTAGCAGCTATGCACTTTACAACAATGATTCTCTCCTCAAGCCTAATAACAATTTTCCTTCTTCTCATCCTTCCAGTCCTAGGTACACTAAACCCTAACCCCACGGGGGGCCTGTGAGCCACAAAAAACGTTAAAACGGCAGTTAAGATAGCCTTTTTTGTAAGTCTTTTGCCTCTTTTTATCTTTCTTAATGAAGGGGTAGAGACTGTTATAACAAACTGAAAATGAATAAATACTCTAATGTTTGAAATTAATATCAGCTTTAAATTTGACCTCTACTCCGTAGTATTTACCCCTGTGGCCCTCTACGTAACATGATCAATTTTAGAGTTCGCATCTTGGTATATACACAGTGATCCCAACATAAACCGATTCTTTAAATATCTTCTAATCTTTCTTATTGCTATGGTCGTTCTGGTCACAGCCAACAACATGTTCCAACTATTTATTGGCTGAGAAGGTGTTGGAATTATATCTTTCTTACTTATTGGCTGGTGATTCGGGCGGGCTGACGCCAACACTGCGGCCCTCCAGGCCGTAGTTTATAACCGAGTTGGTGATATCGGCCTTATTCTAGCAATAGCATGAATAGTAGCAAACCTAAACTCATGAGAAATACAACAGCTCTTTTCTATGTCTAAAGGCCATGATATAACCCTTCCTTTATTAGGCCTAGTACTGGCCGCTACCGGAAAGTCCGCCCAATTTGGACTTCACCCCTGGCTCCCCTCAGCCATAGAAGGTCCAACACCGGTCTCTGCCCTCCTGCACTCCAGCACCATGGTGGTTGCTGGTATTTTTCTTCTTATTCGCCTCAGCCCCTTAATGCAAGAAAGCCCATTAATTCTCTCAACATGCCTTTGCCTGGGGGCCCTAACTACCATCTTTACTGCAACATGTGCCCTTACCCAAAATGACATTAAAAAAATCGTTGCATTTTCTACATCAAGTCAATTAGGACTAATAATAGTTACCATCGGACTAGGCCAGCCCCAGCTCGCCTTTCTTCATATCTGCACCCACGCCTTCTTTAAAGCAATACTTTTCTTATGTTCCGGCTCCATCATTCATAGCCTTAATGATGAGCAAGATATCCGAAAAATAGGCGGACTTCACAAGGTGCTTCCACTGACCTCTTCTTGTTTAACCATTGGCAGCCTAGCTCTAACAGGAGTCCCCTTTTTAGCAGGCTTCTTTTCCAAAGACGCCATCATTGAAGCTATAAATACATCCTACCTTAACGCCTGAGCCCTAATTTTAACGCTTCTAGCTACATCATTCACCGCAGTTTACAGTCTCCGAGTCGTATTCTTTGCCTCTATGGGCCACCCGCGTTTTAATCCAGTCTCCCCAATTAATGAAAATAACCCTACAGTGATAAACCCCATTAAACGACTCGCTTGGGGAAGCATTTTGGCAGGGTTGCTAATTACGACCAATATTGTTCCACTTAAAACCCCCGTTCTAACCATGCCTTTCACCTTAAAAATGGCCGCACTGACTGTAACAATTATAGGACTACTCACAGCCTTAGAACTAGCGTCTCTCACGTCCCAACAATTTAAAATCAAACCCTTATCTTCTACTCACCACTTCTCAAATATATTAGGATTTTTCCCCAGTGTAGTCCATCGACTAGTCCCAAAAACTGGCCTGATTCTAGGACAACTAGTTGCCAATCAGACAGTTGACCAAACCTGACTGGAGAAAACAGGACCAAAAATAGTAACCTCCGTTAACCTTCCAATAGCTACTTCAATTAGCAGCCTACAGCAGGGTGTAATTAAGACCTACTTCTTATTATTTTTCTTCACCATAATACTGGCAATTCTCATCTTTGTCGTCTAACTGCCCGTAAGGTCCCCCGACTTAGCCCCCGAGTTAACTCTAGAACTACGAAAAGCGTCAGTAATAAAACTCATCCCCCAAGCATTAAAACTCCTCCTCCTGAAGAATATATCAGAGCAACCCCACCGAAATCCCCCCGAAAGAGCATGAATTCACTAAACTCGTCAGCAGTTATCCATGACCCCTCATACCAGCCCTCAGAGAAAAAAACAGAGATAGACGCGACCAGGAACACATATACTGACATAAGAAGCAAAACGGGTCAACTTCCCCACCCCTCAGGATAAGGCTCCGAAGCCAGCGCTGCTGAGTACGCAAACACAACTAACATCCCACCTAAATAGATCAAAAACAAAATCAGAGATAAAAATGAACCCCCATGCCCTACTAAAATGCCACAGCCCATTCCTGCTACTGTAACAAGACCTAAAGCAGCAAAGTAGGGTGACGGGTTCGAGGCCACGGCCGCTAGACCTAAGACCAAACCAACTAATAATAAATAAGTCATATAAACCATAATTCTTGCCAGGATTTTAACCAGGGCCTGCGACTTGAAAAACCACCGTTGTACTCAACTACAAGAACCTAATGGCCAATCTTCGAAAAACCCATCCCCTATTAAAAATCGCAAACGATGCCCTCGTTGATCTCCCAGCCCCGTCGAACATTTCAGTTTGATGAAACTTCGGGTCTCTTCTAGGACTTTGTTTGGCCGCCCAAATCGTTACGGGCCTTTTCCTTGCAATACATTATACATCAGACATTGCCACAGCATTTTCATCTGTAGCACATATTTGTCGTGATGTCAACTACGGCTGACTAATCCGGAACATGCATGCAAACGGTGCTTCCTTTTTCTTCATTTGCATCTACCTGCACATCGGACGGGGCTTGTATTATGGATCATACTTATATAAAGAGACATGAAATGTAGGTGTTGTCCTTCTCCTCCTAGTGATAATGACTGCTTTCGTAGGCTACGTCCTACCCTGAGGACAAATGTCATTCTGAGGGGCCACCGTCATTACCAACCTTTTATCAGCCATTCCCTACATTGGAAACGCCCTAGTTCAATGGATCTGAGGCGGATTTTCAGTAGACAACGCCACCCTTACCCGGTTCTTTGCCTTCCATTTCCTCCTCCCCTTTGTAATTGCTGCTGCTACAGTTGTACATCTTATTTTCCTGCACGAGACAGGGTCGAATAACCCAACGGGTTTAAACTCAGACTCTGACAAAGTGTCTTTTCACCCCTACTTTTCTTATAAAGATCTTCTAGGCTTTGCTGCCCTGCTAGTAGCCCTTATCTCTTTAGCCCTCTTCTCCCCTAATCTACTTGGAGACCCTGATAACTTTACCCCTGCTAATCCTTTAGTAACTCCACCTCACATCAAACCTGAGTGATACTTCCTGTTCGCTTACGCCATTCTACGATCCATCCCAAACAAGCTTGGTGGGGTTCTAGCCCTATTAGCCTCTATTCTAGTTCTCTTTCTTGTCCCTATCCTGCACACATCAAAACAACGAAGCCTAACATTCCGACCCCTAACCCAATTCCTCTTCTGATTGCTAGTCGCCGATGTAATAATTTTAACCTGAATCGGAGGTATGCCTGTAGAACACCCTTACATTATCATTGGGCAAGTCGCATCCTTCATTTATTTTTCCCTTTTTCTAGTTATGGCGCCTATGGCCGGCCTACTAGAAAACAAAGTCTTAAAATGACAATGCATTAGAAGCTCAGATGAGAGAGCACCGGTCTTGTAAGCCAGAGGTCGAAGGTTCAAGTCCTTCCTAGTGCTCAGAGAGAAGGGATTCTAACCCCTGCCCCTGGCTCCCAAAGCCAGAATTCTTAGCTAAACTACTCGCTGATTTTCATATACCAGTTTTGCAATCCAGAGCGCATCACTTTTGCCACCAGCGTTAAATTAACGTTGCACAAACGTTGCATCAGCACCCCATGGACACTAAATGACGCGAGGGCGTTAAATAGACACCCTCTACCTCTAGCACCCTTTTAACGATTTCACTTTTTTTTTTTTTTTTGTTTAACGATTACGTTTTTTTTTGCGTTCCCGGACTCTGCCAGATTTCGACCGAAGACTGCCAGAATCCGCTCAAAATCCGCTCAAATACCAATATGTATTATCCCCATAAGTGGTTTAAACCATTTTTGCCTAGTACACACTGACCATGCAAGTCAATTATATTTACCCCGCGCTCCAGGCCGCAGTACATACACCTACAGTTGGTGTATTTAGCACAAGTGTGCCTCAGCTAGTTTCAAGTCACCCACATCCTTCCTTTAATTGTTACTTAATGTAGTAAGAGCCCACCATCAGTTGATTCCTTAATGTCAACGGTTCTTGAAGGTGAGGGACAAAAATCGTGGGGGTTTCACCTCTTGAATTATTCCTGGCATTTGGCTCTACATCTCAAGGCCATACATTTCTCGTCTCTCACACTTTCACTGGCCCTGACATTGGTTAATGGTGGAGTACATACTCCTCGTTACCCCCCATGCCGGGCGTTCTTTCTAATGGACAACGGGTTTTCCTTTTTTTTTCCTTTTCACTTGGCATTTCACAGTGCATACAAACCTTGTTGACAAGGTTGAACATTTAGAAATCGGCCGCAAAGAATATTGGTGAGTTATTTAAAGATATTAACAGATGAATTGCATAACTGATATCAAGAGCATAAATAACCAAATGAAACTAGGAACGTTTCTATAATATGACCCCCCGGCTTCCGCGCGTCAAACCCCCCTACCCCCCTAAACTAGTAAGAAGGCTATTATTCCTGCAAACCCCCCGGAAACAGGAAACCCCCTACTAGCATTTTAGCCCGCCCAAATTTGTGTGTATTTACATTATTTGTAATATTGCAAAA

>JWM 2

GCTAGTGTAGCTTAACTAAAGCATAACACTGAAGATGTTAAGACAAACCTTAGATTGGTTTCACGAGCACAAAAGTTTGGTCCTGACTTTACTATCAACTTTAGCTAAACTTACACATGCAAGTATCCGCAATCCCGTGAGAATGCCCTACAGTTTCCTTAAAGGAAACAAGGAGCTGGTATCAGGCTCAATTACTCCCGCCCATGACACCTTGCTTAGCCACACCCCCAAGGGAACTCAGCAGTGATAGACATTAAGCAATAAGTGAAAACTTGACTTAATTAAAGCTAAGAGAACCGGTTAAACTCGTGCCAGCCACCGCGGTTATACGAGCGGTTCGAGCTGATAGACTACGGCGTAAAGCGTGGTTAATAAGAATAAAACTAAAGTCGAATGTTTTCAAAGCTGTTATACGCACTCGAAAATTAGAAGACCAGAAACGAAAGTGACTTTAACCCTATGAACCCACGAAAACTATGAAACAAACTGGGATTAGATACCCCACTATGCATAGCTGTAAACTTTGATGAGCCATTACATTATCATCCGCCTGGGTACTACGAGCATCAGCTTAAAACCCAAAGGACTTGGCGGTGCTTTAGACCCACCTAGAGGAGCCTGTTCTAGAACCGATAACCCCCGTTAAACCTCACCCTCTCTTGTTTTTCCCGCCTATATACCGCCGTCGTCAGCTTACCCTGTGAAGGTCTAATAGTAAGCACAACCAGTTATACTCAAAACGTCAGGTCGAGGTGTAGCATATGAGAGGGGAAGAAATGGGCTACATTCCTTGTTTCAAGGAAAACGGATAACATAATGAAAGGTACGTTAGAAGGAGGATTTAGCAGTAAGCAGCAAATAGAGTGTTCTGCTGAAACTGGCCCTGAAGCGCGCACACACCGCCCGTCACTCTCCCCAACTCCGAGTTAAAAACCATATATAAACCTTTGAAGGAACAAAGGGGAGGCAAGTCGTAACATGGTAAGTGTACCGGAAGGTGCACTTGGATAAATCAGAGTATAGCTAAGAAAGAAAAGCATCTCCCTTACACCGAGAAGTCATCCGTGCAAATCGGATTACCCTGACTCTAACAAGCTAGCCCAAAACCTTAACTTAAAAATCAAATATTTCTAGTAATTAATAAACCAAACACATTAAATAAATCATTTTTCCCCCTGAGTATGGGAGACAGAAAAGGATAAAGGAGCTATAGACAAAGTACCGCAAGGGAAAGCTGAAAGAGAAATGAAACAAACCAGTAAAGAAGAACAAAGCAGAGATTAACCCTTGTACCTTTTGCATCATGAATTAGCCAGTTTAATCAAGCAAAGAGCACTGTAGTTTGAACCCCCGAAACTTAGTGAGCTACTTCAAGACAGCCTATGAAATAGGGCAAACCCGTCTCTGTGGCAAAAGAGTGGGAAGATCTTCAAGTAGAGGTGACAGACCTATCGAACTAAGTTATAGCTGGTTGCTCGTGAAATGAATAGAAGTTCAGCCTTTTGCTTTCTAAATTTCGATTTAGCACTACTTAGCCTAAATGACTAGAAAACAAAAGAGTTAGTCAAAGAGGGTACAGCCTGTTTGATAAAAGATACAACTTTACTAGGAGGATAAGAATCATAATTTTAAAGGTTTAATGCCCAGGTGGGCCTAAAAGCAGCCACCCTAATCAATAGCGTTAAAGCTTAAGCATAAAACACACCTACAATTCTGATAAATCAGTTTTAATCCCCTAAAGTTAACGAGCTATTTCATACCTTATGAAAGAAATTATGCTAGTATGAGTAATAAGAAGTTACGAACTTCTCCCTGCACACGTGTAAATCGGAACGGACAAACCACCGAATCTTAACGGCCCCAGTCAAAGAGGGGATGTCGGATAAAAAAAAGAACAAGAAACTCCCGACAAAACCACCGTTAACCCCACACCGGAGTGCTCCCTGGGAAAGACAAAAAGGGACAGAAGGAACTCGGCAAATATGCTCAAGCCTCGCCTGTTTACCAAAAACATCGCCTCTTGTAAAAGTTAAATAAGAGGTACCGCCTGCCCTGTGACTAGTAGTTTAACGGCCGCGGTATTTTGACCGTGCAAAGGTAGCGCAATCACTTGCCTTTTAAATGAAGGCCTGTATGAATGGCACGACGAGGGCTTAACTGTCTCCTCTCCCTAGTCAATGAAATTGATCTCCCCGTGCAGAAGCGGGGATAATAACATAAGACGAGAAGACCCTGTGGAGCTTTAGACTATGAGCAGACCATGTCAAGAATAACAAACAAGTAAATTAAACAGATTGGTCCCTGCTTCTCTGTCTTTGGTTGGGGCGACCGCGGGATAATAAAAAGCTCCCACGAGGATTGAGAACCCTTATCTTATAACCAAGAGCTTCTCCTCTAAGTAACAGAACATCTGACCTTAATGATCCGGCCTGGCCGATCAACGGACCGAGTTACCCCAGGGATAACAGCGCAATCCTCTTTTAGAGTCCATATCGACAAGAGGGTTTACGACCTCGATGTTGGATCAGGACATCCTAATGGTGCAGCCGCTATTAAGGGTTTGTTTGTTCAACAATTAAAGTCCTACGTGATCTGAGTTCAGACCGGAGTAATCCAGGTCAGTTTCTATCTATGACGTACTCTCTTCTAGTACGAAAGGACCGAAGAAAGAAGGCCTATGAAAAGTTATGCCTTAGTCTCACCTTATGAAGAAAACTAAATAAGACAAGAGGTTACACCCCTTAGTCATAGAAAATGACATGTTAAGGTGGCAGAGCCCGGATATTGCAAAAGACCTAAGCCCTTTCCACAGAGGTTCAATTCCTCTCCTTAACTATGTTCTCAACAATATTAAGCTTCATTATTAATCCCCTAATTGTTATGGTTTTTGTTTTGCTGGCAGTAGCCCTCTTAACCTTGGTAGAGCGTAAAGTGCTAAGCTACATGCAACTTCGTAAAGGCCCAAATGTTGTTGGCCCTTACGGCCTTTTGCAACCCTTCGCTGATGGCTTGAAACTTTTCATGAAAGAGCCCGTCCGACCCTCCACCTCCTCGCCCGCCTTATTCTTAATTACCCCTATTATAGCCCTTACCTTAGCCCTAACCCTCTGAGCCCCCCTTCCTATGCCTTTTCCCATCACCGACCTAAACTTAGGCATTTTATTTATTTTAGCACTATCGAGCCTGGCAGTATATTCTATTCTTGGCTCCGGATGGGCCTCCAATTCTAAATATGCATTGATTGGTGCTCTTCGAGCAGTCGCCCAAACCATCTCTTATGAAGTGAGCTTGGGCCTTATTCTTCTTAACACAATTGTCTTTACAGGGGGTTTTACTCTTCAAACCTTCAGCACCGCACAAGAAGCCACCTGATTACTTCTACCAGCATGACCACTAGCAGCCATGTGATATATCTCCACACTCGCGGAAACTAACCGGGCCCCTTTCGACTTAACTGAAGGAGAGTCCGAACTAGTGTCTGGCTTCAACGTAGAGTATGCCGGCGGACCTTTTGCCCTTTTTTTTCTGGCAGAATACGGTAACATTTTACTTATAAATACCCTCTCAGCAGTACTATTTCTAGGCTCTTCAACCTACCACAGCTTTCCAGAACTAACCGCGACCTTATTAATGCTTAAAGCCACCCTCCTTTCAGTCGTATTTTTATGAGTGCGAGCATCTTACCCTCGGTTTCGATACGATCAACTAATGCATTTAATTTGAAAAAACTTTTTACCTCTGACCCTAGCACTAGTTATTTGACACCTTTCTCTTCCGATCACGTTGAGCGGCCTTCCCCCTCAACTTTAACTCAGGAAATGTGCCTGAAAAAGGATCACTTTGATAGGGTGAATAATGAGGGTTAAAGCCCCTCCATCTCCTTAGAAAGAAGGGGTTTGAACCCTACCTGAAGAGATCAAAACTCTTAGTGCTTCCACTACACCACTTCCTAGTAAAGTCAGCTAATAAAAGCTTTTGGGCCCATACCCCAAATATGTTGGTTAAAATCCTTCCTTTGCTAATGAATCCTTACGTCCTTTCAATTCTACTTATAGGATTAGGCCTCGGCACTACAGTCACATTCGCTAGCTCACACTGACTATTAGCATGAATAGGCCTTGAAATAAATACCCTCGCCATTTTGCCGTTAATAGCACAACATCACCACCCCCGAGCCGTTGAAGCCACCACCAAGTATTTTTTAATTCAATCGGCAGCCGCAGCAACCATCTTATTTGCCAGCTCAACAAACGCCTGACTTTCGGGCCAGTGGGACATCATAAGTATTAATCACCCTCTTCCAACCGTCATAATTACAGTCGCTCTGTCCTTAAAACTAGGCTTGGCCCCTCTTCACGCGTGACTTCCCGAAGTAATTCAAGGCCTGGACTTAACTACGGGCTTAATCCTCTCCACATGACAAAAACTCGCACCCTTTGCCCTCCTCGTTCAAATCTTCCCCGACACCCCCCTTCTCATCACTTCTCTAGGACTTCTTTCAATATTAGTTGGGGGATGAGGGGGTTTAAACCACACACAGCTCCGCAAAGTGCTCGCATATTCTTCGATCGCCCACTTAGGCTGAATAATAGTAATTATGCAATTCTCCACCCCCCTTACAATTCTTGCTTTATCAACATACATTGTCATAACATCATCTACTTTTCTAATCTTTAAACTCCTTAAATCCACAGATATGAACAGCCTGGCAACATCTTGAGCTAAAACCCCCTCCATTACAGCCCTAGCACCTTTAGTGCTATTATCCTTAGGCGGACTCCCTCCCCTCTCGGGCTTTATGCCAAAATGACTAATTATTCAGGAGTTAACTAAGCAAGACCTAGCCCTAGTTGCGACCTTAGCCGCCCTCTCTGCGCTACTCAGCCTTTTCTTTTACCTACGCATTTGTTACTCCCTCACATTTACCTCCTCTCCTAATAATCTCATGGGAACACCCCCCTGACGACTAGTAACAAAGCAAGTATCACTTCCCCTGGCTATGACAACCGCCCTCTCTATTCTCCTACTCCCGGTTACCCCTGCAATCTTATCAGTAGTTCTCCCCTTGTAAAGAGGCTTAGGATAGTATTAAGACCAAGGGCCTTCAAAGCCCTAAGCGGGAGTGAAAGCCCCCCAGCCTCTGTAAGACCTACGGGACACTAACCCACATCTTCTGTATGCAAAACAGACACTTTAATTAAGCTAAAGCCTTCCTAGGTGGGTAGGCCTCGATCCTACAATCTCTTAGTTAACAGCTAAGCGCCTAAACCAGCGGGCATCCATCTACCTTTCCCCCGCCTTGCCGAAAAAAAAAGGCGGGGGAAAGCCCCGGCAGGGTATTAGCCTGCTACTTAAGATTTGCAATCTAATGTGTTAACACCTCGGAGCTGGTAAGAAGAGGACTTTAACCTCTGTCTATGGGGCTACAATCCACCGCTAAACGCTCAGCCACCTTACCTGTGGCAATCACACGTTGATTTTTCTCAACTAATCACAAAGACATCGGCACCCTATATCTAATCTTTGGTGCCTGGGCGGGAATAGTAGGGACGGCCTTAAGTCTACTCATTCGGGCAGAATTAAGTCAACCAGGCTCCCTATTAGGGGACGACCAGATCTATAACGTAATTGTAACTGCACATGCTTTCGTAATAATTTTCTTTATAGTAATGCCAATTATAATTGGAGGGTTCGGCAACTGATTAATTCCTTTAATGATCGGAGCTCCCGACATGGCCTTCCCCCGGATAAATAATATAAGCTTTTGACTCCTGCCCCCTTCTTTCCTTCTATTATTGGCCTCATCTGGTGTAGAAGCTGGTGCCGGGACAGGATGAACCGTATATCCCCCCTTGTCCGGTAATTTGGCACACGCAGGGGCCTCCGTAGATTTAACCATTTTCTCTCTTCACCTGGCCGGAATCTCTTCTATTCTAGGGGCCATTAATTTCATTACAACTATTATTAATATAAAACCTCCAGCCATTTCCCAATATCAAACCCCTTTATTTGTCTGAGCTGTTCTAATTACCGCAGTATTACTCCTACTCTCTCTTCCTGTTCTAGCTGCAGGTATCACTATGCTTCTCACAGATCGAAACCTAAATACAACATTTTTCGACCCCGCAGGAGGGGGGGACCCCATTCTTTATCAACATTTATTCTGATTCTTTGGGCATCCTGAAGTCTACATTCTGATTTTGCCCGGCTTCGGAATGATTTCTCACATTGTAGCATATTACTCAGGCAAAAAAGAGCCGTTTGGCTACATGGGAATAGTATGAGCTATAATAGCAATTGGCTTACTGGGGTTTATCGTATGAGCCCATCATATGTTCACTGTAGGGATGGACGTGGACACTCGAGCTTATTTTACATCCGCCACTATAATTATCGCAATTCCTACAGGAGTCAAAGTGTTTAGTTGACTAGCTACCTTGCATGGGGGCTCAATCAAATGAGAGACCCCTCTGTTATGAGCTCTAGGCTTTATTTTCTTATTTACTGTCGGAGGTTTAACAGGAATTGTTTTAGCCAACTCATCTCTGGACATTATACTTCATGACACATACTATGTTGTAGCCCACTTCCACTATGTCCTCTCTATAGGAGCAGTCTTTGCCATCATGGGGGCATTCGTTCACTGATTCCCCCTATTCTCAGGCTACACCCTTCACAATACGTGAACAAAAATCCACTTCGGAGTTATGTTTGTAGGTGTAAACCTCACCTTTTTCCCTCAGCACTTCTTAGGATTGGCGGGAATACCTCGACGATATTCAGATTACCCTGACGCATACACACTGTGAAATACTATCTCATCCCTGGGGTCACTAATCTCCCTTATTGCTGTAATTATATTCCTATTTATTATCTGGGAAGCATTCGCGGCAAAACGTGAAGTCTTATCAGTTGAACTAACAGCCACAAACGTAGAATGACTGCACGGGTGTCCTCCCCCTTACCATACATTTGAAGAACCTGCATTCGTTCAAATTCAGCAATCCAAATTTTAATCGAGAAAGGAAGGAGTCGAACCCCCATAAACTGGTTTCAAGCCAGCCACATAACCGCTCTGTCACTTTCTTCTCTAAGTTAATAAGATTCTAGTTAAAAGAATAACGCTGCCTTGTCAAGGCAAAATTGTGGGTTAAAGCCCCACGTATCTTGCTTATGGCACATCCATCTCAACTAGGATTCCAAGATGCAGCTTCACCCGTTATAGAAGAACTTCTCCATTTTCATGACCATGCATTAATAATTGTTTTCTTAATCAGCACCCTTGTTCTTTACATTATTGTGGCTATGGTAACCACCAAGCTAACAAATAAGTTCATTCTGGACTCCCAAGAAATTGAAATCATCTGAACCTTACTACCAGCAATTATCCTAATTCTGATCGCCCTACCCTCCCTTCGCATTCTCTACCTCATGGATGAAATCAATGACCCCCACCTCACAATTAAAGCCATGGGACATCAATGATACTGAAGCTACGAATATACGGATTATGAAGACCTGGGGTTCGACTCATATATGGTCCCTACACAAGATCTCGCCCCTGGTCAATTTCGACTACTTGAGACAGACCATCGCATGGTTATTCCTGTTGAGTCCCCCATCCGAGTTCTTGTCTCCGCCGAGGATGTCTTACATTCATGAGCCGTCCCGAGCCTCGGAGTAAAAATGGACGCCGTCCCCGGCCGCCTAAATCAAACAGCCTTCATTACTTCCCGACCAGGTGTGTTTTATGGACAATGCTCAGAGATTTGCGGAGCTAATCATAGCTTTATACCCATTGTAGTGGAAGCTGTTCCTCTAGAACACTTCGAGAACTGGTCTTACCTAATACTTCAAGATGCCTCACCAGGAAGCTAAAAGGGAATAGCATTAGCCTTTTAAGCTAAAAATTGGTGACTCCCGCCCACCCCTGGTGACATGCCTCAGTTGAACCCCGCACCCTGATTTGCTATTATAGTATTCTCGTGGCTAGTTTTCCTAGCCGTTATTCCACCTAAAGTTCTAGCTCACCATTTTCCCAATGACCCCGCCCCACAGAGCGTAAAAAAATCAAAAACAGAGACTTGACCCTGACCATGACTTTAAGCCTCTTTGATCAATTTATGAGCCCTACACTTCTAGGGGTGCCTCTTATCGGACTCGCCCTAACATTGCCATGAGTCCTTTACTTCCGACCCGGTGCCCGATGACTTAATAACCGCTTGATTACCCTTCAATCTATATTCATAAACTGGTTTGTAAAACAAATCTTTCAACCAATAAGCTTAGGCGGACACAAATGGGCCGCTCTCCTCATATCTTTAATACTATTTTTAATTACCTTAAATATGCTGGGCCTGCTGCCTTACACATTTACTCCAACAACGCAGCTGTCACTTAATATAGCCTTTGCAGTTCCACTTTGACTAGCAACTGTCATTATTGGAATACGAAACCAGCCAACACATGCCCTTGGTCACCTTCTCCCTGAAGGAACTCCTACCGCCCTAATCCCGGTTTTAATCGTGATTGAAACAATTAGCCTTTTTATTCGACCCTTGGCCCTCGGTGTTCGACTTACCGCAAACTTGACAGCCGGACACCTTCTAATTCAACTAATTGCAACTGCGGCTTTTGTTCTTTTCCCTATAATACCAACAGTAGCTGCTCTTACCTCTGTCTTACTATTCTTGCTAACCCTGCTAGAAGTCGCCGTGGCCATAATCCAAGCCTATGTATTTGTACTTCTTTTAAGCCTTTATCTACAAGAAAACGTCTAATGGCCCATCAAGCACATGCATATCATATAGTTGACCCAAGCCCTTGACCCCTCACAGGCGCAGTAGCCGCCCTTCTACTTACGTCTGGAACAGCAATCTGAATACACTTTAACTCCACAGTTCTCATGTCCCTTGGACTTGTCCTGCTACTACTAACCATATATCAATGATGGCGAGACATTATCCGAGAGGGTACCTTTCAAGGTCATCATACACCCCCTGTTCAAAAGGGCCTTCGGTACGGGATAATTCTATTTATTACCTCAGAGGTCTTCTTTTTCCTAGGTTTCTTCTGAGCATTTTATCACTCAAGCCTAGCCCCAACCCCCGAACTTGGTGGGTGTTGACCACCTATGGGTATTACAACACTGGACCCCTTTGAAGTCCCCCTTCTCAATACTGCTGTCCTTCTCGCCTCCGGTGTCACGGTCACTTGAGCTCACCATAGTATTATGGAGGGGCAGCGAAAACAAGCAATTCAATCCTTAACACTCACAATTCTACTGGGGTTCTACTTTACATTCCTTCAAGCAATAGAGTACTACGAGGCACCCTTCACCATTGCAGATGGCGTCTATGGCTCTACATTTTTTGTGGCAACGGGGTTTCATGGCCTCCATGTAATTATTGGGTCAACATTTCTGGCAGTCTGCCTCTTACGACAAGTCCAATTCCACTTTACATCAGAACATCACTTCGGATTTGAAGCTGCAGCATGATACTGACACTTTGTAGACGTAGTCTGACTATTCTTATATATCTCTATCTACTGATGAGGCTCATATCTTTCTAGTATTAAAAAGTACAAGTGACTTCCAATCACTCAGTCTTGGTTAGACTCCAAGGAAAGATAATGAACTTAGTACTAGTCATTATTTGCATCTCATTAGCCCTCGCCGCACTGCTCGCAACTGTTTCATTTTTTCTCCCACAAATAACCCCTGATTATGAGAAACTCTCACCATATGAGTGCGGCTTTGACCCAGTGGGATCCGCCCGTTTGCCATTCTCCATTCGCTTTTTTCTAGTCGCAATCCTATTTCTCCTCTTCGACTTAGAAATTGCCTTACTTCTCCCCCTTCCCTGAGGAGACCAACTCCCCTCCCCTCTGACAACTTTCTTCTGAGCTTCTGCTATCCTTATACTACTAACTCTAGGGTTAATCTATGAATGACTTCAAGGGGGCCTAGAGTGGGCAGAATAGGTACTTAGTTTAATAAAAACATTTGATTTCGGCTCAAAAACTTATGGTTTAAGTCCATATTTACCTGATGACCTTAACTCACTATGCATTCTCGTCAGCCTACTTTGTCAGCTTCATGGGTCTAATTTTTTACCGAAAGCATCTTCTCTCCGCCTTACTTTGCTTAGAAGCCATAATACTTATTCTTTTTATTTCACTATGCCTGTGAGGCCTAGTCTTAGCCTCAAGTGCATTTTCGGCAGGCCCAATGATCTTACTTGCTTTCTCAGCATGTGAAGCAAGTGCAGGCCTAGCACTGCTTGTAGCAATAGCTCGAACCCACGGGACTGACCGTTTAAAAAACCTTAGCCTACTCCAATGTTAATAATTCTTATTCCTACTGTTATGCTTCTACCCACAATCTGACTAAGCCCCGCTAAATACCTGTGGTCCTCAGCACTTGGCCATAGCATAATAATTGCTCTTATAAGCCTCTCCTGACTTAGCCTCCCGGGGGAGGTTGGCTGATCTTCCCTTAACACTTTTATAGCAACAGACCCTCTCTCTACCCCCCTTCTCGTACTTACTTGCTGACTTCTACCCTTAATAATTCTTGCAAGCCAAAACCATATAGCCCAAGAACCTACCAATCGCCAGCGAACCTATATCTCTCTCCTTACTTCCCTTCAAATCTTCTTAATCTTAGCATTTGGAGCAACCGAGATAATTATGTTCTATATTATATTTGAAGCGACCTTAATTCCCACACTCGTAATTATCACACGATGAGGGAACCAAACAGAGCGATTAAACGCAGGTATTTACTTTTTATTTTATACCTTAGCCGGCTCTTTACCACTACTAGTGGCCCTCCTTCTACTTCAGACCTCGACAGGAACTCTTTCTTTTCTAACCACTCAATTTTTTCCCCCCTTACAACTGCATACAGAAGCAAGTAAGTTCTGGTGGGCGGGCTGTTTACTAGCATTCTTAGTAAAAATGCCGCTATATGGGGCACACCTTTGACTTCCAAAAGCTCACGTCGAAGCCCCCATCGCCGGGTCAATAGTCCTTGCAGCCGTTCTTTTAAAACTAGGGGGTTACGGTATGATACGAGTCATTATTATCTTAGAACCCCTAACGAAACAACTCAGCTACCCCTTTATTGTTCTTGCCCTGTGGGGCGTTGTAATAACTGGCTCAATCTGCCTCCGACAAACAGACCTTAAATCACTAATCGCTTACTCCTCAGTAAGCCACATGGGCCTTGTCGCAGCAGGCATCCTGATCCAAACTCCTTGGGGGTTTACAGGAGCATTAATCCTTATAATTGCCCATGGCTTAACCTCCTCCGCCCTATTCTGTTTAGCCAACACTAACTATGAGCGAACACATAGCCGAACCATACTTTTAGCCCGGGGTTTACAAATGGTCCTTCCTCTTTTAGCAACTTGGTGGTTTCTACTTACCCTCGCCAACCTAGCACTCCCTCCGCTACCCAACCTCATAGGGGAGCTTATGATTATCTCATCCTTGTATAACTGGTCAAACTGGTCTCTAATCCTGACCGGAGCGGGAGTACTAATTACCGCTAGCTACTCTCTTCATATATTCCTAACCACTCAACGCGGCCCTATTACTAACCCCGTCTTGGCAATTGAACCAACCCACACACGAGAACATCTCCTCATAATTCTTCACCTTCTTCCTCTCCTCCTTCTAATTTTAAAACCCTGCTTGATCTGGGGCTGAACAGTTTGTAGGCGTAGTTTAAATAAAGCGCTAGATTGTGATTCTAGAAATAAGAGTTAAACCCTCTTCACCCACCGAGAGGGGTCGCCGTGACAGCAAGAACTGCTAATTCTAGCCCCTTTGGTTAAAATCCGAAGCCCACTCGAACAGGCTTCTAAAGGATAACAGCTCATCCGTTGGTCTTAGGAACCAAAAACTCTTGGTGCAACTCCAAGTAGCAGCTATGCACTTTACAACAATGATTCTCTCCTCAAGCCTAATAACAATTTTCCTTCTTCTCATCCTTCCAGTCCTAGGAACACTAAACCCTAACCCCACGGGGGGCCTGTGAGCCACAAAAAACGTTAAAACGGCAGTTAAGATAGCCTTTTTTGTAAGTCTTTTGCCTCTTTTTATCTTTCTTAATGAAGGGGTAGAGACTGTTATAACAAACTGAAAATGAATAAATACTCTAATGTTTGAAATTAATATCAGCTTTAAATTTGACCTCTACTCCGTAGTATTTACCCCTGTGGCCCTCTACGTAACATGATCAATTTTAGAGTTCGCATCTTGGTATATACACAGTGATCCCAACATAAACCGATTCTTTAAATATCTTCTAATCTTTCTTATTGCTATGGTCGTTCTGGTCACAGCCAACAACATGTTCCAACTATTTATTGGCTGAGAAGGTGTTGGAATTATATCTTTCTTACTTATTGGCTGGTGATTCGGGCGGGCTGACGCCAACACTGCGGCCCTCCAGGCCGTAGTTTATAACCGAGTTGGTGATATCGGCCTTATTCTAGCAATAGCATGAATAGTAGCAAACCTAAACTCATGAGAAATACAACAGCTCTTTTCTATGTCTAAAGGCCATGATATAACCCTTCCTTTATTAGGCCTAGTACTGGCCGCTACCGGAAAGTCCGCCCAATTTGGACTTCACCCCTGGCTCCCCTCAGCCATAGAGGGTCCAACACCGGTCTCTGCCCTCCTGCACTCTAGCACCATGGTGGTTGCTGGTATTTTTCTTCTTATTCGCCTCAGCCCCTTAATGCAAGAAAGCCCATTAATTCTCTCAACATGCCTTTGCCTGGGGGCCCTAACTACCGTCTTTACTGCAACATGTGCCCTTACCCAAAATGACATTAAAAAAATCGTTGCATTTTCTACATCAAGTCAATTAGGACTAATAATAGTTACCATCGGACTAGGCCAGCCCCAGCTCGCCTTTCTTCATATCTGCACCCACGCCTTCTTTAAAGCAATACTTTTCTTATGTTCCGGCTCCATCATTCATAGCCTTAACGATGAGCAAGATATCCGAAAAATAGGCGGACTTCACAAGGTGCTTCCACTGACCTCTTCTTGTCTAACCATTGGCAGCCTAGCTCTAACAGGAGTCCCCTTTTTAGCAGGCTTCTTTTCCAAAGACGCCATCATTGAAGCTATAAATACATCCTACCTTAACGCCTGAGCCCTAATTTTAACGCTTCTAGCTACATCATTCACCGCAGTTTACAGTCTCCGAGTCGTATTCTTTGCCTCTATGGGCCACCCGCGTTTTAATCCAGTCTCCCCAATTAATGAAAATAACCCTACAGTGATAAACCCCATTAAACGACTCGCTTGGGGAAGCATTTTGGCAGGGTTGCTAATTACGACCAATATTGTTCCACTTAAAACCCCCGTTTTAACCATGCCTTTCACCTTAAAAATGGCCGCACTGACTGTAACAATTATAGGACTACTCACAGCCTTAGAACTAGCGTCTCTCACGTCCCAACAATTTAAAATCAAACCCTTATCTTCTACTCACCACTTCTCAAATATATTAGGATTTTTCCCCAGTGTAGTCCATCGACTAGTCCCAAAAACTGGCCTGATTCTAGGACAACTAGTTGCCAATCAGACAGTTGACCAAACCTGACTAGAGAAAACAGGACCAAAAATAGTAACCTCCGTTAACCTTCCAATAGCTACTTCAATTAGCAGCCTACAGCAGGGTGTAATTAAGACCTACTTCTTATTATTTTTCTTCACCATAATACTGGCAATTCTCATCTTTGTCGTCTAACTGCCCGTAAGGTCCCCCGACTTAGCCCCCGAGTTAACTCTAGAACTACAAAAAGCGTCAGTAATAAAACTCATCCCCCAAGCATTAAAACTCCTCCTCCTGAAGAATATATCAGAGCAACCCCACCGAAATCCCCCCGAAAGAGCATGAATTCACTAAACTCGTCAGCAGTTACCCATGACCCCTCATACCAGCCCTCAGAGAAAAAAACAGAGATAGACGCGACCAGGAACACATATACTGACATAAGAAGCAAAACGGGTCAACTTCCCCACCCCTCAGGATAAGGCTCCGAAGCCAGCGCTGCTGAGTACGCAAACACAACTAACATCCCACCTAAATAGATCAAAAACAAAATCAGAGATAAAAATGAACCCCCATGCCCTACTAAAATGCCACAGCCCATTCCTGCTACTGTAACAAGACCTAAAGCAGCAAAGTAGGGTGACGGGTTCGAGGCCACGGCCGCTAGACCTAAGACCAAACCAACTAATAATAAATAAGTCATATAAACCATAATTCTTGCCAGGATTTTAACCAGGGCCTGCGACTTGAAAAACCACCGTTGTACTCAACTACAAGAACCTAATGGCCAATCTTCGAAAAACCCATCCCCTATTAAAAATCGCAAACGATGCCCTCGTTGATCTCCCAGCCCCGTCGAACATTTCAGTTTGATGAAACTTCGGGTCTCTTCTAGGACTTTGTTTAGCCGCCCAAATCGTTACGGGCCTTTTCCTTGCAATACATTATACATCAGACATTGCCACAGCATTTTCATCTGTAGCACATATTTGTCGTGATGTCAACTACGGCTGACTAATCCGGAACATGCATGCAAACGGTGCTTCCTTTTTCTTCATTTGCATCTACCTGCACATCGGACGGGGCTTGTATTATGGATCATACTTATATAAAGAGACATGAAATGTAGGTGTTGTCCTTCTCCTCCTAGTGATAATGACTGCTTTCGTAGGCTACGTTCTACCCTGAGGACAAATGTCATTCTGAGGGGCCACCGTCATTACCAACCTTTTATCAGCCATTCCCTACATTGGAAACGCCCTAGTTCAATGGATCTGAGGCGGATTTTCAGTAGACAACGCCACCCTTACCCGGTTTTTTGCCTTCCATTTCCTCCTCCCCTTTGTAATTGCTGCTGCTACAGTTGTACATCTTATTTTCCTGCACGAGACAGGGTCGAATAACCCAACGGGTTTAAACTCAGACTCTGACAAAGTGTCTTTTCACCCCTACTTTTCTTATAAAGATCTTCTAGGCTTTGCTGCCCTGCTAGTAGCCCTTATCTCTTTAGCCCTCTTCTCCCCTAATCTACTTGGAGACCCTGATAACTTTACCCCTGCTAATCCTTTAGTAACTCCACCTCACATCAAACCTGAGTGATACTTCCTGTTCGCTTACGCCATTCTACGATCCATCCCAAACAAGCTTGGTGGGGTTCTAGCCCTATTAGCCTCTATTCTAGTTCTCTTTCTTGTCCCTATCCTGCACACATCAAAACAACGAAGCCTAACATTCCGACCCCTAACCCAATTCCTCTTCTGATTGCTAGTCGCCGATGTAATAATTTTAACCTGAATCGGAGGTATGCCTGTAGAACACCCTTACATTATCATTGGGCAAGTCGCATCCTTCATTTATTTTTCCCTTTTTCTAGTTATGGCGCCTATGGCCGGCCTACTAGAAAACAAAGTCTTAAAATGACAATGCATTAGAAGCTCAGATGAGAGAGCACCGGTCTTGTAAGCCAGAGGTCGAAGGTTCAAGTCCTTCCTAGTGCTCAGAGAGAAGGGATTCTAACCCCTGCCCCTGGCTCCCAAAGCCAGGATTCTTAGCTAAACTACTCGCTGATTTTCATATACCAGTTTTGCAATCCAGAGCGCATCACTTTTGCCACCAGCGTTAAATTAACGTTGCACAAACGTTGCATCAGCACCCCATGGACACTAAATGACGCGAGGGCGTTAAATAGACACCCTCTACCTCTAGCACCCTTTTAACGATTTCACTTTTTTTTTTTTTTTTGTTTAACGATTACGTTTTTTTTTGCGTTCCCGGACTCTGCCAGATTTCGACCGAAGACTGCCAGAATCCGCTCAAAATCCGCTCAAATACCAATATGTATTATCCCCATAAGTGGTTTAAACCATTTTTGCCTAGTACACACTGACCATGCAAGTCAATTATATTTACCCCGCGCTCCAGGCCGCAGTACATACACCTACAGTTGGTGTATTTAGCACAAGTGTGCCTCAGCTAGTTTCAAGTCACCCACATCCTTCCTTTAATTGTTACTTAATGTAGTAAGAGCCCACCATCAGTTGATTCCTTAATGTCAACGGTTCTTGAAGGTGAGGGACAAAAATCGTGGGGGTTTCACCTCTTGAATTATTCCTGGCATTTGGCTCTACATCTCAAGGCCATACATTTCTCGTCTCTCACACTTTCACTGGCCCTGACATTGGTTAATGGTGGAGTACATACTCCTCGTTACCCCCCATGCCGGGCGTTCTTTCTAATGGACAACGGGTTTTCCTTTTTTTTTCCTTTTCACTTGGCATTTCACAGTGCATACAAACCTTGTTGACAAGGTTGAACATTTAGAAATCGGCCGCAAAGAATATTGGTGAATTATTTAAAGATATTAACAGATGAATTGCATAACTGATATCAAGAGCATAAATAACCAAATGAAACTAGGAACGTTTCTATAATATGACCCCCCGGCTTCCGCGCGTCAAACCCCCCTACCCCCCTAAACTAGTAAGAAGGCTATTATTCCTGCAAACCCCCCGGAAACAGGAAACCCCCTACTAGCATTTTAGCCCGCCCAAATTTGTGTGTATTTACATTATTTGTAATATTGCAAAA

>JWM 3

GCTAGTGTAGCTTAACTAAAGCATAACACTGAAGATGTTAAGACAAACCTTAGATTGGTTTCACGAGCACAAAAGTTTGGTCCTGACTTTACTATCAACTTTAGCTAAACTTACACATGCAAGTATCCGCAATCCCGTGAGAATGCCCTACAGTTTCCTTAAAGGAAACAAGGAGCTGGTATCAGGCTCAATTACTCCCGCCCATGACACCTTGCTTAGCCACACCCCCAAGGGAACTCAGCAGTGATAGACATTAAGCAATAAGTGAAAACTTGACTTAATTAAAGCTAAGAGAACCGGTCAAACTCGTGCCAGCCACCGCGGTTATACGAGCGGTTCGAGCTGATAGACTACGGCGTAAAGCGTGGTTAATAAGAATAAAACTAAAGTCGAATGTTTTCAAAGCTGTTATACGCACTCGAAAATTAGAAGACCAGAAACGAAAGTGACTTTAACCCTATGAACCCACGAAAACTATGAAACAAACTGGGATTAGATACCCCACTATGCATAGCTGTAAACTTTGATGAACTATTACATTATCATCCGCCTGGGTACTACGAGCATTAGCTTAAAACCCAAAGGACTTGGCGGTGCTTTAGACCCACCTAGAGGAGCCTGTTCTAGAACCGATAACCCCCGTTAAACCTCACCCTCTCTTGTTTTTCCCGCCTATATACCGCCGTCGTCAGCTTACCCTGTGAAGGTCTAATAGTAAGCACAACCAGTTATACTCAAAACGTCAGGTCGAGGTGTAGCATATGAGAGGGGAAGAAATGGGCTACATTCCTTGTTTCAAGGAAAACGGATAACATAATGAAAGGTACGTTAGAAGGAGGATTTAGCAGTAAGCAGCAAATAGAGTGTTCTGCTGAAACTGGCCCTGAAGCGCGCACACACCGCCCGTCACTCTCCCCAACTCCGAGTTAAAAACCATATGTAAACCTTTGAAGGAACAAAGGGGAGGCAAGTCGTAACATGGTAAGTGTACCGGAAGGTGCACTTGGATAAATCAGAGTATAGCTAAGAAAGAAAAGCATCTCCCTTACACCGAGAAGTCATCCGTGCAAATCGGATTACCCTGACTCTAACAAGCTAGCCCAAAACCTTAACTTAAAAATCAAATATTTCTAATAATTAATAAACTAACCACATTAAATAAATCATTTTTCCCCCTGAGTATGGGAGACAGAAAAGGATAGAGGAGCTATAGACAAAGTACCGCAAGGGAAAGCTGAAAGAGAAATGAAACAAACCAGTAAAGAAGAACAAAGCAGAGATTAACCCTTGTACCTTTTGCATCATGAATTAGCCAGTTTAATCAAGCAAAGAGCACTGTAGTTTGAACCCCCGAAACTTAGTGAGCTACTTCAAGACAGCCTATGAAATAGGGCAAACCCGTCTCTGTGGCAAAAGAGTGGGAAGATCTTCAAGTAGAGGTGACAGACCTATCGAACTAAGTTATAGCTGGTTGCTCGTGAAATGAATAGAAGTTCAGCCTTTTGCTTTCTAAATTTCGATTTAGCACTACTTAGCCTAAATGACTAGAAAACAAAAGAGTTAGTCAAAGAGGGTACAGCCTGTTTGATAAAAGATACAACTTTACTAGGAGGATAAGAATCATAATTTTAAAGGTTTAATGCCCAGGTGGGCCTAAAAGCAGCCACCCTAATCAATAGCGTTAAAGCTTAAGCATAAAACGCACCTACAATTCTGATAAATCAGTTTAAATCCCCTAAGGTTAACGAGCTATTTCATACCTTATGAAAGAAATTATGCTAGTATGAGTAATAAGAAGTTACGAACTTCTCCCTGCACACGTGTAAATCGGAACGGACAAACCACCGAATCTTAACGGCCCCAGTCAAAGAGGGGATGTCGGATAAAAAAAAGAACAAGAAGCTCCCGACAAAACCACCGTTAACCCCACACCGGAGTGCTCCCTGGGAAAGACAAAAAGGGACAGAAGGAACTCGGCAAGTATGCTCAAGCCTCGCCTGTTTACCAAAAACATCGCCTCTTGTAAAAGTTAAATAAGAGGTACCGCCTGCCCTGTGACTAGTAGTTTAACGGCCGCGGTATTTTGACCGTGCAAAGGTAGCGCAATCACTTGCCTTTTAAATGAAGGCCTGTATGAATGGCACGACGAGGGCTTAACTGTCTCCTCTCCCTAGTCAATGAAATTGATCCCCCCGTGCAGAAGCGGGGATAATAACATAAGACGAGAAGACCCTGTGGAGCTTTAGACTATGAGCAGACCATGTCAAGAATAACAAACAAGTAAATTAAACAAATTGGTCCCTGCTTCTCTGTCTTTGGTTGGGGCGACCGCGGGATAATAAAAAACTCCCACGAGGATTGAGAACCCTTATCTTATAACCAAGAGCTTCTCCTCTAAGTAACAGAACATCTGACCTTAATGATCCGGCCTGGCCGATCAACGGACCGAGTTACCCCAGGGATAACAGCGCAATCCTCTTTTAGAGTCCATATCGACAAGAGGGTTTACGACCTCGATGTTGGATCAGGACATCCTAATGGTGCAGCCGCTATTAAGGGTTTGTTTGTTCAACAATTAAAGTCCTACGTGATCTGAGTTCAGACCGGAGTAATCCAGGTCAGTTTCTATCTATGACGTACTCTCTTCTAGTACGAAAGGACCGAAGAAAGAAGGCCTATGAAAAGTTATGCCTTAGTCTCACCTTATGAAGAAAACTAAATAAGACAAGAGGTTACACCCCTTAGTCATAGAAAATGACATGTTAAGGTGGCAGAGCCCGGATATTGCAAAAGACCTAAGCCCTTTCCACAGAGGTTCAATTCCTCTCCTTAACTATGTTCTCAACAATATTAAGCTTCATTATTAATCCCCTAATTGTTATGGTTTTTGTTTTGCTGGCAGTAGCCCTCTTGACCTTGGTAGAACGTAAAGTGCTAAGCTACATGCAACTTCGTAAAGGCCCAAATGTTGTTGGCCCTTACGGCCTTTTACAACCCTTCGCTGATGGCTTAAAACTTTTCATGAAAGAGCCCGTCCGACCCTCCACCTCCTCGCCCGCCTTATTCTTAATTACCCCTATTATAGCCCTTACCTTAGCCCTAACCCTCTGGGCCCCCCTTCCTATGCCTTTTCCCATCACCGACCTAAACTTAGGCATTTTATTTATTTTAGCACTATCGAGCCTGGCAGTATATTCTATTCTTGGCTCCGGATGGGCCTCCAATTCTAAATATGCACTAATTGGTGCTCTTCGAGCGGTCGCCCAAACCATCTCTTATGAGGTGAGCTTGGGCCTTATTCTTCTTAACACAATTGTCTTTACGGGGGGTTTTACTCTTCAAACCTTCAGCACCGCACAAGAAGCCACCTGATTACTTCTACCAGCTTGGCCACTAGCAGCCATGTGATATATTTCCACACTCGCGGAAACTAACCGGGCCCCTTTCGACCTAACTGAAGGAGAGTCCGAACTAGTGTCTGGCTTCAACGTAGAGTATGCCGGCGGACCTTTTGCCCTTTTTTTTCTGGCAGAATACGGTAACATTTTACTTATAAATACCCTCTCAGCAGTACTATTTCTAGGCTCTTCAACCTACCACAGCTTTCCAGAACTAACCGCGACCTTATTAATGCTTAAAGCCACCCTCCTTTCAGTCGTATTTTTATGAGTGCGAGCATCTTACCCTCGGTTTCGATACGATCAACTAATACATTTAATTTGAAAAAACTTTTTACCTCTGACCCTAGCACTAGTTATTTGACACCTTTCTCTTCCGATCACGTTGAGCGGCCTCCCCCCTCAACTTTAACTGGGGAAATGTGCCTGAAAAAGGGTCACTTTGATAGGGTGAATAATGAGGGTTAAAGCCCCTCCATCTCCTTAGAAAGAAGGGGTTTGAACCCTACCTGAAGAGATCAAAACTCTTAGTGCTTCCACTACACCACTTCCTAGTAAAGTCAGCTAATAAAAGCTTTTGGGCCCATACCCCAAATATGTTGGTTAAAATCCTTCCTTTGCTAATGAATCCTTACGTCCTTTCAATTCTACTTATAGGTTTAGGCCTCGGCACTACAGTCACATTCGCTAGCTCACACTGACTATTAGCATGAATAGGCCTTGAAATAAATACCCTCGCCATTTTGCCGTTAATAGCACAACATCACCACCCCCGAGCCGTTGAAGCCACCACCAAGTATTTTTTAATTCAATCGGCAGCCGCAGCAACCATCTTATTTGCCAGCTCAACTAACGCCTGACTTTCGGGCCAGTGGGACATCATAAGTATTAATCACCCTCTTCCAACCGTCATAATTACAGTCGCTCTGTCCTTAAAACTAGGCTTGGCCCCTCTTCACGCGTGACTTCCCGAAGTAATTCAAGGCCTAGACTTAACCACGGGCTTAATCCTCTCCACATGACAAAAACTCGCACCCTTTGCCCTCCTCGTTCAAATCTTCCCCGACACTCCCCTTCTCATCACTTCTCTAGGACTTCTTTCAATATTAGTTGGGGGGTGAGGGGGTTTAAACCACACACAACTCCGCAAAGTGCTCGCATATTCTTCGATCGCCCACTTAGGCTGAATGATAGTAATTATGCAATTCTCCACCCCCCTTACAATTCTTGCTTTATCAACATACATTGTTATAACATCATCTACTTTTCTAATCTTTAAACTCCTTAAATCCACAGATATGAACAGCCTGGCAACATCTTGAGCTAAAACCCCCTCCATTACAGCCCTGGCACCTTTAGTGCTATTATCCTTGGGCGGACTCCCTCCCCTCTCGGGCTTTATGCCAAAATGACTAATTATTCAAGAGTTAACTAAACAAGATCTAGCCCTAGTTGCGACCTTAGCCGCCCTCTCTGCGCTACTCAGCCTTTTCTTTTACTTACGCATTTGTTACTCCCTCACATTTACCTCCTCTCCTAATAATCTCATGGGAACACCCCCCTGACGACTAATAACAAAGCAAGTATCACTTCCCCTGGCTATAACAACCTCCCTCTCTATTCTTCTACTCCCGGTTACCCCTGCAATCTTATCAGTGGTTCTCCCTTTGTAAAGAGGCTTAGGATAGTATTAAGACCAAGGGCCTTCAAAGCCCTAAGCGGGAGTGAAAGCCCCCCAGCCTCTGTAAGACCTACGGGACACTAACCCACATCTTCTGTATGCAAAACAGACACTTTAATTAAGCTAAAGCCTTCCTAGGTGGGTAGGCCTCGATCCTACAATCTCTTAGTTAACAGCTAAGCGCCTAAACCAGCGGGCATCCATCTACCTTTCCCCCGCCTTGCCGAAAAAAAAAGGCGGGGGAAAGCCCCGGCAGGGTATTAGCCTGCTACTTAAGATTTGCAATCTAATGTGTTAACACCTCGGAGCTGGTAAGAAGAGGACTTTAACCTCTGTCTATGGGGCTACAATCCACCGCTAAACGCTCAGCCACCTTACCTGTGGCAATCACACGTTGATTTTTCTCAACTAATCACAAAGACATCGGCACCCTATATCTAATCTTTGGTGCCTGGGCGGGAATAGTAGGGACGGCCTTAAGTCTACTCATTCGGGCAGAATTAAGTCAACCAGGCTCCCTATTAGGAGACGACCAGATCTATAACGTAATTGTAACTGCACATGCTTTCGTAATAATTTTCTTTATAGTAATGCCAATCATAATTGGAGGATTTGGTAACTGATTAATTCCTTTAATGATCGGAGCTCCCGACATGGCCTTCCCCCGGATAAATAATATAAGCTTTTGACTCCTGCCCCCTTCTTTCCTTCTATTATTGGCCTCATCTGGTGTAGAAGCTGGTGCCGGGACAGGATGAACCGTATATCCCCCCTTGTCCGGTAATTTGGCACACGCAGGGGCCTCCGTAGATTTAACCATTTTCTCTCTGCACCTGGCCGGAATTTCTTCTATTCTAGGGGCCATTAATTTCATTACAACTATTATTAATATAAAACCTCCAGCCATTTCCCAATATCAAACCCCTTTATTTGTCTGAGCTGTTCTAATTACCGCAGTATTACTCCTACTCTCTCTTCCTGTTCTAGCTGCGGGTATCACTATGCTTCTCACAGATCGAAACCTAAATACAACATTTTTCGACCCCGCAGGAGGGGGGGACCCCATTCTTTATCAACATTTATTCTGATTCTTTGGGCATCCTGAAGTCTACATTCTGATTTTGCCCGGCTTCGGAATGATTTCTCACATTGTAGCATATTACTCAGGCAAAAAAGAGCCGTTTGGCTACATGGGAATAGTATGAGCTATAATAGCAATTGGCCTACTGGGGTTTATCGTATGAGCCCATCATATGTTCACTGTAGGAATGGACGTGGACACTCGAGCTTATTTTACATCCGCCACTATAATTATCGCAATTCCTACAGGAGTCAAAGTGTTTAGTTGACTAGCTACCTTGCATGGGGGCTCAATCAAATGAGAGACCCCCCTGTTATGAGCTCTAGGCTTTATTTTCTTATTTACTGTTGGAGGTTTAACAGGAATTGTTTTAGCCAACTCATCTCTGGATATTATACTTCATGACACATACTACGTTGTAGCCCACTTCCACTATGTCCTCTCTATAGGAGCAGTCTTTGCCATCATGGGGGCATTCGTTCACTGATTCCCCCTATTCTCAGGCTACACCCTTCACAATACGTGAACAAAAATCCACTTCGGAGTTATGTTTGTAGGTGTAAACCTCACCTTTTTCCCTCAGCACTTCTTAGGATTGGCGGGAATACCTCGACGATATTCAGATTACCCTGACGCATACACACTGTGAAATACTATCTCATCTCTGGGGTCACTAATCTCCCTTATTGCTGTAATTATATTCCTATTTATTATCTGGGAAGCATTCGCGGCAAAACGTGAAGTCTTATCAGTTGAACTAACAGCCACAAACGTAGAATGACTGCACGGGTGTCCTCCCCCTTACCATACATTTGAAGAACCCGCATTCGTTCAAATTCAGCAATCCAAATTTTAATCGAGAAAGGAAGGAGTCGAACCCCCATAAACTGGTTTCAAGCCAGCCACATAACCGCTCTGTCACTTTCTTCCCTAAGTTAATAAGATTCTAGTTAAAAGAATAACGCTGCCTTGTCAAGGCAAAATTGTGGGTTAAAGCCCCACGTATCTTGCTTATGGCACATCCATCTCAACTAGGATTCCAAGATGCAGCTTCACCCGTTATAGAAGAACTTCTCCATTTTCATGACCATGCATTAATAATTGTTTTCTTAATCAGCACCCTTGTTCTTTACATTATTGTGGCTATGGTAACCACCAAGCTAACAAATAAGTTCATTCTGGACTCCCAAGAAATTGAAATCATCTGAACCTTACTACCAGCAATTATCCTAATTCTGATCGCCCTGCCCTCCCTTCGCATTCTCTACCTCATGGATGAAATCAATGACCCCCACCTCACAATTAAAGCCATAGGACATCAATGATACTGAAGCTACGAATATACGGATTATGAAGACCTGGGGTTCGACTCATATATGGTCCCTACACAAGATCTCGCCCCTGGTCAATTTCGACTACTTGAGACAGACCACCGCATGGTTATTCCTGTTGAGTCTCCCATCCGAGTTCTTGTCTCCGCCGAGGATGTCTTACACTCATGAGCCGTCCCGAGCCTCGGAGTAAAAATGGACGCCGTCCCCGGCCGCCTAAATCAAACAGCCTTCATTACTTCCCGCCCAGGTGTGTTTTATGGACAATGCTCAGAAATTTGCGGAGCTAATCATAGCTTTATACCCATTGTAGTGGAGGCTGTTCCTCTAGAACACTTCGAGAACTGGTCTTACCTAATACTTCAAGATGCCTCACCAGGAAGCTAAAAGGGAATAGCATTAGCCTTTTAAGCTAAAAATTGGTGACTCCCGCCCACCCCTGGTGACATGCCTCAGTTGAACCCCGCACCCTGATTTGCTATTATAGTATTCTCGTGACTAGTTTTCCTAGCCGTTATTCCACCTAAAGTTCTAGCTCACCATTTTCCCAATGACCCCGCCCCACAAAGCGTAAAAAAATCAAAAACAGAGACTTGACCCTGACCATGACTTTAAGCCTCTTTGATCAATTTATGAGCCCTACACTTCTAGGGGTGCCTCTTATCGGACTCGCCCTAACATTACCATGAGTCCTTTACTTCCGACCCGGTGCCCGATGACTTAATAACCGCTTGATTACCCTTCAATCTATATTCATGAACTGGTTTGTAAAACAAATCTTTCAGCCAATAAGCTTAGGCGGACACAAATGGGCTGCTCTCCTCATATCTTTAATACTATTTTTAATCACCTTAAATATGCTAGGCCTGCTGCCTTACACATTTACTCCAACAACGCAGCTGTCACTTAATATAGCCTTTGCAGTTCCACTTTGACTAGCAACTGTCATTATTGGAATACGAAACCAGCCAACACATGCCCTTGGTCACCTTCTCCCCGAAGGGACTCCTACCGCCCTAATCCCGGTTTTAATCGTGATTGAAACAATTAGCCTTTTTATTCGACCCTTGGCCCTCGGTGTTCGACTTACCGCAAACTTGACAGCCGGACACCTTCTAATTCAACTAATTGCAACTGCGGCTTTTGTTCTTTTCCCTATAATACCTACAGTAGCTACTCTTACCTCTGTCTTACTATTCTTGCTAACCCTGCTAGAAGTCGCCGTGGCCATAATCCAAGCCTATGTATTTGTACTTCTTTTAAGCCTTTATCTACAAGAAAACGTCTAATGGCCCATCAAGCACATGCATATCATATAGTTGACCCAAGCCCTTGACCCCTCACAGGCGCAGTAGCCGCCCTTCTACTTACGTCTGGAACAGCAATCTGAATACACTTTAACTCCACAGTTCTCATGTCCCTTGGACTTGTCCTGCTACTATTAACCATATATCAATGATGGCGGGACATTATCCGAGAGGGAACCTTTCAAGGTCATCATACACCCCCTGTTCAAAAGGGCCTTCGGTACGGGATAATTCTATTTATTACCTCAGAGGTCTTCTTTTTCCTAGGTTTCTTCTGAGCATTTTATCACTCAAGCCTAGCCCCAACCCCCGAACTTGGCGGGTGTTGACCACCTATGGGTATTACAACACTGGACCCCTTTGAAGTCCCCCTTCTCAATACTGCTGTCCTTCTCGCCTCCGGTGTCACGGTCACTTGAGCTCACCATAGTATTATGGAGGGGCAGCGAAAACAAGCAATTCAGTCCTTAACACTCACAATTCTCCTGGGGTTCTACTTTACGTTCCTTCAAGCAATAGAGTACTACGAGGCACCCTTCACCATTGCAGATGGCGTCTATGGCTCTACATTTTTTGTGGCAACGGGGTTTCATGGCCTCCATGTAATTATTGGATCAACATTCCTGGCAGTCTGCCTCTTACGACAAGTCCAATTCCACTTTACATCAGAACATCACTTCGGATTTGAAGCTGCAGCATGATACTGACACTTTGTAGACGTAGTCTGACTATTCTTATATATCTCTATCTACTGATGAGGCTCATATCTTTCTAGTATTAAAAAGTACAAGTGACTTCCAATCACTCAGTCTTGGTTAGACTCCAAGGAAAGATAATGAACTTAGTACTAGTCATTATTTGCATCTCATTAGCCCTCGCCGCACTGCTCGCAACTGTTTCATTTTTCCTCCCACAAATAACCCCTGATTATGAGAAACTCTCACCGTATGAGTGCGGCTTTGATCCAGTGGGGTCCGCCCGTTTGCCATTCTCCATTCGCTTTTTTCTAGTCGCAATCCTATTTCTCCTCTTCGACTTAGAAATTGCCTTACTCCTTCCCCTTCCCTGAGGGGACCAACTCCCCTCCCCTCTGACAACTTTCTTCTGAGCTTCTGCTATCCTTATGCTACTAACTCTAGGGTTAATCTATGAATGACTTCAAGGGGGCCTAGAATGGGCAGAATAGGTACTTAGTTTAATAAAAACATTTGATTTCGGCTCAAAAACTTATGGTTTAAGTCCATATTTACCTGATGACCTTAACTCACTATGCATTCTCGTCAGCCTACTTTGTCAGCTTCATGGGTCTAATTTTTTACCGAAAGCATCTTCTCTCCGCCTTACTTTGCTTAGAAGCCATAATACTTATTCTTTTTATTTCACTATGCCTGTGAGGCCTAGTCTTAGCCTCAAGTGCATTTTCGGCAGGCCCAATGATCTTACTTGCTTTCTCAGCATGTGAAGCAAGTGCAGGCCTAGCACTGCTTGTAGCAATAGCTCGAACCCACGGGACTGACCGTTTAAAAAACCTAAGCCTACTCCAATGTTAATAATTCTTATTCCTACTGTTATGCTTCTACCCACAATCTGACTAAGCCCCGCTAAATACCTGTGGTCCTCAACACTTGGCCATAGCATAATAATTGCTCTTATAAGCCTCTCCTGACTTAGCCTCCCAGGGGAGGTTGGCTGATCTTCCCTTAACACTTTTATAGCAACAGACCCTCTCTCTACCCCCCTTCTCGTACTTACTTGCTGACTTCTACCCTTAATAATTCTTGCGAGCCAAAACCATATAGCCCAAGAACCTACCAATCGCCAGCGAACCTATATCTCTTTACTTACTTCCCTTCAAATCTTCTTAATCTTAGCATTTGGAGCAACCGAGATAATTATGTTCTACATTATATTTGAAGCGACCTTAATTCCCACACTCGTAATTATCACACGATGAGGAAACCAAACAGAGCGATTAAACGCAGGTATTTACTTTTTATTTTATACCTTAGCTGGCTCTTTACCACTACTAGTGGCACTTCTTCTACTTCAGACCTCGACAGGAACTCTTTCTTTTCTAACCACTCAATTTTTTTCCCCCTTACAACTGCATACAGAAGCAAGTAAGTTCTGGTGGGCGGGCTGTTTACTAGCATTCTTAGTAAAAATACCGCTATATGGGGCACACCTTTGACTTCCAAAAGCTCACGTCGAAGCCCCCATCGCCGGGTCAATAGTCCTTGCAGCCGTTCTTTTAAAACTAGGGGGTTACGGTATGATACGAGTCATTATTATCTTAGACCCCTTAACGAAACAACTCAGCTACCCCTTTATTGTTCTTGCCCTGTGGGGCGTTGTAATAACTGGCTCAATCTGCCTCCGACAAACAGACCTTAAATCACTAATCGCTTACTCCTCAGTAAGCCACATAGGCCTTGTTGCAGCAGGCATCCTGATCCAAACTCCTTGGGGGTTTACAGGAGCATTAATCCTTATAATTGCCCATGGCTTAACTTCCTCCGCCCTATTCTGTTTAGCCAACACTAACTATGAGCGAACACATAGCCGAACCATGCTTTTAGCCCGGGGCCTACAAATGGTCCTTCCTCTCTTAGCAACTTGATGGTTTCTATTTACCCTCGCCAACCTAGCACTCCCCCCGCTACCCAACCTCATAGGAGAACTTATGATTATCTCATCCCTGTATAACTGGTCAAACTGGTCTTTAGTCCTGACCGGGGCGGGAGTACTAATTACCGCTAGCTACTCTCTTCATATATTCCTAACCACTCAACGCGGCCCTATTACTAACCCCGTCTTGGCAATTGAACCAACCCACACACGAGAACATCTCCTCATAATTCTTCACCTTCTTCCCCTCCTCCTTCTAATTTTAAAACCCTGCTTGATCTGGGGCTGAACAGTTTGTAGGCGTAGTTTAAATAAAGCGCTAGATTGTGATTCTAGAAATAAGAGTTAAACCCTCTTCACCCACCGAGAGGGGTCGCCGTGACAGCAAGAACTGCTAATTCTAGCCCCTTTGGTTAAAATCCGAAGCCCACTCGAACAGGCTTCTAAAGGATAATAGCTCATCCGTTGGTCTTAGGAACCAAAAACTCTTGGTGCAACTCCAAGTAGCAGCTATGCACTTTACAACAATGATTCTCTCCTCAAGCCTAATAACAATTTTTCTTCTTCTCATCCTTCCAGTCCTAGGTACACTAAACCCTAACCCCACGGGGGGCCTGTGAGCCACAAAAAACGTTAAAACGGCAGTTAAGATAGCCTTTTTTGTAAGTCTTTTGCCTCTTTTTATCTTTCTTAATGAAGGGGTAGAGACTGTTATAACAAACTGAAAATGAATAAATACTCTAATGTTTGAAATTAATATCAGCTTTAAATTTGACCTCTACTCCGTAGTGTTTACCCCTGTGGCCCTCTACGTAACATGATCAATTTTAGAGTTCGCATCTTGGTATATACACAGTGACCCCAACATAAACCGGTTCTTTAAATATCTTCTAATCTTTCTCATTGCTATGGTCGTTCTGGTCACAGCCAACAACATGTTCCAACTATTTATTGGCTGAGAAGGTGTTGGAATTATGTCTTTCTTACTTATTGGCTGGTGATTCGGGCGGGCTGACGCCAACACTGCAGCCCTCCAGGCCGTAGTTTATAACCGAGTTGGTGATATCGGCCTGATTCTAGCAATAGCATGAATAGTAGTAAACCTAAACTCATGAGAAATACAACAGCTCTTTTCTATGTCTAAAGGCCATGATATAACCCTTCCTTTATTAGGCCTAGTACTGGCCGCTACCGGAAAGTCCGCCCAGTTTGGACTTCACCCCTGGCTCCCGTCAGCCATAGAGGGTCCAACACCGGTCTCTGCCCTCCTGCACTCTAGCACCATGGTTGTTGCTGGTATTTTTCTTCTTATTCGCCTCAGCCCCTTAATGCAAGAAAGCCCGTTAATTCTCTCAACATGCCTTTGCCTGGGGGCCCTAACTACCGTCTTTACTGCAACATGTGCCCTTACCCAAAATGACATTAAAAAAATTGTTGCATTTTCTACATCAAGTCAATTAGGACTAATAATAGTTACCATCGGACTAGGCCAGCCCCAGCTCGCCTTTCTTCATATCTGCACCCACGCCTTCTTTAAAGCAATACTTTTCTTATGTTCCGGCTCCATTATTCATAGCCTTAATGATGAGCAAGATATCCGAAAAATAGGCGGGCTTCACAAGGTGCTTCCACTGACCTCTTCTTGTCTAACCATTGGCAGCCTAGCTCTAACAGGAGTCCCCTTTTTAGCAGGCTTCTTTTCCAAAGACGCCATCATTGAAGCTATAAATACATCCTACCTTAACGCCTGAGCCCTAATTTTAACGCTTCTAGCTACATCATTTACCGCAGTTTACAGTCTCCGAGTCGTATTCTTTGCCTCTATGGGCCACCCGCGTTTTAATCCAATCTCCCCAATTAATGAAAATAACCCTACAGTGATAAACCCCCTTAAACGACTCGCTTGGGGAAGCATTTTGGCAGGGTTGCTAATTACGACCAATATTGTTCCACTTAAAACCCCCGTTTTAACCATGCCTTTCACCTTAAAAATGGCCGCACTGACTGTAACAATCATAGGACTACTCACAGCCTTAGAACTAGCGTCTCTCACGTCCCAACAATTTAAAATCAAACCCTTATCTTCTACTCACCACTTCTCAAATATATTAGGATTTTTCCCCAGTGTAGTCCATCGACTAGTCCCAAAAACTGGCCTGATTCTTGGACAACTAGTTGCCAATCAGACAGTTGACCAAACCTGACTAGAGAAAACCGGGCCAAAAATAGTAACCTCCGTTAACCTTCCAATAGCTACTTCAATTAGCAGCCTGCAGCAGGGTGTAATTAAAACCTACTTCTTATTATTTTTCTTCACCATAATACTGGCAATTCTCATCCTTGTCGTCTAACTGCCCGTAAGGTCCCCCGACTTAGCCCCCGAGTTAACTCTAGAACTACAAAAAGCGTCAGTAATAAAACCCATCCCCCAAGCATTAAAACCCCTCCTCCTGAAGAATATATCAAAGCAACCCCACCAAAATCCCCCCGAAAGAGCATGAATTCACTAAATTCGTCAGCAGTTATCCATGAACCCTCATACCAGCCCTCGGAGAAAAAAACAGAGATAGACGCGACCAGAAACACATATACTGACATAAGAAGCAAAACGGGTCAACTTCCCCACCCCTCAGGATAAGGCTCCGAAGCCAGCGCTGCTGAGTACGCAAACACAACTAACATCCCACCTAAATAAATCAAAAACAAAATCAGAGATAAAAATGAACCCCCATGCCCTACTAAAATGCCACAGCCCATTCCTGCTACTGTAACAAGCCCCAAAGCAGCAAAGTAAGGTGACGGGTTCGAGGCCACGGCCGCTAGACCTAAAACCAAACCAACTAATAATAAATAAGTCATATAAACCATAATTCTTGCCAGGATTTTAACCAGGGCCTGCGACTTGAAAAACCACCGTTGTACTCAACTACAAGAACCTAATGGCCAATCTTCGAAAAACCCATCCCCTATTAAAAATCGCAAACGATGCCCTCGTTGATCTCCCAGCCCCATCGAACATTTCAGTTTGATGAAACTTCGGATCTCTTCTAGGACTTTGTTTGGCCGCCCAGATCGTTACGGGCCTTTTCCTTGCAATACATTATACATCAGACATTGCCACAGCATTTTCATCTGTCGCACATATTTGTCGTGATGTTAACTACGGCTGACTAATCCGAAACATGCATGCAAACGGTGCTTCCTTTTTCTTCATTTGCATCTACCTGCACATCGGACGGGGCTTGTATTATGGATCATACTTATATAAAGAGACATGAAATGTAGGTGTTGTCCTTCTCCTCCTTGTGATAATGACTGCTTTCGTAGGCTACGTCCTACCCTGAGGACAAATATCATTCTGAGGGGCTACCGTCATTACCAACCTTTTATCAGCCATCCCCTACATTGGAAACGCCCTAGTTCAATGGATCTGAGGCGGATTTTCAGTAGACAACGCCACCCTTACCCGGTTCTTTGCCTTCCATTTCCTCCTCCCCTTTGTAATTGCTGCTGCTACAGTTGTACATCTTATTTTCCTGCACGAGACAGGGTCGAATAACCCAACGGGTTTAAACTCAGACTCTGACAAAGTGTCTTTTCACCCCTACTTTTCTTATAAAGATCTTCTAGGCTTTGCTGCCCTGCTAGTAGCCCTTATCTCTTTAGCCCTCTTCTCCCCTAATCTACTTGGAGACCCTGACAACTTTACCCCTGCTAATCCTTTAGTAACTCCACCTCACATCAAACCTGAGTGATACTTCCTGTTCGCTTACGCCATTCTACGATCCATCCCAAACAAGCTTGGCGGGGTTCTAGCCCTATTAGCCTCTATTCTAGTTCTCTTTCTTGTCCCTATCCTGCACACATCAAAACAACGAAGCCTAACATTCCGACCCCTAACCCAATTCCTCTTCTGACTGCTAGTCGCCGATGTAATAATTTTAACCTGAATCGGAGGTATGCCTGTAGAACACCCTTACATTATCATTGGACAAGTCGCATCCTTCATTTATTTCTCCCTTTTTCTAGTCATGGCGCCTATGGCCGGCCTACTAGAAAACAAAGTCTTAAAATGACAATGCATTAGAAGCTCAGATGAAAGAGCACCGGTCTTGTAAGCCAGAGGTCGAAGGTTCAAGCCCTTCCTAATGCTCAGAGAGAAGGGATTCTAACCCCTGCCCCTGGCTCCCAAAGCCAGGATTCTTAGCTAAACTACTCGCTGATTTTCATACACCAGTTTTGCAATCCAGAGCGCATCACTTTTGCTACCAACGTTAAATTAACGTTGCACAAACGTTGCATCAGCACCCCATGGACACTAAATGACGCGAGGACGTTGAATAGACACCCCCTACCTCTAGCACCCTTTTAACGATTTCACTTTTTTTTTTTTTTTTGTTTAACGATTACGTTTTTTTTTGCGTTCCCGGACTCTGCCAGATTTCGACCGAAAACTGCCAGAATCCGCTCAAAATCCGCTCAAATACCAATATGTATTATCCCCATAAATGGTTTAAACCATTTTTGCCTAGTACACACTGACCATGCAAGTCAATTATATTTACCCCGCGCTCCAGGCCGCAGTACATACACCTACAGTTGGTGTATTTAGCACAAGTGTGCCTCAGCTAGTTTCAAGTCACCCACATCCTTCCTTTAATTGTTACTTAATGTAGTAAGAGCCCACCATCAGTTGATTCCTTAATGTCAACGGTTCTTGAAGGTGAGGGACAAAAATCGTGGGGGTTTCACCTCTTGAATTATTCCTGGCATTTGGCTCTACATCTCAAGGCCATACATTTCTCGTCTCTCACACTTTCACTGGCCCTGACATTGGTTAATGGTGGAGTACATACTCCTCGTTACCCCCCATGCCGGGCGTTCTTTCTAATGGACAACGGGTTTTCCTTTTTTTTTCCTTTTCACTTGGCATTTCACAGTGCATACAGACCTTGTTGACAAGGTTGAACATTTAGAAACCGGCCGCAAAGAATATTGGTGAATTATTTAAAGATATTAACAGATGGATTGCATAACTGATATCAAGAGCATAAATAACCAAATGAAACTAGGAACGTTTCTATAATATGACCCCCCGGCTTCCGCGCGTCAAACCCCCCTACCCCCCTAAACTAGTAAGAAGGCTATTATTCCTGCAAACCCCCCGGAAACAGGAAACCCCCTACTAGCATTTTAGCCCGCCCAAATTTGTGTGTATTTACATTATTTGTAATATTGCAAAA

>JWM 4
[truncated: 550,367 more chars]
