# Supplementary material for: Population genetic characteristics of Hainan medaka with whole-genome resequencing
Source: Front Genet. 2022 Oct 12;13:946006. doi: 10.3389/fgene.2022.946006 (PMC9597887; doi:10.3389/fgene.2022.946006)
Supplement: Supplementary file 12 [file Table2.DOC]

**Table 1 Statistics for SNP annotation results**

| **Category** | **Number of SNPs** |
| --- | --- |
| exonic | 875,581 |
| splice site | 1,853 |
| upstream(2kb) | 1,346,688 |
| downstream(2kb) | 1,171,335 |
| upstream/downstream | 325,702 |
| intergenic | 13,595,255 |
| intronic | 14,750,035 |
| 3’ UTR | 574,339 |
| 5’ UTR | 184,901 |
| 3’UTR/5’UTR | 414 |

| **Category of exon** | **Number of SNPs** |
| --- | --- |
| synonymous | 509,253 |
| nonsynonymous | 355,894 |
| stop-gain | 3,796 |
| stop-loss | 683 |

**Table 2 Functional pathway enrichment for selected genes of SY-medaka**

| **Gene name** | **Gene ID in genome** | **Gene annotation** | **KEGG Path** |
| --- | --- | --- | --- |
| ATF7IP | evm.model.Chr1.310 | Activating transcription factor 7-interacting protein 1 | - |
| txnl4a | evm.model.Chr11.1183 | Thioredoxin-like protein 4A | - |
| KAF6729704.1 | evm.model.Chr11.1185* | - | - |
| trit1 | evm.model.Chr11.1188 | tRNA dimethylallyltransferase | - |
| mycl | evm.model.Chr11.1189 | Protein L-Myc-1b | - |
| mfsd2a | evm.model.Chr11.1190 | Sodium-dependent lysophosphatidylcholine symporter 1-B | - |
| stk3 | evm.model.Chr11.497 | Serine/threonine-protein kinase 3 | - |
| ridA | evm.model.Chr11.498 | 2-iminobutanoate/2-iminopropanoate deaminase | - |
| CYCS | evm.model.Chr11.499 | - | - |
| rpl30 | evm.model.Chr11.500 | 60S ribosomal protein L30 | Ribosome,  Coronavirus disease |
| LAPTM4B | evm.model.Chr11.501 | Lysosomal-associated transmembrane protein 4B | Lysosome |
| RRM2B | evm.model.Chr11.502 | Ribonucleoside-diphosphate reductase subunit M2 | Purine metabolism, Pyrimidine metabolism, Glutathione metabolism, Drug metabolism, p53 signaling pathway, DNA Repair and Recombination Proteins |
| DTNBP1 | evm.model.Chr11.508 | Dysbindin | Membrane trafficking |
| RVE67048.1 | evm.model.Chr11.514* | - | - |
| CEP192 | evm.model.Chr16.896 | Centrosomal protein of 192 kDa | - |
| Ankyrin-3-like isoform X1 | evm.model.Chr19.33 | Ankyrin-3 | - |
| TNPO3 | evm.model.Chr23.720 | Transportin-3 | Nucleocytoplasmic transport, Transfer RNA biogenesis |
| IRF5 | evm.model.Chr23.721 | Interferon regulatory factor 5 | Toll-like receptor signaling pathway, Transcription factors |
| IRF5 | evm.model.Chr23.722 | Interferon regulatory factor 6 | Toll-like receptor signaling pathway, Transcription factors |
| XP_011491718.1 | evm.model.Chr23.723* | - | - |
| CDHR5-like isoform X1 | evm.model.Chr23.724 | Cadherin-related family member 5 | Cell adhesion molecules |
| D(4) dopamine receptor-like | evm.model.Chr23.725 | D(4) dopamine receptor | Neuroactive ligand-receptor interaction,  Dopaminergic synapse, G-Protein Coupled Receptors |
| MBC8529904.1 | evm.model.Chr23.726* | - | - |
| TSPAN12 | evm.model.Chr23.745 | Tetraspanin-12 | - |
| ING3 | evm.model.Chr23.746 | Inhibitor of growth protein 3 | Chromosome and associated proteins |
| CPED1 | evm.model.Chr23.747 | Cadherin-like and PC-esterase domain-containing protein 1 | - |
| BUB1B-like isoform X1 | evm.model.Chr24.309 | Mitotic checkpoint serine/threonine-protein kinase BUB1 beta | - |
| SPINT1-like | evm.model.Chr24.310 | Kunitz-type protease inhibitor 1 | - |
| ARHGAP18 | evm.model.Chr24.798 | Rho GTPase-activating protein 18 | - |
| XP_011490382.1 | evm.model.Chr24.799 | - | - |
| TMEM244 | evm.model.Chr24.800 | Transmembrane protein 244 | - |
| Liprin-beta-2-like isoform X1 | evm.model.Chr3.434 | Liprin-beta-2 | - |
| PPFIBP2 | evm.model.Chr6.215 | Liprin-beta-2 | - |
| KAF6737022.1 | evm.model.Chr6.216* | - | - |
| ARNTL protein 1 | evm.model.Chr6.217 | Aryl hydrocarbon receptor nuclear translocator-like protein 1 | Dopaminergic synapse, Circadian rhythm, Transcription factors |
| GALNT18-like isoform X1 | evm.model.Chr6.223 | Polypeptide N-acetylgalactosaminyltransferase 18 | - |
| SHANK3 isoform X1 | evm.model.Chr6.915_evm.model.Chr6.916_evm.model.Chr6.917 | SH3 and multiple ankyrin repeat domains protein 3 | Glutamatergic synapse |
| ATF7IP2 | evm.model.Chr8.986 | Activating transcription factor 7-interacting protein 1 | - |
| EMP2 | evm.model.Chr8.987 | Epithelial membrane protein 2 | - |

Note: "-" indicates no relevant information. "*" indicates that it cannot be annotated, we provide the access number with the highest score by blastx in the corresponding "gene name" column.

**Table S1 Data output and mapping**

| **Sample** | **Clean reads** | **Clean bases** | **Q20(%)** | **Q30(%)** | **GC Content(%)** | **Average depth** | **Mapping rate** |
| --- | --- | --- | --- | --- | --- | --- | --- |
| shantou_1 | 63,750,859 | 9,501,027,342 | 97.22 | 92.56 | 41.89 | 11.28359 | 98.52% |
| shantou_2 | 63,671,505 | 9,481,820,230 | 97.53 | 93.42 | 42.33 | 11.26078 | 98.80% |
| shantou_3 | 60,080,308 | 8,951,852,653 | 97.66 | 93.62 | 41.52 | 10.63138 | 98.77% |
| shantou_4 | 81,418,259 | 12,103,726,101 | 97.69 | 93.77 | 42.69 | 14.3746 | 98.90% |
| shantou_5 | 69,235,877 | 10,318,237,350 | 97.69 | 93.68 | 41.63 | 12.25412 | 98.77% |
| shantou_6 | 70,153,469 | 10,457,992,590 | 97.51 | 93.20 | 41.87 | 12.4201 | 98.67% |
| shantou_7 | 62,141,781 | 9,253,895,119 | 97.47 | 93.26 | 42.52 | 10.99009 | 98.70% |
| shantou_8 | 62,392,175 | 9,293,156,197 | 97.40 | 93.03 | 42.30 | 11.03672 | 98.68% |
| shantou_11 | 74,916,849 | 11,161,998,588 | 97.66 | 93.59 | 41.43 | 13.25619 | 98.81% |
| shantou_12 | 78,299,503 | 11,602,221,339 | 96.34 | 91.20 | 43.27 | 13.77901 | 98.54% |
| shantou_13 | 63,444,515 | 9,456,044,944 | 97.72 | 93.70 | 40.84 | 11.23017 | 98.83% |
| shantou_14 | 74,351,839 | 11,078,146,096 | 97.59 | 93.44 | 41.81 | 13.15661 | 98.82% |
| shantou_15 | 69,458,433 | 10,356,006,210 | 97.55 | 93.31 | 41.27 | 12.29898 | 98.73% |
| shantou_16 | 77,164,437 | 11,505,283,599 | 97.66 | 93.61 | 41.16 | 13.66388 | 98.84% |
| shantou_17 | 74,240,002 | 11,064,950,938 | 97.54 | 93.37 | 41.92 | 13.14093 | 98.75% |
| jiuwangmiao_1 | 70,726,905 | 10,528,195,361 | 97.56 | 93.48 | 42.81 | 12.50347 | 98.67% |
| jiuwangmiao_2 | 60,368,701 | 8,983,864,851 | 97.59 | 93.55 | 42.61 | 10.6694 | 98.78% |
| jiuwangmiao_3 | 61,737,516 | 9,182,206,838 | 97.41 | 93.15 | 43.39 | 10.90495 | 98.73% |
| jiuwangmiao_4 | 69,626,106 | 10,353,564,621 | 97.36 | 93.06 | 43.40 | 12.29608 | 98.66% |
| jiuwangmiao_6 | 66,157,578 | 9,841,847,165 | 97.24 | 92.70 | 43.16 | 11.68835 | 98.67% |
| jiuwangmiao_7 | 67,126,456 | 9,985,512,011 | 97.59 | 93.59 | 43.22 | 11.85897 | 98.67% |
| jiuwangmiao_10 | 66,133,013 | 9,827,601,233 | 96.34 | 91.17 | 43.27 | 11.67144 | 98.43% |
| jiuwangmiao_11 | 84,564,949 | 12,305,972,098 | 96.81 | 92.17 | 45.16 | 14.61479 | 98.83% |
| jiuwangmiao_12 | 79,908,349 | 11,887,380,475 | 97.27 | 92.77 | 43.22 | 14.11767 | 98.78% |
| jiuwangmiao_13 | 59,039,066 | 8,776,202,412 | 97.32 | 92.93 | 43.43 | 10.42278 | 98.72% |
| jiuwangmiao_15 | 63,671,425 | 9,475,325,966 | 97.56 | 93.48 | 42.78 | 11.25307 | 98.68% |
| jiuwangmiao_16 | 73,849,566 | 10,928,407,744 | 97.06 | 92.53 | 43.98 | 12.97877 | 98.48% |
| jiuwangmiao_17 | 59,174,759 | 8,814,358,063 | 97.17 | 92.54 | 42.45 | 10.46809 | 98.61% |
| jiuwangmiao_19 | 73,607,544 | 10,968,799,723 | 97.56 | 93.38 | 41.81 | 13.02674 | 98.54% |
| jiuwangmiao_20 | 70,181,104 | 10,463,603,327 | 42.61 | 90.67 | 41.92 | 12.42676 | 98.47% |
| gaoqiao_3 | 65,148,334 | 9,703,349,939 | 97.47 | 93.23 | 42.52 | 11.52387 | 98.73% |
| gaoqiao_4 | 69,395,023 | 10,349,727,493 | 95.24 | 88.48 | 41.67 | 12.29152 | 98.35% |
| gaoqiao_8 | 60,275,284 | 8,975,145,741 | 97.70 | 93.77 | 42.25 | 10.65904 | 98.80% |
| gaoqiao_11 | 68,629,459 | 10,219,754,754 | 96.60 | 91.62 | 42.20 | 12.13716 | 98.59% |
| gaoqiao_13 | 75,182,369 | 11,188,415,698 | 95.19 | 88.50 | 43.01 | 13.28756 | 98.22% |
| gaoqiao_14 | 91,313,560 | 13,605,995,982 | 95.06 | 88.24 | 42.80 | 16.15873 | 98.24% |
| gaoqiao_17 | 78,462,670 | 11,673,834,216 | 94.61 | 87.65 | 45.08 | 13.86406 | 97.57% |
| gaoqiao_18 | 77,015,939 | 11,456,621,708 | 97.64 | 93.68 | 42.70 | 13.60609 | 98.36% |
| gaoqiao_20 | 64,676,615 | 9,622,611,413 | 97.41 | 93.16 | 42.64 | 11.42799 | 98.15% |
| gaoqiao_21 | 66,399,245 | 9,888,344,990 | 97.62 | 93.63 | 42.48 | 11.74358 | 98.45% |
| gaoqiao_23 | 77,574,341 | 11,543,215,547 | 97.66 | 93.68 | 42.36 | 13.70893 | 98.42% |
| sanya_2 | 61,855,177 | 9,114,182,337 | 96.56 | 91.53 | 42.68 | 10.82417 | 96.82% |
| sanya_7 | 90,835,595 | 13,385,108,059 | 97.53 | 93.38 | 43.13 | 15.89639 | 97.13% |
| sanya_8 | 64,028,786 | 9,433,652,856 | 97.30 | 92.97 | 43.40 | 11.20358 | 97.23% |
| sanya_9 | 65,509,669 | 9,646,345,580 | 97.17 | 92.62 | 42.57 | 11.45617 | 97.20% |
| sanya_10 | 64,990,002 | 9,568,867,468 | 97.52 | 93.42 | 42.47 | 11.36416 | 97.36% |
| sanya_11 | 62,453,548 | 9,197,193,425 | 97.21 | 92.66 | 42.38 | 10.92275 | 97.26% |
| sanya_12 | 80,843,749 | 11,912,411,884 | 97.35 | 92.84 | 41.66 | 14.14739 | 97.14% |
| sanya_14 | 65,535,721 | 9,660,077,966 | 97.38 | 93.02 | 42.60 | 11.47248 | 97.04% |
| sanya_15 | 68,922,990 | 10,154,254,094 | 97.44 | 93.17 | 42.73 | 12.05937 | 97.34% |
| sanya_16 | 71,916,516 | 10,578,123,324 | 97.12 | 92.73 | 44.86 | 12.56277 | 96.88% |
| sanya_18 | 73,166,555 | 10,776,366,322 | 97.45 | 93.36 | 43.33 | 12.79821 | 97.00% |
| sanya_19 | 77,960,626 | 11,465,359,623 | 97.28 | 92.86 | 42.39 | 13.61647 | 97.21% |
| sanya_20 | 76,297,208 | 11,236,805,219 | 97.53 | 93.38 | 42.24 | 13.34503 | 97.33% |
| sanya_25 | 79,299,724 | 11,690,974,662 | 97.57 | 93.48 | 41.88 | 13.88441 | 97.35% |
| sanya_26 | 73,651,332 | 10,860,439,826 | 97.25 | 92.72 | 42.14 | 12.89805 | 97.24% |

**Table S2 Site information for the medaka field survey**

| **Location Name** | **Longitude(°)** | **Latitude(°)** | **Salinity(‰)** | **Dissolved Oxygen(mg/L)** | **Water  Temperature(℃)** | **pH** | **Serial number  in Fig. 1A** |
| --- | --- | --- | --- | --- | --- | --- | --- |
| Raoping | 117.08 | 23.57 | 22.00±0.36 | 10.44±0.9 | 22.93±0.32 | 8.63±0.12 | 1 |
| Dahao | 116.61 | 23.36 | 12.93±0.29 | 3.30±0.08 | 20.20±0.36 | 7.90±0.00 | 2 |
| Shantou(ST)* | 116.57 | 23.38 | 8.26±0.14 | 4.88±0.17 | 25.23±1.5 | 7.97±0.06 | 3 |
| Conghua | 113.41 | 23.56 | - | - | - | - | 4 |
| Jiuwangmiao(JWM)* | 113.58 | 22.76 | - | - | - | - | 5 |
| Zhonggui | 108.71 | 21.66 | 28.3±0.3 | 4.3 ±1.0 | 21.3±0.2 | 7.7±0.1 | 6 |
| Yamchow | 108.71 | 21.66 | 33.6±0.2 | 13.9±2.5 | 24.9±0.1 | 8.6±0.1 | 7 |
| Gaoqiao(GQ)* | 109.74 | 21.55 | 29.67±0.58 | 16.37±0.58 | 9.58±0.12 | 7.20±0.21 | 8 |
| Huguang | 110.29 | 21.10 | 15.1±8.3 | 5.6 ±1.9 | 24.8±1.9 | 7.3±0.3 | 9 |
| Donghai dao | 110.32 | 21.10 | - | - | - | - | 10 |
| Fucheng | 110.16 | 20.92 | 22.6 ±0.2 | 4.3 ±0.3 | 30.0 ±1.2 | 7.9±0.1 | 11 |
| Leizhou | 109.93 | 20.45 | 25.77±0.32 | 2.11±0.43 | 35.43±0.15 | 7.63±0.06 | 12 |
| Lingao County | 109.57 | 19.86 | 0.14 ±0.1 | 6.43±1.19 | 32.2 ±0.8 | 8.0±0.1 | 13 |
| Chengmai | 109.99 | 19.90 | 12.8 ±0.3 | 5.2 ±0.5 | 40.9 ±0.7 | 8.0±0.2 | 14 |
| Dongzhaigang | 110.58 | 19.95 | 10.2 ±0.5 | 5.6 ±0.5 | 27.5 ±0.2 | 7.8±0.2 | 15 |
| Wenchang | 110.79 | 19.62 | 3.0±1.6 | 4.4 ±1.1 | 27.8 ±0.2 | 7.9±0.3 | 16 |
| Sanya(SY)* | 109.50 | 18.25 | - | - | - | - | 17 |

Note: The salinity, dissolved oxygen and water temperature data in the table were collected at the time of sampling and are for reference only; "-" indicates missing data; "*" indicates populations used for WGS.

**Table S3 Mitochondrial sequences of *Oryzias* from NCBI**

| **Species** | **Accession** | **Length** |
| --- | --- | --- |
| *Oryzias sarasinorum* | AB370891.1 | 16,462 bp |
| *Oryzias luzonensis* | AB498064 | 16,666 bp |
| *Oryzias javanicus* | AB498067.1 | 16,892 bp |
| *Oryzias minutillus* | AB498068.1 | 16,953 bp |
| *Oryzias dancena* | AB498069.1 | 16,863 bp |
| *Oryzias celebensis* | AB498070.1 | 16,190 bp |
| *Oryzias latipes* | AP004421.1 | 16,714 bp |
